# Rapid and efficient genetic engineering of both wild type and axenic strains of *Dictyostelium discoideum*

**DOI:** 10.1371/journal.pone.0196809

**Published:** 2018-05-30

**Authors:** Peggy Paschke, David A. Knecht, Augustinas Silale, David Traynor, Thomas D. Williams, Peter A. Thomason, Robert H. Insall, Jonathan R. Chubb, Robert R. Kay, Douwe M. Veltman

**Affiliations:** 1 MRC Laboratory of Molecular Biology, Cambridge, United Kingdom; 2 Department of Molecular and Cell Biology, University of Connecticut, Storrs, Connecticut, United States of America; 3 Cancer Research UK Beatson Institute Glasgow, Glasgow, United Kingdom; 4 MRC Laboratory for Molecular Cell Biology, University College London, London, United Kingdom; 5 Department of Cell and Developmental Biology, University College London, London, United Kingdom; Université de Genève, SWITZERLAND

## Abstract

*Dictyostelium* has a mature technology for molecular-genetic manipulation based around transfection using several different selectable markers, marker re-cycling, homologous recombination and insertional mutagenesis, all supported by a well-annotated genome. However this technology is optimized for mutant, axenic cells that, unlike non-axenic wild type, can grow in liquid medium. There is a pressing need for methods to manipulate wild type cells and ones with defects in macropinocytosis, neither of which can grow in liquid media. Here we present a panel of molecular genetic techniques based on the selection of *Dictyostelium* transfectants by growth on bacteria rather than liquid media. As well as extending the range of strains that can be manipulated, these techniques are faster than conventional methods, often giving usable numbers of transfected cells within a few days. The methods and plasmids described here allow efficient transfection with extrachromosomal vectors, as well as chromosomal integration at a ‘safe haven’ for relatively uniform cell-to-cell expression, efficient gene knock-in and knock-out and an inducible expression system. We have thus created a complete new system for the genetic manipulation of *Dictyostelium* cells that no longer requires cell feeding on liquid media.

## Introduction

*Dictyostelium discoideum* is a soil-dwelling social amoeba that feeds on bacteria. Numerous related species have been isolated world-wide and can be grouped into 4 clades [1]. *D*. *discoideum* has become a popular model organism to study complex cellular processes such as cell migration, phagocytosis, macropinocytosis and the developmental mechanisms that allow individual amoebae to construct a multi-cellular fruiting body. In more recent years there has been increasing interest in *Dictyostelium* for single cell gene expression studies, as a host for intra-cellular pathogens, allo-recognition and the evolution of cellular altruism, as well as in the unusual three-sex mating system [2–11].

The early pioneering work on development and chemotaxis of *Dictyostelium* was based on wild type strains that could only grow on bacteria. A major advance came in the 1970s with the isolation of axenic strains that can grow on liquid media without bacteria (hence ‘axenic’) [12, 13] due to a greatly increased rate of fluid uptake by macropinocytosis. The axenic strains AX2, AX3 and AX4 all derive from a single wild isolate called NC4 and are the standard laboratory workhorses [14]. Molecular genetic manipulation was developed in these axenic strains, starting with transfection [15], progressing to gene knock-outs [16], REMI insertional mutagenesis [17], marker re-cycling [18] and many others, and culminating in the genomic sequence of AX4 in 2005 [19]. While these strains have been responsible for a dramatic increase in our understanding of *Dictyostelium* biology, there is also the need to be able to work with non-axenic strains. This is for three main reasons.

First, it was assumed that the mutations allowing axenic growth were largely neutral so far as the underlying cell physiology is concerned. Unfortunately, this assumption is only partially correct, because there are differences both between different axenic strains and, between axenic strains and the wild type [14]. Most fundamentally, the key axenic gene, *axeB*, has recently been identified as the RasGAP, NF1, whose biochemical function is to restrain Ras activity [20]. NF1 is deleted in all axenic strains tested, leading to excessive Ras activity and the formation of abundant PIP3 patches in the plasma membrane, which template circular ruffles and macropinosomes [21]. This excessive macropinocytic activity competes with pseudopod formation almost preventing chemotaxis of vegetative cells to folate [22], and the PIP3 patches persisting in starved cells can be confused with pseudopods, confounding work on chemotaxis to cyclic-AMP. Since Ras and PIP3 have been placed at the centre of many gradient-sensing schemes, it is necessary to test key chemotaxis results with wild type cells. Second, the NF1 mutation, which so greatly increases the rate of macropinocytosis, makes axenic cells very valuable for investigating fundamental aspects of this process [23]. However, mutation of genes required for macropinocytosis, such as in the PI3-kinases [24], almost abolish axenic growth, making it necessary to manipulate these cells through growth on bacteria. Finally, the increasing use of Dictyostelids to explore questions in the evolution of multicellularity [1, 25], kin recognition [4] and altruistic cellular behaviour [6, 7] all would be facilitated by efficient methods for genetic manipulation of wild isolates, which are non-axenic.

Axenic cells are typically transfected by selection for drug resistance in liquid medium. Because the generation time is at best 8–9 hours these selections typically take 1–2 weeks depending on whether an extrachromosomal or integrating plasmid is used. Dead bacteria have been added to the medium enhance viability and allow slow growing mutants to be isolated, and selection systems based on growth on bacteria have been reported [26, 27]. However despite this progress, selection based on growth on bacteria has not been greatly developed, nor widely adopted.

We have previously reported a series of modular vectors for the transfection of *Dictyostelium* (the pDM series) allowing selectable markers, genes to be expressed and fluorescent reporters readily exchanged and having the ability to express two fluorescent reporters from the same plasmid [28, 29]. We have devised a set of protocols for the efficient transfection of *Dictyostelium* cells based on growth on bacteria and built a new set of plasmids to facilitate a wide range of experiments. The new vectors work equally well in traditional axenic selections and allow both wild type and axenic strains to be manipulated and, as a consequence of their faster growth on bacteria, much more rapidly than previously. As an example of the utility of this system we have addressed the controversial role of RasS in cell movement and macropinocytosis by making a gene deletion in wild type cells [30, 31].

## Material and methods

### Cell culture conditions and transfection

#### Strains and cell culture conditions

Three main strains of *D*. *discoideum* are used in this work. NC4(S) is a derivative of the original NC4 isolate obtained from Pauline Schaap with a well-documented history and bears a small duplication on chromosome 6 (all direct NC4 strains tested have some sort of duplication) [14]. DdB(Wel) is a derivative of NC4 from the Sussman laboratory obtained from Dennis Welker and is the parent of the standard axenic strains. It lacks any major duplication. NC4(S) and DdB are both non-axenic. AX2(Ka) is the standard axenic strain of this laboratory; a few experiments used AX3(K) as indicated in the text. These strains are referred to as NC4, DdB, AX2 and AX3 from hereon.

Cells were cultivated at 22°C. The food bacteria, ideally *Klebsiella aerogenes* or *pneumoniae*, were grown overnight in LB medium at 37°C then harvested by centrifugation for 20 min 6,000 x g, centrifugally washed once in SorMC Buffer (15 mM KH_2_PO_4_, 2mM Na_2_HPO_4_, 50 μM MgCl_2_, 50 μM CalCl_2_, nominal pH 6.0) to remove residual medium, and resuspended in SorMC to an OD_600_ = 100. Alternatively KK2 buffer (16.6 mM KH_2_PO_4_, 3.8 mM K_2_HPO_4_, made by mixing the salts and giving a nominal pH 6.1) supplemented with 50 μM MgCl_2_, 50 μM CaCl_2_ is equally suitable. Bacterial preparations were stored at 4°C for up to three months. *Dictyostelium* cells were grown in tissue culture-treated dishes under bacterial suspensions diluted to OD_600_ = 2 in SorMC (10 ml per 9 cm plate).

Alternatively, cells were grown on *Klebsiella aerogenes* lawns on SM agar plates (peptone 10g/L, yeast extract 1g/L, glucose 10g/L, KH_2_PO_4_ 1.9g/L, K_2_HPO_4_ x 3 H_2_O, 1.3g/L, MgO_4_ anhydrous 0.49 g/L, 1.7% agar) made by Formedium either mixed in with the bacteria to give clearing plates 2 days later, or as growth zones.

Axenic cells were cultivated in HL5 medium including glucose supplemented with vitamins and microelements (peptone 14 g/L, yeast extract 7 g/L, glucose 13.5 g/L, KH_2_PO_4_ 0.5 g/L, Na_2_HPO_4_ 0.5 g/L, FM vitamins and microelements 0.01 g/L from Formedium) either in plates or shaking at 180 rpm in conical flasks.

#### Transfection with selection on bacteria

Cells for transfection were grown on *Klebsiella aerogenes* lawns on SM agar plates, and ideally came from clearing plates prepared 2 days before transfection; alternatively three centimetres of a fresh growth zone (the translucent feeding front of a *Dictyostelium* colony expanding on a bacterial lawn) collected with a disposable 10 μl inoculation loop (2-4x10^6^ cells) was used.

Cells were harvested, washed once in ice-cold H40 buffer (40 mM HEPES, 1 mM MgCl_2_, pH 7.0) at 3 minutes 300 xg or 'flash spun' in a microfuge (the centrifuge is accelerated to 10,000 xg and immediately switched off), then resuspended at 2–4 x 10^7^ per ml in H40 (optimal cell density under all tested conditions) and 100 μl transferred to a pre-chilled 2 mm gap electroporation cuvette (Geneflow), with 1–2 μg DNA added and mixed by careful pipetting up and down. The DNA was dissolved in H_2_O or TE buffer and added in a volume not exceeding 5 μl. The chilled cells were electroporated using a square wave protocol of two pulses of 350 V and 8 milliseconds duration separated by 1 second (GenePulser Xcell, Biorad).

All results reported here were obtained using the square wave protocol. The following exponential decay protocol was used successfully too: *Dictyostelium* cells grown in Petri dishes on bacterial suspensions in SorMC (5 x 10^6^–1 x 10^7^ cells per transfection) were spun down, washed once in SorMC as above and resuspended in 400 μl SorMC. DNA was added (1 μg for an extrachromosomal plasmid or 10 μg for knock-outs or knock-ins), the cells chilled on ice in a 2mm gap cuvette and electroporated twice at 500V and 25 μF with a 2 second gap. The capacitance required depends on the internal resistance of the machine (usually about 50 Ω) and should give a time constant of 0.7–0.8 seconds. Cells were immediately diluted into a bacterial suspension of OD_600_ = 2 in SorMC. After 5 hours recovery, selection was added: 10 μg/ml G418 or 100 μg/ml hygromycin depending on the resistance cassette. Colonies appeared 24–48 hours after transfection with extrachromosomal plasmids, and after 4–5 days with integrating vectors.

#### Transfection with selection in HL5 axenic medium

Axenic cells grown in HL5 were spun down and washed twice with ice-cold H40 buffer, resuspended to 5 x 10^7^ cells/ml and 100 μl mixed with 1–2 μg DNA and electroporated as above. The cells were immediately diluted into HL5 and allowed to recover over-night, due to their slower growth, before starting selection. Selection used 10 μg/ml G418, 10 μg/ml blasticidin or 50 μg/ml hygromycin. Colonies appeared after 3–4 days for extrachromosomal and 7–10 days for integrating plasmids.

### DNA cloning—Gene knockouts and plasmids

The extrachromosomal and integrating plasmids were based on the new vector sets described in this paper. A complete list of all oligonucleotides (oDM and oPI) used is given in S3 Table and cloning plasmid sequences in S1 File. H2B was amplified with oPI229/230 using genomic DNA as template. The PCR product was cloned into pJet1.2 (Fisher Scientific). H2B was cloned into pDM1207, pDM1208 and pDM1516 via *Bam*HI/*Spe*I sites. LifeAct-GFP (oPI118/119) and LifeAct-mCherry (oPI118/120) amplification was performed using pDM625 or pDM1469 as template. After cloning into pJet1.2 (Fisher Scientific), the GFP version was cut with *Bam*HI/*Spe*I and the mCherry variant with *Bam*HI/*Xba*I and cloned into pDM1203 and pDM1501. pPI304 the LifeAct-mCherry/PH-pkgE-GFP reporter was constructed from pDM1209 and a shuttle vector for the red reporter based on pDM1020. The plasmids were united using the *Ngo*MIV site present in both.

The *rasS* KO construct was created by joined PCR (see S1 Protocol for a detailed description). The 5’ arm was amplified using oPI148/146 and the 3’ arm oPI149/147. The resistance cassette of pDM1081 was amplified with oDM1015/1016 and mixed for the final PCR step with both recombination arms. The joined PCR was performed using oPI150/151 creating a *Pvu*II site at both ends of the construct and the product ligated into pJet1.2. Before transfection the plasmid was linearized with *Pvu*II.

Sanger sequencing was performed to confirm all plasmids had the desired sequences.

### Southern blotting

About 5–10 μg of high molecular genomic DNA was digested over night and run on a gel the next day to separate the fragments. The gel was soaked for 10 minutes in 0.125 M HCl, rinsed in ddH_2_O and washed in 0.5M NaOH/1.5M NaCl for 30 minutes. The gel was rinsed again in ddH_2_O, followed by a final washing step in 0.5 M Tris pH7.5/1.5 M NaCl for 30 minutes was carried out.

The transfer was done overnight using 5–10 x SSC. Subsequently the wells were marked, followed by UV crosslinking. The blot was pre-hybridised in 30 ml pre-warmed Church buffer (0.5 M NaPO4, pH 7.2, 7% SDS, 1 mM EDTA) at 65°C for 30–60 minutes. The denatured (5 minutes at 95°C) probe was added to the pre-hybridisation buffer, mixed gently but well and then added to the blot. Hybridisation was done for 3–4 hours at 65°C. Thereafter two washes of 15 minutes each were carried out first with 1 x SSC/0.1% SDS followed by 0.1 x SSC/0.1% SDS, both at 65°C. The blot was covered with saran wrap and exposed until clear bands appeared.

### Imaging

Live cell imaging was performed on a Zeiss 710 confocal microscope using the 20x air, or 40x and 63x oil immersion objectives. Images were recorded using Zen2010 software and processed with Fiji, mainly using the median filter to despeckle the acquired images.

#### Motility assay and data analysis

Random motility was analysed with vegetative amoeba cultivated in Petri dishes on bacterial suspensions in SorMC. In some cases they were transfected with an extrachromosomal vector expressing a nuclear marker—H2B-GFP or H2B-mCherry—to simplify tracking. Drug selection was removed 24 h before the assay. For the motility assay cells were washed 5 times in KK2 and resuspended to a final cell density of 2.5 x 10^5^/ml cells in KK2 +100 μM CaCl_2_ and 2 mM MgSO_4_. Cells were plated in 4-well LabTek cover glass chambers and after 30 minutes, time-lapse movies taken for 30 minutes at 2 frames per minute. Movies were analysed with Fiji using the MTrackJ plugin.

#### Cell-Surface adhesion assay

*Dictyostelium* cells adhering to 9 cm plastic Petri dishes were counted before and after shaking at 30 rpm– 90 rpm for 30 minutes on a rotary shaker.

#### Flow cytometry

Flow cytometry was performed using a BD^™^ LSR II flow cytometer (BD Bioscience). Cells were washed 5 times with KK2 buffer to remove bacteria or culture medium and resuspended to 5 x 10^6^ cells/ml, with 50,000 cells analysed using the appropriate filters (B-525 for GFP and YG-610 for mCherry). Macropinocytosis assays were performed in 96-well flat bottom plates as described [21, 32] using HL5 medium plus 10% of FBS to increase fluid uptake by non-axenic DdB cells. TRITC-dextran (1 mg·ml^-1^ final concentration) uptake was measured after 60 minutes.

## Results

### Cultivation of *Dictyostelium* cells on suspensions of live bacteria

Three main strains were used in this work: NC4, the original wild-isolate, and its derivative DdB, both of which have an intact NF1 gene and are non-axenic. AX2 and AX3 are axenic derivatives of DdB, in which the NF1 gene is largely deleted.

We first explored ways of performing antibiotic selections on *Dictyostelium* cells cultivated on bacteria. Co-culture with bacteria on agar was rejected because of potential effects of the antibiotic on the bacteria and metabolism of the antibiotic by the bacteria. We therefore used pre-grown bacteria, washed free of their culture medium, which can be deleterious to *Dictyostelium* growth. Heat-killed bacteria can be used but are prone to clumping; we therefore preferred to use live, washed bacteria [33].

Wild type amoebae in phosphate buffer [34] did not adhere well to tissue-culture plastic in the presence of bacteria and did not recover well after electroporation. Either Mg^2+^ or Ca^2+^ alleviated these defects but millimolar concentrations reduced the effectiveness of selection agents; however, 50 μM of both cations allowed cells to adhere while maintaining strict sensitivity to G418 and hygromycin. The optimised buffer was named SorMC (Sorensen + Magnesium + Calcium).

**Fig 1:**
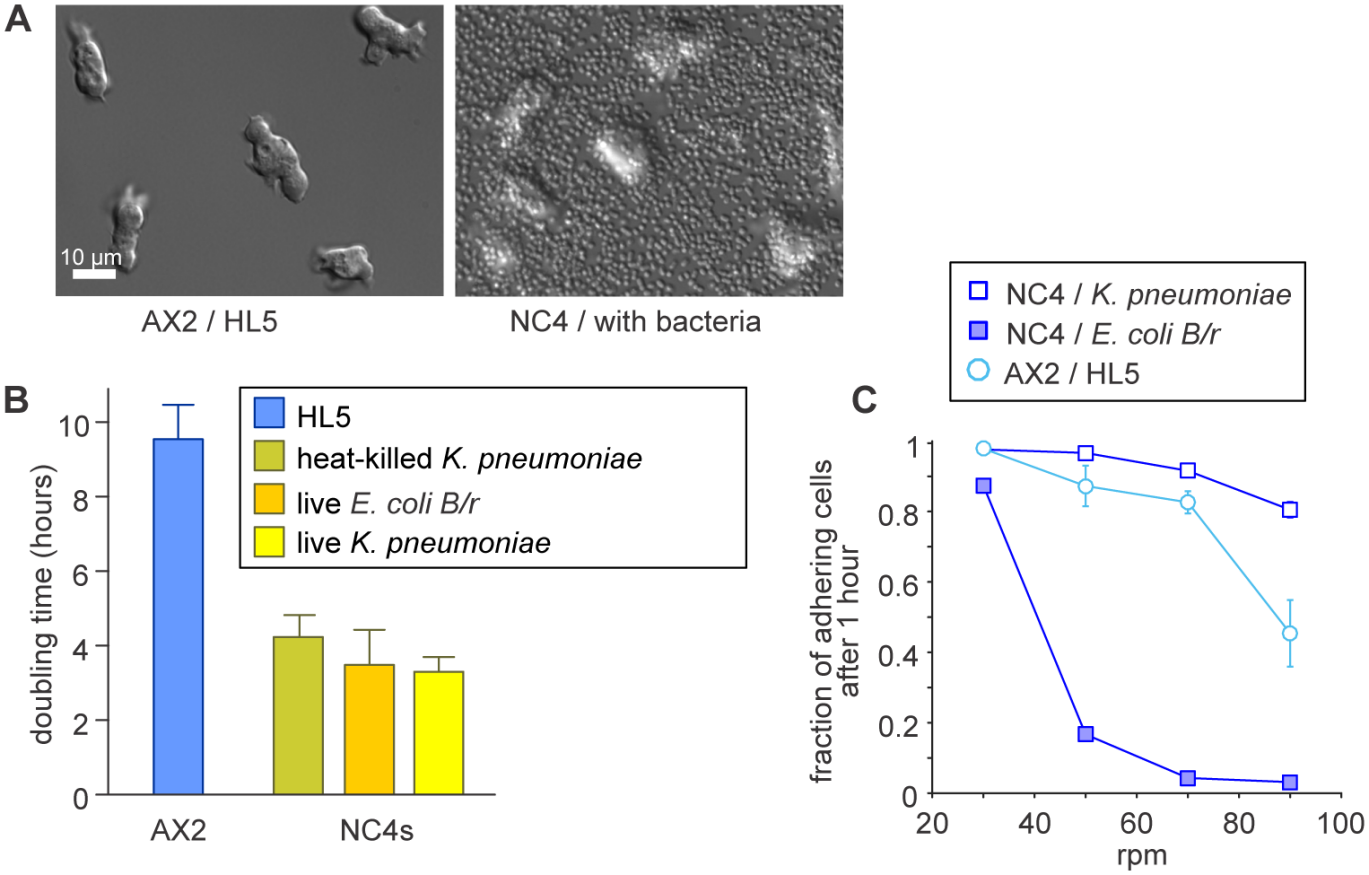
Culturing of cells on bacteria. (A) DIC images of AX2 cells cultured axenically in HL5 medium and of NC4 cells cultured in a suspension of *K*. *aerogenes* bacteria. A disadvantage of growth with bacteria is that bacteria obscure the *Dictyostelium* cells. (B) *Dictyostelium* cells grow much faster on bacteria than in HL5 medium. Cells were grown in tissue-culture dishes with the food source indicated. (C) The adhesion of *Dictyostelium* cells is impaired by co-culturing with *E*. *coli*. Cells in 9 cm Petri dishes were shaken in 10 ml of SorMC buffer supplemented with bacteria to an OD_600_ of 2 on a rotary shaker at the speeds indicated for 30 minutes. The medium was then collected, the cells spun down, resuspended and counted. The cells remaining on the plate were rinsed off with SorMC, spun down, resuspended and counted. Error bars show mean and SD of at least 3 experiments.

The non-virulent sub-strains of *Klebsiella pneumonia* (identical to *Klebsiella aerogenes*), which has been used in *Dictyostelium* laboratories for more than half a century, is the most convenient food source. These bacteria do not form biofilms, remain mono-disperse under all conditions and reproducibly yield dense lawns on SM agar plates. Bacteria of the *E*. *coli* B strain (such as *E*. *coli* BL21 and *E*. *coli* B/r) are a possible alternative if restricted to a safety level 1 environment. However, a disadvantage of using *E*. *coli* is that *Dictyostelium* cells adhere poorly to the Petri dish in their presence (Fig 1C). Neither stringent washing of the bacteria to remove potential toxins nor heat-killing the bacteria overcame the adhesion defect, whose origin remains unknown.

We used a suspension of bacteria at a density of OD_600_ = 2 in SorMC to consistently support growth to confluency of *Dictyostelium* cells, before complete depletion of their food source. Addition of more bacteria does not speed up growth, but does impair visibility of the *Dictyostelium* cells under the microscope (Fig 1A). Wild type NC4 cells double in these conditions in just under 4 hours, which is twice as fast as AX2 cells in axenic medium (Fig 1B). Growth rates are similar with either *K*. *pneumoniae* or *E*. *coli* as food source, but when bacteria are heat-killed the growth rate is somewhat reduced. Axenic strains grow at similar rates on bacteria as wild type NC4 cells (S1 and S2 Movies).

### Selection

**Fig 2:**
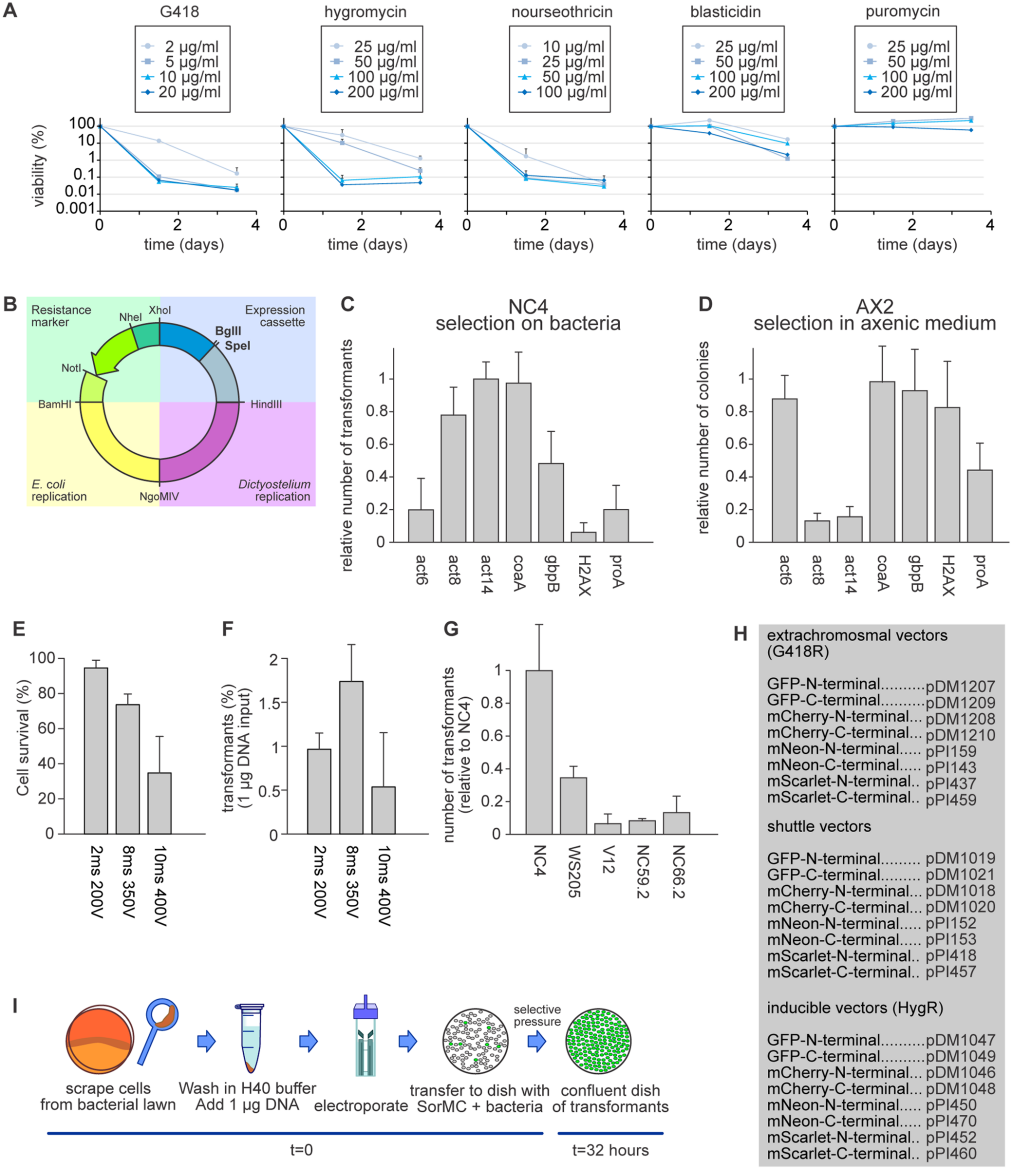
Selection and transfection conditions for bacterially-grown cells. (A) Antibiotic sensitivity of bacterially-grown cells. Cells growing in petri dishes with *K*. *pneumoniae* bacteria in SorMC buffer were treated with the indicated antibiotics. Individual wells were harvested after 36 and 84 hours and the cells plated clonally on SM agar plates to determine viability (error bars are SD). (B) Schematic overview of the extrachromosomal expression vector, with the four modules shown in different colouring: green = resistance marker; blue = expression cassette; purple = *Dictyostelium* replication module; yellow = *E*. *coli* replication module. (C-D) The efficiencies of different promoters at driving the selectable marker in bacterially-cultured cells. Cells were transfected with 1 μg of respective plasmid and the number of colonies counted after 2 days (n = 3; error bars are SD). (E-F) Optimization of electroporation conditions. The survival of cells and number of transfectants obtained after electroporation with 2 square waves, 1 second apart, at the indicated voltages and pulse lengths. Survival was determined by clonal plating on SM agar. (H) Table of extrachromosmal plasmids in the new pDM/pPI system, including inducible and shuttle vectors. (I) Workflow of a non-axenic transfection using an extrachromosomal expression plasmid. If not indicated otherwise, NC4 cells were used in all experiments.

Transfection and selection of cells fed on bacteria were optimised in three steps. First, the antibiotic concentration was optimised, then the plasmid design and finally the electroporation protocol. Kill curves for five different antibiotics (Fig 2A) show that G418 killed cells efficiently at the standard concentration of 10 μg/ml used in axenic selections, while the concentration of hygromycin required increasing from 50 μg/ml to 100 μg/ml. Remarkably, blasticidin, the drug of choice for gene targeting in axenic cells, was ineffective with cells cultivated on live bacteria up to 200 μg/ml. This was true for both *K*. *pneumoniae* and *E*. *coli* as well as also for other *Dictyostelium* wild type strains, such as WS205 and V12M2. When Blasticidin was exposed to bacteria for 4 days it still killed cells growing in HL5 medium, suggesting that the bacteria did not inactivate it (not shown). Puromycin was also ineffective at up to 200 μg/ml. However, nourseothricin was very potent against both bacterially and axenically cultured cells and so could also be used as a selective agent.

### Vector design

Bacterially-cultured cells could be transfected with the standard extrachromosomal vector pDM304 [28], however the number of transfectants was almost 100-fold lower than with cells grown axenically, and the transfected cells grew slowly, with many rounding up and floating. We suspected that the drug-resistance gene was not adequately expressed, possibly because the *act6* promoter fragment that drives expression is insufficiently active in cells grown on bacteria [35]. We therefore tested promoters from 6 highly expressed genes [36] by inserting a fragment of about 350 base pairs from their proximal promoter into the modular expression vector, modified by adding restriction sites before and after the selectable marker to allow convenient swapping of its promoter (Fig 2B). The *act14* or *coaA* (Coactosin) promoters gave 5-fold more colonies than the *act*6 promoter (Fig 2C). The colonies were compact and the transfected cells grew at comparable rates to untransfected cells. As plasmids using the *coaA* promoter to drive the selectable marker also performed well in axenic selections (Fig 2D), it was adopted for the new generation of plasmids (Fig 2H).

### Electroporation

A variety of electroporation protocols were tested using NC4 cells harvested from the feeding front of colonies formed on bacterial lawns [5, 37–41] and a plasmid expressing a GFP marker. The electroporation parameters were iteratively explored to optimise the number of resulting colonies with protocols adapted from Pang et al. [41] giving the best results, but also high levels of cell death, slow cell recovery and variable efficiency between experiments. We therefore further optimized this protocol, starting with the composition of the electroporation buffer.

To avoid the effect of altered conductivity on the time constant when adapting buffer components, we switched to a square wave electroporation, as this keeps the applied voltage and pulse length the same, regardless of the ionic strength of the buffer. The essential components of the buffer were identified by removing them one by one. Surprisingly, a completely stripped buffer containing only HEPES was as efficient as the complex H50 buffer of the standard axenic protocol. Omission of the HEPES (thus water alone) dramatically reduced the electroporation efficiency, as did switching to phosphate or Tris buffer. MOPS and MES worked reasonably well, but not as well as HEPES. Increasing the HEPES concentration to 40 mM increased the number of transfectants, but higher concentrations were detrimental. The transfection frequency varies less than 3-fold at HEPES concentrations between 20 mM and 60 mM and pH between 6.5 and 7.5, meaning that the buffer is robust against small variations. Individual components were then added back, including K^+^, Na^+^, Mg^2+^, Ca^2+^, ATP, NaHCO_3_, sucrose and DMSO. Of these components, only Mg^2+^ improved the transfection frequency, by improving cell survival, allowing higher voltages to be applied, with concomitant increases in transfection efficiency. Including 1 mM MgCl_2_, in 40 mM HEPES pH 7.0 gave the final buffer H40 buffer.

To optimise the electroporation, the pulse length and applied voltage were increased step-wise until cell survival started to drop (Fig 2E). Two pulses of 8 ms and 350 V, 1 seconds apart were optimal (Fig 2F). A similar optimisation using only 1 pulse or using more than 2 pulses resulted in a lower maximum number of transfectants. Extremely high transfection efficiencies of up to 2% were routinely achieved using 5x10^6^ cells and only 1 μg of vector. Since cells double every 4 hours, this means that a confluent Petri dish of transfected cells was obtained in less than 2 days for extrachromosomal plasmids, a notable improvement over axenic cells where this takes one week. Larger vectors transfected at a lower frequency, and the number of transfectants did not scale linearly with the amount of DNA, with more than 5 μg of DNA frequently causing excessive cell death for little useful gain.

These optimized buffer and electroporation settings were tightly coupled to the conditions used to obtain them. High transfection efficiencies were only obtained with input cells taken from bacterial lawns on SM agar. Cells harvested from Petri dishes with SorMC and bacteria did not survive the optimised protocol. The protocol was most efficient when using NC4 cells or the related DdB or AX2 cells as input and was much less efficient with other *Dictyostelium* strains, such as WS205 or V12M2 (Fig 2G). A schematic of the optimised protocol and the new standard expression vector pDM1203 (derived from pDM304) are shown in Fig 2B. If no square wave electroporator is available, an exponential decay protocol can also be used.

### Extrachromosomal expression

#### Constitutive expression

With this efficient transfection system, we next examined protein expression using an extrachromosomal plasmid expressing cytoplasmic mCherry. DdB cells were transfected and maintained under selection for 72 hours. Their fluorescence was quantified afterwards by flow cytometry. This showed that 85–98% of cells expressed mCherry at measurable intensities with slight increases at higher G418 concentrations (S1A Fig).

Based on this, we created a complete set of expression plasmids to produce N-terminal and C-terminal fusion proteins for a variety of different fluorescent proteins (Fig 2H). These include the recently engineered mNeon [42] and mScarlet [43], which offer a considerable increase in brightness compared to GFP and mCherry (S1B Fig). All fluorescent proteins used in the plasmids are *D*. *discoideum* codon optimized.

A valuable feature of the extrachromosomal plasmids, in addition to their modularity, is that they allow two different proteins to be expressed from the same plasmid, and thus the creation of dual reporters having similar expression in single cells. This is a major improvement over single reporter plasmids, which make it difficult to get strong expression of two reporters in individual cells, due to high levels of spontaneous expression heterogeneity. We tested whether this improved co-expression potential applies under non-axenic conditions by creating a plasmid expressing both the F-actin marker LifeAct-mCherry and the PIP3 marker PH-*pkg*E-GFP. After transfection of this plasmid into AX2 cells, fluorescence microscopy showed that cells expressed both reporters with a very strong correlation between their expression levels, similar to axenic cells (S1B Fig). Similar results were obtained with other reporter pairs [21]. After this confirmation we expanded the repertoire of shuttle vectors to include additional tagging options. Overall, the new extrachromosomal plasmids behave in non-axenic conditions like the earlier pDM plasmids under axenic conditions, with the advantage of allowing much faster selection of transfectants [28].

#### Inducible expression

For extrachromosomal inducible expression, we adapted the doxycycline (dox) dependent inducible expression system [11] for cells grown on bacteria. The new vectors (Fig 2H) all use hygromycin resistance as the selectable marker and have a dox-on trans-activator.

Standard dox concentrations were effective under non-axenic conditions when using NC4 cells transfected for cytoplasmic GFP or mCherry expression. The cells were transfected and selected for 72 hours before doxycycline was added to induce expression. Fluorescence was measured 16 hours after the start of induction. Half-maximal expression was obtained at 1 μg/ml doxycycline, which is somewhat higher than with cells grown in HL5 where half-maximal expression was reached at 0.1 μg/ml. Maximal expression is obtained at 10 μg/ml. Importantly, expression levels were very low in the un-induced state and could be induced over 1,000-fold. The fraction of positive expressing cells was 60–70%, increasing slightly at higher doxycycline concentrations (S2A and S2B Fig). Overall, the inducible expression system works well under non-axenic conditions. Similar results could be obtained for axenic cells when grown in HL5.

### Isolation of stably transfected clones

#### Random integration—REMI

**Fig 3:**
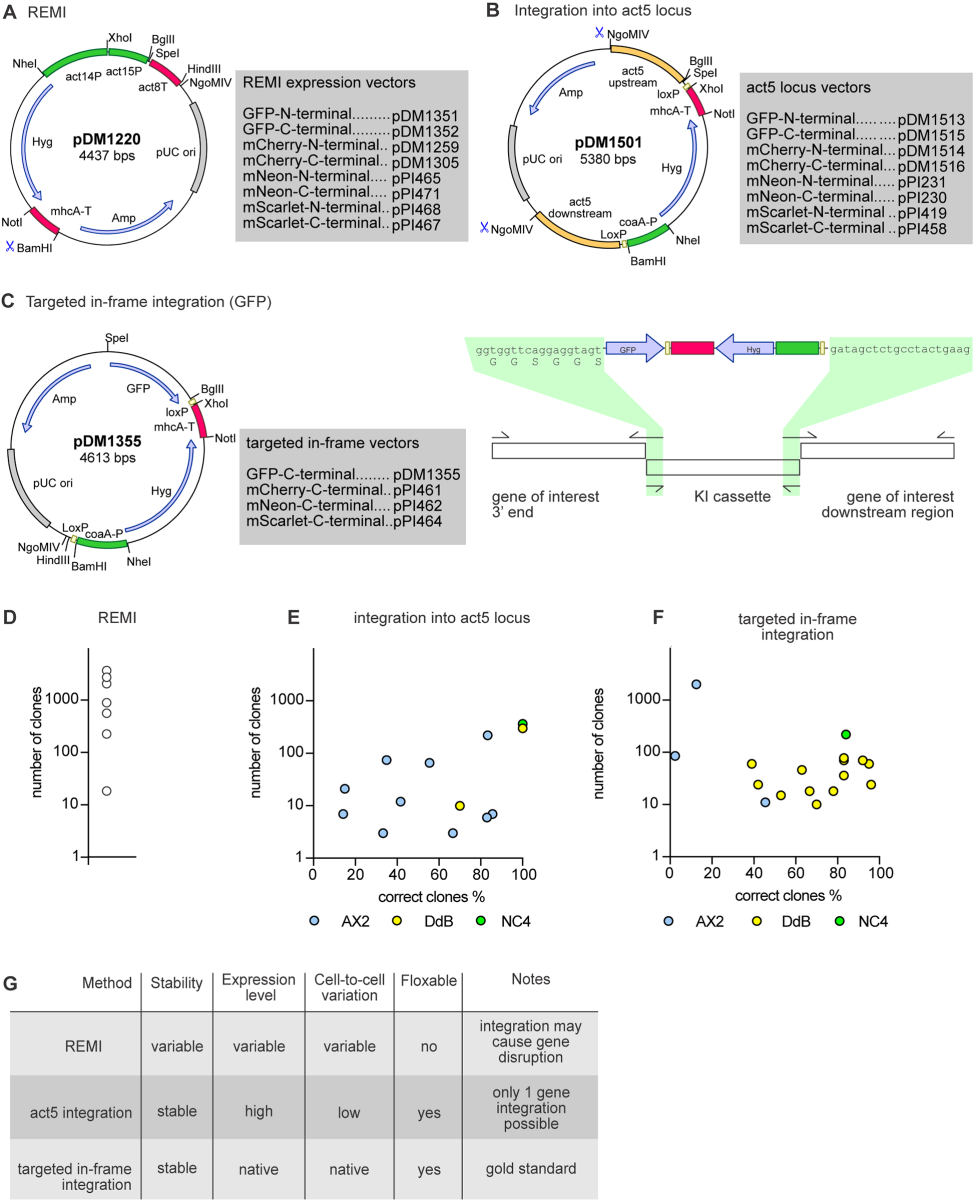
Stable expression using integrating plasmids. (A) Schematic map of the REMI expression vector and table of versions for N-terminal and C-terminal tagging. In all plasmid maps the promoters are highlighted in green, terminators in pink, open reading frames in blue and origins of replication in grey. (B) Map of the *act5* integration plasmid including a table of all different versions available for the creation of fluorescent fusion proteins. The recombination arms used for the integration at the *act5* locus are highlighted in orange. (C) Map and schematic overview of the plasmid for targeted in-frame integration to produce C-terminal knock-ins. The crossover sequences used for the knock-in construct assembly are highlighted in green. (D-F) Efficiency of transfection using the three different strategies for making stable cell lines. Cells were transfected with 1–2 μg DNA using the optimised method outlined in Fig 2. For the *act5* and targeted in-frame integrations the percentage of positive clones is given on the x-axis. The different strains used for the transfection are separately shown indicated by different colouring (AX2 in blue, DdB in yellow and NC4 in green). See Tables 1 and 2 for details of the genes. White circles shown for the REMI efficiency are for NC4. Best results came with 2 μg of *Bam*HI-digested DNA, 5 units of *Dpn*II and a vector with the *coa*A promoter replaced by the *act14* promoter. (G) Table summing up the advantages and disadvantages of the three different integration methods.

Extrachromosomal vectors are useful for rapid gene expression, but for some purposes genomic integration is desirable. Earlier generations of transfection vectors randomly integrated into the genome, but this proved unexpectedly difficult with the new vectors. Integrating expression vectors were constructed by removing the region responsible for extrachromosomal maintenance. Numerous transfection attempts yielded only marginal numbers of colonies, most of which did not express the gene of interest. Several iterations of the vector, different electroporation protocols and selection conditions gave no improvement. Only the REMI protocol (Restriction Enzyme Mediated Integration [17]) reproducibly gave substantial numbers of colonies (Fig 3A and 3D). While clones could be selected with suitable expression and low cell-to-cell variation, many had little or no expression and the highly expressing clones were usually unstable, losing expression of the gene of interest within a week, despite maintaining selective pressure. The nature of these unstable transfectants is unknown. Different variations of the protocol did not alleviate this problem. In conclusion, it is possible to generate stable clones via random insertion into the genome of wild type cells but, as with axenic cells, screening for suitable clones can be tedious and the copy number and genomic location of the plasmid is not defined.

#### Safe locus integration

The variation in expression between random integrants is probably due to variation in their local genomic environments and position effect variegation effects typical of heterochromatic insertion sites. One way to remove this variability is to use specific ‘safe haven sites’, such as the *Rosa26* locus in mouse genetics [44], where integration of foreign DNA results in the stable expression of the integrated gene but has minimal phenotypic effect.

In *Dictyostelium*, the *rps30* locus has been previously used as a ‘safe haven’ [9, 45, 46]. As a gene encoding a ribosomal protein, it provides strong and relatively uniform expression. However, *rps30* is an essential gene, so its use as a ‘safe haven’ is limited to strains with a duplication of the chromosomal region containing *rps30*, such as AX3 and AX4. The *rps30* gene would not be suitable for wild type cells. The *act5* locus has strong potential as a safe landing site [47, 48]. The *act5* gene is a highly expressed member of a family of 17 genes encoding identical actin proteins [49], minimising potential phenotypic effects resulting from its loss.

On this basis, we constructed a vector to replace the *act5* gene with a gene-of-interest. A fragment containing the standard *Bgl*II/*Spe*I cloning site, followed by a polyadenylation signal and hygromycin resistance cassette, was flanked by two recombination arms consisting of ~750 base pairs immediately upstream of the *act5* start codon and downstream of the *act5* gene respectively. LoxP sites were added either side of the resistance marker cassette, allowing it to be recycled (Fig 3B).

We integrated mScarlet into the *act*5 locus of AX3, thus substituting the fluorescent protein and resistance cassette for the *ac*t5 coding sequence (S3A Fig). PCR showed that the *act*5 gene was replaced by the knock-in construct (S3C Fig for two independent clones) and this was confirmed by Southern blots (S3B and S3D Fig). These showed a single band of the expected size, consistent with a single integration at the *act5* locus. Flow cytometry showed highly homogenous expression of the fluorescent protein, indicating limited cell-to-cell variation in expression from the locus (S3E and S3F Fig). The lines were stable in the absence of selection. The generality of the ‘safe haven’ approach was tested with different cell lines and plasmids. NC4 cells were transfected with a derivative plasmid to generate mCherry expressing cell lines. Of the 320 resistant clones obtained, twelve randomly chosen ones all showed correct integration at the *act5* locus (Table 1 and Fig 3E) and four of these were investigated further. Fluorescence microscopy showed minimal variation of cellular fluorescence both within and between clones (S3A and S3B Fig). Similar results were obtained with DdB (240 clones; 11/12 correct integrants) and AX2 (221 clones; 10/12 correct). Flow cytometry confirmed the minimal variation between cells and clones and showed that expression levels are remarkably similar between strains (S4C and S4D Fig). We obtained similar results with a plasmid substituting GFP for the *act*5 coding region in AX2 cells (S5A–S5C Fig).

**Table 1:** Knock-ins to the *act5* locus generated with cells grown in bacterial suspension.

Construct targeted to *act*5 locus	Number of transfectants	Clones checked	Correct clones	Correct clones (%)	*Dictyostelium* strain used
LifeAct-mCherry	7	7	1	14.2	AX2
LifeAct-GFP	12	12	5	41.6	AX2
GFP	3	3	2	66.6	AX2
mCherry	3	3	1	33.3	AX2
GFP	66	9	5	55.5	AX2
mCherry	221	12	10	83.3	AX2
H2B-mCherry	3	3	1	33.3	AX2
H2B-mCherry	7	7	6	85.7	AX2
mCherry	10	10	7	70	DdB
mCherry	240	12	11	91.6	DdB
mCherry	320	12	12	100	NC4

When the resistance cassette was removed by transfecting cells with a Cre expression plasmid, the high proportion of cells expressing GFP was maintained but the individual cell fluorescence fell by about 50% (S5D and S5E Fig). A similar loss was obtained with *act5*::mCherry knock-ins, but not with a histone H2B knock-in (an *act5*::H2Bm-Cherry KI; S6A–S6C Fig). H2B knock-ins are a useful marker of the nucleus and can be used for long-term tracking of cells, for instance during chemotaxis (S3 Movie) and for segmenting nuclear contents and dynamics during image processing [45, 50, 51]. Thus removing the selectable marker may affect expression levels, but the stability and uniform expression of the knock-in are unaffected.

We compared expression levels of GFP at the safe-landing site with expression from an extrachromosomal plasmid for cytoplasmic GFP driven by the *act*15 promoter, using AX2 cells cultured on bacteria for 3 days with 10 μg/ml G418. The extrachromosomal plasmid, which is present in multiple copies per cell, gave about four-fold higher fluorescence than the *act5*::GFP knock-in clone (S5H and S5J Fig), but expression was much more variable, with around 10% of the cells lacking detectable fluorescence (S5G and S5I Fig). In conclusion, integration into the *act5* locus offers a reliable method to generate clones with relatively uniform, stable, and fairly strong expression, which are very suitable for microscopy and other purposes, such as genetic screens for defective gene expression regulation (Fig 3E).

#### Genomic knock-ins

A more sophisticated genetic manipulation is an in-frame targeted integration (also called a knock-in), where a gene of interest in the genome is tagged or mutated with minimal disruption to its genomic environment, thus potentially maintaining native gene expression [52]. We used two different routes for building C-terminal knock-in vectors. In the first, a cassette was created for cloning a GFP knock-in construct (Fig 3C) in two PCR rounds. The 5' and 3' recombination arms and the GFP knock-in cassette were first amplified, then the PCR fragments mixed and stitched together using the region indicated in Fig 3C as overlap between the fragments. This knock-in system is also available for C-terminal fusions with mNeon, mCherry and mScarlet. The second route used stepwise assembly, with the 5' recombination arm inserted using the unique *Bgl*II/*Spe*I sites, and the 3' recombination arm using the *Sal*I/*Sac*I sites (S7 Fig).

We tested the efficiency of knock-ins under non-axenic conditions using a construct targeting the HSPC300 component of the SCAR/WAVE complex. Transfection yielded 220 clones with 10 out of 12 testing correct. The clones yielded essentially identical expression levels with very low cell-to-cell variation (S8 Fig). Using AX2 cells, more than 2,000 colonies were obtained with 2 correct clones out of 16 screened. To test the generality of this approach we attempted knock-ins of 16 further constructs in DdB and AX2, with success in all cases, obtaining between 10 and 2,000 clones with 2% to 95% positive, depending on the gene (Fig 3F and Table 2). In conclusion, knock-ins can be produced with an efficiency realistic for routine derivation.

**Table 2:** Knock-ins to the endogenous locus generated with cells grown in bacterial suspension.

Targeted gene & tag	Number of transfectants	Clones checked	Correct clones	Correct clones (%)	*Dictyostelium* strain used
HSPC300-GFP	2000	16	2	12.5	AX2
HSPC300-GFP	220	12	10	83.3	NC4
FLAG-NF1	36	18	15	83.3	DdB
GFP-NF1	36	12	10	83.3	DdB
NF1-BirA	10	10	7	70	DdB
NF1-BirA	18	18	14	77.7	DdB
NF1-FLAG	15	15	8	53.3	DdB
NF1-mNeon	60	23	9	39.1	DdB
NF1-GFP	70	12	11	91.1	DdB
NF1-GFP PM	78	12	10	83.3	DdB
NF1-GFP IN	70	12	10	83.	DdB
NF1-BirA	46	19	12	63.1	DdB
NF1-BirA PM	60	19	18	94.7	DdB
NF1-BirA IN	15	15	8	53.3	DdB
NF1-mNeon PM	24	24	10	41.6	DdB
NF1-mNeon IN	18	18	12	66.6	DdB
GefB-GFP	11	11	5	45.4	AX2
GefF-GFP	55	55	2	3.6	AX2

#### Gene knock-outs

**Fig 4:**
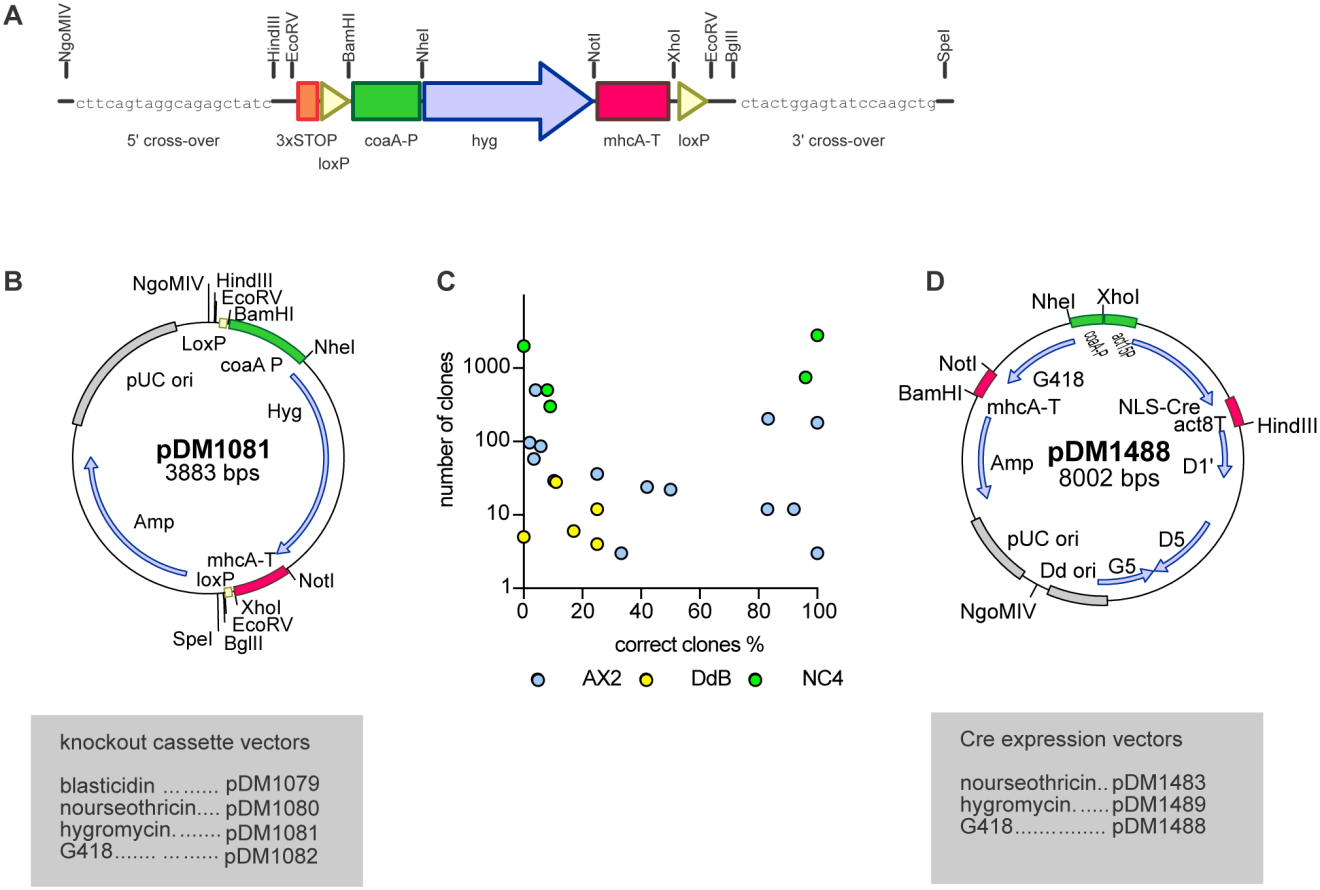
Generation of gene knock-outs. (A) Scheme of the knock out resistance cassette. The resistance marker is flanked by loxP sites (yellow triangles). A 20 bp custom primer site on each end of the cassette allows assembly of the knock-out construct by joined PCR (for a detailed protocol see the S2 File). The resistance marker is driven by the *coaA* promotor (green) and terminated using the *mhcA* terminator (pink). (B) Map of a knock-out plasmid (HygR). Promotors are displayed in green, terminators in pink, open reading frames in blue and origins of replication are highlighted in grey. (C) Knock-out transfection efficiency showing the percentage of correct clones plotted against the number of clones obtained after transfection. The different wild type strains used are indicated through different colouring (AX2 in blue, DdB in yellow and NC4 in green (D) Map of a Cre expression plasmid used to remove the resistance cassette flanked by LoxP sites from mutants. Promoters are displayed in green, terminators in pink, open reading frames in blue and origins of replication are highlighted in grey. D1, D5 and G5 are the minimal set of ORFs necessary to keep the plasmid extrachromosomal.

A knockout cassette for non-axenic strains was produced with the resistance marker cassette flanked by loxP sites and two unique restriction sites each side (Fig 4). Knockout constructs can be prepared in three different ways using this cassette: (1) the knockout cassette can be excised with *Eco*RV and ligated blunt into a cloned gene; (2) the 5' and 3' recombination arms can be obtained by PCR and cloned into the *Ngo*MIV/HindIII and *Bgl*II/*Spe*I sites respectively; (3) PCR fragments of the 5' arm, the knockout cassette and the 3' arm can be stitched together by a second PCR using the indicated sequences as overlap between the fragments. We created 17 different knockout constructs and transfected them into AX2, DdB or NC4 cells under non-axenic condition, with knockout clones being obtained in all but two transfections at efficiencies varying between 2% and 100%, as summarized in Fig 4C and Table 3. In all knockouts tested the resistance cassette could be removed using an extrachromosomal vector expressing the Cre enzyme. Selective pressure was applied for 2 days after the electroporation, after which cells were clonally plated on SM agar. Clones that have lost the selectable markers used for knockout generation and Cre expression could be routinely obtained, typically at a frequency of 60%–90% (Table 4).

**Table 3:** Gene knock-outs generated with cells grown in bacterial suspension.

Disrupted gene	Number of transfectants	Clones checked	Correct clones	Correct clones (%)	*Dictyostelium* strain used
rac1B	750	24	23	95.8	NC4
*rac1*C	2800	24	24	100	NC4
*rac1*A	500	24	2	8.3	NC4
*axe*B (NF1)	2000	15	0	0	NC4
*scr*A (SCAR)	300	11	1	9.1	AX2
*scr*A (SCAR)	500	24	1	4.1	AX2
*ras*S	22	22	11	50	AX2
*ras*S	12	12	3	25	DdB
*cor*A	12	12	11	91.6	AX2
*myo*C	12	12	10	83.3	AX2
*erk*B	28	28	3	10.7	DdB
*erk*B	6	6	1	16.6	DdB
*erk*B	4	4	1	25	DdB
*erk*B	5	5	0	0	DdB
*rac*A	24	24	10	41.6	AX2
*ras*S	24	24	10	41.6	AX2 *ras*G-
*ras*C	96	96	2	2	AX2
*pkb*R1	36	36	9	25	AX2
*pkb*R1	29	29	3	10.3	AX2 *akt*-
*myo*C	203	12	10	83.3	AX2 *myo*B/E-
*myo*C	181	12	12	100	AX2 *myo*E/F-
*for*G	58	58	2	3.4	AX2

**Table 4:** Removal of the selectable marker with cells grown in bacterial suspension.

Gene	Number of transfectants	Clones checked	Correct clones	Correct clones (%)	*Dictyostelium* strain used
*myo*B	198	8	6	75	AX2 *myo*B/E-
*myo*F	197	13	13	100	AX2
*act*5::H2B-mCherry	74	7	7	100	AX2

#### Application to axenic cells

Our transfection procedure is also valuable for cells grown axenically, as the electroporation conditions are milder and less DNA is required than in standard modern procedures. Knock-outs were obtained at similar efficiencies to using bacterial selection and we also readily obtained knock-ins, including those at the *act5* locus. Although the efficiency of promoters can differ depending on the cultivation method, the expression level of axenically-derived *act5* knock-ins is similar to cells grown on bacteria (S9 Fig).

### Different motile behaviour of wild type and axenic cells

**Fig 5:**
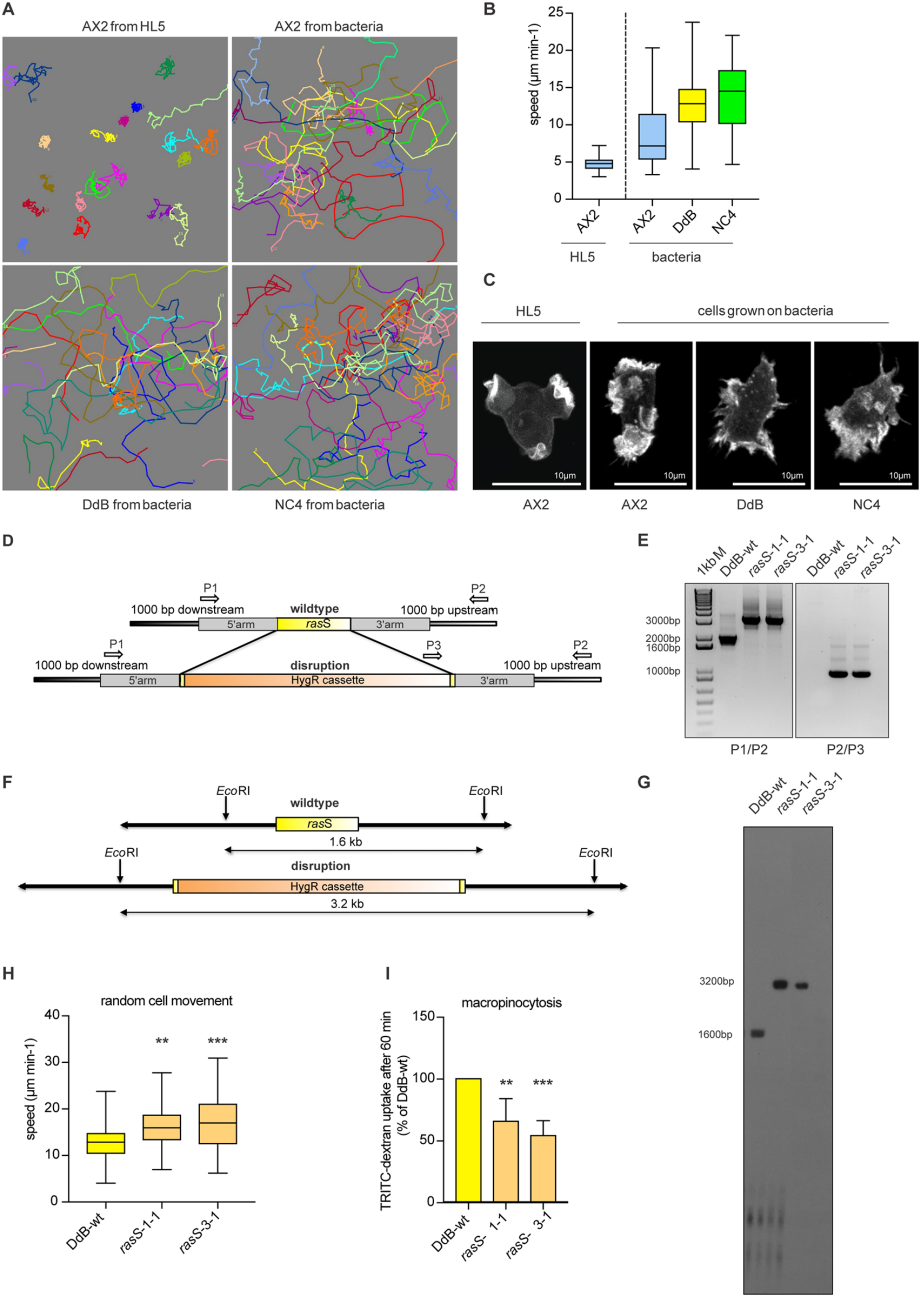
The relationship between axenic growth, cell motility and macropinocytosis and the role of RasS in vegetative cells. (A) Cell tracks of randomly moving vegetative cells. AX2 cells were grown in HL5 or bacterial suspension. DdB and NC4 cells were cultured in bacterial suspension. Pictures show the tracks of 20 individual cells for each strain. The cells were filmed for 30 minutes at 2 frames per minute. (B) The effect of axenic mutations and axenic growth on cell motility. AX2 cells grown in HL5 move more slowly than AX2 grown on bacteria, which in turn are slower than wild type DdB or NC4 cells. Movies were taken at 2 frames/minute, with 3 movies per strain and 20 cells analysed from each movie. Whiskers show maximal and minimal values, with the median shown as a solid black line inside each box, where the top and the bottom represent the first and third quartiles. (C) The actin cytoskeleton of vegetative AX2, DdB and NC4 cells. Macropinosomes are the dominant feature of AX2 cells grown in HL5, whereas pseudopods dominate DdB and NC4 cells grown on bacteria; AX2 cells grown on bacteria are intermediate between these extremes. Cells expressing LifeAct-GFP as a reporter for F-actin were cultured in HL5 or bacterial suspension and examined by confocal microscopy. Scale bar 10 μm. (D) Cloning strategy used for the knock-out of *ras*S. The gene is shown in yellow and the disruption cassette coding for the hygromycin resistance gene in orange. The recombination arms highlighted in grey are 700 bp each and cover just about 30 bp of the coding sequence of *ras*S gene on each side. Primer sites are indicated with arrows. (E) Confirmation by PCR of two independent *rasS-* knock-out mutants (HM1920 *ras*S- 1–1 and HM1921 *ras*S- 3–1) using two different primer combinations. The band shift in first PCR (P1/P2) shows that the gene was successfully disrupted, with no wild type band detectable. The second PCR (P3/P2) confirms the correct insertion of the resistance cassette in the *ras*S gene. DdB wild type DNA was used as control in both reactions. The binding site of Primer 2 is located outside of the 3’ recombination arm and so is locus specific. (F-G) Verification of the disruption of the *ras*S genes by Southern Blotting. Cleanly disrupted recombinants are revealed by the loss of the wild type 1.6 kb band on Southern blots hybridised to a ^32^P labelled *ras*S probe. The appearance of a band of approximately 3.2 kb indicates the genomic *ras*S locus has been successfully disrupted by the hygromycin resistance cassette (The blot has been cropped. The unmodified original is shown in the S2 File). (H) The speed of random movement of vegetative wild type (yellow) and two *rasS-* mutants (orange) was measured as described in Fig 5B. The average of three experiments; maximal and the minimal values are displayed by whiskers and the median by the black line inside each box plot. Statistical analysis used an unpaired two-tailed student-t test (** p <0.005, *** p<0.0005). (G) Fluid uptake by DdB and *rasS-* mutant cells. The cells were incubated over night in HL5 containing 10% foetal calf serum, then the uptake of TRITC-dextran measured after 60 minutes by flow cytometry. Results are normalised to DdB. The error bars indicate the SD, n = 6; statistical analysis by an unpaired two-tailed Student-t test (* p <0.005, ** p<0.0005).

A major motivation for developing methods to transfect non-axenic wild type cells was to escape the confounding effects of the axenic mutations on cell movement and chemotaxis. These are most apparent in axenic cells (such as AX2) grown in HL5 medium, where the cells move very slowly compared to wild type cells (NC4 or DdB) because they preferentially make macropinosomes instead of pseudopods (Fig 5) [22]. The situation is alleviated somewhat by growing AX2 on bacteria, but still their speed is only around half that of wild type cells and their actin cytoskeleton, which is organised into macropinosomes when they are grown axenically, still tends to make un-productive protrusions, compared to the compact pseudopods of wild type cells.

It has been proposed that the competition for cytoskeletal resources between pseudopods and macropinosomes is regulated in part by RasS: vegetative *ras*S- mutants isolated in DH1 (an AX3 derivative) move much faster than their parent but are severely impaired in macropinocytosis [30]. However, subsequent isolation of a *ra*sS- mutant in an AX2 line revealed the opposite phenotype, with the mutant cells moving slower [31]. In an attempt to discover the ancestral role of RasS, in the presence of NF1, we made multiple knock-outs of *ras*S in DdB, the parent of both AX2 and AX3. The disruption construct shown in Fig 5D was used and resistant clones analysed by PCR to confirm the correct insertion into the *ras*S gene (Fig 5E), with confirmation of a single integration in this locus by a Southern blot (Fig 5F and 5G). Analysis of these *ras*S- mutants showed that random cell movement was modestly increased during random cell motility, but less dramatically than in DH1. Macropinocytosis is much lower in DdB cells due to the intact NF1 gene, but it can be stimulated by culturing them in a rich medium supplemented with serum [32]. In these conditions, loss of RasS impairs fluid uptake. In summary, knock-outs of *ras*S in DdB cells resemble those in DH1, and not AX2, although the effects are not so strong. We therefore conclude that RasS is a positive effector of macropinocytosis and a negative effector of cell movement and that these effects are in some way masked in AX2 by additional mutations (Fig 5F and 5G).

## Discussion

Non-axenic, wild type *Dictyostelium* cells have thus far been used only sporadically for molecular research and methods for their genetic engineering are limited [28, 29]. Here we have developed a broad set of tools that can be used to transfect any *D*. *discoideum* strain and by which standard genetic procedures can be performed rapidly and efficiently on cells grown with bacteria. These protocols can also be applied to axenic cells where they are faster and use less DNA. We have developed the *act5* safe locus integration system, which provides stable and uniform expression of the integrated transgene. This will be valuable for reporters, FRET sensors, comparing mutant proteins and genetic screens for regulators of gene expression. To facilitate the use of this bacterial transfection system, detailed protocols and the sequences of the new plasmids are included in the Supplementary Materials.

One small disadvantage of the bacterial transfection system is that blasticidin is no longer effective as a selective agent, as cells growing on live bacteria are highly tolerant to the drug. We do not understand the reason for this resistance, especially as blasticidin selections using heat-killed bacteria have been successful [33]. However cells remain sensitive to G418 and hygromycin and this pair of selectable markers, combined with the Cre/Lox system for recycling the selectable marker [18], allows all standard genetic manipulations, such as making multiple mutants.

How DNA integrates into the genome depends on the balance between homologous and non-homologous events [53]. Random integration of a reporter proved unexpectedly difficult in bacterially-transfected cells, whereas knock-in and knock-out events were generally very efficient, suggesting that homologous recombination is the preferred route of DNA repair in these circumstances. Integration of cut plasmid could be enforced by the simultaneous introduction of a restriction enzyme [17], but our much-preferred route for stable integration is to employ homologous recombination to introduce constructs at the *act*5 “save haven”. Further such sites would also be valuable if they can be identified to allow a range of potential transgene expression levels. We did not further study the REMI process, but optimization for single-site integration and minimal collateral damage to the genome is desirable before it is used for insertional mutagenesis in non-axenic cells.

The major axenic mutation is the loss of the RasGAP NF1 [20] which is expected to regulate Ras and PI3-kinase signalling. It appears that a significant number of the PIP3 and active-Ras patches prevalent in the plasma membrane of axenic cells are macropinosomes caused by the NF1 deletion. These patches have been afforded a central role in schemes for sensing chemoattractant gradients [54] but using the new transfection methods it is now possible to examine the role of PIP3 patches and more general chemotactic mechanisms in wild type cells.

Undesirable strain adaptation is an inherent problem in cell biology. Microarray analysis showed that since the adoption of the AX2 and AX3 strains in 1970 there have been multiple genetic rearrangements [12, 14] and these mutations can lead to synthetic phenotypes. For example, a reported *phg*2 mutant shows decreased adhesion in one laboratory strain background and increased adhesion in another laboratory strain background [55, 56]. It is now possible to resolve such conflicting data by repeating the experiment in the common ancestor strain, in this case DdB, as we have done for RasS. Our results support the original conclusion that RasS stimulates macropinocytosis and impedes cell migration, although the effects are not as strong as those first reported [30, 31, 57]. The modifying mutations affecting the phenotype of *ras*S- mutants in AX2 remain unknown: they could be general to all AX2 strains, or specific to this particular one.

The ability to do genetic experiments in any *D*. *discoideum* strain will facilitate several research lines that depend on the use of multiple wild isolates, notably those of social evolution, kin recognition and the sexual cycle [2–4]. It should also increase the robustness of genetic research, allowing the generation of independent knock-outs in different wild type backgrounds. We conclude that our newly established protocols pave the way to including non-axenic wild type strains in future research as they are now accessible for genetic engineering.

## Supporting information

S1 Fig Expression of fluorescent proteins from an extra-chromosomal vector in non-axenic wild-type cells, including new brighter fluorescent proteins suitable for difficult samples. (A) Quantification of mCherry fluorescence intensity after transfection of the extrachromosomal plasmid pDM1208 in DdB cells. Cells were selected with different concentrations of G418 (10 μg to 30 μg/ml). Bar diagrams show the average of the mean fluorescence intensity measured using flow cytometry. The experiment was repeated three times; error bars indicate the SEM. On the right a typical fluorescence profile is displayed, showing the relative mCherry fluorescence plotted against the cell count. (B) Comparison of whole cell fluorescence intensity of cells expressing mCherry or mScarlet. On the left the relative fluorescence of a clonal cell line expressing *act5*::mCherry or *act5*::mScarlet are shown. The bar diagrams show the fluorescence measured by flow cytometry, using a YG610 filter. The error bars represent the SEM. On the right the fluorescence profile for a representative experiment is shown. Fluorescence intensity is plotted against the cell count. Highlighted in blue is an *act5* driven mCherry and in red an mScarlet clone. (C) Images of AX2 cells expressing a LifeAct-mCherry/PH-pkgE-GFP (pPI304) double reporter for F-actin and PIP3. Cells were grown in bacterial suspension. Scale bars are 10 μm. (D) Correlation plot of mCherry and GFP fluorescence of the cells imaged in (C).
(TIF)

S2 Fig Efficient inducible expression in bacterially cultured cells. Adaptation of the doxycycline inducible expression system to cells grown on bacteria. (A-B) Dose-response curves for GFP (pDM1047) and mCherry (pDM1046) expression induced by doxycycline. NC4 cells were transfected with the respective plasmids and cultured in the absence of doxycycline (dox), then, 16h before the measurement dox was added at the indicated concentration. Cell fluorescence was measured by flow cytometry. The graphs show the average of three experiments with SEM. Below the graphs the fluorescence profile (fluorescence intensity plotted against the cell count) and a micrograph of the assayed cells for one representative experiment is shown. The micrograph shows the overlay of fluorescence and DIC thus giving the proportion of fluorescent cells. Scale bars are 20 μm (C-D).
(TIF)

S3 Fig Validation of knock-ins at the *act5* locus. **Homogenous expression through single copy integration**. (A) Scheme for the integration of the *act*5-KI construct of mScarlet (pPI419) into the *act*5 gene locus. The recombination arms are displayed in orange, mScarlet in red, the *act*5 gene and the Hygromycin resistance cassette in white. In black the upstream and downstream regions of the *act*5 gene are highlighted. Arrows indicate the binding sites for the primers outside the KI construct employed to verify positive clones. The plasmid was linearized with *Ngo*MIV before transfection. (B) Overview of the genomic region around the *act*5 gene. Shown by arrows are the *Bcl*I restrictions sites, which were used for Southern blotting analyses. The hybridisation site of the probe, which spans the 3’UTR and reaches into the coding sequence of the next gene, is outlined as a thick black line. The recombination arms, the *act*5 gene and the Hygromycin resistance cassette are coloured like described in (A). The expected fragment sizes detected by southern blotting are shown as double headed arrows. (C) Screening PCRs of two independent mScarlet *act*5 KIs with wild type AX3 control. All primer combinations are locus specific. The outer primer binding sites are located outside the recombination arms. D) Southern Blot of two independent mScarlet *act*5 KIs with wild type AX3 control. The wild type shows the predicted 5.8 kb band. Both *act*5 KIs possess a single 3.4 kb band showing the correct single insertion, which results from an additional *Bcl*I site in the Hygromycin resistance cassette (The blot has been cropped. The unmodified original is shown in the S3 File). (E) For each mScarlet *act*5 KI, 50,000 cells were analysed by flow cytometry using a YG582 filter to measure mScarlet fluorescence. AX3 cells were used as negative control. (F) Quantification of cell fluorescence intensity from the flow cytometry data shown in (E). The average of the median fluorescence intensity of three independent measurements per cell line is shown with fluorescence intensity in arbitrary units. Error bars indicate the SEM.
(TIF)

S4 Fig Knock-in at the *act5* locus in different *Dictyostelium* strains reproducibly yields high expression with minimal cell-to-cell variability. (A) Images of four independent *act5*::mCherry knock-ins. Clones were generated in the NC4 background using pDM1514 and images taken by confocal microscopy. An overlay of the red fluorescence channel and the DIC is shown. Scale bars are 20 μm. (B) Quantification of cell-to-cell variation in expression. In each case 50 individual cells per clone were analysed, with results shown in arbitrary units. The error bars indicate the SD. Single cells are shown as individual red dots while the median is displayed as a black line. (C) Histogram of the fluorescence intensity of the population of three commonly used strains (AX2, DdB and NC4) transfected with the *act5* knock-in vector pDM1514 and measured by flow cytometry. Four independent clones per strain are shown, for each of which 50,000 cells were analysed using a YG610 filter to measure mCherry fluorescence. (D) Quantification of cell fluorescence intensity from the flow cytometry data shown in (C). The average of the median fluorescence intensity of three independent measurements per cell line is shown with fluorescence intensity in arbitrary units. Error bars indicate the SEM.
(TIF)

S5 Fig Comparison of the fluorescence intensity of GFP expressed as an *act5* knock-in before and after removal of the resistance cassette, and from an extra-chromosomal expression vector. (A) Flow cytometry analysis of cellular fluorescence of four independent *act5*::GFP knock-in clones obtained by transfection of pDM1513 in AX2 cells. (B) Quantification of the experiment shown in (A) replicated 3 times. (C) Comparison of cell-to-cell fluorescence of knock-in clones, measured by confocal microscopy. (D) Flow cytometry analysis of cellular fluorescence of *act5*::GFP knock-in clone 5 before and after removal of the resistance cassette by expression of Cre-recombinase. (E) Quantification of the experiment shown in (D) replicated 3 times. (F) Comparison of cell-to-cell fluorescence of *act5*::GFP knock-in clone 5 before and after removal of the resistance cassette by expression of Cre-recombinase, measured by confocal microscopy. (G) Flow cytometry analysis of cellular fluorescence of an *act5*::GFP knock-in clone and a population of cells expressing GFP from an extra-chromosomal plasmid (pDM1207). This clearly shows the more variable but more intense expression obtained from the extra-chromosomal plasmid. (H) Quantification of the experiment shown in (G) replicated 3 times. (I) Comparison of cell-to-cell fluorescence of *act5*::GFP knock-in clone 5 and extra-chromosomally expressed GFP, measured by confocal microscopy. (J) Confocal microscopic images of cells from a GFP (*act5*) knock-in clone and from a population of cells expressing GFP from an extra-chromosomal vector. Scale bars are 20 μm. For Flow cytometry, 50,000 cells were analysed for each sample using the B-525 filter for GFP. The bar graphs show the average of the median fluorescence intensities, with SEM of 3 FACS experiments. The bee swarm plots show the fluorescence intensities of 50 individual cells for each condition, measured from confocal micrographs.
(TIF)

S6 Fig Organelle markers: *act5* knock-in of histone H2B as a nuclear marker. (A) Flow cytometry analysis of *act5*::H2B-mCherry KI clones, generated in an AX2 background. (B) Quantification of the experiment shown in (A), replicated 3 times. (C) Comparison of cell-to-cell fluorescence of knock-in clones, measured by confocal microscopy (50 cells each). (D) Flow cytometry analysis of *act5*::H2B-mCherry KI clones before and after removal of the resistance cassette by expression of Cre-recombinase. (E) Quantification of the experiment shown in (D), replicated 3 times. (F) Images of *act5*::H2B-mCherry expressing cells before and after resistance cassette removal. Scale bars are 20 μm. (G) Quantification of the images shown in S6F Fig. Each cell is shown as a red dot, with median shown as a black line, error bars and SD (n = 50). Flow cytometry of mCherry-expressing cells used the YG610 filter (n = 50,000 cells). Error bars of bar graphs indicate SEM (n = 3). The bee swarm plots show the fluorescence intensities of 50 individual cells (red dots) for each condition, measured from confocal micrographs, with median shown as a black line, error bars and SD (n = 50).
(TIF)

S7 Fig Alternative knock-in system allowing step-wise assembly. (A) Scheme of an alternative knock-in system. The two recombination-arms are shown in blue. The 5’ arm is cloned using the *BglII*/*SpeI* sites while the 3’ arm is added using *SalI*/*SacI*. The *SpeI* site directly follows the desired tag (light green). The cloned knock-in is terminated by an *act8*-terminator (pink). The resistance cassette (violet arrow) is driven by the *coaA*-promotor (dark green) and terminated by the *mhcA*-terminator (pink). The resistance cassette is flanked by loxP sites represented by yellow triangles. The vectors can be linearized with *Bgl*II or *Sac*I (B) List of vectors for a targeted in-frame insertion. They are ordered by resistance marker and encoded fluorescent protein.
(TIF)

S8 Fig Knock-in to the HSPC300 component of the SCAR/WAVE complex. (A) Confocal micrographs of randomly moving cells of HSPC300-GFP knock-in clones grown in bacterial suspension. Four independent clones show similar patterns and near-equal fluorescence intensities. Scale bar 10 μm. (B) Fluorescence intensity of individual cells of the four clones was determined and shown in panel (A). The median is displayed as a black line and error bars show SD.
(TIF)

S9 Fig The protocols for bacterially grown cells are also efficient under axenic conditions. (A) Efficiencies of knock-out generation, knock-in to the *act5* safe locus and knock-in to targeted loci. The number of correct clones is plotted against the total number of clones obtained. Knock-outs are displayed in blue, *act5* knock-ins in white and targeted knock-ins in black. (B) Stable cell lines expressing *act5*::mCherry in an AX2 background were created using pDM1514. Cells were cultured in HL5 or on bacteria. The whole-cell fluorescence of four clones each was measured by flow cytometry using the YG610 filter for mCherry fluorescence. 50,000 cells per cell line were analysed. Shown is the average of three experiments with SEM. (C) AX2 based *act5*::LifeAct-GFP or *act*5::LifeAct-mCherry expressing cells, cultured in HL5 or in bacterial suspension. Scale bar 20 μm. (D) Comparison of fluorescence variation of *act5*-knock-in and extra-chromosomally expressed LifeAct fusion proteins in AX2 cells. The cells were cultured in HL5. To compensate for the general differences of the fluorescence level due to the expression system used, the median of *act5*:: KI and extra-chromosomal expression was set to 1. Every cell is represented as a single dot, with 50 cells per condition. Error bars show the SD while the central line represents the median.
(TIF)

S1 Table Cell lines used in the paper. (DOCX)

S2 Table Other plasmids used in the paper. (DOCX)

S3 Table Oligonucleotides used in this paper. (DOCX)

S4 Table Transformations in HL5 medium using the new established electroporation conditions. (DOCX)

S1 Movie Cultivation of *Dictyostelium discoideum* cells in bacterial suspension (OD = 2). (MOV)

S2 Movie *Dictyostelium discoideum* feeding on *Klebsiella aerogenes* bacteria. (AVI)

S3 Movie *act5*::H2B-mCherry expressing AX2 cells in SorMC buffer. (AVI)

S1 File DNA sequences of the plasmids. (PDF)

S2 File Southern Blot *ras*S- clones. (TIF)

S3 File Southern Blot *act5*::mScarlet KI clones. (TIF)

S1 Protocol Generation of KOs. (PDF)

S2 Protocol Preparation of food bacteria. (PDF)

S3 Protocol REMI expression. (PDF)

S4 Protocol Extrachromosomal expression. (PDF)
