# Supplementary figures and images for: Rapid and efficient genetic engineering of both wild type and axenic strains of Dictyostelium discoideum
Source: PLoS One. 2018 May 30;13(5):e0196809. doi: 10.1371/journal.pone.0196809 (PMC5976153; doi:10.1371/journal.pone.0196809)

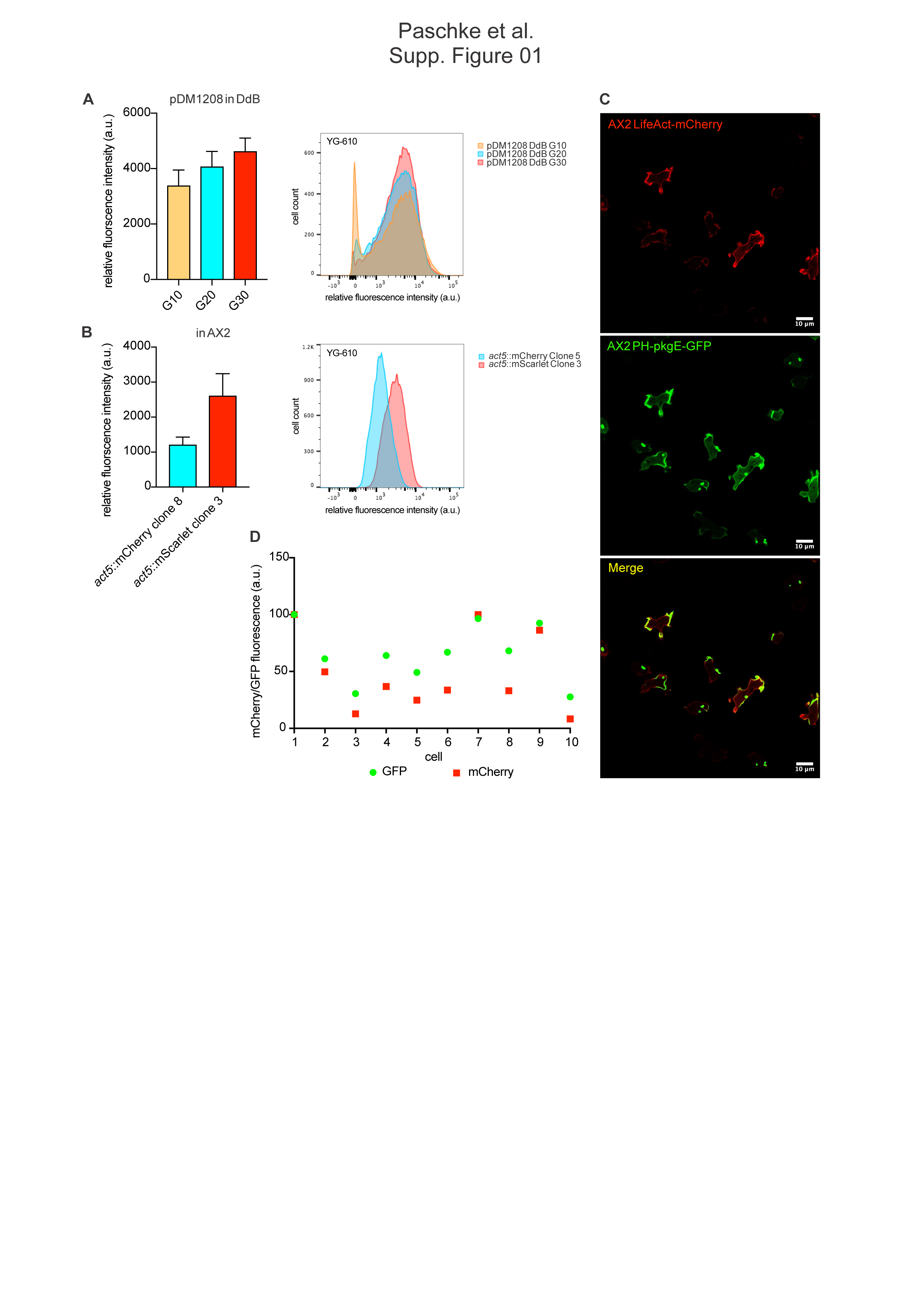

Supplement: S1 Fig — (A) Quantification of mCherry fluorescence intensity after transfection of the extrachromosomal plasmid pDM1208 in DdB cells. Cells were selected with different concentrations of G418 (10 μg to 30 μg/ml). Bar diagrams show the average of the mean fluorescence intensity measured using flow cytometry. The experiment was repeated three times; error bars indicate the SEM. On the right a typical fluorescence profile is displayed, showing the relative mCherry fluorescence plotted against the cell count. (B) Comparison of whole cell fluorescence intensity of cells expressing mCherry or mScarlet. On the left the relative fluorescence of a clonal cell line expressing act5::mCherry or act5::mScarlet are shown. The bar diagrams show the fluorescence measured by flow cytometry, using a YG610 filter. The error bars represent the SEM. On the right the fluorescence profile for a representative experiment is shown. Fluorescence intensity is plotted against the cell count. Highlighted in blue is an act5 driven mCherry and in red an mScarlet clone. (C) Images of AX2 cells expressing a LifeAct-mCherry/PH-pkgE-GFP (pPI304) double reporter for F-actin and PIP3. Cells were grown in bacterial suspension. Scale bars are 10 μm. (D) Correlation plot of mCherry and GFP fluorescence of the cells imaged in (C). (TIF) [file pone.0196809.s001.tif]

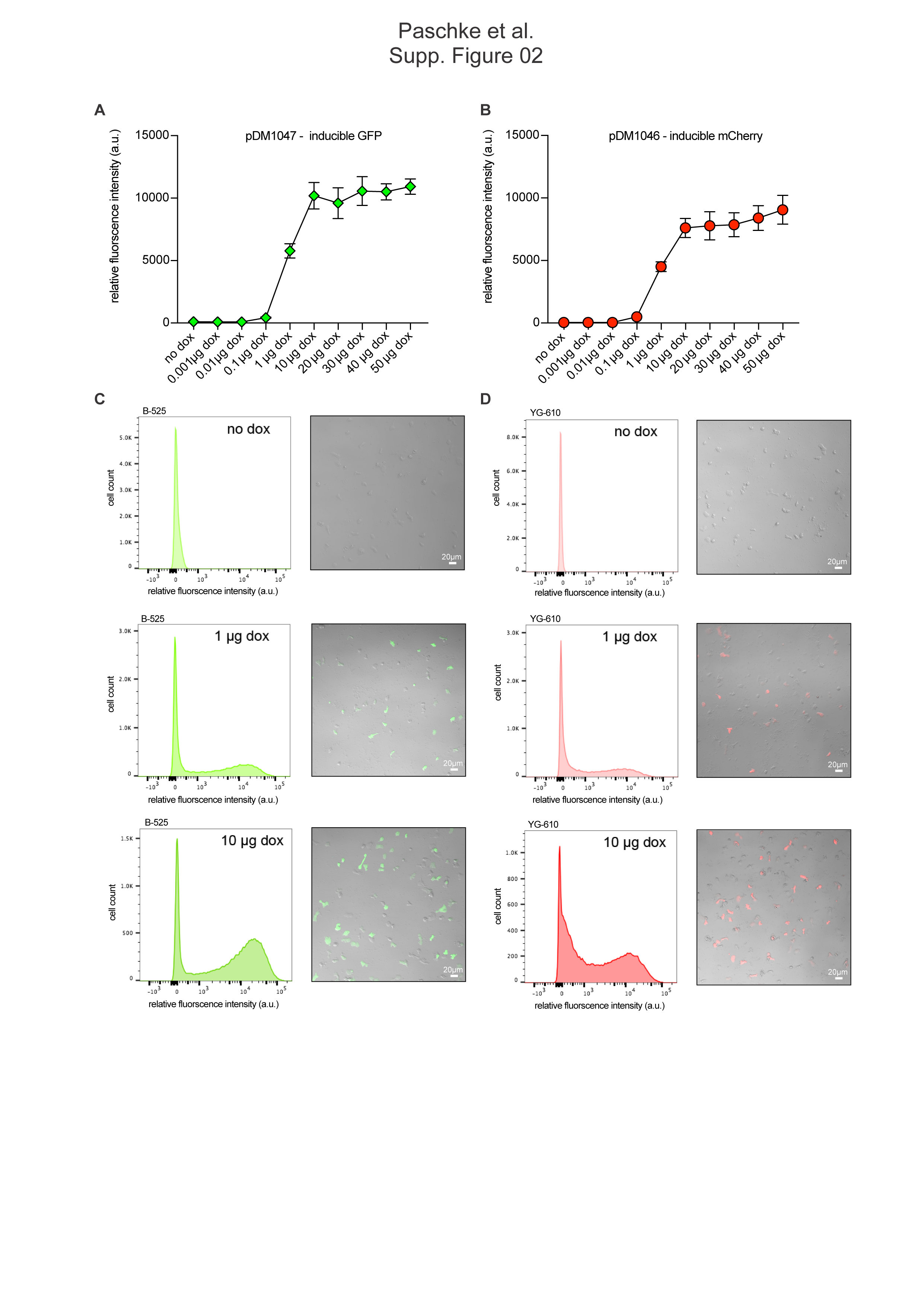

Supplement: S2 Fig — Adaptation of the doxycycline inducible expression system to cells grown on bacteria. (A-B) Dose-response curves for GFP (pDM1047) and mCherry (pDM1046) expression induced by doxycycline. NC4 cells were transfected with the respective plasmids and cultured in the absence of doxycycline (dox), then, 16h before the measurement dox was added at the indicated concentration. Cell fluorescence was measured by flow cytometry. The graphs show the average of three experiments with SEM. Below the graphs the fluorescence profile (fluorescence intensity plotted against the cell count) and a micrograph of the assayed cells for one representative experiment is shown. The micrograph shows the overlay of fluorescence and DIC thus giving the proportion of fluorescent cells. Scale bars are 20 μm (C-D). (TIF) [file pone.0196809.s002.tif]

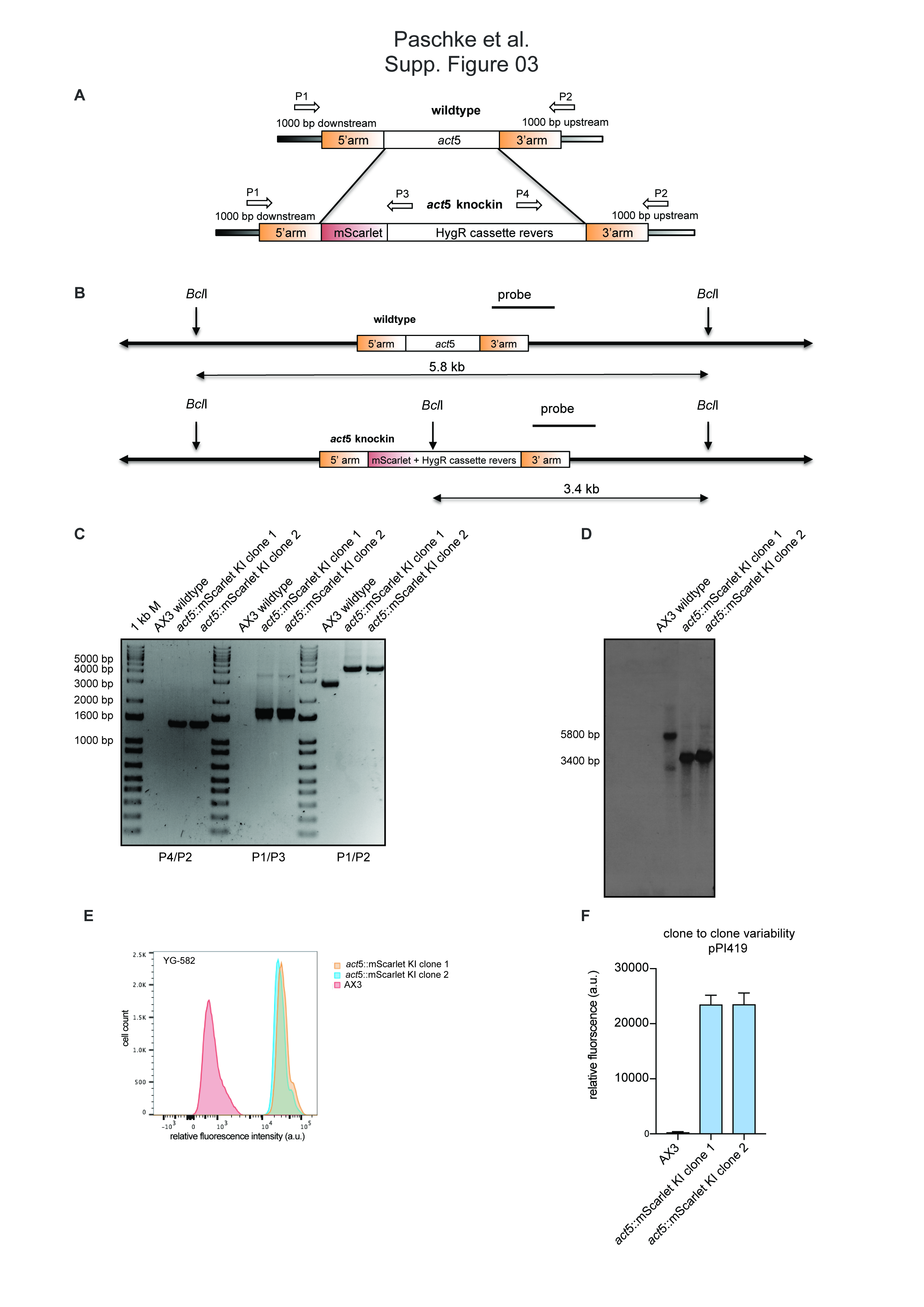

Supplement: S3 Fig — Homogenous expression through single copy integration. (A) Scheme for the integration of the act5-KI construct of mScarlet (pPI419) into the act5 gene locus. The recombination arms are displayed in orange, mScarlet in red, the act5 gene and the Hygromycin resistance cassette in white. In black the upstream and downstream regions of the act5 gene are highlighted. Arrows indicate the binding sites for the primers outside the KI construct employed to verify positive clones. The plasmid was linearized with NgoMIV before transfection. (B) Overview of the genomic region around the act5 gene. Shown by arrows are the BclI restrictions sites, which were used for Southern blotting analyses. The hybridisation site of the probe, which spans the 3’UTR and reaches into the coding sequence of the next gene, is outlined as a thick black line. The recombination arms, the act5 gene and the Hygromycin resistance cassette are coloured like described in (A). The expected fragment sizes detected by southern blotting are shown as double headed arrows. (C) Screening PCRs of two independent mScarlet act5 KIs with wild type AX3 control. All primer combinations are locus specific. The outer primer binding sites are located outside the recombination arms. D) Southern Blot of two independent mScarlet act5 KIs with wild type AX3 control. The wild type shows the predicted 5.8 kb band. Both act5 KIs possess a single 3.4 kb band showing the correct single insertion, which results from an additional BclI site in the Hygromycin resistance cassette (The blot has been cropped. The unmodified original is shown in the S3 File). (E) For each mScarlet act5 KI, 50,000 cells were analysed by flow cytometry using a YG582 filter to measure mScarlet fluorescence. AX3 cells were used as negative control. (F) Quantification of cell fluorescence intensity from the flow cytometry data shown in (E). The average of the median fluorescence intensity of three independent measurements per cell line is shown with fluores [file pone.0196809.s003.tif]

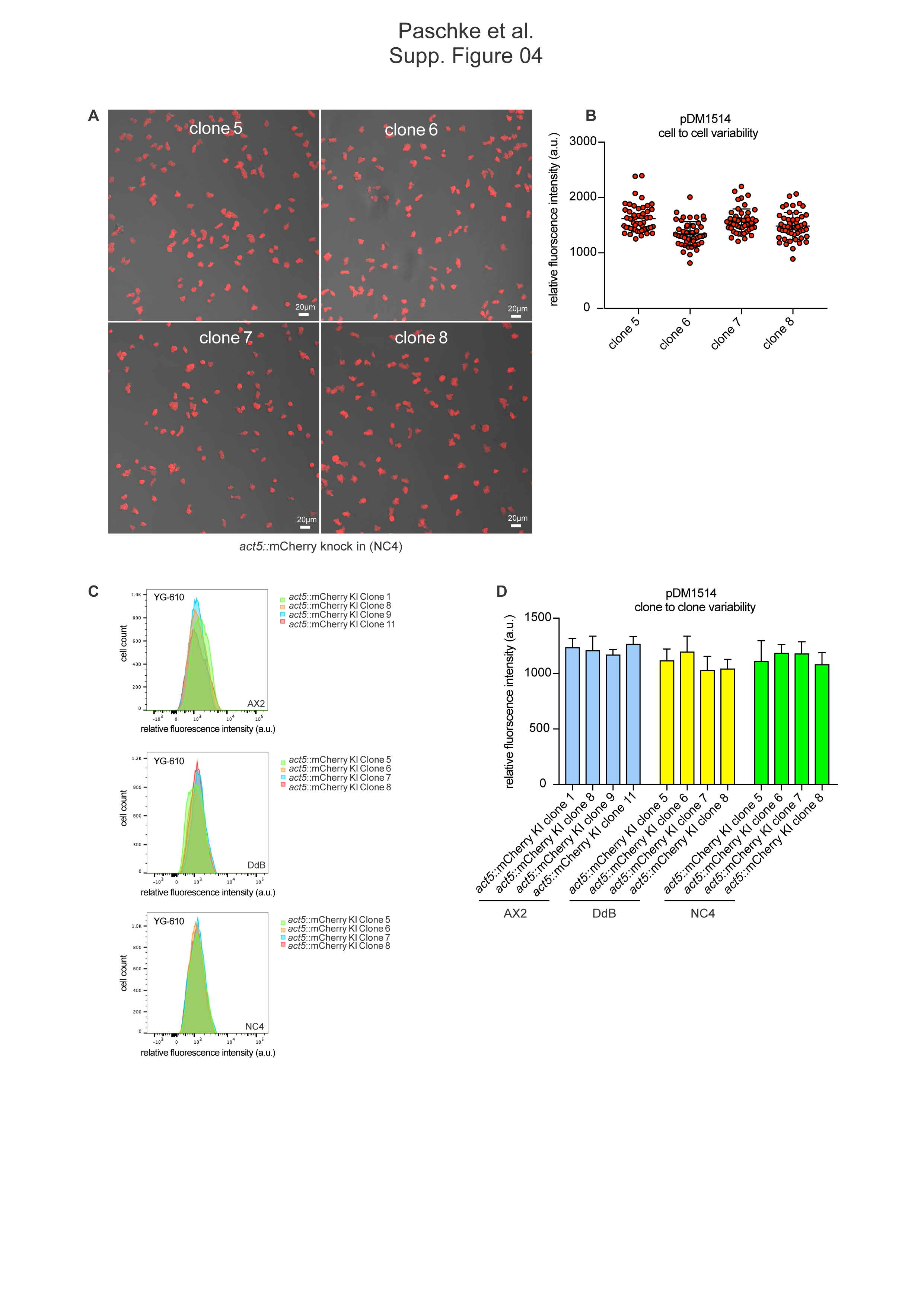

Supplement: S4 Fig — (A) Images of four independent act5::mCherry knock-ins. Clones were generated in the NC4 background using pDM1514 and images taken by confocal microscopy. An overlay of the red fluorescence channel and the DIC is shown. Scale bars are 20 μm. (B) Quantification of cell-to-cell variation in expression. In each case 50 individual cells per clone were analysed, with results shown in arbitrary units. The error bars indicate the SD. Single cells are shown as individual red dots while the median is displayed as a black line. (C) Histogram of the fluorescence intensity of the population of three commonly used strains (AX2, DdB and NC4) transfected with the act5 knock-in vector pDM1514 and measured by flow cytometry. Four independent clones per strain are shown, for each of which 50,000 cells were analysed using a YG610 filter to measure mCherry fluorescence. (D) Quantification of cell fluorescence intensity from the flow cytometry data shown in (C). The average of the median fluorescence intensity of three independent measurements per cell line is shown with fluorescence intensity in arbitrary units. Error bars indicate the SEM. (TIF) [file pone.0196809.s004.tif]

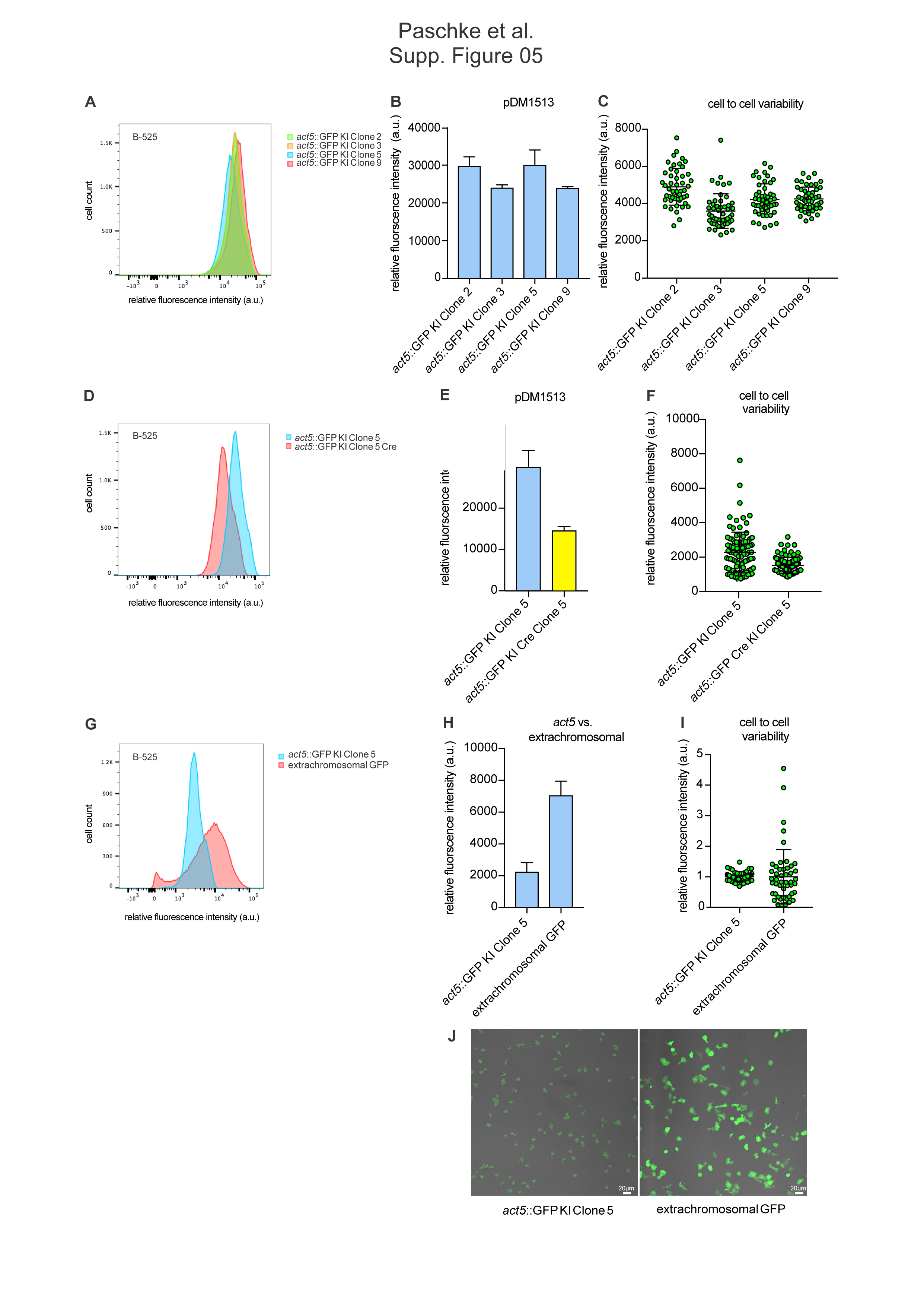

Supplement: S5 Fig — (A) Flow cytometry analysis of cellular fluorescence of four independent act5::GFP knock-in clones obtained by transfection of pDM1513 in AX2 cells. (B) Quantification of the experiment shown in (A) replicated 3 times. (C) Comparison of cell-to-cell fluorescence of knock-in clones, measured by confocal microscopy. (D) Flow cytometry analysis of cellular fluorescence of act5::GFP knock-in clone 5 before and after removal of the resistance cassette by expression of Cre-recombinase. (E) Quantification of the experiment shown in (D) replicated 3 times. (F) Comparison of cell-to-cell fluorescence of act5::GFP knock-in clone 5 before and after removal of the resistance cassette by expression of Cre-recombinase, measured by confocal microscopy. (G) Flow cytometry analysis of cellular fluorescence of an act5::GFP knock-in clone and a population of cells expressing GFP from an extra-chromosomal plasmid (pDM1207). This clearly shows the more variable but more intense expression obtained from the extra-chromosomal plasmid. (H) Quantification of the experiment shown in (G) replicated 3 times. (I) Comparison of cell-to-cell fluorescence of act5::GFP knock-in clone 5 and extra-chromosomally expressed GFP, measured by confocal microscopy. (J) Confocal microscopic images of cells from a GFP (act5) knock-in clone and from a population of cells expressing GFP from an extra-chromosomal vector. Scale bars are 20 μm. For Flow cytometry, 50,000 cells were analysed for each sample using the B-525 filter for GFP. The bar graphs show the average of the median fluorescence intensities, with SEM of 3 FACS experiments. The bee swarm plots show the fluorescence intensities of 50 individual cells for each condition, measured from confocal micrographs. (TIF) [file pone.0196809.s005.tif]

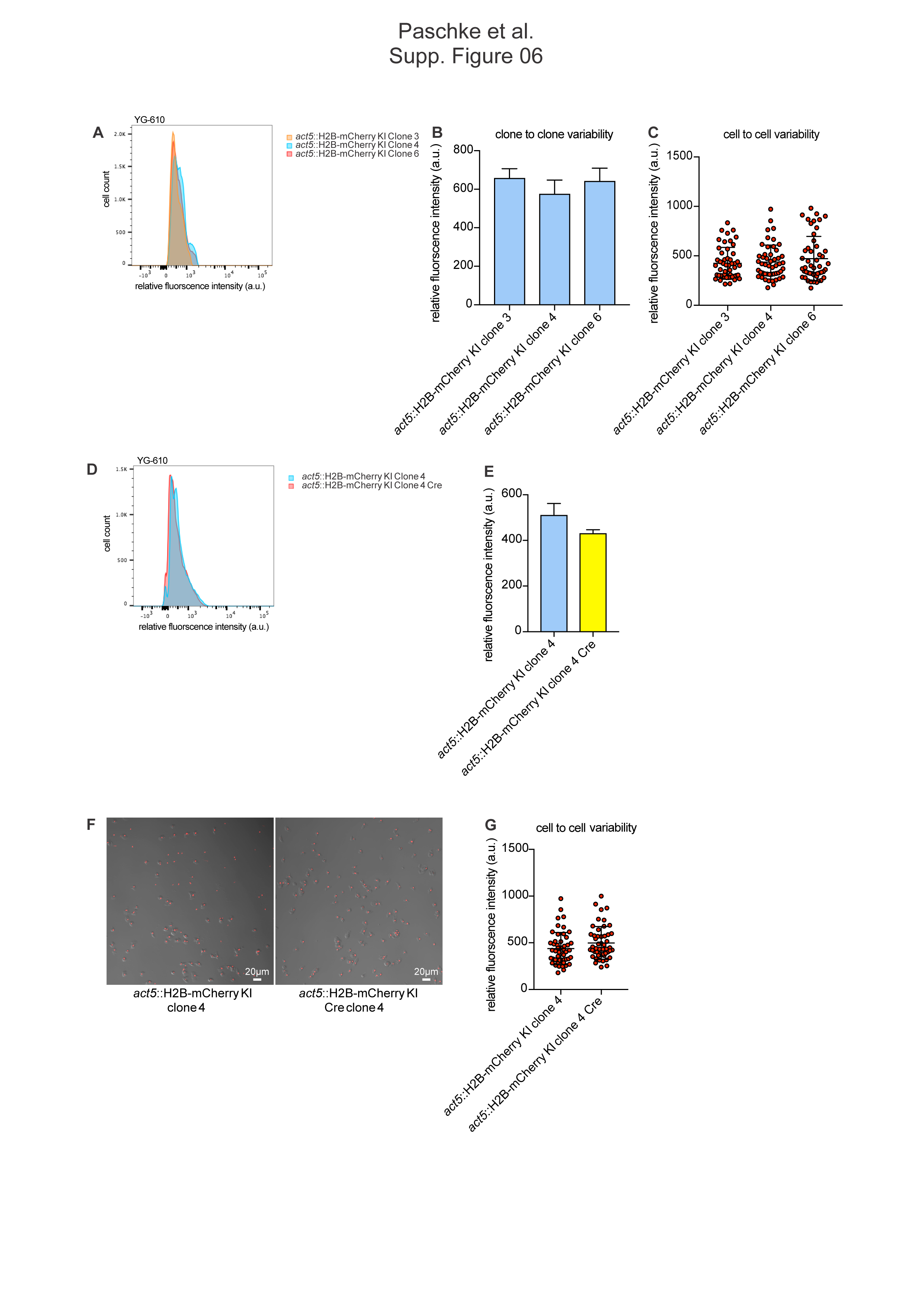

Supplement: S6 Fig — (A) Flow cytometry analysis of act5::H2B-mCherry KI clones, generated in an AX2 background. (B) Quantification of the experiment shown in (A), replicated 3 times. (C) Comparison of cell-to-cell fluorescence of knock-in clones, measured by confocal microscopy (50 cells each). (D) Flow cytometry analysis of act5::H2B-mCherry KI clones before and after removal of the resistance cassette by expression of Cre-recombinase. (E) Quantification of the experiment shown in (D), replicated 3 times. (F) Images of act5::H2B-mCherry expressing cells before and after resistance cassette removal. Scale bars are 20 μm. (G) Quantification of the images shown in S6F Fig. Each cell is shown as a red dot, with median shown as a black line, error bars and SD (n = 50). Flow cytometry of mCherry-expressing cells used the YG610 filter (n = 50,000 cells). Error bars of bar graphs indicate SEM (n = 3). The bee swarm plots show the fluorescence intensities of 50 individual cells (red dots) for each condition, measured from confocal micrographs, with median shown as a black line, error bars and SD (n = 50). (TIF) [file pone.0196809.s006.tif]

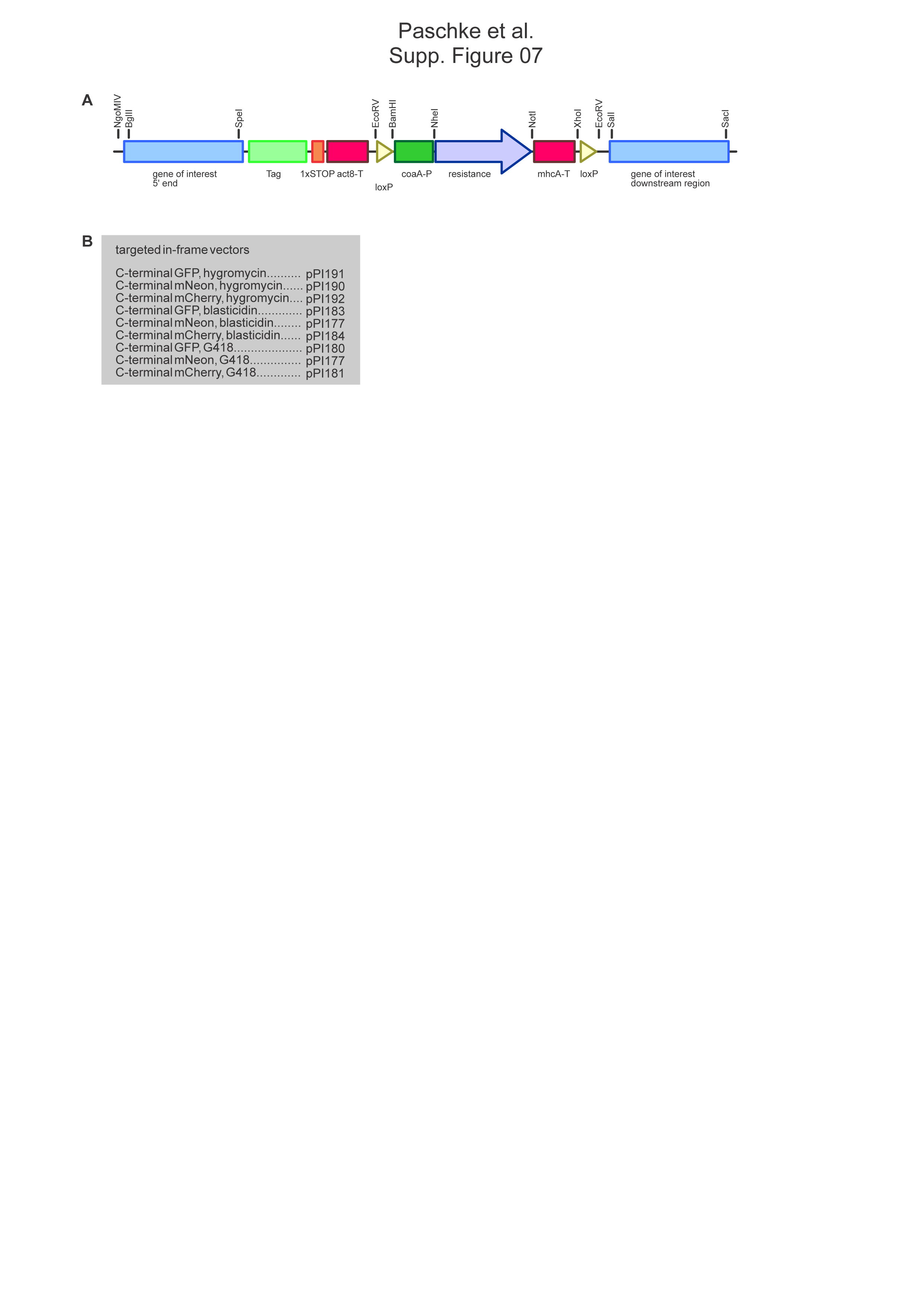

Supplement: S7 Fig — (A) Scheme of an alternative knock-in system. The two recombination-arms are shown in blue. The 5’ arm is cloned using the BglII/SpeI sites while the 3’ arm is added using SalI/SacI. The SpeI site directly follows the desired tag (light green). The cloned knock-in is terminated by an act8-terminator (pink). The resistance cassette (violet arrow) is driven by the coaA-promotor (dark green) and terminated by the mhcA-terminator (pink). The resistance cassette is flanked by loxP sites represented by yellow triangles. The vectors can be linearized with BglII or SacI (B) List of vectors for a targeted in-frame insertion. They are ordered by resistance marker and encoded fluorescent protein. (TIF) [file pone.0196809.s007.tif]

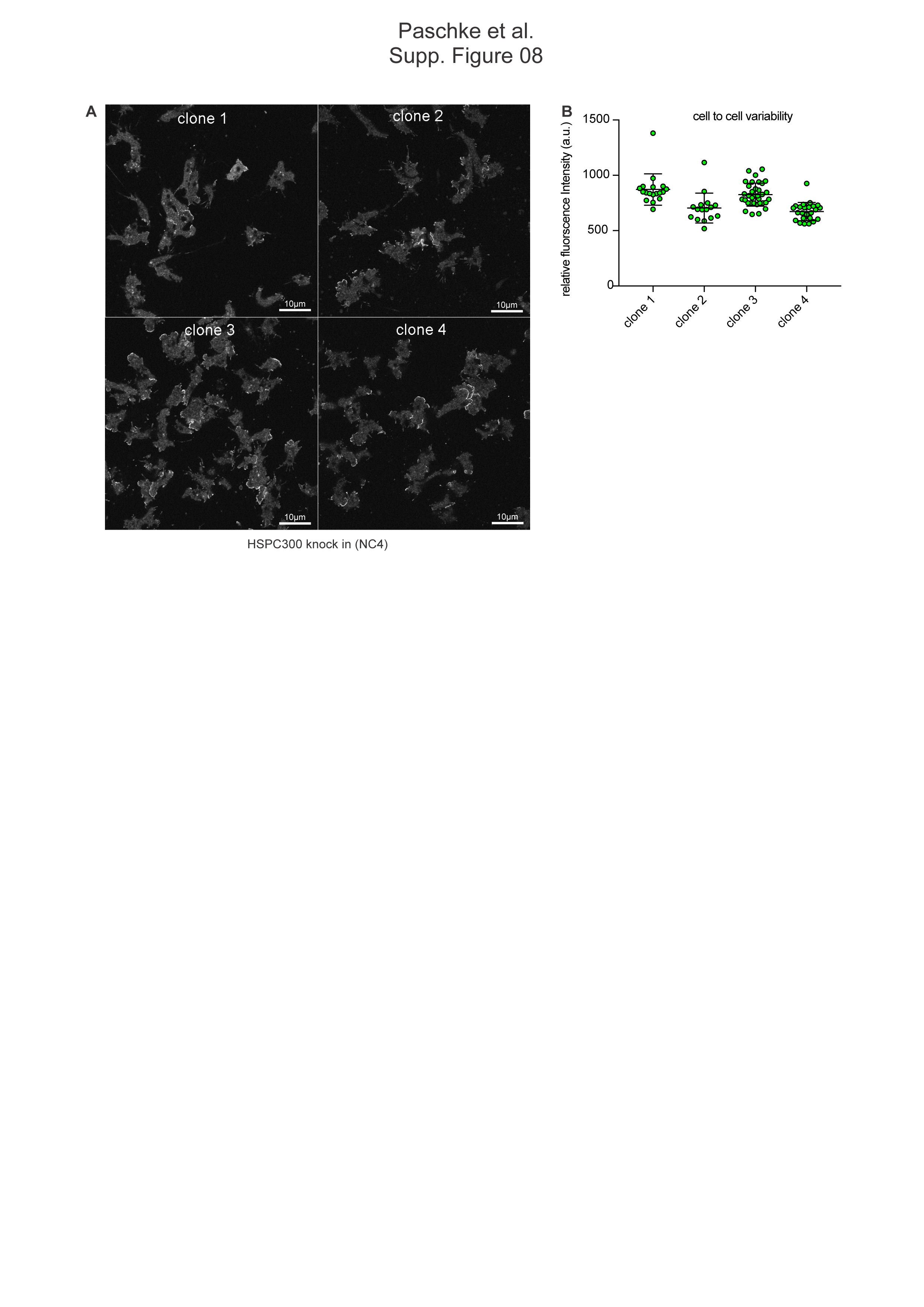

Supplement: S8 Fig — (A) Confocal micrographs of randomly moving cells of HSPC300-GFP knock-in clones grown in bacterial suspension. Four independent clones show similar patterns and near-equal fluorescence intensities. Scale bar 10 μm. (B) Fluorescence intensity of individual cells of the four clones was determined and shown in panel (A). The median is displayed as a black line and error bars show SD. (TIF) [file pone.0196809.s008.tif]

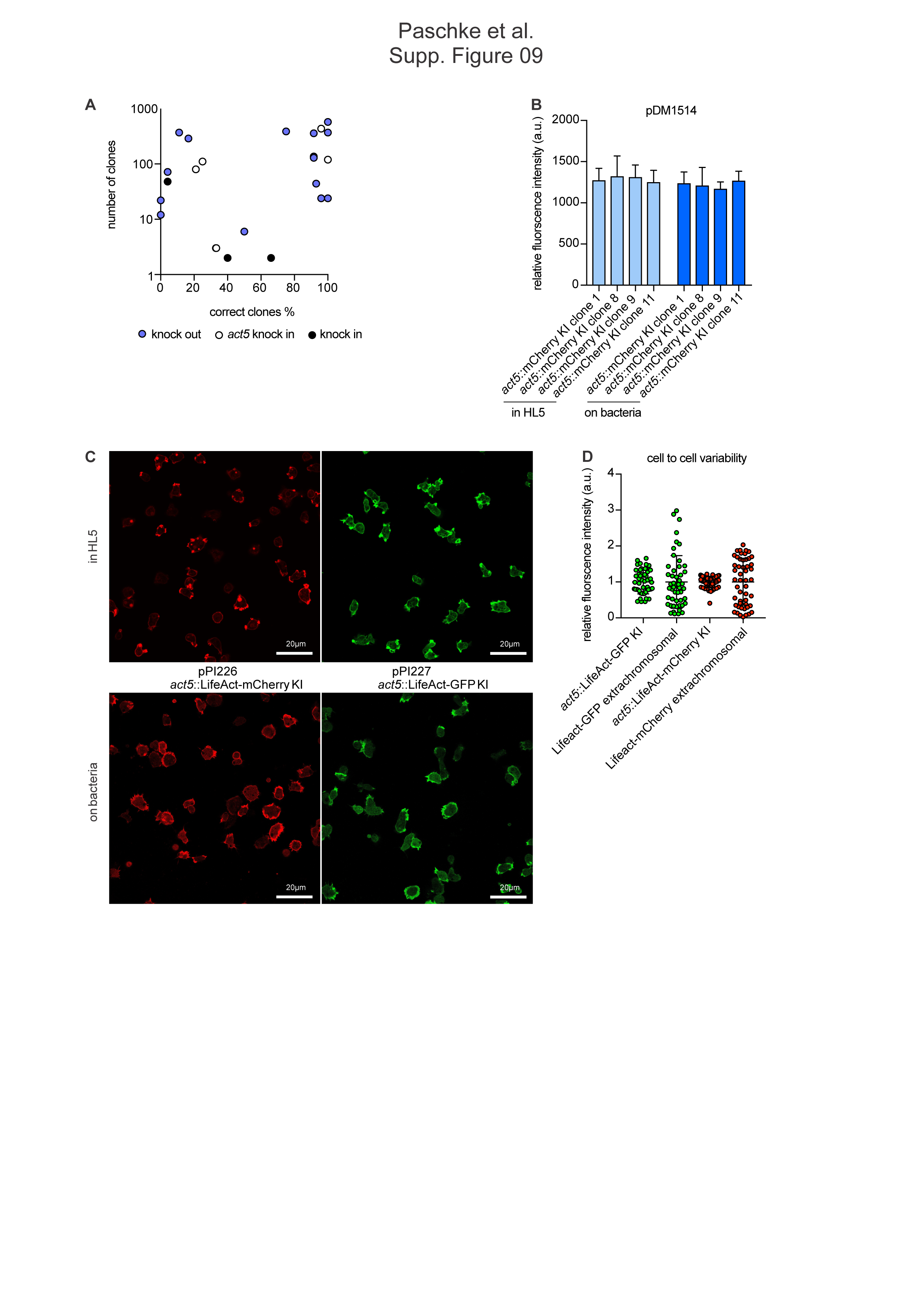

Supplement: S9 Fig — (A) Efficiencies of knock-out generation, knock-in to the act5 safe locus and knock-in to targeted loci. The number of correct clones is plotted against the total number of clones obtained. Knock-outs are displayed in blue, act5 knock-ins in white and targeted knock-ins in black. (B) Stable cell lines expressing act5::mCherry in an AX2 background were created using pDM1514. Cells were cultured in HL5 or on bacteria. The whole-cell fluorescence of four clones each was measured by flow cytometry using the YG610 filter for mCherry fluorescence. 50,000 cells per cell line were analysed. Shown is the average of three experiments with SEM. (C) AX2 based act5::LifeAct-GFP or act5::LifeAct-mCherry expressing cells, cultured in HL5 or in bacterial suspension. Scale bar 20 μm. (D) Comparison of fluorescence variation of act5-knock-in and extra-chromosomally expressed LifeAct fusion proteins in AX2 cells. The cells were cultured in HL5. To compensate for the general differences of the fluorescence level due to the expression system used, the median of act5:: KI and extra-chromosomal expression was set to 1. Every cell is represented as a single dot, with 50 cells per condition. Error bars show the SD while the central line represents the median. (TIF) [file pone.0196809.s009.tif]

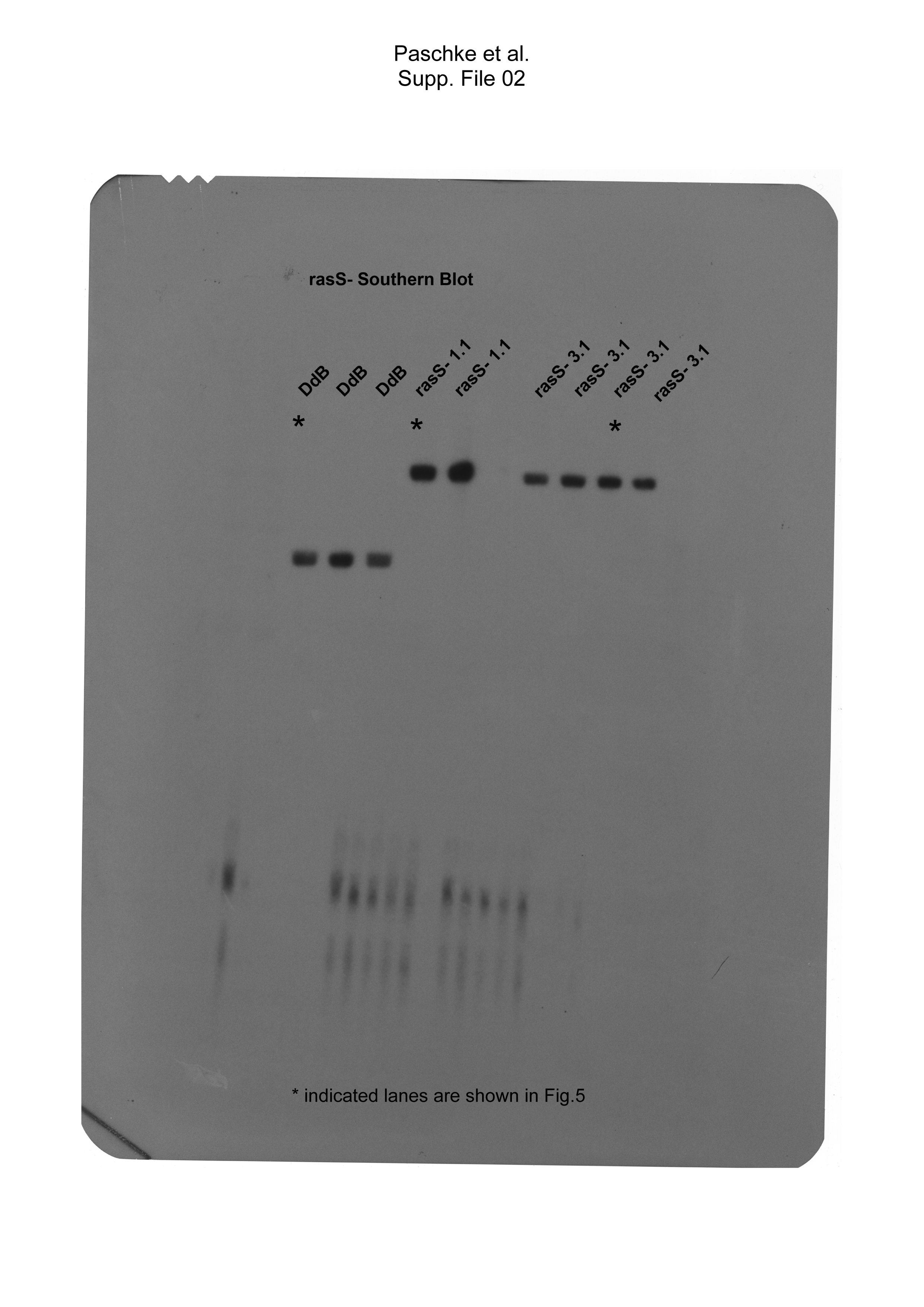

Supplement: S2 File — (TIF) [file pone.0196809.s018.tif]

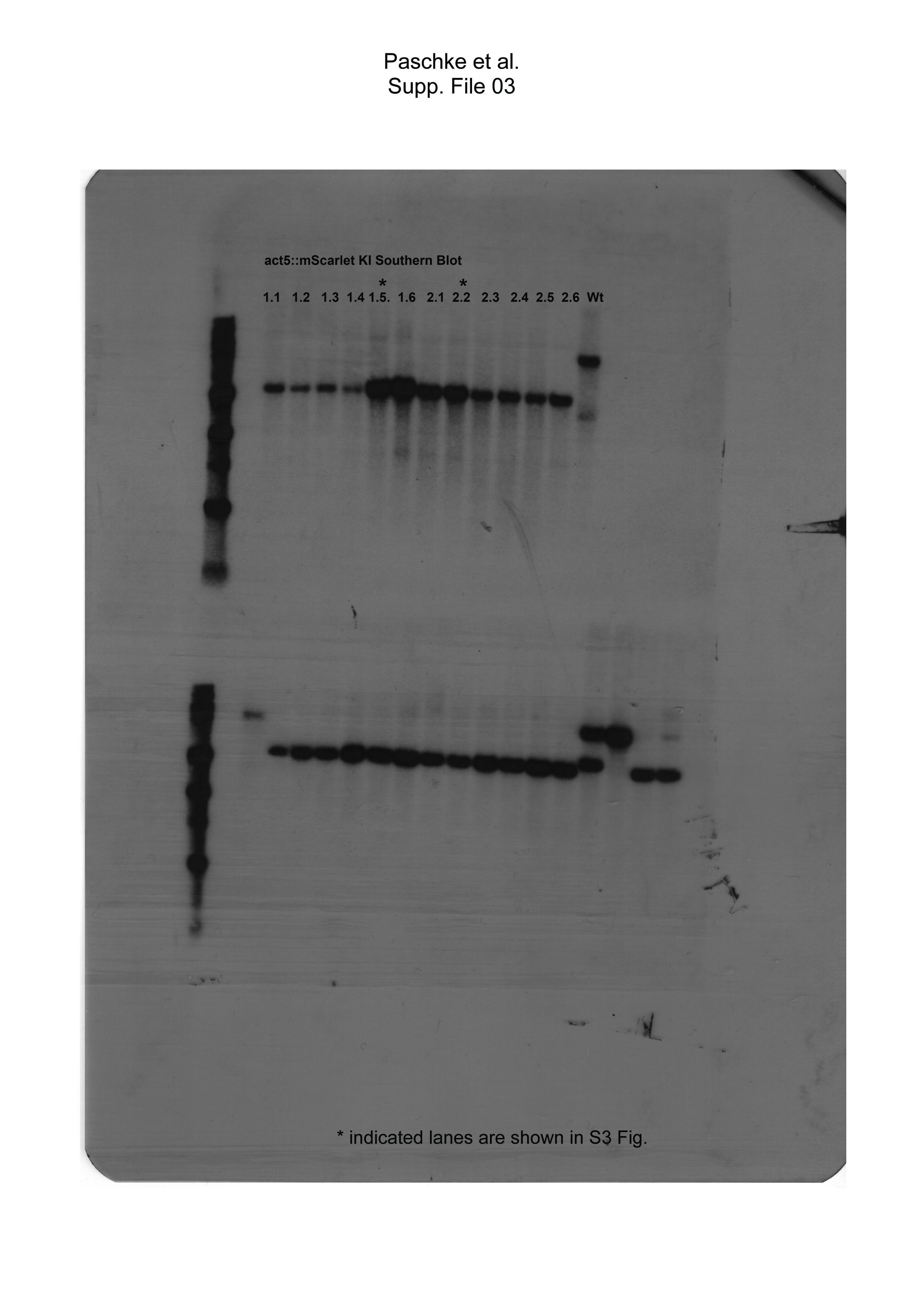

Supplement: S3 File — (TIF) [file pone.0196809.s019.tif]
