# Supplementary material for: Rapid and efficient genetic engineering of both wild type and axenic strains of Dictyostelium discoideum
Source: PLoS One. 2018 May 30;13(5):e0196809. doi: 10.1371/journal.pone.0196809 (PMC5976153; doi:10.1371/journal.pone.0196809)
Supplement: S1 Table — (DOCX) [file pone.0196809.s010.docx]

**S1 Table.**

**Cell lines used in the paper**

| Cell line | Genetically background | Reference number | Published before | Type |
| --- | --- | --- | --- | --- |
| AX2 (Ka) |  | DBS0235521 |  | wild type |
| AX3 (K) |  | DBS0235545 |  |  |
| DdB (Wel) |  | DBS0350772 | Bloomfield *et al*, 2015 | wild type |
| NC4 (S) |  |  |  | wild type |
| HSPC300-GFP  Clone 1 | NC4 | HM1807 | This paper | knock in |
| HSPC300-GFP  Clone 2 | NC4 | HM1808 | This paper | knock in |
| *act*5::mCherry  Clone 5 | NC4 | HM1912 | This paper | *act*5 knock in |
| *act*5::mCherry  Clone 6 | NC4 | HM1913 | This paper | *act*5 knock in |
| *act*5::mCherry  Clone 7 | NC4 | HM1914 | This paper | *act*5 knock in |
| *act*5::mCherry  Clone 8 | NC4 | HM1915 | This paper | *act*5 knock in |
| *act*5::mCherry  Clone 5 | DdB | HM1916 | This paper | *act*5 knock in |
| *act*5::mCherry  Clone 6 | DdB | HM1917 | This paper | *act*5 knock in |
| *act*5::mCherry  Clone 7 | DdB | HM1918 | This paper | *act*5 knock in |
| *act*5::mCherry  Clone 8 | DdB | HM1919 | This paper | *act*5 knock in |
| *act*5::mCherry  Clone 1 | AX2 | HM1935 | This paper | *act*5 knock in |
| *act*5::mCherry  Clone 8 | AX2 | HM1936 | This paper | *act*5 knock in |
| *act*5::mCherry  Clone 9 | AX2 | HM1937 | This paper | *act*5 knock in |
| *act*5::mCherry  Clone 11 | AX2 | HM1938 | This paper | *act*5 knock in |
| *act*5::mCherry  Clone 8 CRE | AX2 | HM1939 | This paper | *act*5 knock in |
| *act*5::GFP  Clone 2 | AX2 | HM1930 | This paper | *act*5 knock in |
| *act*5::GFP  Clone 3 | AX2 | HM1931 | This paper | *act*5 knock in |
| *act*5::GFP  Clone 5 | AX2 | HM1932 | This paper | *act*5 knock in |
| *act*5::GFP  Clone 9 | AX2 | HM1933 | This paper | *act*5 knock in |
| *act*5::GFP  Clone 5 CRE | AX2 | HM1934 | This paper | *act*5 knock in |
| *act*5::mScarlet  Clone 3 | AX2 | HM1941 | This paper | *act*5 knock in |
| *act*5::mScarlet  Clone 1.5 | AX3 | HM1961 | This paper | *act*5 knock in |
| *act*5::mScarlet  Clone 2.2 | AX3 | HM1962 | This paper | *act*5 knock in |
| *act*5::LifeAct-mCherry Clone 1 | AX2 | HM1928 | This paper | *act*5 knock in |
| *act*5::LifeAct-GFP  Clone 1 | AX2 | HM1929 | This paper | *act*5 knock in |
| *act*5::H2B-mCherry Clone 3 | AX2 | HM1904 | This paper | *act*5 knock in |
| *act*5::H2B-mCherry Clone 4 | AX2 | HM1905 | This paper | *act*5 knock in |
| *act*5::H2B-mCherry Clone 6 | AX2 | HM1909 | This paper | *act*5 knock in |
| *ras*S-  Clone 1-3 | DdB | HM1920 | This paper | knock out |
| *ras*S-  Clone 3-1 | DdB | HM1921 | This paper | knock out |
