# Supplementary material for: Rapid and efficient genetic engineering of both wild type and axenic strains of Dictyostelium discoideum
Source: PLoS One. 2018 May 30;13(5):e0196809. doi: 10.1371/journal.pone.0196809 (PMC5976153; doi:10.1371/journal.pone.0196809)
Supplement: S2 Table — (DOCX) [file pone.0196809.s011.docx]

**S2 Table.**

**Other plasmids used in the paper**

| Plasmid | Type | Reference number | Published before | resistance |
| --- | --- | --- | --- | --- |
| HSPC300-GFP | knock-in | pDM1476 | This paper | HygR |
| *ras*S-KO | knock-out | pPI187 | This paper | HygR |
| H2B-GFP | extrachromosomal | pPI278 | This paper | G418R |
| LifeAct-GFP | extrachromosomal | pPI138 | This paper | G418R |
| *act5*-H2B-mCherry | *act*5 knock-in | pPI420 | This paper | HygR |
| *act5*-LifeAct-mCherry | *act*5 knock-in | pPI226 | This paper | HygR |
| *act5*-LifeAct-GFP | *act*5 knock-in | pPI227 | This paper | HygR |
| LifeAct-mCherry/PH-pkgE-GFP | extrachromosomal | pPI304 | This paper | G418R |
