# Supplementary material for: Rapid and efficient genetic engineering of both wild type and axenic strains of Dictyostelium discoideum
Source: PLoS One. 2018 May 30;13(5):e0196809. doi: 10.1371/journal.pone.0196809 (PMC5976153; doi:10.1371/journal.pone.0196809)
Supplement: S3 Table — (DOCX) [file pone.0196809.s012.docx]

**S3 Table.**

**Oligonucleotides used in this paper**

| Name | Sequence |
| --- | --- |
| oDM1015  cross over | CTTCAGTAGGCAGAGCTATC |
| oDM1016  cross over | CAGCTTGGATACTCCAGTAG |
| oPI118  5-*Bam*HI-LifeAct-GFP | GGATCCAAAATGGGAGTAGCTGATTTAATAAAAAAATTTG |
| oPI119  3-*Spe*I-LifeAct-GFP | ACTAGTTTACTTGTATAGTTCATCCATGCCATG |
| oPI120  3-*Spe*I-LifeAct-mCherry | GCACTAGTTTATTTATATAATTCATCCATACCACCTG |
| oPI146  RasS-5-Prime-  revers | GATAGCTCTGCCTACTGAAGGATTTACCAACACCACCTGGTCCAAC |
| oPI147  RasS-3-Prime-forward | CTACTGGAGTATCCAAGCTGCTCAAGAACAAAATACTGATCAAC |
| oPI148  RasS-5-Prime-outer | CATATTGAAATCTAGATATAACCGAC |
| oPI149  RasS-3-Prime-outer | CAAGAGGCAAGAGAATTTGTATCATTCATG |
| oPI150  RasS-5-Prime-inner | GCCAGCTGCTAGATATAACCGACTCTATC |
| oPI151  RasS-3-Prime-inner | GGCAGCTGCATGGTGAATTCAAGATGAACATG |
| oPI155  Resistance-Seq-5-Prime | GATAGCTCTGCCTACTGAAG |
| oPI156  Resistance-Seq-3-Prime | CTACTGGAGTATCCAAGCTG |
| oPI223  Act5-Screening-5-Prime | TTGGTAATATTAAACAAGAATTAGAGATT |
| oPI224  Act5-Screening-3-Prime | CATTAAAGAATTTGGAAAATTTTAAAATTGAATCAAAC |
| oPI229  H2B-*Bam*HI | ATGGATCCAAAATGGTATTCGTTAAAGGTCAAAAGAAAG |
| oPI230  H2B-*Spe*I | GCACTAGTGTTTTTGCTTTCAGTTGGATTGT |
| oPI236  Terminator-Primer-3 | ATCTCGAGAGTAGTATAACTTCGTATAGCATAC |
| oPI237  Promotor-Primer-5 | CAAATAAAGAGTTATAATAATATACAGTTGA |
