# Supplementary material for: Rapid and efficient genetic engineering of both wild type and axenic strains of Dictyostelium discoideum
Source: PLoS One. 2018 May 30;13(5):e0196809. doi: 10.1371/journal.pone.0196809 (PMC5976153; doi:10.1371/journal.pone.0196809)
Supplement: S4 Table — (DOCX) [file pone.0196809.s013.docx]

**S4 Table**

**Transformations in HL5 medium using the new established electroporation conditions**

| Disrupted gene | Number of transfectants | Checked clones | Correct clones | % of correct clones | *Dictyostelium* strain used |
| --- | --- | --- | --- | --- | --- |
| *myo*F | 44 | 44 | 41 | 93.1 | AX2 |
| *myo*K | 24 | 24 | 23 | 95.8 | AX2 |
| *myo*B | 24 | 24 | 23 | 95.8 | AX2 |
| *myo*F | 24 | 24 | 24 | 100 | AX2 *myo*E- |
| *myo*D | 6 | 6 | 3 | 50 | AX2 *my*oE- |
| *myo*A | 22 | 22 | 0 | 0 | AX2 |
| *for*C | 371 | 36 | 4 | 11.1 | AX2 |
| *myo*B | 370 | 12 | 12 | 100 | AX2 *myo*E- |
| *myo*F | 576 | 12 | 12 | 100 | AX2 *myo*E- |
| *myo*A | 12 | 12 | 0 | 0 | AX2 |
| *for*C | 288 | 24 | 4 | 16.6 | AX2 *for*G- |
| *myo*D | 129 | 12 | 11 | 91.6 | AX2 *myo*E/F- |
| *myo*B | 136 | 12 | 11 | 91.6 | AX2 *myo*E/F- |
| *myo*D | 359 | 12 | 11 | 91.6 | AX2 *myo*B/E- |
| *myo*F | 388 | 12 | 9 | 75 | AX2 *myo*B/E- |

| Targeted gene & tag | Number of transfectants | Checked clones | Correct clones | % of correct clones | *Dictyostelium* strain used |
| --- | --- | --- | --- | --- | --- |
| CARMIL-GFP | 5 | 5 | 2 | 40 | AX2 |
| CARMIL-mCherry | 6 | 6 | 2 | 33.3 | AX2 |
| TalA-mNeon | 48 | 48 | 2 | 4.1 | AX2 |
| TalA-GFP | 3 | 3 | 1 | 33.3 | AX2 |

| Targeted construct into the *act5* locus | Number of transfectants | Checked clones | Correct clones | % of correct clones | *Dictyostelium* strain used |
| --- | --- | --- | --- | --- | --- |
| LifeAct-mRFPmars2 | 120 | 12 | 12 | 100 | AX2 |
| PH-CRAC-mCherry | 11 | 12 | 3 | 25 | AX2 |
| HSPC300-GFP | 80 | 24 | 5 | 20.1 | AX2 |
| MyoB-mNeon | 423 | 24 | 23 | 95.8 | AX2 |
| H2B-mCherry | 3 | 3 | 1 | 33.3 | AX2 |
