# Supplementary material for: Rapid and efficient genetic engineering of both wild type and axenic strains of Dictyostelium discoideum
Source: PLoS One. 2018 May 30;13(5):e0196809. doi: 10.1371/journal.pone.0196809 (PMC5976153; doi:10.1371/journal.pone.0196809)
Supplement: S1 File — (PDF) [file pone.0196809.s017.pdf]

Sequences

Extrachromosomal vectors

pDM1203 – no tag

CTCGAGACTAGAGCTAGATAAAAAAAATTTTTATTTATTTTTATTTATTT
TGAATTAAATAGATTACAAATTAATTAATCCCATCAAATCTTTAAAAAAA
AATGGTTTAAAAAAACTTGGGTTGGTTAATTATTATTTGAAAATTTTAAA
ACCCAAATTAAAAAAAAAAAAAATGGGATTCAAAAATTTTTTTTTTTTTTT
TTTTTTTTTTTTTTTTTTTTTTTCAGATTGCATAAAAAGATTTTTTTTT
TTTTTTTTCTTATTTCTTAAAACAAATAAATTAAATTAAATAAAAAATAA
AAATCAGATCTAGTACTAGTTAAATAAATAAATTATTTAATAAATAATAA
AAAAACAAATTGTTGTAATAATCTAATATTTTCTTTTTTTTTTAATTTTT
TTTTTTTAAATCTTAATAATTATTAAGTTATTTTAATTTTTTTTTTTTTT
TTTTTTTTTTTTTTTTTTTTTTTCTATCAAAAAAATCAAATATATTTAA
AAAATTTATTATTTACAGATACATTTTGAATGGTGAAGATAAATATATGC
ATTAGATGTAAAACAGCCAAAGAGTATGAAAATCAAAAAGATAAAGCTTA
TCGATTTCGAAAAAGTAAATAGCAATTATTACAAAATTCAATCCGAATCT
ACCCAAATAAATTCCAATGAAATTGCCGATTTAAAAAAGTTTATTAAAGA
AGAAGTCAATAAAACTTCTTCCAAAATTGATTTCTTTTTAGTTTCTTCAA
CAGATGCCCTTTCAAATCCAGAAAATTATTCTCTCTTAGAAGTAAAGTGT
ATTAATTGTCATTCTTTGTGTCAAGGAAAAAATTTATATATTTCATGTAC
AAGAGATGGATGTCAAAACAATATTTGCTATAATTGTTTAGGAATAAACA
TAAACATATATAATGTTGTTATTAATTCTAAACTTTGCCCTCCATGTTTC
AATGATTCGGTAATCAACAAGAAGTGTGCCATGTGTAGTAAGAACGGAAC
TAAATGTAATTTGAACCAAGAATGTAAACTTCATCTTTGTGCACAGTGTT
CTAAAAAGTGTCTATACATTCTGAGAGTCAAAACTAATTAAATAAAATAT
AAACTTAATTTCTAAATAAACTCATTTAAAAATATTTAAATAATATGAAT
TTATAACTGTAATTATTGTATTAAAAAATTATATAATTATTTAATGTTAA
AAATGTATTAAAATAATTATAAAAAAATATAACAAAAATTTTCGTAAAAA
TAATTTGTAAAAAAGCTATTAAAAATATTATGAAAAAAAAATTAAAAAAA
TTATTAAATTGTTTTTGTAATTAAGCTATTAAAATAATTATAAAAAAAAA
ATTTTTAAAATTTTAAAAATATTTTTTGTAAAAAAGTATTAAAATAATTA
TGAAAAAAAAATTTTCTAAAAAATTAAAAAAAAAATTAAAATATATTTTA
TGTTAAAAACGTATTAAAATAACTATTAAAAAAATTATATTTAAAAAAGT
ATTAACTTTTTTTAGGTGTGGTTGTGGGGTGGGGTTTAATATATTATAA
TAAAAAATTATTTTTTGTTCATTTATTATTTTCATTGTATATAATGTACT
CAACAACGTTATTATTTTTTCTTTTTTTTTTTATTGTATCAAAATCTTCT
GTTCTTCAAAATGATCAGATTGAAGTAAAATATTTTCAACTTCTTATTGT
TATGTATCAAAAAGAAAACTGTGTTGAAAAGTCAATGACAGGCGCCGTAA
TTTATGATGAATGTAATATTCATGGAAGAGTTGAAACAAATAGTACTCAT
GCGCTTTTTTATGATGACATTGAAACAAATAATTCAAGATGTAACAATTT
TCGTAATTTAACAAACTTAATTAAACTTAATGAATGTATTAATGACGAGT
TTGGAGAGTCTATTCTTTATAAAGAATATAATGAAACTGATGATGGTTAT
TTGTTTAGAGTGGAAGACAGCTTTGTTGAAATTACTTCTCTTTCAATGGA
TTGTACAAAAAATAGTAAAACAATTATTGAAAAATTCAACATTTGTTCAA
AATTTGAAAATGTATATCATATTACAAACATTACACAAGAGAAATCCAAT
AGATTTACATGTACAGATCCATTGTGCCACTATTGTAAGAATGAAAACAT
TCAAAACAATCTTGATTTTAAAACAACAAAGTGTACTCCAAAGTATGGTG

CATCTGATTCTGAATTTTTATCAACAATTTACAATCCAAAGCTCGATGGC
TCAAATAACGGTATGGAAAAGTCAGTAACTCAAGAAAAAAACATTTCAAA
TAATTTAAAAATTAATATATATTTAATTTTCTTTTTAATTATTTTTTTAA
TTAAATAAAGTTTTATTATTTTTTAAGAGTAATTATTGCTCTTTTTTCAT
TTGAAACACCAGAAGCTAAACGTAATTGTTGTTGACTGAAATTTTTTATT
TTTTTTGGGGTAATAGGATTTCCTTTTTTATGAAGATTAATATCTTTGAC
TCGTGAAACATTCTTTTTAACTTTTGTTTTTTCTGTTGGTTTATCATTTG
TTTTTTCACTAATTTCAATACCATCTTGACGTTCATTCATAACTTCATCT
TTTTTTTTTCCTGTTTCTGTATCTTCTTCTATTTTTTTTTCTTTATCTTT
TTCTTTATCTTCTTCTTGTTCTTCCTCTTCTTTTTCTTCTTCTGATACTG
CAGGTGTTTCTTCTTCTTCTTCTTCCGATATTGTCGGTTTTTCTACTTCT
TCTTCTTGTTCTTCTTCTTCTTCTTCTTCTTCTTCTTCTTCTTCTTCTTC
TTCTTCTTCTTCCGGTAATTTATTAATTATATTTCTTTTTTTATATGAAT
TACGTTTGGTTTGTGCAGTAATTTCCTTACATAGAGTGCAGCTTTCAAGA
AAAATTTCAATTTCTTCGTTTGTTGCATAATAACCACTGTCTTTGATATG
ATTAAACATTTTTGATTTTCTTAAATGCTTTCCTTCTTTAATATGAAAAT
TATCGAATTCTAATTCATTAAGAACAATAAGCTCCCCTAATTTAAAAAAT
TAGTTAAAATAAATTAAAATGAACATGTATAAAGATGGATTTTACCATTT
TTTGAAATTCTAAATAACTTTTCTTCATCTCCAATCTTTTTGACTGAAAA
ACGATTTTTAATTGAAGTTATTGTTCTGTGAGTGTTTTGAATCGCCCATT
TCTCTAAATCAGTTTGAGATAGTGTTTTATAATCTGAATTGTTATACACA
ACTTTTGCTCTATTAACCAAATATTTAAAGATTTCATCATCAACTGAATA
TTTTGACTTTACGATTCTTGTCCAAAAAACAATTTCTACTACTATCATTT
TTTATTTATAAAATAATTTAAATACAAAAATGAATTTTTTTTTTTTTAAA
AAAAAAAAAATTTGAAAAAAAAAAAAAAAAAAATTTTAAAAAAAAAAAAAA
AAAAAAAAAAAAAAAAAAAAATCAAATAAAAAGTAAAAAATAAAAACCGAA
AAACATTCATTGTAATTTCAAATGTCGAGGCCGGCAGAGGCGGTTTGCGT
ATTGGGCGCTCTTCCGCTTCCTCGCTCACTGACTCGCTGCGCTCGGTCGT
TCGGCTGCGGCGAGCGGTATCAGCTCACTCAAAGGCGGTAATACGGTTAT
CCACAGAATCAGGGGATAACGCAGGAAAGAACATGTGAGCAAAAGGCCAG
CAAAAGGCCAGGAACCGTAAAAAGGCCGCGTTGCTGGCGTTTTTCCATAG
GCTCCGCCCCCCTGACGAGCATCACAAAAATCGACGCTCAAGTCAGAGGT
GGCGAAACCCGACAGGACTATAAAGATACCAGGCGTTTCCCCCTGGAAGC
TCCCTCGTGCGCTCTCCTGTTCCGACCCTGCCGCTTACCGGATACCTGTC
CGCCTTTCTCCCTTCGGGAAGCGTGGCGCTTTCTCATAGCTCACGCTGTA
GGTATCTCAGTTCGGTGTAGGTCGTTCGCTCCAAGCTGGGCTGTGTGCAC
GAACCCCCCGTTCAGCCCGACCGCTGCGCCTTATCCGGTAACTATCGTCT
TGAGTCCAACCCGGTAAGACACGACTTATCGCCACTGGCAGCAGCCACTG
GTAACAGGATTAGCAGAGCGAGGTATGTAGGCGGTGCTACAGAGTTCTTG
AAGTGGTGGCCTAACTACGGCTACACTAGAAGGACAGTATTTGGTATCTG
CGCTCTGCTGAAGCCAGTTACCTTCGGAAAAAGAGTTGGTAGCTCTTGAT
CCGGCAAACAAACCACCGCTGGTAGCGGTGGTTTTTTTGTTTGCAAGCAG
CAGATTACGCGCAGAAAAAAAGGATCTCAAGAAGATCCTTTGATCTTTTC
TACGGGGTCTGACGCTCAGTGGAACGAAAACTCACGTTAAGGGATTTTGG
TCATGAGATTATCAAAAAGGATCTTCACCTAGATCCTTTTAAATTAAAAA
TGAAGTTTTAAATCAATCTAAAGTATATATGAGTAAACTTGGTCTGACAG
TTACCAATGCTTAATCAGTGAGGCACCTATCTCAGCGATCTGTCTATTTC
GTTCATCCATAGTTGCCTGACTCCCCGTCGTGTAGATAACTACGATACGG
GAGGGCTTACCATCTGGCCCCAGTGCTGCAATGATACCGCGAGACCCACG
CTCACCGGCTCCAGATTTATCAGCAATAAACCAGCCAGCCGGAAGGGCCG

AGCGCAGAAGTGGTCCTGCAACTTTATCCGCCTCCATCCAGTCTATTAAT
TGTTGCCGGGAAGCTAGAGTAAGTAGTTCGCCAGTTAATAGTTTGCGCAA
CGTTGTTGCCATTGCTACAGGCATCGTGGTGTCACGCTCGTCGTTTGGTA
TGGCTTCATTCAGCTCCGGTTCCCAACGATCAAGGCGAGTTACATGATCC
CCCATGTTGTGCAAAAAAGCGGTTAGCTCCTTCGGTCCTCCGATCGTTGT
CAGAAGTAAGTTGGCCGCAGTGTTATCACTCATGGTTATGGCAGCACTGC
ATAATTCTCTTACTGTCATGCCATCCGTAAGATGCTTTTCTGTGACTGGT
GAGTACTCAACCAAGTCATTCTGAGAATAGTGTATGCGGCGACCGAGTTG
CTCTTGCCCGGCGTCAATACGGGATAATACCGCGCCACATAGCAGAACTT
TAAAAGTGCTCATCATTGGAAAACGTTCTTCGGGGCGAAAACTCTCAAGG
ATCTTACCGCTGTTGAGATCCAGTTCGATGTAACCCACTCGTGCACCCAA
CTGATCTTCAGCATCTTTTACTTTCACCAGCGTTTCTGGGTGAGCAAAAA
CAGGAAGGCAAAATGCCGCAAAAAAGGGAATAAGGGCGACACGGAAATGT
TGAATACTCATACTCTTCCTTTTTCAATATTATTGAAGCATTTATCAGGG
TTATTGTCTCATGAGCGGATACATATTTGAATGTATTTAGAAAAATAAAC
AAATAGGGGTTCCGCGCACATTTCCCCGAAAAGTGCCACCTGACGCGCCC
TGTAGCGGGATCCATTTTATTTAATATACTAAATAATAAAAAAGTTAAAA
AATGATCATTGGATAAATTTTTTATAATTATAAATAAAGATAATAATTTT
TTTTTTAACAAAACTAAAAATAAAAATAATAAAATAATTGTTAAAATAGG
TTTTTTTTTTTTTTTTTTTTTTTTAATAAATGGTATTTATTAATTTATTT
GTTGTGTGTGTTTTTTTTTTTATAATATTTTTTTTTTTAGCATTGAATTA
AGAAGAAATCAAATTGATGCGGCCGCTCAGAAGAACTCGTCAAGAAGGCG
ATAGAAGGCGATGCGCTGCGAATCGGGAGCGGCGATACCGTAAAGCACGA
GGAAGCGGTCAGCCCATTCGCCGCCAAGCTCTTCAGCAATATCACGGGTA
GCCAACGCTATGTCCTGATAGCGGTCCGCCACACCCAGCCGTCCACAGTC
GATGAATCCAGAAAAGCGGCCATTTTCCACCATGATATTCGGCAAGCAGG
CATCGCCATGGGTCACGACGAGATCCTCGCCGTCGGGCATGCGCGCCTTG
AGCCTGGCGAACAGTTCGGCTGGCGCGAGCCCCTGATGCTCTTCGTCCAG
ATCATCCTGATCGACAAGACCGGCTTCCATCCGAGTACGTGCTCGCTCGA
TGCGATGTTTCGCTTGGTGGTCGAATGGGCAGGTAGCCGGATCAAGCGTA
TGCAGCCGCCGCATTGCATCAGCCATGATGGATACTTTCTCGGCAGGAGC
AAGGTGAGATGACAGGAGATCCTGCCCCGGCACTTCGCCCAATAGCAGCC
AGTCCCTTCCCGCTTCAGTGACAACGTCGAGCACAGCTGCGCAAGGAACG
CCCGTCGTGGCCAGCCACGATAGCCGCGCTGCCTCGTCCTGCAGTTCATT
CAGGGCACCGGACAGGTCGGTCTTGACAAAAAGAACCGGGCGCCCCTGCG
CTGACAGCCGGAACACGGCGGCATCAGAGCAGCCGATTGTCTGTTGTGCC
CAGTCATAGCCGAATAGCCTCTCCACCCAAGCGGCCGGAGAACCTGCGTG
CAATCCATCTTGTTCAATCATGCGAAACGATCCAGCTTGAACATCTTCAC
CATCCATTTTTTGCTAGCTGTGAAATTAGTTTAAAATACAAATAAAGAGT
TATAATAATATACAGTTGAATAAAAAAAAAAAAAAATGAATTGGAAAATTT
ATTTTTATATGAAGAAAAAAAAAATTTTGAAAAAAAAAAAAAAATTAAAAA
AAAAAAAAAAAAAAAAAAAATTTAAAATTATTCCACTGTGGGGGGCCCCA
AATTTTATTTAAAAAAAAAAAAAAAAATGGGTCCCTTTTGGGGGGTTGGAA
AAAAAAAAAAAAAAAAAAAAAAAAATTGAAAATATAATGTTAGTCATATG
ATTAATCATT

pDM1207 – GFP (N)

CTCGAGACTAGAGCTAGATAAAAAAAATTTTTATTTATTTTTATTTATTT
TGAATTAAATAGATTACAATTAATTAATCCCATCAAATCTTTAAAAAAA

AATGGTTTAAAAAAACTTGGGTTGGTTAATTATTATTTGAAAATTTTAAA
ACCCAAATTAAAAAAAAAAAAATGGGATTCAAAAATTTTTTTTTTTTTT
TTTTTTTTTTTTTTTTTTTTTTCAGATTGCATAAAAAGATTTTTTTTT
TTTTTTTTCTTATTTCTTAAAACAAATAAATTAAATTAAATAAAAAATAA
AAATCAGATCCAAAAAATGTCAAAAGGTGAAGAATTATTTACAGGTGTTG
TTCCAATTTTAGTTGAATTAGATGGTGATGTTAATGGTCATAAATTTTCA
GTTTCAGGTGAAGGTGAAGGTGATGCAACATATGGTAAATTAACATTAAA
ATTTATTTGTACAACAGGTAAATTACCAGTTCCATGGCCAACATTAGTTA
CAACATTTACATATGGTGTTCAATGTTTTTCAAGATATCCAGATCATATG
AAACAACATGATTTTTTTAAATCAGCAATGCCAGAAGGTTATGTTCAAGA
AAGAACAATTTTTTTTAAAGATGATGGTAATTATAAAACAAGAGCAGAAG
TTAAATTTGAAGGTGATACATTAGTTAATAGAATTGAATTAAAAGGTATT
GATTTTAAAGAAGATGGTAATATTTTAGGTCATAAATTAGAATATAATTA
TAATTCACATAATGTTTATATTATGGCAGATAAACAAAAAAATGGTATTA
AAGTTAATTTTAAAATTAGACATAATATTGAAGATGGTTCAGTTCAATTA
GCAGATCATTATCAACAAAATACACCAATTGGTGATGGTCCAGTTTTATT
ACCAGATAATCATTATTTATCAACACAATCAGCATTATCAAAAGATCCAA
ATGAAAAAAGAGATCATATGGTTTTATTAGAATTTGTTACAGCAGCAGGT
ATTACACATGGTATGGATGAATTATATAAAGGTGGTTCAGGAGGTAGTAG
ATCTTCAACTAGTTAAATAAATAAATTATTTAATAAATAATAAAAAAACA
AATTGTTGTAATAATCTAATATTTTCTTTTTTTTTTAATTTTTTTTTTTT
AAATCTTAATAATTATTAAGTTATTTTAATTTTTTTTTTTTTTTTTTTTT
TTTTTTTTTTTTTTTTTCTATCAAAAAAATCAAATATATTTAAAAAATTT
ATTATTTACAGATACATTTTGAATGGTGAAGATAAATATATGCATTAGAT
GTAAAACAGCCAAAGAGTATGAAAATCAAAAAGATAAAGCTTATCGATTT
CGAAAAAGTAAATAGCAATTATTACAAAATTCAATCCGAATCTACCCAAA
TAAATTCCAATGAAATTGCCGATTTAAAAAAGTTTATTAAAGAAGAAGTC
AATAAAACTTCTTCCAAAATTGATTTCTTTTTAGTTTCTTCAACAGATGC
CCTTTCAAATCCAGAAAATTATTCTCTCTTAGAAGTAAAGTGTATTAATT
GTCATTCTTTGTGTCAAGGAAAAAATTTATATATTTCATGTACAAGAGAT
GGATGTCAAAACAATATTTGCTATAATTGTTTAGGAATAAACATAAACAT
ATATAATGTTGTTATTAATTCTAAACTTTGCCCTCCATGTTTCAATGATT
CGGTAATCAACAAGAAGTGTGCCATGTGTAGTAAGAACGGAACTAAATGT
AATTTGAACCAAGAATGTAAACTTCATCTTTGTGCACAGTGTTCTAAAAA
GTGTCTATACATTCTGAGAGTCAAAACTAATTAAATAAAATATAAACTTA
ATTTCTAAATAAACTCATTTAAAAATATTTAAATAATATGAATTTATAAC
TGTAATTATTGTATTAAAAAATTATATAATTATTTAATGTTAAAAATGTA
TTAAAATAATTATAAAAAAATATAACAAAAATTTTCGTAAAAATAATTTG
TAAAAAAGCTATTAAAAATATTATGAAAAAAAAATTAAAAAAATTATTAA
ATTGTTTTTGTAATTAAGCTATTAAAATAATTATAAAAAAAAAATTTTTA
AAATTTTAAAAATATTTTTTGTAAAAAAGTATTAAAATAATTATGAAAAA
AAAATTTTCTAAAAAATTAAAAAAAAAATTAAAATATATTTTATGTTAAA
AACGTATTAAAATAACTATTAAAAAAATTATATTTAAAAAAGTATTAACT
TTTTTTTAGGTGTGGTTGTGGGGTGGGGTTTAATATATTATAATAAAAAA
TTATTTTTTGTTCATTTATTATTTTCATTGTATATAATGTACTCAACAAC
GTTATTATTTTTTCTTTTTTTTTTTATTGTATCAAAATCTTCTGTTCTTC
AAAATGATCAGATTGAAGTAAAATATTTTCAACTTCTTATTGTTATGTAT
CAAAAAGAAAACTGTGTTGAAAAGTCAATGACAGGCGCCGTAATTTATGA
TGAATGTAATATTCATGGAAGAGTTGAAACAAATAGTACTCATGCGCTTT
TTTATGATGACATTGAAACAAATAATTCAAGATGTAACAATTTTCGTAAT

TTAACAAACTTAATTAAACTTAATGAATGTATTAATGACGAGTTTGGAGA
GTCTATTCTTTATAAAGAATATAATGAAACTGATGATGGTTATTTGTTTA
GAGTGGAAGACAGCTTTGTTGAAATTACTTCTCTTTCAATGGATTGTACA
AAAAATAGTAAAACAATTATTGAAAAATTCAACATTTGTTCAAAATTTGA
AAATGTATATCATATTACAAACATTACACAAGAGAAATCCAATAGATTTA
CATGTACAGATCCATTGTGCCACTATTGTAAGAATGAAAACATTCAAAAC
AATCTTGATTTTAAAACAACAAAGTGTACTCCAAAGTATGGTGCATCTGA
TTCTGAATTTTTATCAACAATTTACAATCCAAAGCTCGATGGCTCAAATA
ACGGTATGGAAAAGTCAGTAACTCAAGAAAAAAACATTTCAAATAATTTA
AAAATTAATATATATTTAATTTTCTTTTTAATTATTTTTTTAATTAAATA
AAGTTTTATTATTTTTTAAGAGTAATTATTGCTCTTTTTTCATTTGAAAC
ACCAGAAGCTAAACGTAATTGTTGTTGACTGAAATTTTTTATTTTTTTTG
GGGTAATAGGATTTCCTTTTTTATGAAGATTAATATCTTTGACTCGTGAA
ACATTCTTTTTAACTTTTGTTTTTTCTGTTGGTTTATCATTTGTTTTTTC
ACTAATTTCAATACCATCTTGACGTTCATTCATAACTTCATCTTTTTTTT
TTCCTGTTTCTGTATCTTCTTCTATTTTTTTTTTCTTTATCTTTTTCTTTA
TCTTCTTCTTGTTCTTCCTCTTCTTTTTCTTCTTCTGATACTGCAGGTGT
TTCTTCTTCTTCTTCTTCCGATATTGTCGGTTTTTCTACTTCTTCTTCTT
GTTCTTCTTCTTCTTCTTCTTCTTCTTCTTCTTCTTCTTCTTCTTCTTCT
TCTTCCGGTAATTTATTAATTATATTTCTTTTTTTATATGAATTACGTTT
GGTTTGTGCAGTAATTTCCTTACATAGAGTGCAGCTTTCAAGAAAAATTT
CAATTTCTTCGTTTGTTGCATAATAACCACTGTCTTTGATATGATTAAAC
ATTTTTGATTTTCTTAAATGCTTTCCTTCTTTAATATGAAAATTATCGAA
TTCTAATTCATTAAGAACAATAAGCTCCCCTAATTTAAAAAATTAGTTAA
AATAAATTAAAATGAACATGTATAAAGATGGATTTTACCATTTTTTGAAA
TTCTAAATAACTTTTCTTCATCTCCAATCTTTTTGACTGAAAAACGATTT
TTAATTGAAGTTATTGTTCTGTGAGTGTTTTGAATCGCCCATTTCTCTAA
ATCAGTTTGAGATAGTGTTTTATAATCTGAATTGTTATACACAACTTTTG
CTCTATTAACCAAATATTTAAAGATTTCATCATCAACTGAATATTTTGAC
TTTACGATTCTTGTCCAAAAAACAATTTCTACTACTATCATTTTTTATTT
ATAAAATAATTTAAATACAAAAATGAATTTTTTTTTTTTTAAAAAAAAAA
AAATTTGAAAAAAAAAAAAAAAAAAATTTTAAAAAAAAAAAAAAAAAAAAA
AAAAAAAAAAAAAATCAAATAAAAAGTAAAAAATAAAAACCGAAAAACATT
CATTGTAATTTCAAATGTCGAGGCCGGCAGAGGCGGTTTGCGTATTGGGC
GCTCTTCCGCTTCCTCGCTCACTGACTCGCTGCGCTCGGTCGTTCGGCTG
CGGCGAGCGGTATCAGCTCACTCAAAGGCGGTAATACGGTTATCCACAGA
ATCAGGGGATAACGCAGGAAAGAACATGTGAGCAAAAGGCCAGCAAAAGG
CCAGGAACCGTAAAAAGGCCGCGTTGCTGGCGTTTTTCCATAGGCTCCGC
CCCCCTGACGAGCATCACAAAAATCGACGCTCAAGTCAGAGGTGGCGAAA
CCCGACAGGACTATAAAGATACCAGGCGTTTCCCCCTGGAAGCTCCCTCG
TGCGCTCTCCTGTTCCGACCCTGCCGCTTACCGGATACCTGTCCGCCTTT
CTCCCTTCGGGAAGCGTGGCGCTTTCTCATAGCTCACGCTGTAGGTATCT
CAGTTCGGTGTAGGTCGTTCGCTCCAAGCTGGGCTGTGTGCACGAACCCC
CCGTTCAGCCCGACCGCTGCGCCTTATCCGGTAACTATCGTCTTGAGTCC
AACCCGGTAAGACACGACTTATCGCCACTGGCAGCAGCCACTGGTAACAG
GATTAGCAGAGCGAGGTATGTAGGCGGTGCTACAGAGTTCTTGAAGTGGT
GGCCTAACTACGGCTACACTAGAAGGACAGTATTTGGTATCTGCGCTCTG
CTGAAGCCAGTTACCTTCGGAAAAAGAGTTGGTAGCTCTTGATCCGGCAA
ACAAACCACCGCTGGTAGCGGTGGTTTTTTTGTTTGCAAGCAGCAGATTA
CGCGCAGAAAAAAAGGATCTCAAGAAGATCCTTTGATCTTTTCTACGGGG

TCTGACGCTCAGTGGAACGAAAACTCACGTTAAGGGATTTTGGTCATGAG
ATTATCAAAAAGGATCTTCACCTAGATCCTTTTAAATTAAAAATGAAGTT
TTAAATCAATCTAAAGTATATATGAGTAAACTTGGTCTGACAGTTACCAA
TGCTTAATCAGTGAGGCACCTATCTCAGCGATCTGTCTATTTCGTTCATC
CATAGTTGCCTGACTCCCCGTCGTGTAGATAACTACGATACGGGAGGGCT
TACCATCTGGCCCCAGTGCTGCAATGATACCGCGAGACCCACGCTCACCG
GCTCCAGATTTATCAGCAATAAACCAGCCAGCCGGAAGGGCCGAGCGCAG
AAGTGGTCCTGCAACTTTATCCGCCTCCATCCAGTCTATTAATTGTTGCC
GGGAAGCTAGAGTAAGTAGTTCGCCAGTTAATAGTTTGCGCAACGTTGTT
GCCATTGCTACAGGCATCGTGGTGTCACGCTCGTCGTTTGGTATGGCTTC
ATTCAGCTCCGGTTCCCAACGATCAAGGCGAGTTACATGATCCCCCATGT
TGTGCAAAAAAGCGGTTAGCTCCTTCGGTCCTCCGATCGTTGTCAGAAGT
AAGTTGGCCGCAGTGTTATCACTCATGGTTATGGCAGCACTGCATAATTC
TCTTACTGTCATGCCATCCGTAAGATGCTTTTCTGTGACTGGTGAGTACT
CAACCAAGTCATTCTGAGAATAGTGTATGCGGCGACCGAGTTGCTCTTGC
CCGGCGTCAATACGGGATAATACCGCGCCACATAGCAGAACTTTAAAAGT
GCTCATCATTGGAAAACGTTCTTCGGGGCGAAAACTCTCAAGGATCTTAC
CGCTGTTGAGATCCAGTTCGATGTAACCCACTCGTGCACCCAACTGATCT
TCAGCATCTTTTACTTTCACCAGCGTTTCTGGGTGAGCAAAAACAGGAAG
GCAAAATGCCGCAAAAAAGGGAATAAGGGCGACACGGAAATGTTGAATAC
TCATACTCTTCCTTTTTCAATATTATTGAAGCATTTATCAGGGTTATTGT
CTCATGAGCGGATACATATTTGAATGTATTTAGAAAAATAAACAAATAGG
GGTTCCGCGCACATTTCCCCGAAAAGTGCCACCTGACGCGCCCTGTAGCG
GGATCCATTTTATTTAATATACTAAATAATAAAAAAGTTAAAAAATGATC
ATTGGATAAATTTTTTATAATTATAAATAAAGATAATAATTTTTTTTTTA
ACAAAACTAAAAATAAAAATAATAAAATAATTGTTAAAATAGGTTTTTTT
TTTTTTTTTTTTTTTTTAATAAATGGTATTTATTAATTTATTTGTTGTGT
GTGTTTTTTTTTTTATAATATTTTTTTTTTTAGCATTGAATTAAGAAGAA
ATCAAATTGATGCGGCCGCTCAGAAGAACTCGTCAAGAAGGCGATAGAAG
GCGATGCGCTGCGAATCGGGAGCGGCGATACCGTAAAGCACGAGGAAGCG
GTCAGCCCATTCGCCGCCAAGCTCTTCAGCAATATCACGGGTAGCCAACG
CTATGTCCTGATAGCGGTCCGCCACACCCAGCCGTCCACAGTCGATGAAT
CCAGAAAAGCGGCCATTTTCCACCATGATATTCGGCAAGCAGGCATCGCC
ATGGGTCACGACGAGATCCTCGCCGTCGGGCATGCGCGCCTTGAGCCTGG
CGAACAGTTCGGCTGGCGCGAGCCCCTGATGCTCTTCGTCCAGATCATCC
TGATCGACAAGACCGGCTTCCATCCGAGTACGTGCTCGCTCGATGCGATG
TTTCGCTTGGTGGTCGAATGGGCAGGTAGCCGGATCAAGCGTATGCAGCC
GCCGCATTGCATCAGCCATGATGGATACTTTCTCGGCAGGAGCAAGGTGA
GATGACAGGAGATCCTGCCCCGGCACTTCGCCCAATAGCAGCCAGTCCCT
TCCCGCTTCAGTGACAACGTCGAGCACAGCTGCGCAAGGAACGCCCGTCG
TGGCCAGCCACGATAGCCGCGCTGCCTCGTCCTGCAGTTCATTCAGGGCA
CCGGACAGGTCGGTCTTGACAAAAAGAACCGGGCGCCCCTGCGCTGACAG
CCGGAACACGGCGGCATCAGAGCAGCCGATTGTCTGTTGTGCCCAGTCAT
AGCCGAATAGCCTCTCCACCCAAGCGGCCGGAGAACCTGCGTGCAATCCA
TCTTGTTCAATCATGCGAAACGATCCAGCTTGAACATCTTCACCATCCAT
TTTTTGCTAGCTGTGAAATTAGTTTAAAATACAAATAAAGAGTTATAATA
ATATACAGTTGAATAAAAAAAAAAAAAAATGAATTGGAAAATTTATTTTTA
TATGAAGAAAAAAAAATTTTGAAAAAAAAAAAAAAAATTAAAAAAAAAAAA
AAAAAAAAAAAAATTTAAAATTATTCCACTGTGGGGGGCCCCAAATTTTA
TTTAAAAAAAAAAAAAAAAATGGGTCCCTTTTGGGGGGTTGGAAAAAAAAA

AAAAAAAAAAAAAAAAAAATTGAAAATATAATGTTAGTCATATGATTAATC
ATT

pDM1208 – mCherry (N)

CTCGAGACTAGAGCTAGATAAAAAAAATTTTTATTTATTTTTATTTATTT
TGAATTAAATAGATTACAAATTAATTAATCCCATCAAATCTTTAAAAAAA
AATGGTTTAAAAAAACTTGGGTTGGTTAATTATTATTTGAAAATTTTAAA
ACCCAAATTAAAAAAAAAAAAATGGGATTCAAAAATTTTTTTTTTTTTTT
TTTTTTTTTTTTTTTTTTTTTTTCAGATTGCATAAAAAGATTTTTTTTTT
TTTTTTTTCTTATTTCTTAAAACAAATAAATTAAATTAAATAAAAAATAA
AAATCAGATCCAAAAAATGGTTTCAAAAGGTGAAGAAGATAATATGGCAA
TTATTAAAGAATTTATGAGATTTAAAGTTCATATGGAAGGTTCAGTTAAT
GGTCATGAATTTGAAATTGAAGGTGAAGGTGAAGGTAGACCATATGAAGG
TACACAAACAGCAAAATTAAAAGTTACAAAAGGTGGTCCATTACCATTTG
CATGGGATATTTTATCACCACAATTTATGTATGGTTCAAAAGCATATGTT
AAACATCCAGCAGATATTCCAGATTATTTAAAATTATCATTTCCAGAAGG
TTTTAAATGGGAAAGAGTTATGAATTTTGAAGATGGTGGTGTTGTTACAG
TTACACAAGATTCATCATTACAAGATGGTGAATTTATTTATAAAGTTAAA
TTAAGAGGTACAAATTTTCCATCAGATGGTCCAGTTATGCAAAAAAAAAC
AATGGGTTGGGAAGCATCATCAGAAAGAATGTATCCAGAAGATGGTGCAT
TAAAAGGTGAAATTAAACAAAGATTAAAATTAAAAGATGGTGGTCATTAT
GATGCAGAAGTTAAAACAACATATAAAGCAAAAAAACCAGTTCAATTACC
AGGTGCATATAATGTTAATATTAAATTAGATATTACATCACATAATGAAG
ATTATACAATTGTTGAACAATATGAAAGAGCAGAAGGTAGACATTCAACA
GGTGGTATGGATGAATTATATAAAGGTGGTTCAGGAGGTAGTAGATCTTC
AACTAGTTAAATAAATAAATTATTTAATAAATAATAAAAAAACAAATTGT
TGTAATAATCTAATATTTTCTTTTTTTTTTAATTTTTTTTTTTTAAATCT
TAATAATTATTAAGTTATTTTAATTTTTTTTTTTTTTTTTTTTTTTTTTT
TTTTTTTTTTTCTATCAAAAAAATCAAATATATTTAAAAAATTTATTATT
TACAGATACATTTTGAATGGTGAAGATAAATATATGCATTAGATGTAAAA
CAGCCAAAGAGTATGAAAATCAAAAAGATAAAGCTTATCGATTTCGAAAA
AGTAAATAGCAATTATTACAAAATTCAATCCGAATCTACCCAAATAAATT
CCAATGAAATTGCCGATTTAAAAAAGTTTATTAAAGAAGAAGTCAATAAA
ACTTCTTCCAAAATTGATTTCTTTTTAGTTTCTTCAACAGATGCCCTTTC
AAATCCAGAAAATTATTCTCTCTTAGAAGTAAAGTGTATTAATTGTCATT
CTTTGTGTCAAGGAAAAAATTTATATATTTCATGTACAAGAGATGGATGT
CAAAACAATATTTGCTATAATTGTTTAGGAATAAACATAAACATATATAA
TGTTGTTATTAATTCTAAACTTTGCCCTCCATGTTTCAATGATTCGGTAA
TCAACAAGAAGTGTGCCATGTGTAGTAAGAACGGAACTAAATGTAATTTG
AACCAAGAATGTAAACTTCATCTTTGTGCACAGTGTTCTAAAAAGTGTCT
ATACATTCTGAGAGTCAAAACTAATTAAATAAAATATAAACTTAATTTCT
AAATAAACTCATTTAAAAATATTTAAATAATATGAATTTATAACTGTAAT
TATTGTATTAAAAAATTATATAATTATTTAATGTTAAAAATGTATTAAAA
TAATTATAAAAAAATATAACAAAAATTTTCGTAAAAATAATTTGTAAAAA
AGCTATTAAAAATATTATGAAAAAAAAATTAAAAAAATTATTAAATTGTT
TTTGTAATTAAGCTATTAAAATAATTATAAAAAAAAAATTTTTAAAATTT
TAAAAATATTTTTTGTAAAAAAGTATTAAAATAATTATGAAAAAAAAATT
TTCTAAAAAATTAAAAAAAAAATTAAAATATATTTTATGTTAAAAACGTA
TTAAAATAACTATTAAAAAAATTATATTTAAAAAAGTATTAACTTTTTTT

TAGGTGTGGTTGTGGGGTGGGGTTTAATATATTATAATAAAAAATTATTT
TTTGTTCATTTATTATTTTCATTGTATATAATGTACTCAACAACGTTATT
ATTTTTTCTTTTTTTTTTTATTGTATCAAAATCTTCTGTTCTTCAAAATG
ATCAGATTGAAGTAAAATATTTTCAACTTCTTATTGTTATGTATCAAAAA
GAAAACTGTGTTGAAAAGTCAATGACAGGCGCCGTAATTTATGATGAATG
TAATATTCATGGAAGAGTTGAAACAAATAGTACTCATGCGCTTTTTTATG
ATGACATTGAAACAAATAATTCAAGATGTAACAATTTTCGTAATTTAACA
AACTTAATTAAACTTAATGAATGTATTAATGACGAGTTTGGAGAGTCTAT
TCTTTATAAAGAATATAATGAAACTGATGATGGTTATTTGTTTAGAGTGG
AAGACAGCTTTGTTGAAATTACTTCTCTTTCAATGGATTGTACAAAAAAT
AGTAAAACAATTATTGAAAAATTCAACATTTGTTCAAAATTTGAAAATGT
ATATCATATTACAAACATTACACAAGAGAAATCCAATAGATTTACATGTA
CAGATCCATTGTGCCACTATTGTAAGAATGAAAACATTCAAAACAATCTT
GATTTTAAAACAACAAAGTGTACTCCAAAGTATGGTGCATCTGATTCTGA
ATTTTTATCAACAATTTACAATCCAAAGCTCGATGGCTCAAATAACGGTA
TGGAAAAGTCAGTAACTCAAGAAAAAAACATTTCAAATAATTTAAAAATT
AATATATATTTAATTTTCTTTTTAATTATTTTTTTAATTAAATAAAGTTT
TATTATTTTTTAAGAGTAATTATTGCTCTTTTTTCATTTGAAACACCAGA
AGCTAAACGTAATTGTTGTTGACTGAAATTTTTTATTTTTTTTGGGGTAA
TAGGATTTCCTTTTTTTATGAAGATTAATATCTTTGACTCGTGAAACATTC
TTTTTAACTTTTGTTTTTTCTGTTGGTTTATCATTTGTTTTTTCACTAAT
TTCAATACCATCTTGACGTTCATTCATAACTTCATCTTTTTTTTTTCCTG
TTTCTGTATCTTCTTCTATTTTTTTTTTCTTTATCTTTTTCTTTATCTTCT
TCTTGTTCTTCCTCTTCTTTTTCTTCTTCTGATACTGCAGGTGTTTCTTC
TTCTTCTTCTTCCGATATTGTCGGTTTTTCTACTTCTTCTTCTTGTTCTT
CTTCTTCTTCTTCTTCTTCTTCTTCTTCTTCTTCTTCTTCTTCTTCTTCC
GGTAATTTATTAATTATATTTCTTTTTTTATATGAATTACGTTTGGTTTG
TGCAGTAATTTCCTTACATAGAGTGCAGCTTTCAAGAAAAATTTCAATTT
CTTCGTTTGTTGCATAATAACCACTGTCTTTGATATGATTAAACATTTTT
GATTTTCTTAAATGCTTTCCTTCTTTAATATGAAAATTATCGAATTCTAA
TTCATTAAGAACAATAAGCTCCCCTAATTTAAAAAATTAGTTAAAATAAA
TTAAAATGAACATGTATAAAGATGGATTTTACCATTTTTTGAAATTCTAA
ATAACTTTTCTTCATCTCCAATCTTTTTGACTGAAAAACGATTTTTAATT
GAAGTTATTGTTCTGTGAGTGTTTTGAATCGCCCATTTCTCTAAATCAGT
TTGAGATAGTGTTTTATAATCTGAATTGTTATACACAACTTTTGCTCTAT
TAACCAAATATTTAAAGATTTCATCATCAACTGAATATTTTGACTTTACG
ATTCTTGTCCAAAAAACAATTTCTACTACTATCATTTTTTATTTATAAAA
TAATTTAAATACAAAAATGAATTTTTTTTTTTTTAAAAAAAAAAAAATTT
GAAAAAAAAAAAAAAAAAATTTTAAAAAAAAAAAAAAAAAAAAAAAAAAA
AAAAAAATCAAATAAAAAGTAAAAAATAAAAACCGAAAAACATTCATTGT
AATTTCAAATGTCGAGGCCGGCAGAGGCGGTTTGCGTATTGGGCGCTCTT
CCGCTTCCTCGCTCACTGACTCGCTGCGCTCGGTCGTTCGGCTGCGGCGA
GCGGTATCAGCTCACTCAAAGGCGGTAATACGGTTATCCACAGAATCAGG
GGATAACGCAGGAAAGAACATGTGAGCAAAAGGCCAGCAAAAGGCCAGGA
ACCGTAAAAAGGCCGCGTTGCTGGCGTTTTTCCATAGGCTCCGCCCCCCT
GACGAGCATCACAAAAATCGACGCTCAAGTCAGAGGTGGCGAAACCCGAC
AGGACTATAAAGATACCAGGCGTTTCCCCCTGGAAGCTCCCTCGTGCGCT
CTCCTGTTCCGACCCTGCCGCTTACCGGATACCTGTCCGCCTTTCTCCCT
TCGGGAAGCGTGGCGCTTTCTCATAGCTCACGCTGTAGGTATCTCAGTTC
GGTGTAGGTCGTTCGCTCCAAGCTGGGCTGTGTGCACGAACCCCCCGTTC

AGCCCGACCGCTGCGCCTTATCCGGTAACTATCGTCTTGAGTCCAACCCG
GTAAGACACGACTTATCGCCACTGGCAGCAGCCACTGGTAACAGGATTAG
CAGAGCGAGGTATGTAGGCGGTGCTACAGAGTTCTTGAAGTGGTGGCCTA
ACTACGGCTACACTAGAAGGACAGTATTTGGTATCTGCGCTCTGCTGAAG
CCAGTTACCTTCGGAAAAAGAGTTGGTAGCTCTTGATCCGGCAAACAAAC
CACCGCTGGTAGCGGTGGTTTTTTTGTTTGCAAGCAGCAGATTACGCGCA
GAAAAAAAGGATCTCAAGAAGATCCTTTGATCTTTTCTACGGGGTCTGAC
GCTCAGTGGAACGAAAACTCACGTTAAGGGATTTTGGTCATGAGATTATC
AAAAAGGATCTTCACCTAGATCCTTTTAAATTAAAAATGAAGTTTTAAAT
CAATCTAAAGTATATATGAGTAAACTTGGTCTGACAGTTACCAATGCTTA
ATCAGTGAGGCACCTATCTCAGCGATCTGTCTATTTCGTTCATCCATAGT
TGCCTGACTCCCCGTCGTGTAGATAACTACGATACGGGAGGGCTTACCAT
CTGGCCCCAGTGCTGCAATGATACCGCGAGACCCACGCTCACCGGCTCCA
GATTTATCAGCAATAAACCAGCCAGCCGGAAGGGCCGAGCGCAGAAGTGG
TCCTGCAACTTTATCCGCCTCCATCCAGTCTATTAATTGTTGCCGGGAAG
CTAGAGTAAGTAGTTCGCCAGTTAATAGTTTGCGCAACGTTGTTGCCATT
GCTACAGGCATCGTGGTGTCACGCTCGTCGTTTGGTATGGCTTCATTCAG
CTCCGGTTCCCAACGATCAAGGCGAGTTACATGATCCCCCATGTTGTGCA
AAAAAGCGGTTAGCTCCTTCGGTCCTCCGATCGTTGTCAGAAGTAAGTTG
GCCGCAGTGTTATCACTCATGGTTATGGCAGCACTGCATAATTCTCTTAC
TGTCATGCCATCCGTAAGATGCTTTTCTGTGACTGGTGAGTACTCAACCA
AGTCATTCTGAGAATAGTGTATGCGGCGACCGAGTTGCTCTTGCCCGGCG
TCAATACGGGATAATACCGCGCCACATAGCAGAACTTTAAAAGTGCTCAT
CATTGGAAAACGTTCTTCGGGGCGAAAACTCTCAAGGATCTTACCGCTGT
TGAGATCCAGTTCGATGTAACCCACTCGTGCACCCAACTGATCTTCAGCA
TCTTTTACTTTCACCAGCGTTTCTGGGTGAGCAAAAACAGGAAGGCAAAA
TGCCGCAAAAAAGGGAATAAGGGCGACACGGAAATGTTGAATACTCATAC
TCTTCCTTTTTCAATATTATTGAAGCATTTATCAGGGTTATTGTCTCATG
AGCGGATACATATTTGAATGTATTTAGAAAAATAAACAAATAGGGGTTCC
GCGCACATTTCCCCGAAAAGTGCCACCTGACGCGCCCTGTAGCGGGATCC
ATTTTATTTAATATACTAAATAATAAAAAAGTTAAAAAATGATCATTGGA
TAAATTTTTTATAATTATAAATAAAGATAATAATTTTTTTTTTAACAAAA
CTAAAAATAAAAATAATAAAATAATTGTTAAAATAGGTTTTTTTTTTTTT
TTTTTTTTTTTAATAAATGGTATTTATTAATTTATTTGTTGTGTGTGTTT
TTTTTTTTATAATATTTTTTTTTTTAGCATTGAATTAAGAAGAAATCAAA
TTGATGCGGCCGCTCAGAAGAACTCGTCAAGAAGGCGATAGAAGGCGATG
CGCTGCGAATCGGGAGCGGCGATACCGTAAAGCACGAGGAAGCGGTCAGC
CCATTCGCCGCCAAGCTCTTCAGCAATATCACGGGTAGCCAACGCTATGT
CCTGATAGCGGTCCGCCACACCCAGCCGTCCACAGTCGATGAATCCAGAA
AAGCGGCCATTTTCCACCATGATATTCGGCAAGCAGGCATCGCCATGGGT
CACGACGAGATCCTCGCCGTCGGGCATGCGCGCCTTGAGCCTGGCGAACA
GTTCGGCTGGCGCGAGCCCCTGATGCTCTTCGTCCAGATCATCCTGATCG
ACAAGACCGGCTTCCATCCGAGTACGTGCTCGCTCGATGCGATGTTTCGC
TTGGTGGTCGAATGGGCAGGTAGCCGGATCAAGCGTATGCAGCCGCCGCA
TTGCATCAGCCATGATGGATACTTTCTCGGCAGGAGCAAGGTGAGATGAC
AGGAGATCCTGCCCCGGCACTTCGCCCAATAGCAGCCAGTCCCTTCCCGC
TTCAGTGACAACGTCGAGCACAGCTGCGCAAGGAACGCCCGTCGTGGCCA
GCCACGATAGCCGCGCTGCCTCGTCCTGCAGTTCATTCAGGGCACCGGAC
AGGTCGGTCTTGACAAAAAGAACCGGGCGCCCCTGCGCTGACAGCCGGAA
CACGGCGGCATCAGAGCAGCCGATTGTCTGTTGTGCCCAGTCATAGCCGA

ATAGCCTCTCCACCCAAGCGGCCGGAGAACCTGCGTGCAATCCATCTTGT
TCAATCATGCGAAACGATCCAGCTTGAACATCTTCACCATCCATTTTTTG
CTAGCTGTGAAATTAGTTTAAAATACAAATAAAGAGTTATAATAATATAC
AGTTGAATAAAAAAAAAAAAAAATGAATTGGAAAATTTATTTTTATATGAA
GAAAAAAAAATTTTGAAAAAAAAAAAAAAAATTAAAAAAAAAAAAAAAAAA
AAAAAAATTTAAAATTATTCCACTGTGGGGGGCCCCAAATTTTATTTAAA
AAAAAAAAAAAAAATGGGTCCCTTTTGGGGGGTTGGAAAAAAAAAAAAAAA
AAAAAAAAAAAAATTGAAAATATAATGTTAGTCATATGATTAATCATT

pDM1209 – GFP (C)

CTCGAGACTAGAGCTAGATAAAAAAAATTTTTATTTATTTTTATTTATTT
TGAATTAAATAGATTACAAATTAATTAATCCCATCAAATCTTTAAAAAAA
AATGGTTTAAAAAAACTTGGGTTGGTTAATTATTATTTGAAAATTTTAAA
ACCCAAATTAAAAAAAAAAAAATGGGATTCAAAAATTTTTTTTTTTTTTT
TTTTTTTTTTTTTTTTTTTTTTTCAGATTGCATAAAAAGATTTTTTTTT
TTTTTTTTCTTATTTCTTAAAACAAATAAATTAAATTAAATAAAAAATAA
AAATCAGATCTTCAACTAGTGGTGGTTCAGGAGGTAGTTCAAAAGGTGAA
GAATTATTTACAGGTGTTGTTCCAATTTTAGTTGAATTAGATGGTGATGT
TAATGGTCATAAATTTTCAGTTTCAGGTGAAGGTGAAGGTGATGCAACAT
ATGGTAAATTAACATTAAAATTTATTTGTACAACAGGTAAATTACCAGTT
CCATGGCCAACATTAGTTACAACATTTACATATGGTGTTCAATGTTTTTC
AAGATATCCAGATCATATGAAACAACATGATTTTTTTAAATCAGCAATGC
CAGAAGGTTATGTTCAAGAAAGAACAATTTTTTTTAAAGATGATGGTAAT
TATAAAACAAGAGCAGAAGTTAAATTTGAAGGTGATACATTAGTTAATAG
AATTGAATTAAAAGGTATTGATTTTAAAGAAGATGGTAATATTTTAGGTC
ATAAATTAGAATATAATTATAATTCACATAATGTTTATATTATGGCAGAT
AAACAAAAAAATGGTATTAAAGTTAATTTTAAAATTAGACATAATATTGA
AGATGGTTCAGTTCAATTAGCAGATCATTATCAACAAAATACACCAATTG
GTGATGGTCCAGTTTTATTACCAGATAATCATTATTTATCAACACAATCA
GCATTATCAAAAGATCCAAATGAAAAAAGAGATCATATGGTTTTATTAGA
ATTTGTTACAGCAGCAGGTATTACACATGGTATGGATGAATTATATAAAT
AAGCTAGTTAAATAAATAAATTATTTAATAAATAATAAAAAAACAAATTG
TTGTAATAATCTAATATTTTCTTTTTTTTTTAATTTTTTTTTTTTAAATC
TTAATAATTATTAAGTTATTTTAATTTTTTTTTTTTTTTTTTTTTTTTTT
TTTTTTTTTTTTCTATCAAAAAAATCAAATATATTTAAAAAATTTATTAT
TTACAGATACATTTTGAATGGTGAAGATAAATATATGCATTAGATGTAAA
ACAGCCAAAGAGTATGAAAATCAAAAAGATAAAGCTTATCGATTTCGAAA
AAGTAAATAGCAATTATTACAAAATTCAATCCGAATCTACCCAAATAAAT
TCCAATGAAATTGCCGATTTAAAAAAGTTTATTAAAGAAGAAGTCAATAA
AACTTCTTCCAAAATTGATTTCTTTTTAGTTTCTTCAACAGATGCCCTTT
CAAATCCAGAAAATTATTCTCTCTTAGAAGTAAAGTGTATTAATTGTCAT
TCTTTGTGTCAAGGAAAAAATTTATATATTTCATGTACAAGAGATGGATG
TCAAAACAATATTTGCTATAATTGTTTAGGAATAAACATAAACATATATA
ATGTTGTTATTAATTCTAAACTTTGCCCTCCATGTTTCAATGATTCGGTA
ATCAACAAGAAGTGTGCCATGTGTAGTAAGAACGGAACTAAATGTAATTT
GAACCAAGAATGTAAACTTCATCTTTGTGCACAGTGTTCTAAAAAGTGTC
TATACATTCTGAGAGTCAAAACTAATTAAATAAAATATAAACTTAATTTC
TAAATAAACTCATTTAAAAATATTTAAATAATATGAATTTATAACTGTAA
TTATTGTATTAAAAAATTATATAATTATTTAATGTTAAAAATGTATTAAA

ATAATTATAAAAAAATATAACAAAAATTTTCGTAAAAATAATTTGTAAAA
AAGCTATTAAAAATATTATGAAAAAAAAATTAAAAAAATTATTAAATTGT
TTTTGTAATTAAGCTATTAAAATAATTATAAAAAAAAAATTTTTAAAATT
TTAAAAATATTTTTTGTAAAAAAGTATTAAAATAATTATGAAAAAAAAAT
TTTCTAAAAAATTAAAAAAAAAATTAAAATATATTTTATGTTAAAAACGT
ATTAAAATAACTATTAAAAAAATTATATTTAAAAAAGTATTAACTTTTTT
TTAGGTGTGGTTGTGGGGTGGGGTTTAATATATTATAATAAAAAATTATT
TTTTGTTCATTTATTATTTTCATTGTATATAATGTACTCAACAACGTTAT
TATTTTTTCTTTTTTTTTTTATTGTATCAAAATCTTCTGTTCTTCAAAAT
GATCAGATTGAAGTAAAATATTTTCAACTTCTTATTGTTATGTATCAAAA
AGAAAACTGTGTTGAAAAGTCAATGACAGGCGCCGTAATTTATGATGAAT
GTAATATTCATGGAAGAGTTGAAACAAATAGTACTCATGCGCTTTTTTAT
GATGACATTGAAACAAATAATTCAAGATGTAACAATTTTCGTAATTTAAC
AAACTTAATTAAACTTAATGAATGTATTAATGACGAGTTTGGAGAGTCTA
TTCTTTATAAAGAATATAATGAAACTGATGATGGTTATTTGTTTAGAGTG
GAAGACAGCTTTGTTGAAATTACTTCTCTTTCAATGGATTGTACAAAAAA
TAGTAAAACAATTATTGAAAAATTCAACATTTGTTCAAAATTTGAAAATG
TATATCATATTACAAACATTACACAAGAGAAATCCAATAGATTTACATGT
ACAGATCCATTGTGCCACTATTGTAAGAATGAAAACATTCAAAACAATCT
TGATTTTAAAACAACAAAGTGTACTCCAAAGTATGGTGCATCTGATTCTG
AATTTTTATCAACAATTTACAATCCAAAGCTCGATGGCTCAAATAACGGT
ATGGAAAAGTCAGTAACTCAAGAAAAAAACATTTCAAATAATTTAAAAAT
TAATATATATTTAATTTTCTTTTTAATTATTTTTTTAATTAAATAAAGTT
TTATTATTTTTTAAGAGTAATTATTGCTCTTTTTTCATTTGAAACACCAG
AAGCTAAACGTAATTGTTGTTGACTGAAATTTTTTATTTTTTTTGGGGTA
ATAGGATTTCCTTTTTTATGAAGATTAATATCTTTGACTCGTGAAACATT
CTTTTTAACTTTTGTTTTTTCTGTTGGTTTATCATTTGTTTTTTCACTAA
TTTCAATACCATCTTGACGTTCATTCATAACTTCATCTTTTTTTTTTCCT
GTTTCTGTATCTTCTTCTATTTTTTTTTCTTTATCTTTTTCTTTATCTTC
TTCTTGTTCTTCCTCTTCTTTTTCTTCTTCTGATACTGCAGGTGTTTCTT
CTTCTTCTTCTTCCGATATTGTCGGTTTTTCTACTTCTTCTTCTTGTTCT
TCTTCTTCTTCTTCTTCTTCTTCTTCTTCTTCTTCTTCTTCTTCTTCTTC
CGGTAATTTATTAATTATATTTCTTTTTTTATATGAATTACGTTTGGTTT
GTGCAGTAATTTCCTTACATAGAGTGCAGCTTTCAAGAAAAATTTCAATT
TCTTCGTTTGTTGCATAATAACCACTGTCTTTGATATGATTAAACATTTT
TGATTTTCTTAAATGCTTTCCTTCTTTAATATGAAAATTATCGAATTCTA
ATTCATTAAGAACAATAAGCTCCCCTAATTTAAAAAATTAGTTAAAATAA
ATTAAAATGAACATGTATAAAGATGGATTTTACCATTTTTTGAAATTCTA
AATAACTTTTCTTCATCTCCAATCTTTTTGACTGAAAAACGATTTTTAAT
TGAAGTTATTGTTCTGTGAGTGTTTTGAATCGCCCATTTCTCTAAATCAG
TTTGAGATAGTGTTTTATAATCTGAATTGTTATACACAACTTTTGCTCTA
TTAACCAAATATTTAAAGATTTCATCATCAACTGAATATTTTGACTTTAC
GATTCTTGTCCAAAAAACAATTTCTACTACTATCATTTTTTATTTATAAA
ATAATTTAAATACAAAAATGAATTTTTTTTTTTTTAAAAAAAAAAAAAATT
TGAAAAAAAAAAAAAAAAAAATTTTAAAAAAAAAAAAAAAAAAAAAAAAAA
AAAAAAAATCAAATAAAAAGTAAAAAATAAAAACCGAAAAACATTCATTG
TAATTTCAAATGTCGAGGCCGGCAGAGGCGGTTTGCGTATTGGGCGCTCT
TCCGCTTCCTCGCTCACTGACTCGCTGCGCTCGGTCGTTCGGCTGCGGCG
AGCGGTATCAGCTCACTCAAAGGCGGTAATACGGTTATCCACAGAATCAG
GGGATAACGCAGGAAAGAACATGTGAGCAAAAGGCCAGCAAAAGGCCAGG

AACCGTAAAAAGGCCGCGTTGCTGGCGTTTTTCCATAGGCTCCGCCCCCC
TGACGAGCATCACAAAAATCGACGCTCAAGTCAGAGGTGGCGAAACCCGA
CAGGACTATAAAGATACCAGGCGTTTCCCCCTGGAAGCTCCCTCGTGCGC
TCTCCTGTTCCGACCCTGCCGCTTACCGGATACCTGTCCGCCTTTCTCCC
TTCGGGAAGCGTGGCGCTTTCTCATAGCTCACGCTGTAGGTATCTCAGTT
CGGTGTAGGTCGTTCGCTCCAAGCTGGGCTGTGTGCACGAACCCCCCGTT
CAGCCCGACCGCTGCGCCTTATCCGGTAACTATCGTCTTGAGTCCAACCC
GGTAAGACACGACTTATCGCCACTGGCAGCAGCCACTGGTAACAGGATTA
GCAGAGCGAGGTATGTAGGCGGTGCTACAGAGTTCTTGAAGTGGTGGCCT
AACTACGGCTACACTAGAAGGACAGTATTTGGTATCTGCGCTCTGCTGAA
GCCAGTTACCTTCGGAAAAAGAGTTGGTAGCTCTTGATCCGGCAAACAAA
CCACCGCTGGTAGCGGTGGTTTTTTTGTTTGCAAGCAGCAGATTACGCGC
AGAAAAAAAGGATCTCAAGAAGATCCTTTGATCTTTTCTACGGGGTCTGA
CGCTCAGTGGAACGAAAACTCACGTTAAGGGATTTTGGTCATGAGATTAT
CAAAAAGGATCTTCACCTAGATCCTTTTAAATTAAAAATGAAGTTTTAAA
TCAATCTAAAGTATATATGAGTAAACTTGGTCTGACAGTTACCAATGCTT
AATCAGTGAGGCACCTATCTCAGCGATCTGTCTATTTCGTTCATCCATAG
TTGCCTGACTCCCCGTCGTGTAGATAACTACGATACGGGAGGGCTTACCA
TCTGGCCCCAGTGCTGCAATGATACCGCGAGACCCACGCTCACCGGCTCC
AGATTTATCAGCAATAAACCAGCCAGCCGGAAGGGCCGAGCGCAGAAGTG
GTCCTGCAACTTTATCCGCCTCCATCCAGTCTATTAATTGTTGCCGGGAA
GCTAGAGTAAGTAGTTCGCCAGTTAATAGTTTGCGCAACGTTGTTGCCAT
TGCTACAGGCATCGTGGTGTCACGCTCGTCGTTTGGTATGGCTTCATTCA
GCTCCGGTTCCCAACGATCAAGGCGAGTTACATGATCCCCCATGTTGTGC
AAAAAAGCGGTTAGCTCCTTCGGTCCTCCGATCGTTGTCAGAAGTAAGTT
GGCCGCAGTGTTATCACTCATGGTTATGGCAGCACTGCATAATTCTCTTA
CTGTCATGCCATCCGTAAGATGCTTTTCTGTGACTGGTGAGTACTCAACC
AAGTCATTCTGAGAATAGTGTATGCGGCGACCGAGTTGCTCTTGCCCGGC
GTCAATACGGGATAATACCGCGCCACATAGCAGAACTTTAAAAGTGCTCA
TCATTGGAAAACGTTCTTCGGGGCGAAAACTCTCAAGGATCTTACCGCTG
TTGAGATCCAGTTCGATGTAACCCACTCGTGCACCCAACTGATCTTCAGC
ATCTTTTACTTTCACCAGCGTTTCTGGGTGAGCAAAAACAGGAAGGCAAA
ATGCCGCAAAAAAGGGAATAAGGGCGACACGGAAATGTTGAATACTCATA
CTCTTCCTTTTTCAATATTATTGAAGCATTTATCAGGGTTATTGTCTCAT
GAGCGGATACATATTTGAATGTATTTAGAAAAATAAACAAATAGGGGTTC
CGCGCACATTTCCCCGAAAAGTGCCACCTGACGCGCCCTGTAGCGGGATC
CATTTTATTTAATATACTAAATAATAAAAAAGTTAAAAAATGATCATTGG
ATAAATTTTTTATAATTATAAATAAAGATAATAATTTTTTTTTTTAACAAA
ACTAAAAATAAAAATAATAAAATAATTGTTAAAATAGGTTTTTTTTTTTT
TTTTTTTTTTTTAATAAATGGTATTTATTAATTTATTTGTTGTGTGTGTT
TTTTTTTTTATAATATTTTTTTTTTTTAGCATTGAATTAAGAAGAAATCAA
ATTGATGCGGCCGCTCAGAAGAACTCGTCAAGAAGGCGATAGAAGGCGAT
GCGCTGCGAATCGGGAGCGGCGATACCGTAAAGCACGAGGAAGCGGTCAG
CCCATTCGCCGCCAAGCTCTTCAGCAATATCACGGGTAGCCAACGCTATG
TCCTGATAGCGGTCCGCCACACCCAGCCGTCCACAGTCGATGAATCCAGA
AAAGCGGCCATTTTCCACCATGATATTCGGCAAGCAGGCATCGCCATGGG
TCACGACGAGATCCTCGCCGTCGGGCATGCGCGCCTTGAGCCTGGCGAAC
AGTTCGGCTGGCGCGAGCCCCTGATGCTCTTCGTCCAGATCATCCTGATC
GACAAGACCGGCTTCCATCCGAGTACGTGCTCGCTCGATGCGATGTTTCG
CTTGGTGGTCGAATGGGCAGGTAGCCGGATCAAGCGTATGCAGCCGCCGC

ATTGCATCAGCCATGATGGATACTTTCTCGGCAGGAGCAAGGTGAGATGA
CAGGAGATCCTGCCCCGGCACTTCGCCCAATAGCAGCCAGTCCCTTCCCG
CTTCAGTGACAACGTCGAGCACAGCTGCGCAAGGAACGCCCGTCGTGGCC
AGCCACGATAGCCGCGCTGCCTCGTCCTGCAGTTCATTCAGGGCACCGGA
CAGGTCGGTCTTGACAAAAAGAACCGGGCGCCCCTGCGCTGACAGCCGGA
ACACGGCGGCATCAGAGCAGCCGATTGTCTGTTGTGCCCAGTCATAGCCG
AATAGCCTCTCCACCCAAGCGGCCGGAGAACCTGCGTGCAATCCATCTTG
TTCAATCATGCGAAACGATCCAGCTTGAACATCTTCACCATCCATTTTTT
GCTAGCTGTGAAATTAGTTTAAAATACAAATAAAGAGTTATAATAATATA
CAGTTGAATAAAAAAAAAAAAAAATGAATTGGAAAATTTATTTTTATATGA
AGAAAAAAAAATTTTGAAAAAAAAAAAAAAAATTAAAAAAAAAAAAAAAA
AAAAAAAATTTAAAATTATTCCACTGTGGGGGGCCCCAAATTTTATTTAA
AAAAAAAAAAAAAAATGGGTCCCTTTTGGGGGGTTGGAAAAAAAAAAAAAA
AAAAAAAAAAAAAATTGAAAATATAATGTTAGTCATATGATTAATCATT

pDM1210 – mCherry (C)

CTCGAGACTAGAGCTAGATAAAAAAAATTTTTATTTATTTTTATTTATTT
TGAATTAAATAGATTACAAATTAATTAATCCCATCAAATCTTTAAAAAAA
AATGGTTTAAAAAAACTTGGGTTGGTTAATTATTATTTGAAAATTTTAAA
ACCCAAATTAAAAAAAAAAAAATGGGATTCAAAAATTTTTTTTTTTTTTT
TTTTTTTTTTTTTTTTTTTTTTTCAGATTGCATAAAAAGATTTTTTTTTT
TTTTTTTTCTTATTTCTTAAAACAAATAAATTAAATTAAATAAAAAAATAA
AAATCAGATCTTCAACTAGTGGTGGTTCAGGAGGTAGTGTTTCAAAAGGT
GAAGAAGATAATATGGCAATTATTAAAGAATTTATGAGATTTAAAGTTCA
TATGGAAGGTTCAGTTAATGGTCATGAATTTGAAATTGAAGGTGAAGGTG
AAGGTAGACCATATGAAGGTACACAAACAGCAAAATTAAAAGTTACAAAA
GGTGGTCCATTACCATTTGCATGGGATATTTTATCACCACAATTTATGTA
TGGTTCAAAAGCATATGTTAAACATCCAGCAGATATTCCAGATTATTTAA
AATTATCATTTCCAGAAGGTTTTAAATGGGAAAGAGTTATGAATTTTGAA
GATGGTGGTGTTGTTACAGTTACACAAGATTCATCATTACAAGATGGTGA
ATTTATTTATAAAGTTAAATTAAGAGGTACAAATTTTCCATCAGATGGTC
CAGTTATGCAAAAGAAAACAATGGGTTGGGAAGCATCATCAGAAAGAATG
TATCCAGAAGATGGTGCATTAAAAGGTGAAATTAAACAAAGATTAAAATT
AAAAGATGGTGGTCATTATGATGCAGAAGTTAAAACAACATATAAAGCAA
AAAAACCAGTTCAATTACCAGGTGCATATAATGTTAATATTAAATTAGAT
ATTACATCACATAATGAAGATTATACAATTGTTGAACAATATGAAAGAGC
AGAAGGTAGACATTCAACAGGTGGTATGGATGAATTATATAAATAAGCTA
GTTAAATAAATAAATTATTTAATAAATAATAAAAAAACAAATTGTTGTAA
TAATCTAATATTTTCTTTTTTTTTTAATTTTTTTTTTTTAAATCTTAATA
ATTATTAAGTTATTTTAATTTTTTTTTTTTTTTTTTTTTTTTTTTTTTTT
TTTTTTCTATCAAAAAAATCAAATATATTTAAAAAATTTATTATTTACAG
ATACATTTTGAATGGTGAAGATAAATATATGCATTAGATGTAAAACAGCC
AAAGAGTATGAAAATCAAAAAGATAAAGCTTATCGATTTCGAAAAAGTAA
ATAGCAATTATTACAAAATTCAATCCGAATCTACCCAAATAAATTCCAAT
GAAATTGCCGATTTAAAAAAGTTTATTAAAGAAGAAGTCAATAAAACTTC
TTCCAAAATTGATTTCTTTTTAGTTTCTTCAACAGATGCCCTTTCAAATC
CAGAAAATTATTCTCTCTTAGAAGTAAAGTGTATTAATTGTCATTCTTTG
TGTCAAGGAAAAAATTTATATATTTCATGTACAAGAGATGGATGTCAAAA
CAATATTTGCTATAATTGTTTAGGAATAAACATAAACATATATAATGTTG

TTATTAATTCTAAACTTTGCCCTCCATGTTTCAATGATTCGGTAATCAAC
AAGAAGTGTGCCATGTGTAGTAAGAACGGAACTAAATGTAATTTGAACCA
AGAATGTAAACTTCATCTTTGTGCACAGTGTTCTAAAAAGTGTCTATACA
TTCTGAGAGTCAAAACTAATTAAATAAAATATAAACTTAATTTCTAAATA
AACTCATTTAAAAATATTTAAATAATATGAATTTATAACTGTAATTATTG
TATTAAAAAATTATATAATTATTTAATGTTAAAAATGTATTAAAATAATT
ATAAAAAAATATAACAAAAATTTTCGTAAAAATAATTTGTAAAAAAGCTA
TTAAAAATATTATGAAAAAAAAATTAAAAAAATTATTAAATTGTTTTTGT
AATTAAGCTATTAAAATAATTATAAAAAAAAAATTTTTAAAATTTTAAAA
ATATTTTTTGTAAAAAAGTATTAAAATAATTATGAAAAAAAAATTTTCTA
AAAAATTAAAAAAAAAATTAAAATATATTTTATGTTAAAAACGTATTAAA
ATAACTATTAAAAAAATTATATTTAAAAAAGTATTAACTTTTTTTTAGGT
GTGGTTGTGGGGTGGGGTTTAATATATTATAATAAAAAATTATTTTTTGT
TCATTTATTATTTTCATTGTATATAATGTACTCAACAACGTTATTATTTT
TTCTTTTTTTTTTTATTGTATCAAAATCTTCTGTTCTTCAAAATGATCAG
ATTGAAGTAAAATATTTTCAACTTCTTATTGTTATGTATCAAAAAGAAAA
CTGTGTTGAAAAGTCAATGACAGGCGCCGTAATTTATGATGAATGTAATA
TTCATGGAAGAGTTGAAACAAATAGTACTCATGCGCTTTTTTATGATGAC
ATTGAAACAAATAATTCAAGATGTAACAATTTTCGTAATTTAACAAACTT
AATTAAACTTAATGAATGTATTAATGACGAGTTTGGAGAGTCTATTCTTT
ATAAAGAATATAATGAAACTGATGATGGTTATTTGTTTAGAGTGGAAGAC
AGCTTTGTTGAAATTACTTCTCTTTCAATGGATTGTACAAAAAATAGTAA
AACAATTATTGAAAAATTCAACATTTGTTCAAAATTTGAAAATGTATATC
ATATTACAAACATTACACAAGAGAAATCCAATAGATTTACATGTACAGAT
CCATTGTGCCACTATTGTAAGAATGAAAACATTCAAAACAATCTTGATTT
TAAAACAACAAAGTGTACTCCAAAGTATGGTGCATCTGATTCTGAATTTT
TATCAACAATTTACAATCCAAAGCTCGATGGCTCAAATAACGGTATGGAA
AAGTCAGTAACTCAAGAAAAAAACATTTCAAATAATTTAAAAATTAATAT
ATATTTAATTTTCTTTTTAATTATTTTTTTAATTAAATAAAGTTTTATTA
TTTTTTAAGAGTAATTATTGCTCTTTTTTCATTTGAAACACCAGAAGCTA
AACGTAATTGTTGTTGACTGAAATTTTTTATTTTTTTTGGGGTAATAGGA
TTTCCTTTTTTATGAAGATTAATATCTTTGACTCGTGAAACATTCTTTTT
AACTTTTGTTTTTTCTGTTGGTTTATCATTTGTTTTTTCACTAATTTCAA
TACCATCTTGACGTTCATTCATAACTTCATCTTTTTTTTTTCCTGTTTCT
GTATCTTCTTCTATTTTTTTTTCTTTATCTTTTTCTTTATCTTCTTCTTG
TTCTTCCTCTTCTTTTTCTTCTTCTGATACTGCAGGTGTTTCTTCTTCTT
CTTCTTCCGATATTGTCGGTTTTTCTACTTCTTCTTCTTGTTCTTCTTCT
TCTTCTTCTTCTTCTTCTTCTTCTTCTTCTTCTTCTTCTTCTTCCGGTAA
TTTATTAATTATATTTCTTTTTTTATATGAATTACGTTTGGTTTGTGCAG
TAATTTCCTTACATAGAGTGCAGCTTTCAAGAAAAATTTCAATTTCTTCG
TTTGTTGCATAATAACCACTGTCTTTGATATGATTAAACATTTTTGATTT
TCTTAAATGCTTTCCTTCTTTAATATGAAAATTATCGAATTCTAATTCAT
TAAGAACAATAAGCTCCCCTAATTTAAAAAATTAGTTAAAATAAATTAAA
ATGAACATGTATAAAGATGGATTTTACCATTTTTTGAAATTCTAAATAAC
TTTTCTTCATCTCCAATCTTTTTGACTGAAAAACGATTTTTAATTGAAGT
TATTGTTCTGTGAGTGTTTTGAATCGCCCATTTCTCTAAATCAGTTTGAG
ATAGTGTTTTATAATCTGAATTGTTATACACAACTTTTGCTCTATTAACC
AAATATTTAAAGATTTCATCATCAACTGAATATTTTGACTTTACGATTCT
TGTCCAAAAAACAATTTCTACTACTATCATTTTTTATTTATAAAATAATT
TAAATACAAAAATGAATTTTTTTTTTTTTAAAAAAAAAAAAAATTTGAAAA

AAAAAAAAAAAAAAATTTTAAAAAAAAAAAAAAAAAAAAAAAAAAAAAAAAA
AATCAAATAAAAAGTAAAAAATAAAAACCGAAAAACATTCATTGTAATTT
CAAATGTCGAGGCCGGCAGAGGCGGTTTGCGTATTGGGCGCTCTTCCGCT
TCCTCGCTCACTGACTCGCTGCGCTCGGTCGTTCGGCTGCGGCGAGCGGT
ATCAGCTCACTCAAAGGCGGTAATACGGTTATCCACAGAATCAGGGGATA
ACGCAGGAAAGAACATGTGAGCAAAAGGCCAGCAAAAGGCCAGGAACCGT
AAAAAGGCCGCGTTGCTGGCGTTTTTCCATAGGCTCCGCCCCCCTGACGA
GCATCACAAAAATCGACGCTCAAGTCAGAGGTGGCGAAACCCGACAGGAC
TATAAAGATACCAGGCGTTTCCCCCTGGAAGCTCCCTCGTGCGCTCTCCT
GTTCCGACCCTGCCGCTTACCGGATACCTGTCCGCCTTTCTCCCTTCGGG
AAGCGTGGCGCTTTCTCATAGCTCACGCTGTAGGTATCTCAGTTCGGTGT
AGGTCGTTCGCTCCAAGCTGGGCTGTGTGCACGAACCCCCCGTTCAGCCC
GACCGCTGCGCCTTATCCGGTAACTATCGTCTTGAGTCCAACCCGGTAAG
ACACGACTTATCGCCACTGGCAGCAGCCACTGGTAACAGGATTAGCAGAG
CGAGGTATGTAGGCGGTGCTACAGAGTTCTTGAAGTGGTGGCCTAACTAC
GGCTACACTAGAAGGACAGTATTTGGTATCTGCGCTCTGCTGAAGCCAGT
TACCTTCGGAAAAAGAGTTGGTAGCTCTTGATCCGGCAAACAAACCACCG
CTGGTAGCGGTGGTTTTTTTGTTTGCAAGCAGCAGATTACGCGCAGAAAA
AAAGGATCTCAAGAAGATCCTTTGATCTTTTCTACGGGGTCTGACGCTCA
GTGGAACGAAAACTCACGTTAAGGGATTTTGGTCATGAGATTATCAAAAA
GGATCTTCACCTAGATCCTTTTAAATTAAAAATGAAGTTTTAAATCAATC
TAAAGTATATATGAGTAAACTTGGTCTGACAGTTACCAATGCTTAATCAG
TGAGGCACCTATCTCAGCGATCTGTCTATTTCGTTCATCCATAGTTGCCT
GACTCCCCGTCGTGTAGATAACTACGATACGGGAGGGCTTACCATCTGGC
CCCAGTGCTGCAATGATACCGCGAGACCCACGCTCACCGGCTCCAGATTT
ATCAGCAATAAACCAGCCAGCCGGAAGGGCCGAGCGCAGAAGTGGTCCTG
CAACTTTATCCGCCTCCATCCAGTCTATTAATTGTTGCCGGGAAGCTAGA
GTAAGTAGTTCGCCAGTTAATAGTTTGCGCAACGTTGTTGCCATTGCTAC
AGGCATCGTGGTGTCACGCTCGTCGTTTGGTATGGCTTCATTCAGCTCCG
GTTCCCAACGATCAAGGCGAGTTACATGATCCCCCATGTTGTGCAAAAAA
GCGGTTAGCTCCTTCGGTCCTCCGATCGTTGTCAGAAGTAAGTTGGCCGC
AGTGTTATCACTCATGGTTATGGCAGCACTGCATAATTCTCTTACTGTCA
TGCCATCCGTAAGATGCTTTTCTGTGACTGGTGAGTACTCAACCAAGTCA
TTCTGAGAATAGTGTATGCGGCGACCGAGTTGCTCTTGCCCGGCGTCAAT
ACGGGATAATACCGCGCCACATAGCAGAACTTTAAAAGTGCTCATCATTG
GAAAACGTTCTTCGGGGCGAAAACTCTCAAGGATCTTACCGCTGTTGAGA
TCCAGTTCGATGTAACCCACTCGTGCACCCAACTGATCTTCAGCATCTTT
TACTTTCACCAGCGTTTCTGGGTGAGCAAAAACAGGAAGGCAAAATGCCG
CAAAAAAGGGAATAAGGGCGACACGGAAATGTTGAATACTCATACTCTTC
CTTTTTCAATATTATTGAAGCATTTATCAGGGTTATTGTCTCATGAGCGG
ATACATATTTGAATGTATTTAGAAAAATAAACAAATAGGGGTTCCGCGCA
CATTTCCCCGAAAAGTGCCACCTGACGCGCCCTGTAGCGGGATCCATTTT
ATTTAATATACTAAATAATAAAAAAGTTAAAAAATGATCATTGGATAAAT
TTTTTATAATTATAAATAAAGATAATAATTTTTTTTTTAACAAAACTAAA
AATAAAAAATAATAAAATAATTGTTAAAATAGGTTTTTTTTTTTTTTTTT
TTTTTTAATAAATGGTATTTATTAATTTATTTGTTGTGTGTGTTTTTTTT
TTTATAATATTTTTTTTTTTTAGCATTGAATTAAGAAGAAATCAAATTGAT
GCGGCCGCTCAGAAGAACTCGTCAAGAAGGCGATAGAAGGCGATGCGCTG
CGAATCGGGAGCGGCGATACCGTAAAGCACGAGGAAGCGGTCAGCCCATT
CGCCGCCAAGCTCTTCAGCAATATCACGGGTAGCCAACGCTATGTCCTGA

TAGCGGTCCGCCACACCCAGCCGTCCACAGTCGATGAATCCAGAAAAGCG
GCCATTTTCCACCATGATATTCGGCAAGCAGGCATCGCCATGGGTCACGA
CGAGATCCTCGCCGTCGGGCATGCGCGCCTTGAGCCTGGCGAACAGTTCG
GCTGGCGCGAGCCCCTGATGCTCTTCGTCCAGATCATCCTGATCGACAAG
ACCGGCTTCCATCCGAGTACGTGCTCGCTCGATGCGATGTTTCGCTTGGT
GGTCGAATGGGCAGGTAGCCGGATCAAGCGTATGCAGCCGCCGCATTGCA
TCAGCCATGATGGATACTTTCTCGGCAGGAGCAAGGTGAGATGACAGGAG
ATCCTGCCCCGGCACTTCGCCCAATAGCAGCCAGTCCCTTCCCGCTTCAG
TGACAACGTCGAGCACAGCTGCGCAAGGAACGCCCGTCGTGGCCAGCCAC
GATAGCCGCGCTGCCTCGTCCTGCAGTTCATTCAGGGCACCGGACAGGTC
GGTCTTGACAAAAAGAACCGGGCGCCCCTGCGCTGACAGCCGGAACACGG
CGGCATCAGAGCAGCCGATTGTCTGTTGTGCCCAGTCATAGCCGAATAGC
CTCTCCACCCAAGCGGCCGGAGAACCTGCGTGCAATCCATCTTGTTCAAT
CATGCGAAACGATCCAGCTTGAACATCTTCACCATCCATTTTTTGCTAGC
TGTGAAATTAGTTTAAAATACAAATAAAGAGTTATAATAATATACAGTTG
AATAAAAAAAAAAAAAAATGAATTGGAAAATTTATTTTTATATGAAGAAAA
AAAAATTTTGAAAAAAAAAAAAAAAATTAAAAAAAAAAAAAAAAAAAAAAA
AATTTAAAATTATTCCACTGTGGGGGGCCCCAAATTTTATTTAAAAAAAA
AAAAAAAATGGGTCCCTTTTGGGGGGTTGGAAAAAAAAAAAAAAAAAAAA
AAAAAAATTGAAAATATAATGTTAGTCATATGATTAATCATT

pPI143 – mNeon (C)

CTCGAGACTAGAGCTAGATAAAAAAAATTTTTATTTATTTTTATTTATTT
TGAATTAAATAGATTACAAATTAATTAATCCCATCAAATCTTTAAAAAAA
AATGGTTTAAAAAAACTTGGGTTGGTTAATTATTATTTGAAAATTTTAAA
ACCCAAATTAAAAAAAAAAAAAATGGGATTCAAAAATTTTTTTTTTTTTTT
TTTTTTTTTTTTTTTTTTTTTTTCAGATTGCATAAAAAGATTTTTTTTTT
TTTTTTTTCTTATTTCTTAAAACAAATAAATTAAATTAAATAAAAAATAA
AAATCAGATCTTCAACTAGTGGTGGTTCAGGAGGTAGTGTGAGTAAAGGT
GAAGAAGATAATATGGCATCGTTACCAGCTACACATGAGTTACATATATT
CGGTAGCATTAATGGTGTTGATTTTGATATGGTGGGACAAGGTACCGGTA
ATCCTAATGATGGTTACGAAGAACTAAATTTAAAATCGACTAAAGGTGAC
TTACAATTTTCTCCATGGATTTTAGTGCCACATATAGGGTATGGTTTTCA
TCAATACTTACCATATCCAGATGGTATGTCACCATTTCAAGCTGCAATGG
TTGATGGATCAGGTTATCAAGTTCATAGAACAATGCAATTTGAAGATGGT
GCTTCATTAACTGTTAATTATAGATACACATATGAAGGCTCACATATTAA
AGGTGAAGCTCAAGTTAAAGGTACTGGTTTCCCAGCCGATGGCCCAGTTA
TGACAAATAGTTTAACAGCAGCAGATTGGTGTAGATCCAAAAAAACTTAT
CCAAATGATAAAACAATTATTTCAACTTTTAAATGGTCATATACAACCGG
TAATGGTAAACGTTATCGTTCAACAGCCCGTACAACATATACTTTTGCTA
AACCAATGGCAGCTAATTATTTAAAAAATCAACCAATGTATGTTTTTCGT
AAAACAGAGTTAAAACATTCAAAAACAGAACTTAATTTTAAAGAATGGCA
AAAAGCATTTACAGACGTTATGTAAGCTAGTAGTTAAATAAATAAATTAT
TTAATAAATAATAAAAAAACAAATTGTTGTAATAATCTAATATTTTCTTT
TTTTTTTAATTTTTTTTTTTTTAAATCTTAATAATTATTAAGTTATTTTAA
TTTTTTTTTTTTTTTTTTTTTTTTTTTTTTTTTTTTTTTCTATCAAAAAAA
TCAAATATATTTAAAAAATTTATTATTTACAGATACATTTTGAATGGTGA
AGATAAATATATGCATTAGATGTAAAACAGCCAAAGAGTATGAAAATCAA
AAAGATAAAGCTTATCGATTTCGAAAAAGTAAATAGCAATTATTACAAAA

TTCAATCCGAATCTACCCAAATAAATTCCAATGAAATTGCCGATTTAAAA
AAGTTTATTAAAGAAGAAGTCAATAAAACTTCTTCCAAAATTGATTTCTT
TTTAGTTTCTTCAACAGATGCCCTTTCAAATCCAGAAAATTATTCTCTCT
TAGAAGTAAAGTGTATTAATTGTCATTCTTTGTGTCAAGGAAAAAATTTA
TATATTTCATGTACAAGAGATGGATGTCAAAACAATATTTGCTATAATTG
TTTAGGAATAAACATAAACATATATAATGTTGTTATTAATTCTAAACTTT
GCCCTCCATGTTTCAATGATTCGGTAATCAACAAGAAGTGTGCCATGTGT
AGTAAGAACGGAACTAAATGTAATTTGAACCAAGAATGTAAACTTCATCT
TTGTGCACAGTGTTCTAAAAAGTGTCTATACATTCTGAGAGTCAAAACTA
ATTAAATAAAATATAAACTTAATTTCTAAATAAACTCATTTAAAAATATT
TAAATAATATGAATTTATAACTGTAATTATTGTATTAAAAAATTATATAA
TTATTTAATGTTAAAAATGTATTAAAATAATTATAAAAAAATATAACAAA
AATTTTCGTAAAAATAATTTGTAAAAAAGCTATTAAAAATATTATGAAAA
AAAAATTAAAAAAATTATTAAATTGTTTTTGTAATTAAGCTATTAAAATA
ATTATAAAAAAAAAATTTTTAAAATTTTAAAAATATTTTTTGTAAAAAAG
TATTAAAATAATTATGAAAAAAAAATTTTCTAAAAAATTAAAAAAAAAAT
TAAAATATATTTTATGTTAAAAACGTATTAAAATAACTATTAAAAAAATT
ATATTTAAAAAAGTATTAACTTTTTTTTAGGTGTGGTTGTGGGGTGGGGT
TTAATATATTATAATAAAAAAATTATTTTTTGTTCATTTATTATTTTCATT
GTATATAATGTACTCAACAACGTTATTATTTTTTCTTTTTTTTTTTATTG
TATCAAAATCTTCTGTTCTTCAAAATGATCAGATTGAAGTAAAATATTTT
CAACTTCTTATTGTTATGTATCAAAAAGAAAACTGTGTTGAAAAGTCAAT
GACAGGCGCCGTAATTTATGATGAATGTAATATTCATGGAAGAGTTGAAA
CAAATAGTACTCATGCGCTTTTTTATGATGACATTGAAACAAATAATTCA
AGATGTAACAATTTTCGTAATTTAACAAACTTAATTAAACTTAATGAATG
TATTAATGACGAGTTTGGAGAGTCTATTCTTTATAAAGAATATAATGAAA
CTGATGATGGTTATTTGTTTAGAGTGGAAGACAGCTTTGTTGAAATTACT
TCTCTTTCAATGGATTGTACAAAAAATAGTAAAACAATTATTGAAAAATT
CAACATTTGTTCAAAATTTGAAAATGTATATCATATTACAAACATTACAC
AAGAGAAATCCAATAGATTTACATGTACAGATCCATTGTGCCACTATTGT
AAGAATGAAAACATTCAAAACAATCTTGATTTTAAAACAACAAAGTGTAC
TCCAAAGTATGGTGCATCTGATTCTGAATTTTTATCAACAATTTACAATC
CAAAGCTCGATGGCTCAAATAACGGTATGGAAAAGTCAGTAACTCAAGAA
AAAAACATTTCAAATAATTTAAAAATTAATATATATTTAATTTTCTTTTT
AATTATTTTTTTAATTAAATAAAGTTTTATTATTTTTTAAGAGTAATTAT
TGCTCTTTTTTCATTTGAAACACCAGAAGCTAAACGTAATTGTTGTTGAC
TGAAATTTTTTATTTTTTTTGGGGTAATAGGATTTCCTTTTTTATGAAGA
TTAATATCTTTGACTCGTGAAACATTCTTTTTAACTTTTGTTTTTTCTGT
TGGTTTATCATTTGTTTTTTCACTAATTTCAATACCATCTTGACGTTCAT
TCATAACTTCATCTTTTTTTTTTCCTGTTTCTGTATCTTCTTCTATTTTT
TTTTCTTTATCTTTTTCTTTATCTTCTTCTTGTTCTTCCTCTTCTTTTTC
TTCTTCTGATACTGCAGGTGTTTCTTCTTCTTCTTCTTCCGATATTGTCG
GTTTTTCTACTTCTTCTTCTTGTTCTTCTTCTTCTTCTTCTTCTTCTTCT
TCTTCTTCTTCTTCTTCTTCTTCTTCCGGTAATTTATTAATTATATTTCT
TTTTTTATATGAATTACGTTTGGTTTGTGCAGTAATTTCCTTACATAGAG
TGCAGCTTTCAAGAAAAATTTCAATTTCTTCGTTTGTTGCATAATAACCA
CTGTCTTTGATATGATTAAACATTTTTGATTTTCTTAAATGCTTTCCTTC
TTTAATATGAAAATTATCGAATTCTAATTCATTAAGAACAATAAGCTCCC
CTAATTTAAAAAATTAGTTAAAATAAATTAAAATGAACATGTATAAAGAT
GGATTTTACCATTTTTTGAAATTCTAAATAACTTTTCTTCATCTCCAATC

TTTTTGACTGAAAAACGATTTTTAATTGAAGTTATTGTTCTGTGAGTGTT
TTGAATCGCCCATTTCTCTAAATCAGTTTGAGATAGTGTTTTATAATCTG
AATTGTTATACACAACTTTTGCTCTATTAACCAAATATTTAAAGATTTCA
TCATCAACTGAATATTTTGACTTTACGATTCTTGTCCAAAAAACAATTTC
TACTACTATCATTTTTTATTTATAAAATAATTTAAATACAAAAATGAATT
TTTTTTTTTTTAAAAAAAAAAAAATTTGAAAAAAAAAAAAAAAAAATTTT
AAAAAAAAAAAAAAAAAAAAAAAAAAAAAAAAAATCAAATAAAAAGTAAA
AAATAAAAACCGAAAAACATTCATTGTAATTTCAAATGTCGAGGCCGGCA
GAGGCGGTTTGCGTATTGGGCGCTCTTCCGCTTCCTCGCTCACTGACTCG
CTGCGCTCGGTCGTTCGGCTGCGGCGAGCGGTATCAGCTCACTCAAAGGC
GGTAATACGGTTATCCACAGAATCAGGGGATAACGCAGGAAAGAACATGT
GAGCAAAAGGCCAGCAAAAGGCCAGGAACCGTAAAAAGGCCGCGTTGCTG
GCGTTTTTCCATAGGCTCCGCCCCCCTGACGAGCATCACAAAAATCGACG
CTCAAGTCAGAGGTGGCGAAACCCGACAGGACTATAAAGATACCAGGCGT
TTCCCCCTGGAAGCTCCCTCGTGCGCTCTCCTGTTCCGACCCTGCCGCTT
ACCGGATACCTGTCCGCCTTTCTCCCTTCGGGAAGCGTGGCGCTTTCTCA
TAGCTCACGCTGTAGGTATCTCAGTTCGGTGTAGGTCGTTCGCTCCAAGC
TGGGCTGTGTGCACGAACCCCCCGTTCAGCCCGACCGCTGCGCCTTATCC
GGTAACTATCGTCTTGAGTCCAACCCGGTAAGACACGACTTATCGCCACT
GGCAGCAGCCACTGGTAACAGGATTAGCAGAGCGAGGTATGTAGGCGGTG
CTACAGAGTTCTTGAAGTGGTGGCCTAACTACGGCTACACTAGAAGGACA
GTATTTGGTATCTGCGCTCTGCTGAAGCCAGTTACCTTCGGAAAAAGAGT
TGGTAGCTCTTGATCCGGCAAACAAACCACCGCTGGTAGCGGTGGTTTTT
TTGTTTGCAAGCAGCAGATTACGCGCAGAAAAAAAGGATCTCAAGAAGAT
CCTTTGATCTTTTCTACGGGGTCTGACGCTCAGTGGAACGAAAACTCACG
TTAAGGGATTTTGGTCATGAGATTATCAAAAAGGATCTTCACCTAGATCC
TTTTAAATTAAAAATGAAGTTTTAAATCAATCTAAAGTATATATGAGTAA
ACTTGGTCTGACAGTTACCAATGCTTAATCAGTGAGGCACCTATCTCAGC
GATCTGTCTATTTCGTTCATCCATAGTTGCCTGACTCCCCGTCGTGTAGA
TAACTACGATACGGGAGGGCTTACCATCTGGCCCCAGTGCTGCAATGATA
CCGCGAGACCCACGCTCACCGGCTCCAGATTTATCAGCAATAAACCAGCC
AGCCGGAAGGGCCGAGCGCAGAAGTGGTCCTGCAACTTTATCCGCCTCCA
TCCAGTCTATTAATTGTTGCCGGGAAGCTAGAGTAAGTAGTTCGCCAGTT
AATAGTTTGCGCAACGTTGTTGCCATTGCTACAGGCATCGTGGTGTCACG
CTCGTCGTTTGGTATGGCTTCATTCAGCTCCGGTTCCCAACGATCAAGGC
GAGTTACATGATCCCCCATGTTGTGCAAAAAAGCGGTTAGCTCCTTCGGT
CCTCCGATCGTTGTCAGAAGTAAGTTGGCCGCAGTGTTATCACTCATGGT
TATGGCAGCACTGCATAATTCTCTTACTGTCATGCCATCCGTAAGATGCT
TTTCTGTGACTGGTGAGTACTCAACCAAGTCATTCTGAGAATAGTGTATG
CGGCGACCGAGTTGCTCTTGCCCGGCGTCAATACGGGATAATACCGCGCC
ACATAGCAGAACTTTAAAAGTGCTCATCATTGGAAAACGTTCTTCGGGGC
GAAAACTCTCAAGGATCTTACCGCTGTTGAGATCCAGTTCGATGTAACCC
ACTCGTGCACCCAACTGATCTTCAGCATCTTTTACTTTCACCAGCGTTTC
TGGGTGAGCAAAAACAGGAAGGCAAAATGCCGCAAAAAAGGGAATAAGGG
CGACACGGAAATGTTGAATACTCATACTCTTCCTTTTTCAATATTATTGA
AGCATTTATCAGGGTTATTGTCTCATGAGCGGATACATATTTGAATGTAT
TTAGAAAAATAAACAAATAGGGGTTCCGCGCACATTTCCCCGAAAAGTGC
CACCTGACGCGCCCTGTAGCGGGATCCATTTTATTTAATATACTAAATAA
TAAAAAAGTTAAAAAATGATCATTGGATAAATTTTTTATAATTATAAATA
AAGATAATAATTTTTTTTTTAACAAAACTAAAAATAAAAATAATAAAATA

ATTGTTAAAATAGGTTTTTTTTTTTTTTTTTTTTTTTTTAATAAATGGTAT
TTATTAATTTATTTGTTGTGTGTGTTTTTTTTTTTATAATATTTTTTTTT
TTAGCATTGAATTAAGAAGAAATCAAATTGATGCGGCCGCTCAGAAGAAC
TCGTCAAGAAGGCGATAGAAGGCGATGCGCTGCGAATCGGGAGCGGCGAT
ACCGTAAAGCACGAGGAAGCGGTCAGCCCATTCGCCGCCAAGCTCTTCAG
CAATATCACGGGTAGCCAACGCTATGTCCTGATAGCGGTCCGCCACACCC
AGCCGTCCACAGTCGATGAATCCAGAAAAGCGGCCATTTTCCACCATGAT
ATTCGGCAAGCAGGCATCGCCATGGGTCACGACGAGATCCTCGCCGTCGG
GCATGCGCGCCTTGAGCCTGGCGAACAGTTCGGCTGGCGCGAGCCCCTGA
TGCTCTTCGTCCAGATCATCCTGATCGACAAGACCGGCTTCCATCCGAGT
ACGTGCTCGCTCGATGCGATGTTTCGCTTGGTGGTCGAATGGGCAGGTAG
CCGGATCAAGCGTATGCAGCCGCCGCATTGCATCAGCCATGATGGATACT
TTCTCGGCAGGAGCAAGGTGAGATGACAGGAGATCCTGCCCCGGCACTTC
GCCCAATAGCAGCCAGTCCCTTCCCGCTTCAGTGACAACGTCGAGCACAG
CTGCGCAAGGAACGCCCGTCGTGGCCAGCCACGATAGCCGCGCTGCCTCG
TCCTGCAGTTCATTCAGGGCACCGGACAGGTCGGTCTTGACAAAAAGAAC
CGGGCGCCCCTGCGCTGACAGCCGGAACACGGCGGCATCAGAGCAGCCGA
TTGTCTGTTGTGCCCAGTCATAGCCGAATAGCCTCTCCACCCAAGCGGCC
GGAGAACCTGCGTGCAATCCATCTTGTTCAATCATGCGAAACGATCCAGC
TTGAACATCTTCACCATCCATTTTTTGCTAGCTGTGAAATTAGTTTAAAA
TACAAATAAAGAGTTATAATAATATACAGTTGAATAAAAAAAAAAAAAAT
GAATTGGAAAATTTATTTTTATATGAAGAAAAAAAAATTTTGAAAAAAAA
AAAAAAATTAAAAAAAAAAAAAAAAAAAAAAAAAATTTAAAATTATTCCAC
TGTGGGGGGCCCCAAATTTTATTTAAAAAAAAAAAAAAAAATGGGTCCCTT
TTGGGGGGTTGGAAAAAAAAAAAAAAAAAAAAAAAAAAAATTGAAAATATA
ATGTTAGTCATATGATTAATCATT

pPI159 – mNeon (N)

CTCGAGACTAGAGCTAGATAAAAAAAATTTTTATTTATTTTTATTTATTT
TGAATTAAATAGATTACAAATTAATTAATCCCATCAAATCTTTAAAAAAA
AATGGTTTAAAAAAACTTGGGTTGGTTAATTATTATTTGAAAATTTTAAA
ACCCAAATTAAAAAAAAAAAAATGGGATTCAAAAATTTTTTTTTTTTTTT
TTTTTTTTTTTTTTTTTTTTTTTCAGATTGCATAAAAAGATTTTTTTTT
TTTTTTTTCTTATTTCTTAAAACAAATAAATTAAATTAAATAAAAAATAA
AAATCAGATCCAAAAAATGGTGAGTAAAGGTGAAGAAGATAATATGGCAT
CGTTACCAGCTACACATGAGTTACATATATTCGGTAGCATTAATGGTGTT
GATTTTGATATGGTGGGACAAGGTACCGGTAATCCTAATGATGGTTACGA
AGAACTAAATTTAAAATCGACTAAAGGTGACTTACAATTTTCTCCATGGA
TTTTAGTGCCACATATAGGGTATGGTTTTCATCAATACTTACCATATCCA
GATGGTATGTCACCATTTCAAGCTGCAATGGTTGATGGATCAGGTTATCA
AGTTCATAGAACAATGCAATTTGAAGATGGTGCTTCATTAACTGTTAATT
ATAGATACACATATGAAGGCTCACATATTAAAGGTGAAGCTCAAGTTAAA
GGTACTGGTTTCCCAGCCGATGGCCCAGTTATGACAAATAGTTTAACAGC
AGCAGATTGGTGTAGATCCAAAAAAACTTATCCAAATGATAAAACAATTA
TTTCAACTTTTAAATGGTCATATACAACCGGTAATGGTAAACGTTATCGT
TCAACAGCCCGTACAACATATACTTTTGCTAAACCAATGGCAGCTAATTA
TTTAAAAAATCAACCAATGTATGTTTTTCGTAAAACAGAGTTAAAACATT
CAAAAACAGAACTTAATTTTAAAGAATGGCAAAAAGCATTTACAGACGTT
ATGGGTATGGATGAACTTTATAAGGGTGGTTCAGGAGGTAGTAGATCTTC

AACTAGTAGTTAAATAAATAAATTATTTAATAAATAATAAAAAAACAAAT
TGTTGTAATAATCTAATATTTTCTTTTTTTTTTAATTTTTTTTTTTTAAA
TCTTAATAATTATTAAGTTATTTTAATTTTTTTTTTTTTTTTTTTTTTTT
TTTTTTTTTTTTTTCTATCAAAAAAATCAAATATATTTAAAAAATTTATT
ATTTACAGATACATTTTGAATGGTGAAGATAAATATATGCATTAGATGTA
AAACAGCCAAAGAGTATGAAAATCAAAAAGATAAAGCTTATCGATTTCGA
AAAAGTAAATAGCAATTATTACAAAATTCAATCCGAATCTACCCAAATAA
ATTCCAATGAAATTGCCGATTTAAAAAAGTTTATTAAAGAAGAAGTCAAT
AAAACTTCTTCCAAAATTGATTTCTTTTTAGTTTCTTCAACAGATGCCCT
TTCAAATCCAGAAAATTATTCTCTCTTAGAAGTAAAGTGTATTAATTGTC
ATTCTTTGTGTCAAGGAAAAAATTTATATATTTCATGTACAAGAGATGGA
TGTCAAAACAATATTTGCTATAATTGTTTAGGAATAAACATAAACATATA
TAATGTTGTTATTAATTCTAAACTTTGCCCTCCATGTTTCAATGATTCGG
TAATCAACAAGAAGTGTGCCATGTGTAGTAAGAACGGAACTAAATGTAAT
TTGAACCAAGAATGTAAACTTCATCTTTGTGCACAGTGTTCTAAAAAGTG
TCTATACATTCTGAGAGTCAAAACTAATTAAATAAAATATAAACTTAATT
TCTAAATAAACTCATTTAAAAATATTTAAATAATATGAATTTATAACTGT
AATTATTGTATTAAAAAATTATATAATTATTTAATGTTAAAAATGTATTA
AAATAATTATAAAAAAATATAACAAAAATTTTCGTAAAAATAATTTGTAA
AAAAGCTATTAAAAATATTATGAAAAAAAAATTAAAAAAATTATTAAATT
GTTTTTGTAATTAAGCTATTAAAATAATTATAAAAAAAAAATTTTTAAAA
TTTTAAAAATATTTTTTGTAAAAAAGTATTAAAATAATTATGAAAAAAAA
ATTTTCTAAAAAATTAAAAAAAAAATTAAAATATATTTTATGTTAAAAAC
GTATTAAAATAACTATTAAAAAAATTATATTTAAAAAAGTATTAACTTTT
TTTTAGGTGTGGTTGTGGGGTGGGGTTTAATATATTATAATAAAAAATTA
TTTTTTGTTCATTTATTATTTTCATTGTATATAATGTACTCAACAACGTT
ATTATTTTTTCTTTTTTTTTTTATTGTATCAAAATCTTCTGTTCTTCAAA
ATGATCAGATTGAAGTAAAATATTTTCAACTTCTTATTGTTATGTATCAA
AAAGAAAACTGTGTTGAAAAGTCAATGACAGGCGCCGTAATTTATGATGA
ATGTAATATTCATGGAAGAGTTGAAACAAATAGTACTCATGCGCTTTTTT
ATGATGACATTGAAACAAATAATTCAAGATGTAACAATTTTCGTAATTTA
ACAAACTTAATTAAACTTAATGAATGTATTAATGACGAGTTTGGAGAGTC
TATTCTTTATAAAGAATATAATGAAACTGATGATGGTTATTTGTTTAGAG
TGGAAGACAGCTTTGTTGAAATTACTTCTCTTTCAATGGATTGTACAAAA
AATAGTAAAACAATTATTGAAAAATTCAACATTTGTTCAAAATTTGAAAA
TGTATATCATATTACAAACATTACACAAGAGAAATCCAATAGATTTACAT
GTACAGATCCATTGTGCCACTATTGTAAGAATGAAAACATTCAAAACAAT
CTTGATTTTAAAACAACAAAGTGTACTCCAAAGTATGGTGCATCTGATTC
TGAATTTTTATCAACAATTTACAATCCAAAGCTCGATGGCTCAAATAACG
GTATGGAAAAGTCAGTAACTCAAGAAAAAAACATTTCAAATAATTTAAAA
ATTAATATATATTTAATTTTCTTTTTAATTATTTTTTTAATTAAATAAAG
TTTTATTATTTTTTAAGAGTAATTATTGCTCTTTTTTCATTTGAAACACC
AGAAGCTAAACGTAATTGTTGTTGACTGAAATTTTTTATTTTTTTTGGGG
TAATAGGATTTCCTTTTTTATGAAGATTAATATCTTTGACTCGTGAAACA
TTCTTTTTAACTTTTGTTTTTTCTGTTGGTTTATCATTTGTTTTTTCACT
AATTTCAATACCATCTTGACGTTCATTCATAACTTCATCTTTTTTTTTTC
CTGTTTCTGTATCTTCTTCTATTTTTTTTTCTTTATCTTTTTCTTTATCT
TCTTCTTGTTCTTCCTCTTCTTTTTCTTCTTCTGATACTGCAGGTGTTTC
TTCTTCTTCTTCTTCCGATATTGTCGGTTTTTCTACTTCTTCTTCTTGTT
CTTCTTCTTCTTCTTCTTCTTCTTCTTCTTCTTCTTCTTCTTCTTCTTCT

TCCGGTAATTTATTAATTATATTTCTTTTTTTATATGAATTACGTTTGGT
TTGTGCAGTAATTTCCTTACATAGAGTGCAGCTTTCAAGAAAAATTTCAA
TTTCTTCGTTTGTTGCATAATAACCACTGTCTTTGATATGATTAAACATT
TTTGATTTTCTTAAATGCTTTCCTTCTTTAATATGAAAATTATCGAATTC
TAATTCATTAAGAACAATAAGCTCCCCTAATTTAAAAAATTAGTTAAAAT
AAATTAAAATGAACATGTATAAAGATGGATTTTACCATTTTTTGAAATTC
TAAATAACTTTTCTTCATCTCCAATCTTTTTGACTGAAAAACGATTTTTA
ATTGAAGTTATTGTTCTGTGAGTGTTTTGAATCGCCCATTTCTCTAAATC
AGTTTGAGATAGTGTTTTATAATCTGAATTGTTATACACAACTTTTGCTC
TATTAACCAAATATTTAAAGATTTCATCATCAACTGAATATTTTGACTTT
ACGATTCTTGTCCAAAAAACAATTTCTACTACTATCATTTTTTATTTATA
AAATAATTTAAATACAAAAATGAATTTTTTTTTTTTTAAAAAAAAAAAAA
TTTGAAAAAAAAAAAAAAAAAATTTTAAAAAAAAAAAAAAAAAAAAAAAA
AAAAAAAAAATCAAATAAAAAGTAAAAAATAAAAACCGAAAAACATTCAT
TGTAATTTCAAATGTCGAGGCCGGCAGAGGCGGTTTGCGTATTGGGCGCT
CTTCCGCTTCCTCGCTCACTGACTCGCTGCGCTCGGTCGTTCGGCTGCGG
CGAGCGGTATCAGCTCACTCAAAGGCGGTAATACGGTTATCCACAGAATC
AGGGGATAACGCAGGAAAGAACATGTGAGCAAAAGGCCAGCAAAAGGCCA
GGAACCGTAAAAAGGCCGCGTTGCTGGCGTTTTTCCATAGGCTCCGCCCC
CCTGACGAGCATCACAAAAATCGACGCTCAAGTCAGAGGTGGCGAAACCC
GACAGGACTATAAAGATACCAGGCGTTTCCCCCTGGAAGCTCCCTCGTGC
GCTCTCCTGTTCCGACCCTGCCGCTTACCGGATACCTGTCCGCCTTTCTC
CCTTCGGGAAGCGTGGCGCTTTCTCATAGCTCACGCTGTAGGTATCTCAG
TTCGGTGTAGGTCGTTCGCTCCAAGCTGGGCTGTGTGCACGAACCCCCCG
TTCAGCCCGACCGCTGCGCCTTATCCGGTAACTATCGTCTTGAGTCCAAC
CCGGTAAGACACGACTTATCGCCACTGGCAGCAGCCACTGGTAACAGGAT
TAGCAGAGCGAGGTATGTAGGCGGTGCTACAGAGTTCTTGAAGTGGTGGC
CTAACTACGGCTACACTAGAAGGACAGTATTTGGTATCTGCGCTCTGCTG
AAGCCAGTTACCTTCGGAAAAAGAGTTGGTAGCTCTTGATCCGGCAAACA
AACCACCGCTGGTAGCGGTGGTTTTTTTGTTTGCAAGCAGCAGATTACGC
GCAGAAAAAAAGGATCTCAAGAAGATCCTTTGATCTTTTCTACGGGGTCT
GACGCTCAGTGGAACGAAAACTCACGTTAAGGGATTTTGGTCATGAGATT
ATCAAAAAGGATCTTCACCTAGATCCTTTTAAATTAAAAATGAAGTTTTA
AATCAATCTAAAGTATATATGAGTAAACTTGGTCTGACAGTTACCAATGC
TTAATCAGTGAGGCACCTATCTCAGCGATCTGTCTATTTCGTTCATCCAT
AGTTGCCTGACTCCCCGTCGTGTAGATAACTACGATACGGGAGGGCTTAC
CATCTGGCCCCAGTGCTGCAATGATACCGCGAGACCCACGCTCACCGGCT
CCAGATTTATCAGCAATAAACCAGCCAGCCGGAAGGGCCGAGCGCAGAAG
TGGTCCTGCAACTTTATCCGCCTCCATCCAGTCTATTAATTGTTGCCGGG
AAGCTAGAGTAAGTAGTTCGCCAGTTAATAGTTTGCGCAACGTTGTTGCC
ATTGCTACAGGCATCGTGGTGTCACGCTCGTCGTTTGGTATGGCTTCATT
CAGCTCCGGTTCCCAACGATCAAGGCGAGTTACATGATCCCCCATGTTGT
GCAAAAAAGCGGTTAGCTCCTTCGGTCCTCCGATCGTTGTCAGAAGTAAG
TTGGCCGCAGTGTTATCACTCATGGTTATGGCAGCACTGCATAATTCTCT
TACTGTCATGCCATCCGTAAGATGCTTTTCTGTGACTGGTGAGTACTCAA
CCAAGTCATTCTGAGAATAGTGTATGCGGCGACCGAGTTGCTCTTGCCCG
GCGTCAATACGGGATAATACCGCGCCACATAGCAGAACTTTAAAAGTGCT
CATCATTGGAAAACGTTCTTCGGGGCGAAAACTCTCAAGGATCTTACCGC
TGTTGAGATCCAGTTCGATGTAACCCACTCGTGCACCCAACTGATCTTCA
GCATCTTTTACTTTCACCAGCGTTTCTGGGTGAGCAAAAACAGGAAGGCA

AAATGCCGCAAAAAAGGGAATAAGGGCGACACGGAAATGTTGAATACTCA
TACTCTTCCTTTTTCAATATTATTGAAGCATTTATCAGGGTTATTGTCTC
ATGAGCGGATACATATTTGAATGTATTTAGAAAAATAAACAAATAGGGGT
TCCGCGCACATTTCCCCGAAAAGTGCCACCTGACGCGCCCTGTAGCGGGA
TCCATTTTATTTAATATACTAAATAATAAAAAAGTTAAAAAATGATCATT
GGATAAATTTTTTATAATTATAAATAAAGATAATAATTTTTTTTTTAACA
AAACTAAAAATAAAAATAATAAAATAATTGTTAAAATAGGTTTTTTTTTT
TTTTTTTTTTTTTTAATAAATGGTATTTATTAATTTATTTGTTGTGTGTG
TTTTTTTTTTTTATAATATTTTTTTTTTAGCATTGAATTAAGAAGAAATC
AAATTGATGCGGCCGCTCAGAAGAACTCGTCAAGAAGGCGATAGAAGGCG
ATGCGCTGCGAATCGGGAGCGGCGATACCGTAAAGCACGAGGAAGCGGTC
AGCCCATTCGCCGCCAAGCTCTTCAGCAATATCACGGGTAGCCAACGCTA
TGTCCTGATAGCGGTCCGCCACACCCAGCCGTCCACAGTCGATGAATCCA
GAAAAGCGGCCATTTTCCACCATGATATTCGGCAAGCAGGCATCGCCATG
GGTCACGACGAGATCCTCGCCGTCGGGCATGCGCGCCTTGAGCCTGGCGA
ACAGTTCGGCTGGCGCGAGCCCCTGATGCTCTTCGTCCAGATCATCCTGA
TCGACAAGACCGGCTTCCATCCGAGTACGTGCTCGCTCGATGCGATGTTT
CGCTTGGTGGTCGAATGGGCAGGTAGCCGGATCAAGCGTATGCAGCCGCC
GCATTGCATCAGCCATGATGGATACTTTCTCGGCAGGAGCAAGGTGAGAT
GACAGGAGATCCTGCCCCGGCACTTCGCCCAATAGCAGCCAGTCCCTTCC
CGCTTCAGTGACAACGTCGAGCACAGCTGCGCAAGGAACGCCCGTCGTGG
CCAGCCACGATAGCCGCGCTGCCTCGTCCTGCAGTTCATTCAGGGCACCG
GACAGGTCGGTCTTGACAAAAAGAACCGGGCGCCCCTGCGCTGACAGCCG
GAACACGGCGGCATCAGAGCAGCCGATTGTCTGTTGTGCCCAGTCATAGC
CGAATAGCCTCTCCACCCAAGCGGCCGGAGAACCTGCGTGCAATCCATCT
TGTTCAATCATGCGAAACGATCCAGCTTGAACATCTTCACCATCCATTTT
TTGCTAGCTGTGAAATTAGTTTAAAATACAAATAAAGAGTTATAATAATA
TACAGTTGAATAAAAAAAAAAAAAATGAATTGGAAAATTTATTTTTATAT
GAAGAAAAAAAAAATTTTGAAAAAAAAAAAAAAATTAAAAAAAAAAAAAA
AAAAAAAAAATTTAAAATTATTCCACTGTGGGGGGCCCCAAATTTTATTT
AAAAAAAAAAAAAAAAATGGGTCCCTTTTGGGGGGTTGGAAAAAAAAAAAA
AAAAAAAAAAAAAAATTGAAAATATAATGTTAGTCATATGATTAATCATT

pPI437 – mScarlet (N)

CTCGAGACTAGAGCTAGATAAAAAAAAATTTTTATTTATTTTTATTTATTT
TGAATTAAATAGATTACAAATTAATTAATCCCATCAAATCTTTAAAAAAA
AATGGTTTAAAAAAACTTGGGTTGGTTAATTATTATTTGAAAATTTTAAA
ACCCAAATTAAAAAAAAAAAAATGGGATTCAAAAATTTTTTTTTTTTTTT
TTTTTTTTTTTTTTTTTTTTTTCAGATTGCATAAAAAGATTTTTTTTTT
TTTTTTTTCTTATTTCTTAAAACAAATAAATTAAATTAAATAAAAAATAA
AAATCAGATCCAAAAAATGGTTTCAAAAGGTGAAGCCGTTATTAAAGAAT
TTATGAGATTCAAGGTTCACATGGAAGGAAGTATGAACGGTCATGAATTT
GAGATTGAAGGAGAAGGTGAAGGTAGACCATATGAAGGCACCCAAACAGC
TAAATTAAAAGTAACTAAAGGTGGTCCATTACCATTTAGTTGGGATATTT
TATCTCCACAATTTATGTATGGTTCACGTGCTTTCACAAAACATCCAGCA
GATATTCCAGATTATTATAAACAATCATTTCCAGAAGGTTTTAAATGGGA
ACGTGTCATGAACTTTGAAGATGGTGGAGCAGTTACAGTCACACAAGATA
CCTCATTAGAAGATGGTACATTAATATATAAAGTTAAATTACGTGGTACT
AATTTTCCACCAGACGGTCCAGTAATGCAAAAAAAAACAATGGGCTGGGA

AGCTAGTACAGAACGTTTATATCCTGAAGATGGTGTCCTTAAAGGCGATA
TAAAAATGGCCTTGAGATTAAAGGATGGTGGTAGGTATTTAGCAGATTTC
AAAACCACTTATAAAGCAAAAAAACCAGTTCAAATGCCAGGTGCATATAA
TGTTGATAGAAAACTTGATATTACCAGTCATAATGAAGATTACACAGTTG
TCGAACAATACGAACGTTCTGAAGGTCGTCATAGCACTGGTGGTATGGAT
GAATTATACAAAGGTGGTTCAGGAGGTAGTAGATCTTCAACTAGTTAAAT
AAATAAATTATTTAATAAATAATAAAAAAACAAATTGTTGTAATAATCTA
ATATTTTCTTTTTTTTTTAATTTTTTTTTTTTAAATCTTAATAATTATTA
AGTTATTTTAATTTTTTTTTTTTTTTTTTTTTTTTTTTTTTTTTTTTTTC
TATCAAAAAAATCAAATATATTTAAAAAATTTATTATTTACAGATACATT
TTGAATGGTGAAGATAAATATATGCATTAGATGTAAAACAGCCAAAGAGT
ATGAAAATCAAAAAGATAAAGCTTATCGATTTCGAAAAAGTAAATAGCAA
TTATTACAAAATTCAATCCGAATCTACCCAAATAAATTCCAATGAAATTG
CCGATTTAAAAAAGTTTATTAAAGAAGAAGTCAATAAAACTTCTTCCAAA
ATTGATTTCTTTTTAGTTTCTTCAACAGATGCCCTTTCAAATCCAGAAAA
TTATTCTCTCTTAGAAGTAAAGTGTATTAATTGTCATTCTTTGTGTCAAG
GAAAAAATTTATATATTTCATGTACAAGAGATGGATGTCAAAACAATATT
TGCTATAATTGTTTAGGAATAAACATAAACATATATAATGTTGTTATTAA
TTCTAAACTTTGCCCTCCATGTTTCAATGATTCGGTAATCAACAAGAAGT
GTGCCATGTGTAGTAAGAACGGAACTAAATGTAATTTGAACCAAGAATGT
AAACTTCATCTTTGTGCACAGTGTTCTAAAAAGTGTCTATACATTCTGAG
AGTCAAAACTAATTAAATAAAATATAAACTTAATTTCTAAATAAACTCAT
TTAAAAATATTTAAATAATATGAATTTATAACTGTAATTATTGTATTAAA
AAATTATATAATTATTTAATGTTAAAAATGTATTAAAATAATTATAAAAA
AATATAACAAAAATTTTCGTAAAAATAATTTGTAAAAAAGCTATTAAAAA
TATTATGAAAAAAAAAATTAAAAAAATTATTAAATTGTTTTTGTAATTAAG
CTATTAAAATAATTATAAAAAAAAAAATTTTTAAAATTTTAAAAATATTTT
TTGTAAAAAAGTATTAAAATAATTATGAAAAAAAAAATTTTCTAAAAAATT
AAAAAAAAAATTAAAATATATTTTATGTTAAAAACGTATTAAAATAACTA
TTAAAAAAATTATATTTAAAAAAGTATTAACTTTTTTTTAGGTGTGGTTG
TGGGGTGGGGTTTAATATATTATAATAAAAAATTATTTTTTGTTCATTTA
TTATTTTCATTGTATATAATGTACTCAACAACGTTATTATTTTTTCTTTT
TTTTTTTATTGTATCAAAATCTTCTGTTCTTCAAAATGATCAGATTGAAG
TAAAATATTTTCAACTTCTTATTGTTATGTATCAAAAAGAAAACTGTGTT
GAAAAGTCAATGACAGGCGCCGTAATTTATGATGAATGTAATATTCATGG
AAGAGTTGAAACAAATAGTACTCATGCGCTTTTTTATGATGACATTGAAA
CAAATAATTCAAGATGTAACAATTTTCGTAATTTAACAAACTTAATTAAA
CTTAATGAATGTATTAATGACGAGTTTGGAGAGTCTATTCTTTATAAAGA
ATATAATGAAACTGATGATGGTTATTTGTTTAGAGTGGAAGACAGCTTTG
TTGAAATTACTTCTCTTTCAATGGATTGTACAAAAAATAGTAAAACAATT
ATTGAAAAATTCAACATTTGTTCAAAATTTGAAAATGTATATCATATTAC
AAACATTACACAAGAGAAATCCAATAGATTTACATGTACAGATCCATTGT
GCCACTATTGTAAGAATGAAAACATTCAAAACAATCTTGATTTTAAAACA
ACAAAGTGTACTCCAAAGTATGGTGCATCTGATTCTGAATTTTTATCAAC
AATTTACAATCCAAAGCTCGATGGCTCAAATAACGGTATGGAAAAGTCAG
TAACTCAAGAAAAAAACATTTCAAATAATTTAAAAATTAATATATATTTA
ATTTTCTTTTTAATTATTTTTTTAATTAAATAAAGTTTTATTATTTTTTA
AGAGTAATTATTGCTCTTTTTTCATTTGAAACACCAGAAGCTAAACGTAA
TTGTTGTTGACTGAAATTTTTTATTTTTTTTGGGGTAATAGGATTTCCTT
TTTTATGAAGATTAATATCTTTGACTCGTGAAACATTCTTTTTAACTTTT

GTTTTTTCTGTTGGTTTATCATTTGTTTTTTCACTAATTTCAATACCATC
TTGACGTTCATTCATAACTTCATCTTTTTTTTTTCCTGTTTCTGTATCTT
CTTCTATTTTTTTTTCTTTATCTTTTTCTTTATCTTCTTCTTGTTCTTCC
TCTTCTTTTTCTTCTTCTGATACTGCAGGTGTTTCTTCTTCTTCTTCTTC
CGATATTGTCGGTTTTTCTACTTCTTCTTCTTGTTCTTCTTCTTCTTCTT
CTTCTTCTTCTTCTTCTTCTTCTTCTTCTTCTTCTTCCGGTAATTTATTA
ATTATATTTCTTTTTTTATATGAATTACGTTTGGTTTGTGCAGTAATTTC
CTTACATAGAGTGCAGCTTTCAAGAAAAATTTCAATTTCTTCGTTTGTTG
CATAATAACCACTGTCTTTGATATGATTAAACATTTTTGATTTTCTTAAA
TGCTTTCCTTCTTTAATATGAAAATTATCGAATTCTAATTCATTAAGAAC
AATAAGCTCCCCTAATTTAAAAAATTAGTTAAAATAAATTAAAATGAACA
TGTATAAAGATGGATTTTACCATTTTTTGAAATTCTAAATAACTTTTCTT
CATCTCCAATCTTTTTGACTGAAAAACGATTTTTAATTGAAGTTATTGTT
CTGTGAGTGTTTTGAATCGCCCATTTCTCTAAATCAGTTTGAGATAGTGT
TTTATAATCTGAATTGTTATACACAACTTTTGCTCTATTAACCAAATATT
TAAAGATTTCATCATCAACTGAATATTTTGACTTTACGATTCTTGTCCAA
AAAACAATTTCTACTACTATCATTTTTTATTTATAAAATAATTTAAATAC
AAAAATGAATTTTTTTTTTTTTAAAAAAAAAAAAAATTTGAAAAAAAAAAA
AAAAAAATTTTAAAAAAAAAAAAAAAAAAAAAAAAAAAAAAAAAAATCAAA
TAAAAAGTAAAAAATAAAAACCGAAAAACATTCATTGTAATTTCAAATGT
CGAGGCCGGCAGAGGCGGTTTGCGTATTGGGCGCTCTTCCGCTTCCTCGC
TCACTGACTCGCTGCGCTCGGTCGTTCGGCTGCGGCGAGCGGTATCAGCT
CACTCAAAGGCGGTAATACGGTTATCCACAGAATCAGGGGATAACGCAGG
AAAGAACATGTGAGCAAAAGGCCAGCAAAAGGCCAGGAACCGTAAAAAGG
CCGCGTTGCTGGCGTTTTTCCATAGGCTCCGCCCCCCTGACGAGCATCAC
AAAAATCGACGCTCAAGTCAGAGGTGGCGAAACCCGACAGGACTATAAAG
ATACCAGGCGTTTCCCCCTGGAAGCTCCCTCGTGCGCTCTCCTGTTCCGA
CCCTGCCGCTTACCGGATACCTGTCCGCCTTTCTCCCTTCGGGAAGCGTG
GCGCTTTCTCATAGCTCACGCTGTAGGTATCTCAGTTCGGTGTAGGTCGT
TCGCTCCAAGCTGGGCTGTGTGCACGAACCCCCCGTTCAGCCCGACCGCT
GCGCCTTATCCGGTAACTATCGTCTTGAGTCCAACCCGGTAAGACACGAC
TTATCGCCACTGGCAGCAGCCACTGGTAACAGGATTAGCAGAGCGAGGTA
TGTAGGCGGTGCTACAGAGTTCTTGAAGTGGTGGCCTAACTACGGCTACA
CTAGAAGGACAGTATTTGGTATCTGCGCTCTGCTGAAGCCAGTTACCTTC
GGAAAAAGAGTTGGTAGCTCTTGATCCGGCAAACAAACCACCGCTGGTAG
CGGTGGTTTTTTTGTTTGCAAGCAGCAGATTACGCGCAGAAAAAAAGGAT
CTCAAGAAGATCCTTTGATCTTTTCTACGGGGTCTGACGCTCAGTGGAAC
GAAAACTCACGTTAAGGGATTTTGGTCATGAGATTATCAAAAAGGATCTT
CACCTAGATCCTTTTAAATTAAAAATGAAGTTTTAAATCAATCTAAAGTA
TATATGAGTAAACTTGGTCTGACAGTTACCAATGCTTAATCAGTGAGGCA
CCTATCTCAGCGATCTGTCTATTTCGTTCATCCATAGTTGCCTGACTCCC
CGTCGTGTAGATAACTACGATACGGGAGGGCTTACCATCTGGCCCCAGTG
CTGCAATGATACCGCGAGACCCACGCTCACCGGCTCCAGATTTATCAGCA
ATAAACCAGCCAGCCGGAAGGGCCGAGCGCAGAAGTGGTCCTGCAACTTT
ATCCGCCTCCATCCAGTCTATTAATTGTTGCCGGGAAGCTAGAGTAAGTA
GTTCGCCAGTTAATAGTTTGCGCAACGTTGTTGCCATTGCTACAGGCATC
GTGGTGTCACGCTCGTCGTTTGGTATGGCTTCATTCAGCTCCGGTTCCCA
ACGATCAAGGCGAGTTACATGATCCCCCATGTTGTGCAAAAAAGCGGTTA
GCTCCTTCGGTCCTCCGATCGTTGTCAGAAGTAAGTTGGCCGCAGTGTTA
TCACTCATGGTTATGGCAGCACTGCATAATTCTCTTACTGTCATGCCATC

CGTAAGATGCTTTTCTGTGACTGGTGAGTACTCAACCAAGTCATTCTGAG
AATAGTGTATGCGGCGACCGAGTTGCTCTTGCCCGGCGTCAATACGGGAT
AATACCGCGCCACATAGCAGAACTTTAAAAGTGCTCATCATTGGAAAACG
TTCTTCGGGGCGAAAACTCTCAAGGATCTTACCGCTGTTGAGATCCAGTT
CGATGTAACCCACTCGTGCACCCAACTGATCTTCAGCATCTTTTACTTTC
ACCAGCGTTTCTGGGTGAGCAAAAACAGGAAGGCAAAATGCCGCAAAAAA
GGGAATAAGGGCGACACGGAAATGTTGAATACTCATACTCTTCCTTTTTC
AATATTATTGAAGCATTTATCAGGGTTATTGTCTCATGAGCGGATACATA
TTTGAATGTATTTAGAAAAATAAACAAATAGGGGTTCCGCGCACATTTCC
CCGAAAAGTGCCACCTGACGCGCCCTGTAGCGGGATCCATTTTATTTAAT
ATACTAAATAATAAAAAAGTTAAAAAATGATCATTGGATAAATTTTTTAT
AATTATAAATAAAGATAATAATTTTTTTTTTAACAAAACTAAAAATAAAA
ATAATAAAATAATTGTTAAAATAGGTTTTTTTTTTTTTTTTTTTTTTTTA
ATAAATGGTATTTATTAATTTATTTGTTGTGTGTGTTTTTTTTTTTATAA
TATTTTTTTTTTTTAGCATTGAATTAAGAAGAAATCAAATTGATGCGGCCG
CTCAGAAGAACTCGTCAAGAAGGCGATAGAAGGCGATGCGCTGCGAATCG
GGAGCGGCGATACCGTAAAGCACGAGGAAGCGGTCAGCCCATTCGCCGCC
AAGCTCTTCAGCAATATCACGGGTAGCCAACGCTATGTCCTGATAGCGGT
CCGCCACACCCAGCCGTCCACAGTCGATGAATCCAGAAAAGCGGCCATTT
TCCACCATGATATTCGGCAAGCAGGCATCGCCATGGGTCACGACGAGATC
CTCGCCGTCGGGCATGCGCGCCTTGAGCCTGGCGAACAGTTCGGCTGGCG
CGAGCCCCTGATGCTCTTCGTCCAGATCATCCTGATCGACAAGACCGGCT
TCCATCCGAGTACGTGCTCGCTCGATGCGATGTTTCGCTTGGTGGTCGAA
TGGGCAGGTAGCCGGATCAAGCGTATGCAGCCGCCGCATTGCATCAGCCA
TGATGGATACTTTCTCGGCAGGAGCAAGGTGAGATGACAGGAGATCCTGC
CCCGGCACTTCGCCCAATAGCAGCCAGTCCCTTCCCGCTTCAGTGACAAC
GTCGAGCACAGCTGCGCAAGGAACGCCCGTCGTGGCCAGCCACGATAGCC
GCGCTGCCTCGTCCTGCAGTTCATTCAGGGCACCGGACAGGTCGGTCTTG
ACAAAAAGAACCGGGCGCCCCTGCGCTGACAGCCGGAACACGGCGGCATC
AGAGCAGCCGATTGTCTGTTGTGCCCAGTCATAGCCGAATAGCCTCTCCA
CCCAAGCGGCCGGAGAACCTGCGTGCAATCCATCTTGTTCAATCATGCGA
AACGATCCAGCTTGAACATCTTCACCATCCATTTTTTGCTAGCTGTGAAA
TTAGTTTAAAATACAAATAAAGAGTTATAATAATATACAGTTGAATAAAA
AAAAAAAAAATGAATTGGAAAATTTATTTTTATATGAAGAAAAAAAAATT
TTGAAAAAAAAAAAAAAAATTAAAAAAAAAAAAAAAAAAAAAAAAATTTAA
AATTATTCCACTGTGGGGGGCCCCAAATTTTATTTAAAAAAAAAAAAAAA
ATGGGTCCCTTTTGGGGGGTTGGAAAAAAAAAAAAAAAAAAAAAAAAAAA
TTGAAAATATAATGTTAGTCATATGATTAATCATT

pPI459 – mScarlet (C)

CTCGAGACTAGAGCTAGATAAAAAAAATTTTTATTTATTTTTATTTATTT
TGAATTAAATAGATTACAAATTAATTAATCCCATCAAATCTTTAAAAAAA
AATGGTTTAAAAAAACTTGGGTTGGTTAATTATTATTTGAAAATTTTAAA
ACCCAAATTAAAAAAAAAAAAAATGGGATTCAAAAATTTTTTTTTTTTTT
TTTTTTTTTTTTTTTTTTTTTTTCAGATTGCATAAAAAGATTTTTTTTTT
TTTTTTTTCTTATTTCTTAAAACAAATAAATTAAATTAAATAAAAAATAA
AAATCAGATCTTCAACTAGTGGTGGTTCAGGAGGTAGTGTTTCAAAAGGT
GAAGCCGTTATTAAAGAATTTATGAGATTCAAGGTTCACATGGAAGGAAG
TATGAACGGTCATGAATTTGAGATTGAAGGAGAAGGTGAAGGTAGACCAT

ATGAAGGCACCCAAACAGCTAAATTAAAAGTAACTAAAGGTGGTCCATTA
CCATTTAGTTGGGATATTTTATCTCCACAATTTATGTATGGTTCACGTGC
TTTCACAAAACATCCAGCAGATATTCCAGATTATTATAAACAATCATTTC
CAGAAGGTTTTAAATGGGAACGTGTCATGAACTTTGAAGATGGTGGAGCA
GTTACAGTCACACAAGATACCTCATTAGAAGATGGTACATTAATATATAA
AGTTAAATTACGTGGTACTAATTTTCCACCAGACGGTCCAGTAATGCAAA
AAAAAACAATGGGCTGGGAAGCTAGTACAGAACGTTTATATCCTGAAGAT
GGTGTCCTTAAAGGCGATATAAAAATGGCCTTGAGATTAAAGGATGGTGG
TAGGTATTTAGCAGATTTCAAAACCACTTATAAAGCAAAAAAACCAGTTC
AAATGCCAGGTGCATATAATGTTGATAGAAAACTTGATATTACCAGTCAT
AATGAAGATTACACAGTTGTCGAACAATACGAACGTTCTGAAGGTCGTCA
TAGCACTGGTGGTATGGATGAATTATACAAATAAGCTAGTTAAATAAATA
AATTATTTAATAAATAATAAAAAAACAAATTGTTGTAATAATCTAATATT
TTCTTTTTTTTTTAATTTTTTTTTTTTAAATCTTAATAATTATTAAGTTA
TTTTAATTTTTTTTTTTTTTTTTTTTTTTTTTTTTTTTTTTTTTCTATCA
AAAAAATCAAATATATTTAAAAAATTTATTATTTACAGATACATTTTGAA
TGGTGAAGATAAATATATGCATTAGATGTAAAACAGCCAAAGAGTATGAA
AATCAAAAAGATAAAGCTTATCGATTTCGAAAAAGTAAATAGCAATTATT
ACAAAATTCAATCCGAATCTACCCAAATAAATTCCAATGAAATTGCCGAT
TTAAAAAAGTTTATTAAAGAAGAAGTCAATAAAACTTCTTCCAAAATTGA
TTTCTTTTTAGTTTCTTCAACAGATGCCCTTTCAAATCCAGAAAATTATT
CTCTCTTAGAAGTAAAGTGTATTAATTGTCATTCTTTGTGTCAAGGAAAA
AATTTATATATTTCATGTACAAGAGATGGATGTCAAAACAATATTTGCTA
TAATTGTTTAGGAATAAACATAAACATATATAATGTTGTTATTAATTCTA
AACTTTGCCCTCCATGTTTCAATGATTCGGTAATCAACAAGAAGTGTGCC
ATGTGTAGTAAGAACGGAACTAAATGTAATTTGAACCAAGAATGTAAACT
TCATCTTTGTGCACAGTGTTCTAAAAAGTGTCTATACATTCTGAGAGTCA
AAACTAATTAAATAAAATATAAACTTAATTTCTAAATAAACTCATTTAAA
AATATTTAAATAATATGAATTTATAACTGTAATTATTGTATTAAAAAATT
ATATAATTATTTAATGTTAAAAATGTATTAAAATAATTATAAAAAAATAT
AACAAAAATTTTCGTAAAAATAATTTGTAAAAAAGCTATTAAAAATATTA
TGAAAAAAAAATTAAAAAAATTATTAAATTGTTTTTGTAATTAAGCTATT
AAAATAATTATAAAAAAAAAATTTTTAAAATTTTAAAAATATTTTTTGTA
AAAAAGTATTAAAATAATTATGAAAAAAAAAATTTTCTAAAAAATTAAAAA
AAAAATTAAAATATATTTTATGTTAAAAACGTATTAAAATAACTATTAAA
AAAATTATATTTAAAAAAGTATTAACTTTTTTTTAGGTGTGGTTGTGGGG
TGGGGTTTAATATATTATAATAAAAAATTATTTTTTGTTCATTTATTATT
TTCATTGTATATAATGTACTCAACAACGTTATTATTTTTTCTTTTTTTTT
TTATTGTATCAAAATCTTCTGTTCTTCAAAATGATCAGATTGAAGTAAAA
TATTTTCAACTTCTTATTGTTATGTATCAAAAAGAAAACTGTGTTGAAAA
GTCAATGACAGGCGCCGTAATTTATGATGAATGTAATATTCATGGAAGAG
TTGAAACAAATAGTACTCATGCGCTTTTTTATGATGACATTGAAACAAAT
AATTCAAGATGTAACAATTTTCGTAATTTAACAAACTTAATTAAACTTAA
TGAATGTATTAATGACGAGTTTGGAGAGTCTATTCTTTATAAAGAATATA
ATGAAACTGATGATGGTTATTTGTTTAGAGTGGAAGACAGCTTTGTTGAA
ATTACTTCTCTTTCAATGGATTGTACAAAAAATAGTAAAACAATTATTGA
AAAATTCAACATTTGTTCAAAATTTGAAAATGTATATCATATTACAAACA
TTACACAAGAGAAATCCAATAGATTTACATGTACAGATCCATTGTGCCAC
TATTGTAAGAATGAAAACATTCAAAACAATCTTGATTTTAAAACAACAAA
GTGTACTCCAAAGTATGGTGCATCTGATTCTGAATTTTTATCAACAATTT

ACAATCCAAAGCTCGATGGCTCAAATAACGGTATGGAAAAGTCAGTAACT
CAAGAAAAAAACATTTCAAATAATTTAAAAATTAATATATATTTAATTTT
CTTTTTAATTATTTTTTTAATTAAATAAAGTTTTATTATTTTTTTAAGAGT
AATTATTGCTCTTTTTTCATTTGAAACACCAGAAGCTAAACGTAATTGTT
GTTGACTGAAATTTTTTATTTTTTTTGGGGTAATAGGATTTCCTTTTTTA
TGAAGATTAATATCTTTGACTCGTGAAACATTCTTTTTAACTTTTGTTTT
TTCTGTTGGTTTATCATTTGTTTTTTCACTAATTTCAATACCATCTTGAC
GTTCATTCATAACTTCATCTTTTTTTTTTCCTGTTTCTGTATCTTCTTCT
ATTTTTTTTTCTTTATCTTTTTCTTTATCTTCTTCTTGTTCTTCCTCTTC
TTTTTCTTCTTCTGATACTGCAGGTGTTTCTTCTTCTTCTTCTTCCGATA
TTGTCGGTTTTTCTACTTCTTCTTCTTGTTCTTCTTCTTCTTCTTCTTCT
TCTTCTTCTTCTTCTTCTTCTTCTTCTTCTTCCGGTAATTTATTAATTAT
ATTTCTTTTTTTATATGAATTACGTTTGGTTTGTGCAGTAATTTCCTTAC
ATAGAGTGCAGCTTTCAAGAAAAATTTCAATTTCTTCGTTTGTTGCATAA
TAACCACTGTCTTTGATATGATTAAACATTTTTGATTTTCTTAAATGCTT
TCCTTCTTTAATATGAAAATTATCGAATTCTAATTCATTAAGAACAATAA
GCTCCCCTAATTTAAAAAATTAGTTAAAATAAATTAAAATGAACATGTAT
AAAGATGGATTTTACCATTTTTTGAAATTCTAAATAACTTTTCTTCATCT
CCAATCTTTTTGACTGAAAAACGATTTTTAATTGAAGTTATTGTTCTGTG
AGTGTTTTGAATCGCCCATTTCTCTAAATCAGTTTGAGATAGTGTTTTAT
AATCTGAATTGTTATACACAACTTTTGCTCTATTAACCAAATATTTAAAG
ATTTCATCATCAACTGAATATTTTGACTTTACGATTCTTGTCCAAAAAAC
AATTTCTACTACTATCATTTTTTATTTATAAAATAATTTAAATACAAAAA
TGAATTTTTTTTTTTTTAAAAAAAAAAAAAATTTGAAAAAAAAAAAAAAAA
AATTTTAAAAAAAAAAAAAAAAAAAAAAAAAAAAAAAAAATCAAATAAAA
AGTAAAAAATAAAAACCGAAAAACATTCATTGTAATTTCAAATGTCGAGG
CCGGCAGAGGCGGTTTGCGTATTGGGCGCTCTTCCGCTTCCTCGCTCACT
GACTCGCTGCGCTCGGTCGTTCGGCTGCGGCGAGCGGTATCAGCTCACTC
AAAGGCGGTAATACGGTTATCCACAGAATCAGGGGATAACGCAGGAAAGA
ACATGTGAGCAAAAGGCCAGCAAAAGGCCAGGAACCGTAAAAAGGCCGCG
TTGCTGGCGTTTTTCCATAGGCTCCGCCCCCCTGACGAGCATCACAAAAA
TCGACGCTCAAGTCAGAGGTGGCGAAACCCGACAGGACTATAAAGATACC
AGGCGTTTCCCCCTGGAAGCTCCCTCGTGCGCTCTCCTGTTCCGACCCTG
CCGCTTACCGGATACCTGTCCGCCTTTCTCCCTTCGGGAAGCGTGGCGCT
TTCTCATAGCTCACGCTGTAGGTATCTCAGTTCGGTGTAGGTCGTTCGCT
CCAAGCTGGGCTGTGTGCACGAACCCCCCGTTCAGCCCGACCGCTGCGCC
TTATCCGGTAACTATCGTCTTGAGTCCAACCCGGTAAGACACGACTTATC
GCCACTGGCAGCAGCCACTGGTAACAGGATTAGCAGAGCGAGGTATGTAG
GCGGTGCTACAGAGTTCTTGAAGTGGTGGCCTAACTACGGCTACACTAGA
AGGACAGTATTTGGTATCTGCGCTCTGCTGAAGCCAGTTACCTTCGGAAA
AAGAGTTGGTAGCTCTTGATCCGGCAAACAAACCACCGCTGGTAGCGGTG
GTTTTTTTGTTTGCAAGCAGCAGATTACGCGCAGAAAAAAAGGATCTCAA
GAAGATCCTTTGATCTTTTCTACGGGGTCTGACGCTCAGTGGAACGAAAA
CTCACGTTAAGGGATTTTGGTCATGAGATTATCAAAAAGGATCTTCACCT
AGATCCTTTTAAATTAAAAATGAAGTTTTAAATCAATCTAAAGTATATAT
GAGTAAACTTGGTCTGACAGTTACCAATGCTTAATCAGTGAGGCACCTAT
CTCAGCGATCTGTCTATTTCGTTCATCCATAGTTGCCTGACTCCCCGTCG
TGTAGATAACTACGATACGGGAGGGCTTACCATCTGGCCCCAGTGCTGCA
ATGATACCGCGAGACCCACGCTCACCGGCTCCAGATTTATCAGCAATAAA
CCAGCCAGCCGGAAGGGCCGAGCGCAGAAGTGGTCCTGCAACTTTATCCG

CCTCCATCCAGTCTATTAATTGTTGCCGGGAAGCTAGAGTAAGTAGTTCG
CCAGTTAATAGTTTGCGCAACGTTGTTGCCATTGCTACAGGCATCGTGGT
GTCACGCTCGTCGTTTGGTATGGCTTCATTCAGCTCCGGTTCCCAACGAT
CAAGGCGAGTTACATGATCCCCCATGTTGTGCAAAAAAGCGGTTAGCTCC
TTCGGTCCTCCGATCGTTGTCAGAAGTAAGTTGGCCGCAGTGTTATCACT
CATGGTTATGGCAGCACTGCATAATTCTCTTACTGTCATGCCATCCGTAA
GATGCTTTTCTGTGACTGGTGAGTACTCAACCAAGTCATTCTGAGAATAG
TGTATGCGGCGACCGAGTTGCTCTTGCCCGGCGTCAATACGGGATAATAC
CGCGCCACATAGCAGAACTTTAAAAGTGCTCATCATTGGAAAACGTTCTT
CGGGGCGAAAACTCTCAAGGATCTTACCGCTGTTGAGATCCAGTTCGATG
TAACCCACTCGTGCACCCAACTGATCTTCAGCATCTTTTACTTTCACCAG
CGTTTCTGGGTGAGCAAAAACAGGAAGGCAAAATGCCGCAAAAAAGGGAA
TAAGGGCGACACGGAAATGTTGAATACTCATACTCTTCCTTTTTCAATAT
TATTGAAGCATTTATCAGGGTTATTGTCTCATGAGCGGATACATATTTGA
ATGTATTTAGAAAAATAAACAAATAGGGGTTCCGCGCACATTTCCCCGAA
AAGTGCCACCTGACGCGCCCTGTAGCGGGATCCATTTTATTTAATATACT
AAATAATAAAAAAGTTAAAAAATGATCATTGGATAAATTTTTTATAATTA
TAAATAAAGATAATAATTTTTTTTTTAACAAAACTAAAAATAAAAATAAT
AAAATAATTGTTAAAATAGGTTTTTTTTTTTTTTTTTTTTTTTTAATAAA
TGGTATTTATTAATTTATTTGTTGTGTGTGTTTTTTTTTTTATAATATTT
TTTTTTTTAGCATTGAATTAAGAAGAAATCAAATTGATGCGGCCGCTCAG
AAGAACTCGTCAAGAAGGCGATAGAAGGCGATGCGCTGCGAATCGGGAGC
GGCGATACCGTAAAGCACGAGGAAGCGGTCAGCCCATTCGCCGCCAAGCT
CTTCAGCAATATCACGGGTAGCCAACGCTATGTCCTGATAGCGGTCCGCC
ACACCCAGCCGTCCACAGTCGATGAATCCAGAAAAGCGGCCATTTTCCAC
CATGATATTCGGCAAGCAGGCATCGCCATGGGTCACGACGAGATCCTCGC
CGTCGGGCATGCGCGCCTTGAGCCTGGCGAACAGTTCGGCTGGCGCGAGC
CCCTGATGCTCTTCGTCCAGATCATCCTGATCGACAAGACCGGCTTCCAT
CCGAGTACGTGCTCGCTCGATGCGATGTTTCGCTTGGTGGTCGAATGGGC
AGGTAGCCGGATCAAGCGTATGCAGCCGCCGCATTGCATCAGCCATGATG
GATACTTTCTCGGCAGGAGCAAGGTGAGATGACAGGAGATCCTGCCCCGG
CACTTCGCCCAATAGCAGCCAGTCCCTTCCCGCTTCAGTGACAACGTCGA
GCACAGCTGCGCAAGGAACGCCCGTCGTGGCCAGCCACGATAGCCGCGCT
GCCTCGTCCTGCAGTTCATTCAGGGCACCGGACAGGTCGGTCTTGACAAA
AAGAACCGGGCGCCCCTGCGCTGACAGCCGGAACACGGCGGCATCAGAGC
AGCCGATTGTCTGTTGTGCCCAGTCATAGCCGAATAGCCTCTCCACCCAA
GCGGCCGGAGAACCTGCGTGCAATCCATCTTGTTCAATCATGCGAAACGA
TCCAGCTTGAACATCTTCACCATCCATTTTTTGCTAGCTGTGAAATTAGT
TTAAAATACAAATAAAGAGTTATAATAATATACAGTTGAATAAAAAAAAA
AAAAATGAATTGGAAAATTTATTTTTATATGAAGAAAAAAAAAATTTTGAA
AAAAAAAAAAAAAATTAAAAAAAAAAAAAAAAAAAAAAAAAAATTTAAAATTA
TTCCACTGTGGGGGGCCCCAAATTTTATTTAAAAAAAAAAAAAAAAATGGG
TCCCTTTTGGGGGGTTGGAAAAAAAAAAAAAAAAAAAAAAAAAAAAATTGAA
AATATAATGTTAGTCATATGATTAATCATT

shuttle vectors

pDM344 – No tag

GCCGGCTAAAAAAAATTTTTATTTATTTTTATTTATTTTGAATTAAATAG

ATTACAAATTAATTAATCCCATCAAATCTTTAAAAAAAAAATGGTTTAAAA
AAACTTGGGTTGGTTAATTATTATTTGAAAATTTTAAAACCCAAATTAAA
AAAAAAAAATGGGATTCAAAAATTTTTTTTTTTTTTTTTTTTTTTT
TTTTTTTTTTTCAGATTGCATAAAAAGATTTTTTTTTTTTTTTTTCTTA
TTTCTTAAAACAAATAAATTAAATTAAATAAAAAATAAAAATCAGATCTA
GTACTAGTTAAATAAATAAATTATTTAATAAATAATAAAAAAACAAATTG
TTGTAATAATCTAATATTTTCTTTTTTTTTTAATTTTTTTTTTTTAAATC
TTAATAATTATTAAGTTATTTTAATTTTTTTTTTTTTTTTTTTTTTTTT
TTTTTTTTTTTTCTATCAAAAAAATCAAATATATTTAAAAAATTTATTAT
TTACAGATACATTTTGAATGGTGAAGATAAATATATGCATTAGATGTAAA
ACAGCCAAAGAGTATGAAAATCAAAAAGATAGCCGGCCATCAAGCTTGGC
ACTGGCCGTCGTTTTACAACGTCGTGACTGGGAAAACCCTGGCGTTACCC
AACTTAATCGCCTTGCAGCACATCCCCCTTTCGCCAGCTGGCGTAATAGC
GAAGAGGCCCGCACCGATCGCCCTTCCCAACAGTTGCGCAGCCTGAATGG
CGAATGGCGCCTGATGCGGTATTTTCTCCTTACGCATCTGTGCGGTATTT
CACACCGCATATGGTGCACTCTCAGTACAATCTGCTCTGATGCCGCATAG
TTAAGCCAGCCCCGACACCCGCCAACACCCGCTGACGCGCCCTGACGGGC
TTGTCTGCTCCCGGCATCCGCTTACAGACAAGCTGTGACCGTCTCCGGGA
GCTGCATGTGTCAGAGGTTTTCACCGTCATCACCGAAACGCGCGAGACGA
AAGGGCCTCGTGATACGCCTATTTTTATAGGTTAATGTCATGATAATAAT
GGTTTCTTAGACGTCAGGTGGCACTTTTCGGGGAAATGTGCGCGGAACCC
CTATTTGTTTATTTTTCTAAATACATTCAAATATGTATCCGCTCATGAGA
CAATAACCCTGATAAATGCTTCAATAATATTGAAAAAGGAAGAGTATGAG
TATTCAACATTTCCGTGTCGCCCTTATTCCCTTTTTTGCGGCATTTTGCC
TTCCTGTTTTTGCTCACCCAGAAACGCTGGTGAAAGTAAAAGATGCTGAA
GATCAGTTGGGTGCACGAGTGGGTTACATCGAACTGGATCTCAACAGCGG
TAAGATCCTTGAGAGTTTTCGCCCCGAAGAACGTTTTCCAATGATGAGCA
CTTTTAAAGTTCTGCTATGTGGCGCGGTATTATCCCGTATTGACGCCGGG
CAAGAGCAACTCGGTCGCCGCATACACTATTCTCAGAATGACTTGGTTGA
GTACTCACCAGTCACAGAAAAGCATCTTACGGATGGCATGACAGTAAGAG
AATTATGCAGTGCTGCCATAACCATGAGTGATAACACTGCGGCCAACTTA
CTTCTGACAACGATCGGAGGACCGAAGGAGCTAACCGCTTTTTTGCACAA
CATGGGGGATCATGTAACTCGCCTTGATCGTTGGGAACCGGAGCTGAATG
AAGCCATACCAAACGACGAGCGTGACACCACGATGCCTGTAGCAATGGCA
ACAACGTTGCGCAAACTATTAACTGGCGAACTACTTACTCTAGCTTCCCG
GCAACAATTAATAGACTGGATGGAGGCGGATAAAGTTGCAGGACCACTTC
TGCGCTCGGCCCTTCCGGCTGGCTGGTTTATTGCTGATAAATCTGGAGCC
GGTGAGCGTGGGTCTCGCGGTATCATTGCAGCACTGGGGCCAGATGGTAA
GCCCTCCCGTATCGTAGTTATCTACACGACGGGGAGTCAGGCAACTATGG
ATGAACGAAATAGACAGATCGCTGAGATAGGTGCCTCACTGATTAAGCAT
TGGTAACTGTCAGACCAAGTTTACTCATATATACTTTAGATTGATTTAAA
ACTTCATTTTTAATTTAAAAGGATCTAGGTGAAGATCCTTTTTGATAATC
TCATGACCAAAATCCCTTAACGTGAGTTTTCGTTCCACTGAGCGTCAGAC
CCCGTAGAAAAGATCAAAGGATCTTCTTGAGATCCTTTTTTTCTGCGCGT
AATCTGCTGCTTGCAAACAAAAAAACCACCGCTACCAGCGGTGGTTTGTT
TGCCGGATCAAGAGCTACCAACTCTTTTTCCGAAGGTAACTGGCTTCAGC
AGAGCGCAGATACCAAATACTGTCCTTCTAGTGTAGCCGTAGTTAGGCCA
CCACTTCAAGAACTCTGTAGCACCGCCTACATACCTCGCTCTGCTAATCC
TGTTACCAGTGGCTGCTGCCAGTGGCGATAAGTCGTGTCTTACCGGGTTG
GACTCAAGACGATAGTTACCGGATAAGGCGCAGCGGTCGGGCTGAACGGG

GGGTTCGTGCACACAGCCCAGCTTGGAGCGAACGACCTACACCGAACTGA
GATACCTACAGCGTGAGCTATGAGAAAGCGCCACGCTTCCCGAAGGGAGA
AAGGCGGACAGGTATCCGGTAAGCGGCAGGGTCGGAACAGGAGAGCGCAC
GAGGGAGCTTCCAGGGGGAAACGCCTGGTATCTTTATAGTCCTGTCGGGT
TTCGCCACCTCTGACTTGAGCGTCGATTTTTGTGATGCTCGTCAGGGGGG
CGGAGCCTATGGAAAAACGCCAGCAACGCGGCCTTTTTACGGTTCCTGGC
CTTTTGCTGGCCTTTTGCTCACATGTTCTTTCCTGCGTTATCCCCTGATT
CTGTGGATAACCGTATTACCGCCTTTGAGTGAGCTGATACCGCTCGCCGC
AGCCGAACGACCGAGCGCAGCGAGTCAGTGAGCGAGGAAGCGGAAGAGCG
CCCAATACGCAAACCGCCTCTCCCCGCGCGTTGGCCGATTCATTAATGCA
GCTGGCACGACAGGTTTCCCGACTGGAAAGCGGGCAGTGAGCGCAACGCA
ATTAATGTGAGTTAGCTCACTCATTAGGCACCCCAGGCTTTACACTTTAT
GCTTCCGGCTCGTATGTTGTGTGGAATTGTGAGCGGATAACAATTTCACA
CAGGAAACAGCTATGACCATGATTACGAATTCGATG

pDM1019 – GFP (N)

GCCGGCTAAAAAAAATTTTTATTTATTTTTATTTATTTTGAATTAAATAG
ATTACAAATTAATTAATCCCATCAAATCTTTAAAAAAAAATGGTTTAAAA
AAACTTGGGTTGGTTAATTATTATTTGAAAATTTTAAAACCCAAATTAAA
AAAAAAAAATGGGATTCAAAAATTTTTTTTTTTTTTTTTTTTTTTTTTT
TTTTTTTTTTTCAGATTGCATAAAAAGATTTTTTTTTTTTTTTTTCTTA
TTTCTTAAAACAAATAAATTAAATTAAATAAAAAATAAAAATCAGATCCA
AAAAATGTCAAAAGGTGAAGAATTATTTACAGGTGTTGTTCCAATTTTAG
TTGAATTAGATGGTGATGTTAATGGTCATAAATTTTCAGTTTCAGGTGAA
GGTGAAGGTGATGCAACATATGGTAAATTAACATTAAAATTTATTTGTAC
AACAGGTAAATTACCAGTTCCATGGCCAACATTAGTTACAACATTTACAT
ATGGTGTTCAATGTTTTTCAAGATATCCAGATCATATGAAACAACATGAT
TTTTTTAAATCAGCAATGCCAGAAGGTTATGTTCAAGAAAGAACAATTTT
TTTTAAAGATGATGGTAATTATAAAACAAGAGCAGAAGTTAAATTTGAAG
GTGATACATTAGTTAATAGAATTGAATTAAAAGGTATTGATTTTAAAGAA
GATGGTAATATTTTAGGTCATAAATTAGAATATAATTATAATTCACATAA
TGTTTATATTATGGCAGATAAACAAAAAAATGGTATTAAAGTTAATTTTA
AAATTAGACATAATATTGAAGATGGTTCAGTTCAATTAGCAGATCATTAT
CAACAAAATACACCAATTGGTGATGGTCCAGTTTTATTACCAGATAATCA
TTATTTATCAACACAATCAGCATTATCAAAAGATCCAAATGAAAAAAGAG
ATCATATGGTTTTATTAGAATTTGTTACAGCAGCAGGTATTACACATGGT
ATGGATGAATTATATAAAGGTGGTTCAGGAGGTAGTAGATCTTCAACTAG
TTAAATAAATAAATTATTTAATAAATAATAAAAAAACAAATTGTTGTAAT
AATCTAATATTTTCTTTTTTTTTTAATTTTTTTTTTTTAAATCTTAATAA
TTATTAAGTTATTTTAATTTTTTTTTTTTTTTTTTTTTTTTTTTTTTTTT
TTTTTCTATCAAAAAAATCAAATATATTTAAAAAATTTATTATTTACAGA
TACATTTTGAATGGTGAAGATAAATATATGCATTAGATGTAAAACAGCCA
AAGAGTATGAAAATCAAAAAGATAGCCGGCCATCAAGCTTGGCACTGGCC
GTCGTTTTACAACGTCGTGACTGGGAAAACCCTGGCGTTACCCAACTTAA
TCGCCTTGCAGCACATCCCCCTTTCGCCAGCTGGCGTAATAGCGAAGAGG
CCCGCACCGATCGCCCTTCCCAACAGTTGCGCAGCCTGAATGGCGAATGG
CGCCTGATGCGGTATTTTCTCCTTACGCATCTGTGCGGTATTTCACACCG
CATATGGTGCACTCTCAGTACAATCTGCTCTGATGCCGCATAGTTAAGCC
AGCCCCGACACCCGCCAACACCCGCTGACGCGCCCTGACGGGCTTGTCTG

CTCCCGGCATCCGCTTACAGACAAGCTGTGACCGTCTCCGGGAGCTGCAT
GTGTCAGAGGTTTTCACCGTCATCACCGAAACGCGCGAGACGAAAGGGCC
TCGTGATACGCCTATTTTTATAGGTTAATGTCATGATAATAATGGTTTCT
TAGACGTCAGGTGGCACTTTTCGGGGAAATGTGCGCGGAACCCCTATTTG
TTTATTTTTCTAAATACATTCAAATATGTATCCGCTCATGAGACAATAAC
CCTGATAAATGCTTCAATAATATTGAAAAAGGAAGAGTATGAGTATTCAA
CATTTCCGTGTCGCCCTTATTCCCTTTTTTGCGGCATTTTGCCTTCCTGT
TTTTGCTCACCCAGAAACGCTGGTGAAAGTAAAAGATGCTGAAGATCAGT
TGGGTGCACGAGTGGGTTACATCGAACTGGATCTCAACAGCGGTAAGATC
CTTGAGAGTTTTCGCCCCGAAGAACGTTTTCCAATGATGAGCACTTTTAA
AGTTCTGCTATGTGGCGCGGTATTATCCCGTATTGACGCCGGGCAAGAGC
AACTCGGTCGCCGCATACACTATTCTCAGAATGACTTGGTTGAGTACTCA
CCAGTCACAGAAAAGCATCTTACGGATGGCATGACAGTAAGAGAATTATG
CAGTGCTGCCATAACCATGAGTGATAACACTGCGGCCAACTTACTTCTGA
CAACGATCGGAGGACCGAAGGAGCTAACCGCTTTTTTGCACAACATGGGG
GATCATGTAACTCGCCTTGATCGTTGGGAACCGGAGCTGAATGAAGCCAT
ACCAAACGACGAGCGTGACACCACGATGCCTGTAGCAATGGCAACAACGT
TGCGCAAACTATTAACTGGCGAACTACTTACTCTAGCTTCCCGGCAACAA
TTAATAGACTGGATGGAGGCGGATAAAGTTGCAGGACCACTTCTGCGCTC
GGCCCTTCCGGCTGGCTGGTTTATTGCTGATAAATCTGGAGCCGGTGAGC
GTGGGTCTCGCGGTATCATTGCAGCACTGGGGCCAGATGGTAAGCCCTCC
CGTATCGTAGTTATCTACACGACGGGGAGTCAGGCAACTATGGATGAACG
AAATAGACAGATCGCTGAGATAGGTGCCTCACTGATTAAGCATTGGTAAC
TGTCAGACCAAGTTTACTCATATATACTTTAGATTGATTTAAAACTTCAT
TTTTAATTTAAAAGGATCTAGGTGAAGATCCTTTTTGATAATCTCATGAC
CAAAATCCCTTAACGTGAGTTTTCGTTCCACTGAGCGTCAGACCCCGTAG
AAAAGATCAAAGGATCTTCTTGAGATCCTTTTTTTCTGCGCGTAATCTGC
TGCTTGCAAACAAAAAAACCACCGCTACCAGCGGTGGTTTGTTTGCCGGA
TCAAGAGCTACCAACTCTTTTTCCGAAGGTAACTGGCTTCAGCAGAGCGC
AGATACCAAATACTGTCCTTCTAGTGTAGCCGTAGTTAGGCCACCACTTC
AAGAACTCTGTAGCACCGCCTACATACCTCGCTCTGCTAATCCTGTTACC
AGTGGCTGCTGCCAGTGGCGATAAGTCGTGTCTTACCGGGTTGGACTCAA
GACGATAGTTACCGGATAAGGCGCAGCGGTCGGGCTGAACGGGGGGTTCG
TGCACACAGCCCAGCTTGGAGCGAACGACCTACACCGAACTGAGATACCT
ACAGCGTGAGCTATGAGAAAGCGCCACGCTTCCCGAAGGGAGAAAGGCGG
ACAGGTATCCGGTAAGCGGCAGGGTCGGAACAGGAGAGCGCACGAGGGAG
CTTCCAGGGGGAAACGCCTGGTATCTTTATAGTCCTGTCGGGTTTCGCCA
CCTCTGACTTGAGCGTCGATTTTTGTGATGCTCGTCAGGGGGGCGGAGCC
TATGGAAAAACGCCAGCAACGCGGCCTTTTTACGGTTCCTGGCCTTTTGC
TGGCCTTTTGCTCACATGTTCTTTCCTGCGTTATCCCCTGATTCTGTGGA
TAACCGTATTACCGCCTTTGAGTGAGCTGATACCGCTCGCCGCAGCCGAA
CGACCGAGCGCAGCGAGTCAGTGAGCGAGGAAGCGGAAGAGCGCCCAATA
CGCAAACCGCCTCTCCCCGCGCGTTGGCCGATTCATTAATGCAGCTGGCA
CGACAGGTTTCCCGACTGGAAAGCGGGCAGTGAGCGCAACGCAATTAATG
TGAGTTAGCTCACTCATTAGGCACCCCAGGCTTTACACTTTATGCTTCCG
GCTCGTATGTTGTGTGGAATTGTGAGCGGATAACAATTTCACACAGGAAA
CAGCTATGACCATGATTACGAATTCGATG

pDM1021 – GFP (C)

GCCGGCTAAAAAAAATTTTTATTTATTTTTATTTATTTTGAATTAAATAG
ATTACAAATTAATTAATCCCATCAAATCTTTAAAAAAAAAATGGTTTAAAA
AAACTTGGGTTGGTTAATTATTATTTGAAAATTTTAAAACCCAAATTAAA
AAAAAAAAATGGGATTCAAAAATTTTTTTTTTTTTTTTTTTTTTTTT
TTTTTTTTTTTCAGATTGCATAAAAAGATTTTTTTTTTTTTTTTCTTA
TTTCTTAAAACAAATAAATTAAATTAAATAAAAAATAAAAATCAGATCTT
CAACTAGTGGTGGTTCAGGAGGTAGTTCAAAAGGTGAAGAATTATTTACA
GGTGTTGTTCCAATTTTAGTTGAATTAGATGGTGATGTTAATGGTCATAA
ATTTTCAGTTTCAGGTGAAGGTGAAGGTGATGCAACATATGGTAAATTAA
CATTAAAATTTATTTGTACAACAGGTAAATTACCAGTTCCATGGCCAACA
TTAGTTACAACATTTACATATGGTGTTCAATGTTTTTCAAGATATCCAGA
TCATATGAAACAACATGATTTTTTTAAATCAGCAATGCCAGAAGGTTATG
TTCAAGAAAGAACAATTTTTTTTAAAGATGATGGTAATTATAAAACAAGA
GCAGAAGTTAAATTTGAAGGTGATACATTAGTTAATAGAATTGAATTAAA
AGGTATTGATTTTAAAGAAGATGGTAATATTTTAGGTCATAAATTAGAAT
ATAATTATAATTCACATAATGTTTATATTATGGCAGATAAACAAAAAAAT
GGTATTAAAGTTAATTTTAAAATTAGACATAATATTGAAGATGGTTCAGT
TCAATTAGCAGATCATTATCAACAAAATACACCAATTGGTGATGGTCCAG
TTTTATTACCAGATAATCATTATTTATCAACACAATCAGCATTATCAAAA
GATCCAAATGAAAAAAGAGATCATATGGTTTTATTAGAATTTGTTACAGC
AGCAGGTATTACACATGGTATGGATGAATTATATAAATAAGCTAGTTAAA
TAAATAAATTATTTAATAAATAATAAAAAAACAAATTGTTGTAATAATCT
AATATTTTCTTTTTTTTTTAATTTTTTTTTTTTAAATCTTAATAATTATT
AAGTTATTTTAATTTTTTTTTTTTTTTTTTTTTTTTTTTTTTTTTTTTT
CTATCAAAAAAATCAAATATATTTAAAAAATTTATTATTTACAGATACAT
TTTGAATGGTGAAGATAAATATATGCATTAGATGTAAAACAGCCAAAGAG
TATGAAAATCAAAAAGATAGCCGGCCATCAAGCTTGGCACTGGCCGTCGT
TTTACAACGTCGTGACTGGGAAAACCCTGGCGTTACCCAACTTAATCGCC
TTGCAGCACATCCCCCTTTCGCCAGCTGGCGTAATAGCGAAGAGGCCCGC
ACCGATCGCCCTTCCCAACAGTTGCGCAGCCTGAATGGCGAATGGCGCCT
GATGCGGTATTTTCTCCTTACGCATCTGTGCGGTATTTCACACCGCATAT
GGTGCACTCTCAGTACAATCTGCTCTGATGCCGCATAGTTAAGCCAGCCC
CGACACCCGCCAACACCCGCTGACGCGCCCTGACGGGCTTGTCTGCTCCC
GGCATCCGCTTACAGACAAGCTGTGACCGTCTCCGGGAGCTGCATGTGTC
AGAGGTTTTCACCGTCATCACCGAAACGCGCGAGACGAAAGGGCCTCGTG
ATACGCCTATTTTTATAGGTTAATGTCATGATAATAATGGTTTCTTAGAC
GTCAGGTGGCACTTTTCGGGGAAATGTGCGCGGAACCCCTATTTGTTTAT
TTTTCTAAATACATTCAAATATGTATCCGCTCATGAGACAATAACCCTGA
TAAATGCTTCAATAATATTGAAAAAGGAAGAGTATGAGTATTCAACATTT
CCGTGTCGCCCTTATTCCCTTTTTTGCGGCATTTTGCCTTCCTGTTTTTG
CTCACCCAGAAACGCTGGTGAAAGTAAAAGATGCTGAAGATCAGTTGGGT
GCACGAGTGGGTTACATCGAACTGGATCTCAACAGCGGTAAGATCCTTGA
GAGTTTTCGCCCCGAAGAACGTTTTCCAATGATGAGCACTTTTAAAGTTC
TGCTATGTGGCGCGGTATTATCCCGTATTGACGCCGGGCAAGAGCAACTC
GGTCGCCGCATACACTATTCTCAGAATGACTTGGTTGAGTACTCACCAGT
CACAGAAAAGCATCTTACGGATGGCATGACAGTAAGAGAATTATGCAGTG
CTGCCATAACCATGAGTGATAACACTGCGGCCAACTTACTTCTGACAACG
ATCGGAGGACCGAAGGAGCTAACCGCTTTTTTGCACAACATGGGGGATCA
TGTAACTCGCCTTGATCGTTGGGAACCGGAGCTGAATGAAGCCATACCAA
ACGACGAGCGTGACACCACGATGCCTGTAGCAATGGCAACAACGTTGCGC

AAACTATTAACTGGCGAACTACTTACTCTAGCTTCCCGGCAACAATTAAT
AGACTGGATGGAGGCGGATAAAGTTGCAGGACCACTTCTGCGCTCGGCCC
TTCCGGCTGGCTGGTTTATTGCTGATAAATCTGGAGCCGGTGAGCGTGGG
TCTCGCGGTATCATTGCAGCACTGGGGCCAGATGGTAAGCCCTCCCGTAT
CGTAGTTATCTACACGACGGGGAGTCAGGCAACTATGGATGAACGAAATA
GACAGATCGCTGAGATAGGTGCCTCACTGATTAAGCATTGGTAACTGTCA
GACCAAGTTTACTCATATATACTTTAGATTGATTTAAAACTTCATTTTTA
ATTTAAAAGGATCTAGGTGAAGATCCTTTTTGATAATCTCATGACCAAAA
TCCCTTAACGTGAGTTTTCGTTCCACTGAGCGTCAGACCCCGTAGAAAAG
ATCAAAGGATCTTCTTGAGATCCTTTTTTTCTGCGCGTAATCTGCTGCTT
GCAAACAAAAAAACCACCGCTACCAGCGGTGGTTTGTTTGCCGGATCAAG
AGCTACCAACTCTTTTTCCGAAGGTAACTGGCTTCAGCAGAGCGCAGATA
CCAAATACTGTCCTTCTAGTGTAGCCGTAGTTAGGCCACCACTTCAAGAA
CTCTGTAGCACCGCCTACATACCTCGCTCTGCTAATCCTGTTACCAGTGG
CTGCTGCCAGTGGCGATAAGTCGTGTCTTACCGGGTTGGACTCAAGACGA
TAGTTACCGGATAAGGCGCAGCGGTCGGGCTGAACGGGGGGTTCGTGCAC
ACAGCCCAGCTTGGAGCGAACGACCTACACCGAACTGAGATACCTACAGC
GTGAGCTATGAGAAAGCGCCACGCTTCCCGAAGGGAGAAAGGCGGACAGG
TATCCGGTAAGCGGCAGGGTCGGAACAGGAGAGCGCACGAGGGAGCTTCC
AGGGGGAAACGCCTGGTATCTTTATAGTCCTGTCGGGTTTCGCCACCTCT
GACTTGAGCGTCGATTTTTGTGATGCTCGTCAGGGGGGCGGAGCCTATGG
AAAAACGCCAGCAACGCGGCCTTTTTACGGTTCCTGGCCTTTTGCTGGCC
TTTTGCTCACATGTTCTTTCCTGCGTTATCCCCTGATTCTGTGGATAACC
GTATTACCGCCTTTGAGTGAGCTGATACCGCTCGCCGCAGCCGAACGACC
GAGCGCAGCGAGTCAGTGAGCGAGGAAGCGGAAGAGCGCCCAATACGCAA
ACCGCCTCTCCCCGCGCGTTGGCCGATTCATTAATGCAGCTGGCACGACA
GGTTTCCCGACTGGAAAGCGGGCAGTGAGCGCAACGCAATTAATGTGAGT
TAGCTCACTCATTAGGCACCCCAGGCTTTACACTTTATGCTTCCGGCTCG
TATGTTGTGTGGAATTGTGAGCGGATAACAATTTCACACAGGAAACAGCT
ATGACCATGATTACGAATTCGATG

pDM1018 – mCherry (N)

GCCGGCTAAAAAAAATTTTTATTTATTTTTATTTATTTTGAATTAAATAG
ATTACAAATTAATTAATCCCATCAAATCTTTAAAAAAAAATGGTTTAAAA
AAACTTGGGTTGGTTAATTATTATTTGAAAATTTTAAAACCCAAATTAAA
AAAAAAAAATGGGATTCAAAAATTTTTTTTTTTTTTTTTTTTTTTTTTTT
TTTTTTTTTTTCAGATTGCATAAAAAGATTTTTTTTTTTTTTTTTCTTA
TTTCTTAAAACAAATAAATTAAATTAAATAAAAAATAAAAATCAGATCCA
AAAAATGGTTTCAAAAGGTGAAGAAGATAATATGGCAATTATTAAAGAAT
TTATGAGATTTAAAGTTCATATGGAAGGTTCAGTTAATGGTCATGAATTT
GAAATTGAAGGTGAAGGTGAAGGTAGACCATATGAAGGTACACAAACAGC
AAAATTAAAAGTTACAAAAGGTGGTCCATTACCATTTGCATGGGATATTT
TATCACCACAATTTATGTATGGTTCAAAAGCATATGTTAAACATCCAGCA
GATATTCCAGATTATTTAAAATTATCATTTCCAGAAGGTTTTAAATGGGA
AAGAGTTATGAATTTTGAAGATGGTGGTGTTGTTACAGTTACACAAGATT
CATCATTACAAGATGGTGAATTTATTTATAAAGTTAAATTAAGAGGTACA
AATTTTCCATCAGATGGTCCAGTTATGCAAAAAAAAACAATGGGTTGGGA
AGCATCATCAGAAAGAATGTATCCAGAAGATGGTGCATTAAAAGGTGAAA
TTAAACAAAGATTAAAATTAAAAGATGGTGGTCATTATGATGCAGAAGTT

AAAACAACATATAAAGCAAAAAAACCAGTTCAATTACCAGGTGCATATAA
TGTTAATATTAAATTAGATATTACATCACATAATGAAGATTATACAATTG
TTGAACAATATGAAAGAGCAGAAGGTAGACATTCAACAGGTGGTATGGAT
GAATTATATAAAGGTGGTTCAGGAGGTAGTAGATCTTCAACTAGTTAAAT
AAATAAATTATTTAATAAATAATAAAAAAACAAATTGTTGTAATAATCTA
ATATTTTCTTTTTTTTTTTAATTTTTTTTTTTAAATCTTAATAATTATTA
AGTTATTTTAATTTTTTTTTTTTTTTTTTTTTTTTTTTTTTTTTTTTTTC
TATCAAAAAAATCAAATATATTTAAAAAATTTATTATTTACAGATACATT
TTGAATGGTGAAGATAAATATATGCATTAGATGTAAAACAGCCAAAGAGT
ATGAAAATCAAAAAGATAGCCGGCCATCAAGCTTGGCACTGGCCGTCGTT
TTACAACGTCGTGACTGGGAAAACCCTGGCGTTACCCAACTTAATCGCCT
TGCAGCACATCCCCCTTTCGCCAGCTGGCGTAATAGCGAAGAGGCCCGCA
CCGATCGCCCTTCCCAACAGTTGCGCAGCCTGAATGGCGAATGGCGCCTG
ATGCGGTATTTTCTCCTTACGCATCTGTGCGGTATTTCACACCGCATATG
GTGCACTCTCAGTACAATCTGCTCTGATGCCGCATAGTTAAGCCAGCCCC
GACACCCGCCAACACCCGCTGACGCGCCCTGACGGGCTTGTCTGCTCCCG
GCATCCGCTTACAGACAAGCTGTGACCGTCTCCGGGAGCTGCATGTGTCA
GAGGTTTTCACCGTCATCACCGAAACGCGCGAGACGAAAGGGCCTCGTGA
TACGCCTATTTTTATAGGTTAATGTCATGATAATAATGGTTTCTTAGACG
TCAGGTGGCACTTTTCGGGGAAATGTGCGCGGAACCCCTATTTGTTTATT
TTTCTAAATACATTCAAATATGTATCCGCTCATGAGACAATAACCCTGAT
AAATGCTTCAATAATATTGAAAAAGGAAGAGTATGAGTATTCAACATTTC
CGTGTCGCCCTTATTCCCTTTTTTGCGGCATTTTGCCTTCCTGTTTTTGC
TCACCCAGAAACGCTGGTGAAAGTAAAAGATGCTGAAGATCAGTTGGGTG
CACGAGTGGGTTACATCGAACTGGATCTCAACAGCGGTAAGATCCTTGAG
AGTTTTCGCCCCGAAGAACGTTTTCCAATGATGAGCACTTTTAAAGTTCT
GCTATGTGGCGCGGTATTATCCCGTATTGACGCCGGGCAAGAGCAACTCG
GTCGCCGCATACACTATTCTCAGAATGACTTGGTTGAGTACTCACCAGTC
ACAGAAAAGCATCTTACGGATGGCATGACAGTAAGAGAATTATGCAGTGC
TGCCATAACCATGAGTGATAACACTGCGGCCAACTTACTTCTGACAACGA
TCGGAGGACCGAAGGAGCTAACCGCTTTTTTGCACAACATGGGGGATCAT
GTAACTCGCCTTGATCGTTGGGAACCGGAGCTGAATGAAGCCATACCAAA
CGACGAGCGTGACACCACGATGCCTGTAGCAATGGCAACAACGTTGCGCA
AACTATTAACTGGCGAACTACTTACTCTAGCTTCCCGGCAACAATTAATA
GACTGGATGGAGGCGGATAAAGTTGCAGGACCACTTCTGCGCTCGGCCCT
TCCGGCTGGCTGGTTTATTGCTGATAAATCTGGAGCCGGTGAGCGTGGGT
CTCGCGGTATCATTGCAGCACTGGGGCCAGATGGTAAGCCCTCCCGTATC
GTAGTTATCTACACGACGGGGAGTCAGGCAACTATGGATGAACGAAATAG
ACAGATCGCTGAGATAGGTGCCTCACTGATTAAGCATTGGTAACTGTCAG
ACCAAGTTTACTCATATATACTTTAGATTGATTTAAAACTTCATTTTTAA
TTTAAAAGGATCTAGGTGAAGATCCTTTTTGATAATCTCATGACCAAAAT
CCCTTAACGTGAGTTTTCGTTCCACTGAGCGTCAGACCCCGTAGAAAAGA
TCAAAGGATCTTCTTGAGATCCTTTTTTTCTGCGCGTAATCTGCTGCTTG
CAAACAAAAAAACCACCGCTACCAGCGGTGGTTTGTTTGCCGGATCAAGA
GCTACCAACTCTTTTTCCGAAGGTAACTGGCTTCAGCAGAGCGCAGATAC
CAAATACTGTCCTTCTAGTGTAGCCGTAGTTAGGCCACCACTTCAAGAAC
TCTGTAGCACCGCCTACATACCTCGCTCTGCTAATCCTGTTACCAGTGGC
TGCTGCCAGTGGCGATAAGTCGTGTCTTACCGGGTTGGACTCAAGACGAT
AGTTACCGGATAAGGCGCAGCGGTCGGGCTGAACGGGGGGTTCGTGCACA
CAGCCCAGCTTGGAGCGAACGACCTACACCGAACTGAGATACCTACAGCG

TGAGCTATGAGAAAGCGCCACGCTTCCCGAAGGGAGAAAGGCGGACAGGT
ATCCGGTAAGCGGCAGGGTCGGAACAGGAGAGCGCACGAGGGAGCTTCCA
GGGGGAAACGCCTGGTATCTTTATAGTCCTGTCGGGTTTCGCCACCTCTG
ACTTGAGCGTCGATTTTTGTGATGCTCGTCAGGGGGGCGGAGCCTATGGA
AAAACGCCAGCAACGCGGCCTTTTTACGGTTCCTGGCCTTTTGCTGGCCT
TTTGCTCACATGTTCTTTCCTGCGTTATCCCCTGATTCTGTGGATAACCG
TATTACCGCCTTTGAGTGAGCTGATACCGCTCGCCGCAGCCGAACGACCG
AGCGCAGCGAGTCAGTGAGCGAGGAAGCGGAAGAGCGCCCAATACGCAAA
CCGCCTCTCCCCGCGCGTTGGCCGATTCATTAATGCAGCTGGCACGACAG
GTTTCCCGACTGGAAAGCGGGCAGTGAGCGCAACGCAATTAATGTGAGTT
AGCTCACTCATTAGGCACCCCAGGCTTTACACTTTATGCTTCCGGCTCGT
ATGTTGTGTGGAATTGTGAGCGGATAACAATTTCACACAGGAAACAGCTA
TGACCATGATTACGAATTCGATG

pDM1020 – mCherry (C)

GCCGGCTAAAAAAAATTTTATTTATTTTTATTTATTTTGAATTAAATAG
ATTACAAATTAATTAATCCCATCAAATCTTTAAAAAAAAAATGGTTTAAAA
AAACTTGGGTTGGTTAATTATTATTTGAAAATTTTAAAACCCAAATTAAA
AAAAAAAAATGGGATTCAAAAATTTTTTTTTTTTTTTTTTTTTTTTTTTTT
TTTTTTTTTTTCAGATTGCATAAAAAGATTTTTTTTTTTTTTTTTTTTCTTA
TTTCTTAAAACAAATAAATTAAATTAAATAAAAAAATAAAAATCAGATCTT
CAACTAGTGGTGGTTCAGGAGGTAGTGTTTCAAAAGGTGAAGAAGATAAT
ATGGCAATTATTAAAGAATTTATGAGATTTAAAGTTCATATGGAAGGTTC
AGTTAATGGTCATGAATTTGAAATTGAAGGTGAAGGTGAAGGTAGACCAT
ATGAAGGTACACAAACAGCAAAATTAAAAGTTACAAAAGGTGGTCCATTA
CCATTTGCATGGGATATTTTATCACCACAATTTATGTATGGTTCAAAAGC
ATATGTTAAACATCCAGCAGATATTCCAGATTATTTAAAATTATCATTTC
CAGAAGGTTTTAAATGGGAAAGAGTTATGAATTTTGAAGATGGTGGTGTT
GTTACAGTTACACAAGATTCATCATTACAAGATGGTGAATTTATTTATAA
AGTTAAATTAAGAGGTACAAATTTTCCATCAGATGGTCCAGTTATGCAAA
AGAAAACAATGGGTTGGGAAGCATCATCAGAAAGAATGTATCCAGAAGAT
GGTGCATTAAAAGGTGAAATTAAACAAAGATTAAAATTAAAAGATGGTGG
TCATTATGATGCAGAAGTTAAAACAACATATAAAGCAAAAAAACCAGTTC
AATTACCAGGTGCATATAATGTTAATATTAAATTAGATATTACATCACAT
AATGAAGATTATACAATTGTTGAACAATATGAAAGAGCAGAAGGTAGACA
TTCAACAGGTGGTATGGATGAATTATATAAATAAGCTAGTTAAATAAATA
AATTATTTAATAAATAATAAAAAAACAAATTGTTGTAATAATCTAATATT
TTCTTTTTTTTTTTAATTTTTTTTTTTTAAATCTTAATAATTATTAAGTTA
TTTTAATTTTTTTTTTTTTTTTTTTTTTTTTTTTTTTTTTTTTTCTATCA
AAAAAATCAAATATATTTAAAAAATTTATTATTTACAGATACATTTTGAA
TGGTGAAGATAAATATATGCATTAGATGTAAAACAGCCAAAGAGTATGAA
AATCAAAAAGATAGCCGGCCATCAAGCTTGGCACTGGCCGTCGTTTTACA
ACGTCGTGACTGGGAAAACCCTGGCGTTACCCAACTTAATCGCCTTGCAG
CACATCCCCCTTTCGCCAGCTGGCGTAATAGCGAAGAGGCCCGCACCGAT
CGCCCTTCCCAACAGTTGCGCAGCCTGAATGGCGAATGGCGCCTGATGCG
GTATTTTCTCCTTACGCATCTGTGCGGTATTTCACACCGCATATGGTGCA
CTCTCAGTACAATCTGCTCTGATGCCGCATAGTTAAGCCAGCCCCGACAC
CCGCCAACACCCGCTGACGCGCCCTGACGGGCTTGTCTGCTCCCGGCATC
CGCTTACAGACAAGCTGTGACCGTCTCCGGGAGCTGCATGTGTCAGAGGT

TTTCACCGTCATCACCGAAACGCGCGAGACGAAAGGGCCTCGTGATACGC
CTATTTTTATAGGTTAATGTCATGATAATAATGGTTTCTTAGACGTCAGG
TGGCACTTTTCGGGGAAATGTGCGCGGAACCCCTATTTGTTTATTTTTCT
AAATACATTCAAATATGTATCCGCTCATGAGACAATAACCCTGATAAATG
CTTCAATAATATTGAAAAAGGAAGAGTATGAGTATTCAACATTTCCGTGT
CGCCCTTATTCCCTTTTTTGCGGCATTTTGCCTTCCTGTTTTTGCTCACC
CAGAAACGCTGGTGAAAGTAAAAGATGCTGAAGATCAGTTGGGTGCACGA
GTGGGTTACATCGAACTGGATCTCAACAGCGGTAAGATCCTTGAGAGTTT
TCGCCCCGAAGAACGTTTTCCAATGATGAGCACTTTTAAAGTTCTGCTAT
GTGGCGCGGTATTATCCCGTATTGACGCCGGGCAAGAGCAACTCGGTCGC
CGCATACACTATTCTCAGAATGACTTGGTTGAGTACTCACCAGTCACAGA
AAAGCATCTTACGGATGGCATGACAGTAAGAGAATTATGCAGTGCTGCCA
TAACCATGAGTGATAACACTGCGGCCAACTTACTTCTGACAACGATCGGA
GGACCGAAGGAGCTAACCGCTTTTTTGCACAACATGGGGGATCATGTAAC
TCGCCTTGATCGTTGGGAACCGGAGCTGAATGAAGCCATACCAAACGACG
AGCGTGACACCACGATGCCTGTAGCAATGGCAACAACGTTGCGCAAACTA
TTAACTGGCGAACTACTTACTCTAGCTTCCCGGCAACAATTAATAGACTG
GATGGAGGCGGATAAAGTTGCAGGACCACTTCTGCGCTCGGCCCTTCCGG
CTGGCTGGTTTATTGCTGATAAATCTGGAGCCGGTGAGCGTGGGTCTCGC
GGTATCATTGCAGCACTGGGGCCAGATGGTAAGCCCTCCCGTATCGTAGT
TATCTACACGACGGGGAGTCAGGCAACTATGGATGAACGAAATAGACAGA
TCGCTGAGATAGGTGCCTCACTGATTAAGCATTGGTAACTGTCAGACCAA
GTTTACTCATATATACTTTAGATTGATTTAAAACTTCATTTTTAATTTAA
AAGGATCTAGGTGAAGATCCTTTTTGATAATCTCATGACCAAAATCCCTT
AACGTGAGTTTTCGTTCCACTGAGCGTCAGACCCCGTAGAAAAGATCAAA
GGATCTTCTTGAGATCCTTTTTTTCTGCGCGTAATCTGCTGCTTGCAAAC
AAAAAAACCACCGCTACCAGCGGTGGTTTGTTTGCCGGATCAAGAGCTAC
CAACTCTTTTTCCGAAGGTAACTGGCTTCAGCAGAGCGCAGATACCAAAT
ACTGTCCTTCTAGTGTAGCCGTAGTTAGGCCACCACTTCAAGAACTCTGT
AGCACCGCCTACATACCTCGCTCTGCTAATCCTGTTACCAGTGGCTGCTG
CCAGTGGCGATAAGTCGTGTCTTACCGGGTTGGACTCAAGACGATAGTTA
CCGGATAAGGCGCAGCGGTCGGGCTGAACGGGGGGTTCGTGCACACAGCC
CAGCTTGGAGCGAACGACCTACACCGAACTGAGATACCTACAGCGTGAGC
TATGAGAAAGCGCCACGCTTCCCGAAGGGAGAAAGGCGGACAGGTATCCG
GTAAGCGGCAGGGTCGGAACAGGAGAGCGCACGAGGGAGCTTCCAGGGGG
AAACGCCTGGTATCTTTATAGTCCTGTCGGGTTTCGCCACCTCTGACTTG
AGCGTCGATTTTTGTGATGCTCGTCAGGGGGGCGGAGCCTATGGAAAAAC
GCCAGCAACGCGGCCTTTTTACGGTTCCTGGCCTTTTGCTGGCCTTTTGC
TCACATGTTCTTTCCTGCGTTATCCCCTGATTCTGTGGATAACCGTATTA
CCGCCTTTGAGTGAGCTGATACCGCTCGCCGCAGCCGAACGACCGAGCGC
AGCGAGTCAGTGAGCGAGGAAGCGGAAGAGCGCCCAATACGCAAACCGCC
TCTCCCCGCGCGTTGGCCGATTCATTAATGCAGCTGGCACGACAGGTTTC
CCGACTGGAAAGCGGGCAGTGAGCGCAACGCAATTAATGTGAGTTAGCTC
ACTCATTAGGCACCCCAGGCTTTACACTTTATGCTTCCGGCTCGTATGTT
GTGTGGAATTGTGAGCGGATAACAATTTCACACAGGAAACAGCTATGACC
ATGATTACGAATTCGATG

pPI153 – mNeon (C)

GCCGGCTAAAAAAAATTTTTATTTATTTTTATTTATTTTGAATTAAATAG

ATTACAAATTAATTAATCCCATCAAATCTTTAAAAAAAAATGGTTTAAAA
AAACTTGGGTTGGTTAATTATTATTTGAAAATTTTAAAACCCAAATTAAA
AAAAAAAAATGGGATTCAAAAATTTTTTTTTTTTTTTTTTTTTTTTTTTT
TTTTTTTTTTTCAGATTGCATAAAAAGATTTTTTTTTTTTTTTTTTCTTA
TTTCTTAAAACAAATAAATTAAATTAAATAAAAAATAAAAATCAGATCTT
CAACTAGTGGTGGTTCAGGAGGTAGTGTGAGTAAAGGTGAAGAAGATAAT
ATGGCATCGTTACCAGCTACACATGAGTTACATATATTCGGTAGCATTAA
TGGTGTTGATTTTGATATGGTGGGACAAGGTACCGGTAATCCTAATGATG
GTTACGAAGAACTAAATTTAAAATCGACTAAAGGTGACTTACAATTTTCT
CCATGGATTTTAGTGCCACATATAGGGTATGGTTTTCATCAATACTTACC
ATATCCAGATGGTATGTCACCATTTCAAGCTGCAATGGTTGATGGATCAG
GTTATCAAGTTCATAGAACAATGCAATTTGAAGATGGTGCTTCATTAACT
GTTAATTATAGATACACATATGAAGGCTCACATATTAAAGGTGAAGCTCA
AGTTAAAGGTACTGGTTTCCCAGCCGATGGCCCAGTTATGACAAATAGTT
TAACAGCAGCAGATTGGTGTAGATCCAAAAAAACTTATCCAAATGATAAA
ACAATTATTTCAACTTTTAAATGGTCATATACAACCGGTAATGGTAAACG
TTATCGTTCAACAGCCCGTACAACATATACTTTTGCTAAACCAATGGCAG
CTAATTATTTAAAAAATCAACCAATGTATGTTTTTCGTAAAACAGAGTTA
AAACATTCAAAAACAGAACTTAATTTTAAAGAATGGCAAAAAGCATTTAC
AGACGTTATGTAAGCTAGTTAAATAAATAAATTATTTAATAAATAATAAA
AAAACAAATTGTTGTAATAATCTAATATTTTCTTTTTTTTTTAATTTTTT
TTTTTTAAATCTTAATAATTATTAAGTTATTTTAATTTTTTTTTTTTTTT
TTTTTTTTTTTTTTTTTTTTTTTCTATCAAAAAAATCAAATATATTTAAA
AAATTTATTATTTACAGATACATTTTGAATGGTGAAGATAAATATATGCA
TTAGATGTAAAACAGCCAAAGAGTATGAAAATCAAAAAGATAGCCGGCCA
TCAAGCTTGGCACTGGCCGTCGTTTTACAACGTCGTGACTGGGAAAACCC
TGGCGTTACCCAACTTAATCGCCTTGCAGCACATCCCCCTTTCGCCAGCT
GGCGTAATAGCGAAGAGGCCCGCACCGATCGCCCTTCCCAACAGTTGCGC
AGCCTGAATGGCGAATGGCGCCTGATGCGGTATTTTCTCCTTACGCATCT
GTGCGGTATTTCACACCGCATATGGTGCACTCTCAGTACAATCTGCTCTG
ATGCCGCATAGTTAAGCCAGCCCCGACACCCGCCAACACCCGCTGACGCG
CCCTGACGGGCTTGTCTGCTCCCGGCATCCGCTTACAGACAAGCTGTGAC
CGTCTCCGGGAGCTGCATGTGTCAGAGGTTTTCACCGTCATCACCGAAAC
GCGCGAGACGAAAGGGCCTCGTGATACGCCTATTTTTATAGGTTAATGTC
ATGATAATAATGGTTTCTTAGACGTCAGGTGGCACTTTTCGGGGAAATGT
GCGCGGAACCCCTATTTGTTTATTTTTCTAAATACATTCAAATATGTATC
CGCTCATGAGACAATAACCCTGATAAATGCTTCAATAATATTGAAAAAGG
AAGAGTATGAGTATTCAACATTTCCGTGTCGCCCTTATTCCCTTTTTTGC
GGCATTTTGCCTTCCTGTTTTTGCTCACCCAGAAACGCTGGTGAAAGTAA
AAGATGCTGAAGATCAGTTGGGTGCACGAGTGGGTTACATCGAACTGGAT
CTCAACAGCGGTAAGATCCTTGAGAGTTTTCGCCCCGAAGAACGTTTTCC
AATGATGAGCACTTTTAAAGTTCTGCTATGTGGCGCGGTATTATCCCGTA
TTGACGCCGGGCAAGAGCAACTCGGTCGCCGCATACACTATTCTCAGAAT
GACTTGGTTGAGTACTCACCAGTCACAGAAAAGCATCTTACGGATGGCAT
GACAGTAAGAGAATTATGCAGTGCTGCCATAACCATGAGTGATAACACTG
CGGCCAACTTACTTCTGACAACGATCGGAGGACCGAAGGAGCTAACCGCT
TTTTTGCACAACATGGGGGATCATGTAACTCGCCTTGATCGTTGGGAACC
GGAGCTGAATGAAGCCATACCAAACGACGAGCGTGACACCACGATGCCTG
TAGCAATGGCAACAACGTTGCGCAAACTATTAACTGGCGAACTACTTACT
CTAGCTTCCCGGCAACAATTAATAGACTGGATGGAGGCGGATAAAGTTGC

AGGACCACTTCTGCGCTCGGCCCTTCCGGCTGGCTGGTTTATTGCTGATA
AATCTGGAGCCGGTGAGCGTGGGTCTCGCGGTATCATTGCAGCACTGGGG
CCAGATGGTAAGCCCTCCCGTATCGTAGTTATCTACACGACGGGGAGTCA
GGCAACTATGGATGAACGAAATAGACAGATCGCTGAGATAGGTGCCTCAC
TGATTAAGCATTGGTAACTGTCAGACCAAGTTTACTCATATATACTTTAG
ATTGATTTAAAACTTCATTTTTAATTTAAAAGGATCTAGGTGAAGATCCT
TTTTGATAATCTCATGACCAAAATCCCTTAACGTGAGTTTTCGTTCCACT
GAGCGTCAGACCCCGTAGAAAAGATCAAAGGATCTTCTTGAGATCCTTTT
TTTCTGCGCGTAATCTGCTGCTTGCAAACAAAAAAACCACCGCTACCAGC
GGTGGTTTGTTTGCCGGATCAAGAGCTACCAACTCTTTTTCCGAAGGTAA
CTGGCTTCAGCAGAGCGCAGATACCAAATACTGTCCTTCTAGTGTAGCCG
TAGTTAGGCCACCACTTCAAGAACTCTGTAGCACCGCCTACATACCTCGC
TCTGCTAATCCTGTTACCAGTGGCTGCTGCCAGTGGCGATAAGTCGTGTC
TTACCGGGTTGGACTCAAGACGATAGTTACCGGATAAGGCGCAGCGGTCG
GGCTGAACGGGGGGTTCGTGCACACAGCCCAGCTTGGAGCGAACGACCTA
CACCGAACTGAGATACCTACAGCGTGAGCTATGAGAAAGCGCCACGCTTC
CCGAAGGGAGAAAGGCGGACAGGTATCCGGTAAGCGGCAGGGTCGGAACA
GGAGAGCGCACGAGGGAGCTTCCAGGGGGAAACGCCTGGTATCTTTATAG
TCCTGTCGGGTTTCGCCACCTCTGACTTGAGCGTCGATTTTTGTGATGCT
CGTCAGGGGGGCGGAGCCTATGGAAAAACGCCAGCAACGCGGCCTTTTTA
CGGTTCCTGGCCTTTTGCTGGCCTTTTGCTCACATGTTCTTTCCTGCGTT
ATCCCCTGATTCTGTGGATAACCGTATTACCGCCTTTGAGTGAGCTGATA
CCGCTCGCCGCAGCCGAACGACCGAGCGCAGCGAGTCAGTGAGCGAGGAA
GCGGAAGAGCGCCCAATACGCAAACCGCCTCTCCCCGCGCGTTGGCCGAT
TCATTAATGCAGCTGGCACGACAGGTTTCCCGACTGGAAAGCGGGCAGTG
AGCGCAACGCAATTAATGTGAGTTAGCTCACTCATTAGGCACCCCAGGCT
TTACACTTTATGCTTCCGGCTCGTATGTTGTGTGGAATTGTGAGCGGATA
ACAATTTCACACAGGAAACAGCTATGACCATGATTACGAATTCGATG

pPI418 – mScarlet (N)

GCCGGCTAAAAAAAATTTTTATTTATTTTTATTTATTTTGAATTAAATAG
ATTACAAATTAATTAATCCCATCAAATCTTTAAAAAAAAAATGGTTTAAAA
AAACTTGGGTTGGTTAATTATTATTTGAAAATTTTAAAACCCAAATTAAA
AAAAAAAAATGGGATTCAAAAATTTTTTTTTTTTTTTTTTTTTTTTT
TTTTTTTTTTTCAGATTGCATAAAAAGATTTTTTTTTTTTTTTTTCTTA
TTTCTTAAAACAAATAAATTAAATTAAATAAAAAATAAAAATCAGATCCA
AAAAATGGTTTCAAAAGGTGAAGCCGTTATTAAAGAATTTATGAGATTCA
AGGTTCACATGGAAGGAAGTATGAACGGTCATGAATTTGAGATTGAAGGA
GAAGGTGAAGGTAGACCATATGAAGGCACCCAAACAGCTAAATTAAAAGT
AACTAAAGGTGGTCCATTACCATTTAGTTGGGATATTTTATCTCCACAAT
TTATGTATGGTTCACGTGCTTTCACAAAACATCCAGCAGATATTCCAGAT
TATTATAAACAATCATTTCCAGAAGGTTTTAAATGGGAACGTGTCATGAA
CTTTGAAGATGGTGGAGCAGTTACAGTCACACAAGATACCTCATTAGAAG
ATGGTACATTAATATATAAAGTTAAATTACGTGGTACTAATTTTCCACCA
GACGGTCCAGTAATGCAAAAAAAAACAATGGGCTGGGAAGCTAGTACAGA
ACGTTTATATCCTGAAGATGGTGTCCTTAAAGGCGATATAAAAATGGCCT
TGAGATTAAAGGATGGTGGTAGGTATTTAGCAGATTTCAAAACCACTTAT
AAAGCAAAAAAACCAGTTCAAATGCCAGGTGCATATAATGTTGATAGAAA
ACTTGATATTACCAGTCATAATGAAGATTACACAGTTGTCGAACAATACG

AACGTTCTGAAGGTCGTCATAGCACTGGTGGTATGGATGAATTATACAAA
GGTGGTTCAGGAGGTAGTAGATCTTCAACTAGTTAAATAAATAAATTATT
TAATAAATAATAAAAAAACAAATTGTTGTAATAATCTAATATTTTCTTTT
TTTTTTAATTTTTTTTTTTTAAATCTTAATAATTATTAAGTTATTTTAAT
TTTTTTTTTTTTTTTTTTTTTTTTTTTTTTTTTTTTTCTATCAAAAAAAT
CAAATATATTTAAAAAATTTATTATTTACAGATACATTTTGAATGGTGAA
GATAAATATATGCATTAGATGTAAAACAGCCAAAGAGTATGAAAATCAAA
AAGATAGCCGGCCATCAAGCTTGGCACTGGCCGTCGTTTTACAACGTCGT
GACTGGGAAAACCCTGGCGTTACCCAACTTAATCGCCTTGCAGCACATCC
CCCTTTCGCCAGCTGGCGTAATAGCGAAGAGGCCCGCACCGATCGCCCTT
CCCAACAGTTGCGCAGCCTGAATGGCGAATGGCGCCTGATGCGGTATTTT
CTCCTTACGCATCTGTGCGGTATTTCACACCGCATATGGTGCACTCTCAG
TACAATCTGCTCTGATGCCGCATAGTTAAGCCAGCCCCGACACCCGCCAA
CACCCGCTGACGCGCCCTGACGGGCTTGTCTGCTCCCGGCATCCGCTTAC
AGACAAGCTGTGACCGTCTCCGGGAGCTGCATGTGTCAGAGGTTTTCACC
GTCATCACCGAAACGCGCGAGACGAAAGGGCCTCGTGATACGCCTATTTT
TATAGGTTAATGTCATGATAATAATGGTTTCTTAGACGTCAGGTGGCACT
TTTCGGGGAAATGTGCGCGGAACCCCTATTTGTTTATTTTTCTAAATACA
TTCAAATATGTATCCGCTCATGAGACAATAACCCTGATAAATGCTTCAAT
AATATTGAAAAAGGAAGAGTATGAGTATTCAACATTTCCGTGTCGCCCTT
ATTCCCTTTTTTGCGGCATTTTGCCTTCCTGTTTTTGCTCACCCAGAAAC
GCTGGTGAAAGTAAAAGATGCTGAAGATCAGTTGGGTGCACGAGTGGGTT
ACATCGAACTGGATCTCAACAGCGGTAAGATCCTTGAGAGTTTTCGCCCC
GAAGAACGTTTTCCAATGATGAGCACTTTTAAAGTTCTGCTATGTGGCGC
GGTATTATCCCGTATTGACGCCGGGCAAGAGCAACTCGGTCGCCGCATAC
ACTATTCTCAGAATGACTTGGTTGAGTACTCACCAGTCACAGAAAAGCAT
CTTACGGATGGCATGACAGTAAGAGAATTATGCAGTGCTGCCATAACCAT
GAGTGATAACACTGCGGCCAACTTACTTCTGACAACGATCGGAGGACCGA
AGGAGCTAACCGCTTTTTTGCACAACATGGGGGATCATGTAACTCGCCTT
GATCGTTGGGAACCGGAGCTGAATGAAGCCATACCAAACGACGAGCGTGA
CACCACGATGCCTGTAGCAATGGCAACAACGTTGCGCAAACTATTAACTG
GCGAACTACTTACTCTAGCTTCCCGGCAACAATTAATAGACTGGATGGAG
GCGGATAAAGTTGCAGGACCACTTCTGCGCTCGGCCCTTCCGGCTGGCTG
GTTTATTGCTGATAAATCTGGAGCCGGTGAGCGTGGGTCTCGCGGTATCA
TTGCAGCACTGGGGCCAGATGGTAAGCCCTCCCGTATCGTAGTTATCTAC
ACGACGGGGAGTCAGGCAACTATGGATGAACGAAATAGACAGATCGCTGA
GATAGGTGCCTCACTGATTAAGCATTGGTAACTGTCAGACCAAGTTTACT
CATATATACTTTAGATTGATTTAAAACTTCATTTTTAATTTAAAAGGATC
TAGGTGAAGATCCTTTTTGATAATCTCATGACCAAAATCCCTTAACGTGA
GTTTTCGTTCCACTGAGCGTCAGACCCCGTAGAAAAGATCAAAGGATCTT
CTTGAGATCCTTTTTTTCTGCGCGTAATCTGCTGCTTGCAAACAAAAAAA
CCACCGCTACCAGCGGTGGTTTGTTTGCCGGATCAAGAGCTACCAACTCT
TTTTCCGAAGGTAACTGGCTTCAGCAGAGCGCAGATACCAAATACTGTCC
TTCTAGTGTAGCCGTAGTTAGGCCACCACTTCAAGAACTCTGTAGCACCG
CCTACATACCTCGCTCTGCTAATCCTGTTACCAGTGGCTGCTGCCAGTGG
CGATAAGTCGTGTCTTACCGGGTTGGACTCAAGACGATAGTTACCGGATA
AGGCGCAGCGGTCGGGCTGAACGGGGGGTTCGTGCACACAGCCCAGCTTG
GAGCGAACGACCTACACCGAACTGAGATACCTACAGCGTGAGCTATGAGA
AAGCGCCACGCTTCCCGAAGGGAGAAAGGCGGACAGGTATCCGGTAAGCG
GCAGGGTCGGAACAGGAGAGCGCACGAGGGAGCTTCCAGGGGGAAACGCC

TGGTATCTTTATAGTCCTGTCGGGTTTCGCCACCTCTGACTTGAGCGTCG
ATTTTTGTGATGCTCGTCAGGGGGGCGGAGCCTATGGAAAAACGCCAGCA
ACGCGGCCTTTTTACGGTTCCTGGCCTTTTGCTGGCCTTTTGCTCACATG
TTCTTTCCTGCGTTATCCCCTGATTCTGTGGATAACCGTATTACCGCCTT
TGAGTGAGCTGATACCGCTCGCCGCAGCCGAACGACCGAGCGCAGCGAGT
CAGTGAGCGAGGAAGCGGAAGAGCGCCCAATACGCAAACCGCCTCTCCCC
GCGCGTTGGCCGATTCATTAATGCAGCTGGCACGACAGGTTTCCCGACTG
GAAAGCGGGCAGTGAGCGCAACGCAATTAATGTGAGTTAGCTCACTCATT
AGGCACCCCAGGCTTTACACTTTATGCTTCCGGCTCGTATGTTGTGTGGA
ATTGTGAGCGGATAACAATTTCACACAGGAAACAGCTATGACCATGATTA
CGAATTCGATG

pPI457 – mScarlet (C)

GCCGGCTAAAAAAAATTTTATTTATTTTTATTTATTTTGAATTAAATAG
ATTACAAATTAATTAATCCCATCAAATCTTTAAAAAAAAATGGTTTAAAA
AAACTTGGGTTGGTTAATTATTATTTGAAAATTTTAAAACCCAAATTAAA
AAAAAAAAATGGGATTCAAAAATTTTTTTTTTTTTTTTTTTTTTTTTTTT
TTTTTTTTTTTCAGATTGCATAAAAAGATTTTTTTTTTTTTTTTTTCTTA
TTTCTTAAAACAAATAAATTAAATTAAATAAAAAATAAAAATCAGATCTT
CAACTAGTGGTGGTTCAGGAGGTAGTGTTTCAAAAGGTGAAGCCGTTATT
AAAGAATTTATGAGATTCAAGGTTCACATGGAAGGAAGTATGAACGGTCA
TGAATTTGAGATTGAAGGAGAAGGTGAAGGTAGACCATATGAAGGCACCC
AAACAGCTAAATTAAAAGTAACTAAAGGTGGTCCATTACCATTTAGTTGG
GATATTTTATCTCCACAATTTATGTATGGTTCACGTGCTTTCACAAAACA
TCCAGCAGATATTCCAGATTATTATAAACAATCATTTCCAGAAGGTTTTA
AATGGGAACGTGTCATGAACTTTGAAGATGGTGGAGCAGTTACAGTCACA
CAAGATACCTCATTAGAAGATGGTACATTAATATATAAAGTTAAATTACG
TGGTACTAATTTTCCACCAGACGGTCCAGTAATGCAAAAAAAAACAATGG
GCTGGGAAGCTAGTACAGAACGTTTATATCCTGAAGATGGTGTCCTTAAA
GGCGATATAAAAATGGCCTTGAGATTAAAGGATGGTGGTAGGTATTTAGC
AGATTTCAAAACCACTTATAAAGCAAAAAAACCAGTTCAAATGCCAGGTG
CATATAATGTTGATAGAAAACTTGATATTACCAGTCATAATGAAGATTAC
ACAGTTGTCGAACAATACGAACGTTCTGAAGGTCGTCATAGCACTGGTGG
TATGGATGAATTATACAAATAAGCTAGTAGTTAAATAAATAAATTATTTA
ATAAATAATAAAAAAAACAAATTGTTGTAATAATCTAATATTTTCTTTTTT
TTTTAATTTTTTTTTTTTAAATCTTAATAATTATTAAGTTATTTTAATTT
TTTTTTTTTTTTTTTTTTTTTTTTTTTTTTTTTTTCTATCAAAAAAATCA
AATATATTTAAAAAATTTATTATTTACAGATACATTTTGAATGGTGAAGA
TAAATATATGCATTAGATGTAAAACAGCCAAAGAGTATGAAAATCAAAAA
GATAGCCGGCCATCAAGCTTGGCACTGGCCGTCGTTTTACAACGTCGTGA
CTGGGAAAACCCTGGCGTTACCCAACTTAATCGCCTTGCAGCACATCCCC
CTTTCGCCAGCTGGCGTAATAGCGAAGAGGCCCGCACCGATCGCCCTTCC
CAACAGTTGCGCAGCCTGAATGGCGAATGGCGCCTGATGCGGTATTTTCT
CCTTACGCATCTGTGCGGTATTTCACACCGCATATGGTGCACTCTCAGTA
CAATCTGCTCTGATGCCGCATAGTTAAGCCAGCCCCGACACCCGCCAACA
CCCGCTGACGCGCCCTGACGGGCTTGTCTGCTCCCGGCATCCGCTTACAG
ACAAGCTGTGACCGTCTCCGGGAGCTGCATGTGTCAGAGGTTTTCACCGT
CATCACCGAAACGCGCGAGACGAAAGGGCCTCGTGATACGCCTATTTTTA
TAGGTTAATGTCATGATAATAATGGTTTCTTAGACGTCAGGTGGCACTTT

TCGGGGAAATGTGCGCGGAACCCCTATTTGTTTATTTTTCTAAATACATT
CAAATATGTATCCGCTCATGAGACAATAACCCTGATAAATGCTTCAATAA
TATTGAAAAAGGAAGAGTATGAGTATTCAACATTTCCGTGTCGCCCTTAT
TCCCTTTTTTGCGGCATTTTGCCTTCCTGTTTTTGCTCACCCAGAAACGC
TGGTGAAAGTAAAAGATGCTGAAGATCAGTTGGGTGCACGAGTGGGTTAC
ATCGAACTGGATCTCAACAGCGGTAAGATCCTTGAGAGTTTTCGCCCCGA
AGAACGTTTTCCAATGATGAGCACTTTTAAAGTTCTGCTATGTGGCGCGG
TATTATCCCGTATTGACGCCGGGCAAGAGCAACTCGGTCGCCGCATACAC
TATTCTCAGAATGACTTGGTTGAGTACTCACCAGTCACAGAAAAGCATCT
TACGGATGGCATGACAGTAAGAGAATTATGCAGTGCTGCCATAACCATGA
GTGATAACACTGCGGCCAACTTACTTCTGACAACGATCGGAGGACCGAAG
GAGCTAACCGCTTTTTTGCACAACATGGGGGATCATGTAACTCGCCTTGA
TCGTTGGGAACCGGAGCTGAATGAAGCCATACCAAACGACGAGCGTGACA
CCACGATGCCTGTAGCAATGGCAACAACGTTGCGCAAACTATTAACTGGC
GAACTACTTACTCTAGCTTCCCGGCAACAATTAATAGACTGGATGGAGGC
GGATAAAGTTGCAGGACCACTTCTGCGCTCGGCCCTTCCGGCTGGCTGGT
TTATTGCTGATAAATCTGGAGCCGGTGAGCGTGGGTCTCGCGGTATCATT
GCAGCACTGGGGCCAGATGGTAAGCCCTCCCGTATCGTAGTTATCTACAC
GACGGGGAGTCAGGCAACTATGGATGAACGAAATAGACAGATCGCTGAGA
TAGGTGCCTCACTGATTAAGCATTGGTAACTGTCAGACCAAGTTTACTCA
TATATACTTTAGATTGATTTAAAACTTCATTTTTAATTTAAAAGGATCTA
GGTGAAGATCCTTTTTGATAATCTCATGACCAAAATCCCTTAACGTGAGT
TTTCGTTCCACTGAGCGTCAGACCCCGTAGAAAAGATCAAAGGATCTTCT
TGAGATCCTTTTTTTCTGCGCGTAATCTGCTGCTTGCAAACAAAAAAACC
ACCGCTACCAGCGGTGGTTTGTTTGCCGGATCAAGAGCTACCAACTCTTT
TTCCGAAGGTAACTGGCTTCAGCAGAGCGCAGATACCAAATACTGTCCTT
CTAGTGTAGCCGTAGTTAGGCCACCACTTCAAGAACTCTGTAGCACCGCC
TACATACCTCGCTCTGCTAATCCTGTTACCAGTGGCTGCTGCCAGTGGCG
ATAAGTCGTGTCTTACCGGGTTGGACTCAAGACGATAGTTACCGGATAAG
GCGCAGCGGTCGGGCTGAACGGGGGGTTCGTGCACACAGCCCAGCTTGGA
GCGAACGACCTACACCGAACTGAGATACCTACAGCGTGAGCTATGAGAAA
GCGCCACGCTTCCCGAAGGGAGAAAGGCGGACAGGTATCCGGTAAGCGGC
AGGGTCGGAACAGGAGAGCGCACGAGGGAGCTTCCAGGGGGAAACGCCTG
GTATCTTTATAGTCCTGTCGGGTTTCGCCACCTCTGACTTGAGCGTCGAT
TTTTGTGATGCTCGTCAGGGGGGCGGAGCCTATGGAAAAACGCCAGCAAC
GCGGCCTTTTTACGGTTCCTGGCCTTTTGCTGGCCTTTTGCTCACATGTT
CTTTCCTGCGTTATCCCCTGATTCTGTGGATAACCGTATTACCGCCTTTG
AGTGAGCTGATACCGCTCGCCGCAGCCGAACGACCGAGCGCAGCGAGTCA
GTGAGCGAGGAAGCGGAAGAGCGCCCAATACGCAAACCGCCTCTCCCCGC
GCGTTGGCCGATTCATTAATGCAGCTGGCACGACAGGTTTCCCGACTGGA
AAGCGGGCAGTGAGCGCAACGCAATTAATGTGAGTTAGCTCACTCATTAG
GCACCCCAGGCTTTACACTTTATGCTTCCGGCTCGTATGTTGTGTGGAAT
TGTGAGCGGATAACAATTTCACACAGGAAACAGCTATGACCATGATTACG
AATTCGATG

inducible vectors

pDM1038 – No tag

AAGCTTCCGGGGGATCGAGTTTACCACTCCCTATCAGTGATAGAGAAAAG

TGAAAGTCGAGTTTACCACTCCCTATCAGTGATAGAGAAAAGTGAAAGTC
GAGTTTACCACTCCCTATCAGTGATAGAGAAAAGTGAAAGTCGAGTTTAC
CACTCCCTATCAGTGATAGAGAAAAGTGAAAGTCGAGTTTACCACTCCCT
ATCAGTGATAGAGAAAAGTGAAAGTCGAGTTTACCACTCCCTATCAGTGA
TAGAGAAAAGTGAAAGTCGAGTTTACCACTCCCTATCAGTGATAGAGAAA
AGTGAAAGTCGAGCTCGGTACCCAATTTTTTTTTTTTTTTTTTTTTTTTT
TTTTTTTTTTTTTTCAGATTGCATAAAAAGATTTTTTTTTTTTTTTTTTC
TTATTTCTTAAAACAAATAAATTAAATTAAATAAAAAATAAAAATCAGAT
CTAGTACTAGTTAAATAAATAAATTATTTAATAAATAATAAAAAAACAAA
TTGTTGTAATAATCTAATATTTCTTTTTTTTTTAATTTTTTTTTTTTTAA
ATCTTAATAATTATTAAGTTATTTTAATTTTTTTTTTTTTTTTTTTTTTT
TTTTTTTTTTTTTTTCTATCAAAAAAATCAAATATATTTAAAAAATTTAT
TATTTACAGATACATTTTGAATGGTGAAGATAAATATATGCATTAGATGT
AAAACAGCCAAAGAGTATGAAAATCAAAAAGATACTCGAGATTTTATTTA
ATATACTAAATAATAAAAAAGTTAAAAAATGATCATTGGATAAATTTTTT
ATAATTATAAATAAAGATAATAATTTTTTTTTTAACAAAACTAAAAATAA
AAATAATAAAATAATTGTTAAAATAGGTTTTTTTTTTTTTTTTTTTTTTT
TAATAAATGGTATTTATTAATTTATTTGTTGTGTGTTTTTTTTTTTTTAT
AATATTTTTTTTTTTAGCATTGAATTAAGAAGAAATCAAATTGATGCGGC
CGCTTAGTTAGCCTCCCCCATCTCCCGATCCGGACGAGTGCTGGGGCGTC
GGTTTCCACTATCGGCGAGTACTTCTACACAGCCATCGGTCCAAACGGCC
GCGCTTCTGCGGGCGATTTGTGTACGCCCGACAGTCCCGGCTCCGGATCG
GACGATTGCGTCGCATCGACCCTGCGCCCAAGCTGCATCATCGAAATTGC
CGTCAACCAAGCTCTGATAGAGTTGGTCAAGACCAATGCGGAGCATATAC
GCCCGGAGCCGCGGCGATCCTGCAAGCTCCGGATGCCTCCGCTCGAAGTA
GCGCGTCTGCTGCTCCATACAAGCCAACCACGGCCTCCAGAAGAAGATGT
TGGCGACCTCGTATTGGGAATCCCCGAACATCGCCTCGCTCCAGTCAATG
ACCGCTGTTATGCGGCCATTGTCCGTCAGGACATTGTTGGAGCCGAAATC
CGCGTGCACGAGGTGCCGGACTTCGGGGCAGTCCTCGGCCCAAAGCATCA
GCTCATCGAGAGCCTGCGCGACGGACGCACTGACGGTGTCGTCCATCACA
GTTTGCCAGTGATACACATGGGGATCAGCAATCGCGCATATGAAATCACG
CCATGTAGTGTATTGACCGATTCCTTGCGGTCCGAATGGGCCGAACCCGC
TCGTCTGGCTAAGATCGGCCGCAGCGATCGCATCCATGGCCTCCGCGACC
GGCTGCAGAACAGCGGGCAGTTCGGTTTCAGGCAGGTCTTGCAACGTGAC
ACCCTGTGCACGGCGGGAGATGCAATAGGTCAGGCTCTCGCTAAATTCCC
CAATGTCAAGCACTTCCGGAATCGGGAGCGCGGCCGATGCAAAGTGCCGA
TAAACATAACGATCTTTGTAGAAACCATCGGCGCAGCTATTTACCCGCAG
GACATATCCACGCCCTCCTACATCGAAGCTGAAAGCACGAGATTCTTCGC
CCTCCGAGAGCTGCATCAGGTCGGAGACGCTGTCGAACTTTTCGATCAGA
AACTTCTCGACAGACGTCGCGGTGAGTTCAGGCTTTTTCATTAGGTCTTG
TTGAGAAATGTTAAATTGATCCATTTTTTGCTAGCTGTGAAATTAGTTTA
AAATACAAATAAAGAGTTATAATAATATACAGTTGAATAAAAAAAAAAAA
AATGAATTGGAAAATTTATTTTTATATGAAGAAAAAAAAATTTTGAAAAA
AAAAAAAAAAATTAAAAAAAAAAAAAAAAAAAAAAAAAATTAAAAATAAT
TCAACTGTGGGTGACCCAAATTTTATTTAAAAAAAAAAAAAAAATGGATC
ACTTTTGGGTGGTTGGAAAAAAAAAAAAAAAAAAATTGAAAATATAATGT
TAGTCATATGATTAATCATTAATTAATTCTTTTTTTTTTTTTTTTTATTT
TTTATTTTTTTTTTTTCAATATTCCAATTATGACACACACTAACATCACA
AAATACAAAATTTTGGGATCCCGCTACAGGGCGCGTCAGGTGGCACTTTT
CGGGGAAATGTGCGCGGAACCCCTATTTGTTTATTTTTCTAAATACATTC

AAATATGTATCCGCTCATGAGACAATAACCCTGATAAATGCTTCAATAAT
ATTGAAAAAGGAAGAGTATGAGTATTCAACATTTCCGTGTCGCCCTTATT
CCCTTTTTTGCGGCATTTTGCCTTCCTGTTTTTGCTCACCCAGAAACGCT
GGTGAAAGTAAAAGATGCTGAAGATCAGTTGGGTGCACGAGTGGGTTACA
TCGAACTGGATCTCAACAGCGGTAAGATCCTTGAGAGTTTTCGCCCCGAA
GAACGTTTTCCAATGATGAGCACTTTTAAAGTTCTGCTATGTGGCGCGGT
ATTATCCCGTATTGACGCCGGGCAAGAGCAACTCGGTCGCCGCATACACT
ATTCTCAGAATGACTTGGTTGAGTACTCACCAGTCACAGAAAAGCATCTT
ACGGATGGCATGACAGTAAGAGAATTATGCAGTGCTGCCATAACCATGAG
TGATAACACTGCGGCCAACTTACTTCTGACAACGATCGGAGGACCGAAGG
AGCTAACCGCTTTTTTGCACAACATGGGGGATCATGTAACTCGCCTTGAT
CGTTGGGAACCGGAGCTGAATGAAGCCATACCAAACGACGAGCGTGACAC
CACGATGCCTGTAGCAATGGCAACAACGTTGCGCAAACTATTAACTGGCG
AACTACTTACTCTAGCTTCCCGGCAACAATTAATAGACTGGATGGAGGCG
GATAAAGTTGCAGGACCACTTCTGCGCTCGGCCCTTCCGGCTGGCTGGTT
TATTGCTGATAAATCTGGAGCCGGTGAGCGTGGGTCTCGCGGTATCATTG
CAGCACTGGGGCCAGATGGTAAGCCCTCCCGTATCGTAGTTATCTACACG
ACGGGGAGTCAGGCAACTATGGATGAACGAAATAGACAGATCGCTGAGAT
AGGTGCCTCACTGATTAAGCATTGGTAACTGTCAGACCAAGTTTACTCAT
ATATACTTTAGATTGATTTAAAACTTCATTTTTAATTTAAAAGGATCTAG
GTGAAGATCCTTTTTGATAATCTCATGACCAAAATCCCTTAACGTGAGTT
TTCGTTCCACTGAGCGTCAGACCCCGTAGAAAAGATCAAAGGATCTTCTT
GAGATCCTTTTTTTCTGCGCGTAATCTGCTGCTTGCAAACAAAAAAACCA
CCGCTACCAGCGGTGGTTTGTTTGCCGGATCAAGAGCTACCAACTCTTTT
TCCGAAGGTAACTGGCTTCAGCAGAGCGCAGATACCAAATACTGTCCTTC
TAGTGTAGCCGTAGTTAGGCCACCACTTCAAGAACTCTGTAGCACCGCCT
ACATACCTCGCTCTGCTAATCCTGTTACCAGTGGCTGCTGCCAGTGGCGA
TAAGTCGTGTCTTACCGGGTTGGACTCAAGACGATAGTTACCGGATAAGG
CGCAGCGGTCGGGCTGAACGGGGGGTTCGTGCACACAGCCCAGCTTGGAG
CGAACGACCTACACCGAACTGAGATACCTACAGCGTGAGCTATGAGAAAG
CGCCACGCTTCCCGAAGGGAGAAAGGCGGACAGGTATCCGGTAAGCGGCA
GGGTCGGAACAGGAGAGCGCACGAGGGAGCTTCCAGGGGGAAACGCCTGG
TATCTTTATAGTCCTGTCGGGTTTCGCCACCTCTGACTTGAGCGTCGATT
TTTGTGATGCTCGTCAGGGGGGCGGAGCCTATGGAAAAACGCCAGCAACG
CGGCCTTTTTACGGTTCCTGGCCTTTTGCTGGCCTTTTGCTCACATGTTC
TTTCCTGCGTTATCCCCTGATTCTGTGGATAACCGTATTACCGCCTTTGA
GTGAGCTGATACCGCTCGCCGCAGCCGAACGACCGAGCGCAGCGAGTCAG
TGAGCGAGGAAGCGGAAGAGCGCCCAATACGCAAACCGCCTCTGCCGGCT
AAAAAAAATTTTTATTTATTTTTATTTATTTTGAATTAAATAGATTACAA
ATTAATTAATCCCATCAAATCTTTAAAAAAAAAATGGTTTAAAAAAACTTG
GGTTGGTTAATTATTATTTGAAAATTTTAAAACCCAAATTAAAAAAAAAA
AATGGGATTCAAAAATTTTTTTTTTTTTTTTTTTTTTTTTTTTTTTTTTT
TTTTCAGATTGCATAAAAAGATTTTTTTTTTTTTTTTTTCTTATTTCTTA
AAACAAATAAATTAAATTAAATAAAAAATAAAAATCAGATCCAAAATGTC
AAGATTAGATAAAAGTAAAGTGATTAACGGCGCATTAGAGCTGCTTAATG
GGGTCGGAATCGAAGGTTTAACAACCCGTAAACTCGCCCAGAAGCTAGGT
GTAGAGCAGCCTACATTGTATTGGCATGTAAAAAATAAGCGGGCTTTGCT
CGACGCCTTACCCATTGAGATGTTAGATAGGCACCATACTCACTTTTGCC
CTTTAGAAGGGGAAAGCTGGCAAGATTTTTTACGTAATAACGCTAAAAGT
TTTAGATGTGCTTTACTAAGTCATCGCGATGGAGCAAAAGTACATTTAGG

TACACGGCCTACAGAAAAACAGTATGAAACTCTCGAAAATCAATTAGCCT
TTTTATGCCAACAAGGTTTTTCACTAGAGAATGCATTATATGCACTCAGC
GCTGTGGGGCACTTTACTTTAGGTTGCGTATTGGAAGAACAAGAGCATCA
AGTCGCTAAAGAAGAAAGGGAAACACCTACTACTGATAGTATGCCGCCAT
TATTACGACAAGCTATCGAATTATTTGATCGCCAAGGTGCAGAGCCAGCC
TTCTTATTCGGCCTTGAATTGATCATATGCGGATTAGAAAAACAACTTAA
ATGTGAAAGTGGATCACACACTCGTAGACTCTCAACTGCCCCACCAACCG
ATGTCAGCCTGGGGGACGAGCTCCACTTAGACGGCGAGGACGTGGCGATG
GCGCATGCCGACGCGCTAGACGATTTCGATCTGGACATGTTGGGGGACGG
GGATTCCCCGGGCCCGGGATTTACCCCCCACGACTCCGCCCCCTACGGCG
CTCTGGATATGGCCGACTTCGAGTTTGAGCAGATGTTTACCGATGCCCTT
GGAATTGACGAGTACGGTGGTTAATCTAGTACGCGTAAATTGTTCACAGA
AAATGTTTCTAAATTATTTAATAAATAATAAAAAAACAAATTGTTGTAAT
AATCTAATATTTTCTTTTTTTTTTAATTTTTTTTTTTTAAATCTTAATAA
TTATTAAGTTATTTTAATTTTTTTTTTTTTTTTTTTTTTTTTTTTTTTTT
TTTTTCTATCAAAAAAAATCAAATATATTTAAAAAATTTATTATTTACAGA
TACATTTTGAATGGTGAAGATAAATATATGCATTAGATGTAAAACAGCCA
AAGAGTATGAAAATCAAAAAGATAGCCGGCCTCGACATTTGAAATTACAA
TGAATGTTTTTCGGTTTTTATTTTTTACTTTTTATTTGATTTTTTTTTTT
TTTTTTTTTTTTTTTTTTTTTTTAAAATTTTTTTTTTTTTTTTTTCAAAT
TTTTTTTTTTTTAAAAAAAAAAAAAATTCATTTTTGTATTTAAATTATTTT
ATAAATAAAAAATGATAGTAGTAGAAATTGTTTTTTGGACAAGAATCGTA
AAGTCAAAATATTCAGTTGATGATGAAATCTTTAAATATTTGGTTAATAG
AGCAAAAGTTGTGTATAACAATTCAGATTATAAAACACTATCTCAAACTG
ATTTAGAGAAATGGGCGATTCAAAACACTCACAGAACAATAACTTCAATT
AAAAATCGTTTTTCAGTCAAAAAGATTGGAGATGAAGAAAAGTTATTTAG
AATTTCAAAAAATGGTAAAATCCATCTTTATACATGTTCATTTTAATTTA
TTTTAACTAATTTTTTAAATTAGGGGAGCTTATTGTTCTTAATGAATTAG
AATTCGATAATTTTCATATTAAAGAAGGAAAGCATTTAAGAAAATCAAAA
ATGTTTAATCATATCAAAGACAGTGGTTATTATGCAACAAACGAAGAAAT
TGAAATTTTTCTTGAAAGCTGCACTCTATGTAAGGAAATTACTGCACAAA
CCAAACGTAATTCATATAAAAAAAGAAATATAATTAATAAATTACCGGAA
GAAGAAGAAGAAGAAGAAGAAGAAGAAGAAGAAGAAGAAGAAGAAGAAGA
ACAAGAAGAAGAAGTAGAAAAACCGACAATATCGGAAGAAGAAGAAGAAG
AAACACCTGCAGTATCAGAAGAAGAAAAAGAAGAGGAAGAACAAGAAGAA
GATAAAGAAAAAGATAAAGAAAAAAAAATAGAAGAAGATACAGAAACAGG
AAAAAAAAAAGATGAAGTTATGAATGAACGTCAAGATGGTATTGAAATTA
GTGAAAAAACAAATGATAAACCAACAGAAAAAACAAAAGTTAAAAAGAAT
GTTTCACGAGTCAAAGATATTAATCTTCATAAAAAAGGAAATCCTATTAC
CCCAAAAAAAAATAAAAAATTTCAGTCAACAACAATTACGTTTAGCTTCTG
GTGTTTCAAATGAAAAAAGAGCAATAATTACTCTTAAAAAATAATAAAAC
TTTATTTAATTAAAAAAATAATTAAAAAGAAAATTAAATATATATTAATT
TTTAAATTATTTGAAATGTTTTTTTCTTGAGTTACTGACTTTTCCATACC
GTTATTTGAGCCATCGAGCTTTGGATTGTAAATTGTTGATAAAAATTCAG
AATCAGATGCACCATACTTTGGAGTACACTTTGTTGTTTTAAAATCAAGA
TTGTTTTGAATGTTTTCATTCTTACAATAGTGGCACAATGGATCTGTACA
TGTAAATCTATTGGATTTCTCTTGTGTAATGTTTGTAATATGATATACAT
TTTCAAATTTTGAACAAATGTTGAATTTTTCAATAATTGTTTTACTATTT
TTTGTACAATCCATTGAAAGAGAAGTAATTTCAACAAAGCTGTCTTCCAC
TCTAAACAAATAACCATCATCAGTTTCATTATATTCTTTATAAAGAATAG

ACTCTCCAAACTCGTCATTAATACATTCATTAAGTTTAATTAAGTTTGTT
AAATTACGAAAATTGTTACATCTTGAATTATTTGTTTCAATGTCATCATA
AAAAAGCGCATGAGTACTATTTGTTTCAACTCTTCCATGAATATTACATT
CATCATAAATTACGGCGCCTGTCATTGACTTTTCAACACAGTTTTCTTTT
TGATACATAACAATAAGAAGTTGAAAATATTTTACTTCAATCTGATCATT
TTGAAGAACAGAAGATTTTGATACAATAAAAAAAAAAAGAAAAAATAATA
ACGTTGTTGAGTACATTATATACAATGAAAATAATAAATGAACAAAAAAT
AATTTTTTATTATAATATATTAAACCCCACCCCACAACCACACCTAAAAA
AAAGTTAATACTTTTTTAAATATAATTTTTTTAATAGTTATTTTAATACG
TTTTTAACATAAAATATATTTTAATTTTTTTTTTAATTTTTTAGAAAATT
TTTTTTTCATAATTATTTTAATACTTTTTTACAAAAAATATTTTTAAAAT
TTTAAAAATTTTTTTTTTATAATTATTTTAATAGCTTAATTACAAAAACA
ATTTAATAATTTTTTTAATTTTTTTTTCATAATATTTTTAATAGCTTTTT
TACAAATTATTTTTACGAAAATTTTGTTATATTTTTTTTATAATTATTTT
AATACATTTTTAACATTAAATAATTATATAATTTTTTAATACAATAATTA
CAGTTATAAATTCATATTATTTAAATATTTTTAAATGAGTTTATTTAGAA
ATTAAGTTTATATTTTATTTAATTAGTTTTGACTCTCAGAATGTATAGAC
ACTTTTTAGAACACTGTGCACAAAGATGAAGTTTACATTCTTGGTTCAAA
TTACATTTAGTTCCGTTCTTACTACACATGGCACACTTCTTGTTGATTAC
CGAATCATTGAAACATGGAGGGCAAAGTTTAGAATTAATAACAACATTAT
ATATGTTTATGTTTATTCCTAAACAATTATAGCAAATATTGTTTTGACAT
CCATCTCTTGTACATGAAATATATAAATTTTTTCCTTGACACAAAGAATG
ACAATTAATACACTTTACTTCTAAGAGAGAATAATTTTCTGGATTTGAAA
GGGCATCTGTTGAAGAAACTAAAAAGAAATCAATTTTGGAAGAAGTTTTA
TTGACTTCTTCTTTAATAAACTTTTTTAAATCGGCAATTTCATTGGAATT
TATTTGGGTAGATTCGGATTGAATTTTGTAATAATTGCTATTTACTTTTT
CGAAATCGAT

pDM1047 – GFP (N)

AAGCTTCCGGGGGATCGAGTTTACCACTCCCTATCAGTGATAGAGAAAAG
TGAAAGTCGAGTTTACCACTCCCTATCAGTGATAGAGAAAAGTGAAAGTC
GAGTTTACCACTCCCTATCAGTGATAGAGAAAAGTGAAAGTCGAGTTTAC
CACTCCCTATCAGTGATAGAGAAAAGTGAAAGTCGAGTTTACCACTCCCT
ATCAGTGATAGAGAAAAGTGAAAGTCGAGTTTACCACTCCCTATCAGTGA
TAGAGAAAAGTGAAAGTCGAGTTTACCACTCCCTATCAGTGATAGAGAAA
AGTGAAAGTCGAGCTCGGTACCCAATTTTTTTTTTTTTTTTTTTTTTTTT
TTTTTTTTTTTTTTCAGATTGCATAAAAAGATTTTTTTTTTTTTTTTTC
TTATTTCTTAAAACAAATAAATTAAATTAAATAAAAAATAAAAATCAGAT
CCAAAAAATGTCAAAAGGTGAAGAATTATTTACAGGTGTTGTTCCAATTT
TAGTTGAATTAGATGGTGATGTTAATGGTCATAAATTTTCAGTTTCAGGT
GAAGGTGAAGGTGATGCAACATATGGTAAATTAACATTAAAATTTATTTG
TACAACAGGTAAATTACCAGTTCCATGGCCAACATTAGTTACAACATTTA
CATATGGTGTTCAATGTTTTTCAAGATATCCAGATCATATGAAACAACAT
GATTTTTTTAAATCAGCAATGCCAGAAGGTTATGTTCAAGAAAGAACAAT
TTTTTTTAAAGATGATGGTAATTATAAAACAAGAGCAGAAGTTAAATTTG
AAGGTGATACATTAGTTAATAGAATTGAATTAAAAGGTATTGATTTTAAA
GAAGATGGTAATATTTTAGGTCATAAATTAGAATATAATTATAATTCACA
TAATGTTTATATTATGGCAGATAAACAAAAAAATGGTATTAAAGTTAATT
TTAAAATTAGACATAATATTGAAGATGGTTCAGTTCAATTAGCAGATCAT

TATCAACAAAATACACCAATTGGTGATGGTCCAGTTTTATTACCAGATAA
TCATTATTTATCAACACAATCAGCATTATCAAAAGATCCAAATGAAAAAA
GAGATCATATGGTTTTATTAGAATTTGTTACAGCAGCAGGTATTACACAT
GGTATGGATGAATTATATAAAGGTGGTTCAGGAGGTAGTAGATCTTCAAC
TAGTTAAATAAATAAATTATTTAATAAATAATAAAAAAACAAATTGTTGT
AATAATCTAATATTTTCTTTTTTTTTTAATTTTTTTTTTTTAAATCTTAA
TAATTATTAAGTTATTTTAATTTTTTTTTTTTTTTTTTTTTTTTTTTTTT
TTTTTTTTCTATCAAAAAAATCAAATATATTTAAAAAATTTATTATTTAC
AGATACATTTTGAATGGTGAAGATAAATATATGCATTAGATGTAAAACAG
CCAAAGAGTATGAAAATCAAAAAGATACTCGAGATTTTATTTAATATACT
AAATAATAAAAAAGTTAAAAAATGATCATTGGATAAATTTTTTATAATTA
TAAATAAAGATAATAATTTTTTTTTTAACAAAACTAAAAATAAAAATAAT
AAAATAATTGTTAAAATAGGTTTTTTTTTTTTTTTTTTTTTTTTAATAAA
TGGTATTTATTAATTTATTTGTTGTGTGTGTTTTTTTTTTTATAATATTT
TTTTTTTTAGCATTGAATTAAGAAGAAATCAAATTGATGCGGCCGCTTAG
TTAGCCTCCCCCATCTCCCGATCCGGACGAGTGCTGGGGCGTCGGTTTCC
ACTATCGGCGAGTACTTCTACACAGCCATCGGTCCAAACGGCCGCGCTTC
TGCGGGCGATTTGTGTACGCCCGACAGTCCCGGCTCCGGATCGGACGATT
GCGTCGCATCGACCCTGCGCCCAAGCTGCATCATCGAAATTGCCGTCAAC
CAAGCTCTGATAGAGTTGGTCAAGACCAATGCGGAGCATATACGCCCGGA
GCCGCGGCGATCCTGCAAGCTCCGGATGCCTCCGCTCGAAGTAGCGCGTC
TGCTGCTCCATACAAGCCAACCACGGCCTCCAGAAGAAGATGTTGGCGAC
CTCGTATTGGGAATCCCCGAACATCGCCTCGCTCCAGTCAATGACCGCTG
TTATGCGGCCATTGTCCGTCAGGACATTGTTGGAGCCGAAATCCGCGTGC
ACGAGGTGCCGGACTTCGGGGCAGTCCTCGGCCCAAAGCATCAGCTCATC
GAGAGCCTGCGCGACGGACGCACTGACGGTGTCGTCCATCACAGTTTGCC
AGTGATACACATGGGGATCAGCAATCGCGCATATGAAATCACGCCATGTA
GTGTATTGACCGATTCCTTGCGGTCCGAATGGGCCGAACCCGCTCGTCTG
GCTAAGATCGGCCGCAGCGATCGCATCCATGGCCTCCGCGACCGGCTGCA
GAACAGCGGGCAGTTCGGTTTCAGGCAGGTCTTGCAACGTGACACCCTGT
GCACGGCGGGAGATGCAATAGGTCAGGCTCTCGCTAAATTCCCCAATGTC
AAGCACTTCCGGAATCGGGAGCGCGGCCGATGCAAAGTGCCGATAAACAT
AACGATCTTTGTAGAAACCATCGGCGCAGCTATTTACCCGCAGGACATAT
CCACGCCCTCCTACATCGAAGCTGAAAGCACGAGATTCTTCGCCCTCCGA
GAGCTGCATCAGGTCGGAGACGCTGTCGAACTTTTCGATCAGAAACTTCT
CGACAGACGTCGCGGTGAGTTCAGGCTTTTTCATTAGGTCTTGTTGAGAA
ATGTTAAATTGATCCATTTTTTGCTAGCTGTGAAATTAGTTTAAAATACA
AATAAAGAGTTATAATAATATACAGTTGAATAAAAAAAAAAAAAATGAAT
TGGAAAATTTATTTTTATATGAAGAAAAAAAAATTTTGAAAAAAAAAAAA
AAAATTAAAAAAAAAAAAAAAAAAAAAAAAAATTAAAAATAATTCAACTG
TGGGTGACCCAAATTTTATTTAAAAAAAAAAAAAAAATGGATCACTTTTG
GGTGGTTGGAAAAAAAAAAAAAAAAAAATTGAAAATATAATGTTAGTCAT
ATGATTAATCATTAATTAATTCTTTTTTTTTTTTTTTTATTTTTTTATTT
TTTTTTTTTCAATATTCCAATTATGACACACACTAACATCACAAAATACA
AAATTTTGGGATCCCGCTACAGGGCGCGTCAGGTGGCACTTTTCGGGGAA
ATGTGCGCGGAACCCCTATTTGTTTATTTTTCTAAATACATTCAAATATG
TATCCGCTCATGAGACAATAACCCTGATAAATGCTTCAATAATATTGAAA
AAGGAAGAGTATGAGTATTCAACATTTCCGTGTCGCCCTTATTCCCTTTT
TTGCGGCATTTTGCCTTCCTGTTTTTGCTCACCCAGAAACGCTGGTGAAA
GTAAAAGATGCTGAAGATCAGTTGGGTGCACGAGTGGGTTACATCGAACT

GGATCTCAACAGCGGTAAGATCCTTGAGAGTTTTCGCCCCGAAGAACGTT
TTCCAATGATGAGCACTTTTAAAGTTCTGCTATGTGGCGCGGTATTATCC
CGTATTGACGCCGGGCAAGAGCAACTCGGTCGCCGCATACACTATTCTCA
GAATGACTTGGTTGAGTACTCACCAGTCACAGAAAAGCATCTTACGGATG
GCATGACAGTAAGAGAATTATGCAGTGCTGCCATAACCATGAGTGATAAC
ACTGCGGCCAACTTACTTCTGACAACGATCGGAGGACCGAAGGAGCTAAC
CGCTTTTTTGCACAACATGGGGGATCATGTAACTCGCCTTGATCGTTGGG
AACCGGAGCTGAATGAAGCCATACCAAACGACGAGCGTGACACCACGATG
CCTGTAGCAATGGCAACAACGTTGCGCAAACTATTAACTGGCGAACTACT
TACTCTAGCTTCCCGGCAACAATTAATAGACTGGATGGAGGCGGATAAAG
TTGCAGGACCACTTCTGCGCTCGGCCCTTCCGGCTGGCTGGTTTATTGCT
GATAAATCTGGAGCCGGTGAGCGTGGGTCTCGCGGTATCATTGCAGCACT
GGGGCCAGATGGTAAGCCCTCCCGTATCGTAGTTATCTACACGACGGGGA
GTCAGGCAACTATGGATGAACGAAATAGACAGATCGCTGAGATAGGTGCC
TCACTGATTAAGCATTGGTAACTGTCAGACCAAGTTTACTCATATATACT
TTAGATTGATTTAAAACTTCATTTTTAATTTAAAAGGATCTAGGTGAAGA
TCCTTTTTGATAATCTCATGACCAAAATCCCTTAACGTGAGTTTTCGTTC
CACTGAGCGTCAGACCCCGTAGAAAAGATCAAAGGATCTTCTTGAGATCC
TTTTTTTCTGCGCGTAATCTGCTGCTTGCAAACAAAAAAACCACCGCTAC
CAGCGGTGGTTTGTTTGCCGGATCAAGAGCTACCAACTCTTTTTCCGAAG
GTAACTGGCTTCAGCAGAGCGCAGATACCAAATACTGTCCTTCTAGTGTA
GCCGTAGTTAGGCCACCACTTCAAGAACTCTGTAGCACCGCCTACATACC
TCGCTCTGCTAATCCTGTTACCAGTGGCTGCTGCCAGTGGCGATAAGTCG
TGTCTTACCGGGTTGGACTCAAGACGATAGTTACCGGATAAGGCGCAGCG
GTCGGGCTGAACGGGGGGTTCGTGCACACAGCCCAGCTTGGAGCGAACGA
CCTACACCGAACTGAGATACCTACAGCGTGAGCTATGAGAAAGCGCCACG
CTTCCCGAAGGGAGAAAGGCGGACAGGTATCCGGTAAGCGGCAGGGTCGG
AACAGGAGAGCGCACGAGGGAGCTTCCAGGGGGAAACGCCTGGTATCTTT
ATAGTCCTGTCGGGTTTCGCCACCTCTGACTTGAGCGTCGATTTTTGTGA
TGCTCGTCAGGGGGGCGGAGCCTATGGAAAAACGCCAGCAACGCGGCCTT
TTTACGGTTCCTGGCCTTTTGCTGGCCTTTTGCTCACATGTTCTTTCCTG
CGTTATCCCCTGATTCTGTGGATAACCGTATTACCGCCTTTGAGTGAGCT
GATACCGCTCGCCGCAGCCGAACGACCGAGCGCAGCGAGTCAGTGAGCGA
GGAAGCGGAAGAGCGCCCAATACGCAAACCGCCTCTGCCGGCTAAAAAAA
ATTTTTATTTATTTTTATTTATTTTGAATTAAATAGATTACAAATTAATT
AATCCCATCAAATCTTTAAAAAAAAATGGTTTAAAAAAACTTGGGTTGGT
TAATTATTATTTGAAAATTTAAAACCCAAATTAAAAAAAAAAAAAATGGA
TTCAAAAATTTTTTTTTTTTTTTTTTTTTTTTTTTTTTTTTTTTTTTCAG
ATTGCATAAAAAGATTTTTTTTTTTTTTTTTTCTTATTTCTTAAAACAAA
TAAATTAAATTAAATAAAAAATAAAAATCAGATCCAAAATGTCAAGATTA
GATAAAAGTAAAGTGATTAACGGCGCATTAGAGCTGCTTAATGGGGTCGG
AATCGAAGGTTTAACAACCCGTAAACTCGCCCAGAAGCTAGGTGTAGAGC
AGCCTACATTGTATTGGCATGTAAAAAATAAGCGGGCTTTGCTCGACGCC
TTACCCATTGAGATGTTAGATAGGCACCATACTCACTTTTGCCCTTTAGA
AGGGGAAAGCTGGCAAGATTTTTTACGTAATAACGCTAAAAGTTTTAGAT
GTGCTTTACTAAGTCATCGCGATGGAGCAAAAGTACATTTAGGTACACGG
CCTACAGAAAAACAGTATGAAACTCTCGAAAATCAATTAGCCTTTTTATG
CCAACAAGGTTTTTCACTAGAGAATGCATTATATGCACTCAGCGCTGTGG
GGCACTTTACTTTAGGTTGCGTATTGGAAGAACAAGAGCATCAAGTCGCT
AAAGAAGAAAGGGAAACACCTACTACTGATAGTATGCCGCCATTATTACG

ACAAGCTATCGAATTATTTGATCGCCAAGGTGCAGAGCCAGCCTTCTTAT
TCGGCCTTGAATTGATCATATGCGGATTAGAAAAACAACTTAAATGTGAA
AGTGGATCACACACTCGTAGACTCTCAACTGCCCCACCAACCGATGTCAG
CCTGGGGGACGAGCTCCACTTAGACGGCGAGGACGTGGCGATGGCGCATG
CCGACGCGCTAGACGATTTCGATCTGGACATGTTGGGGGACGGGGATTCC
CCGGGCCCGGGATTTACCCCCCACGACTCCGCCCCCTACGGCGCTCTGGA
TATGGCCGACTTCGAGTTTGAGCAGATGTTTACCGATGCCCTTGGAATTG
ACGAGTACGGTGGTTAATCTAGTACGCGTAAATTGTTCACAGAAAATGTT
TCTAAATTATTTAATAAATAATAAAAAAACAAATTGTTGTAATAATCTAA
TATTTTCTTTTTTTTTTTAATTTTTTTTTTTAAATCTTAATAATTATTAA
GTTATTTTAATTTTTTTTTTTTTTTTTTTTTTTTTTTTTTTTTTTTTTCT
ATCAAAAAAATCAAATATATTTAAAAAATTTATTATTTACAGATACATTT
TGAATGGTGAAGATAAATATATGCATTAGATGTAAAACAGCCAAAGAGTA
TGAAAATCAAAAAGATAGCCGGCCTCGACATTTGAAATTACAATGAATGT
TTTTCGGTTTTTATTTTTTACTTTTTATTTGATTTTTTTTTTTTTTTTTT
TTTTTTTTTTTTTTTTAAAATTTTTTTTTTTTTTTTTTCAAATTTTTTTT
TTTTTAAAAAAAAAAAAATTCATTTTTGTATTTAAATTATTTTATAAATA
AAAAATGATAGTAGTAGAAATTGTTTTTTGGACAAGAATCGTAAAGTCAA
AATATTCAGTTGATGATGAAATCTTTAAATATTTGGTTAATAGAGCAAAA
GTTGTGTATAACAATTCAGATTATAAAACACTATCTCAAACTGATTTAGA
GAAATGGGCGATTCAAAACACTCACAGAACAATAACTTCAATTAAAAATC
GTTTTTCAGTCAAAAAGATTGGAGATGAAGAAAAGTTATTTAGAATTTCA
AAAAATGGTAAAATCCATCTTTATACATGTTCATTTTAATTTATTTTAAC
TAATTTTTTAAATTAGGGGAGCTTATTGTTCTTAATGAATTAGAATTCGA
TAATTTTCATATTAAAGAAGGAAAGCATTTAAGAAAATCAAAAATGTTTA
ATCATATCAAAGACAGTGGTTATTATGCAACAAACGAAGAAATTGAAATT
TTTCTTGAAAGCTGCACTCTATGTAAGGAAATTACTGCACAAACCAAACG
TAATTCATATAAAAAAAGAAATATAATTAATAAATTACCGGAAGAAGAAG
AAGAAGAAGAAGAAGAAGAAGAAGAAGAAGAAGAAGAAGAAGAACAAGAA
GAAGAAGTAGAAAAACCGACAATATCGGAAGAAGAAGAAGAAGAAACACC
TGCAGTATCAGAAGAAGAAAAAGAAGAGGAAGAACAAGAAGAAGATAAAG
AAAAAGATAAAGAAAAAAAAATAGAAGAAGATACAGAAACAGGAAAAAAAA
AAAGATGAAGTTATGAATGAACGTCAAGATGGTATTGAAATTAGTGAAAA
AACAAATGATAAACCAACAGAAAAAACAAAAGTTAAAAAGAATGTTTCAC
GAGTCAAAGATATTAATCTTCATAAAAAAGGAAATCCTATTACCCCAAAA
AAAATAAAAAATTTCAGTCAACAACAATTACGTTTAGCTTCTGGTGTTTC
AAATGAAAAAAGAGCAATAATTACTCTTAAAAAATAATAAAACTTTATTT
AATTAAAAAAATAATTAAAAAGAAAATTAAATATATATTAATTTTTAAAT
TATTTGAAATGTTTTTTTCTTGAGTTACTGACTTTTCCATACCGTTATTT
GAGCCATCGAGCTTTGGATTGTAAATTGTTGATAAAAATTCAGAATCAGA
TGCACCATACTTTGGAGTACACTTTGTTGTTTTAAAATCAAGATTGTTTT
GAATGTTTTCATTCTTACAATAGTGGCACAATGGATCTGTACATGTAAAT
CTATTGGATTTCTCTTGTGTAATGTTTGTAATATGATATACATTTTCAAA
TTTTGAACAAATGTTGAATTTTTCAATAATTGTTTTACTATTTTTTGTAC
AATCCATTGAAAGAGAAGTAATTTCAACAAAGCTGTCTTCCACTCTAAAC
AAATAACCATCATCAGTTTCATTATATTCTTTATAAAGAATAGACTCTCC
AAACTCGTCATTAATACATTCATTAAGTTTAATTAAGTTTGTTAAATTAC
GAAAATTGTTACATCTTGAATTATTTGTTTCAATGTCATCATAAAAAAGC
GCATGAGTACTATTTGTTTCAACTCTTCCATGAATATTACATTCATCATA
AATTACGGCGCCTGTCATTGACTTTTCAACACAGTTTTCTTTTTGATACA

TAACAATAAGAAGTTGAAAATATTTTACTTCAATCTGATCATTTTGAAGA
ACAGAAGATTTTGATACAATAAAAAAAAAAAGAAAAAATAATAACGTTGT
TGAGTACATTATATACAATGAAAATAATAAATGAACAAAAAATAATTTTT
TATTATAATATATTAAACCCCACCCCACAACCACACCTAAAAAAAAGTTA
ATACTTTTTTAAATATAATTTTTTTAATAGTTATTTTAATACGTTTTTAA
CATAAAATATATTTTAATTTTTTTTTTAATTTTTTAGAAAATTTTTTTT
CATAATTATTTTAATACTTTTTTACAAAAAATATTTTTAAAATTTTAAAA
ATTTTTTTTTTATAATTATTTTAATAGCTTAATTACAAAAACAATTTAAT
AATTTTTTTAATTTTTTTTTCATAATATTTTTAATAGCTTTTTTACAAAT
TATTTTTACGAAAATTTTTGTTATATTTTTTTATAATTATTTTAATACAT
TTTTAACATTAAATAATTATATAATTTTTTAATACAATAATTACAGTTAT
AAATTCATATTATTTAAATATTTTTAAATGAGTTTATTTAGAAATTAAGT
TTATATTTTATTTAATTAGTTTTGACTCTCAGAATGTATAGACACTTTTT
AGAACACTGTGCACAAAGATGAAGTTTACATTCTTGGTTCAAATTACATT
TAGTTCCGTTCTTACTACACATGGCACACTTCTTGTTGATTACCGAATCA
TTGAAACATGGAGGGCAAAGTTTAGAATTAATAACAACATTATATATGTT
TATGTTTATTCCTAAACAATTATAGCAAATATTGTTTTGACATCCATCTC
TTGTACATGAAATATATAAATTTTTTCCTTGACACAAAGAATGACAATTA
ATACACTTTACTTCTAAGAGAGAATAATTTTCTGGATTTGAAAGGGCATC
TGTTGAAGAAACTAAAAAGAAATCAATTTTGGAAGAAGTTTTATTGACTT
CTTCTTTAATAAACTTTTTTAAATCGGCAATTTCATTGGAATTTATTTGG
GTAGATTCGGATTGAATTTTGTAATAATTGCTATTTACTTTTTCGAAATC
GAT

pDM1049 – GFP (C)

AAGCTTCCGGGGGATCGAGTTTACCACTCCCTATCAGTGATAGAGAAAAG
TGAAAGTCGAGTTTACCACTCCCTATCAGTGATAGAGAAAAGTGAAAGTC
GAGTTTACCACTCCCTATCAGTGATAGAGAAAAGTGAAAGTCGAGTTTAC
CACTCCCTATCAGTGATAGAGAAAAGTGAAAGTCGAGTTTACCACTCCCT
ATCAGTGATAGAGAAAAGTGAAAGTCGAGTTTACCACTCCCTATCAGTGA
TAGAGAAAAGTGAAAGTCGAGTTTACCACTCCCTATCAGTGATAGAGAAA
AGTGAAAGTCGAGCTCGGTACCCAATTTTTTTTTTTTTTTTTTTTTTTTT
TTTTTTTTTTTTTTCAGATTGCATAAAAAGATTTTTTTTTTTTTTTTTTC
TTATTTCTTAAAACAAATAAATTAAATTAAATAAAAAATAAAAATCAGAT
CTTCAACTAGTGGTGGTTCAGGAGGTAGTTCAAAAGGTGAAGAATTATTT
ACAGGTGTTGTTCCAATTTTAGTTGAATTAGATGGTGATGTTAATGGTCA
TAAATTTTCAGTTTCAGGTGAAGGTGAAGGTGATGCAACATATGGTAAAT
TAACATTAAAATTTATTTGTACAACAGGTAAATTACCAGTTCCATGGCCA
ACATTAGTTACAACATTTACATATGGTGTTCAATGTTTTTCAAGATATCC
AGATCATATGAAACAACATGATTTTTTTAAATCAGCAATGCCAGAAGGTT
ATGTTCAAGAAAGAACAATTTTTTTTAAAGATGATGGTAATTATAAAACA
AGAGCAGAAGTTAAATTTGAAGGTGATACATTAGTTAATAGAATTGAATT
AAAAGGTATTGATTTTAAAGAAGATGGTAATATTTTAGGTCATAAATTAG
AATATAATTATAATTCACATAATGTTTATATTATGGCAGATAAACAAAAA
AATGGTATTAAAGTTAATTTTAAAATTAGACATAATATTGAAGATGGTTC
AGTTCAATTAGCAGATCATTATCAACAAAATACACCAATTGGTGATGGTC
CAGTTTTATTACCAGATAATCATTATTTATCAACACAATCAGCATTATCA
AAAGATCCAAATGAAAAAAGAGATCATATGGTTTTATTAGAATTTGTTAC
AGCAGCAGGTATTACACATGGTATGGATGAATTATATAAATAAGCTAGTT

AAATAAATAAATTATTTAATAAATAATAAAAAAACAAATTGTTGTAATAA
TCTAATATTTTCTTTTTTTTTTAATTTTTTTTTTTTAAATCTTAATAATT
ATTAAGTTATTTTAATTTTTTTTTTTTTTTTTTTTTTTTTTTTTTTTTTT
TTTCTATCAAAAAAATCAAATATATTTAAAAAATTTATTATTTACAGATA
CATTTTGAATGGTGAAGATAAATATATGCATTAGATGTAAAACAGCCAAA
GAGTATGAAAATCAAAAAGATACTCGAGATTTTATTTAATATACTAAATA
ATAAAAAAGTTAAAAAATGATCATTGGATAAATTTTTTATAATTATAAAT
AAAGATAATAATTTTTTTTTTAACAAAACTAAAAATAAAAATAATAAAAT
AATTGTTAAAATAGGTTTTTTTTTTTTTTTTTTTTTTTTAATAAATGGTA
TTTATTAATTTATTTGTTGTGTGTGTTTTTTTTTTTATAATATTTTTTTT
TTTAGCATTGAATTAAGAAGAAATCAAATTGATGCGGCCGCTTAGTTAGC
CTCCCCCATCTCCCGATCCGGACGAGTGCTGGGGCGTCGGTTTCCACTAT
CGGCGAGTACTTCTACACAGCCATCGGTCCAAACGGCCGCGCTTCTGCGG
GCGATTTGTGTACGCCCGACAGTCCCGGCTCCGGATCGGACGATTGCGTC
GCATCGACCCTGCGCCCAAGCTGCATCATCGAAATTGCCGTCAACCAAGC
TCTGATAGAGTTGGTCAAGACCAATGCGGAGCATATACGCCCGGAGCCGC
GGCGATCCTGCAAGCTCCGGATGCCTCCGCTCGAAGTAGCGCGTCTGCTG
CTCCATACAAGCCAACCACGGCCTCCAGAAGAAGATGTTGGCGACCTCGT
ATTGGGAATCCCCGAACATCGCCTCGCTCCAGTCAATGACCGCTGTTATG
CGGCCATTGTCCGTCAGGACATTGTTGGAGCCGAAATCCGCGTGCACGAG
GTGCCGGACTTCGGGGCAGTCCTCGGCCCAAAGCATCAGCTCATCGAGAG
CCTGCGCGACGGACGCACTGACGGTGTCGTCCATCACAGTTTGCCAGTGA
TACACATGGGGATCAGCAATCGCGCATATGAAATCACGCCATGTAGTGTA
TTGACCGATTCCTTGCGGTCCGAATGGGCCGAACCCGCTCGTCTGGCTAA
GATCGGCCGCAGCGATCGCATCCATGGCCTCCGCGACCGGCTGCAGAACA
GCGGGCAGTTCGGTTTCAGGCAGGTCTTGCAACGTGACACCCTGTGCACG
GCGGGAGATGCAATAGGTCAGGCTCTCGCTAAATTCCCCAATGTCAAGCA
CTTCCGGAATCGGGAGCGCGGCCGATGCAAAGTGCCGATAAACATAACGA
TCTTTGTAGAAACCATCGGCGCAGCTATTTACCCGCAGGACATATCCACG
CCCTCCTACATCGAAGCTGAAAGCACGAGATTCTTCGCCCTCCGAGAGCT
GCATCAGGTCGGAGACGCTGTCGAACTTTTCGATCAGAAACTTCTCGACA
GACGTCGCGGTGAGTTCAGGCTTTTTCATTAGGTCTTGTTGAGAAATGTT
AAATTGATCCATTTTTTGCTAGCTGTGAAATTAGTTTAAAATACAAATAA
AGAGTTATAATAATATACAGTTGAATAAAAAAAAAAAAAAATGAATTGGAA
AATTTATTTTTATATGAAGAAAAAAAAAATTTTGAAAAAAAAAAAAAAAAT
TAAAAAAAAAAAAAAAAAAAAAAAAAAATTAAAAATAATTCAACTGTGGGT
GACCCAAATTTTATTTAAAAAAAAAAAAAAAAATGGATCACTTTTGGGTGG
TTGGAAAAAAAAAAAAAAAAAAAATTGAAAATATAATGTTAGTCATATGAT
TAATCATTAATTAATTCTTTTTTTTTTTTTTTTTATTTTTTATTTTTTTT
TTTTCAATATTCCAATTATGACACACACTAACATCACAAAATACAAAATT
TTGGGATCCCGCTACAGGGCGCGTCAGGTGGCACTTTTCGGGGAAATGTG
CGCGGAACCCCTATTTGTTTATTTTTCTAAATACATTCAAATATGTATCC
GCTCATGAGACAATAACCCTGATAAATGCTTCAATAATATTGAAAAAGGA
AGAGTATGAGTATTCAACATTTCCGTGTCGCCCTTATTCCCTTTTTTGCG
GCATTTTGCCTTCCTGTTTTTGCTCACCCAGAAACGCTGGTGAAAGTAAA
AGATGCTGAAGATCAGTTGGGTGCACGAGTGGGTTACATCGAACTGGATC
TCAACAGCGGTAAGATCCTTGAGAGTTTTCGCCCCGAAGAACGTTTTCCA
ATGATGAGCACTTTTAAAGTTCTGCTATGTGGCGCGGTATTATCCCGTAT
TGACGCCGGGCAAGAGCAACTCGGTCGCCGCATACACTATTCTCAGAATG
ACTTGGTTGAGTACTCACCAGTCACAGAAAAGCATCTTACGGATGGCATG

ACAGTAAGAGAATTATGCAGTGCTGCCATAACCATGAGTGATAACACTGC
GGCCAACTTACTTCTGACAACGATCGGAGGACCGAAGGAGCTAACCGCTT
TTTTGCACAACATGGGGGATCATGTAACTCGCCTTGATCGTTGGGAACCG
GAGCTGAATGAAGCCATACCAAACGACGAGCGTGACACCACGATGCCTGT
AGCAATGGCAACAACGTTGCGCAAACTATTAACTGGCGAACTACTTACTC
TAGCTTCCCGGCAACAATTAATAGACTGGATGGAGGCGGATAAAGTTGCA
GGACCACTTCTGCGCTCGGCCCTTCCGGCTGGCTGGTTTATTGCTGATAA
ATCTGGAGCCGGTGAGCGTGGGTCTCGCGGTATCATTGCAGCACTGGGGC
CAGATGGTAAGCCCTCCCGTATCGTAGTTATCTACACGACGGGGAGTCAG
GCAACTATGGATGAACGAAATAGACAGATCGCTGAGATAGGTGCCTCACT
GATTAAGCATTGGTAACTGTCAGACCAAGTTTACTCATATATACTTTAGA
TTGATTTAAAACTTCATTTTTAATTTAAAAGGATCTAGGTGAAGATCCTT
TTTGATAATCTCATGACCAAAATCCCTTAACGTGAGTTTTCGTTCCACTG
AGCGTCAGACCCCGTAGAAAAGATCAAAGGATCTTCTTGAGATCCTTTTT
TTCTGCGCGTAATCTGCTGCTTGCAAACAAAAAAACCACCGCTACCAGCG
GTGGTTTGTTTGCCGGATCAAGAGCTACCAACTCTTTTTCCGAAGGTAAC
TGGCTTCAGCAGAGCGCAGATACCAAATACTGTCCTTCTAGTGTAGCCGT
AGTTAGGCCACCACTTCAAGAACTCTGTAGCACCGCCTACATACCTCGCT
CTGCTAATCCTGTTACCAGTGGCTGCTGCCAGTGGCGATAAGTCGTGTCT
TACCGGGTTGGACTCAAGACGATAGTTACCGGATAAGGCGCAGCGGTCGG
GCTGAACGGGGGGTTCGTGCACACAGCCCAGCTTGGAGCGAACGACCTAC
ACCGAACTGAGATACCTACAGCGTGAGCTATGAGAAAGCGCCACGCTTCC
CGAAGGGAGAAAGGCGGACAGGTATCCGGTAAGCGGCAGGGTCGGAACAG
GAGAGCGCACGAGGGAGCTTCCAGGGGGAAACGCCTGGTATCTTTATAGT
CCTGTCGGGTTTCGCCACCTCTGACTTGAGCGTCGATTTTTGTGATGCTC
GTCAGGGGGGCGGAGCCTATGGAAAAACGCCAGCAACGCGGCCTTTTTAC
GGTTCCTGGCCTTTTGCTGGCCTTTTGCTCACATGTTCTTTCCTGCGTTA
TCCCCTGATTCTGTGGATAACCGTATTACCGCCTTTGAGTGAGCTGATAC
CGCTCGCCGCAGCCGAACGACCGAGCGCAGCGAGTCAGTGAGCGAGGAAG
CGGAAGAGCGCCCAATACGCAAACCGCCTCTGCCGGCTAAAAAAAATTTT
TATTTATTTTTATTTATTTTGAATTAAATAGATTACAAATTAATTAATCC
CATCAAATCTTTAAAAAAAAATGGTTTAAAAAAACTTGGGTTGGTTAATT
ATTATTTGAAAATTTTAAAACCCAAATTAAAAAAAAAAAAATGGGATTCAA
AAATTTTTTTTTTTTTTTTTTTTTTTTTTTTTTTTTTTTTCAGATTGC
ATAAAAAGATTTTTTTTTTTTTTTTTTCTTATTTCTTAAAACAAATAAAT
TAAATTAAATAAAAAATAAAAATCAGATCCAAAATGTCAAGATTAGATAA
AAGTAAAGTGATTAACGGCGCATTAGAGCTGCTTAATGGGGTCGGAATCG
AAGGTTTAACAACCCGTAAACTCGCCCAGAAGCTAGGTGTAGAGCAGCCT
ACATTGTATTGGCATGTAAAAAATAAGCGGGCTTTGCTCGACGCCTTACC
CATTGAGATGTTAGATAGGCACCATACTCACTTTTGCCCTTTAGAAGGGG
AAAGCTGGCAAGATTTTTTACGTAATAACGCTAAAAGTTTTAGATGTGCT
TTACTAAGTCATCGCGATGGAGCAAAAGTACATTTAGGTACACGGCCTAC
AGAAAAACAGTATGAAACTCTCGAAAATCAATTAGCCTTTTTATGCCAAC
AAGGTTTTTCACTAGAGAATGCATTATATGCACTCAGCGCTGTGGGGCAC
TTTACTTTAGGTTGCGTATTGGAAGAACAAGAGCATCAAGTCGCTAAAGA
AGAAAGGGAAACACCTACTACTGATAGTATGCCGCCATTATTACGACAAG
CTATCGAATTATTTGATCGCCAAGGTGCAGAGCCAGCCTTCTTATTCGGC
CTTGAATTGATCATATGCGGATTAGAAAAACAACTTAAATGTGAAAGTGG
ATCACACACTCGTAGACTCTCAACTGCCCCACCAACCGATGTCAGCCTGG
GGGACGAGCTCCACTTAGACGGCGAGGACGTGGCGATGGCGCATGCCGAC

GCGCTAGACGATTTCGATCTGGACATGTTGGGGGACGGGGATTCCCCGGG
CCCGGGATTTACCCCCCACGACTCCGCCCCCTACGGCGCTCTGGATATGG
CCGACTTCGAGTTTGAGCAGATGTTTACCGATGCCCTTGGAATTGACGAG
TACGGTGGTTAATCTAGTACGCGTAAATTGTTCACAGAAAATGTTTCTAA
ATTATTTAATAAATAATAAAAAAACAAATTGTTGTAATAATCTAATATTT
TCTTTTTTTTTTAATTTTTTTTTTTTAAATCTTAATAATTATTAAGTTAT
TTTAATTTTTTTTTTTTTTTTTTTTTTTTTTTTTTTTTTTTTTTCTATCAA
AAAAATCAAATATATTTAAAAAATTTATTATTTACAGATACATTTTGAAT
GGTGAAGATAAATATATGCATTAGATGTAAAACAGCCAAAGAGTATGAAA
ATCAAAAAGATAGCCGGCCTCGACATTTGAAATTACAATGAATGTTTTTC
GGTTTTTATTTTTTACTTTTTATTTGATTTTTTTTTTTTTTTTTTTTTTTTTTT
TTTTTTTTTTTTAAAATTTTTTTTTTTTTTTTTTTTCAAATTTTTTTTTTTTTT
AAAAAAAAAAAAAATTCATTTTTGTATTTAAATTATTTTATAAATAAAAAA
TGATAGTAGTAGAAATTGTTTTTTGGACAAGAATCGTAAAGTCAAAATAT
TCAGTTGATGATGAAATCTTTAAATATTTGGTTAATAGAGCAAAAGTTGT
GTATAACAATTCAGATTATAAAACACTATCTCAAACTGATTTAGAGAAAT
GGGCGATTCAAAACACTCACAGAACAATAACTTCAATTAAAAATCGTTTT
TCAGTCAAAAAGATTGGAGATGAAGAAAAGTTATTTAGAATTTCAAAAAA
TGGTAAAATCCATCTTTATACATGTTCATTTTAATTTATTTTAACTAATT
TTTTAAATTAGGGGAGCTTATTGTTCTTAATGAATTAGAATTCGATAATT
TTCATATTAAAGAAGGAAAGCATTTAAGAAAATCAAAAATGTTTAATCAT
ATCAAAGACAGTGGTTATTATGCAACAAACGAAGAAATTGAAATTTTTCT
TGAAAGCTGCACTCTATGTAAGGAAATTACTGCACAAACCAAACGTAATT
CATATAAAAAAAGAAATATAATTAATAAATTACCGGAAGAAGAAGAAGAA
GAAGAAGAAGAAGAAGAAGAAGAAGAAGAAGAAGAACAAGAAGAAGA
AGTAGAAAAACCGACAATATCGGAAGAAGAAGAAGAAACACCTGCAG
TATCAGAAGAAGAAAAAGAAGAGGAAGAACAAGAAGAAGATAAAGAAAAA
GATAAAGAAAAAAAAATAGAAGAAGATACAGAAACAGGAAAAAAAAAAGA
TGAAGTTATGAATGAACGTCAAGATGGTATTGAAATTAGTGAAAAAACAA
ATGATAAACCAACAGAAAAAACAAAAGTTAAAAAGAATGTTTCACGAGTC
AAAGATATTAATCTTCATAAAAAAGGAAATCCTATTACCCCAAAAAAAAT
AAAAAATTTCAGTCAACAACAATTACGTTTAGCTTCTGGTGTTTCAAATG
AAAAAAGAGCAATAATTACTCTTAAAAAATAATAAAACTTTATTTAATTA
AAAAAATAATTAAAAAGAAAATTAAATATATATTAATTTTTAAATTATTT
GAAATGTTTTTTTCTTGAGTTACTGACTTTTCCATACCGTTATTTGAGCC
ATCGAGCTTTGGATTGTAAATTGTTGATAAAAATTCAGAATCAGATGCAC
CATACTTTGGAGTACACTTTGTTGTTTTAAAATCAAGATTGTTTTGAATG
TTTTCATTCTTACAATAGTGGCACAATGGATCTGTACATGTAAATCTATT
GGATTTCTCTTGTGTAATGTTTGTAATATGATATACATTTTCAAATTTTG
AACAAATGTTGAATTTTTCAATAATTGTTTTACTATTTTTTGTACAATCC
ATTGAAAGAGAAGTAATTTCAACAAAGCTGTCTTCCACTCTAAACAAATA
ACCATCATCAGTTTCATTATATTCTTTATAAAGAATAGACTCTCCAAACT
CGTCATTAATACATTCATTAAGTTTAATTAAGTTTGTTAAATTACGAAAA
TTGTTACATCTTGAATTATTTGTTTCAATGTCATCATAAAAAAGCGCATG
AGTACTATTTGTTTCAACTCTTCCATGAATATTACATTCATCATAAATTA
CGGCGCCTGTCATTGACTTTTCAACACAGTTTTCTTTTTGATACATAACA
ATAAGAAGTTGAAAATATTTTACTTCAATCTGATCATTTTGAAGAACAGA
AGATTTTGATACAATAAAAAAAAAAAAGAAAAAATAATAACGTTGTTGAGT
ACATTATATACAATGAAAATAATAAATGAACAAAAAATAATTTTTTATTA
TAATATATTAAACCCCACCCCACAACCACACCTAAAAAAAAGTTAATACT

TTTTTAAATATAATTTTTTTAATAGTTATTTTAATACGTTTTTAACATAA
AATATATTTTAATTTTTTTTTTAATTTTTTAGAAAATTTTTTTTTCATAA
TTATTTTAATACTTTTTTACAAAAAATATTTTTAAAATTTTAAAAATTTT
TTTTTTATAATTATTTTAATAGCTTAATTACAAAAACAATTTAATAATTT
TTTTAATTTTTTTTTCATAATATTTTTAATAGCTTTTTTACAAATTATTT
TTACGAAAATTTTTGTTATATTTTTTTATAATTATTTTAATACATTTTTA
ACATTAAATAATTATATAATTTTTTAATACAATAATTACAGTTATAAATT
CATATTATTTAAATATTTTTAAATGAGTTTATTTAGAAATTAAGTTTATA
TTTTATTTAATTAGTTTTGACTCTCAGAATGTATAGACACTTTTTAGAAC
ACTGTGCACAAAGATGAAGTTTACATTCTTGGTTCAAATTACATTTAGTT
CCGTTCTTACTACACATGGCACACTTCTTGTTGATTACCGAATCATTGAA
ACATGGAGGGCAAAGTTTAGAATTAATAACAACATTATATATGTTTATGT
TTATTCCTAAACAATTATAGCAAATATTGTTTTGACATCCATCTCTTGTA
CATGAAATATATAAATTTTTTCCTTGACACAAAGAATGACAATTAATACA
CTTTACTTCTAAGAGAGAATAATTTTCTGGATTTGAAAGGGCATCTGTTG
AAGAAACTAAAAAGAAATCAATTTTGGAAGAAGTTTTATTGACTTCTTCT
TTAATAAACTTTTTTAAATCGGCAATTTCATTGGAATTTATTTGGGTAGA
TTCGGATTGAATTTTGTAATAATTGCTATTTACTTTTTCGAAATCGAT

pDM1046 – mCherry (N)

AAGCTTCCGGGGGATCGAGTTTACCACTCCCTATCAGTGATAGAGAAAAG
TGAAAGTCGAGTTTACCACTCCCTATCAGTGATAGAGAAAAGTGAAAGTC
GAGTTTACCACTCCCTATCAGTGATAGAGAAAAGTGAAAGTCGAGTTTAC
CACTCCCTATCAGTGATAGAGAAAAGTGAAAGTCGAGTTTACCACTCCCT
ATCAGTGATAGAGAAAAGTGAAAGTCGAGTTTACCACTCCCTATCAGTGA
TAGAGAAAAGTGAAAGTCGAGTTTACCACTCCCTATCAGTGATAGAGAAA
AGTGAAAGTCGAGCTCGGTACCCAATTTTTTTTTTTTTTTTTTTTTTTTT
TTTTTTTTTTTTTTTCAGATTGCATAAAAAGATTTTTTTTTTTTTTTTTC
TTATTTCTTAAAACAAATAAATTAAATTAAATAAAAAATAAAAATCAGAT
CCAAAAAATGGTTTCAAAAGGTGAAGAAGATAATATGGCAATTATTAAAG
AATTTATGAGATTTAAAGTTCATATGGAAGGTTCAGTTAATGGTCATGAA
TTTGAAATTGAAGGTGAAGGTGAAGGTAGACCATATGAAGGTACACAAAC
AGCAAAATTAAAAGTTACAAAAGGTGGTCCATTACCATTTGCATGGGATA
TTTTATCACCACAATTTATGTATGGTTCAAAAGCATATGTTAAACATCCA
GCAGATATTCCAGATTATTTAAAATTATCATTTCCAGAAGGTTTTAAATG
GGAAAGAGTTATGAATTTTGAAGATGGTGGTGTTGTTACAGTTACACAAG
ATTCATCATTACAAGATGGTGAATTTATTTATAAAGTTAAATTAAGAGGT
ACAAATTTTCCATCAGATGGTCCAGTTATGCAAAAAAAAACAATGGGTTG
GGAAGCATCATCAGAAAGAATGTATCCAGAAGATGGTGCATTAAAAGGTG
AAATTAAACAAAGATTAAAATTAAAAGATGGTGGTCATTATGATGCAGAA
GTTAAAACAACATATAAAGCAAAAAAACCAGTTCAATTACCAGGTGCATA
TAATGTTAATATTAAATTAGATATTACATCACATAATGAAGATTATACAA
TTGTTGAACAATATGAAAGAGCAGAAGGTAGACATTCAACAGGTGGTATG
GATGAATTATATAAAGGTGGTTCAGGAGGTAGTAGATCTTCAACTAGTTA
AATAAATAAATTATTTAATAAATAATAAAAAAACAAATTGTTGTAATAAT
CTAATATTTTCTTTTTTTTTTAATTTTTTTTTTTTAAATCTTAATAATTA
TTAAGTTATTTTAATTTTTTTTTTTTTTTTTTTTTTTTTTTTTTTTTTTT
TTCTATCAAAAAAATCAAATATATTTAAAAAATTTATTATTTACAGATAC
ATTTTGAATGGTGAAGATAAATATATGCATTAGATGTAAAACAGCCAAAG

AGTATGAAAATCAAAAAGATACTCGAGATTTTATTTAATATACTAAATAA
TAAAAAAGTTAAAAAATGATCATTGGATAAATTTTTTATAATTATAAATA
AAGATAATAATTTTTTTTTTAACAAAACTAAAAATAAAAATAATAAAATA
ATTGTTAAAATAGGTTTTTTTTTTTTTTTTTTTTTTTTTAATAAATGGTAT
TTATTAATTTATTTGTTGTGTGTGTTTTTTTTTTTATAATATTTTTTTTT
TTAGCATTGAATTAAGAAGAAATCAAATTGATGCGGCCGCTTAGTTAGCC
TCCCCCATCTCCCGATCCGGACGAGTGCTGGGGCGTCGGTTTCCACTATC
GGCGAGTACTTCTACACAGCCATCGGTCCAAACGGCCGCGCTTCTGCGGG
CGATTTGTGTACGCCCGACAGTCCCGGCTCCGGATCGGACGATTGCGTCG
CATCGACCCTGCGCCCAAGCTGCATCATCGAAATTGCCGTCAACCAAGCT
CTGATAGAGTTGGTCAAGACCAATGCGGAGCATATACGCCCGGAGCCGCG
GCGATCCTGCAAGCTCCGGATGCCTCCGCTCGAAGTAGCGCGTCTGCTGC
TCCATACAAGCCAACCACGGCCTCCAGAAGAAGATGTTGGCGACCTCGTA
TTGGGAATCCCCGAACATCGCCTCGCTCCAGTCAATGACCGCTGTTATGC
GGCCATTGTCCGTCAGGACATTGTTGGAGCCGAAATCCGCGTGCACGAGG
TGCCGGACTTCGGGGCAGTCCTCGGCCCAAAGCATCAGCTCATCGAGAGC
CTGCGCGACGGACGCACTGACGGTGTCGTCCATCACAGTTTGCCAGTGAT
ACACATGGGGATCAGCAATCGCGCATATGAAATCACGCCATGTAGTGTAT
TGACCGATTCCTTGCGGTCCGAATGGGCCGAACCCGCTCGTCTGGCTAAG
ATCGGCCGCAGCGATCGCATCCATGGCCTCCGCGACCGGCTGCAGAACAG
CGGGCAGTTCGGTTTCAGGCAGGTCTTGCAACGTGACACCCTGTGCACGG
CGGGAGATGCAATAGGTCAGGCTCTCGCTAAATTCCCCAATGTCAAGCAC
TTCCGGAATCGGGAGCGCGGCCGATGCAAAGTGCCGATAAACATAACGAT
CTTTGTAGAAACCATCGGCGCAGCTATTTACCCGCAGGACATATCCACGC
CCTCCTACATCGAAGCTGAAAGCACGAGATTCTTCGCCCTCCGAGAGCTG
CATCAGGTCGGAGACGCTGTCGAACTTTTCGATCAGAAACTTCTCGACAG
ACGTCGCGGTGAGTTCAGGCTTTTTCATTAGGTCTTGTTGAGAAATGTTA
AATTGATCCATTTTTTGCTAGCTGTGAAATTAGTTTAAAATACAAATAAA
GAGTTATAATAATATACAGTTGAATAAAAAAAAAAAAAAATGAATTGGAAA
ATTTATTTTTATATGAAGAAAAAAAAAATTTTGAAAAAAAAAAAAAAAAATT
AAAAAAAAAAAAAAAAAAAAAAAAAAATTAAAAATAATTCAACTGTGGGTG
ACCCAAATTTTATTTAAAAAAAAAAAAAAAAATGGATCACTTTTGGGTGGT
TGGAAAAAAAAAAAAAAAAAAAATTGAAAATATAATGTTAGTCATATGATT
AATCATTAATTAATTCTTTTTTTTTTTTTTTTTTATTTTTTATTTTTTTTT
TTTCAATATTCCAATTATGACACACACTAACATCACAAAATACAAAATTT
TGGGATCCCGCTACAGGGCGCGTCAGGTGGCACTTTTCGGGGAAATGTGC
GCGGAACCCCTATTTGTTTATTTTTCTAAATACATTCAAATATGTATCCG
CTCATGAGACAATAACCCTGATAAATGCTTCAATAATATTGAAAAAGGAA
GAGTATGAGTATTCAACATTTCCGTGTCGCCCTTATTCCCTTTTTTGCGG
CATTTTGCCTTCCTGTTTTTGCTCACCCAGAAACGCTGGTGAAAGTAAAA
GATGCTGAAGATCAGTTGGGTGCACGAGTGGGTTACATCGAACTGGATCT
CAACAGCGGTAAGATCCTTGAGAGTTTTCGCCCCGAAGAACGTTTTCCAA
TGATGAGCACTTTTAAAGTTCTGCTATGTGGCGCGGTATTATCCCGTATT
GACGCCGGGCAAGAGCAACTCGGTCGCCGCATACACTATTCTCAGAATGA
CTTGGTTGAGTACTCACCAGTCACAGAAAAGCATCTTACGGATGGCATGA
CAGTAAGAGAATTATGCAGTGCTGCCATAACCATGAGTGATAACACTGCG
GCCAACTTACTTCTGACAACGATCGGAGGACCGAAGGAGCTAACCGCTTT
TTTGCACAACATGGGGGATCATGTAACTCGCCTTGATCGTTGGGAACCGG
AGCTGAATGAAGCCATACCAAACGACGAGCGTGACACCACGATGCCTGTA
GCAATGGCAACAACGTTGCGCAAACTATTAACTGGCGAACTACTTACTCT

AGCTTCCCGGCAACAATTAATAGACTGGATGGAGGCGGATAAAGTTGCAG
GACCACTTCTGCGCTCGGCCCTTCCGGCTGGCTGGTTTATTGCTGATAAA
TCTGGAGCCGGTGAGCGTGGGTCTCGCGGTATCATTGCAGCACTGGGGCC
AGATGGTAAGCCCTCCCGTATCGTAGTTATCTACACGACGGGGAGTCAGG
CAACTATGGATGAACGAAATAGACAGATCGCTGAGATAGGTGCCTCACTG
ATTAAGCATTGGTAACTGTCAGACCAAGTTTACTCATATATACTTTAGAT
TGATTTAAAACTTCATTTTTAATTTAAAAGGATCTAGGTGAAGATCCTTT
TTGATAATCTCATGACCAAAATCCCTTAACGTGAGTTTTCGTTCCACTGA
GCGTCAGACCCCGTAGAAAAGATCAAAGGATCTTCTTGAGATCCTTTTTT
TCTGCGCGTAATCTGCTGCTTGCAAACAAAAAAACCACCGCTACCAGCGG
TGGTTTGTTTGCCGGATCAAGAGCTACCAACTCTTTTTCCGAAGGTAACT
GGCTTCAGCAGAGCGCAGATACCAAATACTGTCCTTCTAGTGTAGCCGTA
GTTAGGCCACCACTTCAAGAACTCTGTAGCACCGCCTACATACCTCGCTC
TGCTAATCCTGTTACCAGTGGCTGCTGCCAGTGGCGATAAGTCGTGTCTT
ACCGGGTTGGACTCAAGACGATAGTTACCGGATAAGGCGCAGCGGTCGGG
CTGAACGGGGGGTTCGTGCACACAGCCCAGCTTGGAGCGAACGACCTACA
CCGAACTGAGATACCTACAGCGTGAGCTATGAGAAAGCGCCACGCTTCCC
GAAGGGAGAAAGGCGGACAGGTATCCGGTAAGCGGCAGGGTCGGAACAGG
AGAGCGCACGAGGGAGCTTCCAGGGGGAAACGCCTGGTATCTTTATAGTC
CTGTCGGGTTTCGCCACCTCTGACTTGAGCGTCGATTTTTGTGATGCTCG
TCAGGGGGGCGGAGCCTATGGAAAAACGCCAGCAACGCGGCCTTTTTACG
GTTCCTGGCCTTTTGCTGGCCTTTTGCTCACATGTTCTTTCCTGCGTTAT
CCCCTGATTCTGTGGATAACCGTATTACCGCCTTTGAGTGAGCTGATACC
GCTCGCCGCAGCCGAACGACCGAGCGCAGCGAGTCAGTGAGCGAGGAAGC
GGAAGAGCGCCCAATACGCAAACCGCCTCTGCCGGCTAAAAAAAATTTTT
ATTTATTTTTATTTATTTTGAATTAAATAGATTACAAATTAATTAATCCC
ATCAAATCTTTAAAAAAAAAATGGTTTAAAAAAACTTGGGTTGGTTAATTA
TTATTTGAAAATTTTAAAACCCAAATTAAAAAAAAAAAAATGGGATTCAAA
AATTTTTTTTTTTTTTTTTTTTTTTTTTTTTTTTTTTTTTTTTCAGATTGCA
TAAAAAGATTTTTTTTTTTTTTTTTTCTTATTTCTTAAAACAAATAAATT
AAATTAAATAAAAAAATAAAAATCAGATCCAAAATGTCAAGATTAGATAAA
AGTAAAGTGATTAACGGCGCATTAGAGCTGCTTAATGGGGTCGGAATCGA
AGGTTTAACAACCCGTAAACTCGCCCAGAAGCTAGGTGTAGAGCAGCCTA
CATTGTATTGGCATGTAAAAAATAAGCGGGCTTTGCTCGACGCCTTACCC
ATTGAGATGTTAGATAGGCACCATACTCACTTTTGCCCTTTAGAAGGGGA
AAGCTGGCAAGATTTTTTACGTAATAACGCTAAAAGTTTTAGATGTGCTT
TACTAAGTCATCGCGATGGAGCAAAAGTACATTTAGGTACACGGCCTACA
GAAAAACAGTATGAAACTCTCGAAAATCAATTAGCCTTTTTATGCCAACA
AGGTTTTTCACTAGAGAATGCATTATATGCACTCAGCGCTGTGGGGCACT
TTACTTTAGGTTGCGTATTGGAAGAACAAGAGCATCAAGTCGCTAAAGAA
GAAAGGGAAACACCTACTACTGATAGTATGCCGCCATTATTACGACAAGC
TATCGAATTATTTGATCGCCAAGGTGCAGAGCCAGCCTTCTTATTCGGCC
TTGAATTGATCATATGCGGATTAGAAAAACAACTTAAATGTGAAAGTGGA
TCACACACTCGTAGACTCTCAACTGCCCCACCAACCGATGTCAGCCTGGG
GGACGAGCTCCACTTAGACGGCGAGGACGTGGCGATGGCGCATGCCGACG
CGCTAGACGATTTCGATCTGGACATGTTGGGGGACGGGGATTCCCCGGGC
CCGGGATTTACCCCCCACGACTCCGCCCCCTACGGCGCTCTGGATATGGC
CGACTTCGAGTTTGAGCAGATGTTTACCGATGCCCTTGGAATTGACGAGT
ACGGTGGTTAATCTAGTACGCGTAAATTGTTCACAGAAAATGTTTCTAAA
TTATTTAATAAATAATAAAAAAACAAATTGTTGTAATAATCTAATATTTT

CTTTTTTTTTTAATTTTTTTTTTTTAAATCTTAATAATTATTAAGTTATT
TTAATTTTTTTTTTTTTTTTTTTTTTTTTTTTTTTTTTTTTTCTATCAAA
AAAATCAAATATATTTAAAAAATTTATTATTTACAGATACATTTTGAATG
GTGAAGATAAATATATGCATTAGATGTAAAACAGCCAAAGAGTATGAAAA
TCAAAAAGATAGCCGGCCTCGACATTTGAAATTACAATGAATGTTTTTCG
GTTTTTATTTTTTACTTTTTATTTGATTTTTTTTTTTTTTTTTTTTTTTT
TTTTTTTTTTAAAATTTTTTTTTTTTTTTTTTCAAATTTTTTTTTTTTTA
AAAAAAAAAAAAATTCATTTTTGTATTTAAATTATTTTATAAATAAAAAAT
GATAGTAGTAGAAATTGTTTTTTGGACAAGAATCGTAAAGTCAAAATATT
CAGTTGATGATGAAATCTTTAAATATTTGGTTAATAGAGCAAAAGTTGTG
TATAACAATTCAGATTATAAAACACTATCTCAAACTGATTTAGAGAAATG
GGCGATTCAAAACACTCACAGAACAATAACTTCAATTAAAAATCGTTTTT
CAGTCAAAAAGATTGGAGATGAAGAAAAGTTATTTAGAATTTCAAAAAAT
GGTAAAATCCATCTTTATACATGTTCATTTTAATTTATTTTAACTAATTT
TTTAAATTAGGGGAGCTTATTGTTCTTAATGAATTAGAATTCGATAATTT
TCATATTAAAGAAGGAAAGCATTTAAGAAAATCAAAAATGTTTAATCATA
TCAAAGACAGTGGTTATTATGCAACAAACGAAGAAATTGAAATTTTTCTT
GAAAGCTGCACTCTATGTAAGGAAATTACTGCACAAACCAAACGTAATTC
ATATAAAAAAAGAAATATAATTAATAAATTACCGGAAGAAGAAGAAGAAG
AAGAAGAAGAAGAAGAAGAAGAAGAAGAAGAAGAAGAACAAGAAGAAGAA
GTAGAAAAACCGACAATATCGGAAGAAGAAGAAGAAGAAACACCTGCAGT
ATCAGAAGAAGAAAAAGAAGAGGAAGAACAAGAAGAAGATAAAGAAAAAG
ATAAAGAAAAAAAAAATAGAAGAAGATACAGAAACAGGAAAAAAAAAAGAT
GAAGTTATGAATGAACGTCAAGATGGTATTGAAATTAGTGAAAAAACAAA
TGATAAACCAACAGAAAAAACAAAAGTTAAAAAGAATGTTTCACGAGTCA
AAGATATTAATCTTCATAAAAAAGGAAATCCTATTACCCCAAAAAAAATA
AAAAATTTCAGTCAACAACAATTACGTTTAGCTTCTGGTGTTTCAAATGA
AAAAAGAGCAATAATTACTCTTAAAAAATAATAAAACTTTATTTAATTAA
AAAAATAATTAAAAAGAAAATTAAATATATATTAATTTTTAAATTATTTG
AAATGTTTTTTTCTTGAGTTACTGACTTTTCCATACCGTTATTTGAGCCA
TCGAGCTTTGGATTGTAAATTGTTGATAAAAATTCAGAATCAGATGCACC
ATACTTTGGAGTACACTTTGTTGTTTTAAAATCAAGATTGTTTTGAATGT
TTTCATTCTTACAATAGTGGCACAATGGATCTGTACATGTAAATCTATTG
GATTTCTCTTGTGTAATGTTTGTAATATGATATACATTTTCAAATTTTGA
ACAAATGTTGAATTTTTCAATAATTGTTTTACTATTTTTTGTACAATCCA
TTGAAAGAGAAGTAATTTCAACAAAGCTGTCTTCCACTCTAAACAAATAA
CCATCATCAGTTTCATTATATTCTTTATAAAGAATAGACTCTCCAAACTC
GTCATTAATACATTCATTAAGTTTAATTAAGTTTGTTAAATTACGAAAAT
TGTTACATCTTGAATTATTTGTTTCAATGTCATCATAAAAAAGCGCATGA
GTACTATTTGTTTCAACTCTTCCATGAATATTACATTCATCATAAATTAC
GGCGCCTGTCATTGACTTTTCAACACAGTTTTCTTTTTGATACATAACAA
TAAGAAGTTGAAAATATTTTACTTCAATCTGATCATTTTGAAGAACAGAA
GATTTTGATACAATAAAAAAAAAAAGAAAAAATAATAACGTTGTTGAGTA
CATTATATACAATGAAAATAATAAATGAACAAAAAATAATTTTTTATTAT
AATATATTAAACCCCACCCCACAACCACACCTAAAAAAAAGTTAATACTT
TTTTAAATATAATTTTTTTAATAGTTATTTTAATACGTTTTTAACATAAA
ATATATTTAATTTTTTTTTTTAATTTTTTAGAAAATTTTTTTTTCATAAT
TATTTTAATACTTTTTTACAAAAAATATTTTTAAAATTTTAAAAATTTTT
TTTTTATAATTATTTTAATAGCTTAATTACAAAAACAATTTAATAATTTT
TTTAATTTTTTTTTCATAATATTTTTAATAGCTTTTTTACAAATTATTTT

TACGAAAATTTTGTTATATTTTTTTATAATTATTTTAATACATTTTTAA
CATTAAATAATTATATAATTTTTTAATACAATAATTACAGTTATAAATTC
ATATTATTTAAATATTTTTAAATGAGTTTATTTAGAAATTAAGTTTATAT
TTTATTTAATTAGTTTTGACTCTCAGAATGTATAGACACTTTTTAGAACA
CTGTGCACAAAGATGAAGTTTACATTCTTGGTTCAAATTACATTTAGTTC
CGTTCTTACTACACATGGCACACTTCTTGTTGATTACCGAATCATTGAAA
CATGGAGGGCAAAGTTTAGAATTAATAACAACATTATATATGTTTATGTT
TATTCCTAAACAATTATAGCAAATATTGTTTTGACATCCATCTCTTGTAC
ATGAAATATATAAATTTTTTCCTTGACACAAAGAATGACAATTAATACAC
TTTACTTCTAAGAGAGAATAATTTTCTGGATTTGAAAGGGCATCTGTTGA
AGAAACTAAAAAGAAATCAATTTTGGAAGAAGTTTTATTGACTTCTTCTT
TAATAAACTTTTTTAAATCGGCAATTTCATTGGAATTTATTTGGGTAGAT
TCGGATTGAATTTTGTAATAATTGCTATTTACTTTTTCGAAATCGAT

pDM1048 – mCherry (C)

AAGCTTCCGGGGGATCGAGTTTACCACTCCCTATCAGTGATAGAGAAAAG
TGAAAGTCGAGTTTACCACTCCCTATCAGTGATAGAGAAAAGTGAAAGTC
GAGTTTACCACTCCCTATCAGTGATAGAGAAAAGTGAAAGTCGAGTTTAC
CACTCCCTATCAGTGATAGAGAAAAGTGAAAGTCGAGTTTACCACTCCCT
ATCAGTGATAGAGAAAAGTGAAAGTCGAGTTTACCACTCCCTATCAGTGA
TAGAGAAAAGTGAAAGTCGAGTTTACCACTCCCTATCAGTGATAGAGAAA
AGTGAAAGTCGAGCTCGGTACCCAATTTTTTTTTTTTTTTTTTTTTTTTT
TTTTTTTTTTTTTTCAGATTGCATAAAAAGATTTTTTTTTTTTTTTTTTC
TTATTTCTTAAAACAAATAAATTAAATTAAATAAAAAATAAAAATCAGAT
CTTCAACTAGTGGTGGTTCAGGAGGTAGTGTTTCAAAAGGTGAAGAAGAT
AATATGGCAATTATTAAAGAATTTATGAGATTTAAAGTTCATATGGAAGG
TTCAGTTAATGGTCATGAATTTGAAATTGAAGGTGAAGGTGAAGGTAGAC
CATATGAAGGTACACAAACAGCAAAATTAAAAGTTACAAAAGGTGGTCCA
TTACCATTTGCATGGGATATTTTATCACCACAATTTATGTATGGTTCAAA
AGCATATGTTAAACATCCAGCAGATATTCCAGATTATTTAAAATTATCAT
TTCCAGAAGGTTTTAAATGGGAAAGAGTTATGAATTTTGAAGATGGTGGT
GTTGTTACAGTTACACAAGATTCATCATTACAAGATGGTGAATTTATTTA
TAAAGTTAAATTAAGAGGTACAAATTTTCCATCAGATGGTCCAGTTATGC
AAAAGAAAACAATGGGTTGGGAAGCATCATCAGAAAGAATGTATCCAGAA
GATGGTGCATTAAAAGGTGAAATTAAACAAAGATTAAAATTAAAAGATGG
TGGTCATTATGATGCAGAAGTTAAAACAACATATAAAGCAAAAAAACCAG
TTCAATTACCAGGTGCATATAATGTTAATATTAAATTAGATATTACATCA
CATAATGAAGATTATACAATTGTTGAACAATATGAAAGAGCAGAAGGTAG
ACATTCAACAGGTGGTATGGATGAATTATATAAATAAGCTAGTTAAATAA
ATAAATTATTTAATAAATAATAAAAAAACAAATTGTTGTAATAATCTAAT
ATTTTCTTTTTTTTTTAATTTTTTTTTTTTAAATCTTAATAATTATTAAG
TTATTTTAATTTTTTTTTTTTTTTTTTTTTTTTTTTTTTTTTTTTTTCTA
TCAAAAAAATCAAATATATTTAAAAAATTTATTATTTACAGATACATTTT
GAATGGTGAAGATAAATATATGCATTAGATGTAAAACAGCCAAAGAGTAT
GAAAATCAAAAAGATACTCGAGATTTTATTTAATATACTAAATAATAAAA
AAGTTAAAAAATGATCATTGGATAAATTTTTTATAATTATAAATAAAGAT
AATAATTTTTTTTTTAACAAAACTAAAAATAAAAATAATAAAATAATTGT
TAAAATAGGTTTTTTTTTTTTTTTTTTTTTTTTAATAAATGGTATTTATT
AATTTATTTGTTGTGTGTGTTTTTTTTTTTATAATATTTTTTTTTTTAGC

ATTGAATTAAGAAGAAATCAAATTGATGCGGCCGCTTAGTTAGCCTCCCC
CATCTCCCGATCCGGACGAGTGCTGGGGCGTCGGTTTCCACTATCGGCGA
GTACTTCTACACAGCCATCGGTCCAAACGGCCGCGCTTCTGCGGGCGATT
TGTGTACGCCCGACAGTCCCGGCTCCGGATCGGACGATTGCGTCGCATCG
ACCCTGCGCCCAAGCTGCATCATCGAAATTGCCGTCAACCAAGCTCTGAT
AGAGTTGGTCAAGACCAATGCGGAGCATATACGCCCGGAGCCGCGGCGAT
CCTGCAAGCTCCGGATGCCTCCGCTCGAAGTAGCGCGTCTGCTGCTCCAT
ACAAGCCAACCACGGCCTCCAGAAGAAGATGTTGGCGACCTCGTATTGGG
AATCCCCGAACATCGCCTCGCTCCAGTCAATGACCGCTGTTATGCGGCCA
TTGTCCGTCAGGACATTGTTGGAGCCGAAATCCGCGTGCACGAGGTGCCG
GACTTCGGGGCAGTCCTCGGCCCAAAGCATCAGCTCATCGAGAGCCTGCG
CGACGGACGCACTGACGGTGTCGTCCATCACAGTTTGCCAGTGATACACA
TGGGGATCAGCAATCGCGCATATGAAATCACGCCATGTAGTGTATTGACC
GATTCCTTGCGGTCCGAATGGGCCGAACCCGCTCGTCTGGCTAAGATCGG
CCGCAGCGATCGCATCCATGGCCTCCGCGACCGGCTGCAGAACAGCGGGC
AGTTCGGTTTCAGGCAGGTCTTGCAACGTGACACCCTGTGCACGGCGGGA
GATGCAATAGGTCAGGCTCTCGCTAAATTCCCCAATGTCAAGCACTTCCG
GAATCGGGAGCGCGGCCGATGCAAAGTGCCGATAAACATAACGATCTTTG
TAGAAACCATCGGCGCAGCTATTTACCCGCAGGACATATCCACGCCCTCC
TACATCGAAGCTGAAAGCACGAGATTCTTCGCCCTCCGAGAGCTGCATCA
GGTCGGAGACGCTGTCGAACTTTTCGATCAGAAACTTCTCGACAGACGTC
GCGGTGAGTTCAGGCTTTTTCATTAGGTCTTGTTGAGAAATGTTAAATTG
ATCCATTTTTTGCTAGCTGTGAAATTAGTTTAAAATACAAATAAAGAGTT
ATAATAATATACAGTTGAATAAAAAAAAAAAAAATGAATTGGAAAATTTA
TTTTTATATGAAGAAAAAAAAATTTTGAAAAAAAAAAAAAAAATTAAAAA
AAAAAAAAAAAAAAAAAAAAATTAAAAATAATTCAACTGTGGGTGACCCA
AATTTATTTAAAAAAAAAAAAAAAAATGGATCACTTTTGGGTGGTTGGAA
AAAAAAAAAAAAAAAAATTGAAAATATAATGTTAGTCATATGATTAATCA
TTAATTAATTCTTTTTTTTTTTTTTTTTATTTTTTATTTTTTTTTTTTCA
ATATTCCAATTATGACACACACTAACATCACAAAATACAAAATTTTGGGA
TCCCGCTACAGGGCGCGTCAGGTGGCACTTTTCGGGGAAATGTGCGCGGA
ACCCCTATTTGTTTATTTTTCTAAATACATTCAAATATGTATCCGCTCAT
GAGACAATAACCCTGATAAATGCTTCAATAATATTGAAAAAGGAAGAGTA
TGAGTATTCAACATTTCCGTGTCGCCCTTATTCCCTTTTTTGCGGCATTT
TGCCTTCCTGTTTTTGCTCACCCAGAAACGCTGGTGAAAGTAAAAGATGC
TGAAGATCAGTTGGGTGCACGAGTGGGTTACATCGAACTGGATCTCAACA
GCGGTAAGATCCTTGAGAGTTTTCGCCCCGAAGAACGTTTTCCAATGATG
AGCACTTTTAAAGTTCTGCTATGTGGCGCGGTATTATCCCGTATTGACGC
CGGGCAAGAGCAACTCGGTCGCCGCATACACTATTCTCAGAATGACTTGG
TTGAGTACTCACCAGTCACAGAAAAGCATCTTACGGATGGCATGACAGTA
AGAGAATTATGCAGTGCTGCCATAACCATGAGTGATAACACTGCGGCCAA
CTTACTTCTGACAACGATCGGAGGACCGAAGGAGCTAACCGCTTTTTTGC
ACAACATGGGGGATCATGTAACTCGCCTTGATCGTTGGGAACCGGAGCTG
AATGAAGCCATACCAAACGACGAGCGTGACACCACGATGCCTGTAGCAAT
GGCAACAACGTTGCGCAAACTATTAACTGGCGAACTACTTACTCTAGCTT
CCCGGCAACAATTAATAGACTGGATGGAGGCGGATAAAGTTGCAGGACCA
CTTCTGCGCTCGGCCCTTCCGGCTGGCTGGTTTATTGCTGATAAATCTGG
AGCCGGTGAGCGTGGGTCTCGCGGTATCATTGCAGCACTGGGGCCAGATG
GTAAGCCCTCCCGTATCGTAGTTATCTACACGACGGGGAGTCAGGCAACT
ATGGATGAACGAAATAGACAGATCGCTGAGATAGGTGCCTCACTGATTAA

GCATTGGTAACTGTCAGACCAAGTTTACTCATATATACTTTAGATTGATT
TAAAACTTCATTTTTAATTTAAAAGGATCTAGGTGAAGATCCTTTTTGAT
AATCTCATGACCAAAATCCCTTAACGTGAGTTTTCGTTCCACTGAGCGTC
AGACCCCGTAGAAAAGATCAAAGGATCTTCTTGAGATCCTTTTTTTCTGC
GCGTAATCTGCTGCTTGCAAACAAAAAAACCACCGCTACCAGCGGTGGTT
TGTTTGCCGGATCAAGAGCTACCAACTCTTTTTCCGAAGGTAACTGGCTT
CAGCAGAGCGCAGATACCAAATACTGTCCTTCTAGTGTAGCCGTAGTTAG
GCCACCACTTCAAGAACTCTGTAGCACCGCCTACATACCTCGCTCTGCTA
ATCCTGTTACCAGTGGCTGCTGCCAGTGGCGATAAGTCGTGTCTTACCGG
GTTGGACTCAAGACGATAGTTACCGGATAAGGCGCAGCGGTCGGGCTGAA
CGGGGGGTTCGTGCACACAGCCCAGCTTGGAGCGAACGACCTACACCGAA
CTGAGATACCTACAGCGTGAGCTATGAGAAAGCGCCACGCTTCCCGAAGG
GAGAAAGGCGGACAGGTATCCGGTAAGCGGCAGGGTCGGAACAGGAGAGC
GCACGAGGGAGCTTCCAGGGGGAAACGCCTGGTATCTTTATAGTCCTGTC
GGGTTTCGCCACCTCTGACTTGAGCGTCGATTTTTGTGATGCTCGTCAGG
GGGGCGGAGCCTATGGAAAAACGCCAGCAACGCGGCCTTTTTACGGTTCC
TGGCCTTTTGCTGGCCTTTTGCTCACATGTTCTTTCCTGCGTTATCCCCT
GATTCTGTGGATAACCGTATTACCGCCTTTGAGTGAGCTGATACCGCTCG
CCGCAGCCGAACGACCGAGCGCAGCGAGTCAGTGAGCGAGGAAGCGGAAG
AGCGCCCAATACGCAAACCGCCTCTGCCGGCTAAAAAAAATTTTTATTTA
TTTTTATTTATTTTGAATTAAATAGATTACAAATTAATTAATCCCATCAA
ATCTTTAAAAAAAAATGGTTTAAAAAAACTTGGGTTGGTTAATTATTATT
TGAAAATTTTAAAACCCAAATTAAAAAAAAAAAATGGGATTCAAAAATTT
TTTTTTTTTTTTTTTTTTTTTTTTTTTTTTTTTTTTCAGATTGCATAAAA
AGATTTTTTTTTTTTTTTTTTCTTATTTCTTAAAACAAATAAATTAAATT
AAATAAAAAATAAAAATCAGATCCAAAATGTCAAGATTAGATAAAAGTAA
AGTGATTAACGGCGCATTAGAGCTGCTTAATGGGGTCGGAATCGAAGGTT
TAACAACCCGTAAACTCGCCCAGAAGCTAGGTGTAGAGCAGCCTACATTG
TATTGGCATGTAAAAAATAAGCGGGCTTTGCTCGACGCCTTACCCATTGA
GATGTTAGATAGGCACCATACTCACTTTTGCCCTTTAGAAGGGGAAAGCT
GGCAAGATTTTTTACGTAATAACGCTAAAAGTTTTAGATGTGCTTTACTA
AGTCATCGCGATGGAGCAAAAGTACATTTAGGTACACGGCCTACAGAAAA
ACAGTATGAAACTCTCGAAAATCAATTAGCCTTTTTATGCCAACAAGGTT
TTTCACTAGAGAATGCATTATATGCACTCAGCGCTGTGGGGCACTTTACT
TTAGGTTGCGTATTGGAAGAACAAGAGCATCAAGTCGCTAAAGAAGAAAG
GGAAACACCTACTACTGATAGTATGCCGCCATTATTACGACAAGCTATCG
AATTATTTGATCGCCAAGGTGCAGAGCCAGCCTTCTTATTCGGCCTTGAA
TTGATCATATGCGGATTAGAAAAACAACTTAAATGTGAAAGTGGATCACA
CACTCGTAGACTCTCAACTGCCCCACCAACCGATGTCAGCCTGGGGGACG
AGCTCCACTTAGACGGCGAGGACGTGGCGATGGCGCATGCCGACGCGCTA
GACGATTTCGATCTGGACATGTTGGGGGACGGGGATTCCCCGGGCCCGGG
ATTTACCCCCCACGACTCCGCCCCCTACGGCGCTCTGGATATGGCCGACT
TCGAGTTTGAGCAGATGTTTACCGATGCCCTTGGAATTGACGAGTACGGT
GGTTAATCTAGTACGCGTAAATTGTTCACAGAAAATGTTTCTAAATTATT
TAATAAATAATAAAAAAACAAATTGTTGTAATAATCTAATATTTTCTTTT
TTTTTTAATTTTTTTTTTTTTAAATCTTAATAATTATTAAGTTATTTTAAT
TTTTTTTTTTTTTTTTTTTTTTTTTTTTTTTTTTTTTTCTATCAAAAAAT
CAAATATATTTAAAAAATTTATTATTTACAGATACATTTTGAATGGTGAA
GATAAATATATGCATTAGATGTAAAACAGCCAAAGAGTATGAAAATCAAA
AAGATAGCCGGCCTCGACATTTGAAATTACAATGAATGTTTTTCGGTTTT

TATTTTTTACTTTTTATTTGATTTTTTTTTTTTTTTTTTTTTTTTTTTTTT
TTTTTAAAATTTTTTTTTTTTTTTTTTTCAAATTTTTTTTTTTTTTAAAAAA
AAAAAAATTCATTTTTGTATTTAAATTATTTTATAAATAAAAAATGATAG
TAGTAGAAATTGTTTTTTGGACAAGAATCGTAAAGTCAAAATATTCAGTT
GATGATGAAATCTTTAAATATTTGGTTAATAGAGCAAAAGTTGTGTATAA
CAATTCAGATTATAAAACACTATCTCAAACTGATTTAGAGAAATGGGCGA
TTCAAAACACTCACAGAACAATAACTTCAATTAAAAATCGTTTTTCAGTC
AAAAAGATTGGAGATGAAGAAAAGTTATTTAGAATTTCAAAAAATGGTAA
AATCCATCTTTATACATGTTCATTTTAATTTATTTTAACTAATTTTTTAA
ATTAGGGGAGCTTATTGTTCTTAATGAATTAGAATTCGATAATTTTCATA
TTAAAGAAGGAAAGCATTTAAGAAAATCAAAAATGTTTAATCATATCAAA
GACAGTGGTTATTATGCAACAAACGAAGAAATTGAAATTTTTCTTGAAAG
CTGCACTCTATGTAAGGAAATTACTGCACAAACCAAACGTAATTCATATA
AAAAAAGAAATATAATTAATAAATTACCGGAAGAAGAAGAAGAAGAAGAA
GAAGAAGAAGAAGAAGAAGAAGAAGAAGAAGAACAAGAAGAAGAAGTAGA
AAAACCGACAATATCGGAAGAAGAAGAAGAAGAAACACCTGCAGTATCAG
AAGAAGAAAAAGAAGAGGAAGAACAAGAAGAAGATAAAGAAAAAGATAAA
GAAAAAAAAATAGAAGAAGATACAGAAACAGGAAAAAAAAAAAGATGAAGT
TATGAATGAACGTCAAGATGGTATTGAAATTAGTGAAAAAACAAATGATA
AACCAACAGAAAAAACAAAAGTTAAAAAGAATGTTTCACGAGTCAAAGAT
ATTAATCTTCATAAAAAAGGAAATCCTATTACCCCAAAAAAAATAAAAAA
TTTCAGTCAACAACAATTACGTTTAGCTTCTGGTGTTTCAAATGAAAAAA
GAGCAATAATTACTCTTAAAAAATAATAAAACTTTATTTAATTAAAAAAA
TAATTAAAAAGAAAATTAAATATATATTAATTTTTAAATTATTTGAAATG
TTTTTTTCTTGAGTTACTGACTTTTCCATACCGTTATTTGAGCCATCGAG
CTTTGGATTGTAAATTGTTGATAAAAATTCAGAATCAGATGCACCATACT
TTGGAGTACACTTTGTTGTTTTAAAATCAAGATTGTTTTGAATGTTTTCA
TTCTTACAATAGTGGCACAATGGATCTGTACATGTAAATCTATTGGATTT
CTCTTGTGTAATGTTTGTAATATGATATACATTTTCAAATTTTGAACAAA
TGTTGAATTTTTCAATAATTGTTTTACTATTTTTTGTACAATCCATTGAA
AGAGAAGTAATTTCAACAAAGCTGTCTTCCACTCTAAACAAATAACCATC
ATCAGTTTCATTATATTCTTTATAAAGAATAGACTCTCCAAACTCGTCAT
TAATACATTCATTAAGTTTAATTAAGTTTGTTAAATTACGAAAATTGTTA
CATCTTGAATTATTTGTTTCAATGTCATCATAAAAAAGCGCATGAGTACT
ATTTGTTTCAACTCTTCCATGAATATTACATTCATCATAAATTACGGCGC
CTGTCATTGACTTTTCAACACAGTTTTCTTTTTGATACATAACAATAAGA
AGTTGAAAATATTTTACTTCAATCTGATCATTTTGAAGAACAGAAGATTT
TGATACAATAAAAAAAAAAAAGAAAAAATAATAACGTTGTTGAGTACATTA
TATACAATGAAAATAATAAATGAACAAAAAATAATTTTTTATTATAATAT
ATTAAACCCCACCCCACAACCACACCTAAAAAAAAGTTAATACTTTTTTA
AATATAATTTTTTTAATAGTTATTTTAATACGTTTTTAACATAAAATATA
TTTTAATTTTTTTTTTAATTTTTTAGAAAATTTTTTTTCATAATTATTT
TAATACTTTTTTACAAAAAATATTTTTAAAATTTTAAAAATTTTTTTTTT
ATAATTATTTTAATAGCTTAATTACAAAAACAATTTAATAATTTTTTTAA
TTTTTTTTTCATAATATTTTTAATAGCTTTTTTACAAATTATTTTTACGA
AAATTTTTGTTATATTTTTTTATAATTATTTTAATACATTTTTAACATTA
AATAATTATATAATTTTTTAATACAATAATTACAGTTATAAATTCATATT
ATTTAAATATTTTTAAATGAGTTTATTTAGAAATTAAGTTTATATTTTAT
TTAATTAGTTTTGACTCTCAGAATGTATAGACACTTTTTAGAACACTGTG
CACAAAGATGAAGTTTACATTCTTGGTTCAAATTACATTTAGTTCCGTTC

TTACTACACATGGCACACTTCTTGTTGATTACCGAATCATTGAAACATGG
AGGGCAAAGTTTAGAATTAATAACAACATTATATATGTTTATGTTTATTC
CTAAACAATTATAGCAAATATTGTTTTGACATCCATCTCTTGTACATGAA
ATATATAAATTTTTTCCTTGACACAAAGAATGACAATTAATACACTTTAC
TTCTAAGAGAGAATAATTTTCTGGATTTGAAAGGGCATCTGTTGAAGAAA
CTAAAAAGAAATCAATTTTGGAAGAAGTTTATTGACTTCTTCTTTAATA
AACTTTTTTAAATCGGCAATTTCATTGGAATTTATTTGGGTAGATTCGGA
TTGAATTTTGTAATAATTGCTATTTACTTTTTCGAAATCGAT

pPI450 – mNeon (N)

AAGCTTCCGGGGGATCGAGTTTACCACTCCCTATCAGTGATAGAGAAAAG
TGAAAGTCGAGTTTACCACTCCCTATCAGTGATAGAGAAAAGTGAAAGTC
GAGTTTACCACTCCCTATCAGTGATAGAGAAAAGTGAAAGTCGAGTTTAC
CACTCCCTATCAGTGATAGAGAAAAGTGAAAGTCGAGTTTACCACTCCCT
ATCAGTGATAGAGAAAAGTGAAAGTCGAGTTTACCACTCCCTATCAGTGA
TAGAGAAAAGTGAAAGTCGAGTTTACCACTCCCTATCAGTGATAGAGAAA
AGTGAAAGTCGAGCTCGGTACCCAATTTTTTTTTTTTTTTTTTTTTTTTT
TTTTTTTTTTTTTTCAGATTGCATAAAAAGATTTTTTTTTTTTTTTTTC
TTATTTCTTAAAACAAATAAATTAAATTAAATAAAAAATAAAAATCAGAT
CCAAAAAATGGTGAGTAAAGGTGAAGAAGATAATATGGCATCGTTACCAG
CTACACATGAGTTACATATATTCGGTAGCATTAATGGTGTTGATTTTGAT
ATGGTGGGACAAGGTACCGGTAATCCTAATGATGGTTACGAAGAACTAAA
TTTAAAATCGACTAAAGGTGACTTACAATTTTCTCCATGGATTTTAGTGC
CACATATAGGGTATGGTTTTCATCAATACTTACCATATCCAGATGGTATG
TCACCATTTCAAGCTGCAATGGTTGATGGATCAGGTTATCAAGTTCATAG
AACAATGCAATTTGAAGATGGTGCTTCATTAACTGTTAATTATAGATACA
CATATGAAGGCTCACATATTAAAGGTGAAGCTCAAGTTAAAGGTACTGGT
TTCCCAGCCGATGGCCCAGTTATGACAAATAGTTTAACAGCAGCAGATTG
GTGTAGATCCAAAAAAACTTATCCAAATGATAAAACAATTATTTCAACTT
TTAAATGGTCATATACAACCGGTAATGGTAAACGTTATCGTTCAACAGCC
CGTACAACATATACTTTTGCTAAACCAATGGCAGCTAATTATTTAAAAAA
TCAACCAATGTATGTTTTTCGTAAAACAGAGTTAAAACATTCAAAAACAG
AACTTAATTTTAAAGAATGGCAAAAAGCATTTACAGACGTTATGGGTATG
GATGAACTTTATAAGGGTGGTTCAGGAGGTAGTAGATCTTCAACTAGTTA
AATAAATAAATTATTTAATAAATAATAAAAAAACAAATTGTTGTAATAAT
CTAATATTTTCTTTTTTTTTTAATTTTTTTTTTTTAAATCTTAATAATTA
TTAAGTTATTTTAATTTTTTTTTTTTTTTTTTTTTTTTTTTTTTTTTTTT
TTCTATCAAAAAAATCAAATATATTTAAAAAATTTATTATTTACAGATAC
ATTTTGAATGGTGAAGATAAATATATGCATTAGATGTAAAACAGCCAAAG
AGTATGAAAATCAAAAAGATACTCGAGATTTTATTTAATATACTAAATAA
TAAAAAAGTTAAAAAATGATCATTGGATAAATTTTTTATAATTATAAATA
AAGATAATAATTTTTTTTTTAACAAAACTAAAAATAAAAATAATAAAATA
ATTGTTAAAATAGGTTTTTTTTTTTTTTTTTTTTTTTTTAATAAATGGTAT
TTATTAATTTATTTGTTGTGTGTGTTTTTTTTTTTATAATATTTTTTTTT
TTAGCATTGAATTAAGAAGAAATCAAATTGATGCGGCCGCTTAGTTAGCC
TCCCCCATCTCCCGATCCGGACGAGTGCTGGGGCGTCGGTTTCCACTATC
GGCGAGTACTTCTACACAGCCATCGGTCCAAACGGCCGCGCTTCTGCGGG
CGATTTGTGTACGCCCGACAGTCCCGGCTCCGGATCGGACGATTGCGTCG
CATCGACCCTGCGCCCAAGCTGCATCATCGAAATTGCCGTCAACCAAGCT

CTGATAGAGTTGGTCAAGACCAATGCGGAGCATATACGCCCGGAGCCGCG
GCGATCCTGCAAGCTCCGGATGCCTCCGCTCGAAGTAGCGCGTCTGCTGC
TCCATACAAGCCAACCACGGCCTCCAGAAGAAGATGTTGGCGACCTCGTA
TTGGGAATCCCCGAACATCGCCTCGCTCCAGTCAATGACCGCTGTTATGC
GGCCATTGTCCGTCAGGACATTGTTGGAGCCGAAATCCGCGTGCACGAGG
TGCCGGACTTCGGGGCAGTCCTCGGCCCAAAGCATCAGCTCATCGAGAGC
CTGCGCGACGGACGCACTGACGGTGTCGTCCATCACAGTTTGCCAGTGAT
ACACATGGGGATCAGCAATCGCGCATATGAAATCACGCCATGTAGTGTAT
TGACCGATTCCTTGCGGTCCGAATGGGCCGAACCCGCTCGTCTGGCTAAG
ATCGGCCGCAGCGATCGCATCCATGGCCTCCGCGACCGGCTGCAGAACAG
CGGGCAGTTCGGTTTCAGGCAGGTCTTGCAACGTGACACCCTGTGCACGG
CGGGAGATGCAATAGGTCAGGCTCTCGCTAAATTCCCCAATGTCAAGCAC
TTCCGGAATCGGGAGCGCGGCCGATGCAAAGTGCCGATAAACATAACGAT
CTTTGTAGAAACCATCGGCGCAGCTATTTACCCGCAGGACATATCCACGC
CCTCCTACATCGAAGCTGAAAGCACGAGATTCTTCGCCCTCCGAGAGCTG
CATCAGGTCGGAGACGCTGTCGAACTTTTCGATCAGAAACTTCTCGACAG
ACGTCGCGGTGAGTTCAGGCTTTTTCATTAGGTCTTGTTGAGAAATGTTA
AATTGATCCATTTTTTGCTAGCTGTGAAATTAGTTTAAAATACAAATAAA
GAGTTATAATAATATACAGTTGAATAAAAAAAAAAAAAAATGAATTGGAAA
ATTTATTTTTATATGAAGAAAAAAAAAATTTTGAAAAAAAAAAAAAAAAATT
AAAAAAAAAAAAAAAAAAAAAAAAAATTAAAAATAATTCAACTGTGGGTG
ACCCAAATTTTATTTAAAAAAAAAAAAAAAAATGGATCACTTTTGGGTGGT
TGGAAAAAAAAAAAAAAAAAAAATTGAAAATATAATGTTAGTCATATGATT
AATCATTAATTAATTCTTTTTTTTTTTTTTTTTATTTTTTATTTTTTTTT
TTTCAATATTCCAATTATGACACACACTAACATCACAAAATACAAAATTT
TGGGATCCCGCTACAGGGCGCGTCAGGTGGCACTTTTCGGGGAAATGTGC
GCGGAACCCCTATTTGTTTATTTTTCTAAATACATTCAAATATGTATCCG
CTCATGAGACAATAACCCTGATAAATGCTTCAATAATATTGAAAAAGGAA
GAGTATGAGTATTCAACATTTCCGTGTCGCCCTTATTCCCTTTTTTGCGG
CATTTTGCCTTCCTGTTTTTGCTCACCCAGAAACGCTGGTGAAAGTAAAA
GATGCTGAAGATCAGTTGGGTGCACGAGTGGGTTACATCGAACTGGATCT
CAACAGCGGTAAGATCCTTGAGAGTTTTCGCCCCGAAGAACGTTTTCCAA
TGATGAGCACTTTTAAAGTTCTGCTATGTGGCGCGGTATTATCCCGTATT
GACGCCGGGCAAGAGCAACTCGGTCGCCGCATACACTATTCTCAGAATGA
CTTGGTTGAGTACTCACCAGTCACAGAAAAGCATCTTACGGATGGCATGA
CAGTAAGAGAATTATGCAGTGCTGCCATAACCATGAGTGATAACACTGCG
GCCAACTTACTTCTGACAACGATCGGAGGACCGAAGGAGCTAACCGCTTT
TTTGCACAACATGGGGGATCATGTAACTCGCCTTGATCGTTGGGAACCGG
AGCTGAATGAAGCCATACCAAACGACGAGCGTGACACCACGATGCCTGTA
GCAATGGCAACAACGTTGCGCAAACTATTAACTGGCGAACTACTTACTCT
AGCTTCCCGGCAACAATTAATAGACTGGATGGAGGCGGATAAAGTTGCAG
GACCACTTCTGCGCTCGGCCCTTCCGGCTGGCTGGTTTATTGCTGATAAA
TCTGGAGCCGGTGAGCGTGGGTCTCGCGGTATCATTGCAGCACTGGGGCC
AGATGGTAAGCCCTCCCGTATCGTAGTTATCTACACGACGGGGAGTCAGG
CAACTATGGATGAACGAAATAGACAGATCGCTGAGATAGGTGCCTCACTG
ATTAAGCATTGGTAACTGTCAGACCAAGTTTACTCATATATACTTTAGAT
TGATTTAAAACTTCATTTTTAATTTAAAAGGATCTAGGTGAAGATCCTTT
TTGATAATCTCATGACCAAAATCCCTTAACGTGAGTTTTCGTTCCACTGA
GCGTCAGACCCCGTAGAAAAGATCAAAGGATCTTCTTGAGATCCTTTTTT
TCTGCGCGTAATCTGCTGCTTGCAAACAAAAAAACCACCGCTACCAGCGG

TGGTTTGTTTGCCGGATCAAGAGCTACCAACTCTTTTTCCGAAGGTAACT
GGCTTCAGCAGAGCGCAGATACCAAATACTGTCCTTCTAGTGTAGCCGTA
GTTAGGCCACCACTTCAAGAACTCTGTAGCACCGCCTACATACCTCGCTC
TGCTAATCCTGTTACCAGTGGCTGCTGCCAGTGGCGATAAGTCGTGTCTT
ACCGGGTTGGACTCAAGACGATAGTTACCGGATAAGGCGCAGCGGTCGGG
CTGAACGGGGGGTTCGTGCACACAGCCCAGCTTGGAGCGAACGACCTACA
CCGAACTGAGATACCTACAGCGTGAGCTATGAGAAAGCGCCACGCTTCCC
GAAGGGAGAAAGGCGGACAGGTATCCGGTAAGCGGCAGGGTCGGAACAGG
AGAGCGCACGAGGGAGCTTCCAGGGGGAAACGCCTGGTATCTTTATAGTC
CTGTCGGGTTTCGCCACCTCTGACTTGAGCGTCGATTTTTGTGATGCTCG
TCAGGGGGGCGGAGCCTATGGAAAAACGCCAGCAACGCGGCCTTTTTACG
GTTCCTGGCCTTTTGCTGGCCTTTTGCTCACATGTTCTTTCCTGCGTTAT
CCCCTGATTCTGTGGATAACCGTATTACCGCCTTTGAGTGAGCTGATACC
GCTCGCCGCAGCCGAACGACCGAGCGCAGCGAGTCAGTGAGCGAGGAAGC
GGAAGAGCGCCCAATACGCAAACCGCCTCTGCCGGCTAAAAAAAATTTTT
ATTTATTTTTATTTATTTTGAATTAAATAGATTACAAATTAATTAATCCC
ATCAAATCTTTAAAAAAAAATGGTTTAAAAAAACTTGGGTTGGTTAATTA
TTATTTGAAAATTTTAAAACCCAAATTAAAAAAAAAAAATGGGATTCAAA
AATTTTTTTTTTTTTTTTTTTTTTTTTTTTTTTTTTTTTTTCAGATTGCA
TAAAAAGATTTTTTTTTTTTTTTTTTCTTATTTCTTAAAACAAATAAATT
AAATTAAATAAAAAATAAAAATCAGATCCAAAATGTCAAGATTAGATAAA
AGTAAAGTGATTAACGGCGCATTAGAGCTGCTTAATGGGGTCGGAATCGA
AGGTTTAACAACCCGTAAACTCGCCCAGAAGCTAGGTGTAGAGCAGCCTA
CATTGTATTGGCATGTAAAAAATAAGCGGGCTTTGCTCGACGCCTTACCC
ATTGAGATGTTAGATAGGCACCATACTCACTTTTGCCCTTTAGAAGGGGA
AAGCTGGCAAGATTTTTTACGTAATAACGCTAAAAGTTTTAGATGTGCTT
TACTAAGTCATCGCGATGGAGCAAAAGTACATTTAGGTACACGGCCTACA
GAAAAACAGTATGAAACTCTCGAAAATCAATTAGCCTTTTTATGCCAACA
AGGTTTTTCACTAGAGAATGCATTATATGCACTCAGCGCTGTGGGGCACT
TTACTTTAGGTTGCGTATTGGAAGAACAAGAGCATCAAGTCGCTAAAGAA
GAAAGGGAAACACCTACTACTGATAGTATGCCGCCATTATTACGACAAGC
TATCGAATTATTTGATCGCCAAGGTGCAGAGCCAGCCTTCTTATTCGGCC
TTGAATTGATCATATGCGGATTAGAAAAACAACTTAAATGTGAAAGTGGA
TCACACACTCGTAGACTCTCAACTGCCCCACCAACCGATGTCAGCCTGGG
GGACGAGCTCCACTTAGACGGCGAGGACGTGGCGATGGCGCATGCCGACG
CGCTAGACGATTTCGATCTGGACATGTTGGGGGACGGGGATTCCCCGGGC
CCGGGATTTACCCCCCACGACTCCGCCCCCTACGGCGCTCTGGATATGGC
CGACTTCGAGTTTGAGCAGATGTTTACCGATGCCCTTGGAATTGACGAGT
ACGGTGGTTAATCTAGTACGCGTAAATTGTTCACAGAAAATGTTTCTAAA
TTATTTAATAAATAATAAAAAAACAAATTGTTGTAATAATCTAATATTTT
CTTTTTTTTTTAATTTTTTTTTTTTAAATCTTAATAATTATTAAGTTATT
TTAATTTTTTTTTTTTTTTTTTTTTTTTTTTTTTTTTTTTCTATCAAA
AAAATCAAATATATTTAAAAAATTTATTATTTACAGATACATTTTGAATG
GTGAAGATAAATATATGCATTAGATGTAAAACAGCCAAAGAGTATGAAAA
TCAAAAAGATAGCCGGCCTCGACATTTGAAATTACAATGAATGTTTTTCG
GTTTTTATTTTTTACTTTTTATTTGATTTTTTTTTTTTTTTTTTTTTTTT
TTTTTTTTTTAAAATTTTTTTTTTTTTTTTTTCAAATTTTTTTTTTTTTA
AAAAAAAAAAAATTCATTTTTGTATTTAAATTATTTTATAAATAAAAAAT
GATAGTAGTAGAAATTGTTTTTTGGACAAGAATCGTAAAGTCAAAATATT
CAGTTGATGATGAAATCTTTAAATATTTGGTTAATAGAGCAAAAGTTGTG

TATAACAATTCAGATTATAAAACACTATCTCAAACTGATTTAGAGAAATG
GGCGATTCAAAACACTCACAGAACAATAACTTCAATTAAAAATCGTTTTT
CAGTCAAAAAGATTGGAGATGAAGAAAAGTTATTTAGAATTTCAAAAAAT
GGTAAAATCCATCTTTATACATGTTCATTTTAATTTATTTTAACTAATTT
TTTAAATTAGGGGAGCTTATTGTTCTTAATGAATTAGAATTCGATAATTT
TCATATTAAAGAAGGAAAGCATTTAAGAAAATCAAAAATGTTTAATCATA
TCAAAGACAGTGGTTATTATGCAACAAACGAAGAAATTGAAATTTTTCTT
GAAAGCTGCACTCTATGTAAGGAAATTACTGCACAAACCAAACGTAATTC
ATATAAAAAAAGAAATATAATTAATAAATTACCGGAAGAAGAAGAAGAAG
AAGAAGAAGAAGAAGAAGAAGAAGAAGAAGAAGAAGAACAAGAAGAAGAA
GTAGAAAAACCGACAATATCGGAAGAAGAAGAAGAAGAAACACCTGCAGT
ATCAGAAGAAGAAAAAGAAGAGGAAGAACAAGAAGAAGATAAAGAAAAAG
ATAAAGAAAAAAAAATAGAAGAAGATACAGAAACAGGAAAAAAAAAAGAT
GAAGTTATGAATGAACGTCAAGATGGTATTGAAATTAGTGAAAAAACAAA
TGATAAACCAACAGAAAAAACAAAAGTTAAAAAGAATGTTTCACGAGTCA
AAGATATTAATCTTCATAAAAAAGGAAATCCTATTACCCCAAAAAAAATA
AAAAATTTCAGTCAACAACAATTACGTTTAGCTTCTGGTGTTTCAAATGA
AAAAAGAGCAATAATTACTCTTAAAAAATAATAAAACTTTATTTAATTAA
AAAAATAATTAAAAAGAAAATTAAATATATATTAATTTTTAAATTATTTG
AAATGTTTTTTTCTTGAGTTACTGACTTTTCCATACCGTTATTTGAGCCA
TCGAGCTTTGGATTGTAAATTGTTGATAAAAATTCAGAATCAGATGCACC
ATACTTTGGAGTACACTTTGTTGTTTTAAAATCAAGATTGTTTTGAATGT
TTTCATTCTTACAATAGTGGCACAATGGATCTGTACATGTAAATCTATTG
GATTTCTCTTGTGTAATGTTTGTAATATGATATACATTTTCAAATTTTGA
ACAAATGTTGAATTTTTCAATAATTGTTTTACTATTTTTTGTACAATCCA
TTGAAAGAGAAGTAATTTCAACAAAGCTGTCTTCCACTCTAAACAAATAA
CCATCATCAGTTTCATTATATTCTTTATAAAGAATAGACTCTCCAAACTC
GTCATTAATACATTCATTAAGTTTAATTAAGTTTGTTAAATTACGAAAAT
TGTTACATCTTGAATTATTTGTTTCAATGTCATCATAAAAAAGCGCATGA
GTACTATTTGTTTCAACTCTTCCATGAATATTACATTCATCATAAATTAC
GGCGCCTGTCATTGACTTTTCAACACAGTTTTCTTTTTGATACATAACAA
TAAGAAGTTGAAAATATTTTACTTCAATCTGATCATTTTGAAGAACAGAA
GATTTTGATACAATAAAAAAAAAAAGAAAAAATAATAACGTTGTTGAGTA
CATTATATACAATGAAAATAATAAATGAACAAAAAATAATTTTTTATTAT
AATATATTAAACCCCACCCCACAACCACACCTAAAAAAAAGTTAATACTT
TTTTAAATATAATTTTTTTAATAGTTATTTTAATACGTTTTTAACATAAA
ATATATTTTAATTTTTTTTTTAATTTTTTAGAAAATTTTTTTTTCATAAT
TATTTTAATACTTTTTTACAAAAAATATTTTTAAAATTTTAAAAATTTTT
TTTTTATAATTATTTTAATAGCTTAATTACAAAAACAATTTAATAATTTT
TTTAATTTTTTTTTCATAATATTTTTAATAGCTTTTTTACAAATTATTTT
TACGAAAATTTTTGTTATATTTTTTTATAATTATTTTAATACATTTTTAA
CATTAAATAATTATATAATTTTTTAATACAATAATTACAGTTATAAATTC
ATATTATTTAAATATTTTTAAATGAGTTTATTTAGAAATTAAGTTTATAT
TTTATTTAATTAGTTTTGACTCTCAGAATGTATAGACACTTTTTAGAACA
CTGTGCACAAAGATGAAGTTTACATTCTTGGTTCAAATTACATTTAGTTC
CGTTCTTACTACACATGGCACACTTCTTGTTGATTACCGAATCATTGAAA
CATGGAGGGCAAAGTTTAGAATTAATAACAACATTATATATGTTTATGTT
TATTCCTAAACAATTATAGCAAATATTGTTTTGACATCCATCTCTTGTAC
ATGAAATATATAAATTTTTTCCTTGACACAAAGAATGACAATTAATACAC
TTTACTTCTAAGAGAGAATAATTTTCTGGATTTGAAAGGGCATCTGTTGA

AGAAACTAAAAAGAAATCAATTTTGGAAGAAGTTTTATTGACTTCTTCTT
TAATAAACTTTTTTAAATCGGCAATTTCATTGGAATTTATTTGGGTAGAT
TCGGATTGAATTTTGTAATAATTGCTATTTACTTTTTCGAAATCGAT

pPI470 – mNeon (C)

AAGCTTCCGGGGGATCGAGTTTACCACTCCCTATCAGTGATAGAGAAAAG
TGAAAGTCGAGTTTACCACTCCCTATCAGTGATAGAGAAAAGTGAAAGTC
GAGTTTACCACTCCCTATCAGTGATAGAGAAAAGTGAAAGTCGAGTTTAC
CACTCCCTATCAGTGATAGAGAAAAGTGAAAGTCGAGTTTACCACTCCCT
ATCAGTGATAGAGAAAAGTGAAAGTCGAGTTTACCACTCCCTATCAGTGA
TAGAGAAAAGTGAAAGTCGAGTTTACCACTCCCTATCAGTGATAGAGAAA
AGTGAAAGTCGAGCTCGGTACCCAATTTTTTTTTTTTTTTTTTTTTTTTT
TTTTTTTTTTTTTTTCAGATTGCATAAAAAGATTTTTTTTTTTTTTTTTC
TTATTTCTTAAAACAAATAAATTAAATTAAATAAAAAATAAAAATCAGAT
CTTCAACTAGTGGTGGTTCAGGAGGTAGTGTGAGTAAAGGTGAAGAAGAT
AATATGGCATCGTTACCAGCTACACATGAGTTACATATATTCGGTAGCAT
TAATGGTGTTGATTTTGATATGGTGGGACAAGGTACCGGTAATCCTAATG
ATGGTTACGAAGAACTAAATTTAAAATCGACTAAAGGTGACTTACAATTT
TCTCCATGGATTTTAGTGCCACATATAGGGTATGGTTTTCATCAATACTT
ACCATATCCAGATGGTATGTCACCATTTCAAGCTGCAATGGTTGATGGAT
CAGGTTATCAAGTTCATAGAACAATGCAATTTGAAGATGGTGCTTCATTA
ACTGTTAATTATAGATACACATATGAAGGCTCACATATTAAAGGTGAAGC
TCAAGTTAAAGGTACTGGTTTCCCAGCCGATGGCCCAGTTATGACAAATA
GTTTAACAGCAGCAGATTGGTGTAGATCCAAAAAAACTTATCCAAATGAT
AAAACAATTATTTCAACTTTTAAATGGTCATATACAACCGGTAATGGTAA
ACGTTATCGTTCAACAGCCCGTACAACATATACTTTTGCTAAACCAATGG
CAGCTAATTATTTAAAAAATCAACCAATGTATGTTTTTCGTAAAACAGAG
TTAAAACATTCAAAAACAGAACTTAATTTTAAAGAATGGCAAAAAGCATT
TACAGACGTTATGTAAGCTAGTTTAAATAAATAAATTATTTAATAAATAA
TAAAAAAACAAATTGTTGTAATAATCTAATATTTTCTTTTTTTTTTAATT
TTTTTTTTTTAAATCTTAATAATTATTAAGTTATTTTAATTTTTTTTTTT
TTTTTTTTTTTTTTTTTTTTTTTTTTTCTATCAAAAAAATCAAATATATT
TAAAAAATTTATTATTTACAGATACATTTTGAATGGTGAAGATAAATATA
TGCATTAGATGTAAAACAGCCAAAGAGTATGAAAATCAAAAAGATACTCG
AGATTTTATTTAATATACTAAATAATAAAAAAGTTAAAAAATGATCATTG
GATAAATTTTTTATAATTATAAATAAAGATAATAATTTTTTTTTTAACAA
AACTAAAAATAAAAATAATAAAATAATTGTTAAAATAGGTTTTTTTTTTT
TTTTTTTTTTTTTTTAATAAATGGTATTTATTAATTTATTTGTTGTGTGT
TTTTTTTTTTATAATATTTTTTTTTTTAGCATTGAATTAAGAAGAAATCA
AATTGATGCGGCCGCTTAGTTAGCCTCCCCCATCTCCCGATCCGGACGAG
TGCTGGGGCGTCGGTTTCCACTATCGGCGAGTACTTCTACACAGCCATCG
GTCCAAACGGCCGCGCTTCTGCGGGCGATTTGTGTACGCCCGACAGTCCC
GGCTCCGGATCGGACGATTGCGTCGCATCGACCCTGCGCCCAAGCTGCAT
CATCGAAATTGCCGTCAACCAAGCTCTGATAGAGTTGGTCAAGACCAATG
CGGAGCATATACGCCCGGAGCCGCGGCGATCCTGCAAGCTCCGGATGCCT
CCGCTCGAAGTAGCGCGTCTGCTGCTCCATACAAGCCAACCACGGCCTCC
AGAAGAAGATGTTGGCGACCTCGTATTGGGAATCCCCGAACATCGCCTCG
CTCCAGTCAATGACCGCTGTTATGCGGCCATTGTCCGTCAGGACATTGTT
GGAGCCGAAATCCGCGTGCACGAGGTGCCGGACTTCGGGGCAGTCCTCGG

CCCAAAGCATCAGCTCATCGAGAGCCTGCGCGACGGACGCACTGACGGTG
TCGTCCATCACAGTTTGCCAGTGATACACATGGGGATCAGCAATCGCGCA
TATGAAATCACGCCATGTAGTGTATTGACCGATTCCTTGCGGTCCGAATG
GGCCGAACCCGCTCGTCTGGCTAAGATCGGCCGCAGCGATCGCATCCATG
GCCTCCGCGACCGGCTGCAGAACAGCGGGCAGTTCGGTTTCAGGCAGGTC
TTGCAACGTGACACCCTGTGCACGGCGGGAGATGCAATAGGTCAGGCTCT
CGCTAAATTCCCCAATGTCAAGCACTTCCGGAATCGGGAGCGCGGCCGAT
GCAAAGTGCCGATAAACATAACGATCTTTGTAGAAACCATCGGCGCAGCT
ATTTACCCGCAGGACATATCCACGCCCTCCTACATCGAAGCTGAAAGCAC
GAGATTCTTCGCCCTCCGAGAGCTGCATCAGGTCGGAGACGCTGTCGAAC
TTTTCGATCAGAAACTTCTCGACAGACGTCGCGGTGAGTTCAGGCTTTTT
CATTAGGTCTTGTTGAGAAATGTTAAATTGATCCATTTTTTGCTAGCTGT
GAAATTAGTTTAAAATACAAATAAAGAGTTATAATAATATACAGTTGAAT
AAAAAAAAAAAAAATGAATTGGAAAATTTATTTTTATATGAAGAAAAAAA
AATTTTGAAAAAAAAAAAAAAAATTAAAAAAAAAAAAAAAAAAAAAAAAA
ATTAAAAATAATTCAACTGTGGGTGACCCAAATTTTATTTAAAAAAAAAA
AAAAAATGGATCACTTTTGGGTGGTTGGAAAAAAAAAAAAAAAAAAATTG
AAAATATAATGTTAGTCATATGATTAATCATTAATTAATTCTTTTTTTTT
TTTTTTTTATTTTTTATTTTTTTTTTTTCAATATTCCAATTATGACACAC
ACTAACATCACAAAATACAAAATTTTGGGATCCCGCTACAGGGCGCGTCA
GGTGGCACTTTTCGGGGAAATGTGCGCGGAACCCCTATTTGTTTATTTTT
CTAAATACATTCAAATATGTATCCGCTCATGAGACAATAACCCTGATAAA
TGCTTCAATAATATTGAAAAAGGAAGAGTATGAGTATTCAACATTTCCGT
GTCGCCCTTATTCCCTTTTTTGCGGCATTTTGCCTTCCTGTTTTTGCTCA
CCCAGAAACGCTGGTGAAAGTAAAAGATGCTGAAGATCAGTTGGGTGCAC
GAGTGGGTTACATCGAACTGGATCTCAACAGCGGTAAGATCCTTGAGAGT
TTTCGCCCCGAAGAACGTTTTCCAATGATGAGCACTTTTAAAGTTCTGCT
ATGTGGCGCGGTATTATCCCGTATTGACGCCGGGCAAGAGCAACTCGGTC
GCCGCATACACTATTCTCAGAATGACTTGGTTGAGTACTCACCAGTCACA
GAAAAGCATCTTACGGATGGCATGACAGTAAGAGAATTATGCAGTGCTGC
CATAACCATGAGTGATAACACTGCGGCCAACTTACTTCTGACAACGATCG
GAGGACCGAAGGAGCTAACCGCTTTTTTGCACAACATGGGGGATCATGTA
ACTCGCCTTGATCGTTGGGAACCGGAGCTGAATGAAGCCATACCAAACGA
CGAGCGTGACACCACGATGCCTGTAGCAATGGCAACAACGTTGCGCAAAC
TATTAACTGGCGAACTACTTACTCTAGCTTCCCGGCAACAATTAATAGAC
TGGATGGAGGCGGATAAAGTTGCAGGACCACTTCTGCGCTCGGCCCTTCC
GGCTGGCTGGTTTATTGCTGATAAATCTGGAGCCGGTGAGCGTGGGTCTC
GCGGTATCATTGCAGCACTGGGGCCAGATGGTAAGCCCTCCCGTATCGTA
GTTATCTACACGACGGGGAGTCAGGCAACTATGGATGAACGAAATAGACA
GATCGCTGAGATAGGTGCCTCACTGATTAAGCATTGGTAACTGTCAGACC
AAGTTTACTCATATATACTTTAGATTGATTTAAAACTTCATTTTTAATTT
AAAAGGATCTAGGTGAAGATCCTTTTTGATAATCTCATGACCAAAATCCC
TTAACGTGAGTTTTCGTTCCACTGAGCGTCAGACCCCGTAGAAAAGATCA
AAGGATCTTCTTGAGATCCTTTTTTTCTGCGCGTAATCTGCTGCTTGCAA
ACAAAAAAACCACCGCTACCAGCGGTGGTTTGTTTGCCGGATCAAGAGCT
ACCAACTCTTTTTCCGAAGGTAACTGGCTTCAGCAGAGCGCAGATACCAA
ATACTGTCCTTCTAGTGTAGCCGTAGTTAGGCCACCACTTCAAGAACTCT
GTAGCACCGCCTACATACCTCGCTCTGCTAATCCTGTTACCAGTGGCTGC
TGCCAGTGGCGATAAGTCGTGTCTTACCGGGTTGGACTCAAGACGATAGT
TACCGGATAAGGCGCAGCGGTCGGGCTGAACGGGGGGTTCGTGCACACAG

CCCAGCTTGGAGCGAACGACCTACACCGAACTGAGATACCTACAGCGTGA
GCTATGAGAAAGCGCCACGCTTCCCGAAGGGAGAAAGGCGGACAGGTATC
CGGTAAGCGGCAGGGTCGGAACAGGAGAGCGCACGAGGGAGCTTCCAGGG
GGAAACGCCTGGTATCTTTATAGTCCTGTCGGGTTTCGCCACCTCTGACT
TGAGCGTCGATTTTTGTGATGCTCGTCAGGGGGGCGGAGCCTATGGAAAA
ACGCCAGCAACGCGGCCTTTTTACGGTTCCTGGCCTTTTGCTGGCCTTTT
GCTCACATGTTCTTTCCTGCGTTATCCCCTGATTCTGTGGATAACCGTAT
TACCGCCTTTGAGTGAGCTGATACCGCTCGCCGCAGCCGAACGACCGAGC
GCAGCGAGTCAGTGAGCGAGGAAGCGGAAGAGCGCCCAATACGCAAACCG
CCTCTGCCGGCTAAAAAAAATTTTATTTATTTTTATTTATTTTGAATTA
AATAGATTACAAATTAATTAATCCCATCAAATCTTTAAAAAAAAAATGGTT
TAAAAAAACTTGGGTTGGTTAATTATTATTTGAAAATTTTAAAACCCAAA
TTAAAAAAAAAAAAATGGGATTCAAAAATTTTTTTTTTTTTTTTTTTTTT
TTTTTTTTTTTTTTTTTCAGATTGCATAAAAAGATTTTTTTTTTTTTTT
TCTTATTTCTTAAAACAAATAAATTAAATTAAATAAAAAATAAAAATCAG
ATCCAAAATGTCAAGATTAGATAAAAGTAAAGTGATTAACGGCGCATTAG
AGCTGCTTAATGGGGTCGGAATCGAAGGTTTAACAACCCGTAAACTCGCC
CAGAAGCTAGGTGTAGAGCAGCCTACATTGTATTGGCATGTAAAAAATAA
GCGGGCTTTGCTCGACGCCTTACCCATTGAGATGTTAGATAGGCACCATA
CTCACTTTTGCCCTTTAGAAGGGGAAAGCTGGCAAGATTTTTTACGTAAT
AACGCTAAAAGTTTTAGATGTGCTTTACTAAGTCATCGCGATGGAGCAAA
AGTACATTTAGGTACACGGCCTACAGAAAAACAGTATGAAACTCTCGAAA
ATCAATTAGCCTTTTTATGCCAACAAGGTTTTTCACTAGAGAATGCATTA
TATGCACTCAGCGCTGTGGGGCACTTTACTTTAGGTTGCGTATTGGAAGA
ACAAGAGCATCAAGTCGCTAAAGAAGAAAGGGAAACACCTACTACTGATA
GTATGCCGCCATTATTACGACAAGCTATCGAATTATTTGATCGCCAAGGT
GCAGAGCCAGCCTTCTTATTCGGCCTTGAATTGATCATATGCGGATTAGA
AAAACAACTTAAATGTGAAAGTGGATCACACACTCGTAGACTCTCAACTG
CCCCACCAACCGATGTCAGCCTGGGGGACGAGCTCCACTTAGACGGCGAG
GACGTGGCGATGGCGCATGCCGACGCGCTAGACGATTTCGATCTGGACAT
GTTGGGGGACGGGGATTCCCCGGGCCCGGGATTTACCCCCCACGACTCCG
CCCCCTACGGCGCTCTGGATATGGCCGACTTCGAGTTTGAGCAGATGTTT
ACCGATGCCCTTGGAATTGACGAGTACGGTGGTTAATCTAGTACGCGTAA
ATTGTTCACAGAAAATGTTTCTAAATTATTTAATAAATAATAAAAAAAACA
AATTGTTGTAATAATCTAATATTTTCTTTTTTTTTTAATTTTTTTTTTT
AAATCTTAATAATTATTAAGTTATTTTAATTTTTTTTTTTTTTTTTTTTT
TTTTTTTTTTTTTTTTTTTCTATCAAAAAAATCAAATATATTTAAAAAATTT
ATTATTTACAGATACATTTTGAATGGTGAAGATAAATATATGCATTAGAT
GTAAAACAGCCAAAGAGTATGAAAATCAAAAAGATAGCCGGCCTCGACAT
TTGAAATTACAATGAATGTTTTTCGGTTTTTATTTTTTACTTTTTATTTG
ATTTTTTTTTTTTTTTTTTTTTTTTTTTTTTTTTAAAATTTTTTTTTTT
TTTTTTTCAAATTTTTTTTTTTTTTTAAAAAAAAAAAAAATTCATTTTTGTAT
TTAAATTATTTTATAAATAAAAAATGATAGTAGTAGAAATTGTTTTTTGG
ACAAGAATCGTAAAGTCAAAATATTCAGTTGATGATGAAATCTTTAAATA
TTTGGTTAATAGAGCAAAAGTTGTGTATAACAATTCAGATTATAAAACAC
TATCTCAAACTGATTTAGAGAAATGGGCGATTCAAAACACTCACAGAACA
ATAACTTCAATTAAAAATCGTTTTTCAGTCAAAAAGATTGGAGATGAAGA
AAAGTTATTTAGAATTTCAAAAAATGGTAAAATCCATCTTTATACATGTT
CATTTTAATTTATTTTAACTAATTTTTTAAATTAGGGGAGCTTATTGTTC
TTAATGAATTAGAATTCGATAATTTTCATATTAAAGAAGGAAAGCATTTA

AGAAAATCAAAAATGTTTAATCATATCAAAGACAGTGGTTATTATGCAAC
AAACGAAGAAATTGAAATTTTTCTTGAAAGCTGCACTCTATGTAAGGAAA
TTACTGCACAAACCAAACGTAATTCATATAAAAAAAGAAATATAATTAAT
AAATTACCGGAAGAAGAAGAAGAAGAAGAAGAAGAAGAAGAAGAAGAAGA
AGAAGAAGAAGAACAAGAAGAAGAAGTAGAAAAACCGACAATATCGGAAG
AAGAAGAAGAAGAAACACCTGCAGTATCAGAAGAAGAAAAAGAAGAGGAA
GAACAAGAAGAAGATAAAGAAAAAGATAAAGAAAAAAAAATAGAAGAAGA
TACAGAAACAGGAAAAAAAAAAAGATGAAGTTATGAATGAACGTCAAGATG
GTATTGAAATTAGTGAAAAAACAAATGATAAACCAACAGAAAAAACAAAA
GTTAAAAAGAATGTTTCACGAGTCAAAGATATTAATCTTCATAAAAAAGG
AAATCCTATTACCCCAAAAAAAATAAAAAATTTCAGTCAACAACAATTAC
GTTTAGCTTCTGGTGTTTCAAATGAAAAAAGAGCAATAATTACTCTTAAA
AAATAATAAAACTTTATTTAATTAAAAAAATAATTAAAAAGAAAATTAAA
TATATATTAATTTTTAAATTATTTGAAATGTTTTTTTCTTGAGTTACTGA
CTTTTCCATACCGTTATTTGAGCCATCGAGCTTTGGATTGTAAATTGTTG
ATAAAAATTCAGAATCAGATGCACCATACTTTGGAGTACACTTTGTTGTT
TTAAAATCAAGATTGTTTTGAATGTTTTCATTCTTACAATAGTGGCACAA
TGGATCTGTACATGTAAATCTATTGGATTTCTCTTGTGTAATGTTTGTAA
TATGATATACATTTTCAAATTTTGAACAAATGTTGAATTTTTCAATAATT
GTTTTACTATTTTTTGTACAATCCATTGAAAGAGAAGTAATTTCAACAAA
GCTGTCTTCCACTCTAAACAAATAACCATCATCAGTTTCATTATATTCTT
TATAAAGAATAGACTCTCCAAACTCGTCATTAATACATTCATTAAGTTTA
ATTAAGTTTGTTAAATTACGAAAATTGTTACATCTTGAATTATTTGTTTC
AATGTCATCATAAAAAAGCGCATGAGTACTATTTGTTTCAACTCTTCCAT
GAATATTACATTCATCATAAATTACGGCGCCTGTCATTGACTTTTCAACA
CAGTTTTCTTTTTGATACATAACAATAAGAAGTTGAAAATATTTTACTTC
AATCTGATCATTTTGAAGAACAGAAGATTTTGATACAATAAAAAAAAAAA
GAAAAAATAATAACGTTGTTGAGTACATTATATACAATGAAAATAATAAA
TGAACAAAAAATAATTTTTTATTATAATATATTAAACCCCACCCCACAAC
CACACCTAAAAAAAAGTTAATACTTTTTTAAATATAATTTTTTTAATAGT
TATTTTAATACGTTTTTAACATAAAATATATTTTAATTTTTTTTTTAATT
TTTTAGAAAATTTTTTTTTCATAATTATTTTAATACTTTTTTACAAAAAA
TATTTTTAAAATTTTAAAAATTTTTTTTTTATAATTATTTTAATAGCTTA
ATTACAAAAACAATTTAATAATTTTTTTAATTTTTTTTTCATAATATTTT
TAATAGCTTTTTTACAAATTATTTTTACGAAAATTTTTGTTATATTTTTT
TATAATTATTTTAATACATTTTTAACATTAAATAATTATATAATTTTTTA
ATACAATAATTACAGTTATAAATTCATATTATTTAAATATTTTTAAATGA
GTTTATTTAGAAATTAAGTTTATATTTTATTTAATTAGTTTTGACTCTCA
GAATGTATAGACACTTTTTAGAACACTGTGCACAAAGATGAAGTTTACAT
TCTTGGTTCAAATTACATTTAGTTCCGTTCTTACTACACATGGCACACTT
CTTGTTGATTACCGAATCATTGAAACATGGAGGGCAAAGTTTAGAATTAA
TAACAACATTATATATGTTTATGTTTATTCCTAAACAATTATAGCAAATA
TTGTTTTGACATCCATCTCTTGTACATGAAATATATAAATTTTTTCCTTG
ACACAAAGAATGACAATTAATACACTTTACTTCTAAGAGAGAATAATTTT
CTGGATTTGAAAGGGCATCTGTTGAAGAAACTAAAAAGAAATCAATTTTG
GAAGAAGTTTTATTGACTTCTTCTTTAATAAACTTTTTTAAATCGGCAAT
TTCATTGGAATTTATTTGGGTAGATTCGGATTGAATTTTGTAATAATTGC
TATTTACTTTTTCGAAATCGAT

pPI452 – mScarlet (N)

AAGCTTCCGGGGGATCGAGTTTACCACTCCCTATCAGTGATAGAGAAAAG
TGAAAGTCGAGTTTACCACTCCCTATCAGTGATAGAGAAAAGTGAAAGTC
GAGTTTACCACTCCCTATCAGTGATAGAGAAAAGTGAAAGTCGAGTTTAC
CACTCCCTATCAGTGATAGAGAAAAGTGAAAGTCGAGTTTACCACTCCCT
ATCAGTGATAGAGAAAAGTGAAAGTCGAGTTTACCACTCCCTATCAGTGA
TAGAGAAAAGTGAAAGTCGAGTTTACCACTCCCTATCAGTGATAGAGAAA
AGTGAAAGTCGAGCTCGGTACCCAATTTTTTTTTTTTTTTTTTTTTTTTT
TTTTTTTTTTTTTTCAGATTGCATAAAAAGATTTTTTTTTTTTTTTTTTC
TTATTTCTTAAAACAAATAAATTAAATTAAATAAAAAATAAAAATCAGAT
CCAAAAAATGGTTTCAAAAGGTGAAGCCGTTATTAAAGAATTTATGAGAT
TCAAGGTTCACATGGAAGGAAGTATGAACGGTCATGAATTTGAGATTGAA
GGAGAAGGTGAAGGTAGACCATATGAAGGCACCCAAACAGCTAAATTAAA
AGTAACTAAAGGTGGTCCATTACCATTTAGTTGGGATATTTTATCTCCAC
AATTTATGTATGGTTCACGTGCTTTCACAAAACATCCAGCAGATATTCCA
GATTATTATAAACAATCATTTCCAGAAGGTTTTAAATGGGAACGTGTCAT
GAACTTTGAAGATGGTGGAGCAGTTACAGTCACACAAGATACCTCATTAG
AAGATGGTACATTAATATATAAAGTTAAATTACGTGGTACTAATTTTCCA
CCAGACGGTCCAGTAATGCAAAAAAAAACAATGGGCTGGGAAGCTAGTAC
AGAACGTTTATATCCTGAAGATGGTGTCCTTAAAGGCGATATAAAAATGG
CCTTGAGATTAAAGGATGGTGGTAGGTATTTAGCAGATTTCAAAACCACT
TATAAAGCAAAAAAACCAGTTCAAATGCCAGGTGCATATAATGTTGATAG
AAAACTTGATATTACCAGTCATAATGAAGATTACACAGTTGTCGAACAAT
ACGAACGTTCTGAAGGTCGTCATAGCACTGGTGGTATGGATGAATTATAC
AAAGGTGGTTCAGGAGGTAGTAGATCTTCAACTAGTTAAATAAATAAATT
ATTTAATAAATAATAAAAAAACAAATTGTTGTAATAATCTAATATTTTCT
TTTTTTTTTAATTTTTTTTTTTTAAATCTTAATAATTATTAAGTTATTTT
AATTTTTTTTTTTTTTTTTTTTTTTTTTTTTTTTTTTTTTCTATCAAAAA
AATCAAATATATTTAAAAAATTTATTATTTACAGATACATTTTGAATGGT
GAAGATAAATATATGCATTAGATGTAAAACAGCCAAAGAGTATGAAAATC
AAAAAGATACTCGAGATTTTATTTAATATACTAAATAATAAAAAAGTTAA
AAAATGATCATTGGATAAATTTTTTATAATTATAAATAAAGATAATAATT
TTTTTTTTAACAAAACTAAAAATAAAAATAATAAAATAATTGTTAAAATA
GGTTTTTTTTTTTTTTTTTTTTTTTTAATAAATGGTATTTATTAATTTAT
TTGTTGTGTGTGTTTTTTTTTTTATAATATTTTTTTTTTTAGCATTGAAT
TAAGAAGAAATCAAATTGATGCGGCCGCTTAGTTAGCCTCCCCCATCTCC
CGATCCGGACGAGTGCTGGGGCGTCGGTTTCCACTATCGGCGAGTACTTC
TACACAGCCATCGGTCCAAACGGCCGCGCTTCTGCGGGCGATTTGTGTAC
GCCCGACAGTCCCGGCTCCGGATCGGACGATTGCGTCGCATCGACCCTGC
GCCCAAGCTGCATCATCGAAATTGCCGTCAACCAAGCTCTGATAGAGTTG
GTCAAGACCAATGCGGAGCATATACGCCCGGAGCCGCGGCGATCCTGCAA
GCTCCGGATGCCTCCGCTCGAAGTAGCGCGTCTGCTGCTCCATACAAGCC
AACCACGGCCTCCAGAAGAAGATGTTGGCGACCTCGTATTGGGAATCCCC
GAACATCGCCTCGCTCCAGTCAATGACCGCTGTTATGCGGCCATTGTCCG
TCAGGACATTGTTGGAGCCGAAATCCGCGTGCACGAGGTGCCGGACTTCG
GGGCAGTCCTCGGCCCAAAGCATCAGCTCATCGAGAGCCTGCGCGACGGA
CGCACTGACGGTGTCGTCCATCACAGTTTGCCAGTGATACACATGGGGAT
CAGCAATCGCGCATATGAAATCACGCCATGTAGTGTATTGACCGATTCCT
TGCGGTCCGAATGGGCCGAACCCGCTCGTCTGGCTAAGATCGGCCGCAGC
GATCGCATCCATGGCCTCCGCGACCGGCTGCAGAACAGCGGGCAGTTCGG

TTTCAGGCAGGTCTTGCAACGTGACACCCTGTGCACGGCGGGAGATGCAA
TAGGTCAGGCTCTCGCTAAATTCCCCAATGTCAAGCACTTCCGGAATCGG
GAGCGCGGCCGATGCAAAGTGCCGATAAACATAACGATCTTTGTAGAAAC
CATCGGCGCAGCTATTTACCCGCAGGACATATCCACGCCCTCCTACATCG
AAGCTGAAAGCACGAGATTCTTCGCCCTCCGAGAGCTGCATCAGGTCGGA
GACGCTGTCGAACTTTTCGATCAGAAACTTCTCGACAGACGTCGCGGTGA
GTTCAGGCTTTTTCATTAGGTCTTGTTGAGAAATGTTAAATTGATCCATT
TTTTGCTAGCTGTGAAATTAGTTTAAAATACAAATAAAGAGTTATAATAA
TATACAGTTGAATAAAAAAAAAAAAAATGAATTGGAAAATTTATTTTTAT
ATGAAGAAAAAAAAATTTTGAAAAAAAAAAAAAAAATTAAAAAAAAAAAA
AAAAAAAAAAAAAATTAAAAATAATTCAACTGTGGGTGACCCAAATTTTA
TTTAAAAAAAAAAAAAAAATGGATCACTTTTGGGTGGTTGGAAAAAAAAA
AAAAAAAAAATTGAAAATATAATGTTAGTCATATGATTAATCATTAATTA
ATTCTTTTTTTTTTTTTTTTATTTTTTTTTATTTTTTTTTTCAATATTCC
AATTATGACACACACTAACATCACAAAATACAAAATTTTGGGATCCCGCT
ACAGGGCGCGTCAGGTGGCACTTTTCGGGGAAATGTGCGCGGAACCCCTA
TTTGTTTATTTTTCTAAATACATTCAAATATGTATCCGCTCATGAGACAA
TAACCCTGATAAATGCTTCAATAATATTGAAAAAGGAAGAGTATGAGTAT
TCAACATTTCCGTGTCGCCCTTATTCCCTTTTTTGCGGCATTTTGCCTTC
CTGTTTTTGCTCACCCAGAAACGCTGGTGAAAGTAAAAGATGCTGAAGAT
CAGTTGGGTGCACGAGTGGGTTACATCGAACTGGATCTCAACAGCGGTAA
GATCCTTGAGAGTTTTCGCCCCGAAGAACGTTTTCCAATGATGAGCACTT
TTAAAGTTCTGCTATGTGGCGCGGTATTATCCCGTATTGACGCCGGGCAA
GAGCAACTCGGTCGCCGCATACACTATTCTCAGAATGACTTGGTTGAGTA
CTCACCAGTCACAGAAAAGCATCTTACGGATGGCATGACAGTAAGAGAAT
TATGCAGTGCTGCCATAACCATGAGTGATAACACTGCGGCCAACTTACTT
CTGACAACGATCGGAGGACCGAAGGAGCTAACCGCTTTTTTGCACAACAT
GGGGGATCATGTAACTCGCCTTGATCGTTGGGAACCGGAGCTGAATGAAG
CCATACCAAACGACGAGCGTGACACCACGATGCCTGTAGCAATGGCAACA
ACGTTGCGCAAACTATTAACTGGCGAACTACTTACTCTAGCTTCCCGGCA
ACAATTAATAGACTGGATGGAGGCGGATAAAGTTGCAGGACCACTTCTGC
GCTCGGCCCTTCCGGCTGGCTGGTTTATTGCTGATAAATCTGGAGCCGGT
GAGCGTGGGTCTCGCGGTATCATTGCAGCACTGGGGCCAGATGGTAAGCC
CTCCCGTATCGTAGTTATCTACACGACGGGGAGTCAGGCAACTATGGATG
AACGAAATAGACAGATCGCTGAGATAGGTGCCTCACTGATTAAGCATTGG
TAACTGTCAGACCAAGTTTACTCATATATACTTTAGATTGATTTAAAACT
TCATTTTTAATTTAAAAGGATCTAGGTGAAGATCCTTTTTGATAATCTCA
TGACCAAAATCCCTTAACGTGAGTTTTCGTTCCACTGAGCGTCAGACCCC
GTAGAAAAGATCAAAGGATCTTCTTGAGATCCTTTTTTTCTGCGCGTAAT
CTGCTGCTTGCAAACAAAAAAACCACCGCTACCAGCGGTGGTTTGTTTGC
CGGATCAAGAGCTACCAACTCTTTTTCCGAAGGTAACTGGCTTCAGCAGA
GCGCAGATACCAAATACTGTCCTTCTAGTGTAGCCGTAGTTAGGCCACCA
CTTCAAGAACTCTGTAGCACCGCCTACATACCTCGCTCTGCTAATCCTGT
TACCAGTGGCTGCTGCCAGTGGCGATAAGTCGTGTCTTACCGGGTTGGAC
TCAAGACGATAGTTACCGGATAAGGCGCAGCGGTCGGGCTGAACGGGGGG
TTCGTGCACACAGCCCAGCTTGGAGCGAACGACCTACACCGAACTGAGAT
ACCTACAGCGTGAGCTATGAGAAAGCGCCACGCTTCCCGAAGGGAGAAAG
GCGGACAGGTATCCGGTAAGCGGCAGGGTCGGAACAGGAGAGCGCACGAG
GGAGCTTCCAGGGGGAAACGCCTGGTATCTTTATAGTCCTGTCGGGTTTC
GCCACCTCTGACTTGAGCGTCGATTTTTGTGATGCTCGTCAGGGGGGCGG

AGCCTATGGAAAAACGCCAGCAACGCGGCCTTTTTACGGTTCCTGGCCTT
TTGCTGGCCTTTTGCTCACATGTTCTTTCCTGCGTTATCCCCTGATTCTG
TGGATAACCGTATTACCGCCTTTGAGTGAGCTGATACCGCTCGCCGCAGC
CGAACGACCGAGCGCAGCGAGTCAGTGAGCGAGGAAGCGGAAGAGCGCCC
AATACGCAAACCGCCTCTGCCGGCTAAAAAAAATTTTATTTATTTTTAT
TTATTTTGAATTAAATAGATTACAAATTAATTAATCCCATCAAATCTTTA
AAAAAAAATGGTTTAAAAAAACTTGGGTTGGTTAATTATTATTTGAAAAT
TTTAAAACCCAAATTAAAAAAAAAAAAATGGGATTCAAAAATTTTTTTTT
TTTTTTTTTTTTTTTTTTTTTTTTTTTTTCAGATTGCATAAAAAGATTTT
TTTTTTTTTTTTTTCTTATTTCTTAAAACAAATAAATTAAATTAAATAAA
AAATAAAAATCAGATCCAAAATGTCAAGATTAGATAAAAGTAAAGTGATT
AACGGCGCATTAGAGCTGCTTAATGGGGTCGGAATCGAAGGTTTAACAAC
CCGTAAACTCGCCCAGAAGCTAGGTGTAGAGCAGCCTACATTGTATTGGC
ATGTAAAAAATAAGCGGGCTTTGCTCGACGCCTTACCCATTGAGATGTTA
GATAGGCACCATACTCACTTTTGCCCTTTAGAAGGGGAAAGCTGGCAAGA
TTTTTTACGTAATAACGCTAAAAGTTTTAGATGTGCTTTACTAAGTCATC
GCGATGGAGCAAAAGTACATTTAGGTACACGGCCTACAGAAAAACAGTAT
GAAACTCTCGAAAATCAATTAGCCTTTTTATGCCAACAAGGTTTTTCACT
AGAGAATGCATTATATGCACTCAGCGCTGTGGGGCACTTTACTTTAGGTT
GCGTATTGGAAGAACAAGAGCATCAAGTCGCTAAAGAAGAAAGGGAAACA
CCTACTACTGATAGTATGCCGCCATTATTACGACAAGCTATCGAATTATT
TGATCGCCAAGGTGCAGAGCCAGCCTTCTTATTCGGCCTTGAATTGATCA
TATGCGGATTAGAAAAACAACTTAAATGTGAAAGTGGATCACACACTCGT
AGACTCTCAACTGCCCCACCAACCGATGTCAGCCTGGGGGACGAGCTCCA
CTTAGACGGCGAGGACGTGGCGATGGCGCATGCCGACGCGCTAGACGATT
TCGATCTGGACATGTTGGGGGACGGGGATTCCCCGGGCCCGGGATTTACC
CCCCACGACTCCGCCCCCTACGGCGCTCTGGATATGGCCGACTTCGAGTT
TGAGCAGATGTTTACCGATGCCCTTGGAATTGACGAGTACGGTGGTTAAT
CTAGTACGCGTAAATTGTTCACAGAAAATGTTTCTAAATTATTTAATAAA
TAATAAAAAAAACAAATTGTTGTAATAATCTAATATTTTCTTTTTTTTTA
ATTTTTTTTTTTTAAATCTTAATAATTATTAAGTTATTTTAATTTTTTTT
TTTTTTTTTTTTTTTTTTTTTTTTTTTTTTCTATCAAAAAAATCAAATAT
ATTTAAAAAATTTATTATTTACAGATACATTTTGAATGGTGAAGATAAAT
ATATGCATTAGATGTAAAACAGCCAAAGAGTATGAAAATCAAAAAGATAG
CCGGCCTCGACATTTGAAATTACAATGAATGTTTTTCGGTTTTTATTTTT
TACTTTTTATTTGATTTTTTTTTTTTTTTTTTTTTTTTTTTTTTTTTTAA
AATTTTTTTTTTTTTTTTTTTCAAATTTTTTTTTTTTTTAAAAAAAAAAAAA
TTCATTTTTGTATTTAAATTATTTTATAAATAAAAAATGATAGTAGTAGA
AATTGTTTTTTGGACAAGAATCGTAAAGTCAAAATATTCAGTTGATGATG
AAATCTTTAAATATTTGGTTAATAGAGCAAAAGTTGTGTATAACAATTCA
GATTATAAAACACTATCTCAAACTGATTTAGAGAAATGGGCGATTCAAAA
CACTCACAGAACAATAACTTCAATTAAAAATCGTTTTTCAGTCAAAAAGA
TTGGAGATGAAGAAAAGTTATTTAGAATTTCAAAAAATGGTAAAATCCAT
CTTTATACATGTTCATTTTAATTTATTTTAACTAATTTTTTAAATTAGGG
GAGCTTATTGTTCTTAATGAATTAGAATTCGATAATTTTCATATTAAAGA
AGGAAAGCATTTAAGAAAATCAAAAATGTTTAATCATATCAAAGACAGTG
GTTATTATGCAACAAACGAAGAAATTGAAATTTTTCTTGAAAGCTGCACT
CTATGTAAGGAAATTACTGCACAAACCAAACGTAATTCATATAAAAAAAG
AAATATAATTAATAAATTACCGGAAGAAGAAGAAGAAGAAGAAGAAGAAG
AAGAAGAAGAAGAAGAAGAAGAAGAACAAGAAGAAGAAGTAGAAAAACCG

ACAATATCGGAAGAAGAAGAAGAAGAAACACCTGCAGTATCAGAAGAAGA
AAAAGAAGAGGAAGAACAAGAAGAAGATAAAGAAAAAGATAAAGAAAAAA
AAATAGAAGAAGATACAGAAACAGGAAAAAAAAAAAGATGAAGTTATGAAT
GAACGTCAAGATGGTATTGAAATTAGTGAAAAAACAAATGATAAACCAAC
AGAAAAAACAAAAGTTAAAAAGAATGTTTCACGAGTCAAAGATATTAATC
TTCATAAAAAAGGAAATCCTATTACCCCAAAAAAAATAAAAAATTTCAGT
CAACAACAATTACGTTTAGCTTCTGGTGTTTCAAATGAAAAAAGAGCAAT
AATTACTCTTAAAAAATAATAAAACTTTATTTAATTAAAAAAATAATTAA
AAAGAAAATTAAATATATATTAATTTTTAAATTATTTGAAATGTTTTTTT
CTTGAGTTACTGACTTTTCCATACCGTTATTTGAGCCATCGAGCTTTGGA
TTGTAAATTGTTGATAAAAATTCAGAATCAGATGCACCATACTTTGGAGT
ACACTTTGTTGTTTTAAAATCAAGATTGTTTTGAATGTTTTCATTCTTAC
AATAGTGGCACAATGGATCTGTACATGTAAATCTATTGGATTTCTCTTGT
GTAATGTTTGTAATATGATATACATTTTCAAATTTTGAACAAATGTTGAA
TTTTTCAATAATTGTTTTACTATTTTTTGTACAATCCATTGAAAGAGAAG
TAATTTCAACAAAGCTGTCTTCCACTCTAAACAAATAACCATCATCAGTT
TCATTATATTCTTTATAAAGAATAGACTCTCCAAACTCGTCATTAATACA
TTCATTAAGTTTAATTAAGTTTGTTAAATTACGAAAATTGTTACATCTTG
AATTATTTGTTTCAATGTCATCATAAAAAAGCGCATGAGTACTATTTGTT
TCAACTCTTCCATGAATATTACATTCATCATAAATTACGGCGCCTGTCAT
TGACTTTTCAACACAGTTTTCTTTTTGATACATAACAATAAGAAGTTGAA
AATATTTTACTTCAATCTGATCATTTTGAAGAACAGAAGATTTTGATACA
ATAAAAAAAAAAAGAAAAAATAATAACGTTGTTGAGTACATTATATACAA
TGAAAATAATAAATGAACAAAAAATAATTTTTTATTATAATATATTAAAC
CCCACCCCACAACCACACCTAAAAAAAAGTTAATACTTTTTTAAATATAA
TTTTTTTAATAGTTATTTTAATACGTTTTTAACATAAAATATATTTTAAT
TTTTTTTTTAATTTTTTAGAAAATTTTTTTTTCATAATTATTTTAATACT
TTTTTACAAAAAATATTTTTAAAATTTTAAAAATTTTTTTTTTATAATTA
TTTTAATAGCTTAATTACAAAAACAATTTAATAATTTTTTTAATTTTTTT
TTCATAATATTTTTAATAGCTTTTTTACAAATTATTTTTACGAAAATTTT
TGTTATATTTTTTTATAATTATTTTAATACATTTTTAACATTAAATAATT
ATATAATTTTTTAATACAATAATTACAGTTATAAATTCATATTATTTAAA
TATTTTTAAATGAGTTTATTTAGAAATTAAGTTTATATTTTATTTAATTA
GTTTTGACTCTCAGAATGTATAGACACTTTTTAGAACACTGTGCACAAAG
ATGAAGTTTACATTCTTGGTTCAAATTACATTTAGTTCCGTTCTTACTAC
ACATGGCACACTTCTTGTTGATTACCGAATCATTGAAACATGGAGGGCAA
AGTTTAGAATTAATAACAACATTATATATGTTTATGTTTATTCCTAAACA
ATTATAGCAAATATTGTTTTGACATCCATCTCTTGTACATGAAATATATA
AATTTTTTCCTTGACACAAAGAATGACAATTAATACACTTTACTTCTAAG
AGAGAATAATTTTCTGGATTTGAAAGGGCATCTGTTGAAGAAACTAAAAA
GAAATCAATTTTGGAAGAAGTTTTATTGACTTCTTCTTTAATAAACTTTT
TTAAATCGGCAATTTCATTGGAATTTATTTGGGTAGATTCGGATTGAATT
TTGTAATAATTGCTATTTACTTTTTCGAAATCGAT

pPI460 – mScarlet (C)

AAGCTTCCGGGGGATCGAGTTTACCACTCCCTATCAGTGATAGAGAAAAG
TGAAAGTCGAGTTTACCACTCCCTATCAGTGATAGAGAAAAGTGAAAGTC
GAGTTTACCACTCCCTATCAGTGATAGAGAAAAGTGAAAGTCGAGTTTAC
CACTCCCTATCAGTGATAGAGAAAAGTGAAAGTCGAGTTTACCACTCCCT

ATCAGTGATAGAGAAAAGTGAAAGTCGAGTTTACCACTCCCTATCAGTGA
TAGAGAAAAGTGAAAGTCGAGTTTACCACTCCCTATCAGTGATAGAGAAA
AGTGAAAGTCGAGCTCGGTACCCAATTTTTTTTTTTTTTTTTTTTTTTTT
TTTTTTTTTTTTTTCAGATTGCATAAAAAGATTTTTTTTTTTTTTTTTTC
TTATTTCTTAAAACAAATAAATTAAATTAAATAAAAAATAAAAATCAGAT
CTTCAACTAGTGGTGGTTCAGGAGGTAGTGTTTCAAAAGGTGAAGCCGTT
ATTAAAGAATTTATGAGATTCAAGGTTCACATGGAAGGAAGTATGAACGG
TCATGAATTTGAGATTGAAGGAGAAGGTGAAGGTAGACCATATGAAGGCA
CCCAAACAGCTAAATTAAAAGTAACTAAAGGTGGTCCATTACCATTTAGT
TGGGATATTTATCTCCACAATTTATGTATGGTTCACGTGCTTTCACAAA
ACATCCAGCAGATATTCCAGATTATTATAAACAATCATTTCCAGAAGGTT
TTAAATGGGAACGTGTCATGAACTTTGAAGATGGTGGAGCAGTTACAGTC
ACACAAGATACCTCATTAGAAGATGGTACATTAATATATAAAGTTAAATT
ACGTGGTACTAATTTTCCACCAGACGGTCCAGTAATGCAAAAAAAAACAA
TGGGCTGGGAAGCTAGTACAGAACGTTTATATCCTGAAGATGGTGTCCTT
AAAGGCGATATAAAAATGGCCTTGAGATTAAAGGATGGTGGTAGGTATTT
AGCAGATTTCAAAACCACTTATAAAGCAAAAAAACCAGTTCAAATGCCAG
GTGCATATAATGTTGATAGAAAACTTGATATTACCAGTCATAATGAAGAT
TACACAGTTGTCGAACAATACGAACGTTCTGAAGGTCGTCATAGCACTGG
TGGTATGGATGAATTATACAAATAAGCTAGTTAAATAAATAAATTATTTA
ATAAATAATAAAAAAACAAATTGTTGTAATAATCTAATATTTTCTTTTTT
TTTTAATTTTTTTTTTTTAAATCTTAATAATTATTAAGTTATTTTAATTT
TTTTTTTTTTTTTTTTTTTTTTTTTTTTTTTTTTTCTATCAAAAAAATCA
AATATATTTAAAAAATTTATTATTTACAGATACATTTTGAATGGTGAAGA
TAAATATATGCATTAGATGTAAAACAGCCAAAGAGTATGAAAATCAAAAA
GATACTCGAGATTTTATTTAATATACTAAATAATAAAAAAGTTAAAAAAT
GATCATTGGATAAATTTTTTATAATTATAAATAAAGATAATAATTTTTTT
TTTAACAAAACTAAAAATAAAAATAATAAAATAATTGTTAAAATAGGTTT
TTTTTTTTTTTTTTTTTTTTTAATAAATGGTATTTATTAATTTATTTGTT
GTGTGTGTTTTTTTTTTTATAATATTTTTTTTTTTAGCATTGAATTAAGA
AGAAATCAAATTGATGCGGCCGCTTAGTTAGCCTCCCCCATCTCCCGATC
CGGACGAGTGCTGGGGCGTCGGTTTCCACTATCGGCGAGTACTTCTACAC
AGCCATCGGTCCAAACGGCCGCGCTTCTGCGGGCGATTTGTGTACGCCCG
ACAGTCCCGGCTCCGGATCGGACGATTGCGTCGCATCGACCCTGCGCCCA
AGCTGCATCATCGAAATTGCCGTCAACCAAGCTCTGATAGAGTTGGTCAA
GACCAATGCGGAGCATATACGCCCGGAGCCGCGGCGATCCTGCAAGCTCC
GGATGCCTCCGCTCGAAGTAGCGCGTCTGCTGCTCCATACAAGCCAACCA
CGGCCTCCAGAAGAAGATGTTGGCGACCTCGTATTGGGAATCCCCGAACA
TCGCCTCGCTCCAGTCAATGACCGCTGTTATGCGGCCATTGTCCGTCAGG
ACATTGTTGGAGCCGAAATCCGCGTGCACGAGGTGCCGGACTTCGGGGCA
GTCCTCGGCCCAAAGCATCAGCTCATCGAGAGCCTGCGCGACGGACGCAC
TGACGGTGTCGTCCATCACAGTTTGCCAGTGATACACATGGGGATCAGCA
ATCGCGCATATGAAATCACGCCATGTAGTGTATTGACCGATTCCTTGCGG
TCCGAATGGGCCGAACCCGCTCGTCTGGCTAAGATCGGCCGCAGCGATCG
CATCCATGGCCTCCGCGACCGGCTGCAGAACAGCGGGCAGTTCGGTTTCA
GGCAGGTCTTGCAACGTGACACCCTGTGCACGGCGGGAGATGCAATAGGT
CAGGCTCTCGCTAAATTCCCCAATGTCAAGCACTTCCGGAATCGGGAGCG
CGGCCGATGCAAAGTGCCGATAAACATAACGATCTTTGTAGAAACCATCG
GCGCAGCTATTTACCCGCAGGACATATCCACGCCCTCCTACATCGAAGCT
GAAAGCACGAGATTCTTCGCCCTCCGAGAGCTGCATCAGGTCGGAGACGC

TGTCGAACTTTTCGATCAGAAACTTCTCGACAGACGTCGCGGTGAGTTCA
GGCTTTTTCATTAGGTCTTGTTGAGAAATGTTAAATTGATCCATTTTTTG
CTAGCTGTGAAATTAGTTTAAAATACAAATAAAGAGTTATAATAATATAC
AGTTGAATAAAAAAAAAAAAAATGAATTGGAAAATTTATTTTTATATGAA
GAAAAAAAAATTTTGAAAAAAAAAAAAAAAATTAAAAAAAAAAAAAAAAA
AAAAAAAAATTAAAAATAATTCAACTGTGGGTGACCCAAATTTTATTTAA
AAAAAAAAAAAAAATGGATCACTTTTGGGTGGTTGGAAAAAAAAAAAAAA
AAAAATTGAAAATATAATGTTAGTCATATGATTAATCATTAATTAATTCT
TTTTTTTTTTTTTTTTATTTTTTATTTTTTTTTTTTCAATATTCCAATTA
TGACACACACTAACATCACAAAATACAAAATTTTGGGATCCCGCTACAGG
GCGCGTCAGGTGGCACTTTTCGGGGAAATGTGCGCGGAACCCCTATTTGT
TTATTTTTCTAAATACATTCAAATATGTATCCGCTCATGAGACAATAACC
CTGATAAATGCTTCAATAATATTGAAAAAGGAAGAGTATGAGTATTCAAC
ATTTCCGTGTCGCCCTTATTCCCTTTTTTGCGGCATTTTGCCTTCCTGTT
TTTGCTCACCCAGAAACGCTGGTGAAAGTAAAAGATGCTGAAGATCAGTT
GGGTGCACGAGTGGGTTACATCGAACTGGATCTCAACAGCGGTAAGATCC
TTGAGAGTTTTCGCCCCGAAGAACGTTTTCCAATGATGAGCACTTTTAAA
GTTCTGCTATGTGGCGCGGTATTATCCCGTATTGACGCCGGGCAAGAGCA
ACTCGGTCGCCGCATACACTATTCTCAGAATGACTTGGTTGAGTACTCAC
CAGTCACAGAAAAGCATCTTACGGATGGCATGACAGTAAGAGAATTATGC
AGTGCTGCCATAACCATGAGTGATAACACTGCGGCCAACTTACTTCTGAC
AACGATCGGAGGACCGAAGGAGCTAACCGCTTTTTTGCACAACATGGGGG
ATCATGTAACTCGCCTTGATCGTTGGGAACCGGAGCTGAATGAAGCCATA
CCAAACGACGAGCGTGACACCACGATGCCTGTAGCAATGGCAACAACGTT
GCGCAAACTATTAACTGGCGAACTACTTACTCTAGCTTCCCGGCAACAAT
TAATAGACTGGATGGAGGCGGATAAAGTTGCAGGACCACTTCTGCGCTCG
GCCCTTCCGGCTGGCTGGTTTATTGCTGATAAATCTGGAGCCGGTGAGCG
TGGGTCTCGCGGTATCATTGCAGCACTGGGGCCAGATGGTAAGCCCTCCC
GTATCGTAGTTATCTACACGACGGGGAGTCAGGCAACTATGGATGAACGA
AATAGACAGATCGCTGAGATAGGTGCCTCACTGATTAAGCATTGGTAACT
GTCAGACCAAGTTTACTCATATATACTTTAGATTGATTTAAAACTTCATT
TTTAATTTAAAAGGATCTAGGTGAAGATCCTTTTTGATAATCTCATGACC
AAAATCCCTTAACGTGAGTTTTCGTTCCACTGAGCGTCAGACCCCGTAGA
AAAGATCAAAGGATCTTCTTGAGATCCTTTTTTTCTGCGCGTAATCTGCT
GCTTGCAAACAAAAAAACCACCGCTACCAGCGGTGGTTTGTTTGCCGGAT
CAAGAGCTACCAACTCTTTTTCCGAAGGTAACTGGCTTCAGCAGAGCGCA
GATACCAAATACTGTCCTTCTAGTGTAGCCGTAGTTAGGCCACCACTTCA
AGAACTCTGTAGCACCGCCTACATACCTCGCTCTGCTAATCCTGTTACCA
GTGGCTGCTGCCAGTGGCGATAAGTCGTGTCTTACCGGGTTGGACTCAAG
ACGATAGTTACCGGATAAGGCGCAGCGGTCGGGCTGAACGGGGGGTTCGT
GCACACAGCCCAGCTTGGAGCGAACGACCTACACCGAACTGAGATACCTA
CAGCGTGAGCTATGAGAAAGCGCCACGCTTCCCGAAGGGAGAAAGGCGGA
CAGGTATCCGGTAAGCGGCAGGGTCGGAACAGGAGAGCGCACGAGGGAGC
TTCCAGGGGGAAACGCCTGGTATCTTTATAGTCCTGTCGGGTTTCGCCAC
CTCTGACTTGAGCGTCGATTTTTGTGATGCTCGTCAGGGGGGCGGAGCCT
ATGGAAAAACGCCAGCAACGCGGCCTTTTTACGGTTCCTGGCCTTTTGCT
GGCCTTTTGCTCACATGTTCTTTCCTGCGTTATCCCCTGATTCTGTGGAT
AACCGTATTACCGCCTTTGAGTGAGCTGATACCGCTCGCCGCAGCCGAAC
GACCGAGCGCAGCGAGTCAGTGAGCGAGGAAGCGGAAGAGCGCCCAATAC
GCAAACCGCCTCTGCCGGCTAAAAAAAATTTTATTTATTTTTATTTATT

TTGAATTAAATAGATTACAAATTAATTAATCCCATCAAATCTTTAAAAAA
AAATGGTTTAAAAAAACTTGGGTTGGTTAATTATTATTTGAAAATTTTAA
AACCCAAATTAAAAAAAAAAAAATGGGATTCAAAAATTTTTTTTTTTTT
TTTTTTTTTTTTTTTTTTTTTTTCAGATTGCATAAAAAGATTTTTTTTT
TTTTTTTTTCTTATTTCTTAAAACAAATAAATTAAATTAAATAAAAAATA
AAAATCAGATCCAAAATGTCAAGATTAGATAAAAGTAAAGTGATTAACGG
CGCATTAGAGCTGCTTAATGGGGTCGGAATCGAAGGTTTAACAACCCGTA
AACTCGCCCAGAAGCTAGGTGTAGAGCAGCCTACATTGTATTGGCATGTA
AAAAATAAGCGGGCTTTGCTCGACGCCTTACCCATTGAGATGTTAGATAG
GCACCATACTCACTTTTGCCCTTTAGAAGGGGAAAGCTGGCAAGATTTTT
TACGTAATAACGCTAAAAGTTTTAGATGTGCTTTACTAAGTCATCGCGAT
GGAGCAAAAGTACATTTAGGTACACGGCCTACAGAAAAACAGTATGAAAC
TCTCGAAAATCAATTAGCCTTTTTATGCCAACAAGGTTTTTCACTAGAGA
ATGCATTATATGCACTCAGCGCTGTGGGGCACTTTACTTTAGGTTGCGTA
TTGGAAGAACAAGAGCATCAAGTCGCTAAAGAAGAAAGGGAAACACCTAC
TACTGATAGTATGCCGCCATTATTACGACAAGCTATCGAATTATTTGATC
GCCAAGGTGCAGAGCCAGCCTTCTTATTCGGCCTTGAATTGATCATATGC
GGATTAGAAAAACAACTTAAATGTGAAAGTGGATCACACACTCGTAGACT
CTCAACTGCCCCACCAACCGATGTCAGCCTGGGGGACGAGCTCCACTTAG
ACGGCGAGGACGTGGCGATGGCGCATGCCGACGCGCTAGACGATTTCGAT
CTGGACATGTTGGGGGACGGGGATTCCCCGGGCCCGGGATTTACCCCCCA
CGACTCCGCCCCCTACGGCGCTCTGGATATGGCCGACTTCGAGTTTGAGC
AGATGTTTACCGATGCCCTTGGAATTGACGAGTACGGTGGTTAATCTAGT
ACGCGTAAATTGTTCACAGAAAATGTTTCTAAATTATTTAATAAATAATA
AAAAAACAAATTGTTGTAATAATCTAATATTTTCTTTTTTTTTTTAATTTT
TTTTTTTTAAATCTTAATAATTATTAAGTTATTTTAATTTTTTTTTTTTT
TTTTTTTTTTTTTTTTTTTTTTTTTCTATCAAAAAAATCAAATATATTTA
AAAAATTTATTATTTACAGATACATTTTGAATGGTGAAGATAAATATATG
CATTAGATGTAAAACAGCCAAAGAGTATGAAAATCAAAAAGATAGCCGGC
CTCGACATTTGAAATTACAATGAATGTTTTTCGGTTTTTATTTTTTACTT
TTTATTTGATTTTTTTTTTTTTTTTTTTTTTTTTTTTTTTTTTTAAAATTT
TTTTTTTTTTTTTTTCAAATTTTTTTTTTTTTTAAAAAAAAAAAAAATTCAT
TTTTGTATTTAAATTATTTTATAAATAAAAAATGATAGTAGTAGAAATTG
TTTTTTGGACAAGAATCGTAAAGTCAAAATATTCAGTTGATGATGAAATC
TTTAAATATTTGGTTAATAGAGCAAAAGTTGTGTATAACAATTCAGATTA
TAAAACACTATCTCAAACTGATTTAGAGAAATGGGCGATTCAAAACACTC
ACAGAACAATAACTTCAATTAAAAATCGTTTTTCAGTCAAAAAGATTGGA
GATGAAGAAAAGTTATTTAGAATTTCAAAAAATGGTAAAATCCATCTTTA
TACATGTTCATTTTAATTTATTTTAACTAATTTTTTAAATTAGGGGAGCT
TATTGTTCTTAATGAATTAGAATTCGATAATTTTCATATTAAAGAAGGAA
AGCATTTAAGAAAATCAAAAATGTTTAATCATATCAAAGACAGTGGTTAT
TATGCAACAAACGAAGAAATTGAAATTTTTCTTGAAAGCTGCACTCTATG
TAAGGAAATTACTGCACAAACCAAACGTAATTCATATAAAAAAAGAAATA
TAATTAATAAATTACCGGAAGAAGAAGAAGAAGAAGAAGAAGAAGAAGAA
GAAGAAGAAGAAGAAGAAGAACAAGAAGAAGAAGTAGAAAAACCGACAAT
ATCGGAAGAAGAAGAAGAAGAAACACCTGCAGTATCAGAAGAAGAAAAAG
AAGAGGAAGAACAAGAAGAAGATAAAGAAAAAGATAAAGAAAAAAAAAATA
GAAGAAGATACAGAAACAGGAAAAAAAAAAAGATGAAGTTATGAATGAACG
TCAAGATGGTATTGAAATTAGTGAAAAAACAAATGATAAACCAACAGAAA
AAACAAAAGTTAAAAAGAATGTTTCACGAGTCAAAGATATTAATCTTCAT

AAAAAAGGAAATCCTATTACCCCAAAAAAAATAAAAAATTTCAGTCAACA
ACAATTACGTTTAGCTTCTGGTGTTTCAAATGAAAAAAGAGCAATAATTA
CTCTTAAAAAATAATAAAACTTTATTTAATTAAAAAAATAATTAAAAAGA
AAATTAAATATATATTAATTTTTAAATTATTTGAAATGTTTTTTTCTTGA
GTTACTGACTTTTCCATACCGTTATTTGAGCCATCGAGCTTTGGATTGTA
AATTGTTGATAAAAATTCAGAATCAGATGCACCATACTTTGGAGTACACT
TTGTTGTTTTAAAATCAAGATTGTTTTGAATGTTTTCATTCTTACAATAG
TGGCACAATGGATCTGTACATGTAAATCTATTGGATTTCTCTTGTGTAAT
GTTTGTAATATGATATACATTTTCAAATTTTGAACAAATGTTGAATTTTT
CAATAATTGTTTTACTATTTTTTGTACAATCCATTGAAAGAGAAGTAATT
TCAACAAAGCTGTCTTCCACTCTAAACAAATAACCATCATCAGTTTCATT
ATATTCTTTATAAAGAATAGACTCTCCAAACTCGTCATTAATACATTCAT
TAAGTTTAATTAAGTTTGTTAAATTACGAAAATTGTTACATCTTGAATTA
TTTGTTTCAATGTCATCATAAAAAAGCGCATGAGTACTATTTGTTTCAAC
TCTTCCATGAATATTACATTCATCATAAATTACGGCGCCTGTCATTGACT
TTTCAACACAGTTTTCTTTTTGATACATAACAATAAGAAGTTGAAAATAT
TTTACTTCAATCTGATCATTTTGAAGAACAGAAGATTTTGATACAATAAA
AAAAAAAAGAAAAAATAATAACGTTGTTGAGTACATTATATACAATGAAA
ATAATAAATGAACAAAAAATAATTTTTTATTATAATATATTAAACCCCAC
CCCACAACCACACCTAAAAAAAAGTTAATACTTTTTTAAATATAATTTTT
TTAATAGTTATTTTAATACGTTTTTAACATAAAATATATTTTAATTTTTT
TTTTAATTTTTTAGAAAATTTTTTTTTCATAATTATTTTAATACTTTTTT
ACAAAAAATATTTTTAAAATTTTAAAAATTTTTTTTTTATAATTATTTTA
ATAGCTTAATTACAAAAACAATTTAATAATTTTTTTAATTTTTTTTTCAT
AATATTTTTAATAGCTTTTTTACAAATTATTTTTACGAAAATTTTTGTTA
TATTTTTTTATAATTATTTTAATACATTTTTAACATTAAATAATTATATA
ATTTTTTAATACAATAATTACAGTTATAAATTCATATTATTTAAATATTT
TTAAATGAGTTTATTTAGAAATTAAGTTTATATTTTATTTAATTAGTTTT
GACTCTCAGAATGTATAGACACTTTTTAGAACACTGTGCACAAAGATGAA
GTTTACATTCTTGGTTCAAATTACATTTAGTTCCGTTCTTACTACACATG
GCACACTTCTTGTTGATTACCGAATCATTGAAACATGGAGGGCAAAGTTT
AGAATTAATAACAACATTATATATGTTTATGTTTATTCCTAAACAATTAT
AGCAAATATTGTTTTGACATCCATCTCTTGTACATGAAATATATAAATTT
TTTCCTTGACACAAAGAATGACAATTAATACACTTTACTTCTAAGAGAGA
ATAATTTTCTGGATTTGAAAGGGCATCTGTTGAAGAAACTAAAAAGAAAT
CAATTTTGGAAGAAGTTTTATTGACTTCTTCTTTAATAAACTTTTTTAAA
TCGGCAATTTCATTGGAATTTATTTGGGTAGATTCGGATTGAATTTTGTA
ATAATTGCTATTTACTTTTTCGAAATCGAT

REMI expression vectors

pDM1220 – No tag

CTCGAGACTAGAGCTAGATAAAAAAAAATTTTTATTTATTTTTATTTATTT
TGAATTAAATAGATTACAAATTAATTAATCCCATCAAATCTTTAAAAAAA
AATGGTTTAAAAAAACTTGGGTTGGTTAATTATTATTTGAAAATTTTAAA
ACCCAAATTAAAAAAAAAAAAATGGGATTCAAAAATTTTTTTTTTTTTTT
TTTTTTTTTTTTTTTTTTTTTTTCAGATTGCATAAAAAGATTTTTTTTTT
TTTTTTTTCTTATTTCTTAAAACAAATAAATTAAATTAAATAAAAAATAA
AAATCAGATCTAGTACTAGTTAAATAAATAAATTATTTAATAAATAATAA

AAAAACAAATTGTTGTAATAATCTAATATTTTCTTTTTTTTTTAATTTTT
TTTTTTTAAATCTTAATAATTATTAAGTTATTTTAATTTTTTTTTTTTTT
TTTTTTTTTTTTTTTTTTTTTTTTCTATCAAAAAAATCAAATATATTTAA
AAAATTTATTATTTACAGATACATTTTGAATGGTGAAGATAAATATATGC
ATTAGATGTAAAACAGCCAAAGAGTATGAAAATCAAAAAGATAAAGCTTA
GTGCTCGTCCAGCCGGCAGAGGCGGTTTGCGTATTGGGCGCTCTTCCGCT
TCCTCGCTCACTGACTCGCTGCGCTCGGTCGTTCGGCTGCGGCGAGCGGT
ATCAGCTCACTCAAAGGCGGTAATACGGTTATCCACAGAATCAGGGGATA
ACGCAGGAAAGAACATGTGAGCAAAAGGCCAGCAAAAGGCCAGGAACCGT
AAAAAGGCCGCGTTGCTGGCGTTTTTCCATAGGCTCCGCCCCCCTGACGA
GCATCACAAAAATCGACGCTCAAGTCAGAGGTGGCGAAACCCGACAGGAC
TATAAAGATACCAGGCGTTTCCCCCTGGAAGCTCCCTCGTGCGCTCTCCT
GTTCCGACCCTGCCGCTTACCGGATACCTGTCCGCCTTTCTCCCTTCGGG
AAGCGTGGCGCTTTCTCATAGCTCACGCTGTAGGTATCTCAGTTCGGTGT
AGGTCGTTCGCTCCAAGCTGGGCTGTGTGCACGAACCCCCCGTTCAGCCC
GACCGCTGCGCCTTATCCGGTAACTATCGTCTTGAGTCCAACCCGGTAAG
ACACGACTTATCGCCACTGGCAGCAGCCACTGGTAACAGGATTAGCAGAG
CGAGGTATGTAGGCGGTGCTACAGAGTTCTTGAAGTGGTGGCCTAACTAC
GGCTACACTAGAAGGACAGTATTTGGTATCTGCGCTCTGCTGAAGCCAGT
TACCTTCGGAAAAAGAGTTGGTAGCTCTTGATCCGGCAAACAAACCACCG
CTGGTAGCGGTGGTTTTTTTGTTTGCAAGCAGCAGATTACGCGCAGAAAA
AAAGGATCTCAAGAAGATCCTTTGATCTTTTCTACGGGGTCTGACGCTCA
GTGGAACGAAAACTCACGTTAAGGGATTTTGGTCATGAGATTATCAAAAA
GGATCTTCACCTAGATCCTTTTAAATTAAAAATGAAGTTTTAAATCAATC
TAAAGTATATATGAGTAAACTTGGTCTGACAGTTACCAATGCTTAATCAG
TGAGGCACCTATCTCAGCGATCTGTCTATTTCGTTCATCCATAGTTGCCT
GACTCCCCGTCGTGTAGATAACTACGATACGGGAGGGCTTACCATCTGGC
CCCAGTGCTGCAATGATACCGCGAGACCCACGCTCACCGGCTCCAGATTT
ATCAGCAATAAACCAGCCAGCCGGAAGGGCCGAGCGCAGAAGTGGTCCTG
CAACTTTATCCGCCTCCATCCAGTCTATTAATTGTTGCCGGGAAGCTAGA
GTAAGTAGTTCGCCAGTTAATAGTTTGCGCAACGTTGTTGCCATTGCTAC
AGGCATCGTGGTGTCACGCTCGTCGTTTGGTATGGCTTCATTCAGCTCCG
GTTCCCAACGATCAAGGCGAGTTACATGATCCCCCATGTTGTGCAAAAAA
GCGGTTAGCTCCTTCGGTCCTCCGATCGTTGTCAGAAGTAAGTTGGCCGC
AGTGTTATCACTCATGGTTATGGCAGCACTGCATAATTCTCTTACTGTCA
TGCCATCCGTAAGATGCTTTTCTGTGACTGGTGAGTACTCAACCAAGTCA
TTCTGAGAATAGTGTATGCGGCGACCGAGTTGCTCTTGCCCGGCGTCAAT
ACGGGATAATACCGCGCCACATAGCAGAACTTTAAAAGTGCTCATCATTG
GAAAACGTTCTTCGGGGCGAAAACTCTCAAGGATCTTACCGCTGTTGAGA
TCCAGTTCGATGTAACCCACTCGTGCACCCAACTGATCTTCAGCATCTTT
TACTTTCACCAGCGTTTCTGGGTGAGCAAAAACAGGAAGGCAAAATGCCG
CAAAAAAGGGAATAAGGGCGACACGGAAATGTTGAATACTCATACTCTTC
CTTTTTCAATATTATTGAAGCATTTATCAGGGTTATTGTCTCATGAGCGG
ATACATATTTGAATGTATTTAGAAAAATAAACAAATAGGGGTTCCGCGCA
CATTTCCCCGAAAAGTGCCACCTGACGCGCCCTGTAGCGGGATCCATTTT
ATTTAATATACTAAATAATAAAAAAGTTAAAAAATGATCATTGGATAAAT
TTTTTATAATTATAAATAAAGATAATAATTTTTTTTTTAACAAAACTAAA
AATAAAAATAATAAAATAATTGTTAAAATAGGTTTTTTTTTTTTTTTTTT
TTTTTTAATAAATGGTATTTATTAATTTATTTGTTGTGTGTGTTTTTTTT
TTTATAATATTTTTTTTTTTAGCATTGAATTAAGAAGAAATCAAATTGAT

GCGGCCGCTTAGTTAGCCTCCCCCATCTCCCGATCCGGACGAGTGCTGGG
GCGTCGGTTTCCACTATCGGCGAGTACTTCTACACAGCCATCGGTCCAGA
CGGCCGCGCTTCTGCGGGCGATTTGTGTACGCCCGACAGTCCCGGCTCCG
GATCGGACGATTGCGTCGCATCGACCCTGCGCCCAAGCTGCATCATCGAA
ATTGCCGTCAACCAAGCTCTGATAGAGTTGGTCAAGACCAATGCGGAGCA
TATACGCCCGGAGCCGCGGCGATCCTGCAAGCTCCGGATGCCTCCGCTCG
AAGTAGCGCGTCTGCTGCTCCATACAAGCCAACCACGGCCTCCAGAAGAA
GATGTTGGCGACCTCGTATTGGGAATCCCCGAACATCGCCTCGCTCCAGT
CAATGACCGCTGTTATGCGGCCATTGTCCGTCAGGACATTGTTGGAGCCG
AAATCCGCGTGCACGAGGTGCCGGACTTCGGGGCAGTCCTCGGCCCAAAG
CATCAGCTCATCGAGAGCCTGCGCGACGGACGCACTGACGGTGTCGTCCA
TCACAGTTTGCCAGTGATACACATGGGGATCAGCAATCGCGCATATGAAA
TCACGCCATGTAGTGTATTGACCGATTCCTTGCGGTCCGAATGGGCCGAA
CCCGCTCGTCTGGCTAAGATCGGCCGCAGCGATCGCATCCATGGCCTCCG
CGACCGGCTGCAGAACAGCGGGCAGTTCGGTTTCAGGCAGGTCTTGCAAC
GTGACACCCTGTGCACGGCGGGAGATGCAATAGGTCAGGCTCTCGCTAAA
TTCCCCAATGTCAAGCACTTCCGGAATCGGGAGCGCGGCCGATGCAAAGT
GCCGATAAACATAACGATCTTTGTAGAAACCATCGGCGCAGCTATTTACC
CGCAGGACATATCCACGCCCTCCTACATCGAAGCTGAAAGCACGAGATTC
TTCGCCCTCCGAGAGCTGCATCAGGTCGGAGACGCTGTCGAACTTTTCGA
TCAGAAACTTCTCGACAGACGTCGCGGTGAGTTCAGGCTTTTTCATTAGG
TCTTGTTGAGAAATGTTAAATTGATCCATTTTTTGCTAGCTTTATTGTTG
TATTATATTTAAATATTTGTTTATTAAAACTATAGAAAAAAAAAAAAAAT
GATTTAATTTTTCATTTTATACCCCTTGAAAAAAAAAAATAAAAAAAATT
AAAAACCCAATAAAATCCCATTTTTCAAAAAAAATAATTAAAAAAATTCA
AAAAAAAATAAAATTTTAAAATGGCTTGTTTTTTTTGATGAAATTTTTAA
TGGGAGTGGGTTACCCAATTATTTTTTTGAAAAATATGAAAAAATCTTTG
AAACCACCATAACCAGCAATTCAAATAAAATTCAAAAAAAAAAAAAAAAA
AACATTTTATGACTTAATTATAATTATTTTTTTTGGTGTTTGATTAACAT
ATCTTTAAATTAATTACTATTATTATTATTTTTTTTTTTTTATTATTATT
TTATTTATTTATTTTTTTTTTTTTTATTTTTTTTTTTATTTTTTTTTTGA
ATCCCAATTTTTTAAATTTCTGAACACAACAAAGAAA

pDM1351 – GFP (N)

CTCGAGACTAGAGCTAGATAAAAAAAATTTTTATTTATTTTTATTTATTT
TGAATTAAATAGATTACAAATTAATTAATCCCATCAAATCTTTAAAAAAA
AATGGTTTAAAAAAACTTGGGTTGGTTAATTATTATTTGAAAATTTTAAA
ACCCAAATTAAAAAAAAAAAAATGGGATTCAAAAATTTTTTTTTTTTTTT
TTTTTTTTTTTTTTTTTTTTTTCAGATTGCATAAAAAGATTTTTTTTT
TTTTTTTTCTTATTTCTTAAAACAAATAAATTAAATTAAATAAAAAATAA
AAATCAGATCCAAAAAATGTCAAAAGGTGAAGAATTATTTACAGGTGTTG
TTCCAATTTTAGTTGAATTAGATGGTGATGTTAATGGTCATAAATTTTCA
GTTTCAGGTGAAGGTGAAGGTGATGCAACATATGGTAAATTAACATTAAA
ATTTATTTGTACAACAGGTAAATTACCAGTTCCATGGCCAACATTAGTTA
CAACATTTACATATGGTGTTCAATGTTTTTCAAGATATCCAGATCATATG
AAACAACATGATTTTTTTAAATCAGCAATGCCAGAAGGTTATGTTCAAGA
AAGAACAATTTTTTTTAAAGATGATGGTAATTATAAAACAAGAGCAGAAG
TTAAATTTGAAGGTGATACATTAGTTAATAGAATTGAATTAAAAGGTATT
GATTTTAAAGAAGATGGTAATATTTTAGGTCATAAATTAGAATATAATTA

TAATTCACATAATGTTTATATTATGGCAGATAAACAAAAAAATGGTATTA
AAGTTAATTTTAAAATTAGACATAATATTGAAGATGGTTCAGTTCAATTA
GCAGATCATTATCAACAAAATACACCAATTGGTGATGGTCCAGTTTTATT
ACCAGATAATCATTATTTATCAACACAATCAGCATTATCAAAAGATCCAA
ATGAAAAAAGAGATCATATGGTTTTATTAGAATTTGTTACAGCAGCAGGT
ATTACACATGGTATGGATGAATTATATAAAGGTGGTTCAGGAGGTAGTAG
ATCTTCAACTAGTTAAATAAATAAATTATTTAATAAATAATAAAAAAACA
AATTGTTGTAATAATCTAATATTTTCTTTTTTTTTTAATTTTTTTTTTTT
AAATCTTAATAATTATTAAGTTATTTAATTTTTTTTTTTTTTTTTTTTTT
TTTTTTTTTTTTTTTTTCTATCAAAAAAATCAAATATATTTAAAAAATTT
ATTATTTACAGATACATTTTGAATGGTGAAGATAAATATATGCATTAGAT
GTAAAACAGCCAAAGAGTATGAAAATCAAAAAGATAAAGCTTAGTGCTCG
TCCAGCCGGCAGAGGCGGTTTGCGTATTGGGCGCTCTTCCGCTTCCTCGC
TCACTGACTCGCTGCGCTCGGTCGTTCGGCTGCGGCGAGCGGTATCAGCT
CACTCAAAGGCGGTAATACGGTTATCCACAGAATCAGGGGATAACGCAGG
AAAGAACATGTGAGCAAAAGGCCAGCAAAAGGCCAGGAACCGTAAAAAGG
CCGCGTTGCTGGCGTTTTTCCATAGGCTCCGCCCCCCTGACGAGCATCAC
AAAAATCGACGCTCAAGTCAGAGGTGGCGAAACCCGACAGGACTATAAAG
ATACCAGGCGTTTCCCCCTGGAAGCTCCCTCGTGCGCTCTCCTGTTCCGA
CCCTGCCGCTTACCGGATACCTGTCCGCCTTTCTCCCTTCGGGAAGCGTG
GCGCTTTCTCATAGCTCACGCTGTAGGTATCTCAGTTCGGTGTAGGTCGT
TCGCTCCAAGCTGGGCTGTGTGCACGAACCCCCCGTTCAGCCCGACCGCT
GCGCCTTATCCGGTAACTATCGTCTTGAGTCCAACCCGGTAAGACACGAC
TTATCGCCACTGGCAGCAGCCACTGGTAACAGGATTAGCAGAGCGAGGTA
TGTAGGCGGTGCTACAGAGTTCTTGAAGTGGTGGCCTAACTACGGCTACA
CTAGAAGGACAGTATTTGGTATCTGCGCTCTGCTGAAGCCAGTTACCTTC
GGAAAAAGAGTTGGTAGCTCTTGATCCGGCAAACAAACCACCGCTGGTAG
CGGTGGTTTTTTTGTTTGCAAGCAGCAGATTACGCGCAGAAAAAAAGGAT
CTCAAGAAGATCCTTTGATCTTTTCTACGGGGTCTGACGCTCAGTGGAAC
GAAAACTCACGTTAAGGGATTTTGGTCATGAGATTATCAAAAAGGATCTT
CACCTAGATCCTTTTAAATTAAAAATGAAGTTTTAAATCAATCTAAAGTA
TATATGAGTAAACTTGGTCTGACAGTTACCAATGCTTAATCAGTGAGGCA
CCTATCTCAGCGATCTGTCTATTTCGTTCATCCATAGTTGCCTGACTCCC
CGTCGTGTAGATAACTACGATACGGGAGGGCTTACCATCTGGCCCCAGTG
CTGCAATGATACCGCGAGACCCACGCTCACCGGCTCCAGATTTATCAGCA
ATAAACCAGCCAGCCGGAAGGGCCGAGCGCAGAAGTGGTCCTGCAACTTT
ATCCGCCTCCATCCAGTCTATTAATTGTTGCCGGGAAGCTAGAGTAAGTA
GTTCGCCAGTTAATAGTTTGCGCAACGTTGTTGCCATTGCTACAGGCATC
GTGGTGTCACGCTCGTCGTTTGGTATGGCTTCATTCAGCTCCGGTTCCCA
ACGATCAAGGCGAGTTACATGATCCCCCATGTTGTGCAAAAAAGCGGTTA
GCTCCTTCGGTCCTCCGATCGTTGTCAGAAGTAAGTTGGCCGCAGTGTTA
TCACTCATGGTTATGGCAGCACTGCATAATTCTCTTACTGTCATGCCATC
CGTAAGATGCTTTTCTGTGACTGGTGAGTACTCAACCAAGTCATTCTGAG
AATAGTGTATGCGGCGACCGAGTTGCTCTTGCCCGGCGTCAATACGGGAT
AATACCGCGCCACATAGCAGAACTTTAAAAGTGCTCATCATTGGAAAACG
TTCTTCGGGGCGAAAACTCTCAAGGATCTTACCGCTGTTGAGATCCAGTT
CGATGTAACCCACTCGTGCACCCAACTGATCTTCAGCATCTTTTACTTTC
ACCAGCGTTTCTGGGTGAGCAAAAACAGGAAGGCAAAATGCCGCAAAAAA
GGGAATAAGGGCGACACGGAAATGTTGAATACTCATACTCTTCCTTTTTC
AATATTATTGAAGCATTTATCAGGGTTATTGTCTCATGAGCGGATACATA

TTTGAATGTATTTAGAAAAATAAACAAATAGGGGTTCCGCGCACATTTCC
CCGAAAAGTGCCACCTGACGCGCCCTGTAGCGGGATCCATTTTATTTAAT
ATACTAAATAATAAAAAAGTTAAAAAATGATCATTGGATAAATTTTTTAT
AATTATAAATAAAGATAATAATTTTTTTTTTAACAAAACTAAAAATAAAA
ATAATAAAATAATTGTTAAAATAGGTTTTTTTTTTTTTTTTTTTTTTTA
ATAAATGGTATTTATTAATTTATTTGTTGTGTGTGTTTTTTTTTTTTATAA
TATTTTTTTTTTTAGCATTGAATTAAGAAGAAATCAAATTGATGCGGCCG
CTTAGTTAGCCTCCCCCATCTCCCGATCCGGACGAGTGCTGGGGCGTCGG
TTTCCACTATCGGCGAGTACTTCTACACAGCCATCGGTCCAGACGGCCGC
GCTTCTGCGGGCGATTTGTGTACGCCCGACAGTCCCGGCTCCGGATCGGA
CGATTGCGTCGCATCGACCCTGCGCCCAAGCTGCATCATCGAAATTGCCG
TCAACCAAGCTCTGATAGAGTTGGTCAAGACCAATGCGGAGCATATACGC
CCGGAGCCGCGGCGATCCTGCAAGCTCCGGATGCCTCCGCTCGAAGTAGC
GCGTCTGCTGCTCCATACAAGCCAACCACGGCCTCCAGAAGAAGATGTTG
GCGACCTCGTATTGGGAATCCCCGAACATCGCCTCGCTCCAGTCAATGAC
CGCTGTTATGCGGCCATTGTCCGTCAGGACATTGTTGGAGCCGAAATCCG
CGTGCACGAGGTGCCGGACTTCGGGGCAGTCCTCGGCCCAAAGCATCAGC
TCATCGAGAGCCTGCGCGACGGACGCACTGACGGTGTCGTCCATCACAGT
TTGCCAGTGATACACATGGGGATCAGCAATCGCGCATATGAAATCACGCC
ATGTAGTGTATTGACCGATTCCTTGCGGTCCGAATGGGCCGAACCCGCTC
GTCTGGCTAAGATCGGCCGCAGCGATCGCATCCATGGCCTCCGCGACCGG
CTGCAGAACAGCGGGCAGTTCGGTTTCAGGCAGGTCTTGCAACGTGACAC
CCTGTGCACGGCGGGAGATGCAATAGGTCAGGCTCTCGCTAAATTCCCCA
ATGTCAAGCACTTCCGGAATCGGGAGCGCGGCCGATGCAAAGTGCCGATA
AACATAACGATCTTTGTAGAAACCATCGGCGCAGCTATTTACCCGCAGGA
CATATCCACGCCCTCCTACATCGAAGCTGAAAGCACGAGATTCTTCGCCC
TCCGAGAGCTGCATCAGGTCGGAGACGCTGTCGAACTTTTCGATCAGAAA
CTTCTCGACAGACGTCGCGGTGAGTTCAGGCTTTTTCATTAGGTCTTGTT
GAGAAATGTTAAATTGATCCATTTTTTGCTAGCTTTATTGTTGTATTATA
TTTAAATATTTGTTTATTAAAACTATAGAAAAAAAAAAAAAAATGATTTAA
TTTTTCATTTTATACCCCTTGAAAAAAAAAAATAAAAAAAAATTAAAAACC
CAATAAAATCCCATTTTTCAAAAAAAATAATTAAAAAAATTCAAAAAAAA
ATAAAATTTTAAAATGGCTTGTTTTTTTTGATGAAATTTTTAATGGGAGT
GGGTTACCCAATTATTTTTTTGAAAAATATGAAAAAATCTTTGAAACCAC
CATAACCAGCAATTCAAATAAAATTCAAAAAAAAAAAAAAAAAAACATTT
TATGACTTAATTATAATTATTTTTTTTGGTGTTTGATTAACATATCTTTA
AATTAATTACTATTATTATTATTTTTTTTTTTTTATTATTATTTTATTTA
TTTATTTTTTTTTTTTTTATTTTTTTTTTTTATTTTTTTTTTGAATCCCAA
TTTTTTAAATTTCTGAACACAACAAAGAAA

pDM1352 – GFP (C)

CTCGAGACTAGAGCTAGATAAAAAAAATTTTTATTTATTTTTATTTATTT
TGAATTAAATAGATTACAAATTAATTAATCCCATCAAATCTTTAAAAAAA
AATGGTTTAAAAAAACTTGGGTTGGTTAATTATTATTTGAAAATTTTAAA
ACCCAAATTAAAAAAAAAAAAATGGGATTCAAAAATTTTTTTTTTTTTTT
TTTTTTTTTTTTTTTTTTTTTTTCAGATTGCATAAAAAGATTTTTTTTTT
TTTTTTTTCTTATTTCTTAAAACAAATAAATTAAATTAAATAAAAAATAA
AAATCAGATCTTCAACTAGTGGTGGTTCAGGAGGTAGTTCAAAAGGTGAA
GAATTATTTACAGGTGTTGTTCCAATTTTAGTTGAATTAGATGGTGATGT

TAATGGTCATAAATTTTCAGTTTCAGGTGAAGGTGAAGGTGATGCAACAT
ATGGTAAATTAACATTAAAATTTATTTGTACAACAGGTAAATTACCAGTT
CCATGGCCAACATTAGTTACAACATTTACATATGGTGTTCAATGTTTTTC
AAGATATCCAGATCATATGAAACAACATGATTTTTTTAAATCAGCAATGC
CAGAAGGTTATGTTCAAGAAAGAACAATTTTTTTTAAAGATGATGGTAAT
TATAAAACAAGAGCAGAAGTTAAATTTGAAGGTGATACATTAGTTAATAG
AATTGAATTAAAAGGTATTGATTTTAAAGAAGATGGTAATATTTTAGGTC
ATAAATTAGAATATAATTATAATTCACATAATGTTTATATTATGGCAGAT
AAACAAAAAAATGGTATTAAAGTTAATTTTAAAATTAGACATAATATTGA
AGATGGTTCAGTTCAATTAGCAGATCATTATCAACAAAATACACCAATTG
GTGATGGTCCAGTTTTATTACCAGATAATCATTATTTATCAACACAATCA
GCATTATCAAAAGATCCAAATGAAAAAAGAGATCATATGGTTTTATTAGA
ATTTGTTACAGCAGCAGGTATTACACATGGTATGGATGAATTATATAAAT
AAGCTAGTTAAATAAATAAATTATTTAATAAATAATAAAAAAACAAATTG
TTGTAATAATCTAATATTTTCTTTTTTTTTTAATTTTTTTTTTTTAAATC
TTAATAATTATTAAGTTATTTTAATTTTTTTTTTTTTTTTTTTTTTTTTT
TTTTTTTTTTTTCTATCAAAAAAATCAAATATATTTAAAAAATTTATTAT
TTACAGATACATTTTGAATGGTGAAGATAAATATATGCATTAGATGTAAA
ACAGCCAAAGAGTATGAAAATCAAAAAGATAAAGCTTAGTGCTCGTCCAG
CCGGCAGAGGCGGTTTGCGTATTGGGCGCTCTTCCGCTTCCTCGCTCACT
GACTCGCTGCGCTCGGTCGTTCGGCTGCGGCGAGCGGTATCAGCTCACTC
AAAGGCGGTAATACGGTTATCCACAGAATCAGGGGATAACGCAGGAAAGA
ACATGTGAGCAAAAGGCCAGCAAAAGGCCAGGAACCGTAAAAAGGCCGCG
TTGCTGGCGTTTTTCCATAGGCTCCGCCCCCCTGACGAGCATCACAAAAA
TCGACGCTCAAGTCAGAGGTGGCGAAACCCGACAGGACTATAAAGATACC
AGGCGTTTCCCCCTGGAAGCTCCCTCGTGCGCTCTCCTGTTCCGACCCTG
CCGCTTACCGGATACCTGTCCGCCTTTCTCCCTTCGGGAAGCGTGGCGCT
TTCTCATAGCTCACGCTGTAGGTATCTCAGTTCGGTGTAGGTCGTTCGCT
CCAAGCTGGGCTGTGTGCACGAACCCCCCGTTCAGCCCGACCGCTGCGCC
TTATCCGGTAACTATCGTCTTGAGTCCAACCCGGTAAGACACGACTTATC
GCCACTGGCAGCAGCCACTGGTAACAGGATTAGCAGAGCGAGGTATGTAG
GCGGTGCTACAGAGTTCTTGAAGTGGTGGCCTAACTACGGCTACACTAGA
AGGACAGTATTTGGTATCTGCGCTCTGCTGAAGCCAGTTACCTTCGGAAA
AAGAGTTGGTAGCTCTTGATCCGGCAAACAAACCACCGCTGGTAGCGGTG
GTTTTTTTGTTTGCAAGCAGCAGATTACGCGCAGAAAAAAAGGATCTCAA
GAAGATCCTTTGATCTTTTCTACGGGGTCTGACGCTCAGTGGAACGAAAA
CTCACGTTAAGGGATTTTGGTCATGAGATTATCAAAAAGGATCTTCACCT
AGATCCTTTTAAATTAAAAATGAAGTTTTAAATCAATCTAAAGTATATAT
GAGTAAACTTGGTCTGACAGTTACCAATGCTTAATCAGTGAGGCACCTAT
CTCAGCGATCTGTCTATTTCGTTCATCCATAGTTGCCTGACTCCCCGTCG
TGTAGATAACTACGATACGGGAGGGCTTACCATCTGGCCCCAGTGCTGCA
ATGATACCGCGAGACCCACGCTCACCGGCTCCAGATTTATCAGCAATAAA
CCAGCCAGCCGGAAGGGCCGAGCGCAGAAGTGGTCCTGCAACTTTATCCG
CCTCCATCCAGTCTATTAATTGTTGCCGGGAAGCTAGAGTAAGTAGTTCG
CCAGTTAATAGTTTGCGCAACGTTGTTGCCATTGCTACAGGCATCGTGGT
GTCACGCTCGTCGTTTGGTATGGCTTCATTCAGCTCCGGTTCCCAACGAT
CAAGGCGAGTTACATGATCCCCCATGTTGTGCAAAAAAGCGGTTAGCTCC
TTCGGTCCTCCGATCGTTGTCAGAAGTAAGTTGGCCGCAGTGTTATCACT
CATGGTTATGGCAGCACTGCATAATTCTCTTACTGTCATGCCATCCGTAA
GATGCTTTTCTGTGACTGGTGAGTACTCAACCAAGTCATTCTGAGAATAG

TGTATGCGGCGACCGAGTTGCTCTTGCCCGGCGTCAATACGGGATAATAC
CGCGCCACATAGCAGAACTTTAAAAGTGCTCATCATTGGAAAACGTTCTT
CGGGGCGAAAACTCTCAAGGATCTTACCGCTGTTGAGATCCAGTTCGATG
TAACCCACTCGTGCACCCAACTGATCTTCAGCATCTTTTACTTTCACCAG
CGTTTCTGGGTGAGCAAAAACAGGAAGGCAAAATGCCGCAAAAAAGGGAA
TAAGGGCGACACGGAAATGTTGAATACTCATACTCTTCCTTTTTCAATAT
TATTGAAGCATTTATCAGGGTTATTGTCTCATGAGCGGATACATATTTGA
ATGTATTTAGAAAAATAAACAAATAGGGGTTCCGCGCACATTTCCCCGAA
AAGTGCCACCTGACGCGCCCTGTAGCGGATCCATTTTATTTAATATACT
AAATAATAAAAAAGTTAAAAAATGATCATTGGATAAATTTTTTATAATTA
TAAATAAAGATAATAATTTTTTTTTTAACAAAACTAAAAATAAAAATAAT
AAAATAATTGTTAAAATAGGTTTTTTTTTTTTTTTTTTTTTTTTAATAAA
TGGTATTTATTAATTTATTTGTTGTGTGTGTTTTTTTTTTTATAATATTT
TTTTTTTTAGCATTGAATTAAGAAGAAATCAAATTGATGCGGCCGCTTAG
TTAGCCTCCCCCATCTCCCGATCCGGACGAGTGCTGGGGCGTCGGTTTCC
ACTATCGGCGAGTACTTCTACACAGCCATCGGTCCAGACGGCCGCGCTTC
TGCGGGCGATTTGTGTACGCCCGACAGTCCCGGCTCCGGATCGGACGATT
GCGTCGCATCGACCCTGCGCCCAAGCTGCATCATCGAAATTGCCGTCAAC
CAAGCTCTGATAGAGTTGGTCAAGACCAATGCGGAGCATATACGCCCGGA
GCCGCGGCGATCCTGCAAGCTCCGGATGCCTCCGCTCGAAGTAGCGCGTC
TGCTGCTCCATACAAGCCAACCACGGCCTCCAGAAGAAGATGTTGGCGAC
CTCGTATTGGGAATCCCCGAACATCGCCTCGCTCCAGTCAATGACCGCTG
TTATGCGGCCATTGTCCGTCAGGACATTGTTGGAGCCGAAATCCGCGTGC
ACGAGGTGCCGGACTTCGGGGCAGTCCTCGGCCCAAAGCATCAGCTCATC
GAGAGCCTGCGCGACGGACGCACTGACGGTGTCGTCCATCACAGTTTGCC
AGTGATACACATGGGGATCAGCAATCGCGCATATGAAATCACGCCATGTA
GTGTATTGACCGATTCCTTGCGGTCCGAATGGGCCGAACCCGCTCGTCTG
GCTAAGATCGGCCGCAGCGATCGCATCCATGGCCTCCGCGACCGGCTGCA
GAACAGCGGGCAGTTCGGTTTCAGGCAGGTCTTGCAACGTGACACCCTGT
GCACGGCGGGAGATGCAATAGGTCAGGCTCTCGCTAAATTCCCCAATGTC
AAGCACTTCCGGAATCGGGAGCGCGGCCGATGCAAAGTGCCGATAAACAT
AACGATCTTTGTAGAAACCATCGGCGCAGCTATTTACCCGCAGGACATAT
CCACGCCCTCCTACATCGAAGCTGAAAGCACGAGATTCTTCGCCCTCCGA
GAGCTGCATCAGGTCGGAGACGCTGTCGAACTTTTCGATCAGAAACTTCT
CGACAGACGTCGCGGTGAGTTCAGGCTTTTTCATTAGGTCTTGTTGAGAA
ATGTTAAATTGATCCATTTTTTGCTAGCTTTATTGTTGTATTATATTTAA
ATATTTGTTTATTAAAACTATAGAAAAAAAAAAAAAATGATTTAATTTTT
CATTTTATACCCCTTGAAAAAAAAAATAAAAAAAATTAAAAACCCAATA
AAATCCCATTTTTCAAAAAAAATAATTAAAAAAATTCAAAAAAAAAATAAA
ATTTTAAAATGGCTTGTTTTTTTTGATGAAATTTTTAATGGGAGTGGGTT
ACCCAATTATTTTTTTGAAAAATATGAAAAAATCTTTGAAACCACCATAA
CCAGCAATTCAAATAAAATTCAAAAAAAAAAAAAAAAAAACATTTTATGA
CTTAATTATAATTATTTTTTTTGGTGTTTGATTAACATATCTTTAAATTA
ATTACTATTATTATTATTTTTTTTTTTTATTATTATTTTATTTATTTAT
TTTTTTTTTTTTTATTTTTTTTTATTTTTTTTTTTGAATCCCAATTTTT
TAAATTTCTGAACACAACAAAGAAA

pDM1259 – mCherry (N)

CTCGAGACTAGAGCTAGATAAAAAAAATTTTATTTATTTTTATTTATTT

TGAATTAAATAGATTACAAATTAATTAATCCCATCAAATCTTTAAAAAAA
AATGGTTTAAAAAAACTTGGGTTGGTTAATTATTATTTGAAAATTTTAAA
ACCCAAATTAAAAAAAAAAAAATGGGATTCAAAAATTTTTTTTTTTTTTT
TTTTTTTTTTTTTTTTTTTTTTTTTCAGATTGCATAAAAAGATTTTTTTTT
TTTTTTTTCTTATTTCTTAAAACAAATAAATTAAATTAAATAAAAAATAA
AAATCAGATCCAAAAAATGGTTTCAAAAGGTGAAGAAGATAATATGGCAA
TTATTAAAGAATTTATGAGATTTAAAGTTCATATGGAAGGTTCAGTTAAT
GGTCATGAATTTGAAATTGAAGGTGAAGGTGAAGGTAGACCATATGAAGG
TACACAAACAGCAAAATTAAAAGTTACAAAAGGTGGTCCATTACCATTTG
CATGGGATATTTTATCACCACAATTTATGTATGGTTCAAAAGCATATGTT
AAACATCCAGCAGATATTCCAGATTATTTAAAATTATCATTTCCAGAAGG
TTTTAAATGGGAAAGAGTTATGAATTTTGAAGATGGTGGTGTTGTTACAG
TTACACAAGATTCATCATTACAAGATGGTGAATTTATTTATAAAGTTAAA
TTAAGAGGTACAAATTTTCCATCAGATGGTCCAGTTATGCAAAAAAAAAC
AATGGGTTGGGAAGCATCATCAGAAAGAATGTATCCAGAAGATGGTGCAT
TAAAAGGTGAAATTAAACAAAGATTAAAATTAAAAGATGGTGGTCATTAT
GATGCAGAAGTTAAAACAACATATAAAGCAAAAAAACCAGTTCAATTACC
AGGTGCATATAATGTTAATATTAAATTAGATATTACATCACATAATGAAG
ATTATACAATTGTTGAACAATATGAAAGAGCAGAAGGTAGACATTCAACA
GGTGGTATGGATGAATTATATAAAGGTGGTTCAGGAGGTAGTAGATCTTC
AACTAGTTAAATAAATAAATTATTTAATAAATAATAAAAAAACAAATTGT
TGTAATAATCTAATATTTTCTTTTTTTTTTAATTTTTTTTTTTTAAATCT
TAATAATTATTAAGTTATTTTAATTTTTTTTTTTTTTTTTTTTTTTTTTT
TTTTTTTTTTTCTATCAAAAAAATCAAATATATTTAAAAAATTTATTATT
TACAGATACATTTTGAATGGTGAAGATAAATATATGCATTAGATGTAAAA
CAGCCAAAGAGTATGAAAATCAAAAAGATAAAGCTTAGTGCTCGTCCAGC
CGGCAGAGGCGGTTTGCGTATTGGGCGCTCTTCCGCTTCCTCGCTCACTG
ACTCGCTGCGCTCGGTCGTTCGGCTGCGGCGAGCGGTATCAGCTCACTCA
AAGGCGGTAATACGGTTATCCACAGAATCAGGGGATAACGCAGGAAAGAA
CATGTGAGCAAAAGGCCAGCAAAAGGCCAGGAACCGTAAAAAGGCCGCGT
TGCTGGCGTTTTTCCATAGGCTCCGCCCCCCTGACGAGCATCACAAAAAT
CGACGCTCAAGTCAGAGGTGGCGAAACCCGACAGGACTATAAAGATACCA
GGCGTTTCCCCCTGGAAGCTCCCTCGTGCGCTCTCCTGTTCCGACCCTGC
CGCTTACCGGATACCTGTCCGCCTTTCTCCCTTCGGGAAGCGTGGCGCTT
TCTCATAGCTCACGCTGTAGGTATCTCAGTTCGGTGTAGGTCGTTCGCTC
CAAGCTGGGCTGTGTGCACGAACCCCCCGTTCAGCCCGACCGCTGCGCCT
TATCCGGTAACTATCGTCTTGAGTCCAACCCGGTAAGACACGACTTATCG
CCACTGGCAGCAGCCACTGGTAACAGGATTAGCAGAGCGAGGTATGTAGG
CGGTGCTACAGAGTTCTTGAAGTGGTGGCCTAACTACGGCTACACTAGAA
GGACAGTATTTGGTATCTGCGCTCTGCTGAAGCCAGTTACCTTCGGAAAA
AGAGTTGGTAGCTCTTGATCCGGCAAACAAACCACCGCTGGTAGCGGTGG
TTTTTTTGTTTGCAAGCAGCAGATTACGCGCAGAAAAAAAGGATCTCAAG
AAGATCCTTTGATCTTTTCTACGGGGTCTGACGCTCAGTGGAACGAAAAC
TCACGTTAAGGGATTTTGGTCATGAGATTATCAAAAAGGATCTTCACCTA
GATCCTTTTAAATTAAAAATGAAGTTTTAAATCAATCTAAAGTATATATG
AGTAAACTTGGTCTGACAGTTACCAATGCTTAATCAGTGAGGCACCTATC
TCAGCGATCTGTCTATTTCGTTCATCCATAGTTGCCTGACTCCCCGTCGT
GTAGATAACTACGATACGGGAGGGCTTACCATCTGGCCCCAGTGCTGCAA
TGATACCGCGAGACCCACGCTCACCGGCTCCAGATTTATCAGCAATAAAC
CAGCCAGCCGGAAGGGCCGAGCGCAGAAGTGGTCCTGCAACTTTATCCGC

CTCCATCCAGTCTATTAATTGTTGCCGGGAAGCTAGAGTAAGTAGTTCGC
CAGTTAATAGTTTGCGCAACGTTGTTGCCATTGCTACAGGCATCGTGGTG
TCACGCTCGTCGTTTGGTATGGCTTCATTCAGCTCCGGTTCCCAACGATC
AAGGCGAGTTACATGATCCCCCATGTTGTGCAAAAAAGCGGTTAGCTCCT
TCGGTCCTCCGATCGTTGTCAGAAGTAAGTTGGCCGCAGTGTTATCACTC
ATGGTTATGGCAGCACTGCATAATTCTCTTACTGTCATGCCATCCGTAAG
ATGCTTTTCTGTGACTGGTGAGTACTCAACCAAGTCATTCTGAGAATAGT
GTATGCGGCGACCGAGTTGCTCTTGCCCGGCGTCAATACGGGATAATACC
GCGCCACATAGCAGAACTTTAAAAGTGCTCATCATTGGAAAACGTTCTTC
GGGGCGAAAACTCTCAAGGATCTTACCGCTGTTGAGATCCAGTTCGATGT
AACCCACTCGTGCACCCAACTGATCTTCAGCATCTTTTACTTTCACCAGC
GTTTCTGGGTGAGCAAAAACAGGAAGGCAAAATGCCGCAAAAAAGGGAAT
AAGGGCGACACGGAAATGTTGAATACTCATACTCTTCCTTTTTCAATATT
ATTGAAGCATTTATCAGGGTTATTGTCTCATGAGCGGATACATATTTGAA
TGTATTTAGAAAAATAAACAAATAGGGGTTCCGCGCACATTTCCCCGAAA
AGTGCCACCTGACGCGCCCTGTAGCGGGATCCATTTTATTTAATATACTA
AATAATAAAAAAGTTAAAAAATGATCATTGGATAAATTTTTTATAATTAT
AAATAAAGATAATAATTTTTTTTTTAACAAAACTAAAAATAAAAATAATA
AAATAATTGTTAAAATAGGTTTTTTTTTTTTTTTTTTTTTTTAATAAAT
GGTATTTATTAATTTATTTGTTGTGTGTGTTTTTTTTTTTTATAATATTTT
TTTTTTTAGCATTGAATTAAGAAGAAATCAAATTGATGCGGCCGCTTAGT
TAGCCTCCCCCATCTCCCGATCCGGACGAGTGCTGGGGCGTCGGTTTCCA
CTATCGGCGAGTACTTCTACACAGCCATCGGTCCAGACGGCCGCGCTTCT
GCGGGCGATTTGTGTACGCCCGACAGTCCCGGCTCCGGATCGGACGATTG
CGTCGCATCGACCCTGCGCCCAAGCTGCATCATCGAAATTGCCGTCAACC
AAGCTCTGATAGAGTTGGTCAAGACCAATGCGGAGCATATACGCCCGGAG
CCGCGGCGATCCTGCAAGCTCCGGATGCCTCCGCTCGAAGTAGCGCGTCT
GCTGCTCCATACAAGCCAACCACGGCCTCCAGAAGAAGATGTTGGCGACC
TCGTATTGGGAATCCCCGAACATCGCCTCGCTCCAGTCAATGACCGCTGT
TATGCGGCCATTGTCCGTCAGGACATTGTTGGAGCCGAAATCCGCGTGCA
CGAGGTGCCGGACTTCGGGGCAGTCCTCGGCCCAAAGCATCAGCTCATCG
AGAGCCTGCGCGACGGACGCACTGACGGTGTCGTCCATCACAGTTTGCCA
GTGATACACATGGGGATCAGCAATCGCGCATATGAAATCACGCCATGTAG
TGTATTGACCGATTCCTTGCGGTCCGAATGGGCCGAACCCGCTCGTCTGG
CTAAGATCGGCCGCAGCGATCGCATCCATGGCCTCCGCGACCGGCTGCAG
AACAGCGGGCAGTTCGGTTTCAGGCAGGTCTTGCAACGTGACACCCTGTG
CACGGCGGGAGATGCAATAGGTCAGGCTCTCGCTAAATTCCCCAATGTCA
AGCACTTCCGGAATCGGGAGCGCGGCCGATGCAAAGTGCCGATAAACATA
ACGATCTTTGTAGAAACCATCGGCGCAGCTATTTACCCGCAGGACATATC
CACGCCCTCCTACATCGAAGCTGAAAGCACGAGATTCTTCGCCCTCCGAG
AGCTGCATCAGGTCGGAGACGCTGTCGAACTTTTCGATCAGAAACTTCTC
GACAGACGTCGCGGTGAGTTCAGGCTTTTTCATTAGGTCTTGTTGAGAAA
TGTTAAATTGATCCATTTTTTGCTAGCTTTATTGTTGTATTATATTTAAA
TATTTGTTTATTAAAACTATAGAAAAAAAAAAAAAAATGATTTAATTTTTC
ATTTTATACCCCTTGAAAAAAAAAAATAAAAAAAATTAAAAACCCAATAA
AATCCCATTTTTCAAAAAAAATAATTAAAAAAATTCAAAAAAAAATAAAA
TTTTAAAATGGCTTGTTTTTTTTGATGAAATTTTTAATGGGAGTGGGTTA
CCCAATTATTTTTTTGAAAAATATGAAAAAATCTTTGAAACCACCATAAC
CAGCAATTCAAATAAAATTCAAAAAAAAAAAAAAAAAAAACATTTTATGAC
TTAATTATAATTATTTTTTTTGGTGTTTGATTAACATATCTTTAAATTAA

TTACTATTATTATTATTTTTTTTTTTTTATTATTATTTATTTATTTATT
TTTTTTTTTTTTATTTTTTTTTTTATTTTTTTTTTGAATCCCAATTTTTT
AAATTTCTGAACACAACAAAGAAA

pDM1305 – mCherry (C)

CTCGAGACTAGAGCTAGATAAAAAAAATTTTTATTTATTTTTATTTATTT
TGAATTAAATAGATTACAAATTAATTAATCCCATCAAATCTTTAAAAAAA
AATGGTTTAAAAAAACTTGGGTTGGTTAATTATTATTTGAAAATTTTAAA
ACCCAAATTAAAAAAAAAAAAATGGGATTCAAAAATTTTTTTTTTTTTTT
TTTTTTTTTTTTTTTTTTTTTTTCAGATTGCATAAAAAGATTTTTTTTTT
TTTTTTTTCTTATTTCTTAAAACAAATAAATTAAATTAAATAAAAAATAA
AAATCAGATCTTCAACTAGTGGTGGTTCAGGAGGTAGTGTTTCAAAAGGT
GAAGAAGATAATATGGCAATTATTAAAGAATTTATGAGATTTAAAGTTCA
TATGGAAGGTTCAGTTAATGGTCATGAATTTGAAATTGAAGGTGAAGGTG
AAGGTAGACCATATGAAGGTACACAAACAGCAAAATTAAAAGTTACAAAA
GGTGGTCCATTACCATTTGCATGGGATATTTTATCACCACAATTTATGTA
TGGTTCAAAAGCATATGTTAAACATCCAGCAGATATTCCAGATTATTTAA
AATTATCATTTCCAGAAGGTTTTAAATGGGAAAGAGTTATGAATTTTGAA
GATGGTGGTGTTGTTACAGTTACACAAGATTCATCATTACAAGATGGTGA
ATTTATTTATAAAGTTAAATTAAGAGGTACAAATTTTCCATCAGATGGTC
CAGTTATGCAAAAGAAAACAATGGGTTGGGAAGCATCATCAGAAAGAATG
TATCCAGAAGATGGTGCATTAAAAGGTGAAATTAAACAAAGATTAAAATT
AAAAGATGGTGGTCATTATGATGCAGAAGTTAAAACAACATATAAAGCAA
AAAAACCAGTTCAATTACCAGGTGCATATAATGTTAATATTAAATTAGAT
ATTACATCACATAATGAAGATTATACAATTGTTGAACAATATGAAAGAGC
AGAAGGTAGACATTCAACAGGTGGTATGGATGAATTATATAAATAAGCTA
GTTAAATAAATAAATTATTTAATAAATAATAAAAAAAACAAATTGTTGTAA
TAATCTAATATTTTCTTTTTTTTTTAATTTTTTTTTTTTAAATCTTAATA
ATTATTAAGTTATTTTAATTTTTTTTTTTTTTTTTTTTTTTTTTTTTTTT
TTTTTTCTATCAAAAAAATCAAATATATTTAAAAAATTTATTATTTACAG
ATACATTTTGAATGGTGAAGATAAATATATGCATTAGATGTAAAACAGCC
AAAGAGTATGAAAATCAAAAAGATAAAGCTTAGTGCTCGTCCAGCCGGCA
GAGGCGGTTTGCGTATTGGGCGCTCTTCCGCTTCCTCGCTCACTGACTCG
CTGCGCTCGGTCGTTCGGCTGCGGCGAGCGGTATCAGCTCACTCAAAGGC
GGTAATACGGTTATCCACAGAATCAGGGGATAACGCAGGAAAGAACATGT
GAGCAAAAGGCCAGCAAAAGGCCAGGAACCGTAAAAAGGCCGCGTTGCTG
GCGTTTTTCCATAGGCTCCGCCCCCCTGACGAGCATCACAAAAATCGACG
CTCAAGTCAGAGGTGGCGAAACCCGACAGGACTATAAAGATACCAGGCGT
TTCCCCCTGGAAGCTCCCTCGTGCGCTCTCCTGTTCCGACCCTGCCGCTT
ACCGGATACCTGTCCGCCTTTCTCCCTTCGGGAAGCGTGGCGCTTTCTCA
TAGCTCACGCTGTAGGTATCTCAGTTCGGTGTAGGTCGTTCGCTCCAAGC
TGGGCTGTGTGCACGAACCCCCCGTTCAGCCCGACCGCTGCGCCTTATCC
GGTAACTATCGTCTTGAGTCCAACCCGGTAAGACACGACTTATCGCCACT
GGCAGCAGCCACTGGTAACAGGATTAGCAGAGCGAGGTATGTAGGCGGTG
CTACAGAGTTCTTGAAGTGGTGGCCTAACTACGGCTACACTAGAAGGACA
GTATTTGGTATCTGCGCTCTGCTGAAGCCAGTTACCTTCGGAAAAAGAGT
TGGTAGCTCTTGATCCGGCAAACAAACCACCGCTGGTAGCGGTGGTTTTT
TTGTTTGCAAGCAGCAGATTACGCGCAGAAAAAAAGGATCTCAAGAAGAT
CCTTTGATCTTTTCTACGGGGTCTGACGCTCAGTGGAACGAAAACTCACG

TTAAGGGATTTTGGTCATGAGATTATCAAAAAGGATCTTCACCTAGATCC
TTTTAAATTAAAAATGAAGTTTTAAATCAATCTAAAGTATATATGAGTAA
ACTTGGTCTGACAGTTACCAATGCTTAATCAGTGAGGCACCTATCTCAGC
GATCTGTCTATTTCGTTCATCCATAGTTGCCTGACTCCCCGTCGTGTAGA
TAACTACGATACGGGAGGGCTTACCATCTGGCCCCAGTGCTGCAATGATA
CCGCGAGACCCACGCTCACCGGCTCCAGATTTATCAGCAATAAACCAGCC
AGCCGGAAGGGCCGAGCGCAGAAGTGGTCCTGCAACTTTATCCGCCTCCA
TCCAGTCTATTAATTGTTGCCGGGAAGCTAGAGTAAGTAGTTCGCCAGTT
AATAGTTTGCGCAACGTTGTTGCCATTGCTACAGGCATCGTGGTGTCACG
CTCGTCGTTTGGTATGGCTTCATTCAGCTCCGGTTCCCAACGATCAAGGC
GAGTTACATGATCCCCCATGTTGTGCAAAAAAGCGGTTAGCTCCTTCGGT
CCTCCGATCGTTGTCAGAAGTAAGTTGGCCGCAGTGTTATCACTCATGGT
TATGGCAGCACTGCATAATTCTCTTACTGTCATGCCATCCGTAAGATGCT
TTTCTGTGACTGGTGAGTACTCAACCAAGTCATTCTGAGAATAGTGTATG
CGGCGACCGAGTTGCTCTTGCCCGGCGTCAATACGGGATAATACCGCGCC
ACATAGCAGAACTTTAAAAGTGCTCATCATTGGAAAACGTTCTTCGGGGC
GAAAACTCTCAAGGATCTTACCGCTGTTGAGATCCAGTTCGATGTAACCC
ACTCGTGCACCCAACTGATCTTCAGCATCTTTTACTTTCACCAGCGTTTC
TGGGTGAGCAAAAACAGGAAGGCAAAATGCCGCAAAAAAGGGAATAAGGG
CGACACGGAAATGTTGAATACTCATACTCTTCCTTTTTCAATATTATTGA
AGCATTTATCAGGGTTATTGTCTCATGAGCGGATACATATTTGAATGTAT
TTAGAAAAATAAACAAATAGGGGTTCCGCGCACATTTCCCCGAAAAGTGC
CACCTGACGCGCCCTGTAGCGGGATCCATTTTATTTAATATACTAAATAA
TAAAAAAGTTAAAAAATGATCATTGGATAAATTTTTTATAATTATAAATA
AAGATAATAATTTTTTTTTTTAACAAAACTAAAAATAAAAATAATAAAATA
ATTGTTAAAATAGGTTTTTTTTTTTTTTTTTTTTTTTTAATAAATGGTAT
TTATTAATTTATTTGTTGTGTGTGTTTTTTTTTTTATAATATTTTTTTT
TTAGCATTGAATTAAGAAGAAATCAAATTGATGCGGCCGCTTAGTTAGCC
TCCCCCATCTCCCGATCCGGACGAGTGCTGGGGCGTCGGTTTCCACTATC
GGCGAGTACTTCTACACAGCCATCGGTCCAGACGGCCGCGCTTCTGCGGG
CGATTTGTGTACGCCCGACAGTCCCGGCTCCGGATCGGACGATTGCGTCG
CATCGACCCTGCGCCCAAGCTGCATCATCGAAATTGCCGTCAACCAAGCT
CTGATAGAGTTGGTCAAGACCAATGCGGAGCATATACGCCCGGAGCCGCG
GCGATCCTGCAAGCTCCGGATGCCTCCGCTCGAAGTAGCGCGTCTGCTGC
TCCATACAAGCCAACCACGGCCTCCAGAAGAAGATGTTGGCGACCTCGTA
TTGGGAATCCCCGAACATCGCCTCGCTCCAGTCAATGACCGCTGTTATGC
GGCCATTGTCCGTCAGGACATTGTTGGAGCCGAAATCCGCGTGCACGAGG
TGCCGGACTTCGGGGCAGTCCTCGGCCCAAAGCATCAGCTCATCGAGAGC
CTGCGCGACGGACGCACTGACGGTGTCGTCCATCACAGTTTGCCAGTGAT
ACACATGGGGATCAGCAATCGCGCATATGAAATCACGCCATGTAGTGTAT
TGACCGATTCCTTGCGGTCCGAATGGGCCGAACCCGCTCGTCTGGCTAAG
ATCGGCCGCAGCGATCGCATCCATGGCCTCCGCGACCGGCTGCAGAACAG
CGGGCAGTTCGGTTTCAGGCAGGTCTTGCAACGTGACACCCTGTGCACGG
CGGGAGATGCAATAGGTCAGGCTCTCGCTAAATTCCCCAATGTCAAGCAC
TTCCGGAATCGGGAGCGCGGCCGATGCAAAGTGCCGATAAACATAACGAT
CTTTGTAGAAACCATCGGCGCAGCTATTTACCCGCAGGACATATCCACGC
CCTCCTACATCGAAGCTGAAAGCACGAGATTCTTCGCCCTCCGAGAGCTG
CATCAGGTCGGAGACGCTGTCGAACTTTTCGATCAGAAACTTCTCGACAG
ACGTCGCGGTGAGTTCAGGCTTTTTCATTAGGTCTTGTTGAGAAATGTTA
AATTGATCCATTTTTTGCTAGCTTTATTGTTGTATTATATTTAAATATTT

GTTTATTAAAACTATAGAAAAAAAAAAAAAAATGATTTAATTTTTCATTTT
ATACCCCTTGAAAAAAAAAAATAAAAAAAATTAAAAACCCAATAAAATCC
CATTTTTCAAAAAAAATAATTAAAAAAATTCAAAAAAAAATAAAATTTTA
AAATGGCTTGTTTTTTTTGATGAAATTTTTAATGGGAGTGGGTTACCCAA
TTATTTTTTTGAAAAATATGAAAAAATCTTTGAAACCACCATAACCAGCA
ATTCAAATAAAATTCAAAAAAAAAAAAAAAAAAAACATTTTATGACTTAAT
TATAATTATTTTTTTTGGTGTTTGATTAACATATCTTTAAATTAATTACT
ATTATTATTATTTTTTTTTTTTTATTATTATTTTATTTATTTATTTTTTT
TTTTTTTATTTTTTTTTTTATTTTTTTTTTGAATCCCAATTTTTTAAATT
TCTGAACACAACAAAGAAA

pPI465 – mNeon (N)

CTCGAGACTAGAGCTAGATAAAAAAAATTTTTATTTATTTTTATTTATTT
TGAATTAAATAGATTACAAATTAATTAATCCCATCAAATCTTTAAAAAAA
AATGGTTTAAAAAAACTTGGGTTGGTTAATTATTATTTGAAAATTTTAAA
ACCCAAATTAAAAAAAAAAAAATGGGATTCAAAAATTTTTTTTTTTTTTT
TTTTTTTTTTTTTTTTTTTTTTTTCAGATTGCATAAAAAGATTTTTTTTT
TTTTTTTTCTTATTTCTTAAAACAAATAAATTAAATTAAATAAAAAAATAA
AAATCAGATCCAAAAAATGGTGAGTAAAGGTGAAGAAGATAATATGGCAT
CGTTACCAGCTACACATGAGTTACATATATTCGGTAGCATTAATGGTGTT
GATTTTGATATGGTGGGACAAGGTACCGGTAATCCTAATGATGGTTACGA
AGAACTAAATTTAAAATCGACTAAAGGTGACTTACAATTTTCTCCATGGA
TTTTAGTGCCACATATAGGGTATGGTTTTCATCAATACTTACCATATCCA
GATGGTATGTCACCATTTCAAGCTGCAATGGTTGATGGATCAGGTTATCA
AGTTCATAGAACAATGCAATTTGAAGATGGTGCTTCATTAACTGTTAATT
ATAGATACACATATGAAGGCTCACATATTAAAGGTGAAGCTCAAGTTAAA
GGTACTGGTTTCCCAGCCGATGGCCCAGTTATGACAAATAGTTTAACAGC
AGCAGATTGGTGTAGATCCAAAAAAACTTATCCAAATGATAAAACAATTA
TTTCAACTTTTAAATGGTCATATACAACCGGTAATGGTAAACGTTATCGT
TCAACAGCCCGTACAACATATACTTTTGCTAAACCAATGGCAGCTAATTA
TTTAAAAAATCAACCAATGTATGTTTTTCGTAAAACAGAGTTAAAACATT
CAAAAACAGAACTTAATTTTAAAGAATGGCAAAAAGCATTTACAGACGTT
ATGGGTATGGATGAACTTTATAAGGGTGGTTCAGGAGGTAGTAGATCTTC
AACTAGTTAAATAAATAAATTATTTAATAAATAATAAAAAAACAAATTGT
TGTAATAATCTAATATTTTCTTTTTTTTTTAATTTTTTTTTTTTAAATCT
TAATAATTATTAAGTTATTTTAATTTTTTTTTTTTTTTTTTTTTTTTTTT
TTTTTTTTTTTCTATCAAAAAAATCAAATATATTTAAAAAATTTATTATT
TACAGATACATTTTGAATGGTGAAGATAAATATATGCATTAGATGTAAAA
CAGCCAAAGAGTATGAAAATCAAAAAGATAAAGCTTAGTGCTCGTCCAGC
CGGCAGAGGCGGTTTGCGTATTGGGCGCTCTTCCGCTTCCTCGCTCACTG
ACTCGCTGCGCTCGGTCGTTCGGCTGCGGCGAGCGGTATCAGCTCACTCA
AAGGCGGTAATACGGTTATCCACAGAATCAGGGGATAACGCAGGAAAGAA
CATGTGAGCAAAAGGCCAGCAAAAGGCCAGGAACCGTAAAAAGGCCGCGT
TGCTGGCGTTTTTCCATAGGCTCCGCCCCCCTGACGAGCATCACAAAAAT
CGACGCTCAAGTCAGAGGTGGCGAAACCCGACAGGACTATAAAGATACCA
GGCGTTTCCCCCTGGAAGCTCCCTCGTGCGCTCTCCTGTTCCGACCCTGC
CGCTTACCGGATACCTGTCCGCCTTTCTCCCTTCGGGAAGCGTGGCGCTT
TCTCATAGCTCACGCTGTAGGTATCTCAGTTCGGTGTAGGTCGTTCGCTC
CAAGCTGGGCTGTGTGCACGAACCCCCCGTTCAGCCCGACCGCTGCGCCT

TATCCGGTAACTATCGTCTTGAGTCCAACCCGGTAAGACACGACTTATCG
CCACTGGCAGCAGCCACTGGTAACAGGATTAGCAGAGCGAGGTATGTAGG
CGGTGCTACAGAGTTCTTGAAGTGGTGGCCTAACTACGGCTACACTAGAA
GGACAGTATTTGGTATCTGCGCTCTGCTGAAGCCAGTTACCTTCGGAAAA
AGAGTTGGTAGCTCTTGATCCGGCAAACAAACCACCGCTGGTAGCGGTGG
TTTTTTTGTTTGCAAGCAGCAGATTACGCGCAGAAAAAAAGGATCTCAAG
AAGATCCTTTGATCTTTTCTACGGGGTCTGACGCTCAGTGGAACGAAAAC
TCACGTTAAGGGATTTTGGTCATGAGATTATCAAAAAGGATCTTCACCTA
GATCCTTTTAAATTAAAAATGAAGTTTTAAATCAATCTAAAGTATATATG
AGTAAACTTGGTCTGACAGTTACCAATGCTTAATCAGTGAGGCACCTATC
TCAGCGATCTGTCTATTTCGTTCATCCATAGTTGCCTGACTCCCCGTCGT
GTAGATAACTACGATACGGGAGGGCTTACCATCTGGCCCCAGTGCTGCAA
TGATACCGCGAGACCCACGCTCACCGGCTCCAGATTTATCAGCAATAAAC
CAGCCAGCCGGAAGGGCCGAGCGCAGAAGTGGTCCTGCAACTTTATCCGC
CTCCATCCAGTCTATTAATTGTTGCCGGGAAGCTAGAGTAAGTAGTTCGC
CAGTTAATAGTTTGCGCAACGTTGTTGCCATTGCTACAGGCATCGTGGTG
TCACGCTCGTCGTTTGGTATGGCTTCATTCAGCTCCGGTTCCCAACGATC
AAGGCGAGTTACATGATCCCCCATGTTGTGCAAAAAAGCGGTTAGCTCCT
TCGGTCCTCCGATCGTTGTCAGAAGTAAGTTGGCCGCAGTGTTATCACTC
ATGGTTATGGCAGCACTGCATAATTCTCTTACTGTCATGCCATCCGTAAG
ATGCTTTTCTGTGACTGGTGAGTACTCAACCAAGTCATTCTGAGAATAGT
GTATGCGGCGACCGAGTTGCTCTTGCCCGGCGTCAATACGGGATAATACC
GCGCCACATAGCAGAACTTTAAAAGTGCTCATCATTGGAAAACGTTCTTC
GGGGCGAAAACTCTCAAGGATCTTACCGCTGTTGAGATCCAGTTCGATGT
AACCCACTCGTGCACCCAACTGATCTTCAGCATCTTTTACTTTCACCAGC
GTTTCTGGGTGAGCAAAAACAGGAAGGCAAAATGCCGCAAAAAAGGGAAT
AAGGGCGACACGGAAATGTTGAATACTCATACTCTTCCTTTTTCAATATT
ATTGAAGCATTTATCAGGGTTATTGTCTCATGAGCGGATACATATTTGAA
TGTATTTAGAAAAATAAACAAATAGGGGTTCCGCGCACATTTCCCCGAAA
AGTGCCACCTGACGCGCCCTGTAGCGGGATCCATTTTATTTAATATACTA
AATAATAAAAAAGTTAAAAAATGATCATTGGATAAATTTTTTATAATTAT
AAATAAAGATAATAATTTTTTTTTTAACAAAACTAAAAATAAAAATAATA
AAATAATTGTTAAAATAGGTTTTTTTTTTTTTTTTTTTTTTTTAATAAAT
GGTATTTATTAATTTATTTGTTGTGTGTGTTTTTTTTTTTATAATATTTT
TTTTTTTAGCATTGAATTAAGAAGAAATCAAATTGATGCGGCCGCTTAGT
TAGCCTCCCCCATCTCCCGATCCGGACGAGTGCTGGGGCGTCGGTTTCCA
CTATCGGCGAGTACTTCTACACAGCCATCGGTCCAGACGGCCGCGCTTCT
GCGGGCGATTTGTGTACGCCCGACAGTCCCGGCTCCGGATCGGACGATTG
CGTCGCATCGACCCTGCGCCCAAGCTGCATCATCGAAATTGCCGTCAACC
AAGCTCTGATAGAGTTGGTCAAGACCAATGCGGAGCATATACGCCCGGAG
CCGCGGCGATCCTGCAAGCTCCGGATGCCTCCGCTCGAAGTAGCGCGTCT
GCTGCTCCATACAAGCCAACCACGGCCTCCAGAAGAAGATGTTGGCGACC
TCGTATTGGGAATCCCCGAACATCGCCTCGCTCCAGTCAATGACCGCTGT
TATGCGGCCATTGTCCGTCAGGACATTGTTGGAGCCGAAATCCGCGTGCA
CGAGGTGCCGGACTTCGGGGCAGTCCTCGGCCCAAAGCATCAGCTCATCG
AGAGCCTGCGCGACGGACGCACTGACGGTGTCGTCCATCACAGTTTGCCA
GTGATACACATGGGGATCAGCAATCGCGCATATGAAATCACGCCATGTAG
TGTATTGACCGATTCCTTGCGGTCCGAATGGGCCGAACCCGCTCGTCTGG
CTAAGATCGGCCGCAGCGATCGCATCCATGGCCTCCGCGACCGGCTGCAG
AACAGCGGGCAGTTCGGTTTCAGGCAGGTCTTGCAACGTGACACCCTGTG

CACGGCGGGAGATGCAATAGGTCAGGCTCTCGCTAAATTCCCCAATGTCA
AGCACTTCCGGAATCGGGAGCGCGGCCGATGCAAAGTGCCGATAAACATA
ACGATCTTTGTAGAAACCATCGGCGCAGCTATTTACCCGCAGGACATATC
CACGCCCTCCTACATCGAAGCTGAAAGCACGAGATTCTTCGCCCTCCGAG
AGCTGCATCAGGTCGGAGACGCTGTCGAACTTTTCGATCAGAAACTTCTC
GACAGACGTCGCGGTGAGTTCAGGCTTTTTCATTAGGTCTTGTTGAGAAA
TGTTAAATTGATCCATTTTTTGCTAGCTTTATTGTTGTATTATATTTAAA
TATTTGTTTATTAAAACTATAGAAAAAAAAAAAAAATGATTTAATTTTTC
ATTTTATACCCCTTGAAAAAAAAAAATAAAAAAAATTAAAAACCCAATAA
AATCCCATTTTTCAAAAAAAATAATTAAAAAAATTCAAAAAAAAATAAAA
TTTTAAAATGGCTTGTTTTTTTTGATGAAATTTTTAATGGGAGTGGGTTA
CCCAATTATTTTTTTGAAAAATATGAAAAAATCTTTGAAACCACCATAAC
CAGCAATTCAAATAAAATTCAAAAAAAAAAAAAAAAAAACATTTTATGAC
TTAATTATAATTATTTTTTTTGGTGTTTGATTAACATATCTTTAAATTAA
TTACTATTATTATTATTTTTTTTTTTTTATTATTATTTTATTTATTTATT
TTTTTTTTTTTTATTTTTTTTTTTATTTTTTTTTTGAATCCCAATTTTTT
AAATTTCTGAACACAACAAAGAAA

pPI471 – mNeon (C)

CTCGAGACTAGAGCTAGATAAAAAAAATTTTTATTTATTTTTATTTATTT
TGAATTAAATAGATTACAAATTAATTAATCCCATCAAATCTTTAAAAAAA
AATGGTTTAAAAAAACTTGGGTTGGTTAATTATTATTTGAAAATTTTAAA
ACCCAAATTAAAAAAAAAAAAATGGGATTCAAAAATTTTTTTTTTTTTTT
TTTTTTTTTTTTTTTTTTTTTTCAGATTGCATAAAAAGATTTTTTTTTT
TTTTTTTTCTTATTTCTTAAAACAAATAAATTAAATTAAATAAAAAAATAA
AAATCAGATCTTCAACTAGTGGTGGTTCAGGAGGTAGTGTGAGTAAAGGT
GAAGAAGATAATATGGCATCGTTACCAGCTACACATGAGTTACATATATT
CGGTAGCATTAATGGTGTTGATTTTGATATGGTGGGACAAGGTACCGGTA
ATCCTAATGATGGTTACGAAGAACTAAATTTAAAATCGACTAAAGGTGAC
TTACAATTTTCTCCATGGATTTTAGTGCCACATATAGGGTATGGTTTTCA
TCAATACTTACCATATCCAGATGGTATGTCACCATTTCAAGCTGCAATGG
TTGATGGATCAGGTTATCAAGTTCATAGAACAATGCAATTTGAAGATGGT
GCTTCATTAACTGTTAATTATAGATACACATATGAAGGCTCACATATTAA
AGGTGAAGCTCAAGTTAAAGGTACTGGTTTCCCAGCCGATGGCCCAGTTA
TGACAAATAGTTTAACAGCAGCAGATTGGTGTAGATCCAAAAAAACTTAT
CCAAATGATAAAACAATTATTTCAACTTTTAAATGGTCATATACAACCGG
TAATGGTAAACGTTATCGTTCAACAGCCCGTACAACATATACTTTTGCTA
AACCAATGGCAGCTAATTATTTAAAAAATCAACCAATGTATGTTTTTCGT
AAAACAGAGTTAAAACATTCAAAAACAGAACTTAATTTTAAAGAATGGCA
AAAAGCATTTACAGACGTTATGTAAGCTAGTTTAAATAAATAAATTATTT
AATAAATAATAAAAAAACAAATTGTTGTAATAATCTAATATTTTCTTTTT
TTTTTAATTTTTTTTTTTTTAAATCTTAATAATTATTAAGTTATTTTAATT
TTTTTTTTTTTTTTTTTTTTTTTTTTTTTTTTTTTTTCTATCAAAAAAATC
AAATATATTTAAAAAATTTATTATTTACAGATACATTTTGAATGGTGAAG
ATAAATATATGCATTAGATGTAAAACAGCCAAAGAGTATGAAAATCAAAA
AGATAAAGCTTAGTGCTCGTCCAGCCGGCAGAGGCGGTTTGCGTATTGGG
CGCTCTTCCGCTTCCTCGCTCACTGACTCGCTGCGCTCGGTCGTTCGGCT
GCGGCGAGCGGTATCAGCTCACTCAAAGGCGGTAATACGGTTATCCACAG
AATCAGGGGATAACGCAGGAAAGAACATGTGAGCAAAAGGCCAGCAAAAG

GCCAGGAACCGTAAAAAGGCCGCGTTGCTGGCGTTTTTCCATAGGCTCCG
CCCCCCTGACGAGCATCACAAAAATCGACGCTCAAGTCAGAGGTGGCGAA
ACCCGACAGGACTATAAAGATACCAGGCGTTTCCCCCTGGAAGCTCCCTC
GTGCGCTCTCCTGTTCCGACCCTGCCGCTTACCGGATACCTGTCCGCCTT
TCTCCCTTCGGGAAGCGTGGCGCTTTCTCATAGCTCACGCTGTAGGTATC
TCAGTTCGGTGTAGGTCGTTCGCTCCAAGCTGGGCTGTGTGCACGAACCC
CCCGTTCAGCCCGACCGCTGCGCCTTATCCGGTAACTATCGTCTTGAGTC
CAACCCGGTAAGACACGACTTATCGCCACTGGCAGCAGCCACTGGTAACA
GGATTAGCAGAGCGAGGTATGTAGGCGGTGCTACAGAGTTCTTGAAGTGG
TGGCCTAACTACGGCTACACTAGAAGGACAGTATTTGGTATCTGCGCTCT
GCTGAAGCCAGTTACCTTCGGAAAAAGAGTTGGTAGCTCTTGATCCGGCA
AACAAACCACCGCTGGTAGCGGTGGTTTTTTTGTTTGCAAGCAGCAGATT
ACGCGCAGAAAAAAAGGATCTCAAGAAGATCCTTTGATCTTTTCTACGGG
GTCTGACGCTCAGTGGAACGAAAACTCACGTTAAGGGATTTTGGTCATGA
GATTATCAAAAAGGATCTTCACCTAGATCCTTTTAAATTAAAAATGAAGT
TTTAAATCAATCTAAAGTATATATGAGTAAACTTGGTCTGACAGTTACCA
ATGCTTAATCAGTGAGGCACCTATCTCAGCGATCTGTCTATTTCGTTCAT
CCATAGTTGCCTGACTCCCCGTCGTGTAGATAACTACGATACGGGAGGGC
TTACCATCTGGCCCCAGTGCTGCAATGATACCGCGAGACCCACGCTCACC
GGCTCCAGATTTATCAGCAATAAACCAGCCAGCCGGAAGGGCCGAGCGCA
GAAGTGGTCCTGCAACTTTATCCGCCTCCATCCAGTCTATTAATTGTTGC
CGGGAAGCTAGAGTAAGTAGTTCGCCAGTTAATAGTTTGCGCAACGTTGT
TGCCATTGCTACAGGCATCGTGGTGTCACGCTCGTCGTTTGGTATGGCTT
CATTCAGCTCCGGTTCCCAACGATCAAGGCGAGTTACATGATCCCCCATG
TTGTGCAAAAAAGCGGTTAGCTCCTTCGGTCCTCCGATCGTTGTCAGAAG
TAAGTTGGCCGCAGTGTTATCACTCATGGTTATGGCAGCACTGCATAATT
CTCTTACTGTCATGCCATCCGTAAGATGCTTTTCTGTGACTGGTGAGTAC
TCAACCAAGTCATTCTGAGAATAGTGTATGCGGCGACCGAGTTGCTCTTG
CCCGGCGTCAATACGGGATAATACCGCGCCACATAGCAGAACTTTAAAAG
TGCTCATCATTGGAAAACGTTCTTCGGGGCGAAAACTCTCAAGGATCTTA
CCGCTGTTGAGATCCAGTTCGATGTAACCCACTCGTGCACCCAACTGATC
TTCAGCATCTTTTACTTTCACCAGCGTTTCTGGGTGAGCAAAAACAGGAA
GGCAAAATGCCGCAAAAAAGGGAATAAGGGCGACACGGAAATGTTGAATA
CTCATACTCTTCCTTTTTCAATATTATTGAAGCATTTATCAGGGTTATTG
TCTCATGAGCGGATACATATTTGAATGTATTTAGAAAAATAAACAAATAG
GGGTTCCGCGCACATTTCCCCGAAAAGTGCCACCTGACGCGCCCTGTAGC
GGGATCCATTTTATTTAATATACTAAATAATAAAAAAGTTAAAAAATGAT
CATTGGATAAATTTTTTATAATTATAAATAAAGATAATAATTTTTTTTTT
AACAAAACTAAAAATAAAAATAATAAAATAATTGTTAAAATAGGTTTTTT
TTTTTTTTTTTTTTTTTAATAAATGGTATTTATTAATTTATTTGTTGTG
TGTGTTTTTTTTTTTATAATATTTTTTTTTTTAGCATTGAATTAAGAAGA
AATCAAATTGATGCGGCCGCTTAGTTAGCCTCCCCCATCTCCCGATCCGG
ACGAGTGCTGGGGCGTCGGTTTCCACTATCGGCGAGTACTTCTACACAGC
CATCGGTCCAGACGGCCGCGCTTCTGCGGGCGATTTGTGTACGCCCGACA
GTCCCGGCTCCGGATCGGACGATTGCGTCGCATCGACCCTGCGCCCAAGC
TGCATCATCGAAATTGCCGTCAACCAAGCTCTGATAGAGTTGGTCAAGAC
CAATGCGGAGCATATACGCCCGGAGCCGCGGCGATCCTGCAAGCTCCGGA
TGCCTCCGCTCGAAGTAGCGCGTCTGCTGCTCCATACAAGCCAACCACGG
CCTCCAGAAGAAGATGTTGGCGACCTCGTATTGGGAATCCCCGAACATCG
CCTCGCTCCAGTCAATGACCGCTGTTATGCGGCCATTGTCCGTCAGGACA

TTGTTGGAGCCGAAATCCGCGTGCACGAGGTGCCGGACTTCGGGGCAGTC
CTCGGCCCAAAGCATCAGCTCATCGAGAGCCTGCGCGACGGACGCACTGA
CGGTGTCGTCCATCACAGTTTGCCAGTGATACACATGGGGATCAGCAATC
GCGCATATGAAATCACGCCATGTAGTGTATTGACCGATTCCTTGCGGTCC
GAATGGGCCGAACCCGCTCGTCTGGCTAAGATCGGCCGCAGCGATCGCAT
CCATGGCCTCCGCGACCGGCTGCAGAACAGCGGGCAGTTCGGTTTCAGGC
AGGTCTTGCAACGTGACACCCTGTGCACGGCGGGAGATGCAATAGGTCAG
GCTCTCGCTAAATTCCCCAATGTCAAGCACTTCCGGAATCGGGAGCGCGG
CCGATGCAAAGTGCCGATAAACATAACGATCTTTGTAGAAACCATCGGCG
CAGCTATTTACCCGCAGGACATATCCACGCCCTCCTACATCGAAGCTGAA
AGCACGAGATTCTTCGCCCTCCGAGAGCTGCATCAGGTCGGAGACGCTGT
CGAACTTTTCGATCAGAAACTTCTCGACAGACGTCGCGGTGAGTTCAGGC
TTTTTCATTAGGTCTTGTTGAGAAATGTTAAATTGATCCATTTTTTGCTA
GCTTTATTGTTGTATTATATTTAAATATTTGTTTATTAAAACTATAGAAA
AAAAAAAAAAATGATTTAATTTTTCATTTTATACCCCTTGAAAAAAAAAA
ATAAAAAAAATTAAAAACCCAATAAAATCCCATTTTTCAAAAAAAATAAT
TAAAAAAATTCAAAAAAAAATAAAATTTTAAAATGGCTTGTTTTTTTTGA
TGAAATTTTTAATGGGAGTGGGTTACCCAATTATTTTTTTGAAAAATATG
AAAAAATCTTTGAAACCACCATAACCAGCAATTCAAATAAAATTCAAAAA
AAAAAAAAAAAAAACATTTTATGACTTAATTATAATTATTTTTTTTGGTG
TTTGATTAACATATCTTTAAATTAATTACTATTATTATTATTTTTTTTTT
TTTATTATTATTTTATTTATTTATTTTTTTTTTTTTTATTTTTTTTTTTA
TTTTTTTTTTGAATCCCAATTTTTTAAATTTCTGAACACAACAAAGAAA

pPI468 – mScarlet (N)

CTCGAGACTAGAGCTAGATAAAAAAAATTTTTATTTATTTTTATTTATTT
TGAATTAAATAGATTACAAATTAATTAATCCCATCAAATCTTTAAAAAAA
AATGGTTTAAAAAAACTTGGGTTGGTTAATTATTATTTGAAAATTTTAAA
ACCCAAATTAAAAAAAAAAAAATGGGATTCAAAAATTTTTTTTTTTTTTT
TTTTTTTTTTTTTTTTTTTTTTTCAGATTGCATAAAAAGATTTTTTTTTT
TTTTTTTTCTTATTTCTTAAAACAAATAAATTAAATTAAATAAAAAATAA
AAATCAGATCCAAAAAATGGTTTCAAAAGGTGAAGCCGTTATTAAAGAAT
TTATGAGATTCAAGGTTCACATGGAAGGAAGTATGAACGGTCATGAATTT
GAGATTGAAGGAGAAGGTGAAGGTAGACCATATGAAGGCACCCAAACAGC
TAAATTAAAAGTAACTAAAGGTGGTCCATTACCATTTAGTTGGGATATTT
TATCTCCACAATTTATGTATGGTTCACGTGCTTTCACAAAACATCCAGCA
GATATTCCAGATTATTATAAACAATCATTTCCAGAAGGTTTTAAATGGGA
ACGTGTCATGAACTTTGAAGATGGTGGAGCAGTTACAGTCACACAAGATA
CCTCATTAGAAGATGGTACATTAATATATAAAGTTAAATTACGTGGTACT
AATTTTCCACCAGACGGTCCAGTAATGCAAAAAAAAACAATGGGCTGGGA
AGCTAGTACAGAACGTTTATATCCTGAAGATGGTGTCCTTAAAGGCGATA
TAAAAATGGCCTTGAGATTAAAGGATGGTGGTAGGTATTTAGCAGATTTC
AAAACCACTTATAAAGCAAAAAAACCAGTTCAAATGCCAGGTGCATATAA
TGTTGATAGAAAACTTGATATTACCAGTCATAATGAAGATTACACAGTTG
TCGAACAATACGAACGTTCTGAAGGTCGTCATAGCACTGGTGGTATGGAT
GAATTATACAAAGGTGGTTCAGGAGGTAGTAGATCTTCAACTAGTTAAAT
AAATAAATTATTTAATAAATAATAAAAAAACAAATTGTTGTAATAATCTA
ATATTTTCTTTTTTTTTTAATTTTTTTTTTTTAAATCTTAATAATTATTA
AGTTATTTTAATTTTTTTTTTTTTTTTTTTTTTTTTTTTTTTTTTTTTTC

TATCAAAAAAATCAAATATATTTAAAAAATTTATTATTTACAGATACATT
TTGAATGGTGAAGATAAATATATGCATTAGATGTAAAACAGCCAAAGAGT
ATGAAAATCAAAAAGATAAAGCTTAGTGCTCGTCCAGCCGGCAGAGGCGG
TTTGCGTATTGGGCGCTCTTCCGCTTCCTCGCTCACTGACTCGCTGCGCT
CGGTCGTTCGGCTGCGGCGAGCGGTATCAGCTCACTCAAAGGCGGTAATA
CGGTTATCCACAGAATCAGGGGATAACGCAGGAAAGAACATGTGAGCAAA
AGGCCAGCAAAAGGCCAGGAACCGTAAAAAGGCCGCGTTGCTGGCGTTTT
TCCATAGGCTCCGCCCCCCTGACGAGCATCACAAAAATCGACGCTCAAGT
CAGAGGTGGCGAAACCCGACAGGACTATAAAGATACCAGGCGTTTCCCCC
TGGAAGCTCCCTCGTGCGCTCTCCTGTTCCGACCCTGCCGCTTACCGGAT
ACCTGTCCGCCTTTCTCCCTTCGGGAAGCGTGGCGCTTTCTCATAGCTCA
CGCTGTAGGTATCTCAGTTCGGTGTAGGTCGTTCGCTCCAAGCTGGGCTG
TGTGCACGAACCCCCCGTTCAGCCCGACCGCTGCGCCTTATCCGGTAACT
ATCGTCTTGAGTCCAACCCGGTAAGACACGACTTATCGCCACTGGCAGCA
GCCACTGGTAACAGGATTAGCAGAGCGAGGTATGTAGGCGGTGCTACAGA
GTTCTTGAAGTGGTGGCCTAACTACGGCTACACTAGAAGGACAGTATTTG
GTATCTGCGCTCTGCTGAAGCCAGTTACCTTCGGAAAAAGAGTTGGTAGC
TCTTGATCCGGCAAACAAACCACCGCTGGTAGCGGTGGTTTTTTTGTTTG
CAAGCAGCAGATTACGCGCAGAAAAAAAGGATCTCAAGAAGATCCTTTGA
TCTTTTCTACGGGGTCTGACGCTCAGTGGAACGAAAACTCACGTTAAGGG
ATTTTGGTCATGAGATTATCAAAAAGGATCTTCACCTAGATCCTTTTAAA
TTAAAAATGAAGTTTTAAATCAATCTAAAGTATATATGAGTAAACTTGGT
CTGACAGTTACCAATGCTTAATCAGTGAGGCACCTATCTCAGCGATCTGT
CTATTTCGTTCATCCATAGTTGCCTGACTCCCCGTCGTGTAGATAACTAC
GATACGGGAGGGCTTACCATCTGGCCCCAGTGCTGCAATGATACCGCGAG
ACCCACGCTCACCGGCTCCAGATTTATCAGCAATAAACCAGCCAGCCGGA
AGGGCCGAGCGCAGAAGTGGTCCTGCAACTTTATCCGCCTCCATCCAGTC
TATTAATTGTTGCCGGGAAGCTAGAGTAAGTAGTTCGCCAGTTAATAGTT
TGCGCAACGTTGTTGCCATTGCTACAGGCATCGTGGTGTCACGCTCGTCG
TTTGGTATGGCTTCATTCAGCTCCGGTTCCCAACGATCAAGGCGAGTTAC
ATGATCCCCCATGTTGTGCAAAAAAGCGGTTAGCTCCTTCGGTCCTCCGA
TCGTTGTCAGAAGTAAGTTGGCCGCAGTGTTATCACTCATGGTTATGGCA
GCACTGCATAATTCTCTTACTGTCATGCCATCCGTAAGATGCTTTTCTGT
GACTGGTGAGTACTCAACCAAGTCATTCTGAGAATAGTGTATGCGGCGAC
CGAGTTGCTCTTGCCCGGCGTCAATACGGGATAATACCGCGCCACATAGC
AGAACTTTAAAAGTGCTCATCATTGGAAAACGTTCTTCGGGGCGAAAACT
CTCAAGGATCTTACCGCTGTTGAGATCCAGTTCGATGTAACCCACTCGTG
CACCCAACTGATCTTCAGCATCTTTTACTTTCACCAGCGTTTCTGGGTGA
GCAAAAACAGGAAGGCAAAATGCCGCAAAAAAGGGAATAAGGGCGACACG
GAAATGTTGAATACTCATACTCTTCCTTTTTCAATATTATTGAAGCATTT
ATCAGGGTTATTGTCTCATGAGCGGATACATATTTGAATGTATTTAGAAA
AATAAACAAATAGGGGTTCCGCGCACATTTCCCCGAAAAGTGCCACCTGA
CGCGCCCTGTAGCGGGATCCATTTTATTTAATATACTAAATAATAAAAAA
GTTAAAAAATGATCATTGGATAAATTTTTTATAATTATAAATAAAGATAA
TAATTTTTTTTTTAACAAAACTAAAAATAAAAATAATAAAATAATTGTTA
AAATAGGTTTTTTTTTTTTTTTTTTTTTTTTAATAAATGGTATTTATTAA
TTTATTTGTTGTGTGTGTTTTTTTTTTTATAATATTTTTTTTTTTAGCAT
TGAATTAAGAAGAAATCAAATTGATGCGGCCGCTTAGTTAGCCTCCCCCA
TCTCCCGATCCGGACGAGTGCTGGGGCGTCGGTTTCCACTATCGGCGAGT
ACTTCTACACAGCCATCGGTCCAGACGGCCGCGCTTCTGCGGGCGATTTG

TGTACGCCCGACAGTCCCGGCTCCGGATCGGACGATTGCGTCGCATCGAC
CCTGCGCCCAAGCTGCATCATCGAAATTGCCGTCAACCAAGCTCTGATAG
AGTTGGTCAAGACCAATGCGGAGCATATACGCCCGGAGCCGCGGCGATCC
TGCAAGCTCCGGATGCCTCCGCTCGAAGTAGCGCGTCTGCTGCTCCATAC
AAGCCAACCACGGCCTCCAGAAGAAGATGTTGGCGACCTCGTATTGGGAA
TCCCCGAACATCGCCTCGCTCCAGTCAATGACCGCTGTTATGCGGCCATT
GTCCGTCAGGACATTGTTGGAGCCGAAATCCGCGTGCACGAGGTGCCGGA
CTTCGGGGCAGTCCTCGGCCCAAAGCATCAGCTCATCGAGAGCCTGCGCG
ACGGACGCACTGACGGTGTCGTCCATCACAGTTTGCCAGTGATACACATG
GGGATCAGCAATCGCGCATATGAAATCACGCCATGTAGTGTATTGACCGA
TTCCTTGCGGTCCGAATGGGCCGAACCCGCTCGTCTGGCTAAGATCGGCC
GCAGCGATCGCATCCATGGCCTCCGCGACCGGCTGCAGAACAGCGGGCAG
TTCGGTTTCAGGCAGGTCTTGCAACGTGACACCCTGTGCACGGCGGGAGA
TGCAATAGGTCAGGCTCTCGCTAAATTCCCCAATGTCAAGCACTTCCGGA
ATCGGGAGCGCGGCCGATGCAAAGTGCCGATAAACATAACGATCTTTGTA
GAAACCATCGGCGCAGCTATTTACCCGCAGGACATATCCACGCCCTCCTA
CATCGAAGCTGAAAGCACGAGATTCTTCGCCCTCCGAGAGCTGCATCAGG
TCGGAGACGCTGTCGAACTTTTCGATCAGAAACTTCTCGACAGACGTCGC
GGTGAGTTCAGGCTTTTTCATTAGGTCTTGTTGAGAAATGTTAAATTGAT
CCATTTTTTGCTAGCTTTATTGTTGTATTATATTTAAATATTTGTTTATT
AAAACTATAGAAAAAAAAAAAAAAATGATTTAATTTTTCATTTTATACCCC
TTGAAAAAAAAAAATAAAAAAAATTAAAAACCCAATAAAATCCCATTTTT
CAAAAAAAATAATTAAAAAAATTCAAAAAAAAATAAAATTTTAAAATGGC
TTGTTTTTTTTGATGAAATTTTTAATGGGAGTGGGTTACCCAATTATTTT
TTTGAAAAATATGAAAAAATCTTTGAAACCACCATAACCAGCAATTCAAA
TAAAATTCAAAAAAAAAAAAAAAAAAAACATTTTATGACTTAATTATAATT
ATTTTTTTTGGTGTTTGATTAACATATCTTTAAATTAATTACTATTATTA
TTATTTTTTTTTTTTTATTATTATTTTATTTATTTATTTTTTTTTTTTTT
ATTTTTTTTTTTATTTTTTTTTTGAATCCCAATTTTTTAAATTTCTGAAC
ACAACAAAGAAA

pPI467 – mScarlet (C)

CTCGAGACTAGAGCTAGATAAAAAAAATTTTTATTTATTTTTATTTATTT
TGAATTAAATAGATTACAAATTAATTAATCCCATCAAATCTTTAAAAAAA
AATGGTTTAAAAAAACTTGGGTTGGTTAATTATTATTTGAAAATTTTAAA
ACCCAAATTAAAAAAAAAAAAATGGGATTCAAAAATTTTTTTTTTTTTTT
TTTTTTTTTTTTTTTTTTTTTTTCAGATTGCATAAAAAGATTTTTTTTTT
TTTTTTTTCTTATTTCTTAAAACAAATAAATTAAATTAAATAAAAAATAA
AAATCAGATCTTCAACTAGTGGTGGTTCAGGAGGTAGTGTTTCAAAAGGT
GAAGCCGTTATTAAAGAATTTATGAGATTCAAGGTTCACATGGAAGGAAG
TATGAACGGTCATGAATTTGAGATTGAAGGAGAAGGTGAAGGTAGACCAT
ATGAAGGCACCCAAACAGCTAAATTAAAAGTAACTAAAGGTGGTCCATTA
CCATTTAGTTGGGATATTTTATCTCCACAATTTATGTATGGTTCACGTGC
TTTCACAAAACATCCAGCAGATATTCCAGATTATTATAAACAATCATTTC
CAGAAGGTTTTAAATGGGAACGTGTCATGAACTTTGAAGATGGTGGAGCA
GTTACAGTCACACAAGATACCTCATTAGAAGATGGTACATTAATATATAA
AGTTAAATTACGTGGTACTAATTTTCCACCAGACGGTCCAGTAATGCAAA
AAAAAACAATGGGCTGGGAAGCTAGTACAGAACGTTTATATCCTGAAGAT
GGTGTCCTTAAAGGCGATATAAAAATGGCCTTGAGATTAAAGGATGGTGG

TAGGTATTTAGCAGATTTCAAAACCACTTATAAAGCAAAAAAACCAGTTC
AAATGCCAGGTGCATATAATGTTGATAGAAAACTTGATATTACCAGTCAT
AATGAAGATTACACAGTTGTCGAACAATACGAACGTTCTGAAGGTCGTCA
TAGCACTGGTGGTATGGATGAATTATACAAATAAGCTAGTTAAATAAATA
AATTATTTAATAAATAATAAAAAAACAAATTGTTGTAATAATCTAATATT
TTCTTTTTTTTTTAATTTTTTTTTTTTAAATCTTAATAATTATTAAGTTA
TTTTAATTTTTTTTTTTTTTTTTTTTTTTTTTTTTTTTTTTTTTCTATCA
AAAAAATCAAATATATTTAAAAAATTTATTATTTACAGATACATTTTGAA
TGGTGAAGATAAATATATGCATTAGATGTAAAACAGCCAAAGAGTATGAA
AATCAAAAAGATAAAGCTTAGTGCTCGTCCAGCCGGCAGAGGCGGTTTGC
GTATTGGGCGCTCTTCCGCTTCCTCGCTCACTGACTCGCTGCGCTCGGTC
GTTCGGCTGCGGCGAGCGGTATCAGCTCACTCAAAGGCGGTAATACGGTT
ATCCACAGAATCAGGGGATAACGCAGGAAAGAACATGTGAGCAAAAGGCC
AGCAAAAGGCCAGGAACCGTAAAAAGGCCGCGTTGCTGGCGTTTTTCCAT
AGGCTCCGCCCCCCTGACGAGCATCACAAAAATCGACGCTCAAGTCAGAG
GTGGCGAAACCCGACAGGACTATAAAGATACCAGGCGTTTCCCCCTGGAA
GCTCCCTCGTGCGCTCTCCTGTTCCGACCCTGCCGCTTACCGGATACCTG
TCCGCCTTTCTCCCTTCGGGAAGCGTGGCGCTTTCTCATAGCTCACGCTG
TAGGTATCTCAGTTCGGTGTAGGTCGTTCGCTCCAAGCTGGGCTGTGTGC
ACGAACCCCCCGTTCAGCCCGACCGCTGCGCCTTATCCGGTAACTATCGT
CTTGAGTCCAACCCGGTAAGACACGACTTATCGCCACTGGCAGCAGCCAC
TGGTAACAGGATTAGCAGAGCGAGGTATGTAGGCGGTGCTACAGAGTTCT
TGAAGTGGTGGCCTAACTACGGCTACACTAGAAGGACAGTATTTGGTATC
TGCGCTCTGCTGAAGCCAGTTACCTTCGGAAAAAGAGTTGGTAGCTCTTG
ATCCGGCAAACAAACCACCGCTGGTAGCGGTGGTTTTTTTGTTTGCAAGC
AGCAGATTACGCGCAGAAAAAAAGGATCTCAAGAAGATCCTTTGATCTTT
TCTACGGGGTCTGACGCTCAGTGGAACGAAAACTCACGTTAAGGGATTTT
GGTCATGAGATTATCAAAAAGGATCTTCACCTAGATCCTTTTAAATTAAA
AATGAAGTTTTAAATCAATCTAAAGTATATATGAGTAAACTTGGTCTGAC
AGTTACCAATGCTTAATCAGTGAGGCACCTATCTCAGCGATCTGTCTATT
TCGTTCATCCATAGTTGCCTGACTCCCCGTCGTGTAGATAACTACGATAC
GGGAGGGCTTACCATCTGGCCCCAGTGCTGCAATGATACCGCGAGACCCA
CGCTCACCGGCTCCAGATTTATCAGCAATAAACCAGCCAGCCGGAAGGGC
CGAGCGCAGAAGTGGTCCTGCAACTTTATCCGCCTCCATCCAGTCTATTA
ATTGTTGCCGGGAAGCTAGAGTAAGTAGTTCGCCAGTTAATAGTTTGCGC
AACGTTGTTGCCATTGCTACAGGCATCGTGGTGTCACGCTCGTCGTTTGG
TATGGCTTCATTCAGCTCCGGTTCCCAACGATCAAGGCGAGTTACATGAT
CCCCCATGTTGTGCAAAAAAGCGGTTAGCTCCTTCGGTCCTCCGATCGTT
GTCAGAAGTAAGTTGGCCGCAGTGTTATCACTCATGGTTATGGCAGCACT
GCATAATTCTCTTACTGTCATGCCATCCGTAAGATGCTTTTCTGTGACTG
GTGAGTACTCAACCAAGTCATTCTGAGAATAGTGTATGCGGCGACCGAGT
TGCTCTTGCCCGGCGTCAATACGGGATAATACCGCGCCACATAGCAGAAC
TTTAAAAGTGCTCATCATTGGAAAACGTTCTTCGGGGCGAAAACTCTCAA
GGATCTTACCGCTGTTGAGATCCAGTTCGATGTAACCCACTCGTGCACCC
AACTGATCTTCAGCATCTTTTACTTTCACCAGCGTTTCTGGGTGAGCAAA
AACAGGAAGGCAAAATGCCGCAAAAAAGGGAATAAGGGCGACACGGAAAT
GTTGAATACTCATACTCTTCCTTTTTCAATATTATTGAAGCATTTATCAG
GGTTATTGTCTCATGAGCGGATACATATTTGAATGTATTTAGAAAAATAA
ACAAATAGGGGTTCCGCGCACATTTCCCCGAAAAGTGCCACCTGACGCGC
CCTGTAGCGGGATCCATTTTATTTAATATACTAAATAATAAAAAAGTTAA

AAAATGATCATTGGATAAATTTTTTATAATTATAAATAAAGATAATAATT
TTTTTTTTAACAAAACTAAAAATAAAAATAATAAAATAATTGTTAAAATA
GGTTTTTTTTTTTTTTTTTTTTTTTTAATAAATGGTATTTATTAATTTAT
TTGTTGTGTGTGTTTTTTTTTTTTATAATATTTTTTTTTTTTAGCATTGAAT
TAAGAAGAAATCAAATTGATGCGGCCGCTTAGTTAGCCTCCCCCATCTCC
CGATCCGGACGAGTGCTGGGGCGTCGGTTTCCACTATCGGCGAGTACTTC
TACACAGCCATCGGTCCAGACGGCCGCGCTTCTGCGGGCGATTTGTGTAC
GCCCGACAGTCCCGGCTCCGGATCGGACGATTGCGTCGCATCGACCCTGC
GCCCAAGCTGCATCATCGAAATTGCCGTCAACCAAGCTCTGATAGAGTTG
GTCAAGACCAATGCGGAGCATATACGCCCGGAGCCGCGGCGATCCTGCAA
GCTCCGGATGCCTCCGCTCGAAGTAGCGCGTCTGCTGCTCCATACAAGCC
AACCACGGCCTCCAGAAGAAGATGTTGGCGACCTCGTATTGGGAATCCCC
GAACATCGCCTCGCTCCAGTCAATGACCGCTGTTATGCGGCCATTGTCCG
TCAGGACATTGTTGGAGCCGAAATCCGCGTGCACGAGGTGCCGGACTTCG
GGGCAGTCCTCGGCCCAAAGCATCAGCTCATCGAGAGCCTGCGCGACGGA
CGCACTGACGGTGTCGTCCATCACAGTTTGCCAGTGATACACATGGGGAT
CAGCAATCGCGCATATGAAATCACGCCATGTAGTGTATTGACCGATTCCT
TGCGGTCCGAATGGGCCGAACCCGCTCGTCTGGCTAAGATCGGCCGCAGC
GATCGCATCCATGGCCTCCGCGACCGGCTGCAGAACAGCGGGCAGTTCGG
TTTCAGGCAGGTCTTGCAACGTGACACCCTGTGCACGGCGGGAGATGCAA
TAGGTCAGGCTCTCGCTAAATTCCCCAATGTCAAGCACTTCCGGAATCGG
GAGCGCGGCCGATGCAAAGTGCCGATAAACATAACGATCTTTGTAGAAAC
CATCGGCGCAGCTATTTACCCGCAGGACATATCCACGCCCTCCTACATCG
AAGCTGAAAGCACGAGATTCTTCGCCCTCCGAGAGCTGCATCAGGTCGGA
GACGCTGTCGAACTTTTCGATCAGAAACTTCTCGACAGACGTCGCGGTGA
GTTCAGGCTTTTTCATTAGGTCTTGTTGAGAAATGTTAAATTGATCCATT
TTTTGCTAGCTTTATTGTTGTATTATATTTAAATATTTGTTTATTAAAAC
TATAGAAAAAAAAAAAAAAATGATTTAATTTTTCATTTTATACCCCTTGAA
AAAAAAAAATAAAAAAAATTAAAAACCCAATAAAATCCCATTTTTCAAAA
AAAATAATTAAAAAAAATTCAAAAAAAAATAAAATTTTAAAATGGCTTGTT
TTTTTTGATGAAATTTTTAATGGGAGTGGGTTACCCAATTATTTTTTTGA
AAAATATGAAAAAATCTTTGAAACCACCATAACCAGCAATTCAAATAAAA
TTCAAAAAAAAAAAAAAAAAAAACATTTTATGACTTAATTATAATTATTTT
TTTTGGTGTTTGATTAACATATCTTTAAATTAATTACTATTATTATTATT
TTTTTTTTTTTATTATTATTTTATTTATTTATTTTTTTTTTTTTTATTTT
TTTTTTTATTTTTTTTTTTGAATCCCAATTTTTTAAATTTCTGAACACAAC
AAAGAAA

act5 locus vectors

pDM1501 – No tag

ACTAGCGCCGGCATTACATGTCCATTTATTAGAAGGATTCAATCAAAAAT
TAACACCAGGTATATTACCAGATGGCATTAATAATTTATATTCTTTAATA
TTGGAAAAAGAAAAAAAATAAATAAACAATCAATCAGAAAAGATAATTGAA
AAAAAAAAAAAAAGAAAATGTTTAATAAGAATTAATTTGGAAGTAAAATAA
TTTTTGGATGAGTAAATATTAATAAACTCACAATTAAAATTTAGAATCCA
AAAAAATGAAAAAAAAAAAAAATTTTTAAAACAGAATGGTTGCATTCATTT
TTTTTTTTTTTTTTTTATATCTTTTGGGTATTTTTGGGTGTTCTATTTAT
CTGGATAAAAAATAAAATGGAATATAGTTTTATATATATATTTTTTTCGT

ATCAAGCTTGAATGTTTTTATCAATAATATGATTGTTGGTTGCTTTATTT
TATTTGAAAAAAAAATTAATTTTTAAAATATCTTTTTTTTCATTTTATTT
GAAAAAAAAAAAAAAAAAAAAATGGTTTTGAAAATTTTTAAAAAAATGGTT
GGGTCACCCACAATTCTTATAAAAAAAAAGCCATTTGGATAGTTTTTTTTT
TTTTTTTTTTTATTTTTTTTATTTTTTTTTTTTTGAGATTTGGTATAAAT
ACAAAAAAAAAAATTGTTTTTAAATCATTATTAATAAAAACTTAAAATAAA
TTATATATAAAAAAAGATCTAGTACTAGTATAAAATAAATAAATTGTGGA
TCTGATATCATAACTTCGTATAATGTATGCTATACGAAGTTATACTACTC
TCGAGATTTTATTTAATATACTAAATAATAAAAAAGTTAAAAAATGATCA
TTGGATAAATTTTTTATAATTATAAATAAAGATAATAATTTTTTTTTTAA
CAAAACTAAAAATAAAAATAATAAAATAATTGTTAAAATAGGTTTTTTTT
TTTTTTTTTTTTTTTTAATAAATGGTATTTATTAATTTATTTGTTGTGTG
TGTTTTTTTTTTTATAATATTTTTTTTTTTTAGCATTGAATTAAGAAGAAA
TCAAATTGATGCGGCCGCTTAGTTAGCCTCCCCCATCTCCCGATCCGGAC
GAGTGCTGGGGCGTCGGTTTCCACTATCGGCGAGTACTTCTACACAGCCA
TCGGTCCAGACGGCCGCGCTTCTGCGGGCGATTTGTGTACGCCCGACAGT
CCCGGCTCCGGATCGGACGATTGCGTCGCATCGACCCTGCGCCCAAGCTG
CATCATCGAAATTGCCGTCAACCAAGCTCTGATAGAGTTGGTCAAGACCA
ATGCGGAGCATATACGCCCGGAGCCGCGGCGATCCTGCAAGCTCCGGATG
CCTCCGCTCGAAGTAGCGCGTCTGCTGCTCCATACAAGCCAACCACGGCC
TCCAGAAGAAGATGTTGGCGACCTCGTATTGGGAATCCCCGAACATCGCC
TCGCTCCAGTCAATGACCGCTGTTATGCGGCCATTGTCCGTCAGGACATT
GTTGGAGCCGAAATCCGCGTGCACGAGGTGCCGGACTTCGGGGCAGTCCT
CGGCCCAAAGCATCAGCTCATCGAGAGCCTGCGCGACGGACGCACTGACG
GTGTCGTCCATCACAGTTTGCCAGTGATACACATGGGGATCAGCAATCGC
GCATATGAAATCACGCCATGTAGTGTATTGACCGATTCCTTGCGGTCCGA
ATGGGCCGAACCCGCTCGTCTGGCTAAGATCGGCCGCAGCGATCGCATCC
ATGGCCTCCGCGACCGGCTGCAGAACAGCGGGCAGTTCGGTTTCAGGCAG
GTCTTGCAACGTGACACCCTGTGCACGGCGGGAGATGCAATAGGTCAGGC
TCTCGCTGAACTCCCCAATGTCAAGCACTTCCGGAATCGGGAGCGCGGCC
GATGCAAAGTGCCGATAAACATAACGATCTTTGTAGAAACCATCGGCGCA
GCTATTTACCCGCAGGACATATCCACGCCCTCCTACATCGAAGCTGAAAG
CACGAGATTCTTCGCCCTCCGAGAGCTGCATCAGGTCGGAGACGCTGTCG
AACTTTTCGATCAGAAACTTCTCGACAGACGTCGCGGTGAGTTCAGGCTT
TTTCATTAGGTCTTGTTGAGAAATGTTAAATTGATCCATTTTTTGCTAGC
TGTGAAATTAGTTTAAAATACAAATAAAGAGTTATAATAATATACAGTTG
AATAAAAAAAAAAAAAAATGAATTGGAAAATTTATTTTTATATGAAGAAAA
AAAAATTTTGAAAAAAAAAAAAAAAAATTAAAAAAAAAAAAAAAAAAAAAA
AAAATTAAAAATAATTCAACTGTGGGTGACCCAAATTTTATTTAAAAAAA
AAAAAAAAATGGATCACTTTTGGGTGGTTGGAAAAAAAAAAAAAAAAAAA
TTGAAAATATAATGTTAGTCATATGATTAATCATTAATTAATTCTTTTTT
TTTTTTTTTTTATTTTTTATTTTTTTTTTTTTCAATATTCCAATTATGACA
CACACTAACATCACAAAATACAAAATTTTGGGATCCACTACTATAACTTC
GTATAATGTATGCTATACGAAGTTATTTAATTAATTAAGATATCAAGCTT
GTCCACAGAAAATGTTTCTAAACAAAAAAAAAAAAACCGAGTGATGAAAGC
GCTTCTCACAAAATTATTTATGTAAAATATTTAATAAAATACATTATTTA
AATCTTTTTTATTTTTTTTAGTTGTTGATCTTTATCCGACTTTAAAAATA
AAAAAATTGTAAAAAAAAAAAAAAGTTTATTTGTTTAATTTTATTGTTGTT
TTTAATTTTTTACAACCATTAATTTATAAAACCAACACCTAACTATAATG
AAATACAATTTGTATCTTGGAAAAAAAAAAAATTAATAATATAAATAAAAT

GTAAACAAGTATCCGGTGGAAGGAACTAACAGTTGCCTTATCCAGTTCAA
AAAAAAAAAAAAAAAAAAGAAATTAAATTACCATTTTATATTTAACTAAAA
TAAATTAAAAGGTTTATTTTAATAATAGATTTATTATTTTTTAAAAATCA
ATAATTAATGTTGGATAAGATGAACGAATAAAAGATAGAGTTTCAGGTCT
TTTAAATGATGAGGATTTATGAATTCTTAAAAGTTTTGGGCAAGAGAAAA
TGATTTTAACGATTGAACCATCACTAATTGCAAAATCTCTTGAACTAAGA
GTTTTTAAGTTTGAAGCAAGTGGAGAATTACAAAGAGCAATCATAGAGTT
ATCAGTTAAATCATAACAACAAGATAAAGATAAAGAACTAATATGATGTA
AATTTTCAATCATACCTCTAAAAGAAACATCATTCATACTTTTTGGATAA
ATTAAATCTAATGAATCTAAATTTGGAAATAGTAAACCAATATTTGGCCG
GCAGAGGCGGTTTGCGTATTGGGCGCTCTTCCGCTTCCTCGCTCACTGAC
TCGCTGCGCTCGGTCGTTCGGCTGCGGCGAGCGGTATCAGCTCACTCAAA
GGCGGTAATACGGTTATCCACAGAATCAGGGGATAACGCAGGAAAGAACA
TGTGAGCAAAAGGCCAGCAAAAGGCCAGGAACCGTAAAAAGGCCGCGTTG
CTGGCGTTTTTCCATAGGCTCCGCCCCCCTGACGAGCATCACAAAAATCG
ACGCTCAAGTCAGAGGTGGCGAAACCCGACAGGACTATAAAGATACCAGG
CGTTTCCCCCTGGAAGCTCCCTCGTGCGCTCTCCTGTTCCGACCCTGCCG
CTTACCGGATACCTGTCCGCCTTTCTCCCTTCGGGAAGCGTGGCGCTTTC
TCATAGCTCACGCTGTAGGTATCTCAGTTCGGTGTAGGTCGTTCGCTCCA
AGCTGGGCTGTGTGCACGAACCCCCCGTTCAGCCCGACCGCTGCGCCTTA
TCCGGTAACTATCGTCTTGAGTCCAACCCGGTAAGACACGACTTATCGCC
ACTGGCAGCAGCCACTGGTAACAGGATTAGCAGAGCGAGGTATGTAGGCG
GTGCTACAGAGTTCTTGAAGTGGTGGCCTAACTACGGCTACACTAGAAGG
ACAGTATTTGGTATCTGCGCTCTGCTGAAGCCAGTTACCTTCGGAAAAAG
AGTTGGTAGCTCTTGATCCGGCAAACAAACCACCGCTGGTAGCGGTGGTT
TTTTTGTTTGCAAGCAGCAGATTACGCGCAGAAAAAAAGGATCTCAAGAA
GATCCTTTGATCTTTTCTACGGGGTCTGACGCTCAGTGGAACGAAAACTC
ACGTTAAGGGATTTTGGTCATGAGATTATCAAAAAGGATCTTCACCTAGA
TCCTTTTAAATTAAAAATGAAGTTTTAAATCAATCTAAAGTATATATGAG
TAAACTTGGTCTGACAGTTACCAATGCTTAATCAGTGAGGCACCTATCTC
AGCGATCTGTCTATTTCGTTCATCCATAGTTGCCTGACTCCCCGTCGTGT
AGATAACTACGATACGGGAGGGCTTACCATCTGGCCCCAGTGCTGCAATG
ATACCGCGAGACCCACGCTCACCGGCTCCAGATTTATCAGCAATAAACCA
GCCAGCCGGAAGGGCCGAGCGCAGAAGTGGTCCTGCAACTTTATCCGCCT
CCATCCAGTCTATTAATTGTTGCCGGGAAGCTAGAGTAAGTAGTTCGCCA
GTTAATAGTTTGCGCAACGTTGTTGCCATTGCTACAGGCATCGTGGTGTC
ACGCTCGTCGTTTGGTATGGCTTCATTCAGCTCCGGTTCCCAACGATCAA
GGCGAGTTACATGATCCCCCATGTTGTGCAAAAAAGCGGTTAGCTCCTTC
GGTCCTCCGATCGTTGTCAGAAGTAAGTTGGCCGCAGTGTTATCACTCAT
GGTTATGGCAGCACTGCATAATTCTCTTACTGTCATGCCATCCGTAAGAT
GCTTTTCTGTGACTGGTGAGTACTCAACCAAGTCATTCTGAGAATAGTGT
ATGCGGCGACCGAGTTGCTCTTGCCCGGCGTCAATACGGGATAATACCGC
GCCACATAGCAGAACTTTAAAAGTGCTCATCATTGGAAAACGTTCTTCGG
GGCGAAAACTCTCAAGGATCTTACCGCTGTTGAGATCCAGTTCGATGTAA
CCCACTCGTGCACCCAACTGATCTTCAGCATCTTTTACTTTCACCAGCGT
TTCTGGGTGAGCAAAAACAGGAAGGCAAAATGCCGCAAAAAAGGGAATAA
GGGCGACACGGAAATGTTGAATACTCATACTCTTCCTTTTTCAATATTAT
TGAAGCATTTATCAGGGTTATTGTCTCATGAGCGGATACATATTTGAATG
TATTTAGAAAAATAAACAAATAGGGGTTCCGCGCACATTTCCCCGAAAAG
TGCCACCTGACGCGCCCTGTAGCGGGATCA

pDM1513 – GFP (N)

ACTAGCGCCGGCATTACATGTCCATTTATTAGAAGGATTCAATCAAAAAT
TAACACCAGGTATATTACCAGATGGCATTAATAATTTATATTCTTTAATA
TTGGAAAAAGAAAAAAATAAATAAACAATCAATCAGAAAAGATAATTGAA
AAAAAAAAAAAAAGAAAATGTTTAATAAGAATTAATTTGGAAGTAAAATAA
TTTTTGGATGAGTAAATATTAATAAACTCACAATTAAAATTTAGAATCCA
AAAAAATGAAAAAAAAAAAAAATTTTTAAAACAGAATGGTTGCATTCATTT
TTTTTTTTTTTTTTTTATATCTTTTGGGTATTTTTGGGTGTTCTATTTAT
CTGGATAAAAAATAAAATGGAATATAGTTTTATATATATATTTTTTTCGT
ATCAAGCTTGAATGTTTTTATCAATAATATGATTGTTGGTTGCTTTATTT
TATTTGAAAAAAAAATTAATTTTTAAAATATCTTTTTTTTCATTTTATTT
GAAAAAAAAAAAAAAAAAAAAATGGTTTTGAAAATTTTTAAAAAAATGGTT
GGGTCACCCACAATTCTTATAAAAAAAAGCCATTTGGATAGTTTTTTTTT
TTTTTTTTTTATTTTTTTTATTTTTTTTTTTTTGAGATTTGGTATAAAT
ACAAAAAAAAAAATTGTTTTTAAATCATTATTAATAAAAACTTAAAATAAA
TTATATATAAAAAAAGATCCAAAAAATGTCAAAAGGTGAAGAATTATTTA
CAGGTGTTGTTCCAATTTTAGTTGAATTAGATGGTGATGTTAATGGTCAT
AAATTTTCAGTTTCAGGTGAAGGTGAAGGTGATGCAACATATGGTAAATT
AACATTAAAATTTATTTGTACAACAGGTAAATTACCAGTTCCATGGCCAA
CATTAGTTACAACATTTACATATGGTGTTCAATGTTTTTCAAGATATCCA
GATCATATGAAACAACATGATTTTTTTAAATCAGCAATGCCAGAAGGTTA
TGTTCAAGAAAGAACAATTTTTTTTAAAGATGATGGTAATTATAAAACAA
GAGCAGAAGTTAAATTTGAAGGTGATACATTAGTTAATAGAATTGAATTA
AAAGGTATTGATTTTAAAGAAGATGGTAATATTTTAGGTCATAAATTAGA
ATATAATTATAATTCACATAATGTTTATATTATGGCAGATAAACAAAAAA
ATGGTATTAAAGTTAATTTTAAAATTAGACATAATATTGAAGATGGTTCA
GTTCAATTAGCAGATCATTATCAACAAAATACACCAATTGGTGATGGTCC
AGTTTTATTACCAGATAATCATTATTTATCAACACAATCAGCATTATCAA
AAGATCCAAATGAAAAAAGAGATCATATGGTTTTATTAGAATTTGTTACA
GCAGCAGGTATTACACATGGTATGGATGAATTATATAAAGGTGGTTCAGG
AGGTAGTAGATCTTCAACTAGTATAAAATAAATAAATTGTGGATCTGATA
TCATAACTTCGTATAATGTATGCTATACGAAGTTATACTACTCTCGAGAT
TTTATTTAATATACTAAATAATAAAAAAGTTAAAAAATGATCATTGGATA
AATTTTTTATAATTATAAATAAAGATAATAATTTTTTTTTTAACAAAACT
AAAAATAAAAATAATAAAATAATTGTTAAAATAGGTTTTTTTTTTTTTTT
TTTTTTTTTAATAAATGGTATTTATTAATTTATTTGTTGTGTGTGTTTTT
TTTTTTATAATATTTTTTTTTTTTAGCATTGAATTAAGAAGAAATCAAATT
GATGCGGCCGCTTAGTTAGCCTCCCCCATCTCCCGATCCGGACGAGTGCT
GGGGCGTCGGTTTCCACTATCGGCGAGTACTTCTACACAGCCATCGGTCC
AGACGGCCGCGCTTCTGCGGGCGATTTGTGTACGCCCGACAGTCCCGGCT
CCGGATCGGACGATTGCGTCGCATCGACCCTGCGCCCAAGCTGCATCATC
GAAATTGCCGTCAACCAAGCTCTGATAGAGTTGGTCAAGACCAATGCGGA
GCATATACGCCCGGAGCCGCGGCGATCCTGCAAGCTCCGGATGCCTCCGC
TCGAAGTAGCGCGTCTGCTGCTCCATACAAGCCAACCACGGCCTCCAGAA
GAAGATGTTGGCGACCTCGTATTGGGAATCCCCGAACATCGCCTCGCTCC
AGTCAATGACCGCTGTTATGCGGCCATTGTCCGTCAGGACATTGTTGGAG
CCGAAATCCGCGTGCACGAGGTGCCGGACTTCGGGGCAGTCCTCGGCCCA
AAGCATCAGCTCATCGAGAGCCTGCGCGACGGACGCACTGACGGTGTCGT

CCATCACAGTTTGCCAGTGATACACATGGGGATCAGCAATCGCGCATATG
AAATCACGCCATGTAGTGTATTGACCGATTCCTTGCGGTCCGAATGGGCC
GAACCCGCTCGTCTGGCTAAGATCGGCCGCAGCGATCGCATCCATGGCCT
CCGCGACCGGCTGCAGAACAGCGGGCAGTTCGGTTTCAGGCAGGTCTTGC
AACGTGACACCCTGTGCACGGCGGGAGATGCAATAGGTCAGGCTCTCGCT
GAACTCCCCAATGTCAAGCACTTCCGGAATCGGGAGCGCGGCCGATGCAA
AGTGCCGATAAACATAACGATCTTTGTAGAAACCATCGGCGCAGCTATTT
ACCCGCAGGACATATCCACGCCCTCCTACATCGAAGCTGAAAGCACGAGA
TTCTTCGCCCTCCGAGAGCTGCATCAGGTCGGAGACGCTGTCGAACTTTT
CGATCAGAAACTTCTCGACAGACGTCGCGGTGAGTTCAGGCTTTTTCATT
AGGTCTTGTTGAGAAATGTTAAATTGATCCATTTTTTGCTAGCTGTGAAA
TTAGTTTAAAATACAAATAAAGAGTTATAATAATATACAGTTGAATAAAA
AAAAAAAAAATGAATTGGAAAATTTATTTTTATATGAAGAAAAAAAAATT
TTGAAAAAAAAAAAAAAATTAAAAAAAAAAAAAAAAAAAAAAAAAAATTA
AAAATAATTCAACTGTGGGTGACCCAAATTTTATTTAAAAAAAAAAAAAA
AATGGATCACTTTTGGGTGGTTGGAAAAAAAAAAAAAAAAAAATTGAAAA
TATAATGTTAGTCATATGATTAATCATTAATTAATTCTTTTTTTTTTTTT
TTTTATTTTTTATTTTTTTTTTTTCAATATTCCAATTATGACACACACTA
ACATCACAAAATACAAAATTTTGGGATCCACTACTATAACTTCGTATAAT
GTATGCTATACGAAGTTATTTAATTAATTAAGATATCAAGCTTGTCCACA
GAAAATGTTTCTAAACAAAAAAAAAAAACCGAGTGATGAAAGCGCTTCTC
ACAAAATTATTTATGTAAAATATTTAATAAAATACATTATTTAAATCTTT
TTTATTTTTTTTAGTTGTTGATCTTTATCCGACTTTAAAAATAAAAAAAT
TGTAAAAAAAAAAAAAGTTTATTTGTTTAATTTTATTGTTGTTTTTAATT
TTTTACAACCATTAATTTATAAAACCAACACCTAACTATAATGAAATACA
ATTTGTATCTTGGAAAAAAAAAAATTAATAATATAAATAAAATGTAAACA
AGTATCCGGTGGAAGGAACTAACAGTTGCCTTATCCAGTTCAAAAAAAAA
AAAAAAAAAAGAAATTAAATTACCATTTTATATTTAACTAAAATAAATTA
AAAGGTTTATTTTAATAATAGATTTATTATTTTTTAAAAATCAATAATTA
ATGTTGGATAAGATGAACGAATAAAAGATAGAGTTTCAGGTCTTTTAAAT
GATGAGGATTTATGAATTCTTAAAAGTTTTGGGCAAGAGAAAATGATTTT
AACGATTGAACCATCACTAATTGCAAAATCTCTTGAACTAAGAGTTTTTA
AGTTTGAAGCAAGTGGAGAATTACAAAGAGCAATCATAGAGTTATCAGTT
AAATCATAACAACAAGATAAAGATAAAGAACTAATATGATGTAAATTTTC
AATCATACCTCTAAAAGAAACATCATTCATACTTTTTGGATAAATTAAAT
CTAATGAATCTAAATTTGGAAATAGTAAACCAATATTTGGCCGGCAGAGG
CGGTTTGCGTATTGGGCGCTCTTCCGCTTCCTCGCTCACTGACTCGCTGC
GCTCGGTCGTTCGGCTGCGGCGAGCGGTATCAGCTCACTCAAAGGCGGTA
ATACGGTTATCCACAGAATCAGGGGATAACGCAGGAAAGAACATGTGAGC
AAAAGGCCAGCAAAAGGCCAGGAACCGTAAAAAGGCCGCGTTGCTGGCGT
TTTTCCATAGGCTCCGCCCCCCTGACGAGCATCACAAAAATCGACGCTCA
AGTCAGAGGTGGCGAAACCCGACAGGACTATAAAGATACCAGGCGTTTCC
CCCTGGAAGCTCCCTCGTGCGCTCTCCTGTTCCGACCCTGCCGCTTACCG
GATACCTGTCCGCCTTTCTCCCTTCGGGAAGCGTGGCGCTTTCTCATAGC
TCACGCTGTAGGTATCTCAGTTCGGTGTAGGTCGTTCGCTCCAAGCTGGG
CTGTGTGCACGAACCCCCCGTTCAGCCCGACCGCTGCGCCTTATCCGGTA
ACTATCGTCTTGAGTCCAACCCGGTAAGACACGACTTATCGCCACTGGCA
GCAGCCACTGGTAACAGGATTAGCAGAGCGAGGTATGTAGGCGGTGCTAC
AGAGTTCTTGAAGTGGTGGCCTAACTACGGCTACACTAGAAGGACAGTAT
TTGGTATCTGCGCTCTGCTGAAGCCAGTTACCTTCGGAAAAAGAGTTGGT

AGCTCTTGATCCGGCAAACAAACCACCGCTGGTAGCGGTGGTTTTTTTGT
TTGCAAGCAGCAGATTACGCGCAGAAAAAAAGGATCTCAAGAAGATCCTT
TGATCTTTTCTACGGGGTCTGACGCTCAGTGGAACGAAAACTCACGTTAA
GGGATTTTGGTCATGAGATTATCAAAAAGGATCTTCACCTAGATCCTTTT
AAATTAAAAATGAAGTTTTAAATCAATCTAAAGTATATATGAGTAAACTT
GGTCTGACAGTTACCAATGCTTAATCAGTGAGGCACCTATCTCAGCGATC
TGTCTATTTCGTTCATCCATAGTTGCCTGACTCCCCGTCGTGTAGATAAC
TACGATACGGGAGGGCTTACCATCTGGCCCCAGTGCTGCAATGATACCGC
GAGACCCACGCTCACCGGCTCCAGATTTATCAGCAATAAACCAGCCAGCC
GGAAGGGCCGAGCGCAGAAGTGGTCCTGCAACTTTATCCGCCTCCATCCA
GTCTATTAATTGTTGCCGGGAAGCTAGAGTAAGTAGTTCGCCAGTTAATA
GTTTGCGCAACGTTGTTGCCATTGCTACAGGCATCGTGGTGTCACGCTCG
TCGTTTGGTATGGCTTCATTCAGCTCCGGTTCCCAACGATCAAGGCGAGT
TACATGATCCCCCATGTTGTGCAAAAAAGCGGTTAGCTCCTTCGGTCCTC
CGATCGTTGTCAGAAGTAAGTTGGCCGCAGTGTTATCACTCATGGTTATG
GCAGCACTGCATAATTCTCTTACTGTCATGCCATCCGTAAGATGCTTTTC
TGTGACTGGTGAGTACTCAACCAAGTCATTCTGAGAATAGTGTATGCGGC
GACCGAGTTGCTCTTGCCCGGCGTCAATACGGGATAATACCGCGCCACAT
AGCAGAACTTTAAAAGTGCTCATCATTGGAAAACGTTCTTCGGGGCGAAA
ACTCTCAAGGATCTTACCGCTGTTGAGATCCAGTTCGATGTAACCCACTC
GTGCACCCAACTGATCTTCAGCATCTTTTACTTTCACCAGCGTTTCTGGG
TGAGCAAAAACAGGAAGGCAAAATGCCGCAAAAAAGGGAATAAGGGCGAC
ACGGAAATGTTGAATACTCATACTCTTCCTTTTTCAATATTATTGAAGCA
TTTATCAGGGTTATTGTCTCATGAGCGGATACATATTTGAATGTATTTAG
AAAAATAAACAAATAGGGGTTCCGCGCACATTTCCCCGAAAAGTGCCACC
TGACGCGCCCTGTAGCGGGATCA

pDM1515 – GFP (C)

ACTAGCGCCGGCATTACATGTCCATTTATTAGAAGGATTCAATCAAAAAT
TAACACCAGGTATATTACCAGATGGCATTAATAATTTATATTCTTTAATA
TTGGAAAAAGAAAAAAATAAATAAACAATCAATCAGAAAAGATAATTGAA
AAAAAAAAAAAAGAAAATGTTTAATAAGAATTAATTTGGAAGTAAAATAA
TTTTTGGATGAGTAAATATTAATAAACTCACAATTAAAATTTAGAATCCA
AAAAAATGAAAAAAAAAAAAATTTTTAAAACAGAATGGTTGCATTCATTT
TTTTTTTTTTTTTTTTATATCTTTTGGGTATTTTTGGGTGTTCTATTTAT
CTGGATAAAAAATAAAATGGAATATAGTTTTATATATATATTTTTTTCGT
ATCAAGCTTGAATGTTTTTATCAATAATATGATTGTTGGTTGCTTTATTT
TATTTGAAAAAAAAATTAATTTTTAAAATATCTTTTTTTTCATTTTATTT
GAAAAAAAAAAAAAAAAAAAATGGTTTTGAAAATTTTTAAAAAAATGGTT
GGGTCACCCACAATTCTTATAAAAAAAAGCCATTTGGATAGTTTTTTTTT
TTTTTTTTTTTATTTTTTTTTATTTTTTTTTTTTGAGATTTGGTATAAAT
ACAAAAAAAAAATTGTTTTTAAATCATTATTAATAAAAACTTAAAATAAA
TTATATATAAAAAAAGATCTTCAACTAGTGGTGGTTCAGGAGGTAGTTCA
AAAGGTGAAGAATTATTTACAGGTGTTGTTCCAATTTTAGTTGAATTAGA
TGGTGATGTTAATGGTCATAAATTTTCAGTTTCAGGTGAAGGTGAAGGTG
ATGCAACATATGGTAAATTAACATTAAAATTTATTTGTACAACAGGTAAA
TTACCAGTTCCATGGCCAACATTAGTTACAACATTTACATATGGTGTTCA
ATGTTTTTCAAGATATCCAGATCATATGAAACAACATGATTTTTTTAAAT
CAGCAATGCCAGAAGGTTATGTTCAAGAAAGAACAATTTTTTTAAAGAT

GATGGTAATTATAAAACAAGAGCAGAAGTTAAATTTGAAGGTGATACATT
AGTTAATAGAATTGAATTAAAAGGTATTGATTTTAAAGAAGATGGTAATA
TTTTAGGTCATAAATTAGAATATAATTATAATTCACATAATGTTTATATT
ATGGCAGATAAACAAAAAAATGGTATTAAAGTTAATTTTAAAATTAGACA
TAATATTGAAGATGGTTCAGTTCAATTAGCAGATCATTATCAACAAAATA
CACCAATTGGTGATGGTCCAGTTTTATTACCAGATAATCATTATTTATCA
ACACAATCAGCATTATCAAAAGATCCAAATGAAAAAAGAGATCATATGGT
TTTATTAGAATTTGTTACAGCAGCAGGTATTACACATGGTATGGATGAAT
TATATAAATAAGCTAGTATAAAATAAATAAATTGTGGATCTGATATCATA
ACTTCGTATAATGTATGCTATACGAAGTTATACTACTCTCGAGATTTTAT
TTAATATACTAAATAATAAAAAAGTTAAAAAATGATCATTGGATAAATTT
TTTATAATTATAAATAAAGATAATAATTTTTTTTTTAACAAAACTAAAAA
TAAAAATAATAAAATAATTGTTAAAATAGGTTTTTTTTTTTTTTTTTTTT
TTTTAATAAATGGTATTTATTAATTTATTTGTTGTGTGTGTTTTTTTTTT
TATAATATTTTTTTTTTTAGCATTGAATTAAGAAGAAATCAAATTGATGC
GGCCGCTTAGTTAGCCTCCCCCATCTCCCGATCCGGACGAGTGCTGGGGC
GTCGGTTTCCACTATCGGCGAGTACTTCTACACAGCCATCGGTCCAGACG
GCCGCGCTTCTGCGGGCGATTTGTGTACGCCCGACAGTCCCGGCTCCGGA
TCGGACGATTGCGTCGCATCGACCCTGCGCCCAAGCTGCATCATCGAAAT
TGCCGTCAACCAAGCTCTGATAGAGTTGGTCAAGACCAATGCGGAGCATA
TACGCCCGGAGCCGCGGCGATCCTGCAAGCTCCGGATGCCTCCGCTCGAA
GTAGCGCGTCTGCTGCTCCATACAAGCCAACCACGGCCTCCAGAAGAAGA
TGTTGGCGACCTCGTATTGGGAATCCCCGAACATCGCCTCGCTCCAGTCA
ATGACCGCTGTTATGCGGCCATTGTCCGTCAGGACATTGTTGGAGCCGAA
ATCCGCGTGCACGAGGTGCCGGACTTCGGGGCAGTCCTCGGCCCAAAGCA
TCAGCTCATCGAGAGCCTGCGCGACGGACGCACTGACGGTGTCGTCCATC
ACAGTTTGCCAGTGATACACATGGGGATCAGCAATCGCGCATATGAAATC
ACGCCATGTAGTGTATTGACCGATTCCTTGCGGTCCGAATGGGCCGAACC
CGCTCGTCTGGCTAAGATCGGCCGCAGCGATCGCATCCATGGCCTCCGCG
ACCGGCTGCAGAACAGCGGGCAGTTCGGTTTCAGGCAGGTCTTGCAACGT
GACACCCTGTGCACGGCGGGAGATGCAATAGGTCAGGCTCTCGCTGAACT
CCCCAATGTCAAGCACTTCCGGAATCGGGAGCGCGGCCGATGCAAAGTGC
CGATAAACATAACGATCTTTGTAGAAACCATCGGCGCAGCTATTTACCCG
CAGGACATATCCACGCCCTCCTACATCGAAGCTGAAAGCACGAGATTCTT
CGCCCTCCGAGAGCTGCATCAGGTCGGAGACGCTGTCGAACTTTTCGATC
AGAAACTTCTCGACAGACGTCGCGGTGAGTTCAGGCTTTTTCATTAGGTC
TTGTTGAGAAATGTTAAATTGATCCATTTTTTGCTAGCTGTGAAATTAGT
TTAAAATACAAATAAAGAGTTATAATAATATACAGTTGAATAAAAAAAAA
AAAAATGAATTGGAAAATTTATTTTTATATGAAGAAAAAAAAATTTTGAA
AAAAAAAAAAAAAATTAAAAAAAAAAAAAAAAAAAAAAAAAATTAAAAAT
AATTCAACTGTGGGTGACCCAAATTTTATTTAAAAAAAAAAAAAAAATGG
ATCACTTTTGGGTGGTTGGAAAAAAAAAAAAAAAAAAATTGAAAATATAA
TGTTAGTCATATGATTAATCATTAATTAATTCTTTTTTTTTTTTTTTTTA
TTTTTTATTTTTTTTTTTTCAATATTCCAATTATGACACACACTAACATC
ACAAAATACAAAATTTTGGGATCCACTACTATAACTTCGTATAATGTATG
CTATACGAAGTTATTTAATTAATTAAGATATCAAGCTTGTCCACAGAAAA
TGTTTCTAAACAAAAAAAAAAAACCGAGTGATGAAAGCGCTTCTCACAAA
ATTATTTATGTAAAATATTTAATAAAATACATTATTTAAATCTTTTTTAT
TTTTTTTAGTTGTTGATCTTTATCCGACTTTAAAAATAAAAAAATTGTAA
AAAAAAAAAAAGTTTATTTGTTTAATTTTATTGTTGTTTTAATTTTTA

CAACCATTAATTTATAAAACCAACACCTAACTATAATGAAATACAATTTG
TATCTTGGAAAAAAAAAAAATTAATAATATAAATAAAATGTAAACAAGTAT
CCGGTGGAAGGAACTAACAGTTGCCTTATCCAGTTCAAAAAAAAAAAAAA
AAAAAGAAATTAAATTACCATTTTATATTTAACTAAAATAAATTAAAAGG
TTTATTTTAATAATAGATTTATTATTTTTTAAAAATCAATAATTAATGTT
GGATAAGATGAACGAATAAAAGATAGAGTTTCAGGTCTTTTAAATGATGA
GGATTTATGAATTCTTAAAAGTTTTGGGCAAGAGAAAATGATTTTAACGA
TTGAACCATCACTAATTGCAAAATCTCTTGAACTAAGAGTTTTTAAGTTT
GAAGCAAGTGGAGAATTACAAAGAGCAATCATAGAGTTATCAGTTAAATC
ATAACAACAAGATAAAGATAAAGAACTAATATGATGTAAATTTTCAATCA
TACCTCTAAAAGAAACATCATTCATACTTTTTGGATAAATTAAATCTAAT
GAATCTAAATTTGGAAATAGTAAACCAATATTTGGCCGGCAGAGGCGGTT
TGCGTATTGGGCGCTCTTCCGCTTCCTCGCTCACTGACTCGCTGCGCTCG
GTCGTTCGGCTGCGGCGAGCGGTATCAGCTCACTCAAAGGCGGTAATACG
GTTATCCACAGAATCAGGGGATAACGCAGGAAAGAACATGTGAGCAAAAG
GCCAGCAAAAGGCCAGGAACCGTAAAAAGGCCGCGTTGCTGGCGTTTTTC
CATAGGCTCCGCCCCCCTGACGAGCATCACAAAAATCGACGCTCAAGTCA
GAGGTGGCGAAACCCGACAGGACTATAAAGATACCAGGCGTTTCCCCCTG
GAAGCTCCCTCGTGCGCTCTCCTGTTCCGACCCTGCCGCTTACCGGATAC
CTGTCCGCCTTTCTCCCTTCGGGAAGCGTGGCGCTTTCTCATAGCTCACG
CTGTAGGTATCTCAGTTCGGTGTAGGTCGTTCGCTCCAAGCTGGGCTGTG
TGCACGAACCCCCCGTTCAGCCCGACCGCTGCGCCTTATCCGGTAACTAT
CGTCTTGAGTCCAACCCGGTAAGACACGACTTATCGCCACTGGCAGCAGC
CACTGGTAACAGGATTAGCAGAGCGAGGTATGTAGGCGGTGCTACAGAGT
TCTTGAAGTGGTGGCCTAACTACGGCTACACTAGAAGGACAGTATTTGGT
ATCTGCGCTCTGCTGAAGCCAGTTACCTTCGGAAAAAGAGTTGGTAGCTC
TTGATCCGGCAAACAAACCACCGCTGGTAGCGGTGGTTTTTTTGTTTGCA
AGCAGCAGATTACGCGCAGAAAAAAAGGATCTCAAGAAGATCCTTTGATC
TTTTCTACGGGGTCTGACGCTCAGTGGAACGAAAACTCACGTTAAGGGAT
TTTGGTCATGAGATTATCAAAAAGGATCTTCACCTAGATCCTTTTAAATT
AAAAATGAAGTTTTAAATCAATCTAAAGTATATATGAGTAAACTTGGTCT
GACAGTTACCAATGCTTAATCAGTGAGGCACCTATCTCAGCGATCTGTCT
ATTTCGTTCATCCATAGTTGCCTGACTCCCCGTCGTGTAGATAACTACGA
TACGGGAGGGCTTACCATCTGGCCCCAGTGCTGCAATGATACCGCGAGAC
CCACGCTCACCGGCTCCAGATTTATCAGCAATAAACCAGCCAGCCGGAAG
GGCCGAGCGCAGAAGTGGTCCTGCAACTTTATCCGCCTCCATCCAGTCTA
TTAATTGTTGCCGGGAAGCTAGAGTAAGTAGTTCGCCAGTTAATAGTTTG
CGCAACGTTGTTGCCATTGCTACAGGCATCGTGGTGTCACGCTCGTCGTT
TGGTATGGCTTCATTCAGCTCCGGTTCCCAACGATCAAGGCGAGTTACAT
GATCCCCCATGTTGTGCAAAAAAGCGGTTAGCTCCTTCGGTCCTCCGATC
GTTGTCAGAAGTAAGTTGGCCGCAGTGTTATCACTCATGGTTATGGCAGC
ACTGCATAATTCTCTTACTGTCATGCCATCCGTAAGATGCTTTTCTGTGA
CTGGTGAGTACTCAACCAAGTCATTCTGAGAATAGTGTATGCGGCGACCG
AGTTGCTCTTGCCCGGCGTCAATACGGGATAATACCGCGCCACATAGCAG
AACTTTAAAAGTGCTCATCATTGGAAAACGTTCTTCGGGGCGAAAACTCT
CAAGGATCTTACCGCTGTTGAGATCCAGTTCGATGTAACCCACTCGTGCA
CCCAACTGATCTTCAGCATCTTTTACTTTCACCAGCGTTTCTGGGTGAGC
AAAAACAGGAAGGCAAAATGCCGCAAAAAAGGGAATAAGGGCGACACGGA
AATGTTGAATACTCATACTCTTCCTTTTTCAATATTATTGAAGCATTTAT
CAGGGTTATTGTCTCATGAGCGGATACATATTTGAATGTATTTAGAAAAA

TAAACAAATAGGGGTTCCGCGCACATTTCCCCGAAAAGTGCCACCTGACG
CGCCCTGTAGCGGGATCA

pDM1514 – mCherry (N)

ACTAGCGCCGGCATTACATGTCCATTTATTAGAAGGATTCAATCAAAAAT
TAACACCAGGTATATTACCAGATGGCATTAATAATTTATATTCTTTAATA
TTGGAAAAAGAAAAAAATAAATAAACAATCAATCAGAAAAGATAATTGAA
AAAAAAAAAAAAAGAAAATGTTTAATAAGAATTAATTTGGAAGTAAAATAA
TTTTTGGATGAGTAAATATTAATAAACTCACAATTAAAATTTAGAATCCA
AAAAAATGAAAAAAAAAAAAAATTTTTAAAACAGAATGGTTGCATTCATTT
TTTTTTTTTTTTTTTTATATCTTTTGGGTATTTTTGGGTGTTCTATTTAT
CTGGATAAAAAATAAAATGGAATATAGTTTTATATATATATTTTTTTCGT
ATCAAGCTTGAATGTTTTTATCAATAATATGATTGTTGGTTGCTTTATTT
TATTTGAAAAAAAAATTAATTTTTAAAATATCTTTTTTTTCATTTTATTT
GAAAAAAAAAAAAAAAAAAAATGGTTTTGAAAATTTTTAAAAAAATGGTT
GGGTCACCCACAATTCTTATAAAAAAAAAGCCATTTGGATAGTTTTTTTT
TTTTTTTTTTTATTTTTTTTATTTTTTTTTTTTTGAGATTTGGTATAAAT
ACAAAAAAAAAAATTGTTTTTAAATCATTATTAATAAAAACTTAAAATAAA
TTATATATAAAAAAAGATCCAAAAAATGGTTTCAAAAGGTGAAGAAGATA
ATATGGCAATTATTAAAGAATTTATGAGATTTAAAGTTCATATGGAAGGT
TCAGTTAATGGTCATGAATTTGAAATTGAAGGTGAAGGTGAAGGTAGACC
ATATGAAGGTACACAAACAGCAAAATTAAAAGTTACAAAAGGTGGTCCAT
TACCATTTGCATGGGATATTTTATCACCACAATTTATGTATGGTTCAAAA
GCATATGTTAAACATCCAGCAGATATTCCAGATTATTTAAAATTATCATT
TCCAGAAGGTTTTAAATGGGAAAGAGTTATGAATTTTGAAGATGGTGGTG
TTGTTACAGTTACACAAGATTCATCATTACAAGATGGTGAATTTATTTAT
AAAGTTAAATTAAGAGGTACAAATTTTCCATCAGATGGTCCAGTTATGCA
AAAAAAAACAATGGGTTGGGAAGCATCATCAGAAAGAATGTATCCAGAAG
ATGGTGCATTAAAAGGTGAAATTAAACAAAGATTAAAATTAAAAGATGGT
GGTCATTATGATGCAGAAGTTAAAACAACATATAAAGCAAAAAAACCAGT
TCAATTACCAGGTGCATATAATGTTAATATTAAATTAGATATTACATCAC
ATAATGAAGATTATACAATTGTTGAACAATATGAAAGAGCAGAAGGTAGA
CATTCAACAGGTGGTATGGATGAATTATATAAAGGTGGTTCAGGAGGTAG
TAGATCTTCAACTAGTATAAAATAAATAAATTGTGGATCTGATATCATAA
CTTCGTATAATGTATGCTATACGAAGTTATACTACTCTCGAGATTTTATT
TAATATACTAAATAATAAAAAAGTTAAAAAATGATCATTGGATAAATTTT
TTATAATTATAAATAAAGATAATAATTTTTTTTTTAACAAAACTAAAAAT
AAAAATAATAAAATAATTGTTAAAATAGGTTTTTTTTTTTTTTTTTTTTT
TTTAATAAATGGTATTTATTAATTTATTTGTTGTGTGTGTTTTTTTTTTT
ATAATATTTTTTTTTTTTAGCATTGAATTAAGAAGAAATCAAATTGATGCG
GCCGCTTAGTTAGCCTCCCCCATCTCCCGATCCGGACGAGTGCTGGGGCG
TCGGTTTCCACTATCGGCGAGTACTTCTACACAGCCATCGGTCCAGACGG
CCGCGCTTCTGCGGGCGATTTGTGTACGCCCGACAGTCCCGGCTCCGGAT
CGGACGATTGCGTCGCATCGACCCTGCGCCCAAGCTGCATCATCGAAATT
GCCGTCAACCAAGCTCTGATAGAGTTGGTCAAGACCAATGCGGAGCATAT
ACGCCCGGAGCCGCGGCGATCCTGCAAGCTCCGGATGCCTCCGCTCGAAG
TAGCGCGTCTGCTGCTCCATACAAGCCAACCACGGCCTCCAGAAGAAGAT
GTTGGCGACCTCGTATTGGGAATCCCCGAACATCGCCTCGCTCCAGTCAA
TGACCGCTGTTATGCGGCCATTGTCCGTCAGGACATTGTTGGAGCCGAAA

TCCGCGTGCACGAGGTGCCGGACTTCGGGGCAGTCCTCGGCCCAAAGCAT
CAGCTCATCGAGAGCCTGCGCGACGGACGCACTGACGGTGTCGTCCATCA
CAGTTTGCCAGTGATACACATGGGGATCAGCAATCGCGCATATGAAATCA
CGCCATGTAGTGTATTGACCGATTCCTTGCGGTCCGAATGGGCCGAACCC
GCTCGTCTGGCTAAGATCGGCCGCAGCGATCGCATCCATGGCCTCCGCGA
CCGGCTGCAGAACAGCGGGCAGTTCGGTTTCAGGCAGGTCTTGCAACGTG
ACACCCTGTGCACGGCGGGAGATGCAATAGGTCAGGCTCTCGCTGAACTC
CCCAATGTCAAGCACTTCCGGAATCGGGAGCGCGGCCGATGCAAAGTGCC
GATAAACATAACGATCTTTGTAGAAACCATCGGCGCAGCTATTTACCCGC
AGGACATATCCACGCCCTCCTACATCGAAGCTGAAAGCACGAGATTCTTC
GCCCTCCGAGAGCTGCATCAGGTCGGAGACGCTGTCGAACTTTTCGATCA
GAAACTTCTCGACAGACGTCGCGGTGAGTTCAGGCTTTTTCATTAGGTCT
TGTTGAGAAATGTTAAATTGATCCATTTTTTGCTAGCTGTGAAATTAGTT
TAAAATACAAATAAAGAGTTATAATAATATACAGTTGAATAAAAAAAAAA
AAAATGAATTGGAAAATTTATTTTTATATGAAGAAAAAAAAATTTTGAAA
AAAAAAAAAAAAAATTAAAAAAAAAAAAAAAAAAAAAAAAATTAAAAATA
ATTCAACTGTGGGTGACCCAAATTTTATTTAAAAAAAAAAAAAAAATGGA
TCACTTTTGGGTGGTTGGAAAAAAAAAAAAAAAAAAATTGAAAATATAAT
GTTAGTCATATGATTAATCATTAATTAATTCTTTTTTTTTTTTTTTTTAT
TTTTTATTTTTTTTTTTTTTCAATATTCCAATTATGACACACTAACATCA
CAAAATACAAAATTTTGGGATCCACTACTATAACTTCGTATAATGTATGC
TATACGAAGTTATTTAATTAATTAAGATATCAAGCTTGTCCACAGAAAAT
GTTTCTAAACAAAAAAAAAAAACCGAGTGATGAAAGCGCTTCTCACAAAA
TTATTTATGTAAAATATTTAATAAAATACATTATTTAAATCTTTTTTATT
TTTTTTAGTTGTTGATCTTTATCCGACTTTAAAAATAAAAAAAATTGTAAA
AAAAAAAAAAAGTTTATTTGTTTAATTTTATTGTTGTTTTTAATTTTTAC
AACCATTAATTTATAAAACCAACACCTAACTATAATGAAATACAATTTGT
ATCTTGGAAAAAAAAAAATTAATAATATAAATAAAATGTAAACAAGTATC
CGGTGGAAGGAACTAACAGTTGCCTTATCCAGTTCAAAAAAAAAAAAAAA
AAAAGAAATTAAATTACCATTTTATATTTAACTAAAATAAATTAAAAGGT
TTATTTTAATAATAGATTTATTATTTTTTAAAAATCAATAATTAATGTTG
GATAAGATGAACGAATAAAAGATAGAGTTTCAGGTCTTTTAAATGATGAG
GATTTATGAATTCTTAAAAGTTTTGGGCAAGAGAAAATGATTTTAACGAT
TGAACCATCACTAATTGCAAAATCTCTTGAACTAAGAGTTTTTAAGTTTG
AAGCAAGTGGAGAATTACAAAGAGCAATCATAGAGTTATCAGTTAAATCA
TAACAACAAGATAAAGATAAAGAACTAATATGATGTAAATTTTCAATCAT
ACCTCTAAAAGAAACATCATTCATACTTTTTGGATAAATTAAATCTAATG
AATCTAAATTTGGAAATAGTAAACCAATATTTGGCCGGCAGAGGCGGTTT
GCGTATTGGGCGCTCTTCCGCTTCCTCGCTCACTGACTCGCTGCGCTCGG
TCGTTCGGCTGCGGCGAGCGGTATCAGCTCACTCAAAGGCGGTAATACGG
TTATCCACAGAATCAGGGGATAACGCAGGAAAGAACATGTGAGCAAAAGG
CCAGCAAAAGGCCAGGAACCGTAAAAAGGCCGCGTTGCTGGCGTTTTTCC
ATAGGCTCCGCCCCCCTGACGAGCATCACAAAAATCGACGCTCAAGTCAG
AGGTGGCGAAACCCGACAGGACTATAAAGATACCAGGCGTTTCCCCCTGG
AAGCTCCCTCGTGCGCTCTCCTGTTCCGACCCTGCCGCTTACCGGATACC
TGTCCGCCTTTCTCCCTTCGGGAAGCGTGGCGCTTTCTCATAGCTCACGC
TGTAGGTATCTCAGTTCGGTGTAGGTCGTTCGCTCCAAGCTGGGCTGTGT
GCACGAACCCCCCGTTCAGCCCGACCGCTGCGCCTTATCCGGTAACTATC
GTCTTGAGTCCAACCCGGTAAGACACGACTTATCGCCACTGGCAGCAGCC
ACTGGTAACAGGATTAGCAGAGCGAGGTATGTAGGCGGTGCTACAGAGTT

CTTGAAGTGGTGGCCTAACTACGGCTACACTAGAAGGACAGTATTTGGTA
TCTGCGCTCTGCTGAAGCCAGTTACCTTCGGAAAAAGAGTTGGTAGCTCT
TGATCCGGCAAACAAACCACCGCTGGTAGCGGTGGTTTTTTTGTTTGCAA
GCAGCAGATTACGCGCAGAAAAAAAGGATCTCAAGAAGATCCTTTGATCT
TTTCTACGGGGTCTGACGCTCAGTGGAACGAAAACTCACGTTAAGGGATT
TTGGTCATGAGATTATCAAAAAGGATCTTCACCTAGATCCTTTTAAATTA
AAAATGAAGTTTTAAATCAATCTAAAGTATATATGAGTAAACTTGGTCTG
ACAGTTACCAATGCTTAATCAGTGAGGCACCTATCTCAGCGATCTGTCTA
TTTCGTTCATCCATAGTTGCCTGACTCCCCGTCGTGTAGATAACTACGAT
ACGGGAGGGCTTACCATCTGGCCCCAGTGCTGCAATGATACCGCGAGACC
CACGCTCACCGGCTCCAGATTTATCAGCAATAAACCAGCCAGCCGGAAGG
GCCGAGCGCAGAAGTGGTCCTGCAACTTTATCCGCCTCCATCCAGTCTAT
TAATTGTTGCCGGGAAGCTAGAGTAAGTAGTTCGCCAGTTAATAGTTTGC
GCAACGTTGTTGCCATTGCTACAGGCATCGTGGTGTCACGCTCGTCGTTT
GGTATGGCTTCATTCAGCTCCGGTTCCCAACGATCAAGGCGAGTTACATG
ATCCCCCATGTTGTGCAAAAAAGCGGTTAGCTCCTTCGGTCCTCCGATCG
TTGTCAGAAGTAAGTTGGCCGCAGTGTTATCACTCATGGTTATGGCAGCA
CTGCATAATTCTCTTACTGTCATGCCATCCGTAAGATGCTTTTCTGTGAC
TGGTGAGTACTCAACCAAGTCATTCTGAGAATAGTGTATGCGGCGACCGA
GTTGCTCTTGCCCGGCGTCAATACGGGATAATACCGCGCCACATAGCAGA
ACTTTAAAAGTGCTCATCATTGGAAAACGTTCTTCGGGGCGAAAACTCTC
AAGGATCTTACCGCTGTTGAGATCCAGTTCGATGTAACCCACTCGTGCAC
CCAACTGATCTTCAGCATCTTTTACTTTCACCAGCGTTTCTGGGTGAGCA
AAAACAGGAAGGCAAAATGCCGCAAAAAAGGGAATAAGGGCGACACGGAA
ATGTTGAATACTCATACTCTTCCTTTTTCAATATTATTGAAGCATTTATC
AGGGTTATTGTCTCATGAGCGGATACATATTTGAATGTATTTAGAAAAAT
AAACAAATAGGGGTTCCGCGCACATTTCCCCGAAAAGTGCCACCTGACGC
GCCCTGTAGCGGGATCA

pDM1516 – mCherry (C)

ACTAGCGCCGGCATTACATGTCCATTTATTAGAAGGATTCAATCAAAAAT
TAACACCAGGTATATTACCAGATGGCATTAATAATTTATATTCTTTAATA
TTGGAAAAAGAAAAAAATAAATAAACAATCAATCAGAAAAGATAATTGAA
AAAAAAAAAAAAGAAAATGTTTAATAAGAATTAATTTGGAAGTAAAATAA
TTTTTGGATGAGTAAATATTAATAAACTCACAATTAAAATTTAGAATCCA
AAAAAATGAAAAAAAAAAAAATTTTTAAAACAGAATGGTTGCATTCATTT
TTTTTTTTTTTTTTTTATATCTTTTGGGTATTTTTGGGTGTTCTATTTAT
CTGGATAAAAAATAAAATGGAATATAGTTTTATATATATATTTTTTTCGT
ATCAAGCTTGAATGTTTTTATCAATAATATGATTGTTGGTTGCTTTATTT
TATTTGAAAAAAAAATTAATTTTTAAAATATCTTTTTTTTCATTTTATTT
GAAAAAAAAAAAAAAAAAAAATGGTTTTGAAAATTTTTAAAAAAATGGTT
GGGTCACCCACAATTCTTATAAAAAAAAAGCCATTTGGATAGTTTTTTTT
TTTTTTTTTTTATTTTTTTTATTTTTTTTTTTTTGAGATTTGGTATAAAT
ACAAAAAAAAAATTGTTTTTAAATCATTATTAATAAAAACTTAAAATAAA
TTATATATAAAAAAAGATCTTCAACTAGTGGTGGTTCAGGAGGTAGTGTT
TCAAAAGGTGAAGAAGATAATATGGCAATTATTAAAGAATTTATGAGATT
TAAAGTTCATATGGAAGGTTCAGTTAATGGTCATGAATTTGAAATTGAAG
GTGAAGGTGAAGGTAGACCATATGAAGGTACACAAACAGCAAAATTAAAA
GTTACAAAAGGTGGTCCATTACCATTTGCATGGGATATTTTATCACCACA

ATTTATGTATGGTTCAAAAGCATATGTTAAACATCCAGCAGATATTCCAG
ATTATTTAAAATTATCATTTCCAGAAGGTTTTAAATGGGAAAGAGTTATG
AATTTTGAAGATGGTGGTGTTGTTACAGTTACACAAGATTCATCATTACA
AGATGGTGAATTTATTTATAAAGTTAAATTAAGAGGTACAAATTTTCCAT
CAGATGGTCCAGTTATGCAAAAGAAAACAATGGGTTGGGAAGCATCATCA
GAAAGAATGTATCCAGAAGATGGTGCATTAAAAGGTGAAATTAAACAAAG
ATTAAAATTAAAAGATGGTGGTCATTATGATGCAGAAGTTAAAACAACAT
ATAAAGCAAAAAAACCAGTTCAATTACCAGGTGCATATAATGTTAATATT
AAATTAGATATTACATCACATAATGAAGATTATACAATTGTTGAACAATA
TGAAAGAGCAGAAGGTAGACATTCAACAGGTGGTATGGATGAATTATATA
AATAAGCTAGTATAAAATAAATAAATTGTGGATCTGATATCATAACTTCG
TATAATGTATGCTATACGAAGTTATACTACTCTCGAGATTTTATTTAATA
TACTAAATAATAAAAAAGTTAAAAAATGATCATTGGATAAATTTTTTATA
ATTATAAATAAAGATAATAATTTTTTTTTTAACAAAACTAAAAATAAAAA
TAATAAAATAATTGTTAAAATAGGTTTTTTTTTTTTTTTTTTTTTTTTAA
TAAATGGTATTTATTAATTTATTTGTTGTGTGTGTTTTTTTTTTTATAAT
ATTTTTTTTTTTTAGCATTGAATTAAGAAGAATCAAATTGATGCGGCCGC
TTAGTTAGCCTCCCCCATCTCCCGATCCGGACGAGTGCTGGGGCGTCGGT
TTCCACTATCGGCGAGTACTTCTACACAGCCATCGGTCCAGACGGCCGCG
CTTCTGCGGGCGATTTGTGTACGCCCGACAGTCCCGGCTCCGGATCGGAC
GATTGCGTCGCATCGACCCTGCGCCCAAGCTGCATCATCGAAATTGCCGT
CAACCAAGCTCTGATAGAGTTGGTCAAGACCAATGCGGAGCATATACGCC
CGGAGCCGCGGCGATCCTGCAAGCTCCGGATGCCTCCGCTCGAAGTAGCG
CGTCTGCTGCTCCATACAAGCCAACCACGGCCTCCAGAAGAAGATGTTGG
CGACCTCGTATTGGGAATCCCCGAACATCGCCTCGCTCCAGTCAATGACC
GCTGTTATGCGGCCATTGTCCGTCAGGACATTGTTGGAGCCGAAATCCGC
GTGCACGAGGTGCCGGACTTCGGGGCAGTCCTCGGCCCAAAGCATCAGCT
CATCGAGAGCCTGCGCGACGGACGCACTGACGGTGTCGTCCATCACAGTT
TGCCAGTGATACACATGGGGATCAGCAATCGCGCATATGAAATCACGCCA
TGTAGTGTATTGACCGATTCCTTGCGGTCCGAATGGGCCGAACCCGCTCG
TCTGGCTAAGATCGGCCGCAGCGATCGCATCCATGGCCTCCGCGACCGGC
TGCAGAACAGCGGGCAGTTCGGTTTCAGGCAGGTCTTGCAACGTGACACC
CTGTGCACGGCGGGAGATGCAATAGGTCAGGCTCTCGCTGAACTCCCCAA
TGTCAAGCACTTCCGGAATCGGGAGCGCGGCCGATGCAAAGTGCCGATAA
ACATAACGATCTTTGTAGAAACCATCGGCGCAGCTATTTACCCGCAGGAC
ATATCCACGCCCTCCTACATCGAAGCTGAAAGCACGAGATTCTTCGCCCT
CCGAGAGCTGCATCAGGTCGGAGACGCTGTCGAACTTTTCGATCAGAAAC
TTCTCGACAGACGTCGCGGTGAGTTCAGGCTTTTTCATTAGGTCTTGTTG
AGAAATGTTAAATTGATCCATTTTTTGCTAGCTGTGAAATTAGTTTAAAA
TACAAATAAAGAGTTATAATAATATACAGTTGAATAAAAAAAAAAAAAAT
GAATTGGAAAATTTATTTTTATATGAAGAAAAAAAAATTTTGAAAAAAAA
AAAAAAAATTAAAAAAAAAAAAAAAAAAAAAAAAAATTAAAAATAATTCA
ACTGTGGGTGACCCAAATTTTATTTAAAAAAAAAAAAAAAATGGATCACT
TTTGGGTGGTTGGAAAAAAAAAAAAAAAAAAATTGAAAATATAATGTTAG
TCATATGATTAATCATTAATTAATTCTTTTTTTTTTTTTTTTTATTTTTT
ATTTTTTTTTTTTCAATATTCCAATTATGACACACACTAACATCACAAAA
TACAAAATTTTGGGATCCACTACTATAACTTCGTATAATGTATGCTATAC
GAAGTTATTTAATTAATTAAGATATCAAGCTTGTCCACAGAAAATGTTTC
TAAACAAAAAAAAAAAACCGAGTGATGAAAGCGCTTCTCACAAAATTATT
TATGTAAAATATTTAATAAAATACATTATTTAAATCTTTTTTATTTTTTT

TAGTTGTTGATCTTTATCCGACTTTAAAAATAAAAAAATTGTAAAAAAAA
AAAAAGTTTATTTGTTTAATTTTATTGTTGTTTTTAATTTTTTACAACCA
TTAATTTATAAAACCAACACCTAACTATAATGAAATACAATTTGTATCTT
GGAAAAAAAAAAATTAATAATATAAATAAAATGTAAACAAGTATCCGGTG
GAAGGAACTAACAGTTGCCTTATCCAGTTCAAAAAAAAAAAAAAAAAAAG
AAATTAAATTACCATTTTATATTTAACTAAAATAAATTAAAAGGTTTATT
TTAATAATAGATTTATTATTTTTTAAAAATCAATAATTAATGTTGGATAA
GATGAACGAATAAAAGATAGAGTTTCAGGTCTTTTAAATGATGAGGATTT
ATGAATTCTTAAAAGTTTTGGGCAAGAGAAAATGATTTTAACGATTGAAC
CATCACTAATTGCAAAATCTCTTGAACTAAGAGTTTTTAAGTTTGAAGCA
AGTGGAGAATTACAAAGAGCAATCATAGAGTTATCAGTTAAATCATAACA
ACAAGATAAAGATAAAGAACTAATATGATGTAAATTTTCAATCATACCTC
TAAAAGAAACATCATTCATACTTTTTGGATAAATTAAATCTAATGAATCT
AAATTTGGAAATAGTAAACCAATATTTGGCCGGCAGAGGCGGTTTGCGTA
TTGGGCGCTCTTCCGCTTCCTCGCTCACTGACTCGCTGCGCTCGGTCGTT
CGGCTGCGGCGAGCGGTATCAGCTCACTCAAAGGCGGTAATACGGTTATC
CACAGAATCAGGGGATAACGCAGGAAAGAACATGTGAGCAAAAGGCCAGC
AAAAGGCCAGGAACCGTAAAAAGGCCGCGTTGCTGGCGTTTTTCCATAGG
CTCCGCCCCCCTGACGAGCATCACAAAAATCGACGCTCAAGTCAGAGGTG
GCGAAACCCGACAGGACTATAAAGATACCAGGCGTTTCCCCCTGGAAGCT
CCCTCGTGCGCTCTCCTGTTCCGACCCTGCCGCTTACCGGATACCTGTCC
GCCTTTCTCCCTTCGGGAAGCGTGGCGCTTTCTCATAGCTCACGCTGTAG
GTATCTCAGTTCGGTGTAGGTCGTTCGCTCCAAGCTGGGCTGTGTGCACG
AACCCCCCGTTCAGCCCGACCGCTGCGCCTTATCCGGTAACTATCGTCTT
GAGTCCAACCCGGTAAGACACGACTTATCGCCACTGGCAGCAGCCACTGG
TAACAGGATTAGCAGAGCGAGGTATGTAGGCGGTGCTACAGAGTTCTTGA
AGTGGTGGCCTAACTACGGCTACACTAGAAGGACAGTATTTGGTATCTGC
GCTCTGCTGAAGCCAGTTACCTTCGGAAAAAGAGTTGGTAGCTCTTGATC
CGGCAAACAAACCACCGCTGGTAGCGGTGGTTTTTTTGTTTGCAAGCAGC
AGATTACGCGCAGAAAAAAAGGATCTCAAGAAGATCCTTTGATCTTTTCT
ACGGGGTCTGACGCTCAGTGGAACGAAAACTCACGTTAAGGGATTTTGGT
CATGAGATTATCAAAAAGGATCTTCACCTAGATCCTTTTAAATTAAAAAT
GAAGTTTTAAATCAATCTAAAGTATATATGAGTAAACTTGGTCTGACAGT
TACCAATGCTTAATCAGTGAGGCACCTATCTCAGCGATCTGTCTATTTCG
TTCATCCATAGTTGCCTGACTCCCCGTCGTGTAGATAACTACGATACGGG
AGGGCTTACCATCTGGCCCCAGTGCTGCAATGATACCGCGAGACCCACGC
TCACCGGCTCCAGATTTATCAGCAATAAACCAGCCAGCCGGAAGGGCCGA
GCGCAGAAGTGGTCCTGCAACTTTATCCGCCTCCATCCAGTCTATTAATT
GTTGCCGGGAAGCTAGAGTAAGTAGTTCGCCAGTTAATAGTTTGCGCAAC
GTTGTTGCCATTGCTACAGGCATCGTGGTGTCACGCTCGTCGTTTGGTAT
GGCTTCATTCAGCTCCGGTTCCCAACGATCAAGGCGAGTTACATGATCCC
CCATGTTGTGCAAAAAAGCGGTTAGCTCCTTCGGTCCTCCGATCGTTGTC
AGAAGTAAGTTGGCCGCAGTGTTATCACTCATGGTTATGGCAGCACTGCA
TAATTCTCTTACTGTCATGCCATCCGTAAGATGCTTTTCTGTGACTGGTG
AGTACTCAACCAAGTCATTCTGAGAATAGTGTATGCGGCGACCGAGTTGC
TCTTGCCCGGCGTCAATACGGGATAATACCGCGCCACATAGCAGAACTTT
AAAAGTGCTCATCATTGGAAAACGTTCTTCGGGGCGAAAACTCTCAAGGA
TCTTACCGCTGTTGAGATCCAGTTCGATGTAACCCACTCGTGCACCCAAC
TGATCTTCAGCATCTTTTACTTTCACCAGCGTTTCTGGGTGAGCAAAAAC
AGGAAGGCAAAATGCCGCAAAAAAGGGAATAAGGGCGACACGGAAATGTT

GAATACTCATACTCTTCCTTTTTCAATATTATTGAAGCATTTATCAGGGT
TATTGTCTCATGAGCGGATACATATTTGAATGTATTTAGAAAAATAAACA
AATAGGGGTTCCGCGCACATTTCCCCGAAAAGTGCCACCTGACGCGCCCT
GTAGCGGGATCA

pPI231 – mNeon (N)

ACTAGCGCCGGCATTACATGTCCATTTATTAGAAGGATTCAATCAAAAAT
TAACACCAGGTATATTACCAGATGGCATTAATAATTTATATTCTTTAATA
TTGGAAAAAGAAAAAAATAAATAAACAATCAATCAGAAAAGATAATTGAA
AAAAAAAAAAAAGAAAATGTTTAATAAGAATTAATTTGGAAGTAAAATAA
TTTTTGGATGAGTAAATATTAATAAACTCACAATTAAAATTTAGAATCCA
AAAAAATGAAAAAAAAAAAAATTTTTAAAACAGAATGGTTGCATTCATTT
TTTTTTTTTTTTTTTATATCTTTTGGGTATTTTTGGGTGTTCTATTTAT
CTGGATAAAAAATAAAATGGAATATAGTTTTATATATATATTTTTTTCGT
ATCAAGCTTGAATGTTTTTATCAATAATATGATTGTTGGTTGCTTTATTT
TATTTGAAAAAAAAATTAATTTTTAAAATATCTTTTTTTTCATTTTATTT
GAAAAAAAAAAAAAAAAAAAAATGGTTTTGAAAATTTTTAAAAAAATGGTT
GGGTCACCCACAATTCTTATAAAAAAAAGCCATTTGGATAGTTTTTTTT
TTTTTTTTTTATTTTTTTTATTTTTTTTTTTTTGAGATTTGGTATAAAT
ACAAAAAAAAAATTGTTTTTAAATCATTATTAATAAAAACTTAAAATAAA
TTATATATAAAAAAAGATCCAAAAAATGGTGAGTAAAGGTGAAGAAGATA
ATATGGCATCGTTACCAGCTACACATGAGTTACATATATTCGGTAGCATT
AATGGTGTTGATTTTGATATGGTGGGACAAGGTACCGGTAATCCTAATGA
TGGTTACGAAGAACTAAATTTAAAATCGACTAAAGGTGACTTACAATTTT
CTCCATGGATTTTAGTGCCACATATAGGGTATGGTTTTCATCAATACTTA
CCATATCCAGATGGTATGTCACCATTTCAAGCTGCAATGGTTGATGGATC
AGGTTATCAAGTTCATAGAACAATGCAATTTGAAGATGGTGCTTCATTAA
CTGTTAATTATAGATACACATATGAAGGCTCACATATTAAAGGTGAAGCT
CAAGTTAAAGGTACTGGTTTCCCAGCCGATGGCCCAGTTATGACAAATAG
TTTAACAGCAGCAGATTGGTGTAGATCCAAAAAAACTTATCCAAATGATA
AAACAATTATTTCAACTTTTAAATGGTCATATACAACCGGTAATGGTAAA
CGTTATCGTTCAACAGCCCGTACAACATATACTTTTGCTAAACCAATGGC
AGCTAATTATTTAAAAAATCAACCAATGTATGTTTTTCGTAAAACAGAGT
TAAAACATTCAAAAACAGAACTTAATTTTAAAGAATGGCAAAAAGCATTT
ACAGACGTTATGGGTATGGATGAACTTTATAAGGGTGGTTCAGGAGGTAG
TAGATCTTCAACTAGTATAAAATAAATAAATTGTGGATCTGATATCATAA
CTTCGTATAATGTATGCTATACGAAGTTATACTACTCTCGAGATTTTATT
TAATATACTAAATAATAAAAAAGTTAAAAAATGATCATTGGATAAATTTT
TTATAATTATAAATAAAGATAATAATTTTTTTTTTAACAAAACTAAAAAT
AAAAATAATAAAATAATTGTTAAAATAGGTTTTTTTTTTTTTTTTTTTTT
TTTAATAAATGGTATTTATTAATTTATTTGTTGTGTGTGTTTTTTTTTTT
ATAATATTTTTTTTTTTTAGCATTGAATTAAGAAGAAATCAAATTGATGCG
GCCGCTTAGTTAGCCTCCCCCATCTCCCGATCCGGACGAGTGCTGGGGCG
TCGGTTTCCACTATCGGCGAGTACTTCTACACAGCCATCGGTCCAGACGG
CCGCGCTTCTGCGGGCGATTTGTGTACGCCCGACAGTCCCGGCTCCGGAT
CGGACGATTGCGTCGCATCGACCCTGCGCCCAAGCTGCATCATCGAAATT
GCCGTCAACCAAGCTCTGATAGAGTTGGTCAAGACCAATGCGGAGCATAT
ACGCCCGGAGCCGCGGCGATCCTGCAAGCTCCGGATGCCTCCGCTCGAAG
TAGCGCGTCTGCTGCTCCATACAAGCCAACCACGGCCTCCAGAAGAAGAT

GTTGGCGACCTCGTATTGGGAATCCCCGAACATCGCCTCGCTCCAGTCAA
TGACCGCTGTTATGCGGCCATTGTCCGTCAGGACATTGTTGGAGCCGAAA
TCCGCGTGCACGAGGTGCCGGACTTCGGGGCAGTCCTCGGCCCAAAGCAT
CAGCTCATCGAGAGCCTGCGCGACGGACGCACTGACGGTGTCGTCCATCA
CAGTTTGCCAGTGATACACATGGGGATCAGCAATCGCGCATATGAAATCA
CGCCATGTAGTGTATTGACCGATTCCTTGCGGTCCGAATGGGCCGAACCC
GCTCGTCTGGCTAAGATCGGCCGCAGCGATCGCATCCATGGCCTCCGCGA
CCGGCTGCAGAACAGCGGGCAGTTCGGTTTCAGGCAGGTCTTGCAACGTG
ACACCCTGTGCACGGCGGGAGATGCAATAGGTCAGGCTCTCGCTGAACTC
CCCAATGTCAAGCACTTCCGGAATCGGGAGCGCGGCCGATGCAAAGTGCC
GATAAACATAACGATCTTTGTAGAAACCATCGGCGCAGCTATTTACCCGC
AGGACATATCCACGCCCTCCTACATCGAAGCTGAAAGCACGAGATTCTTC
GCCCTCCGAGAGCTGCATCAGGTCGGAGACGCTGTCGAACTTTTCGATCA
GAAACTTCTCGACAGACGTCGCGGTGAGTTCAGGCTTTTTCATTAGGTCT
TGTTGAGAAATGTTAAATTGATCCATTTTTTGCTAGCTGTGAAATTAGTT
TAAAATACAAATAAAGAGTTATAATAATATACAGTTGAATAAAAAAAAAA
AAAATGAATTGGAAAATTTATTTTTATATGAAGAAAAAAAAAATTTTGAAA
AAAAAAAAAAAAAATTAAAAAAAAAAAAAAAAAAAAAAAAAAAAATTAAAAATA
ATTCAACTGTGGGTGACCCAAATTTTATTTAAAAAAAAAAAAAAAAATGGA
TCACTTTTGGGTGGTTGGAAAAAAAAAAAAAAAAAAAAATTGAAAATATAAT
GTTAGTCATATGATTAATCATTAATTAATTCTTTTTTTTTTTTTTTTTAT
TTTTTATTTTTTTTTTTTCAATATTCCAATTATGACACACACTAACATCA
CAAAATACAAAATTTTGGGATCCACTACTATAACTTCGTATAATGTATGC
TATACGAAGTTATTTAATTAATTAAGATATCAAGCTTGTCCACAGAAAAT
GTTTCTAAACAAAAAAAAAAAAACCGAGTGATGAAAGCGCTTCTCACAAAA
TTATTTATGTAAAATATTTAATAAAATACATTATTTAAATCTTTTTTATT
TTTTTTAGTTGTTGATCTTTATCCGACTTTAAAAATAAAAAAAATTGTAAA
AAAAAAAAAAGTTTATTTGTTTAATTTTATTGTTGTTTTTAATTTTTTAC
AACCATTAATTTATAAAACCAACACCTAACTATAATGAAATACAATTTGT
ATCTTGGAAAAAAAAAAAATTAATAATATAAATAAAATGTAAACAAGTATC
CGGTGGAAGGAACTAACAGTTGCCTTATCCAGTTCAAAAAAAAAAAAAAA
AAAAGAAATTAAATTACCATTTTATATTTAACTAAAATAAATTAAAAGGT
TTATTTTAATAATAGATTTATTATTTTTTAAAAATCAATAATTAATGTTG
GATAAGATGAACGAATAAAAGATAGAGTTTCAGGTCTTTTAAATGATGAG
GATTTATGAATTCTTAAAAGTTTTGGGCAAGAGAAAATGATTTTAACGAT
TGAACCATCACTAATTGCAAAATCTCTTGAACTAAGAGTTTTTAAGTTTG
AAGCAAGTGGAGAATTACAAAGAGCAATCATAGAGTTATCAGTTAAATCA
TAACAACAAGATAAAGATAAAGAACTAATATGATGTAAATTTTCAATCAT
ACCTCTAAAAGAAACATCATTCATACTTTTTGGATAAATTAAATCTAATG
AATCTAAATTTGGAAATAGTAAACCAATATTTGGCCGGCAGAGGCGGTTT
GCGTATTGGGCGCTCTTCCGCTTCCTCGCTCACTGACTCGCTGCGCTCGG
TCGTTCGGCTGCGGCGAGCGGTATCAGCTCACTCAAAGGCGGTAATACGG
TTATCCACAGAATCAGGGGATAACGCAGGAAAGAACATGTGAGCAAAAGG
CCAGCAAAAGGCCAGGAACCGTAAAAAGGCCGCGTTGCTGGCGTTTTTCC
ATAGGCTCCGCCCCCCTGACGAGCATCACAAAAATCGACGCTCAAGTCAG
AGGTGGCGAAACCCGACAGGACTATAAAGATACCAGGCGTTTCCCCCTGG
AAGCTCCCTCGTGCGCTCTCCTGTTCCGACCCTGCCGCTTACCGGATACC
TGTCCGCCTTTCTCCCTTCGGGAAGCGTGGCGCTTTCTCATAGCTCACGC
TGTAGGTATCTCAGTTCGGTGTAGGTCGTTCGCTCCAAGCTGGGCTGTGT
GCACGAACCCCCCGTTCAGCCCGACCGCTGCGCCTTATCCGGTAACTATC

GTCTTGAGTCCAACCCGGTAAGACACGACTTATCGCCACTGGCAGCAGCC
ACTGGTAACAGGATTAGCAGAGCGAGGTATGTAGGCGGTGCTACAGAGTT
CTTGAAGTGGTGGCCTAACTACGGCTACACTAGAAGGACAGTATTTGGTA
TCTGCGCTCTGCTGAAGCCAGTTACCTTCGGAAAAAGAGTTGGTAGCTCT
TGATCCGGCAAACAAACCACCGCTGGTAGCGGTGGTTTTTTTGTTTGCAA
GCAGCAGATTACGCGCAGAAAAAAAGGATCTCAAGAAGATCCTTTGATCT
TTTCTACGGGGTCTGACGCTCAGTGGAACGAAAACTCACGTTAAGGGATT
TTGGTCATGAGATTATCAAAAAGGATCTTCACCTAGATCCTTTTAAATTA
AAAATGAAGTTTTAAATCAATCTAAAGTATATATGAGTAAACTTGGTCTG
ACAGTTACCAATGCTTAATCAGTGAGGCACCTATCTCAGCGATCTGTCTA
TTTCGTTCATCCATAGTTGCCTGACTCCCCGTCGTGTAGATAACTACGAT
ACGGGAGGGCTTACCATCTGGCCCCAGTGCTGCAATGATACCGCGAGACC
CACGCTCACCGGCTCCAGATTTATCAGCAATAAACCAGCCAGCCGGAAGG
GCCGAGCGCAGAAGTGGTCCTGCAACTTTATCCGCCTCCATCCAGTCTAT
TAATTGTTGCCGGGAAGCTAGAGTAAGTAGTTCGCCAGTTAATAGTTTGC
GCAACGTTGTTGCCATTGCTACAGGCATCGTGGTGTCACGCTCGTCGTTT
GGTATGGCTTCATTCAGCTCCGGTTCCCAACGATCAAGGCGAGTTACATG
ATCCCCCATGTTGTGCAAAAAAGCGGTTAGCTCCTTCGGTCCTCCGATCG
TTGTCAGAAGTAAGTTGGCCGCAGTGTTATCACTCATGGTTATGGCAGCA
CTGCATAATTCTCTTACTGTCATGCCATCCGTAAGATGCTTTTCTGTGAC
TGGTGAGTACTCAACCAAGTCATTCTGAGAATAGTGTATGCGGCGACCGA
GTTGCTCTTGCCCGGCGTCAATACGGGATAATACCGCGCCACATAGCAGA
ACTTTAAAAGTGCTCATCATTGGAAAACGTTCTTCGGGGCGAAAACTCTC
AAGGATCTTACCGCTGTTGAGATCCAGTTCGATGTAACCCACTCGTGCAC
CCAACTGATCTTCAGCATCTTTTACTTTCACCAGCGTTTCTGGGTGAGCA
AAAACAGGAAGGCAAAATGCCGCAAAAAAGGGAATAAGGGCGACACGGAA
ATGTTGAATACTCATACTCTTCCTTTTTCAATATTATTGAAGCATTTATC
AGGGTTATTGTCTCATGAGCGGATACATATTTGAATGTATTTAGAAAAAT
AAACAAATAGGGGTTCCGCGCACATTTCCCCGAAAAGTGCCACCTGACGC
GCCCTGTAGCGGGATCA

pPI230 – mNeon (C)

ACTAGCGCCGGCATTACATGTCCATTTATTAGAAGGATTCAATCAAAAAT
TAACACCAGGTATATTACCAGATGGCATTAATAATTTATATTCTTTAATA
TTGGAAAAAGAAAAAAATAAATAAACAATCAATCAGAAAAGATAATTGAA
AAAAAAAAAAAAGAAAATGTTTAATAAGAATTAATTTGGAAGTAAAATAA
TTTTTGGATGAGTAAATATTAATAAACTCACAATTAAAATTTAGAATCCA
AAAAAATGAAAAAAAAAAAAAATTTTTAAAACAGAATGGTTGCATTCATTT
TTTTTTTTTTTTTTTTATATCTTTTGGGTATTTTTGGGTGTTCTATTTAT
CTGGATAAAAAATAAAATGGAATATAGTTTTATATATATATATTTTTTTCGT
ATCAAGCTTGAATGTTTTTATCAATAATATGATTGTTGGTTGCTTTATTT
TATTTGAAAAAAAAATTAATTTTTAAAATATCTTTTTTTTCATTTTATTT
GAAAAAAAAAAAAAAAAAAAATGGTTTTGAAAATTTTTAAAAAAATGGTT
GGGTCACCCACAATTCTTATAAAAAAAAAGCCATTTGGATAGTTTTTTTTT
TTTTTTTTTTTATTTTTTTTATTTTTTTTTTTTTTGAGATTTGGTATAAAT
ACAAAAAAAAAAATTGTTTTTAAATCATTATTAATAAAAACTTAAAATAAA
TTATATATAAAAAAAGATCTTCAACTAGTGGTGGTTCAGGAGGTAGTGTG
AGTAAAGGTGAAGAAGATAATATGGCATCGTTACCAGCTACACATGAGTT
ACATATATTCGGTAGCATTAATGGTGTTGATTTTGATATGGTGGGACAAG

GTACCGGTAATCCTAATGATGGTTACGAAGAACTAAATTTAAAATCGACT
AAAGGTGACTTACAATTTTCTCCATGGATTTTAGTGCCACATATAGGGTA
TGGTTTTCATCAATACTTACCATATCCAGATGGTATGTCACCATTTCAAG
CTGCAATGGTTGATGGATCAGGTTATCAAGTTCATAGAACAATGCAATTT
GAAGATGGTGCTTCATTAACTGTTAATTATAGATACACATATGAAGGCTC
ACATATTAAAGGTGAAGCTCAAGTTAAAGGTACTGGTTTCCCAGCCGATG
GCCCAGTTATGACAAATAGTTTAACAGCAGCAGATTGGTGTAGATCCAAA
AAAACTTATCCAAATGATAAAACAATTATTTCAACTTTTAAATGGTCATA
TACAACCGGTAATGGTAAACGTTATCGTTCAACAGCCCGTACAACATATA
CTTTTGCTAAACCAATGGCAGCTAATTATTTAAAAAATCAACCAATGTAT
GTTTTTCGTAAAACAGAGTTAAAACATTCAAAAACAGAACTTAATTTTAA
AGAATGGCAAAAAGCATTTACAGACGTTATGTAAGCTAGTATAAAATAAA
TAAATTGTGGATCTGATATCATAACTTCGTATAATGTATGCTATACGAAG
TTATACTACTCTCGAGATTTTATTTAATATACTAAATAATAAAAAAGTTA
AAAAATGATCATTGGATAAATTTTTTATAATTATAAATAAAGATAATAAT
TTTTTTTTTAACAAAACTAAAAATAAAAATAATAAAATAATTGTTAAAAT
AGGTTTTTTTTTTTTTTTTTTTTTTTTAATAAATGGTATTTATTAATTTA
TTTGTTGTGTGTGTTTTTTTTTTTATAATATTTTTTTTTTTAGCATTGAA
TTAAGAAGAAATCAAATTGATGCGGCCGCTTAGTTAGCCTCCCCCATCTC
CCGATCCGGACGAGTGCTGGGGCGTCGGTTTCCACTATCGGCGAGTACTT
CTACACAGCCATCGGTCCAGACGGCCGCGCTTCTGCGGGCGATTTGTGTA
CGCCCGACAGTCCCGGCTCCGGATCGGACGATTGCGTCGCATCGACCCTG
CGCCCAAGCTGCATCATCGAAATTGCCGTCAACCAAGCTCTGATAGAGTT
GGTCAAGACCAATGCGGAGCATATACGCCCGGAGCCGCGGCGATCCTGCA
AGCTCCGGATGCCTCCGCTCGAAGTAGCGCGTCTGCTGCTCCATACAAGC
CAACCACGGCCTCCAGAAGAAGATGTTGGCGACCTCGTATTGGGAATCCC
CGAACATCGCCTCGCTCCAGTCAATGACCGCTGTTATGCGGCCATTGTCC
GTCAGGACATTGTTGGAGCCGAAATCCGCGTGCACGAGGTGCCGGACTTC
GGGGCAGTCCTCGGCCCAAAGCATCAGCTCATCGAGAGCCTGCGCGACGG
ACGCACTGACGGTGTCGTCCATCACAGTTTGCCAGTGATACACATGGGGA
TCAGCAATCGCGCATATGAAATCACGCCATGTAGTGTATTGACCGATTCC
TTGCGGTCCGAATGGGCCGAACCCGCTCGTCTGGCTAAGATCGGCCGCAG
CGATCGCATCCATGGCCTCCGCGACCGGCTGCAGAACAGCGGGCAGTTCG
GTTTCAGGCAGGTCTTGCAACGTGACACCCTGTGCACGGCGGGAGATGCA
ATAGGTCAGGCTCTCGCTGAACTCCCCAATGTCAAGCACTTCCGGAATCG
GGAGCGCGGCCGATGCAAAGTGCCGATAAACATAACGATCTTTGTAGAAA
CCATCGGCGCAGCTATTTACCCGCAGGACATATCCACGCCCTCCTACATC
GAAGCTGAAAGCACGAGATTCTTCGCCCTCCGAGAGCTGCATCAGGTCGG
AGACGCTGTCGAACTTTTCGATCAGAAACTTCTCGACAGACGTCGCGGTG
AGTTCAGGCTTTTTCATTAGGTCTTGTTGAGAAATGTTAAATTGATCCAT
TTTTTGCTAGCTGTGAAATTAGTTTAAAATACAAATAAAGAGTTATAATA
ATATACAGTTGAATAAAAAAAAAAAAAAATGAATTGGAAAATTTATTTTA
TATGAAGAAAAAAAAAATTTTGAAAAAAAAAAAAAAAAATTAAAAAAAAAAA
AAAAAAAAAAAAAAATTAAAAATAATTCAACTGTGGGTGACCCAAATTTT
ATTTAAAAAAAAAAAAAAAATGGATCACTTTTGGGTGGTTGGAAAAAAAA
AAAAAAAAAAATTGAAAATATAATGTTAGTCATATGATTAATCATTAATT
AATTCTTTTTTTTTTTTTTTTATTTTTTATTTTTTTTTTTTTCAATATTC
CAATTATGACACACACTAACATCACAAAATACAAAATTTTGGGATCCACT
ACTATAACTTCGTATAATGTATGCTATACGAAGTTATTTAATTAATTAAG
ATATCAAGCTTGTCCACAGAAAATGTTTCTAAACAAAAAAAAAAAAACCGA

GTGATGAAAGCGCTTCTCACAAAATTATTTATGTAAAATATTTAATAAAA
TACATTATTTAAATCTTTTTTATTTTTTTTAGTTGTTGATCTTTATCCGA
CTTTAAAAATAAAAAAAATTGTAAAAAAAAAAAAAAGTTTATTTGTTTAATT
TTATTGTTGTTTTTAATTTTTTACAACCATTAATTTATAAAACCAACACC
TAACTATAATGAAATACAATTTGTATCTTGGAAAAAAAAAAAATTAATAAT
ATAAATAAAATGTAAACAAGTATCCGGTGGAAGGAACTAACAGTTGCCTT
ATCCAGTTCAAAAAAAAAAAAAAAAAAAAAGAAATTAAATTACCATTTTATA
TTTAACTAAAATAAATTAAAAGGTTTATTTTAATAATAGATTTATTATTT
TTTAAAAATCAATAATTAATGTTGGATAAGATGAACGAATAAAAGATAGA
GTTTCAGGTCTTTTAAATGATGAGGATTTATGAATTCTTAAAAGTTTTGG
GCAAGAGAAAATGATTTTAACGATTGAACCATCACTAATTGCAAAATCTC
TTGAACTAAGAGTTTTTAAGTTTGAAGCAAGTGGAGAATTACAAAGAGCA
ATCATAGAGTTATCAGTTAAATCATAACAACAAGATAAAGATAAAGAACT
AATATGATGTAAATTTTCAATCATACCTCTAAAAGAAACATCATTCATAC
TTTTTGGATAAATTAAATCTAATGAATCTAAATTTGGAAATAGTAAACCA
ATATTTGGCCGGCAGAGGCGGTTTGCGTATTGGGCGCTCTTCCGCTTCCT
CGCTCACTGACTCGCTGCGCTCGGTCGTTCGGCTGCGGCGAGCGGTATCA
GCTCACTCAAAGGCGGTAATACGGTTATCCACAGAATCAGGGGATAACGC
AGGAAAGAACATGTGAGCAAAAGGCCAGCAAAAGGCCAGGAACCGTAAAA
AGGCCGCGTTGCTGGCGTTTTTCCATAGGCTCCGCCCCCCTGACGAGCAT
CACAAAAATCGACGCTCAAGTCAGAGGTGGCGAAACCCGACAGGACTATA
AAGATACCAGGCGTTTCCCCCTGGAAGCTCCCTCGTGCGCTCTCCTGTTC
CGACCCTGCCGCTTACCGGATACCTGTCCGCCTTTCTCCCTTCGGGAAGC
GTGGCGCTTTCTCATAGCTCACGCTGTAGGTATCTCAGTTCGGTGTAGGT
CGTTCGCTCCAAGCTGGGCTGTGTGCACGAACCCCCCGTTCAGCCCGACC
GCTGCGCCTTATCCGGTAACTATCGTCTTGAGTCCAACCCGGTAAGACAC
GACTTATCGCCACTGGCAGCAGCCACTGGTAACAGGATTAGCAGAGCGAG
GTATGTAGGCGGTGCTACAGAGTTCTTGAAGTGGTGGCCTAACTACGGCT
ACACTAGAAGGACAGTATTTGGTATCTGCGCTCTGCTGAAGCCAGTTACC
TTCGGAAAAAGAGTTGGTAGCTCTTGATCCGGCAAACAAACCACCGCTGG
TAGCGGTGGTTTTTTTGTTTGCAAGCAGCAGATTACGCGCAGAAAAAAAG
GATCTCAAGAAGATCCTTTGATCTTTTCTACGGGGTCTGACGCTCAGTGG
AACGAAAACTCACGTTAAGGGATTTTGGTCATGAGATTATCAAAAAGGAT
CTTCACCTAGATCCTTTTAAATTAAAAATGAAGTTTTAAATCAATCTAAA
GTATATATGAGTAAACTTGGTCTGACAGTTACCAATGCTTAATCAGTGAG
GCACCTATCTCAGCGATCTGTCTATTTCGTTCATCCATAGTTGCCTGACT
CCCCGTCGTGTAGATAACTACGATACGGGAGGGCTTACCATCTGGCCCCA
GTGCTGCAATGATACCGCGAGACCCACGCTCACCGGCTCCAGATTTATCA
GCAATAAACCAGCCAGCCGGAAGGGCCGAGCGCAGAAGTGGTCCTGCAAC
TTTATCCGCCTCCATCCAGTCTATTAATTGTTGCCGGGAAGCTAGAGTAA
GTAGTTCGCCAGTTAATAGTTTGCGCAACGTTGTTGCCATTGCTACAGGC
ATCGTGGTGTCACGCTCGTCGTTTGGTATGGCTTCATTCAGCTCCGGTTC
CCAACGATCAAGGCGAGTTACATGATCCCCCATGTTGTGCAAAAAAGCGG
TTAGCTCCTTCGGTCCTCCGATCGTTGTCAGAAGTAAGTTGGCCGCAGTG
TTATCACTCATGGTTATGGCAGCACTGCATAATTCTCTTACTGTCATGCC
ATCCGTAAGATGCTTTTCTGTGACTGGTGAGTACTCAACCAAGTCATTCT
GAGAATAGTGTATGCGGCGACCGAGTTGCTCTTGCCCGGCGTCAATACGG
GATAATACCGCGCCACATAGCAGAACTTTAAAAGTGCTCATCATTGGAAA
ACGTTCTTCGGGGCGAAAACTCTCAAGGATCTTACCGCTGTTGAGATCCA
GTTCGATGTAACCCACTCGTGCACCCAACTGATCTTCAGCATCTTTTACT

TTCACCAGCGTTTCTGGGTGAGCAAAAACAGGAAGGCAAAATGCCGCAAA
AAAGGGAATAAGGGCGACACGGAAATGTTGAATACTCATACTCTTCCTTT
TTCAATATTATTGAAGCATTTATCAGGGTTATTGTCTCATGAGCGGATAC
ATATTTGAATGTATTTAGAAAAATAAACAAATAGGGGTTCCGCGCACATT
TCCCCGAAAAGTGCCACCTGACGCGCCCTGTAGCGGGATCA

pPI419 – mScarlet (N)

ACTAGCGCCGGCATTACATGTCCATTTATTAGAAGGATTCAATCAAAAAT
TAACACCAGGTATATTACCAGATGGCATTAATAATTTATATTCTTTAATA
TTGGAAAAAGAAAAAAATAAATAAACAATCAATCAGAAAAGATAATTGAA
AAAAAAAAAAAAGAAAATGTTTAATAAGAATTAATTTGGAAGTAAAATAA
TTTTTGGATGAGTAAATATTAATAAACTCACAATTAAAATTTAGAATCCA
AAAAAATGAAAAAAAAAAAAAATTTTTAAAACAGAATGGTTGCATTCATTT
TTTTTTTTTTTTTTTTATATCTTTTGGGTATTTTTGGGTGTTCTATTTAT
CTGGATAAAAAATAAAATGGAATATAGTTTTATATATATATTTTTTTCGT
ATCAAGCTTGAATGTTTTTATCAATAATATGATTGTTGGTTGCTTTATTT
TATTTGAAAAAAAAATTAATTTTTAAAATATCTTTTTTTTCATTTTATTT
GAAAAAAAAAAAAAAAAAAAATGGTTTTGAAAATTTTTAAAAAAATGGTT
GGGTCACCCACAATTCTTATAAAAAAAAGCCATTTGGATAGTTTTTTTTT
TTTTTTTTTTATTTTTTTTATTTTTTTTTTTTTTGAGATTTGGTATAAAT
ACAAAAAAAAAATTGTTTTTAAATCATTATTAATAAAAACTTAAAATAAA
TTATATATAAAAAAAGATCCAAAAAATGGTTTCAAAAGGTGAAGCCGTTA
TTAAAGAATTTATGAGATTCAAGGTTCACATGGAAGGAAGTATGAACGGT
CATGAATTTGAGATTGAAGGAGAAGGTGAAGGTAGACCATATGAAGGCAC
CCAAACAGCTAAATTAAAAGTAACTAAAGGTGGTCCATTACCATTTAGTT
GGGATATTTTATCTCCACAATTTATGTATGGTTCACGTGCTTTCACAAAA
CATCCAGCAGATATTCCAGATTATTATAAACAATCATTTCCAGAAGGTTT
TAAATGGGAACGTGTCATGAACTTTGAAGATGGTGGAGCAGTTACAGTCA
CACAAGATACCTCATTAGAAGATGGTACATTAATATATAAAGTTAAATTA
CGTGGTACTAATTTTCCACCAGACGGTCCAGTAATGCAAAAAAAAACAAT
GGGCTGGGAAGCTAGTACAGAACGTTTATATCCTGAAGATGGTGTCCTTA
AAGGCGATATAAAAATGGCCTTGAGATTAAAGGATGGTGGTAGGTATTTA
GCAGATTTCAAAACCACTTATAAAGCAAAAAAACCAGTTCAAATGCCAGG
TGCATATAATGTTGATAGAAAACTTGATATTACCAGTCATAATGAAGATT
ACACAGTTGTCGAACAATACGAACGTTCTGAAGGTCGTCATAGCACTGGT
GGTATGGATGAATTATACAAAGGTGGTTCAGGAGGTAGTAGATCTTCAAC
TAGTATAAAATAAATAAATTGTGGATCTGATATCATAACTTCGTATAATG
TATGCTATACGAAGTTATACTACTCTCGAGATTTTATTTAATATACTAAA
TAATAAAAAAGTTAAAAAATGATCATTGGATAAATTTTTTATAATTATAA
ATAAAGATAATAATTTTTTTTTTAACAAAACTAAAAATAAAAATAATAAA
ATAATTGTTAAAATAGGTTTTTTTTTTTTTTTTTTTTTTTTAATAAATGG
TATTTATTAATTTATTTGTTGTGTGTGTTTTTTTTTTTTATAATATTTTTT
TTTTTAGCATTGAATTAAGAAGAAATCAAATTGATGCGGCCGCTTAGTTA
GCCTCCCCCATCTCCCGATCCGGACGAGTGCTGGGGCGTCGGTTTCCACT
ATCGGCGAGTACTTCTACACAGCCATCGGTCCAGACGGCCGCGCTTCTGC
GGGCGATTTGTGTACGCCCGACAGTCCCGGCTCCGGATCGGACGATTGCG
TCGCATCGACCCTGCGCCCAAGCTGCATCATCGAAATTGCCGTCAACCAA
GCTCTGATAGAGTTGGTCAAGACCAATGCGGAGCATATACGCCCGGAGCC
GCGGCGATCCTGCAAGCTCCGGATGCCTCCGCTCGAAGTAGCGCGTCTGC

TGCTCCATACAAGCCAACCACGGCCTCCAGAAGAAGATGTTGGCGACCTC
GTATTGGGAATCCCCGAACATCGCCTCGCTCCAGTCAATGACCGCTGTTA
TGCGGCCATTGTCCGTCAGGACATTGTTGGAGCCGAAATCCGCGTGCACG
AGGTGCCGGACTTCGGGGCAGTCCTCGGCCCAAAGCATCAGCTCATCGAG
AGCCTGCGCGACGGACGCACTGACGGTGTCGTCCATCACAGTTTGCCAGT
GATACACATGGGGATCAGCAATCGCGCATATGAAATCACGCCATGTAGTG
TATTGACCGATTCCTTGCGGTCCGAATGGGCCGAACCCGCTCGTCTGGCT
AAGATCGGCCGCAGCGATCGCATCCATGGCCTCCGCGACCGGCTGCAGAA
CAGCGGGCAGTTCGGTTTCAGGCAGGTCTTGCAACGTGACACCCTGTGCA
CGGCGGGAGATGCAATAGGTCAGGCTCTCGCTGAACTCCCCAATGTCAAG
CACTTCCGGAATCGGGAGCGCGGCCGATGCAAAGTGCCGATAAACATAAC
GATCTTTGTAGAAACCATCGGCGCAGCTATTTACCCGCAGGACATATCCA
CGCCCTCCTACATCGAAGCTGAAAGCACGAGATTCTTCGCCCTCCGAGAG
CTGCATCAGGTCGGAGACGCTGTCGAACTTTTCGATCAGAAACTTCTCGA
CAGACGTCGCGGTGAGTTCAGGCTTTTTCATTAGGTCTTGTTGAGAAATG
TTAAATTGATCCATTTTTTGCTAGCTGTGAAATTAGTTTAAAATACAAAT
AAAGAGTTATAATAATATACAGTTGAATAAAAAAAAAAAAAATGAATTGG
AAAATTTATTTTTATATGAAGAAAAAAAAATTTTGAAAAAAAAAAAAAAA
ATTAAAAAAAAAAAAAAAAAAAAAAAAAATTAAAAATAATTCAACTGTGG
GTGACCCAAATTTATTTAAAAAAAAAAAAAAAAATGGATCACTTTTGGGT
GGTTGGAAAAAAAAAAAAAAAAAAATTGAAAATATAATGTTAGTCATATG
ATTAATCATTAATTAATTCTTTTTTTTTTTTTTTTATTTTTTTATTTTTT
TTTTTTCAATATTCCAATTATGACACACACTAACATCACAAAATACAAAA
TTTTGGGATCCACTACTATAACTTCGTATAATGTATGCTATACGAAGTTA
TTTAATTAATTAAGATATCAAGCTTGTCCACAGAAAATGTTTCTAAACAA
AAAAAAAAAACCGAGTGATGAAAGCGCTTCTCACAAAATTATTTATGTAA
AATATTTAATAAAATACATTATTTAAATCTTTTTTATTTTTTTTAGTTGT
TGATCTTTATCCGACTTTAAAAATAAAAAAATTGTAAAAAAAAAAAAAGT
TTATTTGTTTAATTTTATTGTTGTTTTTAATTTTTTACAACCATTAATTT
ATAAAACCAACACCTAACTATAATGAAATACAATTTGTATCTTGGAAAAA
AAAAAATTAATAATATAAATAAAATGTAAACAAGTATCCGGTGGAAGGAA
CTAACAGTTGCCTTATCCAGTTCAAAAAAAAAAAAAAAAAAAGAAATTAA
ATTACCATTTTATATTTAACTAAAATAAATTAAAAGGTTTATTTTAATAA
TAGATTTATTATTTTTTAAAAATCAATAATTAATGTTGGATAAGATGAAC
GAATAAAAGATAGAGTTTCAGGTCTTTTAAATGATGAGGATTTATGAATT
CTTAAAAGTTTTGGGCAAGAGAAAATGATTTTAACGATTGAACCATCACT
AATTGCAAAATCTCTTGAACTAAGAGTTTTTAAGTTTGAAGCAAGTGGAG
AATTACAAAGAGCAATCATAGAGTTATCAGTTAAATCATAACAACAAGAT
AAAGATAAAGAACTAATATGATGTAAATTTTCAATCATACCTCTAAAAGA
AACATCATTCATACTTTTTGGATAAATTAAATCTAATGAATCTAAATTTG
GAAATAGTAAACCAATATTTGGCCGGCAGAGGCGGTTTGCGTATTGGGCG
CTCTTCCGCTTCCTCGCTCACTGACTCGCTGCGCTCGGTCGTTCGGCTGC
GGCGAGCGGTATCAGCTCACTCAAAGGCGGTAATACGGTTATCCACAGAA
TCAGGGGATAACGCAGGAAAGAACATGTGAGCAAAAGGCCAGCAAAAGGC
CAGGAACCGTAAAAAGGCCGCGTTGCTGGCGTTTTTCCATAGGCTCCGCC
CCCCTGACGAGCATCACAAAAATCGACGCTCAAGTCAGAGGTGGCGAAAC
CCGACAGGACTATAAAGATACCAGGCGTTTCCCCCTGGAAGCTCCCTCGT
GCGCTCTCCTGTTCCGACCCTGCCGCTTACCGGATACCTGTCCGCCTTTC
TCCCTTCGGGAAGCGTGGCGCTTTCTCATAGCTCACGCTGTAGGTATCTC
AGTTCGGTGTAGGTCGTTCGCTCCAAGCTGGGCTGTGTGCACGAACCCCC

CGTTCAGCCCGACCGCTGCGCCTTATCCGGTAACTATCGTCTTGAGTCCA
ACCCGGTAAGACACGACTTATCGCCACTGGCAGCAGCCACTGGTAACAGG
ATTAGCAGAGCGAGGTATGTAGGCGGTGCTACAGAGTTCTTGAAGTGGTG
GCCTAACTACGGCTACACTAGAAGGACAGTATTTGGTATCTGCGCTCTGC
TGAAGCCAGTTACCTTCGGAAAAAGAGTTGGTAGCTCTTGATCCGGCAAA
CAAACCACCGCTGGTAGCGGTGGTTTTTTTGTTTGCAAGCAGCAGATTAC
GCGCAGAAAAAAAGGATCTCAAGAAGATCCTTTGATCTTTTCTACGGGGT
CTGACGCTCAGTGGAACGAAAACTCACGTTAAGGGATTTTGGTCATGAGA
TTATCAAAAAGGATCTTCACCTAGATCCTTTTAAATTAAAAATGAAGTTT
TAAATCAATCTAAAGTATATATGAGTAAACTTGGTCTGACAGTTACCAAT
GCTTAATCAGTGAGGCACCTATCTCAGCGATCTGTCTATTTCGTTCATCC
ATAGTTGCCTGACTCCCCGTCGTGTAGATAACTACGATACGGGAGGGCTT
ACCATCTGGCCCCAGTGCTGCAATGATACCGCGAGACCCACGCTCACCGG
CTCCAGATTTATCAGCAATAAACCAGCCAGCCGGAAGGGCCGAGCGCAGA
AGTGGTCCTGCAACTTTATCCGCCTCCATCCAGTCTATTAATTGTTGCCG
GGAAGCTAGAGTAAGTAGTTCGCCAGTTAATAGTTTGCGCAACGTTGTTG
CCATTGCTACAGGCATCGTGGTGTCACGCTCGTCGTTTGGTATGGCTTCA
TTCAGCTCCGGTTCCCAACGATCAAGGCGAGTTACATGATCCCCCATGTT
GTGCAAAAAAGCGGTTAGCTCCTTCGGTCCTCCGATCGTTGTCAGAAGTA
AGTTGGCCGCAGTGTTATCACTCATGGTTATGGCAGCACTGCATAATTCT
CTTACTGTCATGCCATCCGTAAGATGCTTTTCTGTGACTGGTGAGTACTC
AACCAAGTCATTCTGAGAATAGTGTATGCGGCGACCGAGTTGCTCTTGCC
CGGCGTCAATACGGGATAATACCGCGCCACATAGCAGAACTTTAAAAGTG
CTCATCATTGGAAAACGTTCTTCGGGGCGAAAACTCTCAAGGATCTTACC
GCTGTTGAGATCCAGTTCGATGTAACCCACTCGTGCACCCAACTGATCTT
CAGCATCTTTTACTTTCACCAGCGTTTCTGGGTGAGCAAAAACAGGAAGG
CAAAATGCCGCAAAAAAGGGAATAAGGGCGACACGGAAATGTTGAATACT
CATACTCTTCCTTTTTCAATATTATTGAAGCATTTATCAGGGTTATTGTC
TCATGAGCGGATACATATTTGAATGTATTTAGAAAAATAAACAAATAGGG
GTTCCGCGCACATTTCCCCGAAAAGTGCCACCTGACGCGCCCTGTAGCGG
GATCA

pPI458 – mScarlet (C)

ACTAGCGCCGGCATTACATGTCCATTTATTAGAAGGATTCAATCAAAAAT
TAACACCAGGTATATTACCAGATGGCATTAATAATTTATATTCTTTAATA
TTGGAAAAAGAAAAAAATAAATAAACAATCAATCAGAAAAGATAATTGAA
AAAAAAAAAAAAGAAAATGTTTAATAAGAATTAATTTGGAAGTAAAATAA
TTTTTGGATGAGTAAATATTAATAAACTCACAATTAAAATTTAGAATCCA
AAAAAATGAAAAAAAAAAAAAATTTTTAAAACAGAATGGTTGCATTCATTT
TTTTTTTTTTTTTTTATATCTTTTGGGTATTTTTGGGTGTTCTATTTAT
CTGGATAAAAAATAAAATGGAATATAGTTTATATATATATATTTTTTTCGT
ATCAAGCTTGAATGTTTTTATCAATAATATGATTGTTGGTTGCTTTATTT
TATTTGAAAAAAAAATTAATTTTTAAAATATCTTTTTTTTCATTTTATTT
GAAAAAAAAAAAAAAAAAAAAATGGTTTTGAAAATTTTTAAAAAAATGGTT
GGGTCACCCACAATTCTTATAAAAAAAAAGCCATTTGGATAGTTTTTTTTT
TTTTTTTTTTTATTTTTTTTATTTTTTTTTTTTTTGAGATTTGGTATAAAT
ACAAAAAAAAAAATTGTTTTTAAATCATTATTAATAAAAACTTAAAATAAA
TTATATATAAAAAAAGATCTTCAACTAGTGGTGGTTCAGGAGGTAGTGTT
TCAAAAGGTGAAGCCGTTATTAAAGAATTTATGAGATTCAAGGTTCACAT

GGAAGGAAGTATGAACGGTCATGAATTTGAGATTGAAGGAGAAGGTGAAG
GTAGACCATATGAAGGCACCCAAACAGCTAAATTAAAAGTAACTAAAGGT
GGTCCATTACCATTTAGTTGGGATATTTTATCTCCACAATTTATGTATGG
TTCACGTGCTTTCACAAAACATCCAGCAGATATTCCAGATTATTATAAAC
AATCATTTCCAGAAGGTTTTAAATGGGAACGTGTCATGAACTTTGAAGAT
GGTGGAGCAGTTACAGTCACACAAGATACCTCATTAGAAGATGGTACATT
AATATATAAAGTTAAATTACGTGGTACTAATTTTCCACCAGACGGTCCAG
TAATGCAAAAAAAAACAATGGGCTGGGAAGCTAGTACAGAACGTTTATAT
CCTGAAGATGGTGTCCTTAAAGGCGATATAAAAATGGCCTTGAGATTAAA
GGATGGTGGTAGGTATTTAGCAGATTTCAAAACCACTTATAAAGCAAAAA
AACCAGTTCAAATGCCAGGTGCATATAATGTTGATAGAAAACTTGATATT
ACCAGTCATAATGAAGATTACACAGTTGTCGAACAATACGAACGTTCTGA
AGGTCGTCATAGCACTGGTGGTATGGATGAATTATACAAATAAGCTAGTA
TAAAATAAATAAATTGTGGATCTGATATCATAACTTCGTATAATGTATGC
TATACGAAGTTATACTACTCTCGAGATTTTATTTAATATACTAAATAATA
AAAAAGTTAAAAAATGATCATTGGATAAATTTTTTATAATTATAAATAAA
GATAATAATTTTTTTTTTAACAAAACTAAAAATAAAAATAATAAAATAAT
TGTTAAAATAGGTTTTTTTTTTTTTTTTTTTTTTTTAATAAATGGTATTT
ATTAATTTATTTGTTGTGTGTGTTTTTTTTTTTTATAATATTTTTTTTTT
AGCATTGAATTAAGAAGAAATCAAATTGATGCGGCCGCTTAGTTAGCCTC
CCCCATCTCCCGATCCGGACGAGTGCTGGGGCGTCGGTTTCCACTATCGG
CGAGTACTTCTACACAGCCATCGGTCCAGACGGCCGCGCTTCTGCGGGCG
ATTTGTGTACGCCCGACAGTCCCGGCTCCGGATCGGACGATTGCGTCGCA
TCGACCCTGCGCCCAAGCTGCATCATCGAAATTGCCGTCAACCAAGCTCT
GATAGAGTTGGTCAAGACCAATGCGGAGCATATACGCCCGGAGCCGCGGC
GATCCTGCAAGCTCCGGATGCCTCCGCTCGAAGTAGCGCGTCTGCTGCTC
CATACAAGCCAACCACGGCCTCCAGAAGAAGATGTTGGCGACCTCGTATT
GGGAATCCCCGAACATCGCCTCGCTCCAGTCAATGACCGCTGTTATGCGG
CCATTGTCCGTCAGGACATTGTTGGAGCCGAAATCCGCGTGCACGAGGTG
CCGGACTTCGGGGCAGTCCTCGGCCCAAAGCATCAGCTCATCGAGAGCCT
GCGCGACGGACGCACTGACGGTGTCGTCCATCACAGTTTGCCAGTGATAC
ACATGGGGATCAGCAATCGCGCATATGAAATCACGCCATGTAGTGTATTG
ACCGATTCCTTGCGGTCCGAATGGGCCGAACCCGCTCGTCTGGCTAAGAT
CGGCCGCAGCGATCGCATCCATGGCCTCCGCGACCGGCTGCAGAACAGCG
GGCAGTTCGGTTTCAGGCAGGTCTTGCAACGTGACACCCTGTGCACGGCG
GGAGATGCAATAGGTCAGGCTCTCGCTGAACTCCCCAATGTCAAGCACTT
CCGGAATCGGGAGCGCGGCCGATGCAAAGTGCCGATAAACATAACGATCT
TTGTAGAAACCATCGGCGCAGCTATTTACCCGCAGGACATATCCACGCCC
TCCTACATCGAAGCTGAAAGCACGAGATTCTTCGCCCTCCGAGAGCTGCA
TCAGGTCGGAGACGCTGTCGAACTTTTCGATCAGAAACTTCTCGACAGAC
GTCGCGGTGAGTTCAGGCTTTTTCATTAGGTCTTGTTGAGAAATGTTAAA
TTGATCCATTTTTTGCTAGCTGTGAAATTAGTTTAAAATACAAATAAAGA
GTTATAATAATATACAGTTGAATAAAAAAAAAAAAAATGAATTGGAAAAT
TTATTTTTATATGAAGAAAAAAAAAAATTTTGAAAAAAAAAAAAAATTAA
AAAAAAAAAAAAAAAAAAAAAAAATTAAAAATAATTCAACTGTGGGTGAC
CCAAATTTTATTTAAAAAAAAAAAAAAAATGGATCACTTTTGGGTGGTTG
GAAAAAAAAAAAAAAAAAAATTGAAAATATAATGTTAGTCATATGATTAA
TCATTAATTAATTCTTTTTTTTTTTTTTTTTATTTTTTATTTTTTTTTTT
TCAATATTCCAATTATGACACACACTAACATCACAAAATACAAAATTTTG
GGATCCACTACTATAACTTCGTATAATGTATGCTATACGAAGTTATTTAA

TTAATTAAGATATCAAGCTTGTCCACAGAAAATGTTTCTAAACAAAAAAA
AAAAACCGAGTGATGAAAGCGCTTCTCACAAAATTATTTATGTAAAATAT
TTAATAAAATACATTATTTAAATCTTTTTTATTTTTTTTAGTTGTTGATC
TTTATCCGACTTTAAAAATAAAAAAATTGTAAAAAAAAAAAAAAGTTTATT
TGTTTAATTTTATTGTTGTTTTTAATTTTTTACAACCATTAATTTATAAA
ACCAACACCTAACTATAATGAAATACAATTTGTATCTTGGAAAAAAAAAA
ATTAATAATATAAATAAAATGTAAACAAGTATCCGGTGGAAGGAACTAAC
AGTTGCCTTATCCAGTTCAAAAAAAAAAAAAAAAAAAAGAAATTAAATTAC
CATTTTATATTTAACTAAAATAAATTAAAAGGTTTATTTTAATAATAGAT
TTATTATTTTTTAAAAATCAATAATTAATGTTGGATAAGATGAACGAATA
AAAGATAGAGTTTCAGGTCTTTTAAATGATGAGGATTTATGAATTCTTAA
AAGTTTTGGGCAAGAGAAAATGATTTTAACGATTGAACCATCACTAATTG
CAAAATCTCTTGAACTAAGAGTTTTTAAGTTTGAAGCAAGTGGAGAATTA
CAAAGAGCAATCATAGAGTTATCAGTTAAATCATAACAACAAGATAAAGA
TAAAGAACTAATATGATGTAAATTTTCAATCATACCTCTAAAAGAAACAT
CATTCATACTTTTTGGATAAATTAAATCTAATGAATCTAAATTTGGAAAT
AGTAAACCAATATTTGGCCGGCAGAGGCGGTTTGCGTATTGGGCGCTCTT
CCGCTTCCTCGCTCACTGACTCGCTGCGCTCGGTCGTTCGGCTGCGGCGA
GCGGTATCAGCTCACTCAAAGGCGGTAATACGGTTATCCACAGAATCAGG
GGATAACGCAGGAAAGAACATGTGAGCAAAAGGCCAGCAAAAGGCCAGGA
ACCGTAAAAAGGCCGCGTTGCTGGCGTTTTTCCATAGGCTCCGCCCCCCT
GACGAGCATCACAAAAATCGACGCTCAAGTCAGAGGTGGCGAAACCCGAC
AGGACTATAAAGATACCAGGCGTTTCCCCCTGGAAGCTCCCTCGTGCGCT
CTCCTGTTCCGACCCTGCCGCTTACCGGATACCTGTCCGCCTTTCTCCCT
TCGGGAAGCGTGGCGCTTTCTCATAGCTCACGCTGTAGGTATCTCAGTTC
GGTGTAGGTCGTTCGCTCCAAGCTGGGCTGTGTGCACGAACCCCCCGTTC
AGCCCGACCGCTGCGCCTTATCCGGTAACTATCGTCTTGAGTCCAACCCG
GTAAGACACGACTTATCGCCACTGGCAGCAGCCACTGGTAACAGGATTAG
CAGAGCGAGGTATGTAGGCGGTGCTACAGAGTTCTTGAAGTGGTGGCCTA
ACTACGGCTACACTAGAAGGACAGTATTTGGTATCTGCGCTCTGCTGAAG
CCAGTTACCTTCGGAAAAAGAGTTGGTAGCTCTTGATCCGGCAAACAAAC
CACCGCTGGTAGCGGTGGTTTTTTTGTTTGCAAGCAGCAGATTACGCGCA
GAAAAAAAGGATCTCAAGAAGATCCTTTGATCTTTTCTACGGGGTCTGAC
GCTCAGTGGAACGAAAACTCACGTTAAGGGATTTTGGTCATGAGATTATC
AAAAAGGATCTTCACCTAGATCCTTTTAAATTAAAAATGAAGTTTTAAAT
CAATCTAAAGTATATATGAGTAAACTTGGTCTGACAGTTACCAATGCTTA
ATCAGTGAGGCACCTATCTCAGCGATCTGTCTATTTCGTTCATCCATAGT
TGCCTGACTCCCCGTCGTGTAGATAACTACGATACGGGAGGGCTTACCAT
CTGGCCCCAGTGCTGCAATGATACCGCGAGACCCACGCTCACCGGCTCCA
GATTTATCAGCAATAAACCAGCCAGCCGGAAGGGCCGAGCGCAGAAGTGG
TCCTGCAACTTTATCCGCCTCCATCCAGTCTATTAATTGTTGCCGGGAAG
CTAGAGTAAGTAGTTCGCCAGTTAATAGTTTGCGCAACGTTGTTGCCATT
GCTACAGGCATCGTGGTGTCACGCTCGTCGTTTGGTATGGCTTCATTCAG
CTCCGGTTCCCAACGATCAAGGCGAGTTACATGATCCCCCATGTTGTGCA
AAAAAGCGGTTAGCTCCTTCGGTCCTCCGATCGTTGTCAGAAGTAAGTTG
GCCGCAGTGTTATCACTCATGGTTATGGCAGCACTGCATAATTCTCTTAC
TGTCATGCCATCCGTAAGATGCTTTTCTGTGACTGGTGAGTACTCAACCA
AGTCATTCTGAGAATAGTGTATGCGGCGACCGAGTTGCTCTTGCCCGGCG
TCAATACGGGATAATACCGCGCCACATAGCAGAACTTTAAAAGTGCTCAT
CATTGGAAAACGTTCTTCGGGGCGAAAACTCTCAAGGATCTTACCGCTGT

TGAGATCCAGTTCGATGTAACCCACTCGTGCACCCAACTGATCTTCAGCA
TCTTTTACTTTCACCAGCGTTTCTGGGTGAGCAAAAACAGGAAGGCAAAA
TGCCGCAAAAAAGGGAATAAGGGCGACACGGAAATGTTGAATACTCATAC
TCTTCCTTTTTCAATATTATTGAAGCATTTATCAGGGTTATTGTCTCATG
AGCGGATACATATTTGAATGTATTTAGAAAAATAAACAAATAGGGGTTCC
GCGCACATTTCCCCGAAAAGTGCCACCTGACGCGCCCTGTAGCGGGATCA

targeted in-frame vectors

pDM1355 – GFP (C)

ACTAGTGGTGGTTCAGGAGGTAGTTCAAAAGGTGAAGAATTATTTACAGG
TGTTGTTCCAATTTTAGTTGAATTAGATGGTGATGTTAATGGTCATAAAT
TTTCAGTTTCAGGTGAAGGTGAAGGTGATGCAACATATGGTAAATTAACA
TTAAAATTTATTTGTACAACAGGTAAATTACCAGTTCCATGGCCAACATT
AGTTACAACATTTACATATGGTGTTCAATGTTTTTCAAGATATCCAGATC
ATATGAAACAACATGATTTTTTTAAATCAGCAATGCCAGAAGGTTATGTT
CAAGAAAGAACAATTTTTTTTAAAGATGATGGTAATTATAAAACAAGAGC
AGAAGTTAAATTTGAAGGTGATACATTAGTTAATAGAATTGAATTAAAAG
GTATTGATTTTAAAGAAGATGGTAATATTTTAGGTCATAAATTAGAATAT
AATTATAATTCACATAATGTTTATATTATGGCAGATAAACAAAAAAATGG
TATTAAAGTTAATTTTAAAATTAGACATAATATTGAAGATGGTTCAGTTC
AATTAGCAGATCATTATCAACAAAATACACCAATTGGTGATGGTCCAGTT
TTATTACCAGATAATCATTATTTATCAACACAATCAGCATTATCAAAAGA
TCCAAATGAAAAAAGAGATCATATGGTTTTATTAGAATTTGTTACAGCAG
CAGGTATTACACATGGTATGGATGAATTATATAAATAAATAAAATAAATA
AATTGTAGATCTGATATCATAACTTCGTATAATGTATGCTATACGAAGTT
ATACTACTCTCGAGATTTTATTTAATATACTAAATAATAAAAAAGTTAAA
AAATGATCATTGGATAAATTTTTTATAATTATAAATAAAGATAATAATTT
TTTTTTTAACAAAACTAAAAATAAAAATAATAAAATAATTGTTAAAATAG
GTTTTTTTTTTTTTTTTTTTTTTTTTAATAAATGGTATTTATTAATTTATT
TGTTGTGTGTGTTTTTTTTTTTATAATATTTTTTTTTTTAGCATTGAATT
AAGAAGAAATCAAATTGATGCGGCCGCTTAGTTAGCCTCCCCCATCTCCC
GATCCGGACGAGTGCTGGGGCGTCGGTTTCCACTATCGGCGAGTACTTCT
ACACAGCCATCGGTCCAGACGGCCGCGCTTCTGCGGGCGATTTGTGTACG
CCCGACAGTCCCGGCTCCGGATCGGACGATTGCGTCGCATCGACCCTGCG
CCCAAGCTGCATCATCGAAATTGCCGTCAACCAAGCTCTGATAGAGTTGG
TCAAGACCAATGCGGAGCATATACGCCCGGAGCCGCGGCGATCCTGCAAG
CTCCGGATGCCTCCGCTCGAAGTAGCGCGTCTGCTGCTCCATACAAGCCA
ACCACGGCCTCCAGAAGAAGATGTTGGCGACCTCGTATTGGGAATCCCCG
AACATCGCCTCGCTCCAGTCAATGACCGCTGTTATGCGGCCATTGTCCGT
CAGGACATTGTTGGAGCCGAAATCCGCGTGCACGAGGTGCCGGACTTCGG
GGCAGTCCTCGGCCCAAAGCATCAGCTCATCGAGAGCCTGCGCGACGGAC
GCACTGACGGTGTCGTCCATCACAGTTTGCCAGTGATACACATGGGGATC
AGCAATCGCGCATATGAAATCACGCCATGTAGTGTATTGACCGATTCCTT
GCGGTCCGAATGGGCCGAACCCGCTCGTCTGGCTAAGATCGGCCGCAGCG
ATCGCATCCATGGCCTCCGCGACCGGCTGCAGAACAGCGGGCAGTTCGGT
TTCAGGCAGGTCTTGCAACGTGACACCCTGTGCACGGCGGGAGATGCAAT
AGGTCAGGCTCTCGCTGAACTCCCCAATGTCAAGCACTTCCGGAATCGGG
AGCGCGGCCGATGCAAAGTGCCGATAAACATAACGATCTTTGTAGAAACC

ATCGGCGCAGCTATTTACCCGCAGGACATATCCACGCCCTCCTACATCGA
AGCTGAAAGCACGAGATTCTTCGCCCTCCGAGAGCTGCATCAGGTCGGAG
ACGCTGTCGAACTTTTCGATCAGAAACTTCTCGACAGACGTCGCGGTGAG
TTCAGGCTTTTTCATTAGGTCTTGTTGAGAAATGTTAAATTGATCCATTT
TTTGCTAGCTGTGAAATTAGTTTAAAATACAAATAAAGAGTTATAATAAT
ATACAGTTGAATAAAAAAAAAAAAAATGAATTGGAAAATTTATTTTTATA
TGAAGAAAAAAAAATTTTGAAAAAAAAAAAAAAAATTAAAAAAAAAAAAA
AAAAAAAAAAAAATTAAAAATAATTCAACTGTGGGTGACCCAAATTTTAT
TTAAAAAAAAAAAAAAAATGGATCACTTTTGGGTGGTTGGAAAAAAAAAA
AAAAAAAAATTGAAAATATAATGTTAGTCATATGATTAATCATTAATTAA
TTCTTTTTTTTTTTTTTTTTATTTTTTATTTTTTTTTTTTCAATATTCCA
ATTATGACACACACTAACATCACAAAATACAAAATTTTGGGATCCACTAC
TATAACTTCGTATAATGTATGCTATACGAAGTTATTTAATTAATTAAGAT
ATCAAGCTTGATAGCTCTGCCTACTGAAGGCCGGCAGAGGCGGTTTGCGT
ATTGGGCGCTCTTCCGCTTCCTCGCTCACTGACTCGCTGCGCTCGGTCGT
TCGGCTGCGGCGAGCGGTATCAGCTCACTCAAAGGCGGTAATACGGTTAT
CCACAGAATCAGGGGATAACGCAGGAAAGAACATGTGAGCAAAAGGCCAG
CAAAAGGCCAGGAACCGTAAAAAGGCCGCGTTGCTGGCGTTTTTCCATAG
GCTCCGCCCCCCTGACGAGCATCACAAAAATCGACGCTCAAGTCAGAGGT
GGCGAAACCCGACAGGACTATAAAGATACCAGGCGTTTCCCCCTGGAAGC
TCCCTCGTGCGCTCTCCTGTTCCGACCCTGCCGCTTACCGGATACCTGTC
CGCCTTTCTCCCTTCGGGAAGCGTGGCGCTTTCTCATAGCTCACGCTGTA
GGTATCTCAGTTCGGTGTAGGTCGTTCGCTCCAAGCTGGGCTGTGTGCAC
GAACCCCCCGTTCAGCCCGACCGCTGCGCCTTATCCGGTAACTATCGTCT
TGAGTCCAACCCGGTAAGACACGACTTATCGCCACTGGCAGCAGCCACTG
GTAACAGGATTAGCAGAGCGAGGTATGTAGGCGGTGCTACAGAGTTCTTG
AAGTGGTGGCCTAACTACGGCTACACTAGAAGGACAGTATTTGGTATCTG
CGCTCTGCTGAAGCCAGTTACCTTCGGAAAAAGAGTTGGTAGCTCTTGAT
CCGGCAAACAAACCACCGCTGGTAGCGGTGGTTTTTTTGTTTGCAAGCAG
CAGATTACGCGCAGAAAAAAAGGATCTCAAGAAGATCCTTTGATCTTTTC
TACGGGGTCTGACGCTCAGTGGAACGAAAACTCACGTTAAGGGATTTTGG
TCATGAGATTATCAAAAAGGATCTTCACCTAGATCCTTTTAAATTAAAAA
TGAAGTTTTAAATCAATCTAAAGTATATATGAGTAAACTTGGTCTGACAG
TTACCAATGCTTAATCAGTGAGGCACCTATCTCAGCGATCTGTCTATTTC
GTTCATCCATAGTTGCCTGACTCCCCGTCGTGTAGATAACTACGATACGG
GAGGGCTTACCATCTGGCCCCAGTGCTGCAATGATACCGCGAGACCCACG
CTCACCGGCTCCAGATTTATCAGCAATAAACCAGCCAGCCGGAAGGGCCG
AGCGCAGAAGTGGTCCTGCAACTTTATCCGCCTCCATCCAGTCTATTAAT
TGTTGCCGGGAAGCTAGAGTAAGTAGTTCGCCAGTTAATAGTTTGCGCAA
CGTTGTTGCCATTGCTACAGGCATCGTGGTGTCACGCTCGTCGTTTGGTA
TGGCTTCATTCAGCTCCGGTTCCCAACGATCAAGGCGAGTTACATGATCC
CCCATGTTGTGCAAAAAAGCGGTTAGCTCCTTCGGTCCTCCGATCGTTGT
CAGAAGTAAGTTGGCCGCAGTGTTATCACTCATGGTTATGGCAGCACTGC
ATAATTCTCTTACTGTCATGCCATCCGTAAGATGCTTTTCTGTGACTGGT
GAGTACTCAACCAAGTCATTCTGAGAATAGTGTATGCGGCGACCGAGTTG
CTCTTGCCCGGCGTCAATACGGGATAATACCGCGCCACATAGCAGAACTT
TAAAAGTGCTCATCATTGGAAAACGTTCTTCGGGGCGAAAACTCTCAAGG
ATCTTACCGCTGTTGAGATCCAGTTCGATGTAACCCACTCGTGCACCCAA
CTGATCTTCAGCATCTTTTACTTTCACCAGCGTTTCTGGGTGAGCAAAAA
CAGGAAGGCAAAATGCCGCAAAAAAGGGAATAAGGGCGACACGGAAATGT

TGAATACTCATACTCTTCCTTTTTCAATATTATTGAAGCATTTATCAGGG
TTATTGTCTCATGAGCGGATACATATTTGAATGTATTTAGAAAAATAAAC
AAATAGGGGTTCCGCGCACATTTCCCCGAAAAGTGCCACCTGACGCGCCC
TGTAGCGGGATCA

pPI461 – mCherry (C)

ACTAGTGGTGGTTCAGGAGGTAGTGTTTCAAAAGGTGAAGAAGATAATAT
GGCAATTATTAAAGAATTTATGAGATTTAAAGTTCATATGGAAGGTTCAG
TTAATGGTCATGAATTTGAAATTGAAGGTGAAGGTGAAGGTAGACCATAT
GAAGGTACACAAACAGCAAAATTAAAAGTTACAAAAGGTGGTCCATTACC
ATTTGCATGGGATATTTTATCACCACAATTTATGTATGGTTCAAAAGCAT
ATGTTAAACATCCAGCAGATATTCCAGATTATTTAAAATTATCATTTCCA
GAAGGTTTTAAATGGGAAAGAGTTATGAATTTTGAAGATGGTGGTGTTGT
TACAGTTACACAAGATTCATCATTACAAGATGGTGAATTTATTTATAAAG
TTAAATTAAGAGGTACAAATTTTCCATCAGATGGTCCAGTTATGCAAAAG
AAAACAATGGGTTGGGAAGCATCATCAGAAAGAATGTATCCAGAAGATGG
TGCATTAAAAGGTGAAATTAAACAAAGATTAAAATTAAAAGATGGTGGTC
ATTATGATGCAGAAGTTAAAACAACATATAAAGCAAAAAAACCAGTTCAA
TTACCAGGTGCATATAATGTTAATATTAAATTAGATATTACATCACATAA
TGAAGATTATACAATTGTTGAACAATATGAAAGAGCAGAAGGTAGACATT
CAACAGGTGGTATGGATGAATTATATAAATAAAGATCTGATATCATAACT
TCGTATAATGTATGCTATACGAAGTTATACTACTCTCGAGATTTTATTTA
ATATACTAAATAATAAAAAAGTTAAAAAATGATCATTGGATAAATTTTTT
ATAATTATAAATAAAGATAATAATTTTTTTTTTAACAAAACTAAAAATAA
AAATAATAAAATAATTGTTAAAATAGGTTTTTTTTTTTTTTTTTTTTTTT
TAATAAATGGTATTTATTAATTTATTTGTTGTGTGTGTTTTTTTTTTTAT
AATATTTTTTTTTTTAGCATTGAATTAAGAAGAAATCAAATTGATGCGGC
CGCTTAGTTAGCCTCCCCCATCTCCCGATCCGGACGAGTGCTGGGGCGTC
GGTTTCCACTATCGGCGAGTACTTCTACACAGCCATCGGTCCAGACGGCC
GCGCTTCTGCGGGCGATTTGTGTACGCCCGACAGTCCCGGCTCCGGATCG
GACGATTGCGTCGCATCGACCCTGCGCCCAAGCTGCATCATCGAAATTGC
CGTCAACCAAGCTCTGATAGAGTTGGTCAAGACCAATGCGGAGCATATAC
GCCCGGAGCCGCGGCGATCCTGCAAGCTCCGGATGCCTCCGCTCGAAGTA
GCGCGTCTGCTGCTCCATACAAGCCAACCACGGCCTCCAGAAGAAGATGT
TGGCGACCTCGTATTGGGAATCCCCGAACATCGCCTCGCTCCAGTCAATG
ACCGCTGTTATGCGGCCATTGTCCGTCAGGACATTGTTGGAGCCGAAATC
CGCGTGCACGAGGTGCCGGACTTCGGGGCAGTCCTCGGCCCAAAGCATCA
GCTCATCGAGAGCCTGCGCGACGGACGCACTGACGGTGTCGTCCATCACA
GTTTGCCAGTGATACACATGGGGATCAGCAATCGCGCATATGAAATCACG
CCATGTAGTGTATTGACCGATTCCTTGCGGTCCGAATGGGCCGAACCCGC
TCGTCTGGCTAAGATCGGCCGCAGCGATCGCATCCATGGCCTCCGCGACC
GGCTGCAGAACAGCGGGCAGTTCGGTTTCAGGCAGGTCTTGCAACGTGAC
ACCCTGTGCACGGCGGGAGATGCAATAGGTCAGGCTCTCGCTGAACTCCC
CAATGTCAAGCACTTCCGGAATCGGGAGCGCGGCCGATGCAAAGTGCCGA
TAAACATAACGATCTTTGTAGAAACCATCGGCGCAGCTATTTACCCGCAG
GACATATCCACGCCCTCCTACATCGAAGCTGAAAGCACGAGATTCTTCGC
CCTCCGAGAGCTGCATCAGGTCGGAGACGCTGTCGAACTTTTCGATCAGA
AACTTCTCGACAGACGTCGCGGTGAGTTCAGGCTTTTTCATTAGGTCTTG
TTGAGAAATGTTAAATTGATCCATTTTTTGCTAGCTGTGAAATTAGTTTA

AAATACAAATAAAGAGTTATAATAATATACAGTTGAATAAAAAAAAAAAA
AATGAATTGGAAAATTTATTTTTATATGAAGAAAAAAAAAATTTTGAAAAA
AAAAAAAAAAAATTAAAAAAAAAAAAAAAAAAAAAAAAAAAATTAAAAATAAT
TCAACTGTGGGTGACCCAAATTTTATTTAAAAAAAAAAAAAAAAATGGATC
ACTTTTGGGTGGTTGGAAAAAAAAAAAAAAAAAAAATTGAAAATATAATGT
TAGTCATATGATTAATCATTAATTAATTCTTTTTTTTTTTTTTTTTTATTT
TTTATTTTTTTTTTTTCAATATTCCAATTATGACACACACTAACATCACA
AAATACAAAATTTTGGGATCCACTACTATAACTTCGTATAATGTATGCTA
TACGAAGTTATTTAATTAATTAAGATATCAAGCTTGATAGCTCTGCCTAC
TGAAGGCCGGCAGAGGCGGTTTGCGTATTGGGCGCTCTTCCGCTTCCTCG
CTCACTGACTCGCTGCGCTCGGTCGTTCGGCTGCGGCGAGCGGTATCAGC
TCACTCAAAGGCGGTAATACGGTTATCCACAGAATCAGGGGATAACGCAG
GAAAGAACATGTGAGCAAAAGGCCAGCAAAAGGCCAGGAACCGTAAAAAG
GCCGCGTTGCTGGCGTTTTTCCATAGGCTCCGCCCCCCTGACGAGCATCA
CAAAAATCGACGCTCAAGTCAGAGGTGGCGAAACCCGACAGGACTATAAA
GATACCAGGCGTTTCCCCCTGGAAGCTCCCTCGTGCGCTCTCCTGTTCCG
ACCCTGCCGCTTACCGGATACCTGTCCGCCTTTCTCCCTTCGGGAAGCGT
GGCGCTTTCTCATAGCTCACGCTGTAGGTATCTCAGTTCGGTGTAGGTCG
TTCGCTCCAAGCTGGGCTGTGTGCACGAACCCCCCGTTCAGCCCGACCGC
TGCGCCTTATCCGGTAACTATCGTCTTGAGTCCAACCCGGTAAGACACGA
CTTATCGCCACTGGCAGCAGCCACTGGTAACAGGATTAGCAGAGCGAGGT
ATGTAGGCGGTGCTACAGAGTTCTTGAAGTGGTGGCCTAACTACGGCTAC
ACTAGAAGGACAGTATTTGGTATCTGCGCTCTGCTGAAGCCAGTTACCTT
CGGAAAAAGAGTTGGTAGCTCTTGATCCGGCAAACAAACCACCGCTGGTA
GCGGTGGTTTTTTTGTTTGCAAGCAGCAGATTACGCGCAGAAAAAAAGGA
TCTCAAGAAGATCCTTTGATCTTTTCTACGGGGTCTGACGCTCAGTGGAA
CGAAAACTCACGTTAAGGGATTTTGGTCATGAGATTATCAAAAAGGATCT
TCACCTAGATCCTTTTAAATTAAAAATGAAGTTTTAAATCAATCTAAAGT
ATATATGAGTAAACTTGGTCTGACAGTTACCAATGCTTAATCAGTGAGGC
ACCTATCTCAGCGATCTGTCTATTTCGTTCATCCATAGTTGCCTGACTCC
CCGTCGTGTAGATAACTACGATACGGGAGGGCTTACCATCTGGCCCCAGT
GCTGCAATGATACCGCGAGACCCACGCTCACCGGCTCCAGATTTATCAGC
AATAAACCAGCCAGCCGGAAGGGCCGAGCGCAGAAGTGGTCCTGCAACTT
TATCCGCCTCCATCCAGTCTATTAATTGTTGCCGGGAAGCTAGAGTAAGT
AGTTCGCCAGTTAATAGTTTGCGCAACGTTGTTGCCATTGCTACAGGCAT
CGTGGTGTCACGCTCGTCGTTTGGTATGGCTTCATTCAGCTCCGGTTCCC
AACGATCAAGGCGAGTTACATGATCCCCCATGTTGTGCAAAAAAGCGGTT
AGCTCCTTCGGTCCTCCGATCGTTGTCAGAAGTAAGTTGGCCGCAGTGTT
ATCACTCATGGTTATGGCAGCACTGCATAATTCTCTTACTGTCATGCCAT
CCGTAAGATGCTTTTCTGTGACTGGTGAGTACTCAACCAAGTCATTCTGA
GAATAGTGTATGCGGCGACCGAGTTGCTCTTGCCCGGCGTCAATACGGGA
TAATACCGCGCCACATAGCAGAACTTTAAAAGTGCTCATCATTGGAAAAC
GTTCTTCGGGGCGAAAACTCTCAAGGATCTTACCGCTGTTGAGATCCAGT
TCGATGTAACCCACTCGTGCACCCAACTGATCTTCAGCATCTTTTACTTT
CACCAGCGTTTCTGGGTGAGCAAAAACAGGAAGGCAAAATGCCGCAAAAA
AGGGAATAAGGGCGACACGGAAATGTTGAATACTCATACTCTTCCTTTTT
CAATATTATTGAAGCATTTATCAGGGTTATTGTCTCATGAGCGGATACAT
ATTTGAATGTATTTAGAAAAATAAACAAATAGGGGTTCCGCGCACATTTC
CCCGAAAAGTGCCACCTGACGCGCCCTGTAGCGGGATCA

pPI462 – mNeon (C)

ACTAGTTGGTGGTTCAGGAGGTAGTGTGAGTAAAGGTGAAGAAGATAATA
TGGCATCGTTACCAGCTACACATGAGTTACATATATTCGGTAGCATTAAT
GGTGTTGATTTTGATATGGTGGGACAAGGTACCGGTAATCCTAATGATGG
TTACGAAGAACTAAATTTAAAATCGACTAAAGGTGACTTACAATTTTCTC
CATGGATTTTAGTGCCACATATAGGGTATGGTTTTCATCAATACTTACCA
TATCCAGATGGTATGTCACCATTTCAAGCTGCAATGGTTGATGGATCAGG
TTATCAAGTTCATAGAACAATGCAATTTGAAGATGGTGCTTCATTAACTG
TTAATTATAGATACACATATGAAGGCTCACATATTAAAGGTGAAGCTCAA
GTTAAAGGTACTGGTTTCCCAGCCGATGGCCCAGTTATGACAAATAGTTT
AACAGCAGCAGATTGGTGTAGATCCAAAAAAACTTATCCAAATGATAAAA
CAATTATTTCAACTTTTAAATGGTCATATACAACCGGTAATGGTAAACGT
TATCGTTCAACAGCCCGTACAACATATACTTTTGCTAAACCAATGGCAGC
TAATTATTTAAAAAATCAACCAATGTATGTTTTTCGTAAAACAGAGTTAA
AACATTCAAAAACAGAACTTAATTTTAAAGAATGGCAAAAAGCATTTACA
GACGTTATGTAAAGATCTGATATCATAACTTCGTATAATGTATGCTATAC
GAAGTTATACTACTCTCGAGATTTTATTTAATATACTAAATAATAAAAAA
GTTAAAAAATGATCATTGGATAAATTTTTTATAATTATAAATAAAGATAA
TAATTTTTTTTTTAACAAAACTAAAAATAAAAATAATAAAATAATTGTTA
AAATAGGTTTTTTTTTTTTTTTTTTTTTTTTAATAAATGGTATTTATTAA
TTTATTTGTTGTGTGTGTTTTTTTTTTTATAATATTTTTTTTTTTAGCAT
TGAATTAAGAAGAAATCAAATTGATGCGGCCGCTTAGTTAGCCTCCCCCA
TCTCCCGATCCGGACGAGTGCTGGGGCGTCGGTTTCCACTATCGGCGAGT
ACTTCTACACAGCCATCGGTCCAGACGGCCGCGCTTCTGCGGGCGATTTG
TGTACGCCCGACAGTCCCGGCTCCGGATCGGACGATTGCGTCGCATCGAC
CCTGCGCCCAAGCTGCATCATCGAAATTGCCGTCAACCAAGCTCTGATAG
AGTTGGTCAAGACCAATGCGGAGCATATACGCCCGGAGCCGCGGCGATCC
TGCAAGCTCCGGATGCCTCCGCTCGAAGTAGCGCGTCTGCTGCTCCATAC
AAGCCAACCACGGCCTCCAGAAGAAGATGTTGGCGACCTCGTATTGGGAA
TCCCCGAACATCGCCTCGCTCCAGTCAATGACCGCTGTTATGCGGCCATT
GTCCGTCAGGACATTGTTGGAGCCGAAATCCGCGTGCACGAGGTGCCGGA
CTTCGGGGCAGTCCTCGGCCCAAAGCATCAGCTCATCGAGAGCCTGCGCG
ACGGACGCACTGACGGTGTCGTCCATCACAGTTTGCCAGTGATACACATG
GGGATCAGCAATCGCGCATATGAAATCACGCCATGTAGTGTATTGACCGA
TTCCTTGCGGTCCGAATGGGCCGAACCCGCTCGTCTGGCTAAGATCGGCC
GCAGCGATCGCATCCATGGCCTCCGCGACCGGCTGCAGAACAGCGGGCAG
TTCGGTTTCAGGCAGGTCTTGCAACGTGACACCCTGTGCACGGCGGGAGA
TGCAATAGGTCAGGCTCTCGCTGAACTCCCCAATGTCAAGCACTTCCGGA
ATCGGGAGCGCGGCCGATGCAAAGTGCCGATAAACATAACGATCTTTGTA
GAAACCATCGGCGCAGCTATTTACCCGCAGGACATATCCACGCCCTCCTA
CATCGAAGCTGAAAGCACGAGATTCTTCGCCCTCCGAGAGCTGCATCAGG
TCGGAGACGCTGTCGAACTTTTCGATCAGAAACTTCTCGACAGACGTCGC
GGTGAGTTCAGGCTTTTTCATTAGGTCTTGTTGAGAAATGTTAAATTGAT
CCATTTTTTGCTAGCTGTGAAATTAGTTTAAAATACAAATAAAGAGTTAT
AATAATATACAGTTGAATAAAAAAAAAAAAAATGAATTGGAAAATTTATT
TTTATATGAAGAAAAAAAAAATTTTGAAAAAAAAAAAAAAAAATTAAAAAAA
AAAAAAAAAAAAAAAAAAATTAAAAATAATTCAACTGTGGGTGACCCAAA
TTTTATTTAAAAAAAAAAAAAAAATGGATCACTTTTGGGTGGTTGGAAAA
AAAAAAAAAAAAAAAATTGAAAATATAATGTTAGTCATATGATTAATCATT

AATTAATTCTTTTTTTTTTTTTTTTTATTTTTATTTTTTTTTTTTCAAT
ATTCCAATTATGACACACACTAACATCACAAAATACAAAATTTTGGGATC
CACTACTATAACTTCGTATAATGTATGCTATACGAAGTTATTTAATTAAT
TAAGATATCAAGCTTGATAGCTCTGCCTACTGAAGGCCGGCAGAGGCGGT
TTGCGTATTGGGCGCTCTTCCGCTTCCTCGCTCACTGACTCGCTGCGCTC
GGTCGTTCGGCTGCGGCGAGCGGTATCAGCTCACTCAAAGGCGGTAATAC
GGTTATCCACAGAATCAGGGGATAACGCAGGAAAGAACATGTGAGCAAAA
GGCCAGCAAAAGGCCAGGAACCGTAAAAAGGCCGCGTTGCTGGCGTTTTT
CCATAGGCTCCGCCCCCCTGACGAGCATCACAAAAATCGACGCTCAAGTC
AGAGGTGGCGAAACCCGACAGGACTATAAAGATACCAGGCGTTTCCCCCT
GGAAGCTCCCTCGTGCGCTCTCCTGTTCCGACCCTGCCGCTTACCGGATA
CCTGTCCGCCTTTCTCCCTTCGGGAAGCGTGGCGCTTTCTCATAGCTCAC
GCTGTAGGTATCTCAGTTCGGTGTAGGTCGTTCGCTCCAAGCTGGGCTGT
GTGCACGAACCCCCCGTTCAGCCCGACCGCTGCGCCTTATCCGGTAACTA
TCGTCTTGAGTCCAACCCGGTAAGACACGACTTATCGCCACTGGCAGCAG
CCACTGGTAACAGGATTAGCAGAGCGAGGTATGTAGGCGGTGCTACAGAG
TTCTTGAAGTGGTGGCCTAACTACGGCTACACTAGAAGGACAGTATTTGG
TATCTGCGCTCTGCTGAAGCCAGTTACCTTCGGAAAAAGAGTTGGTAGCT
CTTGATCCGGCAAACAAACCACCGCTGGTAGCGGTGGTTTTTTTGTTTGC
AAGCAGCAGATTACGCGCAGAAAAAAAGGATCTCAAGAAGATCCTTTGAT
CTTTTCTACGGGGTCTGACGCTCAGTGGAACGAAAACTCACGTTAAGGGA
TTTTGGTCATGAGATTATCAAAAAGGATCTTCACCTAGATCCTTTTAAAT
TAAAAATGAAGTTTTAAATCAATCTAAAGTATATATGAGTAAACTTGGTC
TGACAGTTACCAATGCTTAATCAGTGAGGCACCTATCTCAGCGATCTGTC
TATTTCGTTCATCCATAGTTGCCTGACTCCCCGTCGTGTAGATAACTACG
ATACGGGAGGGCTTACCATCTGGCCCCAGTGCTGCAATGATACCGCGAGA
CCCACGCTCACCGGCTCCAGATTTATCAGCAATAAACCAGCCAGCCGGAA
GGGCCGAGCGCAGAAGTGGTCCTGCAACTTTATCCGCCTCCATCCAGTCT
ATTAATTGTTGCCGGGAAGCTAGAGTAAGTAGTTCGCCAGTTAATAGTTT
GCGCAACGTTGTTGCCATTGCTACAGGCATCGTGGTGTCACGCTCGTCGT
TTGGTATGGCTTCATTCAGCTCCGGTTCCCAACGATCAAGGCGAGTTACA
TGATCCCCCATGTTGTGCAAAAAAGCGGTTAGCTCCTTCGGTCCTCCGAT
CGTTGTCAGAAGTAAGTTGGCCGCAGTGTTATCACTCATGGTTATGGCAG
CACTGCATAATTCTCTTACTGTCATGCCATCCGTAAGATGCTTTTCTGTG
ACTGGTGAGTACTCAACCAAGTCATTCTGAGAATAGTGTATGCGGCGACC
GAGTTGCTCTTGCCCGGCGTCAATACGGGATAATACCGCGCCACATAGCA
GAACTTTAAAAGTGCTCATCATTGGAAAACGTTCTTCGGGGCGAAAACTC
TCAAGGATCTTACCGCTGTTGAGATCCAGTTCGATGTAACCCACTCGTGC
ACCCAACTGATCTTCAGCATCTTTTACTTTCACCAGCGTTTCTGGGTGAG
CAAAAACAGGAAGGCAAAATGCCGCAAAAAAGGGAATAAGGGCGACACGG
AAATGTTGAATACTCATACTCTTCCTTTTTCAATATTATTGAAGCATTTA
TCAGGGTTATTGTCTCATGAGCGGATACATATTTGAATGTATTTAGAAAA
ATAAACAAATAGGGGTTCCGCGCACATTTCCCCGAAAAGTGCCACCTGAC
GCGCCCTGTAGCGGGATCA

pPI464 – mScarlet (C)

ACTAGTGGTGGTTCAGGAGGTAGTGTTTCAAAAGGTGAAGCCGTTATTAA
AGAATTTATGAGATTCAAGGTTCACATGGAAGGAAGTATGAACGGTCATG
AATTTGAGATTGAAGGAGAAGGTGAAGGTAGACCATATGAAGGCACCCAA

ACAGCTAAATTAAAAGTAACTAAAGGTGGTCCATTACCATTTAGTTGGGA
TATTTTATCTCCACAATTTATGTATGGTTCACGTGCTTTCACAAAACATC
CAGCAGATATTCCAGATTATTATAAACAATCATTTCCAGAAGGTTTTAAA
TGGGAACGTGTCATGAACTTTGAAGATGGTGGAGCAGTTACAGTCACACA
AGATACCTCATTAGAAGATGGTACATTAATATATAAAGTTAAATTACGTG
GTACTAATTTTCCACCAGACGGTCCAGTAATGCAAAAAAAAACAATGGGC
TGGGAAGCTAGTACAGAACGTTTATATCCTGAAGATGGTGTCCTTAAAGG
CGATATAAAAATGGCCTTGAGATTAAAGGATGGTGGTAGGTATTTAGCAG
ATTTCAAAACCACTTATAAAGCAAAAAAACCAGTTCAAATGCCAGGTGCA
TATAATGTTGATAGAAAACTTGATATTACCAGTCATAATGAAGATTACAC
AGTTGTCGAACAATACGAACGTTCTGAAGGTCGTCATAGCACTGGTGGTA
TGGATGAATTATACAAATAAAGATCTGATATCATAACTTCGTATAATGTA
TGCTATACGAAGTTATACTACTCTCGAGATTTTATTTAATATACTAAATA
ATAAAAAAGTTAAAAAATGATCATTGGATAAATTTTTTATAATTATAAAT
AAAGATAATAATTTTTTTTTTAACAAAACTAAAAATAAAAATAATAAAAT
AATTGTTAAAATAGGTTTTTTTTTTTTTTTTTTTTTTTAATAAATGGTA
TTTATTAATTTATTTGTTGTGTGTGTTTTTTTTTTTATAATATTTTTTTT
TTTAGCATTGAATTAAGAAGAAATCAAATTGATGCGGCCGCTTAGTTAGC
CTCCCCCATCTCCCGATCCGGACGAGTGCTGGGGCGTCGGTTTCCACTAT
CGGCGAGTACTTCTACACAGCCATCGGTCCAGACGGCCGCGCTTCTGCGG
GCGATTTGTGTACGCCCGACAGTCCCGGCTCCGGATCGGACGATTGCGTC
GCATCGACCCTGCGCCCAAGCTGCATCATCGAAATTGCCGTCAACCAAGC
TCTGATAGAGTTGGTCAAGACCAATGCGGAGCATATACGCCCGGAGCCGC
GGCGATCCTGCAAGCTCCGGATGCCTCCGCTCGAAGTAGCGCGTCTGCTG
CTCCATACAAGCCAACCACGGCCTCCAGAAGAAGATGTTGGCGACCTCGT
ATTGGGAATCCCCGAACATCGCCTCGCTCCAGTCAATGACCGCTGTTATG
CGGCCATTGTCCGTCAGGACATTGTTGGAGCCGAAATCCGCGTGCACGAG
GTGCCGGACTTCGGGGCAGTCCTCGGCCCAAAGCATCAGCTCATCGAGAG
CCTGCGCGACGGACGCACTGACGGTGTCGTCCATCACAGTTTGCCAGTGA
TACACATGGGGATCAGCAATCGCGCATATGAAATCACGCCATGTAGTGTA
TTGACCGATTCCTTGCGGTCCGAATGGGCCGAACCCGCTCGTCTGGCTAA
GATCGGCCGCAGCGATCGCATCCATGGCCTCCGCGACCGGCTGCAGAACA
GCGGGCAGTTCGGTTTCAGGCAGGTCTTGCAACGTGACACCCTGTGCACG
GCGGGAGATGCAATAGGTCAGGCTCTCGCTGAACTCCCCAATGTCAAGCA
CTTCCGGAATCGGGAGCGCGGCCGATGCAAAGTGCCGATAAACATAACGA
TCTTTGTAGAAACCATCGGCGCAGCTATTTACCCGCAGGACATATCCACG
CCCTCCTACATCGAAGCTGAAAGCACGAGATTCTTCGCCCTCCGAGAGCT
GCATCAGGTCGGAGACGCTGTCGAACTTTTCGATCAGAAACTTCTCGACA
GACGTCGCGGTGAGTTCAGGCTTTTTCATTAGGTCTTGTTGAGAAATGTT
AAATTGATCCATTTTTTGCTAGCTGTGAAATTAGTTTAAAATACAAATAA
AGAGTTATAATAATATACAGTTGAATAAAAAAAAAAAAAAATGAATTGGAA
AATTTATTTTTATATGAAGAAAAAAAAAATTTTGAAAAAAAAAAAAAAAAAT
TAAAAAAAAAAAAAAAAAAAAAAAAAAATTAAAAATAATTCAACTGTGGGT
GACCCAAATTTTATTTAAAAAAAAAAAAAAAAATGGATCACTTTTGGGTGG
TTGGAAAAAAAAAAAAAAAAAAAAATTGAAAATATAATGTTAGTCATATGAT
TAATCATTAATTAATTCTTTTTTTTTTTTTTTTTTATTTTTTTATTTTTTTT
TTTTCAATATTCCAATTATGACACACACTAACATCACAAAATACAAAATT
TTGGGATCCACTACTATAACTTCGTATAATGTATGCTATACGAAGTTATT
TAATTAATTAAGATATCAAGCTTGATAGCTCTGCCTACTGAAGGCCGGCA
GAGGCGGTTTGCGTATTGGGCGCTCTTCCGCTTCCTCGCTCACTGACTCG

CTGCGCTCGGTCGTTCGGCTGCGGCGAGCGGTATCAGCTCACTCAAAGGC
GGTAATACGGTTATCCACAGAATCAGGGGATAACGCAGGAAAGAACATGT
GAGCAAAAGGCCAGCAAAAGGCCAGGAACCGTAAAAAGGCCGCGTTGCTG
GCGTTTTTCCATAGGCTCCGCCCCCCTGACGAGCATCACAAAAATCGACG
CTCAAGTCAGAGGTGGCGAAACCCGACAGGACTATAAAGATACCAGGCGT
TTCCCCCTGGAAGCTCCCTCGTGCGCTCTCCTGTTCCGACCCTGCCGCTT
ACCGGATACCTGTCCGCCTTTCTCCCTTCGGGAAGCGTGGCGCTTTCTCA
TAGCTCACGCTGTAGGTATCTCAGTTCGGTGTAGGTCGTTCGCTCCAAGC
TGGGCTGTGTGCACGAACCCCCCGTTCAGCCCGACCGCTGCGCCTTATCC
GGTAACTATCGTCTTGAGTCCAACCCGGTAAGACACGACTTATCGCCACT
GGCAGCAGCCACTGGTAACAGGATTAGCAGAGCGAGGTATGTAGGCGGTG
CTACAGAGTTCTTGAAGTGGTGGCCTAACTACGGCTACACTAGAAGGACA
GTATTTGGTATCTGCGCTCTGCTGAAGCCAGTTACCTTCGGAAAAAGAGT
TGGTAGCTCTTGATCCGGCAAACAAACCACCGCTGGTAGCGGTGGTTTTT
TTGTTTGCAAGCAGCAGATTACGCGCAGAAAAAAAGGATCTCAAGAAGAT
CCTTTGATCTTTTCTACGGGGTCTGACGCTCAGTGGAACGAAAACTCACG
TTAAGGGATTTTGGTCATGAGATTATCAAAAAGGATCTTCACCTAGATCC
TTTTAAATTAAAAATGAAGTTTTAAATCAATCTAAAGTATATATGAGTAA
ACTTGGTCTGACAGTTACCAATGCTTAATCAGTGAGGCACCTATCTCAGC
GATCTGTCTATTTCGTTCATCCATAGTTGCCTGACTCCCCGTCGTGTAGA
TAACTACGATACGGGAGGGCTTACCATCTGGCCCCAGTGCTGCAATGATA
CCGCGAGACCCACGCTCACCGGCTCCAGATTTATCAGCAATAAACCAGCC
AGCCGGAAGGGCCGAGCGCAGAAGTGGTCCTGCAACTTTATCCGCCTCCA
TCCAGTCTATTAATTGTTGCCGGGAAGCTAGAGTAAGTAGTTCGCCAGTT
AATAGTTTGCGCAACGTTGTTGCCATTGCTACAGGCATCGTGGTGTCACG
CTCGTCGTTTGGTATGGCTTCATTCAGCTCCGGTTCCCAACGATCAAGGC
GAGTTACATGATCCCCCATGTTGTGCAAAAAAGCGGTTAGCTCCTTCGGT
CCTCCGATCGTTGTCAGAAGTAAGTTGGCCGCAGTGTTATCACTCATGGT
TATGGCAGCACTGCATAATTCTCTTACTGTCATGCCATCCGTAAGATGCT
TTTCTGTGACTGGTGAGTACTCAACCAAGTCATTCTGAGAATAGTGTATG
CGGCGACCGAGTTGCTCTTGCCCGGCGTCAATACGGGATAATACCGCGCC
ACATAGCAGAACTTTAAAAGTGCTCATCATTGGAAAACGTTCTTCGGGGC
GAAAACTCTCAAGGATCTTACCGCTGTTGAGATCCAGTTCGATGTAACCC
ACTCGTGCACCCAACTGATCTTCAGCATCTTTTACTTTCACCAGCGTTTC
TGGGTGAGCAAAAACAGGAAGGCAAAATGCCGCAAAAAAGGGAATAAGGG
CGACACGGAAATGTTGAATACTCATACTCTTCCTTTTTCAATATTATTGA
AGCATTTATCAGGGTTATTGTCTCATGAGCGGATACATATTTGAATGTAT
TTAGAAAAATAAACAAATAGGGGTTCCGCGCACATTTCCCCGAAAAGTGC
CACCTGACGCGCCCTGTAGCGGGATCA

pPI191 – GFP (C) Hyg

CTAAATTGTAAGCGTTAATATTTTGTTAAAATTCGCGTTAAATTTTTGTT
AAATCAGCTCATTTTTTAACCAATAGGCCGAAATCGGCAAAATCCCTTAT
AAATCAAAAGAATAGACCGAGATAGGGTTGAGTGTTGTTCCAGTTTGGAA
CAAGAGTCCACTATTAAAGAACGTGGACTCCAACGTCAAAGGGCGAAAAA
CCGTCTATCAGGGCGATGGCCCACTACGTGAACCATCACCCTAATCAAGT
TTTTTGGGGTCGAGGTGCCGTAAAGCACTAAATCGGAACCCTAAAGGGAG
CCCCCGATTTAGAGCTTGACGGGGAAAGCCGGCGAACGTGGCGAGAAAGG
AAGGGAAGAAAGCGAAAGGAGCGGGCGCTAGGGCGCTGGCAAGTGTAGCG

GTCACGCTGCGCGTAACCACCACACCCGCCGCGCTTAATGCGCCGCTACA
GGGCGCGTCCCATTCGCCATTCAGGCTGCGCAACTGTTGGGAAGGGCGAT
CGGTGCGGGCCTCTTCGCTATTACGCCAGCTGGCGAAAGGGGGATGTGCT
GCAAGGCGATTAAGTTGGGTAACGCCAGGGTTTTCCCAGTCACGACGTTG
TAAAACGACGGCCAGTGAGCGCGCGTAATACGACTCACTATAGGGCGAAT
TGGGTACCGGGCCCCCCCTCGACTACGCCGGCTCAAGATCTTCAACTAGTG
GTGGTTCAGGAGGTAGTTCAAAAGGTGAAGAATTATTTACAGGTGTTGTT
CCAATTTTAGTTGAATTAGATGGTGATGTTAATGGTCATAAATTTTCAGT
TTCAGGTGAAGGTGAAGGTGATGCAACATATGGTAAATTAACATTAAAAT
TTATTTGTACAACAGGTAAATTACCAGTTCCATGGCCAACATTAGTTACA
ACATTTACATATGGTGTTCAATGTTTTTCAAGATATCCAGATCATATGAA
ACAACATGATTTTTTTAAATCAGCAATGCCAGAAGGTTATGTTCAAGAAA
GAACAATTTTTTTTAAAGATGATGGTAATTATAAAACAAGAGCAGAAGTT
AAATTTGAAGGTGATACATTAGTTAATAGAATTGAATTAAAAGGTATTGA
TTTTAAAGAAGATGGTAATATTTTAGGTCATAAATTAGAATATAATTATA
ATTCACATAATGTTTATATTATGGCAGATAAACAAAAAAATGGTATTAAA
GTTAATTTTAAAATTAGACATAATATTGAAGATGGTTCAGTTCAATTAGC
AGATCATTATCAACAAAATACACCAATTGGTGATGGTCCAGTTTTATTAC
CAGATAATCATTATTTATCAACACAATCAGCATTATCAAAAGATCCAAAT
GAAAAAAGAGATCATATGGTTTTATTAGAATTTGTTACAGCAGCAGGTAT
TACACATGGTATGGATGAATTATATAAATAAGCTAGTTAAATAAATAAAT
TATTTAATAAATAATAAAAAAAACAAATTGTTGTAATAATCTAATATTTTC
TTTTTTTTTTAATTTTTTTTTTTTAAATCTTAATAATTATTAAGTTATTT
TAATTTTTTTTTTTTTTTTTTTTTTTTTTTTTTTTTTTTTCTATCAAAA
AAATCAAATATATTTAAAAAATTTATTATTTACAGATACATTTTGAATGG
TGAAGATAAATATATGCATTAGATGTAAAACAGCCAAAGAGTATGAAAAT
CAAAAAGATAAAGCTTGATATCTTAATTAATTAAATAACTTCGTATAGCA
TACATTATACGAAGTTATAGTAGTGGATCCCAAAATTTTGTATTTTGTGA
TGTTAGTGTGTGTCATAATTGGAATATTGAAAAAAAAAAAATAAAAAATA
AAAAAAAAAAAAAAAAGAATTAATTAATGATTAATCATATGACTAACATT
ATATTTTCAATTTTTTTTTTTTTTTTTTTCCAACCACCCAAAAGTGATCC
ATTTTTTTTTTTTTTTTAAATAAAATTTGGGTCACCCACAGTTGAATTAT
TTTTAATTTTTTTTTTTTTTTTTTTTTTTTTTAATTTTTTTTTTTTTTTT
CAAAATTTTTTTTTCTTCATATAAAAATAAATTTTCCAATTCATTTTTTT
TTTTTTTATTCAACTGTATATTATTATAACTCTTTATTTGTATTTTAAAC
TAATTTCACAGCTAGCAAAAAATGGATCAATTTAACATTTCTCAACAAGA
CCTAATGAAAAAGCCTGAACTCACCGCGACGTCTGTCGAGAAGTTTCTGA
TCGAAAAGTTCGACAGCGTCTCCGACCTGATGCAGCTCTCGGAGGGCGAA
GAATCTCGTGCTTTCAGCTTCGATGTAGGAGGGCGTGGATATGTCCTGCG
GGTAAATAGCTGCGCCGATGGTTTCTACAAAGATCGTTATGTTTATCGGC
ACTTTGCATCGGCCGCGCTCCCGATTCCGGAAGTGCTTGACATTGGGGAG
TTCAGCGAGAGCCTGACCTATTGCATCTCCCGCCGTGCACAGGGTGTCAC
GTTGCAAGACCTGCCTGAAACCGAACTGCCCGCTGTTCTGCAGCCGGTCG
CGGAGGCCATGGATGCGATCGCTGCGGCCGATCTTAGCCAGACGAGCGGG
TTCGGCCCATTCGGACCGCAAGGAATCGGTCAATACACTACATGGCGTGA
TTTCATATGCGCGATTGCTGATCCCCATGTGTATCACTGGCAAACTGTGA
TGGACGACACCGTCAGTGCGTCCGTCGCGCAGGCTCTCGATGAGCTGATG
CTTTGGGCCGAGGACTGCCCCGAAGTCCGGCACCTCGTGCACGCGGATTT
CGGCTCCAACAATGTCCTGACGGACAATGGCCGCATAACAGCGGTCATTG
ACTGGAGCGAGGCGATGTTCGGGGATTCCCAATACGAGGTCGCCAACATC

TTCTTCTGGAGGCCGTGGTTGGCTTGTATGGAGCAGCAGACGCGCTACTT
CGAGCGGAGGCATCCGGAGCTTGCAGGATCGCCGCGGCTCCGGGCGTATA
TGCTCCGCATTGGTCTTGACCAACTCTATCAGAGCTTGGTTGACGGCAAT
TTCGATGATGCAGCTTGGGCGCAGGGTCGATGCGACGCAATCGTCCGATC
CGGAGCCGGGACTGTCGGGCGTACACAAATCGCCCGCAGAAGCGCGGCCG
TCTGGACCGATGGCTGTGTAGAAGTACTCGCCGATAGTGGAAACCGACGC
CCCAGCACTCGTCCGGATCGGGAGATGGGGGAGGCTAACTAAGCGGCCGC
ATCAATTTGATTTCTTCTTAATTCAATGCTAAAAAAAAAAAATATTATAAA
AAAAAAAACACACACAACAAATAAATTAATAAATACCATTTATTAAAAAA
AAAAAAAAAAAAAAAAAAACCTATTTTAACAATTATTTTATTATTTTTATT
TTTAGTTTTGTTAAAAAAAAAATTATTATCTTTATTTATAATTATAAAAA
ATTTATCCAATGATCATTTTTTAACTTTTTTATTATTTAGTATATTAAAT
AAAATCTCGAGAGTAGTATAACTTCGTATAGCATACATTATACGAAGTTA
TGATATCTCATCTAGATCAGTCGACTCAGCCGGCTCAGAGCTCCAGCTTT
TGTTCCCTTTAGTGAGGGTTAATTGCGCGCTTGGCGTAATCATGGTCATA
GCTGTTTCCTGTGTGAAATTGTTATCCGCTCACAATTCCACACAACATAC
GAGCCGGAAGCATAAAGTGTAAAGCCTGGGGTGCCTAATGAGTGAGCTAA
CTCACATTAATTGCGTTGCGCTCACTGCCCGCTTTCCAGTCGGGAAACCT
GTCGTGCCAGCTGCATTAATGAATCGGCCAACGCGCGGGGAGAGGCGGTT
TGCGTATTGGGCGCTCTTCCGCTTCCTCGCTCACTGACTCGCTGCGCTCG
GTCGTTCGGCTGCGGCGAGCGGTATCAGCTCACTCAAAGGCGGTAATACG
GTTATCCACAGAATCAGGGGATAACGCAGGAAAGAACATGTGAGCAAAAG
GCCAGCAAAAGGCCAGGAACCGTAAAAAGGCCGCGTTGCTGGCGTTTTTC
CATAGGCTCCGCCCCCCTGACGAGCATCACAAAAATCGACGCTCAAGTCA
GAGGTGGCGAAACCCGACAGGACTATAAAGATACCAGGCGTTTCCCCCTG
GAAGCTCCCTCGTGCGCTCTCCTGTTCCGACCCTGCCGCTTACCGGATAC
CTGTCCGCCTTTCTCCCTTCGGGAAGCGTGGCGCTTTCTCATAGCTCACG
CTGTAGGTATCTCAGTTCGGTGTAGGTCGTTCGCTCCAAGCTGGGCTGTG
TGCACGAACCCCCCGTTCAGCCCGACCGCTGCGCCTTATCCGGTAACTAT
CGTCTTGAGTCCAACCCGGTAAGACACGACTTATCGCCACTGGCAGCAGC
CACTGGTAACAGGATTAGCAGAGCGAGGTATGTAGGCGGTGCTACAGAGT
TCTTGAAGTGGTGGCCTAACTACGGCTACACTAGAAGGACAGTATTTGGT
ATCTGCGCTCTGCTGAAGCCAGTTACCTTCGGAAAAAGAGTTGGTAGCTC
TTGATCCGGCAAACAAACCACCGCTGGTAGCGGTGGTTTTTTTGTTTGCA
AGCAGCAGATTACGCGCAGAAAAAAAGGATCTCAAGAAGATCCTTTGATC
TTTTCTACGGGGTCTGACGCTCAGTGGAACGAAAACTCACGTTAAGGGAT
TTTGGTCATGAGATTATCAAAAAGGATCTTCACCTAGATCCTTTTAAATT
AAAAATGAAGTTTTAAATCAATCTAAAGTATATATGAGTAAACTTGGTCT
GACAGTTACCAATGCTTAATCAGTGAGGCACCTATCTCAGCGATCTGTCT
ATTTCGTTCATCCATAGTTGCCTGACTCCCCGTCGTGTAGATAACTACGA
TACGGGAGGGCTTACCATCTGGCCCCAGTGCTGCAATGATACCGCGAGAC
CCACGCTCACCGGCTCCAGATTTATCAGCAATAAACCAGCCAGCCGGAAG
GGCCGAGCGCAGAAGTGGTCCTGCAACTTTATCCGCCTCCATCCAGTCTA
TTAATTGTTGCCGGGAAGCTAGAGTAAGTAGTTCGCCAGTTAATAGTTTG
CGCAACGTTGTTGCCATTGCTACAGGCATCGTGGTGTCACGCTCGTCGTT
TGGTATGGCTTCATTCAGCTCCGGTTCCCAACGATCAAGGCGAGTTACAT
GATCCCCCATGTTGTGCAAAAAAGCGGTTAGCTCCTTCGGTCCTCCGATC
GTTGTCAGAAGTAAGTTGGCCGCAGTGTTATCACTCATGGTTATGGCAGC
ACTGCATAATTCTCTTACTGTCATGCCATCCGTAAGATGCTTTTCTGTGA
CTGGTGAGTACTCAACCAAGTCATTCTGAGAATAGTGTATGCGGCGACCG

AGTTGCTCTTGCCCGGCGTCAATACGGGATAATACCGCGCCACATAGCAG
AACTTTAAAAGTGCTCATCATTGGAAAACGTTCTTCGGGGCGAAAACTCT
CAAGGATCTTACCGCTGTTGAGATCCAGTTCGATGTAACCCACTCGTGCA
CCCAACTGATCTTCAGCATCTTTTACTTTCACCAGCGTTTCTGGGTGAGC
AAAAACAGGAAGGCAAAATGCCGCAAAAAAGGGAATAAGGGCGACACGGA
AATGTTGAATACTCATACTCTTCCTTTTTCAATATTATTGAAGCATTTAT
CAGGGTTATTGTCTCATGAGCGGATACATATTTGAATGTATTTAGAAAAA
TAAACAAATAGGGGTTCCGCGCACATTTCCCCGAAAAGTGCCAC

pPI190 – mNeon (C) Hyg

CTAAATTGTAAGCGTTAATATTTTGTTAAAATTCGCGTTAAATTTTTGTT
AAATCAGCTCATTTTTTAACCAATAGGCCGAAATCGGCAAAATCCCTTAT
AAATCAAAAGAATAGACCGAGATAGGGTTGAGTGTTGTTCCAGTTTGGAA
CAAGAGTCCACTATTAAAGAACGTGGACTCCAACGTCAAAGGGCGAAAAA
CCGTCTATCAGGGCGATGGCCCACTACGTGAACCATCACCCTAATCAAGT
TTTTTGGGGTCGAGGTGCCGTAAAGCACTAAATCGGAACCCTAAAGGGAG
CCCCCGATTTAGAGCTTGACGGGGAAAGCCGGCGAACGTGGCGAGAAAGG
AAGGGAAGAAAGCGAAAGGAGCGGGCGCTAGGGCGCTGGCAAGTGTAGCG
GTCACGCTGCGCGTAACCACCACACCCGCCGCGCTTAATGCGCCGCTACA
GGGCGCGTCCCATTCGCCATTCAGGCTGCGCAACTGTTGGGAAGGGCGAT
CGGTGCGGGCCTCTTCGCTATTACGCCAGCTGGCGAAAGGGGGATGTGCT
GCAAGGCGATTAAGTTGGGTAACGCCAGGGTTTTCCCAGTCACGACGTTG
TAAAACGACGGCCAGTGAGCGCGCGTAATACGACTCACTATAGGGCGAAT
TGGGTACCGGGCCCCCCCTCGACTACGCCGGCTCAAGATCTTCAACTAGTG
GTGGTTCAGGAGGTAGTGTGAGTAAAGGTGAAGAAGATAATATGGCATCG
TTACCAGCTACACATGAGTTACATATATTCGGTAGCATTAATGGTGTTGA
TTTTGATATGGTGGGACAAGGTACCGGTAATCCTAATGATGGTTACGAAG
AACTAAATTTAAAATCGACTAAAGGTGACTTACAATTTTCTCCATGGATT
TTAGTGCCACATATAGGGTATGGTTTTCATCAATACTTACCATATCCAGA
TGGTATGTCACCATTTCAAGCTGCAATGGTTGATGGATCAGGTTATCAAG
TTCATAGAACAATGCAATTTGAAGATGGTGCTTCATTAACTGTTAATTAT
AGATACACATATGAAGGCTCACATATTAAAGGTGAAGCTCAAGTTAAAGG
TACTGGTTTCCCAGCCGATGGCCCAGTTATGACAAATAGTTTAACAGCAG
CAGATTGGTGTAGATCCAAAAAAACTTATCCAAATGATAAAACAATTATT
TCAACTTTTAAATGGTCATATACAACCGGTAATGGTAAACGTTATCGTTC
AACAGCCCGTACAACATATACTTTTGCTAAACCAATGGCAGCTAATTATT
TAAAAAATCAACCAATGTATGTTTTTCGTAAAACAGAGTTAAAACATTCA
AAAACAGAACTTAATTTTAAAGAATGGCAAAAAGCATTTACAGACGTTAT
GTAAGCTAGTAGTTAAATAAATAAATTATTTAATAAATAATAAAAAAACA
AATTGTTGTAATAATCTAATATTTTCTTTTTTTTTTAATTTTTTTTTTTT
AAATCTTAATAATTATTAAGTTATTTTAATTTTTTTTTTTTTTTTTTTTT
TTTTTTTTTTTTTTTTTCTATCAAAAAAATCAAATATATTTAAAAAATTT
ATTATTTACAGATACATTTTGAATGGTGAAGATAAATATATGCATTAGAT
GTAAAACAGCCAAAGAGTATGAAAATCAAAAAGATAAAGCTTGATATCTT
AATTAATTAAATAACTTCGTATAGCATACATTATACGAAGTTATAGTAGT
GGATCCCAAAATTTTGTATTTTGTGATGTTAGTGTGTGTCATAATTGGAA
TATTGAAAAAAAAAAAATAAAAAATAAAAAAAAAAAAAAAAAGAATTAAT
TAATGATTAATCATATGACTAACATTATATTTCAATTTTTTTTTTTTTT
TTTTTCCAACCACCCAAAAGTGATCCATTTTTTTTTTTTTTTAAATAAA

ATTTGGGTCACCCACAGTTGAATTATTTTAATTTTTTTTTTTTTTTTTTT
TTTTTTTTAATTTTTTTTTTTTTTTTCAAAATTTTTTTTTCTTCATATAA
AAATAAATTTTCCAATTCATTTTTTTTTTTTTTATTCAACTGTATATTAT
TATAACTCTTTATTTGTATTTTAAACTAATTTCACAGCTAGCAAAAAATG
GATCAATTTAACATTTCTCAACAAGACCTAATGAAAAAGCCTGAACTCAC
CGCGACGTCTGTCGAGAAGTTTCTGATCGAAAAGTTCGACAGCGTCTCCG
ACCTGATGCAGCTCTCGGAGGGCGAAGAATCTCGTGCTTTCAGCTTCGAT
GTAGGAGGGCGTGGATATGTCCTGCGGGTAAATAGCTGCGCCGATGGTTT
CTACAAAGATCGTTATGTTTATCGGCACTTTGCATCGGCCGCGCTCCCGA
TTCCGGAAGTGCTTGACATTGGGGAGTTCAGCGAGAGCCTGACCTATTGC
ATCTCCCGCCGTGCACAGGGTGTCACGTTGCAAGACCTGCCTGAAACCGA
ACTGCCCGCTGTTCTGCAGCCGGTCGCGGAGGCCATGGATGCGATCGCTG
CGGCCGATCTTAGCCAGACGAGCGGGTTCGGCCCATTCGGACCGCAAGGA
ATCGGTCAATACACTACATGGCGTGATTTCATATGCGCGATTGCTGATCC
CCATGTGTATCACTGGCAAACTGTGATGGACGACACCGTCAGTGCGTCCG
TCGCGCAGGCTCTCGATGAGCTGATGCTTTGGGCCGAGGACTGCCCCGAA
GTCCGGCACCTCGTGCACGCGGATTTCGGCTCCAACAATGTCCTGACGGA
CAATGGCCGCATAACAGCGGTCATTGACTGGAGCGAGGCGATGTTCGGGG
ATTCCCAATACGAGGTCGCCAACATCTTCTTCTGGAGGCCGTGGTTGGCT
TGTATGGAGCAGCAGACGCGCTACTTCGAGCGGAGGCATCCGGAGCTTGC
AGGATCGCCGCGGCTCCGGGCGTATATGCTCCGCATTGGTCTTGACCAAC
TCTATCAGAGCTTGGTTGACGGCAATTTCGATGATGCAGCTTGGGCGCAG
GGTCGATGCGACGCAATCGTCCGATCCGGAGCCGGGACTGTCGGGCGTAC
ACAAATCGCCCGCAGAAGCGCGGCCGTCTGGACCGATGGCTGTGTAGAAG
TACTCGCCGATAGTGGAAACCGACGCCCCAGCACTCGTCCGGATCGGGAG
ATGGGGGAGGCTAACTAAGCGGCCGCATCAATTTGATTTCTTCTTAATTC
AATGCTAAAAAAAAAAATATTATAAAAAAAAAAACACACACAACAAATAA
ATTAATAAATACCATTTATTAAAAAAAAAAAAAAAAAAAAAAAACCTATT
TTAACAATTATTTTATTATTTTTATTTTTAGTTTTGTTAAAAAAAAAATT
ATTATCTTTATTTATAATTATAAAAAATTTATCCAATGATCATTTTTTAA
CTTTTTTATTATTTAGTATATTAAATAAAATCTCGAGAGTAGTATAACTT
CGTATAGCATACATTATACGAAGTTATGATATCTCATCTAGATCAGTCGA
CTCAGCCGGCTCAGAGCTCCAGCTTTTGTTCCCTTTAGTGAGGGTTAATT
GCGCGCTTGGCGTAATCATGGTCATAGCTGTTTCCTGTGTGAAATTGTTA
TCCGCTCACAATTCCACACAACATACGAGCCGGAAGCATAAAGTGTAAAG
CCTGGGGTGCCTAATGAGTGAGCTAACTCACATTAATTGCGTTGCGCTCA
CTGCCCGCTTTCCAGTCGGGAAACCTGTCGTGCCAGCTGCATTAATGAAT
CGGCCAACGCGCGGGGAGAGGCGGTTTGCGTATTGGGCGCTCTTCCGCTT
CCTCGCTCACTGACTCGCTGCGCTCGGTCGTTCGGCTGCGGCGAGCGGTA
TCAGCTCACTCAAAGGCGGTAATACGGTTATCCACAGAATCAGGGGATAA
CGCAGGAAAGAACATGTGAGCAAAAGGCCAGCAAAAGGCCAGGAACCGTA
AAAAGGCCGCGTTGCTGGCGTTTTTCCATAGGCTCCGCCCCCCTGACGAG
CATCACAAAAATCGACGCTCAAGTCAGAGGTGGCGAAACCCGACAGGACT
ATAAAGATACCAGGCGTTTCCCCCTGGAAGCTCCCTCGTGCGCTCTCCTG
TTCCGACCCTGCCGCTTACCGGATACCTGTCCGCCTTTCTCCCTTCGGGA
AGCGTGGCGCTTTCTCATAGCTCACGCTGTAGGTATCTCAGTTCGGTGTA
GGTCGTTCGCTCCAAGCTGGGCTGTGTGCACGAACCCCCCGTTCAGCCCG
ACCGCTGCGCCTTATCCGGTAACTATCGTCTTGAGTCCAACCCGGTAAGA
CACGACTTATCGCCACTGGCAGCAGCCACTGGTAACAGGATTAGCAGAGC
GAGGTATGTAGGCGGTGCTACAGAGTTCTTGAAGTGGTGGCCTAACTACG

GCTACACTAGAAGGACAGTATTTGGTATCTGCGCTCTGCTGAAGCCAGTT
ACCTTCGGAAAAAGAGTTGGTAGCTCTTGATCCGGCAAACAAACCACCGC
TGGTAGCGGTGGTTTTTTTGTTTGCAAGCAGCAGATTACGCGCAGAAAAA
AAGGATCTCAAGAAGATCCTTTGATCTTTTCTACGGGGTCTGACGCTCAG
TGGAACGAAAACTCACGTTAAGGGATTTTGGTCATGAGATTATCAAAAAG
GATCTTCACCTAGATCCTTTTAAATTAAAAATGAAGTTTTAAATCAATCT
AAAGTATATATGAGTAAACTTGGTCTGACAGTTACCAATGCTTAATCAGT
GAGGCACCTATCTCAGCGATCTGTCTATTTCGTTCATCCATAGTTGCCTG
ACTCCCCGTCGTGTAGATAACTACGATACGGGAGGGCTTACCATCTGGCC
CCAGTGCTGCAATGATACCGCGAGACCCACGCTCACCGGCTCCAGATTTA
TCAGCAATAAACCAGCCAGCCGGAAGGGCCGAGCGCAGAAGTGGTCCTGC
AACTTTATCCGCCTCCATCCAGTCTATTAATTGTTGCCGGGAAGCTAGAG
TAAGTAGTTCGCCAGTTAATAGTTTGCGCAACGTTGTTGCCATTGCTACA
GGCATCGTGGTGTCACGCTCGTCGTTTGGTATGGCTTCATTCAGCTCCGG
TTCCCAACGATCAAGGCGAGTTACATGATCCCCCATGTTGTGCAAAAAAG
CGGTTAGCTCCTTCGGTCCTCCGATCGTTGTCAGAAGTAAGTTGGCCGCA
GTGTTATCACTCATGGTTATGGCAGCACTGCATAATTCTCTTACTGTCAT
GCCATCCGTAAGATGCTTTTCTGTGACTGGTGAGTACTCAACCAAGTCAT
TCTGAGAATAGTGTATGCGGCGACCGAGTTGCTCTTGCCCGGCGTCAATA
CGGGATAATACCGCGCCACATAGCAGAACTTTAAAAGTGCTCATCATTGG
AAAACGTTCTTCGGGGCGAAAACTCTCAAGGATCTTACCGCTGTTGAGAT
CCAGTTCGATGTAACCCACTCGTGCACCCAACTGATCTTCAGCATCTTTT
ACTTTCACCAGCGTTTCTGGGTGAGCAAAAACAGGAAGGCAAAATGCCGC
AAAAAAGGGAATAAGGGCGACACGGAAATGTTGAATACTCATACTCTTCC
TTTTTCAATATTATTGAAGCATTTATCAGGGTTATTGTCTCATGAGCGGA
TACATATTTGAATGTATTTAGAAAAATAAACAAATAGGGGTTCCGCGCAC
ATTTCCCCGAAAAGTGCCAC

pPI192 – mCherry (C) Hyg

CTAAATTGTAAGCGTTAATATTTTGTTAAAATTCGCGTTAAATTTTTGTT
AAATCAGCTCATTTTTTAACCAATAGGCCGAAATCGGCAAAATCCCTTAT
AAATCAAAAGAATAGACCGAGATAGGGTTGAGTGTTGTTCCAGTTTGGAA
CAAGAGTCCACTATTAAAGAACGTGGACTCCAACGTCAAAGGGCGAAAAA
CCGTCTATCAGGGCGATGGCCCACTACGTGAACCATCACCCTAATCAAGT
TTTTTGGGGTCGAGGTGCCGTAAAGCACTAAATCGGAACCCTAAAGGGAG
CCCCCGATTTAGAGCTTGACGGGGAAAGCCGGCGAACGTGGCGAGAAAGG
AAGGGAAGAAAGCGAAAGGAGCGGGCGCTAGGGCGCTGGCAAGTGTAGCG
GTCACGCTGCGCGTAACCACCACACCCGCCGCGCTTAATGCGCCGCTACA
GGGCGCGTCCCATTCGCCATTCAGGCTGCGCAACTGTTGGGAAGGGCGAT
CGGTGCGGGCCTCTTCGCTATTACGCCAGCTGGCGAAAGGGGGATGTGCT
GCAAGGCGATTAAGTTGGGTAACGCCAGGGTTTTCCCAGTCACGACGTTG
TAAAACGACGGCCAGTGAGCGCGCGTAATACGACTCACTATAGGGCGAAT
TGGGTACCGGGCCCCCCCTCGACTACGCCGGCTCAAGATCTTCAACTAGTG
GTGGTTCAGGAGGTAGTGTTTCAAAAGGTGAAGAAGATAATATGGCAATT
ATTAAAGAATTTATGAGATTTAAAGTTCATATGGAAGGTTCAGTTAATGG
TCATGAATTTGAAATTGAAGGTGAAGGTGAAGGTAGACCATATGAAGGTA
CACAAACAGCAAAATTAAAAGTTACAAAAGGTGGTCCATTACCATTTGCA
TGGGATATTTTATCACCACAATTTATGTATGGTTCAAAAGCATATGTTAA
ACATCCAGCAGATATTCCAGATTATTTAAAATTATCATTTCCAGAAGGTT

TTAAATGGGAAAGAGTTATGAATTTTGAAGATGGTGGTGTTGTTACAGTT
ACACAAGATTCATCATTACAAGATGGTGAATTTATTTATAAAGTTAAATT
AAGAGGTACAAATTTTCCATCAGATGGTCCAGTTATGCAAAAGAAAACAA
TGGGTTGGGAAGCATCATCAGAAAGAATGTATCCAGAAGATGGTGCATTA
AAAGGTGAAATTAAACAAAGATTAAAATTAAAAGATGGTGGTCATTATGA
TGCAGAAGTTAAAACAACATATAAAGCAAAAAAACCAGTTCAATTACCAG
GTGCATATAATGTTAATATTAAATTAGATATTACATCACATAATGAAGAT
TATACAATTGTTGAACAATATGAAAGAGCAGAAGGTAGACATTCAACAGG
TGGTATGGATGAATTATATAAATAAGCTAGTTAAATAAATAAATTATTTA
ATAAATAATAAAAAAACAAATTGTTGTAATAATCTAATATTTTCTTTTTT
TTTTAATTTTTTTTTTTTAAATCTTAATAATTATTAAGTTATTTTAATTT
TTTTTTTTTTTTTTTTTTTTTTTTTTTTTTTTTTTCTATCAAAAAAATCA
AATATATTTAAAAAATTTATTATTTACAGATACATTTTGAATGGTGAAGA
TAAATATATGCATTAGATGTAAAACAGCCAAAGAGTATGAAAATCAAAAA
GATAAAGCTTGATATCTTAATTAATTAAATAACTTCGTATAGCATACATT
ATACGAAGTTATAGTAGTGGATCCCAAAATTTTGTATTTTGTGATGTTAG
TGTGTGTCATAATTGGAATATTGAAAAAAAAAAAATAAAAAATAAAAAAA
AAAAAAAAAAGAATTAATTAATGATTAATCATATGACTAACATTATATTT
TCAATTTTTTTTTTTTTTTTTTTCCAACCACCCAAAAGTGATCCATTTTT
TTTTTTTTTTTAAATAAAATTTGGGTCACCCACAGTTGAATTATTTTTAA
TTTTTTTTTTTTTTTTTTTTTTTTTTAATTTTTTTTTTTTTTTTTCAAAT
TTTTTTTTCTTCATATAAAAATAAATTTTCCAATTCATTTTTTTTTTTTT
TATTCAACTGTATATTATTATAACTCTTTATTTGTATTTTAAACTAATTT
CACAGCTAGCAAAAAATGGATCAATTTAACATTTCTCAACAAGACCTAAT
GAAAAAGCCTGAACTCACCGCGACGTCTGTCGAGAAGTTTCTGATCGAAA
AGTTCGACAGCGTCTCCGACCTGATGCAGCTCTCGGAGGGCGAAGAATCT
CGTGCTTTCAGCTTCGATGTAGGAGGGCGTGGATATGTCCTGCGGGTAAA
TAGCTGCGCCGATGGTTTCTACAAAGATCGTTATGTTTATCGGCACTTTG
CATCGGCCGCGCTCCCGATTCCGGAAGTGCTTGACATTGGGGAGTTCAGC
GAGAGCCTGACCTATTGCATCTCCCGCCGTGCACAGGGTGTCACGTTGCA
AGACCTGCCTGAAACCGAACTGCCCGCTGTTCTGCAGCCGGTCGCGGAGG
CCATGGATGCGATCGCTGCGGCCGATCTTAGCCAGACGAGCGGGTTCGGC
CCATTCGGACCGCAAGGAATCGGTCAATACACTACATGGCGTGATTTCAT
ATGCGCGATTGCTGATCCCCATGTGTATCACTGGCAAACTGTGATGGACG
ACACCGTCAGTGCGTCCGTCGCGCAGGCTCTCGATGAGCTGATGCTTTGG
GCCGAGGACTGCCCCGAAGTCCGGCACCTCGTGCACGCGGATTTCGGCTC
CAACAATGTCCTGACGGACAATGGCCGCATAACAGCGGTCATTGACTGGA
GCGAGGCGATGTTCGGGGATTCCCAATACGAGGTCGCCAACATCTTCTTC
TGGAGGCCGTGGTTGGCTTGTATGGAGCAGCAGACGCGCTACTTCGAGCG
GAGGCATCCGGAGCTTGCAGGATCGCCGCGGCTCCGGGCGTATATGCTCC
GCATTGGTCTTGACCAACTCTATCAGAGCTTGGTTGACGGCAATTTCGAT
GATGCAGCTTGGGCGCAGGGTCGATGCGACGCAATCGTCCGATCCGGAGC
CGGGACTGTCGGGCGTACACAAATCGCCCGCAGAAGCGCGGCCGTCTGGA
CCGATGGCTGTGTAGAAGTACTCGCCGATAGTGGAAACCGACGCCCCAGC
ACTCGTCCGGATCGGGAGATGGGGGAGGCTAACTAAGCGGCCGCATCAAT
TTGATTTCTTCTTAATTCAATGCTAAAAAAAAAAATATTATAAAAAAAAA
AACACACACAACAAATAAATTAATAAATACCATTTATTAAAAAAAAAAAA
AAAAAAAAAAAACCTATTTTAACAATTATTTTATTATTTTTATTTTTAGT
TTTGTTAAAAAAAAAATTATTATCTTTATTTATAATTATAAAAAATTTAT
CCAATGATCATTTTTTAACTTTTTTATTATTTAGTATATTAAATAAAATC

TCGAGAGTAGTATAACTTCGTATAGCATACATTATACGAAGTTATGATAT
CTCATCTAGATCAGTCGACTCAGCCGGCTCAGAGCTCCAGCTTTTGTTCC
CTTTAGTGAGGGTTAATTGCGCGCTTGGCGTAATCATGGTCATAGCTGTT
TCCTGTGTGAAATTGTTATCCGCTCACAATTCCACACAACATACGAGCCG
GAAGCATAAAGTGTAAAGCCTGGGGTGCCTAATGAGTGAGCTAACTCACA
TTAATTGCGTTGCGCTCACTGCCCGCTTTCCAGTCGGGAAACCTGTCGTG
CCAGCTGCATTAATGAATCGGCCAACGCGCGGGGAGAGGCGGTTTGCGTA
TTGGGCGCTCTTCCGCTTCCTCGCTCACTGACTCGCTGCGCTCGGTCGTT
CGGCTGCGGCGAGCGGTATCAGCTCACTCAAAGGCGGTAATACGGTTATC
CACAGAATCAGGGGATAACGCAGGAAAGAACATGTGAGCAAAAGGCCAGC
AAAAGGCCAGGAACCGTAAAAAGGCCGCGTTGCTGGCGTTTTTCCATAGG
CTCCGCCCCCCTGACGAGCATCACAAAAATCGACGCTCAAGTCAGAGGTG
GCGAAACCCGACAGGACTATAAAGATACCAGGCGTTTCCCCCTGGAAGCT
CCCTCGTGCGCTCTCCTGTTCCGACCCTGCCGCTTACCGGATACCTGTCC
GCCTTTCTCCCTTCGGGAAGCGTGGCGCTTTCTCATAGCTCACGCTGTAG
GTATCTCAGTTCGGTGTAGGTCGTTCGCTCCAAGCTGGGCTGTGTGCACG
AACCCCCCGTTCAGCCCGACCGCTGCGCCTTATCCGGTAACTATCGTCTT
GAGTCCAACCCGGTAAGACACGACTTATCGCCACTGGCAGCAGCCACTGG
TAACAGGATTAGCAGAGCGAGGTATGTAGGCGGTGCTACAGAGTTCTTGA
AGTGGTGGCCTAACTACGGCTACACTAGAAGGACAGTATTTGGTATCTGC
GCTCTGCTGAAGCCAGTTACCTTCGGAAAAAGAGTTGGTAGCTCTTGATC
CGGCAAACAAACCACCGCTGGTAGCGGTGGTTTTTTTGTTTGCAAGCAGC
AGATTACGCGCAGAAAAAAAGGATCTCAAGAAGATCCTTTGATCTTTTCT
ACGGGGTCTGACGCTCAGTGGAACGAAAACTCACGTTAAGGGATTTTGGT
CATGAGATTATCAAAAAGGATCTTCACCTAGATCCTTTTAAATTAAAAAT
GAAGTTTTAAATCAATCTAAAGTATATATGAGTAAACTTGGTCTGACAGT
TACCAATGCTTAATCAGTGAGGCACCTATCTCAGCGATCTGTCTATTTCG
TTCATCCATAGTTGCCTGACTCCCCGTCGTGTAGATAACTACGATACGGG
AGGGCTTACCATCTGGCCCCAGTGCTGCAATGATACCGCGAGACCCACGC
TCACCGGCTCCAGATTTATCAGCAATAAACCAGCCAGCCGGAAGGGCCGA
GCGCAGAAGTGGTCCTGCAACTTTATCCGCCTCCATCCAGTCTATTAATT
GTTGCCGGGAAGCTAGAGTAAGTAGTTCGCCAGTTAATAGTTTGCGCAAC
GTTGTTGCCATTGCTACAGGCATCGTGGTGTCACGCTCGTCGTTTGGTAT
GGCTTCATTCAGCTCCGGTTCCCAACGATCAAGGCGAGTTACATGATCCC
CCATGTTGTGCAAAAAAGCGGTTAGCTCCTTCGGTCCTCCGATCGTTGTC
AGAAGTAAGTTGGCCGCAGTGTTATCACTCATGGTTATGGCAGCACTGCA
TAATTCTCTTACTGTCATGCCATCCGTAAGATGCTTTTCTGTGACTGGTG
AGTACTCAACCAAGTCATTCTGAGAATAGTGTATGCGGCGACCGAGTTGC
TCTTGCCCGGCGTCAATACGGGATAATACCGCGCCACATAGCAGAACTTT
AAAAGTGCTCATCATTGGAAAACGTTCTTCGGGGCGAAAACTCTCAAGGA
TCTTACCGCTGTTGAGATCCAGTTCGATGTAACCCACTCGTGCACCCAAC
TGATCTTCAGCATCTTTTACTTTCACCAGCGTTTCTGGGTGAGCAAAAAC
AGGAAGGCAAAATGCCGCAAAAAAGGGAATAAGGGCGACACGGAAATGTT
GAATACTCATACTCTTCCTTTTTCAATATTATTGAAGCATTTATCAGGGT
TATTGTCTCATGAGCGGATACATATTTGAATGTATTTAGAAAAATAAACA
AATAGGGGTTCCGCGCACATTTCCCCGAAAAGTGCCAC

pPI183 – GFP (C) BS

CTAAATTGTAAGCGTTAATATTTTGTTAAAATTCGCGTTAAATTTTTGTT

AAATCAGCTCATTTTTTAACCAATAGGCCGAAATCGGCAAAATCCCTTAT
AAATCAAAAGAATAGACCGAGATAGGGTTGAGTGTTGTTCCAGTTTGGAA
CAAGAGTCCACTATTAAAGAACGTGGACTCCAACGTCAAAGGGCGAAAAA
CCGTCTATCAGGGCGATGGCCCACTACGTGAACCATCACCCTAATCAAGT
TTTTTGGGGTCGAGGTGCCGTAAAGCACTAAATCGGAACCCTAAAGGGAG
CCCCCGATTTAGAGCTTGACGGGGAAAGCCGGCGAACGTGGCGAGAAAGG
AAGGGAAGAAAGCGAAAGGAGCGGGCGCTAGGGCGCTGGCAAGTGTAGCG
GTCACGCTGCGCGTAACCACCACACCCGCCGCGCTTAATGCGCCGCTACA
GGGCGCGTCCCATTCGCCATTCAGGCTGCGCAACTGTTGGGAAGGGCGAT
CGGTGCGGGCCTCTTCGCTATTACGCCAGCTGGCGAAAGGGGGATGTGCT
GCAAGGCGATTAAGTTGGGTAACGCCAGGGTTTTCCCAGTCACGACGTTG
TAAAACGACGGCCAGTGAGCGCGCGTAATACGACTCACTATAGGGCGAAT
TGGGTACCGGGCCCCCCCTCGACTACGCCGGCTCAAGATCTTCAACTAGTG
GTGGTTCAGGAGGTAGTTCAAAAGGTGAAGAATTATTTACAGGTGTTGTT
CCAATTTTAGTTGAATTAGATGGTGATGTTAATGGTCATAAATTTTCAGT
TTCAGGTGAAGGTGAAGGTGATGCAACATATGGTAAATTAACATTAAAAT
TTATTTGTACAACAGGTAAATTACCAGTTCCATGGCCAACATTAGTTACA
ACATTTACATATGGTGTTCAATGTTTTTCAAGATATCCAGATCATATGAA
ACAACATGATTTTTTTAAATCAGCAATGCCAGAAGGTTATGTTCAAGAAA
GAACAATTTTTTTTAAAGATGATGGTAATTATAAAACAAGAGCAGAAGTT
AAATTTGAAGGTGATACATTAGTTAATAGAATTGAATTAAAAGGTATTGA
TTTTAAAGAAGATGGTAATATTTTAGGTCATAAATTAGAATATAATTATA
ATTCACATAATGTTTATATTATGGCAGATAAACAAAAAAATGGTATTAAA
GTTAATTTTAAAATTAGACATAATATTGAAGATGGTTCAGTTCAATTAGC
AGATCATTATCAACAAAATACACCAATTGGTGATGGTCCAGTTTTATTAC
CAGATAATCATTATTTATCAACACAATCAGCATTATCAAAAGATCCAAAT
GAAAAAAGAGATCATATGGTTTTATTAGAATTTGTTACAGCAGCAGGTAT
TACACATGGTATGGATGAATTATATAAATAAGCTAGTTAAATAAATAAAT
TATTTAATAAATAATAAAAAAACAAATTGTTGTAATAATCTAATATTTTC
TTTTTTTTTTAATTTTTTTTTTTTAAATCTTAATAATTATTAAGTTATTT
TAATTTTTTTTTTTTTTTTTTTTTTTTTTTTTTTTTTTTTTCTATCAAAA
AAATCAAATATATTTAAAAAATTTATTATTTACAGATACATTTTGAATGG
TGAAGATAAATATATGCATTAGATGTAAAACAGCCAAAGAGTATGAAAAT
CAAAAAGATAAAGCTTGATATCTTAATTAATTAAATAACTTCGTATAGCA
TACATTATACGAAGTTATAGTAGTGGATCCCAAAATTTTGTATTTTGTGA
TGTTAGTGTGTGTCATAATTGGAATATTGAAAAAAAAAAAATAAAAAATA
AAAAAAAAAAAAAAAAGAATTAATTAATGATTAATCATATGACTAACATT
ATATTTTCAATTTTTTTTTTTTTTTTTTTCCAACCACCCAAAAGTGATCC
ATTTTTTTTTTTTTTTTAAATAAAATTTGGGTCACCCACAGTTGAATTAT
TTTTAATTTTTTTTTTTTTTTTTTTTTTTTTTAATTTTTTTTTTTTTTTT
CAAAATTTTTTTTTCTTCATATAAAAATAAATTTTCCAATTCATTTTTTT
TTTTTTTATTCAACTGTATATTATTATAACTCTTTATTTGTATTTTAAAC
TAATTTCACAGCTAGCAAAAAATGGATCAATTTAACATTTCTCAACAGGA
TCTAGAATTAGTAGAAGTAGCGACAGAGAAGATTACAATGCTTTATGAGG
ATAATAAACATCATGTGGGAGCGGCAATTCGTACGAAAACAGGAGAAATC
ATTTCGGCAGTACATATTGAAGCGTATATAGGACGAGTAACTGTTTGTGC
AGAAGCCATTGCGATTGGTAGTGCAGTTTCGAATGGACAAAAGGATTTTG
ACACGATTGTAGCTGTTAGACACCCTTATTCTGACGAAGTAGATAGAAGT
ATTCGAGTGGTAAGTCCTTGTGGTATGTGTAGGGAGTTGATTTCAGACTA
TGCACCAGATTGTTTTGTGTTAATAGAAATGAATGGCAAGTTAGTCAAAA

CTACGATTGAAGAACTCATTCCACTCAAATATACCCGAAATTAAGCGGCC
GCATCAATTTGATTTCTTCTTAATTCAATGCTAAAAAAAAAAAATATTATA
AAAAAAAAAACACACACAACAAATAAATTAATAAATACCATTTATTAAAA
AAAAAAAAAAAAAAAAAAAACCTATTTTAACAATTATTTTATTATTTTTA
TTTTTAGTTTTGTTAAAAAAAAAATTATTATCTTTATTTATAATTATAAA
AAATTTATCCAATGATCATTTTTTAACTTTTTTATTATTTAGTATATTAA
ATAAAATCTCGAGAGTAGTATAACTTCGTATAGCATACATTATACGAAGT
TATGATATCTCATCTAGATCAGTCGACTCAGCCGGCTCAGAGCTCCAGCT
TTTGTTCCCTTTAGTGAGGGTTAATTGCGCGCTTGGCGTAATCATGGTCA
TAGCTGTTTCCTGTGTGAAATTGTTATCCGCTCACAATTCCACACAACAT
ACGAGCCGGAAGCATAAAGTGTAAAGCCTGGGGTGCCTAATGAGTGAGCT
AACTCACATTAATTGCGTTGCGCTCACTGCCCGCTTTCCAGTCGGGAAAC
CTGTCGTGCCAGCTGCATTAATGAATCGGCCAACGCGCGGGGAGAGGCGG
TTTGCGTATTGGGCGCTCTTCCGCTTCCTCGCTCACTGACTCGCTGCGCT
CGGTCGTTCGGCTGCGGCGAGCGGTATCAGCTCACTCAAAGGCGGTAATA
CGGTTATCCACAGAATCAGGGGATAACGCAGGAAAGAACATGTGAGCAAA
AGGCCAGCAAAAGGCCAGGAACCGTAAAAAGGCCGCGTTGCTGGCGTTTT
TCCATAGGCTCCGCCCCCCTGACGAGCATCACAAAAATCGACGCTCAAGT
CAGAGGTGGCGAAACCCGACAGGACTATAAAGATACCAGGCGTTTCCCCC
TGGAAGCTCCCTCGTGCGCTCTCCTGTTCCGACCCTGCCGCTTACCGGAT
ACCTGTCCGCCTTTCTCCCTTCGGGAAGCGTGGCGCTTTCTCATAGCTCA
CGCTGTAGGTATCTCAGTTCGGTGTAGGTCGTTCGCTCCAAGCTGGGCTG
TGTGCACGAACCCCCCGTTCAGCCCGACCGCTGCGCCTTATCCGGTAACT
ATCGTCTTGAGTCCAACCCGGTAAGACACGACTTATCGCCACTGGCAGCA
GCCACTGGTAACAGGATTAGCAGAGCGAGGTATGTAGGCGGTGCTACAGA
GTTCTTGAAGTGGTGGCCTAACTACGGCTACACTAGAAGGACAGTATTTG
GTATCTGCGCTCTGCTGAAGCCAGTTACCTTCGGAAAAAGAGTTGGTAGC
TCTTGATCCGGCAAACAAACCACCGCTGGTAGCGGTGGTTTTTTTGTTTG
CAAGCAGCAGATTACGCGCAGAAAAAAAGGATCTCAAGAAGATCCTTTGA
TCTTTTCTACGGGGTCTGACGCTCAGTGGAACGAAAACTCACGTTAAGGG
ATTTTGGTCATGAGATTATCAAAAAGGATCTTCACCTAGATCCTTTTAAA
TTAAAAATGAAGTTTTAAATCAATCTAAAGTATATATGAGTAAACTTGGT
CTGACAGTTACCAATGCTTAATCAGTGAGGCACCTATCTCAGCGATCTGT
CTATTTCGTTCATCCATAGTTGCCTGACTCCCCGTCGTGTAGATAACTAC
GATACGGGAGGGCTTACCATCTGGCCCCAGTGCTGCAATGATACCGCGAG
ACCCACGCTCACCGGCTCCAGATTTATCAGCAATAAACCAGCCAGCCGGA
AGGGCCGAGCGCAGAAGTGGTCCTGCAACTTTATCCGCCTCCATCCAGTC
TATTAATTGTTGCCGGGAAGCTAGAGTAAGTAGTTCGCCAGTTAATAGTT
TGCGCAACGTTGTTGCCATTGCTACAGGCATCGTGGTGTCACGCTCGTCG
TTTGGTATGGCTTCATTCAGCTCCGGTTCCCAACGATCAAGGCGAGTTAC
ATGATCCCCCATGTTGTGCAAAAAAGCGGTTAGCTCCTTCGGTCCTCCGA
TCGTTGTCAGAAGTAAGTTGGCCGCAGTGTTATCACTCATGGTTATGGCA
GCACTGCATAATTCTCTTACTGTCATGCCATCCGTAAGATGCTTTTCTGT
GACTGGTGAGTACTCAACCAAGTCATTCTGAGAATAGTGTATGCGGCGAC
CGAGTTGCTCTTGCCCGGCGTCAATACGGGATAATACCGCGCCACATAGC
AGAACTTTAAAAGTGCTCATCATTGGAAAACGTTCTTCGGGGCGAAAACT
CTCAAGGATCTTACCGCTGTTGAGATCCAGTTCGATGTAACCCACTCGTG
CACCCAACTGATCTTCAGCATCTTTTACTTTCACCAGCGTTTCTGGGTGA
GCAAAAACAGGAAGGCAAAATGCCGCAAAAAAGGGAATAAGGGCGACACG
GAAATGTTGAATACTCATACTCTTCCTTTTTCAATATTATTGAAGCATTT

ATCAGGGTTATTGTCTCATGAGCGGATACATATTTGAATGTATTTAGAAA
AATAAACAAATAGGGGTTCCGCGCACATTTCCCCGAAAAGTGCCAC

pPI177 – mNeon (C) BS

CTAAATTGTAAGCGTTAATATTTTGTTAAAATTCGCGTTAAATTTTTGTT
AAATCAGCTCATTTTTTAACCAATAGGCCGAAATCGGCAAAATCCCTTAT
AAATCAAAAGAATAGACCGAGATAGGGTTGAGTGTTGTTCCAGTTTGGAA
CAAGAGTCCACTATTAAAGAACGTGGACTCCAACGTCAAAGGGCGAAAAA
CCGTCTATCAGGGCGATGGCCCACTACGTGAACCATCACCCTAATCAAGT
TTTTTGGGGTCGAGGTGCCGTAAAGCACTAAATCGGAACCCTAAAGGGAG
CCCCCGATTTAGAGCTTGACGGGGAAAGCCGGCGAACGTGGCGAGAAAGG
AAGGGAAGAAAGCGAAAGGAGCGGGCGCTAGGGCGCTGGCAAGTGTAGCG
GTCACGCTGCGCGTAACCACCACACCCGCCGCGCTTAATGCGCCGCTACA
GGGCGCGTCCCATTCGCCATTCAGGCTGCGCAACTGTTGGGAAGGGCGAT
CGGTGCGGGCCTCTTCGCTATTACGCCAGCTGGCGAAAGGGGGATGTGCT
GCAAGGCGATTAAGTTGGGTAACGCCAGGGTTTTCCCAGTCACGACGTTG
TAAAACGACGGCCAGTGAGCGCGCGTAATACGACTCACTATAGGGCGAAT
TGGGTACCGGGCCCCCCCTCGACTACGCCGGCTCAAGATCTTCAACTAGTG
GTGGTTCAGGAGGTAGTGTGAGTAAAGGTGAAGAAGATAATATGGCATCG
TTACCAGCTACACATGAGTTACATATATTCGGTAGCATTAATGGTGTTGA
TTTTGATATGGTGGGACAAGGTACCGGTAATCCTAATGATGGTTACGAAG
AACTAAATTTAAAATCGACTAAAGGTGACTTACAATTTTCTCCATGGATT
TTAGTGCCACATATAGGGTATGGTTTTCATCAATACTTACCATATCCAGA
TGGTATGTCACCATTTCAAGCTGCAATGGTTGATGGATCAGGTTATCAAG
TTCATAGAACAATGCAATTTGAAGATGGTGCTTCATTAACTGTTAATTAT
AGATACACATATGAAGGCTCACATATTAAAGGTGAAGCTCAAGTTAAAGG
TACTGGTTTCCCAGCCGATGGCCCAGTTATGACAAATAGTTTAACAGCAG
CAGATTGGTGTAGATCCAAAAAAACTTATCCAAATGATAAAACAATTATT
TCAACTTTTAAATGGTCATATACAACCGGTAATGGTAAACGTTATCGTTC
AACAGCCCGTACAACATATACTTTTGCTAAACCAATGGCAGCTAATTATT
TAAAAAATCAACCAATGTATGTTTTTCGTAAAACAGAGTTAAAACATTCA
AAAACAGAACTTAATTTTAAAGAATGGCAAAAAGCATTTACAGACGTTAT
GTAAGCTAGTAGTTAAATAAATAAATTATTTAATAAATAATAAAAAAACA
AATTGTTGTAATAATCTAATATTTTCTTTTTTTTTTAATTTTTTTTTTTT
AAATCTTAATAATTATTAAGTTATTTTAATTTTTTTTTTTTTTTTTTTTT
TTTTTTTTTTTTTTTTTCTATCAAAAAAATCAAATATATTTAAAAAATTT
ATTATTTACAGATACATTTTGAATGGTGAAGATAAATATATGCATTAGAT
GTAAAACAGCCAAAGAGTATGAAAATCAAAAAGATAAAGCTTGATATCTT
AATTAATTAAATAACTTCGTATAGCATACATTATACGAAGTTATAGTAGT
GGATCCCAAAATTTTGTATTTTGTGATGTTAGTGTGTGTCATAATTGGAA
TATTGAAAAAAAAAAAAATAAAAAATAAAAAAAAAAAAAAAAAAGAATTAAT
TAATGATTAATCATATGACTAACATTATATTTTCAATTTTTTTTTTTTTT
TTTTTCCAACCACCCAAAAGTGATCCATTTTTTTTTTTTTTTTAAATAAA
ATTTGGGTCACCCACAGTTGAATTATTTTTAATTTTTTTTTTTTTTTTTT
TTTTTTTTAATTTTTTTTTTTTTTTTCAAAATTTTTTTTTCTTCATATAA
AAATAAATTTTCCAATTCATTTTTTTTTTTTTTTATTCAACTGTATATTAT
TATAACTCTTTATTTGTATTTTAAACTAATTTCACAGCTAGCAAAAAATG
GATCAATTTAACATTTCTCAACAGGATCTAGAATTAGTAGAAGTAGCGAC
AGAGAAGATTACAATGCTTTATGAGGATAATAAACATCATGTGGGAGCGG

CAATTCGTACGAAAACAGGAGAAATCATTTCGGCAGTACATATTGAAGCG
TATATAGGACGAGTAACTGTTTGTGCAGAAGCCATTGCGATTGGTAGTGC
AGTTTCGAATGGACAAAAGGATTTTGACACGATTGTAGCTGTTAGACACC
CTTATTCTGACGAAGTAGATAGAAGTATTCGAGTGGTAAGTCCTTGTGGT
ATGTGTAGGGAGTTGATTTCAGACTATGCACCAGATTGTTTTGTGTTAAT
AGAAATGAATGGCAAGTTAGTCAAAACTACGATTGAAGAACTCATTCCAC
TCAAATATACCCGAAATTAAGCGGCCGCATCAATTTGATTTCTTCTTAAT
TCAATGCTAAAAAAAAAAATATTATAAAAAAAAAAACACACACAACAAAT
AAATTAATAAATACCATTTATTAAAAAAAAAAAAAAAAAAAAAAAACCTA
TTTTAACAATTATTTTATTATTTTTATTTTTAGTTTTGTTAAAAAAAAAA
TTATTATCTTTATTTATAATTATAAAAAATTTATCCAATGATCATTTTTT
AACTTTTTTATTATTTAGTATATTAAATAAAATCTCGAGAGTAGTATAAC
TTCGTATAGCATACATTATACGAAGTTATGATATCTCATCTAGATCAGTC
GACTCAGCCGGCTCAGAGCTCCAGCTTTTGTTCCCTTTAGTGAGGGTTAA
TTGCGCGCTTGGCGTAATCATGGTCATAGCTGTTTCCTGTGTGAAATTGT
TATCCGCTCACAATTCCACACAACATACGAGCCGGAAGCATAAAGTGTAA
AGCCTGGGGTGCCTAATGAGTGAGCTAACTCACATTAATTGCGTTGCGCT
CACTGCCCGCTTTCCAGTCGGGAAACCTGTCGTGCCAGCTGCATTAATGA
ATCGGCCAACGCGCGGGGAGAGGCGGTTTGCGTATTGGGCGCTCTTCCGC
TTCCTCGCTCACTGACTCGCTGCGCTCGGTCGTTCGGCTGCGGCGAGCGG
TATCAGCTCACTCAAAGGCGGTAATACGGTTATCCACAGAATCAGGGGAT
AACGCAGGAAAGAACATGTGAGCAAAAGGCCAGCAAAAGGCCAGGAACCG
TAAAAAGGCCGCGTTGCTGGCGTTTTTCCATAGGCTCCGCCCCCCTGACG
AGCATCACAAAAATCGACGCTCAAGTCAGAGGTGGCGAAACCCGACAGGA
CTATAAAGATACCAGGCGTTTCCCCCTGGAAGCTCCCTCGTGCGCTCTCC
TGTTCCGACCCTGCCGCTTACCGGATACCTGTCCGCCTTTCTCCCTTCGG
GAAGCGTGGCGCTTTCTCATAGCTCACGCTGTAGGTATCTCAGTTCGGTG
TAGGTCGTTCGCTCCAAGCTGGGCTGTGTGCACGAACCCCCCGTTCAGCC
CGACCGCTGCGCCTTATCCGGTAACTATCGTCTTGAGTCCAACCCGGTAA
GACACGACTTATCGCCACTGGCAGCAGCCACTGGTAACAGGATTAGCAGA
GCGAGGTATGTAGGCGGTGCTACAGAGTTCTTGAAGTGGTGGCCTAACTA
CGGCTACACTAGAAGGACAGTATTTGGTATCTGCGCTCTGCTGAAGCCAG
TTACCTTCGGAAAAAGAGTTGGTAGCTCTTGATCCGGCAAACAAACCACC
GCTGGTAGCGGTGGTTTTTTTGTTTGCAAGCAGCAGATTACGCGCAGAAA
AAAAGGATCTCAAGAAGATCCTTTGATCTTTTCTACGGGGTCTGACGCTC
AGTGGAACGAAAACTCACGTTAAGGGATTTTGGTCATGAGATTATCAAAA
AGGATCTTCACCTAGATCCTTTTAAATTAAAAATGAAGTTTTAAATCAAT
CTAAAGTATATATGAGTAAACTTGGTCTGACAGTTACCAATGCTTAATCA
GTGAGGCACCTATCTCAGCGATCTGTCTATTTCGTTCATCCATAGTTGCC
TGACTCCCCGTCGTGTAGATAACTACGATACGGGAGGGCTTACCATCTGG
CCCCAGTGCTGCAATGATACCGCGAGACCCACGCTCACCGGCTCCAGATT
TATCAGCAATAAACCAGCCAGCCGGAAGGGCCGAGCGCAGAAGTGGTCCT
GCAACTTTATCCGCCTCCATCCAGTCTATTAATTGTTGCCGGGAAGCTAG
AGTAAGTAGTTCGCCAGTTAATAGTTTGCGCAACGTTGTTGCCATTGCTA
CAGGCATCGTGGTGTCACGCTCGTCGTTTGGTATGGCTTCATTCAGCTCC
GGTTCCCAACGATCAAGGCGAGTTACATGATCCCCCATGTTGTGCAAAAA
AGCGGTTAGCTCCTTCGGTCCTCCGATCGTTGTCAGAAGTAAGTTGGCCG
CAGTGTTATCACTCATGGTTATGGCAGCACTGCATAATTCTCTTACTGTC
ATGCCATCCGTAAGATGCTTTTCTGTGACTGGTGAGTACTCAACCAAGTC
ATTCTGAGAATAGTGTATGCGGCGACCGAGTTGCTCTTGCCCGGCGTCAA

TACGGGATAATACCGCGCCACATAGCAGAACTTTAAAAGTGCTCATCATT
GGAAAACGTTCTTCGGGGCGAAAACTCTCAAGGATCTTACCGCTGTTGAG
ATCCAGTTCGATGTAACCCACTCGTGCACCCAACTGATCTTCAGCATCTT
TTACTTTCACCAGCGTTTCTGGGTGAGCAAAAACAGGAAGGCAAAATGCC
GCAAAAAAGGGAATAAGGGCGACACGGAAATGTTGAATACTCATACTCTT
CCTTTTTCAATATTATTGAAGCATTTATCAGGGTTATTGTCTCATGAGCG
GATACATATTTGAATGTATTTAGAAAAATAAACAAATAGGGGTTCCGCGC
ACATTTCCCCGAAAAGTGCCAC

pPI184 – mCherry (C) BS

CTAAATTGTAAGCGTTAATATTTTGTTAAAATTCGCGTTAAATTTTTGTT
AAATCAGCTCATTTTTTAACCAATAGGCCGAAATCGGCAAAATCCCTTAT
AAATCAAAAGAATAGACCGAGATAGGGTTGAGTGTTGTTCCAGTTTGGAA
CAAGAGTCCACTATTAAAGAACGTGGACTCCAACGTCAAAGGGCGAAAAA
CCGTCTATCAGGGCGATGGCCCACTACGTGAACCATCACCCTAATCAAGT
TTTTTGGGGTCGAGGTGCCGTAAAGCACTAAATCGGAACCCTAAAGGGAG
CCCCCGATTTAGAGCTTGACGGGGAAAGCCGGCGAACGTGGCGAGAAAGG
AAGGGAAGAAAGCGAAAGGAGCGGGCGCTAGGGCGCTGGCAAGTGTAGCG
GTCACGCTGCGCGTAACCACCACACCCGCCGCGCTTAATGCGCCGCTACA
GGGCGCGTCCCATTCGCCATTCAGGCTGCGCAACTGTTGGGAAGGGCGAT
CGGTGCGGGCCTCTTCGCTATTACGCCAGCTGGCGAAAGGGGGATGTGCT
GCAAGGCGATTAAGTTGGGTAACGCCAGGGTTTTCCCAGTCACGACGTTG
TAAAACGACGGCCAGTGAGCGCGCGTAATACGACTCACTATAGGGCGAAT
TGGGTACCGGGCCCCCCCTCGACTACGCCGGCTCAAGATCTTCAACTAGTG
GTGGTTCAGGAGGTAGTGTTTCAAAAGGTGAAGAAGATAATATGGCAATT
ATTAAAGAATTTATGAGATTTAAAGTTCATATGGAAGGTTCAGTTAATGG
TCATGAATTTGAAATTGAAGGTGAAGGTGAAGGTAGACCATATGAAGGTA
CACAAACAGCAAAATTAAAAGTTACAAAAGGTGGTCCATTACCATTTGCA
TGGGATATTTTATCACCACAATTTATGTATGGTTCAAAAGCATATGTTAA
ACATCCAGCAGATATTCCAGATTATTTAAAATTATCATTTCCAGAAGGTT
TTAAATGGGAAAGAGTTATGAATTTTGAAGATGGTGGTGTTGTTACAGTT
ACACAAGATTCATCATTACAAGATGGTGAATTTATTTATAAAGTTAAATT
AAGAGGTACAAATTTTCCATCAGATGGTCCAGTTATGCAAAAGAAAACAA
TGGGTTGGGAAGCATCATCAGAAAGAATGTATCCAGAAGATGGTGCATTA
AAAGGTGAAATTAAACAAAGATTAAAATTAAAAGATGGTGGTCATTATGA
TGCAGAAGTTAAAACAACATATAAAGCAAAAAAACCAGTTCAATTACCAG
GTGCATATAATGTTAATATTAAATTAGATATTACATCACATAATGAAGAT
TATACAATTGTTGAACAATATGAAAGAGCAGAAGGTAGACATTCAACAGG
TGGTATGGATGAATTATATAAATAAGCTAGTTAAATAAATAAATTATTTA
ATAAATAATAAAAAAACAAATTGTTGTAATAATCTAATATTTTCTTTTTT
TTTTAATTTTTTTTTTTTTAAATCTTAATAATTATTAAGTTATTTTAATTT
TTTTTTTTTTTTTTTTTTTTTTTTTTTTTTTTTTTCTATCAAAAAAATCA
AATATATTTAAAAAATTTATTATTTACAGATACATTTTGAATGGTGAAGA
TAAATATATGCATTAGATGTAAAACAGCCAAAGAGTATGAAAATCAAAAA
GATAAAGCTTGATATCTTAATTAATTAAATAACTTCGTATAGCATACATT
ATACGAAGTTATAGTAGTGGATCCCAAAATTTTGTATTTTGTGATGTTAG
TGTGTGTCATAATTGGAATATTGAAAAAAAAAAAATAAAAAATAAAAAAA
AAAAAAAAAGAATTAATTAATGATTAATCATATGACTAACATTATATTT
TCAATTTTTTTTTTTTTTTTTTTCCAACCACCCAAAAGTGATCCATTTTT

TTTTTTTTTTTAAATAAAATTTGGGTCACCCACAGTTGAATTATTTTTAA
TTTTTTTTTTTTTTTTTTTTTTTTTTAATTTTTTTTTTTTTTTTCAAAAT
TTTTTTTTCTTCATATAAAAATAAATTTTCCAATTCATTTTTTTTTTTTT
TATTCAACTGTATATTATTATAACTCTTTATTTGTATTTTAAACTAATTT
CACAGCTAGCAAAAAATGGATCAATTTAACATTTCTCAACAGGATCTAGA
ATTAGTAGAAGTAGCGACAGAGAAGATTACAATGCTTTATGAGGATAATA
AACATCATGTGGGAGCGGCAATTCGTACGAAAACAGGAGAAATCATTTCG
GCAGTACATATTGAAGCGTATATAGGACGAGTAACTGTTTGTGCAGAAGC
CATTGCGATTGGTAGTGCAGTTTCGAATGGACAAAAGGATTTTGACACGA
TTGTAGCTGTTAGACACCCTTATTCTGACGAAGTAGATAGAAGTATTCGA
GTGGTAAGTCCTTGTGGTATGTGTAGGGAGTTGATTTCAGACTATGCACC
AGATTGTTTTGTGTTAATAGAAATGAATGGCAAGTTAGTCAAAACTACGA
TTGAAGAACTCATTCCACTCAAATATACCCGAAATTAAGCGGCCGCATCA
ATTTGATTTCTTCTTAATTCAATGCTAAAAAAAAAAAATATTATAAAAAAA
AAAACACACACAACAAATAAATTAATAAATACCATTTATTAAAAAAAAAA
AAAAAAAAAAAAAACCTATTTTAACAATTATTTTATTATTTTTATTTTTA
GTTTTGTTAAAAAAAAAATTATTATCTTTATTTATAATTATAAAAAATTT
ATCCAATGATCATTTTTTAACTTTTTTATTATTTAGTATATTAAATAAAA
TCTCGAGAGTAGTATAACTTCGTATAGCATACATTATACGAAGTTATGAT
ATCTCATCTAGATCAGTCGACTCAGCCGGCTCAGAGCTCCAGCTTTTGTT
CCCTTTAGTGAGGGTTAATTGCGCGCTTGGCGTAATCATGGTCATAGCTG
TTTCCTGTGTGAAATTGTTATCCGCTCACAATTCCACACAACATACGAGC
CGGAAGCATAAAGTGTAAAGCCTGGGGTGCCTAATGAGTGAGCTAACTCA
CATTAATTGCGTTGCGCTCACTGCCCGCTTTCCAGTCGGGAAACCTGTCG
TGCCAGCTGCATTAATGAATCGGCCAACGCGCGGGGAGAGGCGGTTTGCG
TATTGGGCGCTCTTCCGCTTCCTCGCTCACTGACTCGCTGCGCTCGGTCG
TTCGGCTGCGGCGAGCGGTATCAGCTCACTCAAAGGCGGTAATACGGTTA
TCCACAGAATCAGGGGATAACGCAGGAAAGAACATGTGAGCAAAAGGCCA
GCAAAAGGCCAGGAACCGTAAAAAGGCCGCGTTGCTGGCGTTTTTCCATA
GGCTCCGCCCCCCTGACGAGCATCACAAAAATCGACGCTCAAGTCAGAGG
TGGCGAAACCCGACAGGACTATAAAGATACCAGGCGTTTCCCCCTGGAAG
CTCCCTCGTGCGCTCTCCTGTTCCGACCCTGCCGCTTACCGGATACCTGT
CCGCCTTTCTCCCTTCGGGAAGCGTGGCGCTTTCTCATAGCTCACGCTGT
AGGTATCTCAGTTCGGTGTAGGTCGTTCGCTCCAAGCTGGGCTGTGTGCA
CGAACCCCCCGTTCAGCCCGACCGCTGCGCCTTATCCGGTAACTATCGTC
TTGAGTCCAACCCGGTAAGACACGACTTATCGCCACTGGCAGCAGCCACT
GGTAACAGGATTAGCAGAGCGAGGTATGTAGGCGGTGCTACAGAGTTCTT
GAAGTGGTGGCCTAACTACGGCTACACTAGAAGGACAGTATTTGGTATCT
GCGCTCTGCTGAAGCCAGTTACCTTCGGAAAAAGAGTTGGTAGCTCTTGA
TCCGGCAAACAAACCACCGCTGGTAGCGGTGGTTTTTTTGTTTGCAAGCA
GCAGATTACGCGCAGAAAAAAAGGATCTCAAGAAGATCCTTTGATCTTTT
CTACGGGGTCTGACGCTCAGTGGAACGAAAACTCACGTTAAGGGATTTTG
GTCATGAGATTATCAAAAAGGATCTTCACCTAGATCCTTTTAAATTAAAA
ATGAAGTTTTAAATCAATCTAAAGTATATATGAGTAAACTTGGTCTGACA
GTTACCAATGCTTAATCAGTGAGGCACCTATCTCAGCGATCTGTCTATTT
CGTTCATCCATAGTTGCCTGACTCCCCGTCGTGTAGATAACTACGATACG
GGAGGGCTTACCATCTGGCCCCAGTGCTGCAATGATACCGCGAGACCCAC
GCTCACCGGCTCCAGATTTATCAGCAATAAACCAGCCAGCCGGAAGGGCC
GAGCGCAGAAGTGGTCCTGCAACTTTATCCGCCTCCATCCAGTCTATTAA
TTGTTGCCGGGAAGCTAGAGTAAGTAGTTCGCCAGTTAATAGTTTGCGCA

ACGTTGTTGCCATTGCTACAGGCATCGTGGTGTCACGCTCGTCGTTTGGT
ATGGCTTCATTCAGCTCCGGTTCCCAACGATCAAGGCGAGTTACATGATC
CCCCATGTTGTGCAAAAAAGCGGTTAGCTCCTTCGGTCCTCCGATCGTTG
TCAGAAGTAAGTTGGCCGCAGTGTTATCACTCATGGTTATGGCAGCACTG
CATAATTCTCTTACTGTCATGCCATCCGTAAGATGCTTTTCTGTGACTGG
TGAGTACTCAACCAAGTCATTCTGAGAATAGTGTATGCGGCGACCGAGTT
GCTCTTGCCCGGCGTCAATACGGGATAATACCGCGCCACATAGCAGAACT
TTAAAAGTGCTCATCATTGGAAAACGTTCTTCGGGGCGAAAACTCTCAAG
GATCTTACCGCTGTTGAGATCCAGTTCGATGTAACCCACTCGTGCACCCA
ACTGATCTTCAGCATCTTTTACTTTCACCAGCGTTTCTGGGTGAGCAAAA
ACAGGAAGGCAAAATGCCGCAAAAAAGGGAATAAGGGCGACACGGAAATG
TTGAATACTCATACTCTTCCTTTTTCAATATTATTGAAGCATTTATCAGG
GTTATTGTCTCATGAGCGGATACATATTTGAATGTATTTAGAAAAATAAA
CAAATAGGGGTTCCGCGCACATTTCCCCGAAAAGTGCCAC

pPI180 – GFP (C) G418

CTAAATTGTAAGCGTTAATATTTTGTTAAAATTCGCGTTAAATTTTTGTT
AAATCAGCTCATTTTTTAACCAATAGGCCGAAATCGGCAAAATCCCTTAT
AAATCAAAAGAATAGACCGAGATAGGGTTGAGTGTTGTTCCAGTTTGGAA
CAAGAGTCCACTATTAAAGAACGTGGACTCCAACGTCAAAGGGCGAAAAA
CCGTCTATCAGGGCGATGGCCCACTACGTGAACCATCACCCTAATCAAGT
TTTTTGGGGTCGAGGTGCCGTAAAGCACTAAATCGGAACCCTAAAGGGAG
CCCCCGATTTAGAGCTTGACGGGGAAAGCCGGCGAACGTGGCGAGAAAGG
AAGGGAAGAAAGCGAAAGGAGCGGGCGCTAGGGCGCTGGCAAGTGTAGCG
GTCACGCTGCGCGTAACCACCACACCCGCCGCGCTTAATGCGCCGCTACA
GGGCGCGTCCCATTCGCCATTCAGGCTGCGCAACTGTTGGGAAGGGCGAT
CGGTGCGGGCCTCTTCGCTATTACGCCAGCTGGCGAAAGGGGGATGTGCT
GCAAGGCGATTAAGTTGGGTAACGCCAGGGTTTTCCCAGTCACGACGTTG
TAAAACGACGGCCAGTGAGCGCGCGTAATACGACTCACTATAGGGCGAAT
TGGGTACCGGGCCCCCCCTCGACTACGCCGGCTCAAGATCTTCAACTAGTG
GTGGTTCAGGAGGTAGTTCAAAAGGTGAAGAATTATTTACAGGTGTTGTT
CCAATTTTAGTTGAATTAGATGGTGATGTTAATGGTCATAAATTTTCAGT
TTCAGGTGAAGGTGAAGGTGATGCAACATATGGTAAATTAACATTAAAAT
TTATTTGTACAACAGGTAAATTACCAGTTCCATGGCCAACATTAGTTACA
ACATTTACATATGGTGTTCAATGTTTTTCAAGATATCCAGATCATATGAA
ACAACATGATTTTTTTAAATCAGCAATGCCAGAAGGTTATGTTCAAGAAA
GAACAATTTTTTTTAAAGATGATGGTAATTATAAAACAAGAGCAGAAGTT
AAATTTGAAGGTGATACATTAGTTAATAGAATTGAATTAAAAGGTATTGA
TTTTAAAGAAGATGGTAATATTTTAGGTCATAAATTAGAATATAATTATA
ATTCACATAATGTTTATATTATGGCAGATAAACAAAAAAATGGTATTAAA
GTTAATTTTAAAATTAGACATAATATTGAAGATGGTTCAGTTCAATTAGC
AGATCATTATCAACAAAATACACCAATTGGTGATGGTCCAGTTTTATTAC
CAGATAATCATTATTTATCAACACAATCAGCATTATCAAAAGATCCAAAT
GAAAAAAGAGATCATATGGTTTTATTAGAATTTGTTACAGCAGCAGGTAT
TACACATGGTATGGATGAATTATATAAATAAGCTAGTTAAATAAATAAAT
TATTTAATAAATAATAAAAAAACAAATTGTTGTAATAATCTAATATTTTC
TTTTTTTTTTAATTTTTTTTTTTTAAATCTTAATAATTATTAAGTTATTT
TAATTTTTTTTTTTTTTTTTTTTTTTTTTTTTTTTTTTTTTTCTATCAAAA
AAATCAAATATATTTAAAAAATTTATTATTTACAGATACATTTTGAATGG

TGAAGATAAATATATGCATTAGATGTAAAACAGCCAAAGAGTATGAAAAT
CAAAAAGATAAAGCTTGATATCTTAATTAATTAAATAACTTCGTATAGCA
TACATTATACGAAGTTATAGTAGTGGATCCCAAAATTTTGTATTTTGTGA
TGTTAGTGTGTGTCATAATTGGAATATTGAAAAAAAAAAAATAAAAAATA
AAAAAAAAAAAAAAAAGAATTAATTAATGATTAATCATATGACTAACATT
ATATTTCAATTTTTTTTTTTTTTTTTTTTCCAACCACCCAAAAGTGATCC
ATTTTTTTTTTTTTTTTAAATAAAATTTGGGTCACCCACAGTTGAATTAT
TTTTAATTTTTTTTTTTTTTTTTTTTTTTTTTAATTTTTTTTTTTTTTTT
CAAAATTTTTTTTTCTTCATATAAAAATAAATTTTCCAATTCATTTTTTT
TTTTTTTATTCAACTGTATATTATTATAACTCTTTATTTGTATTTTAAAC
TAATTTCACAGCTAGCAAAAAATGGATGGTGAAGATGTTCAAGCTGGATC
GTTTCGCATGATTGAACAAGATGGATTGCACGCAGGTTCTCCGGCCGCTT
GGGTGGAGAGGCTATTCGGCTATGACTGGGCACAACAGACAATCGGCTGC
TCTGATGCCGCCGTGTTCCGGCTGTCAGCGCAGGGGCGCCCGGTTCTTTT
TGTCAAGACCGACCTGTCCGGTGCCCTGAATGAACTGCAGGACGAGGCAG
CGCGGCTATCGTGGCTGGCCACGACGGGCGTTCCTTGCGCAGCTGTGCTC
GACGTTGTCACTGAAGCGGGAAGGGACTGGCTGCTATTGGGCGAAGTGCC
GGGGCAGGATCTCCTGTCATCTCACCTTGCTCCTGCCGAGAAAGTATCCA
TCATGGCTGATGCAATGCGGCGGCTGCATACGCTTGATCCGGCTACCTGC
CCATTCGACCACCAAGCGAAACATCGCATCGAGCGAGCACGTACTCGGAT
GGAAGCCGGTCTTGTCGATCAGGATGATCTGGACGAAGAGCATCAGGGGC
TCGCGCCAGCCGAACTGTTCGCCAGGCTCAAGGCGCGCATGCCCGACGGC
GAGGATCTCGTCGTGACCCATGGCGATGCCTGCTTGCCGAATATCATGGT
GGAAAATGGCCGCTTTTCTGGATTCATCGACTGTGGACGGCTGGGTGTGG
CGGACCGCTATCAGGACATAGCGTTGGCTACCCGTGATATTGCTGAAGAG
CTTGGCGGCGAATGGGCTGACCGCTTCCTCGTGCTTTACGGTATCGCCGC
TCCCGATTCGCAGCGCATCGCCTTCTATCGCCTTCTTGACGAGTTCTTCT
GAGCGGCCGCATCAATTTGATTTCTTCTTAATTCAATGCTAAAAAAAAAA
ATATTATAAAAAAAAAAACACACACAACAAATAAATTAATAAATACCATT
TATTAAAAAAAAAAAAAAAAAAAAAAAACCTATTTTAACAATTATTTTAT
TATTTTTATTTTTAGTTTTGTTAAAAAAAAAATTATTATCTTTATTTATA
ATTATAAAAAATTTATCCAATGATCATTTTTTAACTTTTTTATTATTTAG
TATATTAAATAAAATCTCGAGAGTAGTATAACTTCGTATAGCATACATTA
TACGAAGTTATGATATCTCATCTAGATCAGTCGACTCAGCCGGCTCAGAG
CTCCAGCTTTTGTTCCCTTTAGTGAGGGTTAATTGCGCGCTTGGCGTAAT
CATGGTCATAGCTGTTTCCTGTGTGAAATTGTTATCCGCTCACAATTCCA
CACAACATACGAGCCGGAAGCATAAAGTGTAAAGCCTGGGGTGCCTAATG
AGTGAGCTAACTCACATTAATTGCGTTGCGCTCACTGCCCGCTTTCCAGT
CGGGAAACCTGTCGTGCCAGCTGCATTAATGAATCGGCCAACGCGCGGGG
AGAGGCGGTTTGCGTATTGGGCGCTCTTCCGCTTCCTCGCTCACTGACTC
GCTGCGCTCGGTCGTTCGGCTGCGGCGAGCGGTATCAGCTCACTCAAAGG
CGGTAATACGGTTATCCACAGAATCAGGGGATAACGCAGGAAAGAACATG
TGAGCAAAAGGCCAGCAAAAGGCCAGGAACCGTAAAAAGGCCGCGTTGCT
GGCGTTTTTCCATAGGCTCCGCCCCCCTGACGAGCATCACAAAAATCGAC
GCTCAAGTCAGAGGTGGCGAAACCCGACAGGACTATAAAGATACCAGGCG
TTTCCCCCTGGAAGCTCCCTCGTGCGCTCTCCTGTTCCGACCCTGCCGCT
TACCGGATACCTGTCCGCCTTTCTCCCTTCGGGAAGCGTGGCGCTTTCTC
ATAGCTCACGCTGTAGGTATCTCAGTTCGGTGTAGGTCGTTCGCTCCAAG
CTGGGCTGTGTGCACGAACCCCCCGTTCAGCCCGACCGCTGCGCCTTATC
CGGTAACTATCGTCTTGAGTCCAACCCGGTAAGACACGACTTATCGCCAC

TGGCAGCAGCCACTGGTAACAGGATTAGCAGAGCGAGGTATGTAGGCGGT
GCTACAGAGTTCTTGAAGTGGTGGCCTAACTACGGCTACACTAGAAGGAC
AGTATTTGGTATCTGCGCTCTGCTGAAGCCAGTTACCTTCGGAAAAAGAG
TTGGTAGCTCTTGATCCGGCAAACAAACCACCGCTGGTAGCGGTGGTTTT
TTTGTTTGCAAGCAGCAGATTACGCGCAGAAAAAAAGGATCTCAAGAAGA
TCCTTTGATCTTTTCTACGGGGTCTGACGCTCAGTGGAACGAAAACTCAC
GTTAAGGGATTTTGGTCATGAGATTATCAAAAAGGATCTTCACCTAGATC
CTTTTAAATTAAAAATGAAGTTTTAAATCAATCTAAAGTATATATGAGTA
AACTTGGTCTGACAGTTACCAATGCTTAATCAGTGAGGCACCTATCTCAG
CGATCTGTCTATTTCGTTCATCCATAGTTGCCTGACTCCCCGTCGTGTAG
ATAACTACGATACGGGAGGGCTTACCATCTGGCCCCAGTGCTGCAATGAT
ACCGCGAGACCCACGCTCACCGGCTCCAGATTTATCAGCAATAAACCAGC
CAGCCGGAAGGGCCGAGCGCAGAAGTGGTCCTGCAACTTTATCCGCCTCC
ATCCAGTCTATTAATTGTTGCCGGGAAGCTAGAGTAAGTAGTTCGCCAGT
TAATAGTTTGCGCAACGTTGTTGCCATTGCTACAGGCATCGTGGTGTCAC
GCTCGTCGTTTGGTATGGCTTCATTCAGCTCCGGTTCCCAACGATCAAGG
CGAGTTACATGATCCCCCATGTTGTGCAAAAAAGCGGTTAGCTCCTTCGG
TCCTCCGATCGTTGTCAGAAGTAAGTTGGCCGCAGTGTTATCACTCATGG
TTATGGCAGCACTGCATAATTCTCTTACTGTCATGCCATCCGTAAGATGC
TTTTCTGTGACTGGTGAGTACTCAACCAAGTCATTCTGAGAATAGTGTAT
GCGGCGACCGAGTTGCTCTTGCCCGGCGTCAATACGGGATAATACCGCGC
CACATAGCAGAACTTTAAAAGTGCTCATCATTGGAAAACGTTCTTCGGGG
CGAAAACTCTCAAGGATCTTACCGCTGTTGAGATCCAGTTCGATGTAACC
CACTCGTGCACCCAACTGATCTTCAGCATCTTTTACTTTCACCAGCGTTT
CTGGGTGAGCAAAAACAGGAAGGCAAAATGCCGCAAAAAAGGGAATAAGG
GCGACACGGAAATGTTGAATACTCATACTCTTCCTTTTTCAATATTATTG
AAGCATTTATCAGGGTTATTGTCTCATGAGCGGATACATATTTGAATGTA
TTTAGAAAAATAAACAAATAGGGGTTCCGCGCACATTTCCCCGAAAAGTG
CCAC

pPI177 – mNeon (C) G418

CTAAATTGTAAGCGTTAATATTTTGTTAAAATTCGCGTTAAATTTTTGTT
AAATCAGCTCATTTTTTAACCAATAGGCCGAAATCGGCAAAATCCCTTAT
AAATCAAAAGAATAGACCGAGATAGGGTTGAGTGTTGTTCCAGTTTGGAA
CAAGAGTCCACTATTAAAGAACGTGGACTCCAACGTCAAAGGGCGAAAAA
CCGTCTATCAGGGCGATGGCCCACTACGTGAACCATCACCCTAATCAAGT
TTTTTGGGGTCGAGGTGCCGTAAAGCACTAAATCGGAACCCTAAAGGGAG
CCCCCGATTTAGAGCTTGACGGGGAAAGCCGGCGAACGTGGCGAGAAAGG
AAGGGAAGAAAGCGAAAGGAGCGGGCGCTAGGGCGCTGGCAAGTGTAGCG
GTCACGCTGCGCGTAACCACCACACCCGCCGCGCTTAATGCGCCGCTACA
GGGCGCGTCCCATTCGCCATTCAGGCTGCGCAACTGTTGGGAAGGGCGAT
CGGTGCGGGCCTCTTCGCTATTACGCCAGCTGGCGAAAGGGGGATGTGCT
GCAAGGCGATTAAGTTGGGTAACGCCAGGGTTTTCCCAGTCACGACGTTG
TAAAACGACGGCCAGTGAGCGCGCGTAATACGACTCACTATAGGGCGAAT
TGGGTACCGGGCCCCCCCTCGACTACGCCGGCTCAAGATCTTCAACTAGTG
GTGGTTCAGGAGGTAGTGTGAGTAAAGGTGAAGAAGATAATATGGCATCG
TTACCAGCTACACATGAGTTACATATATTCGGTAGCATTAATGGTGTTGA
TTTTGATATGGTGGGACAAGGTACCGGTAATCCTAATGATGGTTACGAAG
AACTAAATTTAAAATCGACTAAAGGTGACTTACAATTTTCTCCATGGATT

TTAGTGCCACATATAGGGTATGGTTTTCATCAATACTTACCATATCCAGA
TGGTATGTCACCATTTCAAGCTGCAATGGTTGATGGATCAGGTTATCAAG
TTCATAGAACAATGCAATTTGAAGATGGTGCTTCATTAACTGTTAATTAT
AGATACACATATGAAGGCTCACATATTAAAGGTGAAGCTCAAGTTAAAGG
TACTGGTTTCCCAGCCGATGGCCCAGTTATGACAAATAGTTTAACAGCAG
CAGATTGGTGTAGATCCAAAAAAACTTATCCAAATGATAAAACAATTATT
TCAACTTTTAAATGGTCATATACAACCGGTAATGGTAAACGTTATCGTTC
AACAGCCCGTACAACATATACTTTTGCTAAACCAATGGCAGCTAATTATT
TAAAAAATCAACCAATGTATGTTTTTCGTAAAACAGAGTTAAAACATTCA
AAAACAGAACTTAATTTTAAAGAATGGCAAAAAGCATTTACAGACGTTAT
GTAAGCTAGTAGTTAAATAAATAAATTATTTAATAAATAATAAAAAAACA
AATTGTTGTAATAATCTAATATTTTCTTTTTTTTTTAATTTTTTTTTTTT
AAATCTTAATAATTATTAAGTTATTTTAATTTTTTTTTTTTTTTTTTTT
TTTTTTTTTTTTTTTTCTATCAAAAAAATCAAATATATTTAAAAAATTT
ATTATTTACAGATACATTTTGAATGGTGAAGATAAATATATGCATTAGAT
GTAAAACAGCCAAAGAGTATGAAAATCAAAAAGATAAAGCTTGATATCTT
AATTAATTAAATAACTTCGTATAGCATACATTATACGAAGTTATAGTAGT
GGATCCCAAAATTTTGTATTTTGTGATGTTAGTGTGTGTCATAATTGGAA
TATTGAAAAAAAAAAAATAAAAAATAAAAAAAAAAAAAAAAAGAATTAAT
TAATGATTAATCATATGACTAACATTATATTTTCAATTTTTTTTTTTTTT
TTTTTCCAACCACCCAAAAGTGATCCATTTTTTTTTTTTTTTTAAATAAA
ATTTGGGTCACCCACAGTTGAATTATTTTTAATTTTTTTTTTTTTTTTTT
TTTTTTTTAATTTTTTTTTTTTTTTTTCAAAATTTTTTTTTCTTCATATAA
AAATAAATTTTCCAATTCATTTTTTTTTTTTTTTATTCAACTGTATATTAT
TATAACTCTTTATTTGTATTTTAAACTAATTTCACAGCTAGCAAAAAATG
GATCAATTTAACATTTCTCAACAGGATCTAGAATTAGTAGAAGTAGCGAC
AGAGAAGATTACAATGCTTTATGAGGATAATAAACATCATGTGGGAGCGG
CAATTCGTACGAAAACAGGAGAAATCATTTCGGCAGTACATATTGAAGCG
TATATAGGACGAGTAACTGTTTGTGCAGAAGCCATTGCGATTGGTAGTGC
AGTTTCGAATGGACAAAAGGATTTTGACACGATTGTAGCTGTTAGACACC
CTTATTCTGACGAAGTAGATAGAAGTATTCGAGTGGTAAGTCCTTGTGGT
ATGTGTAGGGAGTTGATTTCAGACTATGCACCAGATTGTTTTGTGTTAAT
AGAAATGAATGGCAAGTTAGTCAAAACTACGATTGAAGAACTCATTCCAC
TCAAATATACCCGAAATTAAGCGGCCGCATCAATTTGATTTCTTCTTAAT
TCAATGCTAAAAAAAAAAAATATTATAAAAAAAAAAAACACACACAACAAAT
AAATTAATAAATACCATTTATTAAAAAAAAAAAAAAAAAAAAAAAAACCTA
TTTTAACAATTATTTTATTATTTTTATTTTTAGTTTTGTTAAAAAAAAAA
TTATTATCTTTATTTATAATTATAAAAAATTTATCCAATGATCATTTTTT
AACTTTTTTATTATTTAGTATATTAAATAAAATCTCGAGAGTAGTATAAC
TTCGTATAGCATACATTATACGAAGTTATGATATCTCATCTAGATCAGTC
GACTCAGCCGGCTCAGAGCTCCAGCTTTTGTTCCCTTTAGTGAGGGTTAA
TTGCGCGCTTGGCGTAATCATGGTCATAGCTGTTTCCTGTGTGAAATTGT
TATCCGCTCACAATTCCACACAACATACGAGCCGGAAGCATAAAGTGTAA
AGCCTGGGGTGCCTAATGAGTGAGCTAACTCACATTAATTGCGTTGCGCT
CACTGCCCGCTTTCCAGTCGGGAAACCTGTCGTGCCAGCTGCATTAATGA
ATCGGCCAACGCGCGGGGAGAGGCGGTTTGCGTATTGGGCGCTCTTCCGC
TTCCTCGCTCACTGACTCGCTGCGCTCGGTCGTTCGGCTGCGGCGAGCGG
TATCAGCTCACTCAAAGGCGGTAATACGGTTATCCACAGAATCAGGGGAT
AACGCAGGAAAGAACATGTGAGCAAAAGGCCAGCAAAAGGCCAGGAACCG
TAAAAAGGCCGCGTTGCTGGCGTTTTTCCATAGGCTCCGCCCCCCTGACG

AGCATCACAAAAATCGACGCTCAAGTCAGAGGTGGCGAAACCCGACAGGA
CTATAAAGATACCAGGCGTTTCCCCCTGGAAGCTCCCTCGTGCGCTCTCC
TGTTCCGACCCTGCCGCTTACCGGATACCTGTCCGCCTTTCTCCCTTCGG
GAAGCGTGGCGCTTTCTCATAGCTCACGCTGTAGGTATCTCAGTTCGGTG
TAGGTCGTTCGCTCCAAGCTGGGCTGTGTGCACGAACCCCCCGTTCAGCC
CGACCGCTGCGCCTTATCCGGTAACTATCGTCTTGAGTCCAACCCGGTAA
GACACGACTTATCGCCACTGGCAGCAGCCACTGGTAACAGGATTAGCAGA
GCGAGGTATGTAGGCGGTGCTACAGAGTTCTTGAAGTGGTGGCCTAACTA
CGGCTACACTAGAAGGACAGTATTTGGTATCTGCGCTCTGCTGAAGCCAG
TTACCTTCGGAAAAAGAGTTGGTAGCTCTTGATCCGGCAAACAAACCACC
GCTGGTAGCGGTGGTTTTTTTGTTTGCAAGCAGCAGATTACGCGCAGAAA
AAAAGGATCTCAAGAAGATCCTTTGATCTTTTCTACGGGGTCTGACGCTC
AGTGGAACGAAAACTCACGTTAAGGGATTTTGGTCATGAGATTATCAAAA
AGGATCTTCACCTAGATCCTTTTAAATTAAAAATGAAGTTTTAAATCAAT
CTAAAGTATATATGAGTAAACTTGGTCTGACAGTTACCAATGCTTAATCA
GTGAGGCACCTATCTCAGCGATCTGTCTATTTCGTTCATCCATAGTTGCC
TGACTCCCCGTCGTGTAGATAACTACGATACGGGAGGGCTTACCATCTGG
CCCCAGTGCTGCAATGATACCGCGAGACCCACGCTCACCGGCTCCAGATT
TATCAGCAATAAACCAGCCAGCCGGAAGGGCCGAGCGCAGAAGTGGTCCT
GCAACTTTATCCGCCTCCATCCAGTCTATTAATTGTTGCCGGGAAGCTAG
AGTAAGTAGTTCGCCAGTTAATAGTTTGCGCAACGTTGTTGCCATTGCTA
CAGGCATCGTGGTGTCACGCTCGTCGTTTGGTATGGCTTCATTCAGCTCC
GGTTCCCAACGATCAAGGCGAGTTACATGATCCCCCATGTTGTGCAAAAA
AGCGGTTAGCTCCTTCGGTCCTCCGATCGTTGTCAGAAGTAAGTTGGCCG
CAGTGTTATCACTCATGGTTATGGCAGCACTGCATAATTCTCTTACTGTC
ATGCCATCCGTAAGATGCTTTTCTGTGACTGGTGAGTACTCAACCAAGTC
ATTCTGAGAATAGTGTATGCGGCGACCGAGTTGCTCTTGCCCGGCGTCAA
TACGGGATAATACCGCGCCACATAGCAGAACTTTAAAAGTGCTCATCATT
GGAAAACGTTCTTCGGGGCGAAAACTCTCAAGGATCTTACCGCTGTTGAG
ATCCAGTTCGATGTAACCCACTCGTGCACCCAACTGATCTTCAGCATCTT
TTACTTTCACCAGCGTTTCTGGGTGAGCAAAAACAGGAAGGCAAAATGCC
GCAAAAAAGGGAATAAGGGCGACACGGAAATGTTGAATACTCATACTCTT
CCTTTTTCAATATTATTGAAGCATTTATCAGGGTTATTGTCTCATGAGCG
GATACATATTTGAATGTATTTAGAAAAATAAACAAATAGGGGTTCCGCGC
ACATTTCCCCGAAAAGTGCCAC

pPI181 – mCherry (C) G418

CTAAATTGTAAGCGTTAATATTTTGTTAAAATTCGCGTTAAATTTTTGTT
AAATCAGCTCATTTTTTAACCAATAGGCCGAAATCGGCAAAATCCCTTAT
AAATCAAAAGAATAGACCGAGATAGGGTTGAGTGTTGTTCCAGTTTGGAA
CAAGAGTCCACTATTAAAGAACGTGGACTCCAACGTCAAAGGGCGAAAAA
CCGTCTATCAGGGCGATGGCCCACTACGTGAACCATCACCCTAATCAAGT
TTTTTGGGGTCGAGGTGCCGTAAAGCACTAAATCGGAACCCTAAAGGGAG
CCCCCGATTTAGAGCTTGACGGGGAAAGCCGGCGAACGTGGCGAGAAAGG
AAGGGAAGAAAGCGAAAGGAGCGGGCGCTAGGGCGCTGGCAAGTGTAGCG
GTCACGCTGCGCGTAACCACCACACCCGCCGCGCTTAATGCGCCGCTACA
GGGCGCGTCCCATTCGCCATTCAGGCTGCGCAACTGTTGGGAAGGGCGAT
CGGTGCGGGCCTCTTCGCTATTACGCCAGCTGGCGAAAGGGGGATGTGCT
GCAAGGCGATTAAGTTGGGTAACGCCAGGGTTTTCCCAGTCACGACGTTG

TAAAACGACGGCCAGTGAGCGCGCGTAATACGACTCACTATAGGGCGAAT
TGGGTACCGGGCCCCCCCTCGACTACGCCGGCTCAAGATCTTCAACTAGTG
GTGGTTCAGGAGGTAGTGTTTCAAAAGGTGAAGAAGATAATATGGCAATT
ATTAAAGAATTTATGAGATTTAAAGTTCATATGGAAGGTTCAGTTAATGG
TCATGAATTTGAAATTGAAGGTGAAGGTGAAGGTAGACCATATGAAGGTA
CACAAACAGCAAAATTAAAAGTTACAAAAGGTGGTCCATTACCATTTGCA
TGGGATATTTTATCACCACAATTTATGTATGGTTCAAAAGCATATGTTAA
ACATCCAGCAGATATTCCAGATTATTTAAAATTATCATTTCCAGAAGGTT
TTAAATGGGAAAGAGTTATGAATTTTGAAGATGGTGGTGTTGTTACAGTT
ACACAAGATTCATCATTACAAGATGGTGAATTTATTTATAAAGTTAAATT
AAGAGGTACAAATTTTCCATCAGATGGTCCAGTTATGCAAAAGAAAACAA
TGGGTTGGGAAGCATCATCAGAAAGAATGTATCCAGAAGATGGTGCATTA
AAAGGTGAAATTAAACAAAGATTAAAATTAAAAGATGGTGGTCATTATGA
TGCAGAAGTTAAAACAACATATAAAGCAAAAAAACCAGTTCAATTACCAG
GTGCATATAATGTTAATATTAAATTAGATATTACATCACATAATGAAGAT
TATACAATTGTTGAACAATATGAAAGAGCAGAAGGTAGACATTCAACAGG
TGGTATGGATGAATTATATAAATAAGCTAGTTAAATAAATAAATTATTTA
ATAAATAATAAAAAAACAAATTGTTGTAATAATCTAATATTTTCTTTTTT
TTTTAATTTTTTTTTTTTAAATCTTAATAATTATTAAGTTATTTTAATTT
TTTTTTTTTTTTTTTTTTTTTTTTTTTTTTTTTTTCTATCAAAAAAATCA
AATATATTTAAAAAATTTATTATTTACAGATACATTTTGAATGGTGAAGA
TAAATATATGCATTAGATGTAAAACAGCCAAAGAGTATGAAAATCAAAAA
GATAAAGCTTGATATCTTAATTAATTAAATAACTTCGTATAGCATACATT
ATACGAAGTTATAGTAGTGGATCCCAAAATTTTGTATTTTGTGATGTTAG
TGTGTGTCATAATTGGAATATTGAAAAAAAAAAAATAAAAAATAAAAAAA
AAAAAAAAAAGAATTAATTAATGATTAATCATATGACTAACATTATATTT
TCAATTTTTTTTTTTTTTTTTTTCCAACCACCCAAAAGTGATCCATTTTT
TTTTTTTTTTTTAAATAAAATTTGGGTCACCCACAGTTGAATTATTTTAA
TTTTTTTTTTTTTTTTTTTTTTTTAATTTTTTTTTTTTTTTTTTTCAAAT
TTTTTTTTCTTCATATAAAAATAAATTTTCCAATTCATTTTTTTTTTTTT
TATTCAACTGTATATTATTATAACTCTTTATTTGTATTTTAAACTAATTT
CACAGCTAGCAAAAAATGGATGGTGAAGATGTTCAAGCTGGATCGTTTCG
CATGATTGAACAAGATGGATTGCACGCAGGTTCTCCGGCCGCTTGGGTGG
AGAGGCTATTCGGCTATGACTGGGCACAACAGACAATCGGCTGCTCTGAT
GCCGCCGTGTTCCGGCTGTCAGCGCAGGGGCGCCCGGTTCTTTTTGTCAA
GACCGACCTGTCCGGTGCCCTGAATGAACTGCAGGACGAGGCAGCGCGGC
TATCGTGGCTGGCCACGACGGGCGTTCCTTGCGCAGCTGTGCTCGACGTT
GTCACTGAAGCGGGAAGGGACTGGCTGCTATTGGGCGAAGTGCCGGGGCA
GGATCTCCTGTCATCTCACCTTGCTCCTGCCGAGAAAGTATCCATCATGG
CTGATGCAATGCGGCGGCTGCATACGCTTGATCCGGCTACCTGCCCATTC
GACCACCAAGCGAAACATCGCATCGAGCGAGCACGTACTCGGATGGAAGC
CGGTCTTGTCGATCAGGATGATCTGGACGAAGAGCATCAGGGGCTCGCGC
CAGCCGAACTGTTCGCCAGGCTCAAGGCGCGCATGCCCGACGGCGAGGAT
CTCGTCGTGACCCATGGCGATGCCTGCTTGCCGAATATCATGGTGGAAAA
TGGCCGCTTTTCTGGATTCATCGACTGTGGACGGCTGGGTGTGGCGGACC
GCTATCAGGACATAGCGTTGGCTACCCGTGATATTGCTGAAGAGCTTGGC
GGCGAATGGGCTGACCGCTTCCTCGTGCTTTACGGTATCGCCGCTCCCGA
TTCGCAGCGCATCGCCTTCTATCGCCTTCTTGACGAGTTCTTCTGAGCGG
CCGCATCAATTTGATTTCTTCTTAATTCAATGCTAAAAAAAAAAATATTA
TAAAAAAAAAAACACACACAACAAATAAATTAATAAATACCATTTATTAA

AAAAAAAAAAAAAAAAAAAAAACCTATTTTAACAATTATTTTATTATTTT
TATTTTTAGTTTTGTTAAAAAAAAAATTATTATCTTTATTTATAATTATA
AAAAATTTATCCAATGATCATTTTTTAACTTTTTTATTATTTAGTATATT
AAATAAAATCTCGAGAGTAGTATAACTTCGTATAGCATACATTATACGAA
GTTATGATATCTCATCTAGATCAGTCGACTCAGCCGGCTCAGAGCTCCAG
CTTTTGTTCCCTTTAGTGAGGGTTAATTGCGCGCTTGGCGTAATCATGGT
CATAGCTGTTTCCTGTGTGAAATTGTTATCCGCTCACAATTCCACACAAC
ATACGAGCCGGAAGCATAAAGTGTAAAGCCTGGGGTGCCTAATGAGTGAG
CTAACTCACATTAATTGCGTTGCGCTCACTGCCCGCTTTCCAGTCGGGAA
ACCTGTCGTGCCAGCTGCATTAATGAATCGGCCAACGCGCGGGGAGAGGC
GGTTTGCGTATTGGGCGCTCTTCCGCTTCCTCGCTCACTGACTCGCTGCG
CTCGGTCGTTCGGCTGCGGCGAGCGGTATCAGCTCACTCAAAGGCGGTAA
TACGGTTATCCACAGAATCAGGGGATAACGCAGGAAAGAACATGTGAGCA
AAAGGCCAGCAAAAGGCCAGGAACCGTAAAAAGGCCGCGTTGCTGGCGTT
TTTCCATAGGCTCCGCCCCCCTGACGAGCATCACAAAAATCGACGCTCAA
GTCAGAGGTGGCGAAACCCGACAGGACTATAAAGATACCAGGCGTTTCCC
CCTGGAAGCTCCCTCGTGCGCTCTCCTGTTCCGACCCTGCCGCTTACCGG
ATACCTGTCCGCCTTTCTCCCTTCGGGAAGCGTGGCGCTTTCTCATAGCT
CACGCTGTAGGTATCTCAGTTCGGTGTAGGTCGTTCGCTCCAAGCTGGGC
TGTGTGCACGAACCCCCCGTTCAGCCCGACCGCTGCGCCTTATCCGGTAA
CTATCGTCTTGAGTCCAACCCGGTAAGACACGACTTATCGCCACTGGCAG
CAGCCACTGGTAACAGGATTAGCAGAGCGAGGTATGTAGGCGGTGCTACA
GAGTTCTTGAAGTGGTGGCCTAACTACGGCTACACTAGAAGGACAGTATT
TGGTATCTGCGCTCTGCTGAAGCCAGTTACCTTCGGAAAAAGAGTTGGTA
GCTCTTGATCCGGCAAACAAACCACCGCTGGTAGCGGTGGTTTTTTTGTT
TGCAAGCAGCAGATTACGCGCAGAAAAAAAGGATCTCAAGAAGATCCTTT
GATCTTTTCTACGGGGTCTGACGCTCAGTGGAACGAAAACTCACGTTAAG
GGATTTTGGTCATGAGATTATCAAAAAGGATCTTCACCTAGATCCTTTTA
AATTAAAAATGAAGTTTTAAATCAATCTAAAGTATATATGAGTAAACTTG
GTCTGACAGTTACCAATGCTTAATCAGTGAGGCACCTATCTCAGCGATCT
GTCTATTTCGTTCATCCATAGTTGCCTGACTCCCCGTCGTGTAGATAACT
ACGATACGGGAGGGCTTACCATCTGGCCCCAGTGCTGCAATGATACCGCG
AGACCCACGCTCACCGGCTCCAGATTTATCAGCAATAAACCAGCCAGCCG
GAAGGGCCGAGCGCAGAAGTGGTCCTGCAACTTTATCCGCCTCCATCCAG
TCTATTAATTGTTGCCGGGAAGCTAGAGTAAGTAGTTCGCCAGTTAATAG
TTTGCGCAACGTTGTTGCCATTGCTACAGGCATCGTGGTGTCACGCTCGT
CGTTTGGTATGGCTTCATTCAGCTCCGGTTCCCAACGATCAAGGCGAGTT
ACATGATCCCCCATGTTGTGCAAAAAAGCGGTTAGCTCCTTCGGTCCTCC
GATCGTTGTCAGAAGTAAGTTGGCCGCAGTGTTATCACTCATGGTTATGG
CAGCACTGCATAATTCTCTTACTGTCATGCCATCCGTAAGATGCTTTTCT
GTGACTGGTGAGTACTCAACCAAGTCATTCTGAGAATAGTGTATGCGGCG
ACCGAGTTGCTCTTGCCCGGCGTCAATACGGGATAATACCGCGCCACATA
GCAGAACTTTAAAAGTGCTCATCATTGGAAAACGTTCTTCGGGGCGAAAA
CTCTCAAGGATCTTACCGCTGTTGAGATCCAGTTCGATGTAACCCACTCG
TGCACCCAACTGATCTTCAGCATCTTTTACTTTCACCAGCGTTTCTGGGT
GAGCAAAAACAGGAAGGCAAAATGCCGCAAAAAAGGGAATAAGGGCGACA
CGGAAATGTTGAATACTCATACTCTTCCTTTTTCAATATTATTGAAGCAT
TTATCAGGGTTATTGTCTCATGAGCGGATACATATTTGAATGTATTTAGA
AAAATAAACAAATAGGGGTTCCGCGCACATTTCCCCGAAAAGTGCCAC

knockout cassette vectors

pDM1079 – BS

GCCGGCCTTCAGTAGGCAGAGCTATCAAGCTTGATATCTTAATTAATTAA
ATAACTTCGTATAGCATACATTATACGAAGTTATAGTAGTGGATCCCAAA
ATTTTGTATTTTGTGATGTTAGTGTGTGTCATAATTGGAATATTGAAAAA
AAAAAAATAAAAAAATAAAAAAAAAAAAAAAAGAATTAATTAATGATTAA
TCATATGACTAACATTATATTTTCAATTTTTTTTTTTTTTTTTTTCCAAC
CACCCAAAAGTGATCCATTTTTTTTTTTTTTTTAAATAAAATTTGGGTCA
CCCACAGTTGAATTATTTTAATTTTTTTTTTTTTTTTTTTTTTTTTTTAA
TTTTTTTTTTTTTTTCAAAATTTTTTTTTTCTTCATATAAAAATAAATTT
TCCAATTCATTTTTTTTTTTTTTATTCAACTGTATATTATTATAACTCTT
TATTTGTATTTTAAACTAATTTCACAGCTAGCAAAAAATGGATCAATTTA
ACATTTCTCAACAGGATCTAGAATTAGTAGAAGTAGCGACAGAGAAGATT
ACAATGCTTTATGAGGATAATAAACATCATGTGGGAGCGGCAATTCGTAC
GAAAACAGGAGAAATCATTTCGGCAGTACATATTGAAGCGTATATAGGAC
GAGTAACTGTTTGTGCAGAAGCCATTGCGATTGGTAGTGCAGTTTCGAAT
GGACAAAAGGATTTTGACACGATTGTAGCTGTTAGACACCCTTATTCTGA
CGAAGTAGATAGAAGTATTCGAGTGGTAAGTCCTTGTGGTATGTGTAGGG
AGTTGATTTCAGACTATGCACCAGATTGTTTTGTGTTAATAGAAATGAAT
GGCAAGTTAGTCAAAACTACGATTGAAGAACTCATTCCACTCAAATATAC
CCGAAATTAAGCGGCCGCATCAATTTGATTTCTTCTTAATTCAATGCTAA
AAAAAAAAATATTATAAAAAAAAAAACACACACAACAAATAAATTAATAA
ATACCATTTATTAAAAAAAAAAAAAAAAAAAAAAAACCTATTTTAACAAT
TATTTTATTATTTTTATTTTTAGTTTTGTTAAAAAAAAAATTATTATCTT
TATTTATAATTATAAAAAATTTATCCAATGATCATTTTTTAACTTTTTTA
TTATTTAGTATATTAAATAAAATCTCGAGAGTAGTATAACTTCGTATAGC
ATACATTATACGAAGTTATGATATCAGATCTCTACTGGAGTATCCAAGCT
GACTAGTTGATCCCGCTACAGGGCGCGTCAGGTGGCACTTTTCGGGGAAA
TGTGCGCGGAACCCCTATTTGTTTATTTTTCTAAATACATTCAAATATGT
ATCCGCTCATGAGACAATAACCCTGATAAATGCTTCAATAATATTGAAAA
AGGAAGAGTATGAGTATTCAACATTTCCGTGTCGCCCTTATTCCCTTTTT
TGCGGCATTTTGCCTTCCTGTTTTTGCTCACCCAGAAACGCTGGTGAAAG
TAAAAGATGCTGAAGATCAGTTGGGTGCACGAGTGGGTTACATCGAACTG
GATCTCAACAGCGGTAAGATCCTTGAGAGTTTTCGCCCCGAAGAACGTTT
TCCAATGATGAGCACTTTTAAAGTTCTGCTATGTGGCGCGGTATTATCCC
GTATTGACGCCGGGCAAGAGCAACTCGGTCGCCGCATACACTATTCTCAG
AATGACTTGGTTGAGTACTCACCAGTCACAGAAAAGCATCTTACGGATGG
CATGACAGTAAGAGAATTATGCAGTGCTGCCATAACCATGAGTGATAACA
CTGCGGCCAACTTACTTCTGACAACGATCGGAGGACCGAAGGAGCTAACC
GCTTTTTTGCACAACATGGGGGATCATGTAACTCGCCTTGATCGTTGGGA
ACCGGAGCTGAATGAAGCCATACCAAACGACGAGCGTGACACCACGATGC
CTGTAGCAATGGCAACAACGTTGCGCAAACTATTAACTGGCGAACTACTT
ACTCTAGCTTCCCGGCAACAATTAATAGACTGGATGGAGGCGGATAAAGT
TGCAGGACCACTTCTGCGCTCGGCCCTTCCGGCTGGCTGGTTTATTGCTG
ATAAATCTGGAGCCGGTGAGCGTGGGTCTCGCGGTATCATTGCAGCACTG
GGGCCAGATGGTAAGCCCTCCCGTATCGTAGTTATCTACACGACGGGGAG
TCAGGCAACTATGGATGAACGAAATAGACAGATCGCTGAGATAGGTGCCT
CACTGATTAAGCATTGGTAACTGTCAGACCAAGTTTACTCATATATACTT

TAGATTGATTTAAAACTTCATTTTTAATTTAAAAGGATCTAGGTGAAGAT
CCTTTTTGATAATCTCATGACCAAAATCCCTTAACGTGAGTTTTCGTTCC
ACTGAGCGTCAGACCCCGTAGAAAAGATCAAAGGATCTTCTTGAGATCCT
TTTTTTCTGCGCGTAATCTGCTGCTTGCAAACAAAAAAACCACCGCTACC
AGCGGTGGTTTGTTTGCCGGATCAAGAGCTACCAACTCTTTTTCCGAAGG
TAACTGGCTTCAGCAGAGCGCAGATACCAAATACTGTCCTTCTAGTGTAG
CCGTAGTTAGGCCACCACTTCAAGAACTCTGTAGCACCGCCTACATACCT
CGCTCTGCTAATCCTGTTACCAGTGGCTGCTGCCAGTGGCGATAAGTCGT
GTCTTACCGGGTTGGACTCAAGACGATAGTTACCGGATAAGGCGCAGCGG
TCGGGCTGAACGGGGGGTTCGTGCACACAGCCCAGCTTGGAGCGAACGAC
CTACACCGAACTGAGATACCTACAGCGTGAGCTATGAGAAAGCGCCACGC
TTCCCGAAGGGAGAAAGGCGGACAGGTATCCGGTAAGCGGCAGGGTCGGA
ACAGGAGAGCGCACGAGGGAGCTTCCAGGGGGAAACGCCTGGTATCTTTA
TAGTCCTGTCGGGTTTCGCCACCTCTGACTTGAGCGTCGATTTTTGTGAT
GCTCGTCAGGGGGGCGGAGCCTATGGAAAAACGCCAGCAACGCGGCCTTT
TTACGGTTCCTGGCCTTTTGCTGGCCTTTTGCTCACATGTTCTTTCCTGC
GTTATCCCCTGATTCTGTGGATAACCGTATTACCGCCTTTGAGTGAGCTG
ATACCGCTCGCCGCAGCCGAACGACCGAGCGCAGCGAGTCAGTGAGCGAG
GAAGCGGAAGAGCGCCCAATACGCAAACCGCCTCT

pDM1080 – NT

GCCGGCCTTCAGTAGGCAGAGCTATCAAGCTTGATATCTTAATTAATTAA
ATAACTTCGTATAGCATACATTATACGAAGTTATAGTAGTGGATCCCAAA
ATTTTGTATTTTGTGATGTTAGTGTGTGTCATAATTGGAATATTGAAAAA
AAAAAAATAAAAAATAAAAAAAAAAAAAAAAAGAATTAATTAATGATTAA
TCATATGACTAACATTATATTTTCAATTTTTTTTTTTTTTTTTTTCCAAC
CACCCAAAAGTGATCCATTTTTTTTTTTTTTTTAAATAAAATTTGGGTCA
CCCACAGTTGAATTATTTTTAATTTTTTTTTTTTTTTTTTTTTTTTTTAA
TTTTTTTTTTTTTTTTCAAAATTTTTTTTTCTTCATATAAAAATAAATTT
TCCAATTCATTTTTTTTTTTTTTATTCAACTGTATATTATTATAACTCTT
TATTTGTATTTTAAACTAATTTCACAGCTAGCAAAAAATGAAAATTTCAG
TTATTCCAGAACAAGTTGCAGAAACATTAGATGCAGAAAATCATTTTATT
GTTAGAGAAGTTTTTGATGTTCATTTATCAGATCAAGGTTTTGAATTATC
AACAAGATCAGTTTCACCATATAGAAAAGATTATATTTCAGATGATGATT
CAGATGAAGATTCAGCATGTTATGGTGCATTTATTGATCAAGAATTAGTT
GGTAAAATTGAATTAAATTCAACATGGAATGATTTAGCATCAATTGAACA
TATTGTTGTTTCACATACACATAGAGGTAAAGGTGTTGCACATTCATTAA
TTGAATTTGCAAAAAAAATGGGCATTATCAAGACAATTATTAGGTATTAGA
TTAGAAACACAAACAAATAATGTTCCAGCATGTAATTTATATGCAAAATG
TGGTTTTACATTAGGTGGTATTGATTTATTTACATATAAAACAAGACCAC
AAGTTTCAAATGAAACAGCAATGTATTGGTATTGGTTTTCAGGTGCACAA
GATGATGCATAAGCGGCCGCATCAATTTGATTTCTTCTTAATTCAATGCT
AAAAAAAAAAATATTATAAAAAAAAAAACACACACAACAAATAAATTAAT
AAATACCATTTATTAAAAAAAAAAAAAAAAAAAAAAAACCTATTTTAACA
ATTATTTTATTATTTTTATTTTTAGTTTTGTTAAAAAAAAAATTATTATC
TTTATTTATAATTATAAAAAATTTATCCAATGATCATTTTTTAACTTTTT
TATTATTTAGTATATTAAATAAAATCTCGAGAGTAGTATAACTTCGTATA
GCATACATTATACGAAGTTATGATATCAGATCTCTACTGGAGTATCCAAG
CTGACTAGTTGATCCCGCTACAGGGCGCGTCAGGTGGCACTTTTCGGGGA

AATGTGCGCGGAACCCCTATTTGTTTATTTTTCTAAATACATTCAAATAT
GTATCCGCTCATGAGACAATAACCCTGATAAATGCTTCAATAATATTGAA
AAAGGAAGAGTATGAGTATTCAACATTTCCGTGTCGCCCTTATTCCCTTT
TTTGCGGCATTTTGCCTTCCTGTTTTTGCTCACCCAGAAACGCTGGTGAA
AGTAAAAGATGCTGAAGATCAGTTGGGTGCACGAGTGGGTTACATCGAAC
TGGATCTCAACAGCGGTAAGATCCTTGAGAGTTTTCGCCCCGAAGAACGT
TTTCCAATGATGAGCACTTTTAAAGTTCTGCTATGTGGCGCGGTATTATC
CCGTATTGACGCCGGGCAAGAGCAACTCGGTCGCCGCATACACTATTCTC
AGAATGACTTGGTTGAGTACTCACCAGTCACAGAAAAGCATCTTACGGAT
GGCATGACAGTAAGAGAATTATGCAGTGCTGCCATAACCATGAGTGATAA
CACTGCGGCCAACTTACTTCTGACAACGATCGGAGGACCGAAGGAGCTAA
CCGCTTTTTTGCACAACATGGGGGATCATGTAACTCGCCTTGATCGTTGG
GAACCGGAGCTGAATGAAGCCATACCAAACGACGAGCGTGACACCACGAT
GCCTGTAGCAATGGCAACAACGTTGCGCAAACTATTAACTGGCGAACTAC
TTACTCTAGCTTCCCGGCAACAATTAATAGACTGGATGGAGGCGGATAAA
GTTGCAGGACCACTTCTGCGCTCGGCCCTTCCGGCTGGCTGGTTTATTGC
TGATAAATCTGGAGCCGGTGAGCGTGGGTCTCGCGGTATCATTGCAGCAC
TGGGGCCAGATGGTAAGCCCTCCCGTATCGTAGTTATCTACACGACGGGG
AGTCAGGCAACTATGGATGAACGAAATAGACAGATCGCTGAGATAGGTGC
CTCACTGATTAAGCATTGGTAACTGTCAGACCAAGTTTACTCATATATAC
TTTAGATTGATTTAAAACTTCATTTTTAATTTAAAAGGATCTAGGTGAAG
ATCCTTTTTGATAATCTCATGACCAAAATCCCTTAACGTGAGTTTTCGTT
CCACTGAGCGTCAGACCCCGTAGAAAAGATCAAAGGATCTTCTTGAGATC
CTTTTTTTCTGCGCGTAATCTGCTGCTTGCAAACAAAAAAACCACCGCTA
CCAGCGGTGGTTTGTTTGCCGGATCAAGAGCTACCAACTCTTTTTCCGAA
GGTAACTGGCTTCAGCAGAGCGCAGATACCAAATACTGTCCTTCTAGTGT
AGCCGTAGTTAGGCCACCACTTCAAGAACTCTGTAGCACCGCCTACATAC
CTCGCTCTGCTAATCCTGTTACCAGTGGCTGCTGCCAGTGGCGATAAGTC
GTGTCTTACCGGGTTGGACTCAAGACGATAGTTACCGGATAAGGCGCAGC
GGTCGGGCTGAACGGGGGGTTCGTGCACACAGCCCAGCTTGGAGCGAACG
ACCTACACCGAACTGAGATACCTACAGCGTGAGCTATGAGAAAGCGCCAC
GCTTCCCGAAGGGAGAAAGGCGGACAGGTATCCGGTAAGCGGCAGGGTCG
GAACAGGAGAGCGCACGAGGGAGCTTCCAGGGGGAAACGCCTGGTATCTT
TATAGTCCTGTCGGGTTTCGCCACCTCTGACTTGAGCGTCGATTTTTGTG
ATGCTCGTCAGGGGGGCGGAGCCTATGGAAAAACGCCAGCAACGCGGCCT
TTTTACGGTTCCTGGCCTTTTGCTGGCCTTTTGCTCACATGTTCTTTCCT
GCGTTATCCCCTGATTCTGTGGATAACCGTATTACCGCCTTTGAGTGAGC
TGATACCGCTCGCCGCAGCCGAACGACCGAGCGCAGCGAGTCAGTGAGCG
AGGAAGCGGAAGAGCGCCCAATACGCAAACCGCCTCT

pDM1081 – Hyg

GCCGGCCTTCAGTAGGCAGAGCTATCAAGCTTGATATCTTAATTAATTAA
ATAACTTCGTATAGCATACATTATACGAAGTTATAGTAGTGGATCCCAAA
ATTTTGTATTTTGTGATGTTAGTGTGTGTCATAATTGGAATATTGAAAAA
AAAAAAATAAAAAAATAAAAAAAAAAAAAAAAGAATTAATTAATGATTAA
TCATATGACTAACATTATATTTCAATTTTTTTTTTTTTTTTTTTTCCAAC
CACCCAAAAGTGATCCATTTTTTTTTTTTTTTTAAATAAAATTTGGGTCA
CCCACAGTTGAATTATTTTAATTTTTTTTTTTTTTTTTTTTTTTTTTTAA
TTTTTTTTTTTTTTTTCAAAATTTTTTTTTCTTCATATAAAAATAAATTT

TCCAATTCATTTTTTTTTTTTTTATTCAACTGTATATTATTATAACTCTT
TATTTGTATTTTAAACTAATTTCACAGCTAGCAAAAAATGGATCAATTTA
ACATTTCTCAACAAGACCTAATGAAAAAGCCTGAACTCACCGCGACGTCT
GTCGAGAAGTTTCTGATCGAAAAGTTCGACAGCGTCTCCGACCTGATGCA
GCTCTCGGAGGGCGAAGAATCTCGTGCTTTCAGCTTCGATGTAGGAGGGC
GTGGATATGTCCTGCGGGTAAATAGCTGCGCCGATGGTTTCTACAAAGAT
CGTTATGTTTATCGGCACTTTGCATCGGCCGCGCTCCCGATTCCGGAAGT
GCTTGACATTGGGGAGTTCAGCGAGAGCCTGACCTATTGCATCTCCCGCC
GTGCACAGGGTGTCACGTTGCAAGACCTGCCTGAAACCGAACTGCCCGCT
GTTCTGCAGCCGGTCGCGGAGGCCATGGATGCGATCGCTGCGGCCGATCT
TAGCCAGACGAGCGGGTTCGGCCCATTCGGACCGCAAGGAATCGGTCAAT
ACACTACATGGCGTGATTTCATATGCGCGATTGCTGATCCCCATGTGTAT
CACTGGCAAACTGTGATGGACGACACCGTCAGTGCGTCCGTCGCGCAGGC
TCTCGATGAGCTGATGCTTTGGGCCGAGGACTGCCCCGAAGTCCGGCACC
TCGTGCACGCGGATTTCGGCTCCAACAATGTCCTGACGGACAATGGCCGC
ATAACAGCGGTCATTGACTGGAGCGAGGCGATGTTCGGGGATTCCCAATA
CGAGGTCGCCAACATCTTCTTCTGGAGGCCGTGGTTGGCTTGTATGGAGC
AGCAGACGCGCTACTTCGAGCGGAGGCATCCGGAGCTTGCAGGATCGCCG
CGGCTCCGGGCGTATATGCTCCGCATTGGTCTTGACCAACTCTATCAGAG
CTTGGTTGACGGCAATTTCGATGATGCAGCTTGGGCGCAGGGTCGATGCG
ACGCAATCGTCCGATCCGGAGCCGGGACTGTCGGGCGTACACAAATCGCC
CGCAGAAGCGCGGCCGTCTGGACCGATGGCTGTGTAGAAGTACTCGCCGA
TAGTGGAAACCGACGCCCCAGCACTCGTCCGGATCGGGAGATGGGGGAGG
CTAACTAAGCGGCCGCATCAATTTGATTTCTTCTTAATTCAATGCTAAAA
AAAAAAATATTATAAAAAAAAAAACACACACAACAAATAAATTAATAAAT
ACCATTTATTAAAAAAAAAAAAAAAAAAAAAAAACCTATTTTAACAATTA
TTTTATTATTTTTATTTTTAGTTTTGTTAAAAAAAAAATTATTATCTTTA
TTTATAATTATAAAAAATTTATCCAATGATCATTTTTTAACTTTTTTATT
ATTTAGTATATTAAATAAAATCTCGAGAGTAGTATAACTTCGTATAGCAT
ACATTATACGAAGTTATGATATCAGATCTCTACTGGAGTATCCAAGCTGA
CTAGTTGATCCCGCTACAGGGCGCGTCAGGTGGCACTTTTCGGGGAAATG
TGCGCGGAACCCCTATTTGTTTATTTTTCTAAATACATTCAAATATGTAT
CCGCTCATGAGACAATAACCCTGATAAATGCTTCAATAATATTGAAAAAG
GAAGAGTATGAGTATTCAACATTTCCGTGTCGCCCTTATTCCCTTTTTTG
CGGCATTTTGCCTTCCTGTTTTTGCTCACCCAGAAACGCTGGTGAAAGTA
AAAGATGCTGAAGATCAGTTGGGTGCACGAGTGGGTTACATCGAACTGGA
TCTCAACAGCGGTAAGATCCTTGAGAGTTTTCGCCCCGAAGAACGTTTTC
CAATGATGAGCACTTTTAAAGTTCTGCTATGTGGCGCGGTATTATCCCGT
ATTGACGCCGGGCAAGAGCAACTCGGTCGCCGCATACACTATTCTCAGAA
TGACTTGGTTGAGTACTCACCAGTCACAGAAAAGCATCTTACGGATGGCA
TGACAGTAAGAGAATTATGCAGTGCTGCCATAACCATGAGTGATAACACT
GCGGCCAACTTACTTCTGACAACGATCGGAGGACCGAAGGAGCTAACCGC
TTTTTTGCACAACATGGGGGATCATGTAACTCGCCTTGATCGTTGGGAAC
CGGAGCTGAATGAAGCCATACCAAACGACGAGCGTGACACCACGATGCCT
GTAGCAATGGCAACAACGTTGCGCAAACTATTAACTGGCGAACTACTTAC
TCTAGCTTCCCGGCAACAATTAATAGACTGGATGGAGGCGGATAAAGTTG
CAGGACCACTTCTGCGCTCGGCCCTTCCGGCTGGCTGGTTTATTGCTGAT
AAATCTGGAGCCGGTGAGCGTGGGTCTCGCGGTATCATTGCAGCACTGGG
GCCAGATGGTAAGCCCTCCCGTATCGTAGTTATCTACACGACGGGGAGTC
AGGCAACTATGGATGAACGAAATAGACAGATCGCTGAGATAGGTGCCTCA

CTGATTAAGCATTGGTAACTGTCAGACCAAGTTTACTCATATATACTTTA
GATTGATTTAAAACTTCATTTTTAATTTAAAAGGATCTAGGTGAAGATCC
TTTTTGATAATCTCATGACCAAAATCCCTTAACGTGAGTTTTCGTTCCAC
TGAGCGTCAGACCCCGTAGAAAAGATCAAAGGATCTTCTTGAGATCCTTT
TTTTCTGCGCGTAATCTGCTGCTTGCAAACAAAAAAACCACCGCTACCAG
CGGTGGTTTGTTTGCCGGATCAAGAGCTACCAACTCTTTTTCCGAAGGTA
ACTGGCTTCAGCAGAGCGCAGATACCAAATACTGTCCTTCTAGTGTAGCC
GTAGTTAGGCCACCACTTCAAGAACTCTGTAGCACCGCCTACATACCTCG
CTCTGCTAATCCTGTTACCAGTGGCTGCTGCCAGTGGCGATAAGTCGTGT
CTTACCGGGTTGGACTCAAGACGATAGTTACCGGATAAGGCGCAGCGGTC
GGGCTGAACGGGGGGTTCGTGCACACAGCCCAGCTTGGAGCGAACGACCT
ACACCGAACTGAGATACCTACAGCGTGAGCTATGAGAAAGCGCCACGCTT
CCCGAAGGGAGAAAGGCGGACAGGTATCCGGTAAGCGGCAGGGTCGGAAC
AGGAGAGCGCACGAGGGAGCTTCCAGGGGGAAACGCCTGGTATCTTTATA
GTCCTGTCGGGTTTCGCCACCTCTGACTTGAGCGTCGATTTTTGTGATGC
TCGTCAGGGGGGCGGAGCCTATGGAAAAACGCCAGCAACGCGGCCTTTTT
ACGGTTCCTGGCCTTTTGCTGGCCTTTTGCTCACATGTTCTTTCCTGCGT
TATCCCCTGATTCTGTGGATAACCGTATTACCGCCTTTGAGTGAGCTGAT
ACCGCTCGCCGCAGCCGAACGACCGAGCGCAGCGAGTCAGTGAGCGAGGA
AGCGGAAGAGCGCCCAATACGCAAACCGCCTCT

pDM1082 – G418

GCCGGCCTTCAGTAGGCAGAGCTATCAAGCTTGATATCTTAATTAATTAA
ATAACTTCGTATAGCATACATTATACGAAGTTATAGTAGTGGATCCCAAA
ATTTTGTATTTTGTGATGTTAGTGTGTGTCATAATTGGAATATTGAAAAA
AAAAAAATAAAAAATAAAAAAAAAAAAAAAAAGAATTAATTAATGATTAA
TCATATGACTAACATTATATTTTCAATTTTTTTTTTTTTTTTTTTCCAAC
CACCCAAAAGTGATCCATTTTTTTTTTTTTTTTAAATAAAATTTGGGTCA
CCCACAGTTGAATTATTTTAATTTTTTTTTTTTTTTTTTTTTTTTTTTAA
TTTTTTTTTTTTTTTTCAAAATTTTTTTTTCTTCATATAAAAATAAATTT
TCCAATTCATTTTTTTTTTTTTTATTCAACTGTATATTATTATAACTCTT
TATTTGTATTTTAAACTAATTTCACAGCTAGCAAAAAATGGATGGTGAAG
ATGTTCAAGCTGGATCGTTTCGCATGATTGAACAAGATGGATTGCACGCA
GGTTCTCCGGCCGCTTGGGTGGAGAGGCTATTCGGCTATGACTGGGCACA
ACAGACAATCGGCTGCTCTGATGCCGCCGTGTTCCGGCTGTCAGCGCAGG
GGCGCCCGGTTCTTTTTGTCAAGACCGACCTGTCCGGTGCCCTGAATGAA
CTGCAGGACGAGGCAGCGCGGCTATCGTGGCTGGCCACGACGGGCGTTCC
TTGCGCAGCTGTGCTCGACGTTGTCACTGAAGCGGGAAGGGACTGGCTGC
TATTGGGCGAAGTGCCGGGGCAGGATCTCCTGTCATCTCACCTTGCTCCT
GCCGAGAAAGTATCCATCATGGCTGATGCAATGCGGCGGCTGCATACGCT
TGATCCGGCTACCTGCCCATTCGACCACCAAGCGAAACATCGCATCGAGC
GAGCACGTACTCGGATGGAAGCCGGTCTTGTCGATCAGGATGATCTGGAC
GAAGAGCATCAGGGGCTCGCGCCAGCCGAACTGTTCGCCAGGCTCAAGGC
GCGCATGCCCGACGGCGAGGATCTCGTCGTGACCCATGGCGATGCCTGCT
TGCCGAATATCATGGTGGAAAATGGCCGCTTTTCTGGATTCATCGACTGT
GGACGGCTGGGTGTGGCGGACCGCTATCAGGACATAGCGTTGGCTACCCG
TGATATTGCTGAAGAGCTTGGCGGCGAATGGGCTGACCGCTTCCTCGTGC
TTTACGGTATCGCCGCTCCCGATTCGCAGCGCATCGCCTTCTATCGCCTT
CTTGACGAGTTCTTCTGAGCGGCCGCATCAATTTGATTTCTTCTTAATTC

AATGCTAAAAAAAAAAATATTATAAAAAAAAAAACACACACAACAAATAA
ATTAATAAATACCATTTATTAAAAAAAAAAAAAAAAAAAAAAAAACCTATT
TTAACAATTATTTTATTATTTTTATTTTTAGTTTTGTTAAAAAAAAAATT
ATTATCTTTATTTATAATTATAAAAAATTTATCCAATGATCATTTTTTAA
CTTTTTTATTATTTAGTATATTAAATAAAATCTCGAGAGTAGTATAACTT
CGTATAGCATACATTATACGAAGTTATGATATCAGATCTCTACTGGAGTA
TCCAAGCTGACTAGTTGATCCCGCTACAGGGCGCGTCAGGTGGCACTTTT
CGGGGAAATGTGCGCGGAACCCCTATTTGTTTATTTTTCTAAATACATTC
AAATATGTATCCGCTCATGAGACAATAACCCTGATAAATGCTTCAATAAT
ATTGAAAAAGGAAGAGTATGAGTATTCAACATTTCCGTGTCGCCCTTATT
CCCTTTTTTGCGGCATTTTGCCTTCCTGTTTTTGCTCACCCAGAAACGCT
GGTGAAAGTAAAAGATGCTGAAGATCAGTTGGGTGCACGAGTGGGTTACA
TCGAACTGGATCTCAACAGCGGTAAGATCCTTGAGAGTTTTCGCCCCGAA
GAACGTTTTCCAATGATGAGCACTTTTAAAGTTCTGCTATGTGGCGCGGT
ATTATCCCGTATTGACGCCGGGCAAGAGCAACTCGGTCGCCGCATACACT
ATTCTCAGAATGACTTGGTTGAGTACTCACCAGTCACAGAAAAGCATCTT
ACGGATGGCATGACAGTAAGAGAATTATGCAGTGCTGCCATAACCATGAG
TGATAACACTGCGGCCAACTTACTTCTGACAACGATCGGAGGACCGAAGG
AGCTAACCGCTTTTTTGCACAACATGGGGGATCATGTAACTCGCCTTGAT
CGTTGGGAACCGGAGCTGAATGAAGCCATACCAAACGACGAGCGTGACAC
CACGATGCCTGTAGCAATGGCAACAACGTTGCGCAAACTATTAACTGGCG
AACTACTTACTCTAGCTTCCCGGCAACAATTAATAGACTGGATGGAGGCG
GATAAAGTTGCAGGACCACTTCTGCGCTCGGCCCTTCCGGCTGGCTGGTT
TATTGCTGATAAATCTGGAGCCGGTGAGCGTGGGTCTCGCGGTATCATTG
CAGCACTGGGGCCAGATGGTAAGCCCTCCCGTATCGTAGTTATCTACACG
ACGGGGAGTCAGGCAACTATGGATGAACGAAATAGACAGATCGCTGAGAT
AGGTGCCTCACTGATTAAGCATTGGTAACTGTCAGACCAAGTTTACTCAT
ATATACTTTAGATTGATTTAAAACTTCATTTTTAATTTAAAAGGATCTAG
GTGAAGATCCTTTTTGATAATCTCATGACCAAAATCCCTTAACGTGAGTT
TTCGTTCCACTGAGCGTCAGACCCCGTAGAAAAGATCAAAGGATCTTCTT
GAGATCCTTTTTTTCTGCGCGTAATCTGCTGCTTGCAAACAAAAAAACCA
CCGCTACCAGCGGTGGTTTGTTTGCCGGATCAAGAGCTACCAACTCTTTT
TCCGAAGGTAACTGGCTTCAGCAGAGCGCAGATACCAAATACTGTCCTTC
TAGTGTAGCCGTAGTTAGGCCACCACTTCAAGAACTCTGTAGCACCGCCT
ACATACCTCGCTCTGCTAATCCTGTTACCAGTGGCTGCTGCCAGTGGCGA
TAAGTCGTGTCTTACCGGGTTGGACTCAAGACGATAGTTACCGGATAAGG
CGCAGCGGTCGGGCTGAACGGGGGGTTCGTGCACACAGCCCAGCTTGGAG
CGAACGACCTACACCGAACTGAGATACCTACAGCGTGAGCTATGAGAAAG
CGCCACGCTTCCCGAAGGGAGAAAGGCGGACAGGTATCCGGTAAGCGGCA
GGGTCGGAACAGGAGAGCGCACGAGGGAGCTTCCAGGGGGAAACGCCTGG
TATCTTTATAGTCCTGTCGGGTTTCGCCACCTCTGACTTGAGCGTCGATT
TTTGTGATGCTCGTCAGGGGGGCGGAGCCTATGGAAAAACGCCAGCAACG
CGGCCTTTTTACGGTTCCTGGCCTTTTGCTGGCCTTTTGCTCACATGTTC
TTTCCTGCGTTATCCCCTGATTCTGTGGATAACCGTATTACCGCCTTTGA
GTGAGCTGATACCGCTCGCCGCAGCCGAACGACCGAGCGCAGCGAGTCAG
TGAGCGAGGAAGCGGAAGAGCGCCCAATACGCAAACCGCCTCT

Cre expression vectors

pDM1483 – NT

CTCGAGACTAGAGCTAGATAAAAAAAATTTTTATTTATTTTTATTTATTT
TGAATTAAATAGATTACAAATTAATTAATCCCATCAAATCTTTAAAAAAA
AATGGTTTAAAAAAACTTGGGTTGGTTAATTATTATTTGAAAATTTTAAA
ACCCAAATTAAAAAAAAAAAAAATGGGATTCAAAAATTTTTTTTTTTTTT
TTTTTTTTTTTTTTTTTTTTTTTCAGATTGCATAAAAAGATTTTTTTTT
TTTTTTTTCTTATTTCTTAAAACAAATAAATTAAATTAAATAAAAAATAA
AAATCAGATCCAAAATGCATCATCATCATCACCATCATCATGATATAGGT
ACAATGCCAAAGAAGAAACGTAAGGTTAGTAATTTATTAACAGTGCATCA
GAATTTGCCAGCATTACCAGTAGATGCTACATCAGATGAAGTGCGTAAAA
ATTTGATGGATATGTTCAGAGATAGACAGGCTTTTAGTGAACACACATGG
AAGATGTTATTAAGCGTATGCCGTTCATGGGCCGCTTGGTGTAAACTCAA
CAATAGAAAATGGTTTCCAGCTGAACCAGAGGATGTAAGAGATTATTTAC
TCTACTTACAAGCAAGAGGTTTAGCTGTAAAAACAATTCAACAGCATTTA
GGTCAATTAAATATGTTACATCGTAGATCGGGTTTGCCAAGACCATCAGA
TTCAAATGCTGTTTCATTAGTCATGCGTCGTATACGTAAAGAGAATGTTG
ATGCAGGAGAAAGAGCTAAGCAAGCACTAGCTTTTGAACGTACAGATTTT
GATCAAGTTCGTAGTTTAATGGAAAATTCAGACAGATGTCAAGATATTAG
AAATTTAGCTTTCTTAGGGATTGCATACAATACATTGCTTCGTATTGCAG
AAATTGCCAGAATTAGAGTTAAAGATATCAGTCGTACAGACGGTGGTAGA
ATGTTAATACATATCGGTAGGACTAAAACTTTAGTTAGTACAGCCGGTGT
CGAAAAAGCATTATCATTGGGTGTAACTAAATTGGTAGAGCGTTGGATTA
GTGTAAGTGGTGTTGCCGATGATCCAAATAATTATCTTTTCTGTAGAGTT
AGAAAGAATGGAGTTGCTGCACCTAGCGCTACATCTCAACTCTCAACAAG
AGCTTTGGAAGGTATCTTTGAAGCTACTCATAGATTAATTTACGGTGCTA
AAGATGACTCAGGTCAACGTTATTTAGCATGGTCTGGTCACTCAGCAAGA
GTCGGTGCAGCACGTGATATGGCTCGTGCTGGTGTATCAATACCAGAAAT
TATGCAAGCTGGTGGATGGACTAATGTGAATATAGTGATGAATTATATTA
GGAATTTAGATTCTGAAACAGGTGCAATGGTTAGATTACTACAAGATGGT
GATTAAGCTAGTTAAATAAATAAATTATTTAATAAATAATAAAAAAACAA
ATTGTTGTAATAATCTAATATTTTCTTTTTTTTTTAATTTTTTTTTTTTA
AATCTTAATAATTATTAAGTTATTTTAATTTTTTTTTTTTTTTTTTTTTT
TTTTTTTTTTTTTTTTCTATCAAAAAAATCAAATATATTTAAAAAATTTA
TTATTTACAGATACATTTTGAATGGTGAAGATAAATATATGCATTAGATG
TAAAACAGCCAAAGAGTATGAAAATCAAAAAGATAAAGCTTATCGATTTC
GAAAAAGTAAATAGCAATTATTACAAAATTCAATCCGAATCTACCCAAAT
AAATTCCAATGAAATTGCCGATTTAAAAAAGTTTATTAAAGAAGAAGTCA
ATAAAACTTCTTCCAAAATTGATTTCTTTTTAGTTTCTTCAACAGATGCC
CTTTCAAATCCAGAAAATTATTCTCTCTTAGAAGTAAAGTGTATTAATTG
TCATTCTTTGTGTCAAGGAAAAAATTTATATATTTCATGTACAAGAGATG
GATGTCAAAACAATATTTGCTATAATTGTTTAGGAATAAACATAAACATA
TATAATGTTGTTATTAATTCTAAACTTTGCCCTCCATGTTTCAATGATTC
GGTAATCAACAAGAAGTGTGCCATGTGTAGTAAGAACGGAACTAAATGTA
ATTTGAACCAAGAATGTAAACTTCATCTTTGTGCACAGTGTTCTAAAAAG
TGTCTATACATTCTGAGAGTCAAAACTAATTAAATAAAATATAAACTTAA
TTTCTAAATAAACTCATTTAAAAATATTTAAATAATATGAATTTATAACT
GTAATTATTGTATTAAAAAATTATATAATTATTTAATGTTAAAAATGTAT
TAAAATAATTATAAAAAAATATAACAAAAATTTTCGTAAAAATAATTTGT
AAAAAAGCTATTAAAAATATTATGAAAAAAAAATTAAAAAAATTATTAAA
TTGTTTTTGTAATTAAGCTATTAAAATAATTATAAAAAAAAAATTTTTAA

AATTTTAAAAATATTTTTTGTAAAAAAGTATTAAAATAATTATGAAAAAA
AAATTTTCTAAAAAATTAAAAAAAAAATTAAAATATATTTTATGTTAAAA
ACGTATTAAAATAACTATTAAAAAAATTATATTTAAAAAAGTATTAACTT
TTTTTTAGGTGTGGTTGTGGGGTGGGGTTTAATATATTATAATAAAAAAT
TATTTTTTGTTCATTTATTATTTTCATTGTATATAATGTACTCAACAACG
TTATTATTTTTTCTTTTTTTTTTTATTGTATCAAAATCTTCTGTTCTTCA
AAATGATCAGATTGAAGTAAAATATTTTCAACTTCTTATTGTTATGTATC
AAAAAGAAAACTGTGTTGAAAAGTCAATGACAGGCGCCGTAATTTATGAT
GAATGTAATATTCATGGAAGAGTTGAAACAAATAGTACTCATGCGCTTTT
TTATGATGACATTGAAACAAATAATTCAAGATGTAACAATTTTCGTAATT
TAACAAACTTAATTAAACTTAATGAATGTATTAATGACGAGTTTGGAGAG
TCTATTCTTTATAAAGAATATAATGAAACTGATGATGGTTATTTGTTTAG
AGTGGAAGACAGCTTTGTTGAAATTACTTCTCTTTCAATGGATTGTACAA
AAAATAGTAAAACAATTATTGAAAAATTCAACATTTGTTCAAAATTTGAA
AATGTATATCATATTACAAACATTACACAAGAGAAATCCAATAGATTTAC
ATGTACAGATCCATTGTGCCACTATTGTAAGAATGAAAACATTCAAAACA
ATCTTGATTTTAAAACAACAAAGTGTACTCCAAAGTATGGTGCATCTGAT
TCTGAATTTTTATCAACAATTTACAATCCAAAGCTCGATGGCTCAAATAA
CGGTATGGAAAAGTCAGTAACTCAAGAAAAAAACATTTCAAATAATTTAA
AAATTAATATATATTTAATTTTCTTTTTAATTATTTTTTTAATTAAATAA
AGTTTTATTATTTTTTAAGAGTAATTATTGCTCTTTTTTCATTTGAAACA
CCAGAAGCTAAACGTAATTGTTGTTGACTGAAATTTTTTATTTTTTTTGG
GGTAATAGGATTTCCTTTTTTATGAAGATTAATATCTTTGACTCGTGAAA
CATTCTTTTTAACTTTTGTTTTTTCTGTTGGTTTATCATTTGTTTTTTCA
CTAATTTCAATACCATCTTGACGTTCATTCATAACTTCATCTTTTTTTTT
TCCTGTTTCTGTATCTTCTTCTATTTTTTTTTCTTTATCTTTTTCTTTAT
CTTCTTCTTGTTCTTCCTCTTCTTTTTCTTCTTCTGATACTGCAGGTGTT
TCTTCTTCTTCTTCTTCCGATATTGTCGGTTTTTCTACTTCTTCTTCTTG
TTCTTCTTCTTCTTCTTCTTCTTCTTCTTCTTCTTCTTCTTCTTCTTCTT
CTTCCGGTAATTTATTAATTATATTTCTTTTTTTTATATGAATTACGTTTG
GTTTGTGCAGTAATTTCCTTACATAGAGTGCAGCTTTCAAGAAAAATTTC
AATTTCTTCGTTTGTTGCATAATAACCACTGTCTTTGATATGATTAAACA
TTTTTGATTTTCTTAAATGCTTTCCTTCTTTAATATGAAAATTATCGAAT
TCTAATTCATTAAGAACAATAAGCTCCCCTAATTTAAAAAATTAGTTAAA
ATAAATTAAAATGAACATGTATAAAGATGGATTTTACCATTTTTTGAAAT
TCTAAATAACTTTTCTTCATCTCCAATCTTTTTGACTGAAAAACGATTTT
TAATTGAAGTTATTGTTCTGTGAGTGTTTTGAATCGCCCATTTCTCTAAA
TCAGTTTGAGATAGTGTTTTATAATCTGAATTGTTATACACAACTTTTGC
TCTATTAACCAAATATTTAAAGATTTCATCATCAACTGAATATTTTGACT
TTACGATTCTTGTCCAAAAAAACAATTTCTACTACTATCATTTTTTATTTA
TAAAATAATTTAAATACAAAAATGAATTTTTTTTTTTTTAAAAAAAAAAA
AATTTGAAAAAAAAAAAAAAAAAAATTTTAAAAAAAAAAAAAAAAAAAAAA
AAAAAAAAAAAATCAAATAAAAAGTAAAAAATAAAAACCGAAAAACATTC
ATTGTAATTTCAAATGTCGAGGCCGGCAGAGGCGGTTTGCGTATTGGGCG
CTCTTCCGCTTCCTCGCTCACTGACTCGCTGCGCTCGGTCGTTCGGCTGC
GGCGAGCGGTATCAGCTCACTCAAAGGCGGTAATACGGTTATCCACAGAA
TCAGGGGATAACGCAGGAAAGAACATGTGAGCAAAAGGCCAGCAAAAGGC
CAGGAACCGTAAAAAGGCCGCGTTGCTGGCGTTTTTCCATAGGCTCCGCC
CCCCTGACGAGCATCACAAAAATCGACGCTCAAGTCAGAGGTGGCGAAAC
CCGACAGGACTATAAAGATACCAGGCGTTTCCCCCTGGAAGCTCCCTCGT

GCGCTCTCCTGTTCCGACCCTGCCGCTTACCGGATACCTGTCCGCCTTTC
TCCCTTCGGGAAGCGTGGCGCTTTCTCATAGCTCACGCTGTAGGTATCTC
AGTTCGGTGTAGGTCGTTCGCTCCAAGCTGGGCTGTGTGCACGAACCCCC
CGTTCAGCCCGACCGCTGCGCCTTATCCGGTAACTATCGTCTTGAGTCCA
ACCCGGTAAGACACGACTTATCGCCACTGGCAGCAGCCACTGGTAACAGG
ATTAGCAGAGCGAGGTATGTAGGCGGTGCTACAGAGTTCTTGAAGTGGTG
GCCTAACTACGGCTACACTAGAAGGACAGTATTTGGTATCTGCGCTCTGC
TGAAGCCAGTTACCTTCGGAAAAAGAGTTGGTAGCTCTTGATCCGGCAAA
CAAACCACCGCTGGTAGCGGTGGTTTTTTTGTTTGCAAGCAGCAGATTAC
GCGCAGAAAAAAAGGATCTCAAGAAGATCCTTTGATCTTTTCTACGGGGT
CTGACGCTCAGTGGAACGAAAACTCACGTTAAGGGATTTTGGTCATGAGA
TTATCAAAAAGGATCTTCACCTAGATCCTTTTAAATTAAAAATGAAGTTT
TAAATCAATCTAAAGTATATATGAGTAAACTTGGTCTGACAGTTACCAAT
GCTTAATCAGTGAGGCACCTATCTCAGCGATCTGTCTATTTCGTTCATCC
ATAGTTGCCTGACTCCCCGTCGTGTAGATAACTACGATACGGGAGGGCTT
ACCATCTGGCCCCAGTGCTGCAATGATACCGCGAGACCCACGCTCACCGG
CTCCAGATTTATCAGCAATAAACCAGCCAGCCGGAAGGGCCGAGCGCAGA
AGTGGTCCTGCAACTTTATCCGCCTCCATCCAGTCTATTAATTGTTGCCG
GGAAGCTAGAGTAAGTAGTTCGCCAGTTAATAGTTTGCGCAACGTTGTTG
CCATTGCTACAGGCATCGTGGTGTCACGCTCGTCGTTTGGTATGGCTTCA
TTCAGCTCCGGTTCCCAACGATCAAGGCGAGTTACATGATCCCCCATGTT
GTGCAAAAAAGCGGTTAGCTCCTTCGGTCCTCCGATCGTTGTCAGAAGTA
AGTTGGCCGCAGTGTTATCACTCATGGTTATGGCAGCACTGCATAATTCT
CTTACTGTCATGCCATCCGTAAGATGCTTTTCTGTGACTGGTGAGTACTC
AACCAAGTCATTCTGAGAATAGTGTATGCGGCGACCGAGTTGCTCTTGCC
CGGCGTCAATACGGGATAATACCGCGCCACATAGCAGAACTTTAAAAGTG
CTCATCATTGGAAAACGTTCTTCGGGGCGAAAACTCTCAAGGATCTTACC
GCTGTTGAGATCCAGTTCGATGTAACCCACTCGTGCACCCAACTGATCTT
CAGCATCTTTTACTTTCACCAGCGTTTCTGGGTGAGCAAAAACAGGAAGG
CAAAATGCCGCAAAAAAGGGAATAAGGGCGACACGGAAATGTTGAATACT
CATACTCTTCCTTTTTCAATATTATTGAAGCATTTATCAGGGTTATTGTC
TCATGAGCGGATACATATTTGAATGTATTTAGAAAAATAAACAAATAGGG
GTTCCGCGCACATTTCCCCGAAAAGTGCCACCTGACGCGCCCTGTAGCGG
GATCCATTTTATTTAATATACTAAATAATAAAAAAGTTAAAAAATGATCA
TTGGATAAATTTTTTATAATTATAAATAAAGATAATAATTTTTTTTTTAA
CAAAACTAAAAATAAAAATAATAAAATAATTGTTAAAATAGGTTTTTTTT
TTTTTTTTTTTTTTTTAATAAATGGTATTTATTAATTTATTTGTTGTGTG
TGTTTTTTTTTTTATAATATTTTTTTTTTTAGCATTGAATTAAGAAGAAA
TCAAATTGATGCGGCCGCTTATGCATCATCTTGTGCACCTGAAAACCAAT
ACCAATACATTGCTGTTTCATTTGAAACTTGTGGTCTTGTTTTATATGTA
AATAAATCAATACCACCTAATGTAAAACCACATTTTGCATATAAATTACA
TGCTGGAACATTATTTGTTTGTGTTTCTAATCTAATACCTAATAATTGTC
TTGATAATGCCCATTTTTTTGCAAATTCAATTAATGAATGTGCAACACCT
TTACCTCTATGTGTATGTGAAACAACAATATGTTCAATTGATGCTAAATC
ATTCCATGTTGAATTTAATTCAATTTTACCAACTAATTCTTGATCAATAA
ATGCACCATAACATGCTGAATCTTCATCTGAATCATCATCTGAAATATAA
TCTTTTCTATATGGTGAAACTGATCTTGTTGATAATTCAAAACCTTGATC
TGATAAATGAACATCAAAAACTTCTCTAACAATAAAATGATTTTCTGCAT
CTAATGTTTCTGCAACTTGTTCTGGAATAACTGAAATTTTCATTTTTTGC
TAGCTGTGAAATTAGTTTAAAATACAAATAAAGAGTTATAATAATATACA

GTTGAATAAAAAAAAAAAAAAATGAATTGGAAAATTTATTTTTATATGAAG
AAAAAAAAATTTTGAAAAAAAAAAAAAAATTAAAAAAAAAAAAAAAAAAAA
AAAAAATTTAAAATTATTCCACTGTGGGGGGCCCCAAATTTTATTTAAAA
AAAAAAAAAAAAATGGGTCCCTTTTGGGGGGTTGGAAAAAAAAAAAAAAAA
AAAAAAAAAAAATTGAAAATATAATGTTAGTCATATGATTAATCATT

pDM1489 – Hyg

CTCGAGACTAGAGCTAGATAAAAAAAATTTTTATTTATTTTTATTTATTT
TGAATTAAATAGATTACAAATTAATTAATCCCATCAAATCTTTAAAAAAA
AATGGTTTAAAAAAACTTGGGTTGGTTAATTATTATTTGAAAATTTTAAA
ACCCAAATTAAAAAAAAAAAAAATGGGATTCAAAAATTTTTTTTTTTTTT
TTTTTTTTTTTTTTTTTTTTTTCAGATTGCATAAAAAGATTTTTTTTTT
TTTTTTTTCTTATTTCTTAAAACAAATAAATTAAATTAAATAAAAAAATAA
AAATCAGATCCAAAATGCATCATCATCATCACCATCATCATGATATAGGT
ACAATGCCAAAGAAGAAACGTAAGGTTAGTAATTTATTAACAGTGCATCA
GAATTTGCCAGCATTACCAGTAGATGCTACATCAGATGAAGTGCGTAAAA
ATTTGATGGATATGTTCAGAGATAGACAGGCTTTTAGTGAACACACATGG
AAGATGTTATTAAGCGTATGCCGTTCATGGGCCGCTTGGTGTAAACTCAA
CAATAGAAAATGGTTTCCAGCTGAACCAGAGGATGTAAGAGATTATTTAC
TCTACTTACAAGCAAGAGGTTTAGCTGTAAAAACAATTCAACAGCATTTA
GGTCAATTAAATATGTTACATCGTAGATCGGGTTTGCCAAGACCATCAGA
TTCAAATGCTGTTTCATTAGTCATGCGTCGTATACGTAAAGAGAATGTTG
ATGCAGGAGAAAGAGCTAAGCAAGCACTAGCTTTTGAACGTACAGATTTT
GATCAAGTTCGTAGTTTAATGGAAAATTCAGACAGATGTCAAGATATTAG
AAATTTAGCTTTCTTAGGGATTGCATACAATACATTGCTTCGTATTGCAG
AAATTGCCAGAATTAGAGTTAAAGATATCAGTCGTACAGACGGTGGTAGA
ATGTTAATACATATCGGTAGGACTAAAACTTTAGTTAGTACAGCCGGTGT
CGAAAAAGCATTATCATTGGGTGTAACTAAATTGGTAGAGCGTTGGATTA
GTGTAAGTGGTGTTGCCGATGATCCAAATAATTATCTTTTCTGTAGAGTT
AGAAAGAATGGAGTTGCTGCACCTAGCGCTACATCTCAACTCTCAACAAG
AGCTTTGGAAGGTATCTTTGAAGCTACTCATAGATTAATTTACGGTGCTA
AAGATGACTCAGGTCAACGTTATTTAGCATGGTCTGGTCACTCAGCAAGA
GTCGGTGCAGCACGTGATATGGCTCGTGCTGGTGTATCAATACCAGAAAT
TATGCAAGCTGGTGGATGGACTAATGTGAATATAGTGATGAATTATATTA
GGAATTTAGATTCTGAAACAGGTGCAATGGTTAGATTACTACAAGATGGT
GATTAAGCTAGTTAAATAAATAAATTATTTAATAAATAATAAAAAAACAA
ATTGTTGTAATAATCTAATATTTTCTTTTTTTTTTAATTTTTTTTTTTTA
AATCTTAATAATTATTAAGTTATTTTAATTTTTTTTTTTTTTTTTTTTTT
TTTTTTTTTTTTTTTCTATCAAAAAAATCAAATATATTTAAAAAATTTA
TTATTTACAGATACATTTTGAATGGTGAAGATAAATATATGCATTAGATG
TAAAACAGCCAAAGAGTATGAAAATCAAAAAGATAAAGCTTATCGATTTC
GAAAAAGTAAATAGCAATTATTACAAAATTCAATCCGAATCTACCCAAAT
AAATTCCAATGAAATTGCCGATTTAAAAAAGTTTATTAAAGAAGAAGTCA
ATAAAACTTCTTCCAAAATTGATTTCTTTTTAGTTTCTTCAACAGATGCC
CTTTCAAATCCAGAAAATTATTCTCTCTTAGAAGTAAAGTGTATTAATTG
TCATTCTTTGTGTCAAGGAAAAAATTTATATATTTCATGTACAAGAGATG
GATGTCAAAACAATATTTGCTATAATTGTTTAGGAATAAACATAAACATA
TATAATGTTGTTATTAATTCTAAACTTTGCCCTCCATGTTTCAATGATTC
GGTAATCAACAAGAAGTGTGCCATGTGTAGTAAGAACGGAACTAAATGTA

ATTTGAACCAAGAATGTAAACTTCATCTTTGTGCACAGTGTTCTAAAAAG
TGTCTATACATTCTGAGAGTCAAAACTAATTAAATAAAATATAAACTTAA
TTTCTAAATAAACTCATTTAAAAATATTTAAATAATATGAATTTATAACT
GTAATTATTGTATTAAAAAATTATATAATTATTTAATGTTAAAAATGTAT
TAAAATAATTATAAAAAAATATAACAAAAATTTTCGTAAAAATAATTTGT
AAAAAAGCTATTAAAAATATTATGAAAAAAAAATTAAAAAAATTATTAAA
TTGTTTTTGTAATTAAGCTATTAAAATAATTATAAAAAAAAAATTTTTAA
AATTTTAAAAATATTTTTTGTAAAAAAGTATTAAAATAATTATGAAAAAA
AAATTTTCTAAAAAATTAAAAAAAAAATTAAAATATATTTTATGTTAAAA
ACGTATTAAAATAACTATTAAAAAAATTATATTTAAAAAAGTATTAACTT
TTTTTTAGGTGTGGTTGTGGGGTGGGGTTTAATATATTATAATAAAAAAT
TATTTTTTGTTCATTTATTATTTTCATTGTATATAATGTACTCAACAACG
TTATTATTTTTTCTTTTTTTTTTTATTGTATCAAAATCTTCTGTTCTTCA
AAATGATCAGATTGAAGTAAAATATTTTCAACTTCTTATTGTTATGTATC
AAAAAGAAAACTGTGTTGAAAAGTCAATGACAGGCGCCGTAATTTATGAT
GAATGTAATATTCATGGAAGAGTTGAAACAAATAGTACTCATGCGCTTTT
TTATGATGACATTGAAACAAATAATTCAAGATGTAACAATTTTCGTAATT
TAACAAACTTAATTAAACTTAATGAATGTATTAATGACGAGTTTGGAGAG
TCTATTCTTTATAAAGAATATAATGAAACTGATGATGGTTATTTGTTTAG
AGTGGAAGACAGCTTTGTTGAAATTACTTCTCTTTCAATGGATTGTACAA
AAAATAGTAAAACAATTATTGAAAAATTCAACATTTGTTCAAAATTTGAA
AATGTATATCATATTACAAACATTACACAAGAGAAATCCAATAGATTTAC
ATGTACAGATCCATTGTGCCACTATTGTAAGAATGAAAACATTCAAAACA
ATCTTGATTTTAAAACAACAAAGTGTACTCCAAAGTATGGTGCATCTGAT
TCTGAATTTTTATCAACAATTTACAATCCAAAGCTCGATGGCTCAAATAA
CGGTATGGAAAAGTCAGTAACTCAAGAAAAAAACATTTCAAATAATTTAA
AAATTAATATATATTTAATTTTCTTTTTAATTATTTTTTTAATTAAATAA
AGTTTTATTATTTTTTAAGAGTAATTATTGCTCTTTTTTCATTTGAAACA
CCAGAAGCTAAACGTAATTGTTGTTGACTGAAATTTTTTATTTTTTTTGG
GGTAATAGGATTTCCTTTTTTATGAAGATTAATATCTTTGACTCGTGAAA
CATTCTTTTTAACTTTTGTTTTTTCTGTTGGTTTATCATTTGTTTTTTCA
CTAATTTCAATACCATCTTGACGTTCATTCATAACTTCATCTTTTTTTTT
TCCTGTTTCTGTATCTTCTTCTATTTTTTTTTCTTTATCTTTTTCTTTAT
CTTCTTCTTGTTCTTCCTCTTCTTTTTCTTCTTCTGATACTGCAGGTGTT
TCTTCTTCTTCTTCTTCCGATATTGTCGGTTTTTCTACTTCTTCTTCTTG
TTCTTCTTCTTCTTCTTCTTCTTCTTCTTCTTCTTCTTCTTCTTCTTCTT
CTTCCGGTAATTTATTAATTATATTTCTTTTTTTATATGAATTACGTTTG
GTTTGTGCAGTAATTTCCTTACATAGAGTGCAGCTTTCAAGAAAAATTTC
AATTTCTTCGTTTGTTGCATAATAACCACTGTCTTTGATATGATTAAACA
TTTTTGATTTTCTTAAATGCTTTCCTTCTTTAATATGAAAATTATCGAAT
TCTAATTCATTAAGAACAATAAGCTCCCCTAATTTAAAAAATTAGTTAAA
ATAAATTAAAATGAACATGTATAAAGATGGATTTTACCATTTTTTGAAAT
TCTAAATAACTTTTCTTCATCTCCAATCTTTTTGACTGAAAAACGATTTT
TAATTGAAGTTATTGTTCTGTGAGTGTTTTGAATCGCCCATTTCTCTAAA
TCAGTTTGAGATAGTGTTTTATAATCTGAATTGTTATACACAACTTTTGC
TCTATTAACCAAATATTTAAAGATTTCATCATCAACTGAATATTTTGACT
TTACGATTCTTGTCCAAAAAACAATTTCTACTACTATCATTTTTTATTTA
TAAAATAATTTAAATACAAAAATGAATTTTTTTTTTTTTAAAAAAAAAAA
AATTTGAAAAAAAAAAAAAAAAAAATTTTAAAAAAAAAAAAAAAAAAAAAA
AAAAAAAAAAAATCAAATAAAAAGTAAAAAATAAAAACCGAAAAACATTC

ATTGTAATTTCAAATGTCGAGGCCGGCAGAGGCGGTTTGCGTATTGGGCG
CTCTTCCGCTTCCTCGCTCACTGACTCGCTGCGCTCGGTCGTTCGGCTGC
GGCGAGCGGTATCAGCTCACTCAAAGGCGGTAATACGGTTATCCACAGAA
TCAGGGGATAACGCAGGAAAGAACATGTGAGCAAAAGGCCAGCAAAAGGC
CAGGAACCGTAAAAAGGCCGCGTTGCTGGCGTTTTTCCATAGGCTCCGCC
CCCCTGACGAGCATCACAAAAATCGACGCTCAAGTCAGAGGTGGCGAAAC
CCGACAGGACTATAAAGATACCAGGCGTTTCCCCCTGGAAGCTCCCTCGT
GCGCTCTCCTGTTCCGACCCTGCCGCTTACCGGATACCTGTCCGCCTTTC
TCCCTTCGGGAAGCGTGGCGCTTTCTCATAGCTCACGCTGTAGGTATCTC
AGTTCGGTGTAGGTCGTTCGCTCCAAGCTGGGCTGTGTGCACGAACCCCC
CGTTCAGCCCGACCGCTGCGCCTTATCCGGTAACTATCGTCTTGAGTCCA
ACCCGGTAAGACACGACTTATCGCCACTGGCAGCAGCCACTGGTAACAGG
ATTAGCAGAGCGAGGTATGTAGGCGGTGCTACAGAGTTCTTGAAGTGGTG
GCCTAACTACGGCTACACTAGAAGGACAGTATTTGGTATCTGCGCTCTGC
TGAAGCCAGTTACCTTCGGAAAAAGAGTTGGTAGCTCTTGATCCGGCAAA
CAAACCACCGCTGGTAGCGGTGGTTTTTTTGTTTGCAAGCAGCAGATTAC
GCGCAGAAAAAAAGGATCTCAAGAAGATCCTTTGATCTTTTCTACGGGGT
CTGACGCTCAGTGGAACGAAAACTCACGTTAAGGGATTTTGGTCATGAGA
TTATCAAAAAGGATCTTCACCTAGATCCTTTTAAATTAAAAATGAAGTTT
TAAATCAATCTAAAGTATATATGAGTAAACTTGGTCTGACAGTTACCAAT
GCTTAATCAGTGAGGCACCTATCTCAGCGATCTGTCTATTTCGTTCATCC
ATAGTTGCCTGACTCCCCGTCGTGTAGATAACTACGATACGGGAGGGCTT
ACCATCTGGCCCCAGTGCTGCAATGATACCGCGAGACCCACGCTCACCGG
CTCCAGATTTATCAGCAATAAACCAGCCAGCCGGAAGGGCCGAGCGCAGA
AGTGGTCCTGCAACTTTATCCGCCTCCATCCAGTCTATTAATTGTTGCCG
GGAAGCTAGAGTAAGTAGTTCGCCAGTTAATAGTTTGCGCAACGTTGTTG
CCATTGCTACAGGCATCGTGGTGTCACGCTCGTCGTTTGGTATGGCTTCA
TTCAGCTCCGGTTCCCAACGATCAAGGCGAGTTACATGATCCCCCATGTT
GTGCAAAAAAGCGGTTAGCTCCTTCGGTCCTCCGATCGTTGTCAGAAGTA
AGTTGGCCGCAGTGTTATCACTCATGGTTATGGCAGCACTGCATAATTCT
CTTACTGTCATGCCATCCGTAAGATGCTTTTCTGTGACTGGTGAGTACTC
AACCAAGTCATTCTGAGAATAGTGTATGCGGCGACCGAGTTGCTCTTGCC
CGGCGTCAATACGGGATAATACCGCGCCACATAGCAGAACTTTAAAAGTG
CTCATCATTGGAAAACGTTCTTCGGGGCGAAAACTCTCAAGGATCTTACC
GCTGTTGAGATCCAGTTCGATGTAACCCACTCGTGCACCCAACTGATCTT
CAGCATCTTTTACTTTCACCAGCGTTTCTGGGTGAGCAAAAACAGGAAGG
CAAAATGCCGCAAAAAAGGGAATAAGGGCGACACGGAAATGTTGAATACT
CATACTCTTCCTTTTTCAATATTATTGAAGCATTTATCAGGGTTATTGTC
TCATGAGCGGATACATATTTGAATGTATTTAGAAAAATAAACAAATAGGG
GTTCCGCGCACATTTCCCCGAAAAGTGCCACCTGACGCGCCCTGTAGCGG
GATCCATTTTATTTAATATACTAAATAATAAAAAAGTTAAAAAATGATCA
TTGGATAAATTTTTTATAATTATAAATAAAGATAATAATTTTTTTTTTAA
CAAAACTAAAAATAAAAATAATAAAATAATTGTTAAAATAGGTTTTTTTT
TTTTTTTTTTTTTTTTAATAAATGGTATTTATTAATTTATTTGTTGTGTG
TGTTTTTTTTTTTATAATATTTTTTTTTTTTAGCATTGAATTAAGAAGAAA
TCAAATTGATGCGGCCGCTTAGTTAGCCTCCCCCATCTCCCGATCCGGAC
GAGTGCTGGGGCGTCGGTTTCCACTATCGGCGAGTACTTCTACACAGCCA
TCGGTCCAGACGGCCGCGCTTCTGCGGGCGATTTGTGTACGCCCGACAGT
CCCGGCTCCGGATCGGACGATTGCGTCGCATCGACCCTGCGCCCAAGCTG
CATCATCGAAATTGCCGTCAACCAAGCTCTGATAGAGTTGGTCAAGACCA

ATGCGGAGCATATACGCCCGGAGCCGCGGCGATCCTGCAAGCTCCGGATG
CCTCCGCTCGAAGTAGCGCGTCTGCTGCTCCATACAAGCCAACCACGGCC
TCCAGAAGAAGATGTTGGCGACCTCGTATTGGGAATCCCCGAACATCGCC
TCGCTCCAGTCAATGACCGCTGTTATGCGGCCATTGTCCGTCAGGACATT
GTTGGAGCCGAAATCCGCGTGCACGAGGTGCCGGACTTCGGGGCAGTCCT
CGGCCCAAAGCATCAGCTCATCGAGAGCCTGCGCGACGGACGCACTGACG
GTGTCGTCCATCACAGTTTGCCAGTGATACACATGGGGATCAGCAATCGC
GCATATGAAATCACGCCATGTAGTGTATTGACCGATTCCTTGCGGTCCGA
ATGGGCCGAACCCGCTCGTCTGGCTAAGATCGGCCGCAGCGATCGCATCC
ATGGCCTCCGCGACCGGCTGCAGAACAGCGGGCAGTTCGGTTTCAGGCAG
GTCTTGCAACGTGACACCCTGTGCACGGCGGGAGATGCAATAGGTCAGGC
TCTCGCTGAACTCCCCAATGTCAAGCACTTCCGGAATCGGGAGCGCGGCC
GATGCAAAGTGCCGATAAACATAACGATCTTTGTAGAAACCATCGGCGCA
GCTATTTACCCGCAGGACATATCCACGCCCTCCTACATCGAAGCTGAAAG
CACGAGATTCTTCGCCCTCCGAGAGCTGCATCAGGTCGGAGACGCTGTCG
AACTTTTCGATCAGAAACTTCTCGACAGACGTCGCGGTGAGTTCAGGCTT
TTTCATTAGGTCTTGTTGAGAAATGTTAAATTGATCCATTTTTTGCTAGC
TGTGAAATTAGTTTAAAATACAAATAAAGAGTTATAATAATATACAGTTG
AATAAAAAAAAAAAAAATGAATTGGAAAATTTATTTTTATATGAAGAAAA
AAAAATTTTGAAAAAAAAAAAAAAATTAAAAAAAAAAAAAAAAAAAAAAA
AATTTAAAATTATTCCACTGTGGGGGGCCCCAAATTTTATTTAAAAAAAA
AAAAAAAATGGGTCCCTTTTGGGGGGTTGGAAAAAAAAAAAAAAAAAAAA
AAAAAAATTGAAAATATAATGTTAGTCATATGATTAATCATT

pDM1488 – G418

CTCGAGACTAGAGCTAGATAAAAAAAATTTTTATTTATTTTTATTTATTT
TGAATTAAATAGATTACAAATTAATTAATCCCATCAAATCTTTAAAAAAA
AATGGTTTAAAAAAACTTGGGTTGGTTAATTATTATTTGAAAATTTTAAA
ACCCAAATTAAAAAAAAAAATGGGATTCAAAAATTTTTTTTTTTTTTTTT
TTTTTTTTTTTTTTTTTTTTTTTCAGATTGCATAAAAAGATTTTTTTTTT
TTTTTTTTCTTATTTCTTAAAACAAATAAATTAAATTAAATAAAAAATAA
AAATCAGATCCAAAATGCATCATCATCATCACCATCATCATGATATAGGT
ACAATGCCAAAGAAGAAACGTAAGGTTAGTAATTTATTAACAGTGCATCA
GAATTTGCCAGCATTACCAGTAGATGCTACATCAGATGAAGTGCGTAAAA
ATTTGATGGATATGTTCAGAGATAGACAGGCTTTTAGTGAACACACATGG
AAGATGTTATTAAGCGTATGCCGTTCATGGGCCGCTTGGTGTAAACTCAA
CAATAGAAAATGGTTTCCAGCTGAACCAGAGGATGTAAGAGATTATTTAC
TCTACTTACAAGCAAGAGGTTTAGCTGTAAAAACAATTCAACAGCATTTA
GGTCAATTAAATATGTTACATCGTAGATCGGGTTTGCCAAGACCATCAGA
TTCAAATGCTGTTTCATTAGTCATGCGTCGTATACGTAAAGAGAATGTTG
ATGCAGGAGAAAGAGCTAAGCAAGCACTAGCTTTTGAACGTACAGATTTT
GATCAAGTTCGTAGTTTAATGGAAAATTCAGACAGATGTCAAGATATTAG
AAATTTAGCTTTCTTAGGGATTGCATACAATACATTGCTTCGTATTGCAG
AAATTGCCAGAATTAGAGTTAAAGATATCAGTCGTACAGACGGTGGTAGA
ATGTTAATACATATCGGTAGGACTAAAACTTTAGTTAGTACAGCCGGTGT
CGAAAAAGCATTATCATTGGGTGTAACTAAATTGGTAGAGCGTTGGATTA
GTGTAAGTGGTGTTGCCGATGATCCAAATAATTATCTTTTCTGTAGAGTT
AGAAAGAATGGAGTTGCTGCACCTAGCGCTACATCTCAACTCTCAACAAG
AGCTTTGGAAGGTATCTTTGAAGCTACTCATAGATTAATTTACGGTGCTA

AAGATGACTCAGGTCAACGTTATTTAGCATGGTCTGGTCACTCAGCAAGA
GTCGGTGCAGCACGTGATATGGCTCGTGCTGGTGTATCAATACCAGAAAT
TATGCAAGCTGGTGGATGGACTAATGTGAATATAGTGATGAATTATATTA
GGAATTTAGATTCTGAAACAGGTGCAATGGTTAGATTACTACAAGATGGT
GATTAAGCTAGTTAAATAAATAAATTATTTAATAAATAATAAAAAAACAA
ATTGTTGTAATAATCTAATATTTTCTTTTTTTTTTAATTTTTTTTTTTTA
AATCTTAATAATTATTAAGTTATTTTAATTTTTTTTTTTTTTTTTTTTTT
TTTTTTTTTTTTTTTTCTATCAAAAAAATCAAATATATTTAAAAAATTTA
TTATTTACAGATACATTTTGAATGGTGAAGATAAATATATGCATTAGATG
TAAAACAGCCAAAGAGTATGAAAATCAAAAAGATAAAGCTTATCGATTTC
GAAAAAGTAAATAGCAATTATTACAAAATTCAATCCGAATCTACCCAAAT
AAATTCCAATGAAATTGCCGATTTAAAAAAGTTTATTAAAGAAGAAGTCA
ATAAAACTTCTTCCAAAATTGATTTCTTTTTAGTTTCTTCAACAGATGCC
CTTTCAAATCCAGAAAATTATTCTCTCTTAGAAGTAAAGTGTATTAATTG
TCATTCTTTGTGTCAAGGAAAAAATTTATATATTTCATGTACAAGAGATG
GATGTCAAAACAATATTTGCTATAATTGTTTAGGAATAAACATAAACATA
TATAATGTTGTTATTAATTCTAAACTTTGCCCTCCATGTTTCAATGATTC
GGTAATCAACAAGAAGTGTGCCATGTGTAGTAAGAACGGAACTAAATGTA
ATTTGAACCAAGAATGTAAACTTCATCTTTGTGCACAGTGTTCTAAAAAG
TGTCTATACATTCTGAGAGTCAAAACTAATTAAATAAAATATAAACTTAA
TTTCTAAATAAACTCATTTAAAAATATTTAAATAATATGAATTTATAACT
GTAATTATTGTATTAAAAAATTATATAATTATTTAATGTTAAAAATGTAT
TAAAATAATTATAAAAAAATATAACAAAAATTTTCGTAAAAATAATTTGT
AAAAAAGCTATTAAAAATATTATGAAAAAAAAATTAAAAAAATTATTAAA
TTGTTTTTGTAATTAAGCTATTAAAATAATTATAAAAAAAAAATTTTTAA
AATTTTAAAAATATTTTTTGTAAAAAAGTATTAAAATAATTATGAAAAAA
AAATTTTCTAAAAAATTAAAAAAAAAATTAAAATATATTTTATGTTAAAA
ACGTATTAAAATAACTATTAAAAAAATTATATTTAAAAAAGTATTAACTT
TTTTTTAGGTGTGGTTGTGGGGTGGGGTTTAATATATTATAATAAAAAAT
TATTTTTTGTTCATTTATTATTTTCATTGTATATAATGTACTCAACAACG
TTATTATTTTTTCTTTTTTTTTTTATTGTATCAAAATCTTCTGTTCTTCA
AAATGATCAGATTGAAGTAAAATATTTTCAACTTCTTATTGTTATGTATC
AAAAAGAAAACTGTGTTGAAAAGTCAATGACAGGCGCCGTAATTTATGAT
GAATGTAATATTCATGGAAGAGTTGAAACAAATAGTACTCATGCGCTTTT
TTATGATGACATTGAAACAAATAATTCAAGATGTAACAATTTTCGTAATT
TAACAAACTTAATTAAACTTAATGAATGTATTAATGACGAGTTTGGAGAG
TCTATTCTTTATAAAGAATATAATGAAACTGATGATGGTTATTTGTTTAG
AGTGGAAGACAGCTTTGTTGAAATTACTTCTCTTTCAATGGATTGTACAA
AAAATAGTAAAACAATTATTGAAAAATTCAACATTTGTTCAAAATTTGAA
AATGTATATCATATTACAAACATTACACAAGAGAAATCCAATAGATTTAC
ATGTACAGATCCATTGTGCCACTATTGTAAGAATGAAAACATTCAAAACA
ATCTTGATTTTAAAACAACAAAGTGTACTCCAAAGTATGGTGCATCTGAT
TCTGAATTTTTATCAACAATTTACAATCCAAAGCTCGATGGCTCAAATAA
CGGTATGGAAAAGTCAGTAACTCAAGAAAAAAACATTTCAAATAATTTAA
AAATTAATATATATTTAATTTTCTTTTTAATTATTTTTTTAATTAAATAA
AGTTTATTATTTTTTTAAGAGTAATTATTGCTCTTTTTTCATTTGAAACA
CCAGAAGCTAAACGTAATTGTTGTTGACTGAAATTTTTATTTTTTTTTGG
GGTAATAGGATTTCCTTTTTTATGAAGATTAATATCTTTGACTCGTGAAA
CATTCTTTTTAACTTTTGTTTTTTCTGTTGGTTTATCATTTGTTTTTTCA
CTAATTTCAATACCATCTTGACGTTCATTCATAACTTCATCTTTTTTTTT

TCCTGTTTCTGTATCTTCTTCTATTTTTTTTTCTTTATCTTTTTCTTTAT
CTTCTTCTTGTTCTTCCTCTTCTTTTTCTTCTTCTGATACTGCAGGTGTT
TCTTCTTCTTCTTCTTCCGATATTGTCGGTTTTTCTACTTCTTCTTCTTG
TTCTTCTTCTTCTTCTTCTTCTTCTTCTTCTTCTTCTTCTTCTTCTTCTT
CTTCCGGTAATTTATTAATTATATTTCTTTTTTTATATGAATTACGTTTG
GTTTGTGCAGTAATTTCCTTACATAGAGTGCAGCTTTCAAGAAAAATTTC
AATTTCTTCGTTTGTTGCATAATAACCACTGTCTTTGATATGATTAAACA
TTTTTGATTTTCTTAAATGCTTTCCTTCTTTAATATGAAAATTATCGAAT
TCTAATTCATTAAGAACAATAAGCTCCCCTAATTTAAAAAATTAGTTAAA
ATAAATTAAAATGAACATGTATAAAGATGGATTTTACCATTTTTTGAAAT
TCTAAATAACTTTTCTTCATCTCCAATCTTTTTGACTGAAAAACGATTTT
TAATTGAAGTTATTGTTCTGTGAGTGTTTTGAATCGCCCATTTCTCTAAA
TCAGTTTGAGATAGTGTTTTATAATCTGAATTGTTATACACAACTTTTGC
TCTATTAACCAAATATTTAAAGATTTCATCATCAACTGAATATTTTGACT
TTACGATTCTTGTCCAAAAAACAATTTCTACTACTATCATTTTTTATTTA
TAAAATAATTTAAATACAAAAATGAATTTTTTTTTTTTTAAAAAAAAAAA
AATTTGAAAAAAAAAAAAAAAAAAATTTTAAAAAAAAAAAAAAAAAAAAAA
AAAAAAAAAAAAATCAAATAAAAAGTAAAAAATAAAAACCGAAAAACATTC
ATTGTAATTTCAAATGTCGAGGCCGGCAGAGGCGGTTTGCGTATTGGGCG
CTCTTCCGCTTCCTCGCTCACTGACTCGCTGCGCTCGGTCGTTCGGCTGC
GGCGAGCGGTATCAGCTCACTCAAAGGCGGTAATACGGTTATCCACAGAA
TCAGGGGATAACGCAGGAAAGAACATGTGAGCAAAAGGCCAGCAAAAGGC
CAGGAACCGTAAAAAGGCCGCGTTGCTGGCGTTTTTCCATAGGCTCCGCC
CCCCTGACGAGCATCACAAAAATCGACGCTCAAGTCAGAGGTGGCGAAAC
CCGACAGGACTATAAAGATACCAGGCGTTTCCCCCTGGAAGCTCCCTCGT
GCGCTCTCCTGTTCCGACCCTGCCGCTTACCGGATACCTGTCCGCCTTTC
TCCCTTCGGGAAGCGTGGCGCTTTCTCATAGCTCACGCTGTAGGTATCTC
AGTTCGGTGTAGGTCGTTCGCTCCAAGCTGGGCTGTGTGCACGAACCCCC
CGTTCAGCCCGACCGCTGCGCCTTATCCGGTAACTATCGTCTTGAGTCCA
ACCCGGTAAGACACGACTTATCGCCACTGGCAGCAGCCACTGGTAACAGG
ATTAGCAGAGCGAGGTATGTAGGCGGTGCTACAGAGTTCTTGAAGTGGTG
GCCTAACTACGGCTACACTAGAAGGACAGTATTTGGTATCTGCGCTCTGC
TGAAGCCAGTTACCTTCGGAAAAAGAGTTGGTAGCTCTTGATCCGGCAAA
CAAACCACCGCTGGTAGCGGTGGTTTTTTTGTTTGCAAGCAGCAGATTAC
GCGCAGAAAAAAAGGATCTCAAGAAGATCCTTTGATCTTTTCTACGGGGT
CTGACGCTCAGTGGAACGAAAACTCACGTTAAGGGATTTTGGTCATGAGA
TTATCAAAAAGGATCTTCACCTAGATCCTTTTAAATTAAAAATGAAGTTT
TAAATCAATCTAAAGTATATATGAGTAAACTTGGTCTGACAGTTACCAAT
GCTTAATCAGTGAGGCACCTATCTCAGCGATCTGTCTATTTCGTTCATCC
ATAGTTGCCTGACTCCCCGTCGTGTAGATAACTACGATACGGGAGGGCTT
ACCATCTGGCCCCAGTGCTGCAATGATACCGCGAGACCCACGCTCACCGG
CTCCAGATTTATCAGCAATAAACCAGCCAGCCGGAAGGGCCGAGCGCAGA
AGTGGTCCTGCAACTTTATCCGCCTCCATCCAGTCTATTAATTGTTGCCG
GGAAGCTAGAGTAAGTAGTTCGCCAGTTAATAGTTTGCGCAACGTTGTTG
CCATTGCTACAGGCATCGTGGTGTCACGCTCGTCGTTTGGTATGGCTTCA
TTCAGCTCCGGTTCCCAACGATCAAGGCGAGTTACATGATCCCCCATGTT
GTGCAAAAAAGCGGTTAGCTCCTTCGGTCCTCCGATCGTTGTCAGAAGTA
AGTTGGCCGCAGTGTTATCACTCATGGTTATGGCAGCACTGCATAATTCT
CTTACTGTCATGCCATCCGTAAGATGCTTTTCTGTGACTGGTGAGTACTC
AACCAAGTCATTCTGAGAATAGTGTATGCGGCGACCGAGTTGCTCTTGCC

CGGCGTCAATACGGGATAATACCGCGCCACATAGCAGAACTTTAAAAGTG
CTCATCATTGGAAAACGTTCTTCGGGGCGAAAACTCTCAAGGATCTTACC
GCTGTTGAGATCCAGTTCGATGTAACCCACTCGTGCACCCAACTGATCTT
CAGCATCTTTTACTTTCACCAGCGTTTCTGGGTGAGCAAAAACAGGAAGG
CAAAATGCCGCAAAAAAGGGAATAAGGGCGACACGGAAATGTTGAATACT
CATACTCTTCCTTTTTCAATATTATTGAAGCATTTATCAGGGTTATTGTC
TCATGAGCGGATACATATTTGAATGTATTTAGAAAAATAAACAAATAGGG
GTTCCGCGCACATTTCCCCGAAAAGTGCCACCTGACGCGCCCTGTAGCGG
GATCCATTTTATTTAATATACTAAATAATAAAAAAGTTAAAAAATGATCA
TTGGATAAATTTTTTATAATTATAAATAAAGATAATAATTTTTTTTTTAA
CAAAACTAAAAATAAAAATAATAAAATAATTGTTAAAATAGGTTTTTTTT
TTTTTTTTTTTTTTTTAATAAATGGTATTTATTAATTTATTTGTTGTGTG
TGTTTTTTTTTTTATAATATTTTTTTTTTTAGCATTGAATTAAGAAGAAA
TCAAATTGATGCGGCCGCTCAGAAGAACTCGTCAAGAAGGCGATAGAAGG
CGATGCGCTGCGAATCGGGAGCGGCGATACCGTAAAGCACGAGGAAGCGG
TCAGCCCATTCGCCGCCAAGCTCTTCAGCAATATCACGGGTAGCCAACGC
TATGTCCTGATAGCGGTCCGCCACACCCAGCCGTCCACAGTCGATGAATC
CAGAAAAGCGGCCATTTTCCACCATGATATTCGGCAAGCAGGCATCGCCA
TGGGTCACGACGAGATCCTCGCCGTCGGGCATGCGCGCCTTGAGCCTGGC
GAACAGTTCGGCTGGCGCGAGCCCCTGATGCTCTTCGTCCAGATCATCCT
GATCGACAAGACCGGCTTCCATCCGAGTACGTGCTCGCTCGATGCGATGT
TTCGCTTGGTGGTCGAATGGGCAGGTAGCCGGATCAAGCGTATGCAGCCG
CCGCATTGCATCAGCCATGATGGATACTTTCTCGGCAGGAGCAAGGTGAG
ATGACAGGAGATCCTGCCCCGGCACTTCGCCCAATAGCAGCCAGTCCCTT
CCCGCTTCAGTGACAACGTCGAGCACAGCTGCGCAAGGAACGCCCGTCGT
GGCCAGCCACGATAGCCGCGCTGCCTCGTCCTGCAGTTCATTCAGGGCAC
CGGACAGGTCGGTCTTGACAAAAAGAACCGGGCGCCCCTGCGCTGACAGC
CGGAACACGGCGGCATCAGAGCAGCCGATTGTCTGTTGTGCCCAGTCATA
GCCGAATAGCCTCTCCACCCAAGCGGCCGGAGAACCTGCGTGCAATCCAT
CTTGTTCAATCATGCGAAACGATCCAGCTTGAACATCTTCACCATCCATT
TTTTGCTAGCTGTGAAATTAGTTTAAAATACAAATAAAGAGTTATAATAA
TATACAGTTGAATAAAAAAAAAAAAAATGAATTGGAAAATTTATTTTTAT
ATGAAGAAAAAAAAATTTTGAAAAAAAAAAAAAAATTAAAAAAAAAAAAA
AAAAAAAAAAAATTTAAAATTATTCCACTGTGGGGGGCCCCAAATTTTAT
TTAAAAAAAAAAAAAAAATGGGTCCCTTTTGGGGGGTTGGAAAAAAAAAA
AAAAAAAAAAAAAAAAATTGAAAATATAATGTTAGTCATATGATTAATCA
TT
