# Supplementary material for: Rapid and efficient genetic engineering of both wild type and axenic strains of Dictyostelium discoideum
Source: PLoS One. 2018 May 30;13(5):e0196809. doi: 10.1371/journal.pone.0196809 (PMC5976153; doi:10.1371/journal.pone.0196809)
Supplement: S1 Protocol — (PDF) [file pone.0196809.s020.pdf]

Protocol for the generation of Knockouts

Knockout vector diagram

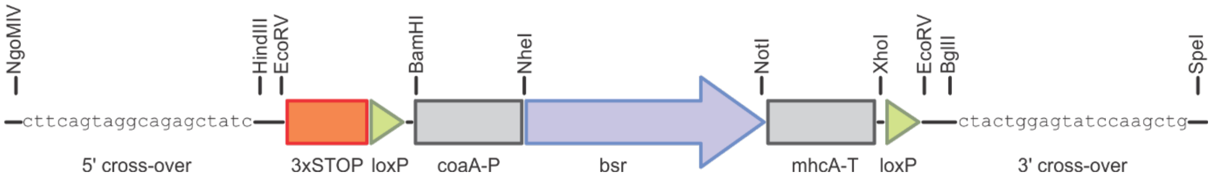

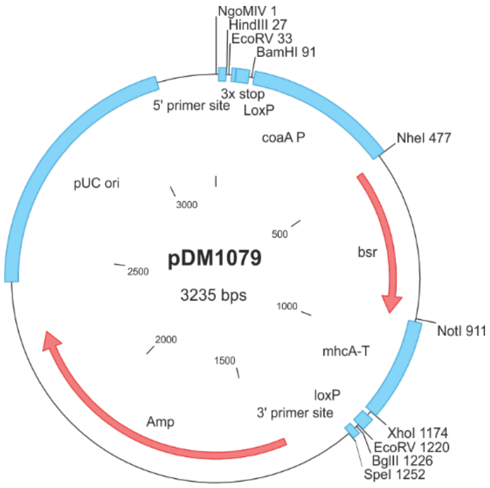

Knock out cassette vectors

blasticidin	pDM1079
nourseothricin	pDM1080
hygromycin	pDM1081
G418	pDM1082

A knockout vector can be obtained in several ways. This document describes the fastest way: PCR both recombination arms, mix products with the resistance cassette and perform a final PCR to stitch all three fragments together.

Amplification of the resistance cassette

First amplify the resistance cassette using the following primers.

forward primer: cttcagtaggcagagctatc
reverse primer: cagcttggatactccagtag

Use one of the above vectors as a template, depending on the resistance that you would like to use. The default resistance marker is hygromycin. G418 also works fine. Selection with nourseothricin is problematic and blasticidin does not work at all when selecting on bacteria.

Example protocol

<u>Template DNA:</u>
Dilute 1 µl of pDM1081 miniprep DNA with 1 ml of MiliQ water (ca. 0.2 -0.5 ng DNA).

Prepare the following program on the PCR machine:

1x	20'' 98°C
25x	10'' 98°C
	10'' 45°C
	10'' 68°C
1x	1' 68°c

- Lower annealing temperatures usually give better yield with PrimeStar Max (Takara Bio). Non-specific bands are not usually a problem
- Extension at 68°C gives better results for AT-rich template DNA. Extension rate is about 1 kb/5 sec.

Pipette together in a PCR tube (on ice):

17 µl	MiliQ water
1 µl	template DNA (1000-fold diluted miniprep)
1 µl	forward primer (10 µM stock)
1 µl	reverse primer (10 µM stock)
20 µl	PrimeStar Max

- Mix by pipetting up and down 2-3 times. Start the PCR.
- Prepare an agarose gel with wells that are able to hold 50 µl.
- Add 8 µl of loading dye and load the entire contents of the completed PCR onto the gel.
- Run the gel, excise the expected band and purify with a DNA extraction kit (Zymo Research is highly recommended)
- The purified DNA can be used for multiple KO construct PCRs.
- Store at -20°C.

Amplification of the recombination arms

The aim is to remove the gene entirely. Example gene schematic:

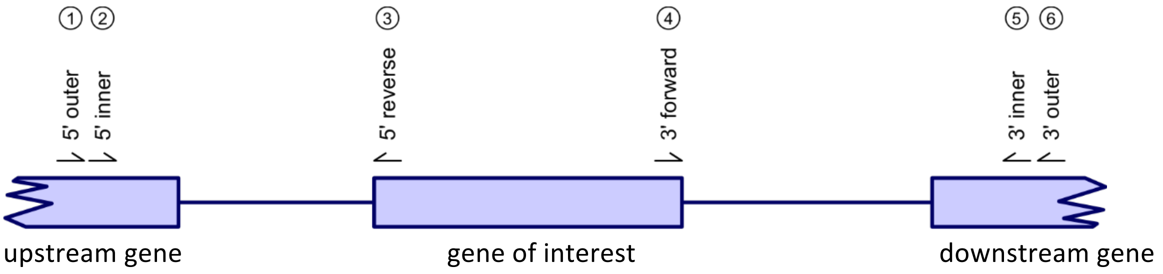

This is the general design of the primers:

(1)	5' outer	complementary<br>xxx
(2)	5' inner	PvuII complementary<br>cagctg xxx
(3)	5' reverse	5' cross-over reverse complementary<br>gatagctctgcctactgaag xxx
(4)	3' forward	3' cross-over complementary<br>ctactggagtatccaagctg xxx
(5)	3' inner	PvuII reverse complementary<br>cagctg xxx
(6)	3' outer	reverse complementary<br>xxx

- Minimal arm size is about 600 bases. Large arms may not be stable in E. coli. A practical maximum size is about 2 kb per arm. Typical arm size would be 900 bp. Each arm should contain at least 300 bases of complex sequence (usually an ORF). Arms that contain only low-complex DNA generate lower frequencies of homologous recombination (2 observations).
- Nested primers (2) and (5) lie just inside primers (1) and (6). We usually use *Pvu*II as restriction enzyme to digest the resulting KO vector. Check whether there are no *Pvu*II sites in the recombination arms. If so, then use another restriction site. *Pvu*II is also not suitable if using the G418 resistance gene (pDM1082) because it contains a *Pvu*II site.
- Ideally, primers (3) and (4) should lie just outside the gene of interest. However, this is usually low complex sequence and in practice it is only possible to design primers on the gene itself. Try and keep them as much to the ends of the gene as possible, so that the maximal amount of gene sequence is deleted.
- Amplify the 5' arm with primer (1) and (3) and the 3' arm with primer (4) and (6). Use either purified genomic DNA or lysed cells as a template (See Appendix for how to PCR on cell lysate).

Example protocol

Prepare the following program on the PCR machine:

1x	20'' 98°C
35x	10'' 98°C
	10'' 45°C (depends on annealing temp of primer)
	40'' 68°C (depends on fragment length)
1x	1' 68°c

- For complex templates such as gDNA or cDNA PrimeStar Max prefers a longer extenstion time. I usually take 40'' per kb at 68°C.
- Use 35 cycles, as the initial amplicon number will be quite low. More cycles are not necessary.
- Adjust annealing temperature and elongation time as required. Lower annealing temperatures usually give better yield with PrimeStar Max. Non-specific bands are not usually a problem.

Pipette together in a PCR tube (on ice):

17 µl	MiliQ water
1 µl	template DNA (lysed cells or purified genomic DNA)
1 µl	forward primer (10 µM stock)
1 µl	reverse primer (10 µM stock)
20 µl	PrimeStar Max

Mix by pipetting up and down 2-3 times. Start the PCR.

- Prepare an agarose gel with wells that are able to hold 50 µl.
- Add 8µl of loading dye and load the entire contents of the completed PCR onto the gel.
- Run the gel, excise the expected band and purify with a DNA extraction kit

Final PCR - construction of the KO vector

Mix purified PCR products of the resistance cassette, the 5' arm and the 3' arm and do a final PCR with the nested primers. The use of nested primers greatly reduces non-specific PCR products in the second PCR. Purify the PCR product from gel and ligate into any PCR cloning vector.

Example protocol

Prepare the following program on the PCR machine:

1x	20'' 98°C
20x	10'' 98°C
	10'' 45°C (depends on annealing temp of primer)
	20'' 68°C (depends on fragment length)
1x	1' 68°c

- 20 cycles is plenty. Extension rate is 1 kb / 5 sec for a PCR on purified fragments.
- Try and add equal (molar) amounts of each fragment. I usually guesstimate the amounts from agarose gel.
- Adjust annealing temperature and elongation time as required.

Pipette together in a PCR tube (on ice):

15 µl	MiliQ water
1 µl	gel-purified 5' arm PCR
1 µl	gel-purified resistance cassette PCR
1 µl	gel-purified 3' arm PCR
1 µl	nested forward primer (2) (10 µM stock)
1 µl	nested reverse primer (5) (10 µM stock)
20 µl	PrimeStar Max

- Mix by pipetting up and down 2-3 times. Put the samples in the PCR machine.
- Prepare an agarose gel. To avoid smearing of large amounts of high molecular weight DNA, use a low percentage gel (0.6%), use a comb with wide slots (they will hold 200 µl). Dilute the PCR product with water/loading dye until 200 µl and load entire contents on gel.
- Run the gel, excise the expected band and purify with a DNA extraction kit
- Clone the PCR fragment into a PCR ligation vector according to manufacturer's instructions (Thermo Scientific CloneJET or pDM368)
- Have the construct sequenced. Make sure there are no errors in the flanking genes.
- Make a midiprep of a correctly sequenced KO plasmid.

Linear KO construct DNA preparation

- Digest 50 µg of the KO plasmid. A typical example would be

xx µl midiprep DNA
20 µl buffer 2
10 µl *Pvu*II (100 units)
up to 200µl miliQ water
2 hours 37 °C

Ethanol precipitation of the DNA

- Add 20 µl 3M NaAc pH 5.2. Mix.
- Add 440 µl absolute ethanol. Mix by inverting tube a couple of times (mixing by pipetting is not recommended as the DNA may clog the tip).
- Incubate ~1 hour at room temperature (or overnight at -20°C for maximum yield).
- Microcentrifuge 15 mins max rpm.
- Carefully aspirate supernatant.
- Add 1 ml 70% Ethanol and mix carefully by turning tube upside down a few times.
- If pellet is loose, then centrifuge at max rpm for another 10 mins.
- Carefully aspirate supernatant.
- Short spin to collect the last droplet of ethanol at the bottom of the tube.
- Carefully aspirate the last droplet of ethanol.
- Air dry for about 20 minutes.
- Add 50 µl of water. Resuspending the pellet is sometimes difficult. Best strategy is to vortex or pipette up and down a few times. Let it rest. And repeat every 5 minutes until fully dissolved.
- Determine the yield using a spectrophotometer (eg. Nanodrop).
- Store at -20 °C

The KO Transfection

It takes about 5-7 days for colonies to come up. Selection can therefore be conveniently done over weekend and Friday is a good day to do a knockout zap.

Transfection

- Pre-chill a 2 mm gap electroporation cuvette on ice.
  (Or better, use an ice/water slurry. This increases contact area and decreases the time it takes for the cuvette to cool down).
- Pre-chill tube(s) with the DNA on ice (use maximally 2 µg per zap in no more than 5 µl volume).
- Prepare a 10cm Petri dish with 10 ml SorMC buffer + K. aerogens at OD_{600}=2
- Scrape cells from the feeding front of an SM agar plate with a bacterial lawn or use a clearing plate. You need ~2-5 $\cdot 10^6$ per zap (That is about a single 3 cm scrape with the blue disposable inoculation loops).
- Transfer cells to an Eppendorf tube with 1 ml H40.
- Pipet cells up and down to get them into suspension (cells can be counted at this point, but exact numbers are not critical).
- Flash-spin cells 2 seconds 10,000 xg (fast and convenient and the cells don't mind. 3' @ 300xg also works).
- Resuspend to a density of about ~2-5 $\cdot 10^6$ cells per 100 µl in H40.
- Place on ice and let cells cool down. Make sure cells are ice-cold before proceeding.

- Add 100 µl cells to the tube with DNA. Mix by pipetting.
- Transfer cell/DNA mixture to the electroporation cuvette.
- Zap the cells using the following square-wave settings.
  - 350V
  - 8ms
  - 2 pulses
  - 1 second pulse interval
- Without delay, transfer the cells to the 10cm Petri dish (use a gel-loading tip to get the cells out. Normal tips are too wide to reach to the bottom of the cuvette). Expect round cells and >90% survival. Confluency will be about 20-40%. Let cells recover on plate for 5 hours (they may double once during recovery).

Setting up 96-wells plates for selection

- Get six flat-bottom 96-wells plates and label them.
- Resuspend transfected cells on the 10cm dish after 5 hours of recovery and make up the following suspensions in a 50 ml Falcon tube:

	High	Medium	Low
Transfected cells	9 ml	900 µl	90 µl
Bacteria	600 µl	600µl	600µl
selection marker	xx µl	xx µl	xx µl
SorMC buffer	20.4 ml	28.5 ml	29.3 ml

Aliquot 150 µl of the "High", "Medium" and "Low" suspensions across two 96-wells plates each.

Cells respond in different ways to different selection markers

- 10 µg/ml G418: Cells promptly stop doubling. After 24 hours cells have either lysed or are rounded up and floating. After 48 hours all cells are lysed.
- 100 µg/ml Hygromycin: Cells round up and stay rounded for about 3 days after which they start lysing. After 5 days all cells have lysed. Sometimes cells continue growth for a while before rounding up. I am not sure what causes this. It may be that the hygromycin stock degrades when kept in the fridge for too long. Anyway, this residual growth does not affect the experiment.

Checking for knockouts

It is most convenient to transfer these cells to a 12-well plate. These plates allow good visualisation on a phase-contrast microscope. 24-wells plates have too small wells. Wells will overgrow fast and due to the meniscus, visualisation of cells is difficult. 6-wells are too large.

Screen the plates for colonies. Number all wells with colonies. Tight, dense colonies are usually best. Disperse colonies are not always properly resistant and may stop growing when transferred to a new well. Round colonies have the best odds of being clonal. Avoid dumbbell-shaped ones if you have a choice, as those may not be clonal. Start the numbering of wells from the plate with the lowest number of colonies (We prefer these colonies as they have the highest chance of being clonal, have the lowest number of non-transformed, dying cells and the lowest amount of input DNA floating around).

Example protocol (for 23 colonies)

Step 1: transfer to a bigger well

This step is not absolutely essential. PCR can also be done directly from 96-wells plates, but it improves the clarity of the PCR as it reduces the number of dead/dying cells that will give rise to wild-type bands.

Prepare plates:

Get two 12-wells plates and label them.

Prepare medium:

25 ml SorMC
500 µl bacteria
25 µl of selection antibiotic
Aliquot 1 ml in each well.

- Set a P200 pipette at 100µl. Pipette the contents of a well with a colony up and down a few times to get the cells into suspension.
- Transfer 100 µl to the 12-well plate.
- Repeat for all other colonies.
- Put $1 \cdot 10^5$ wild type cells in the last well (well should be about 5-10% confluent).
- Let cells grow overnight.

Step 2: perform a test PCR

- Check that the cells have grown as expected overnight. Wells need to be >10% confluent for the PCR to be reproducibly successful.
- Resuspend cells in their own medium using a P1000 pipette.
- Take 20 µl of cells out and use for a colony PCR (see appendix 1 and 2)

The cell lysate is used to perform 3 PCRs:

PCR	forward primer	reverse primer
5' arm	outer primer (1)	5' cross-over sequence<br>gatagctctgcctactgaag
3' arm	3' cross-over sequence<br>ctactggagtatccaagctg	outer primer (6)
entire locus	outer primer (1)	outer primer (6)

We usually use the OneTaq polymerase 2x mix from NEB for PCR. If both 5' arm PCR and 3' arm PCR give expected results, then that should be strong evidence that the gene is knocked out. PCR across entire locus is even better evidence than the single arms, but only feasible with OneTaq polymerase if the expected band size is lower than 5 kb. If the band is larger, then use another enzyme that is able to handle large templates.

The re-use of the outer primers of the nested pair that was used to generate KO construct is nice as the primers are proven to work and economical. Ensure that their sequence is not present in the used KO construct, or random integrants will also give positive bands for the test PCR!

Appendix 1A - Preparation of lysed Dicty cells for PCR

adapted from Charette S.J. and Cosson P. (2004) Preparation of genomic DNA from Dictyostelium discoideum for PCR analysis. Biotechniques. 36:574-5.

Materials:

Lysis Buffer
50 mM KCl
10 mM TRIS pH 8.3
2.5 mM MgCl2
0.45% NP40
0.45% Tween 20
Add 1 µl of 20 µg/µl of Proteinase K for every 20 µl of Lysis Buffer <u>immediately before use</u> (Proteinase K is not stable in lysis buffer).

Protocol

- Harvest sufficient cells for the PCR reaction. Minimal cell density should be $5x10^4$ cells/ml and maximally around $2x10^7$ cells/ml. There is no need to wash cells. SorMC + bacteria does not inhibit DNA polymerase.
  - confluent 96-wells plate in 100 µl --> $1x10^6$ cells/ml
  - confluent 24-wells plate in 500 µl --> $1x10^6$ cells/ml
  - confluent 6-wells plate in 2 ml --> $1x10^6$ cells/ml
  - confluent 9 cm plate in 10 ml --> $2x10^6$ cells/ml

- Pipette 20 µl cells into a PCR tube
- Add 20 µl lysis buffer. Mix.
- Incubate 1 min at 95°C (using a PCR machine) to inactivate the proteinase K.
- Use immediately or store at -20°C.

Appendix 1B - Preparation of clean gDNA of Dicty cells for PCR

Especially if the product of the entire locus is very large we recommend to use the Zymo Research Quick DNA Mini Prep Kit to isolate clean high molecular DNA for the screening PCRs.

- Harvest the cells of a confluent 12 well plate
- Wash once with KK2
- Resuspend cell pellet in 600 µl genomic lysis buffer
- Transfer the lysate on the column and spin down at 10,000xg for 1 minute
- Discard flow through and add 200 µl DNA-pre-wash-buffer
- spin down at 10,000 xg for 1 minute
- Discard flow through and add 500 µl DNA-wash-buffer
- spin down at 10,000 xg for 1 minute
- place column on a fresh 1.5 ml tube and elute DNA in 50 µl elution buffer
- incubate at room temperature for 5 min
- spin down at 10,000xg for 1 minute
- the isolate DNA can be stored at -20°C or 4 °C

Appendix 2 - Colony PCR with NEB's OneTaq DNA polymerase

OneTaq from NEB is an economical DNA polymerase. Cost per 40 µl reaction is 20p (KOD Hot start is 50p). It is robust up to about 4-6 kb. It is conveniently available as a 2x mix (even contains the loading dye already), simplifying the pipetting steps.

The Tm for primers in Taq-based buffers is quite low compared to other polymerases. Use the Tm calculator from NEB's website to find the appropriate Tm (tmcalculator.neb.com). Polymerization rate of OneTaq DNA polymerase is approximately 1 kb/minute.

General PCR program:

1x	30 '', 95°C
35x	10 '', 95°C
	10 '', 45°C (depends on annealing temp of primer)
	1 min, 68°C (depends on fragment length)
1x	2 min, 68°C

Pipetting together the PCR

- Transfer 2 µl of cell lysate or 2 µl of clean DNA to each PCR tube. Put tubes on ice.
- Prepare the PCR master mix on ice (enough for 24 PCR reactions in this example)

0.8 µl forward primer (10 µM)	x 25 = 20 µl
0.8 µl reverse primer(10 µM)	x 25 = 20 µl
16.4 µl MilliQ water	x 25 = 410 µl
20 µl OneTaq	x 25 = 500 µl

- Add 38 µl of master mix to each tube and start the PCR.
- Put 20 µl of PCR product on gel.
