# Supplementary material for: Rapid and efficient genetic engineering of both wild type and axenic strains of Dictyostelium discoideum
Source: PLoS One. 2018 May 30;13(5):e0196809. doi: 10.1371/journal.pone.0196809 (PMC5976153; doi:10.1371/journal.pone.0196809)
Supplement: S2 Protocol — (PDF) [file pone.0196809.s021.pdf]

Protocol for the preparation of food bacteria

Bacteria

Suspension of *K. aerogenes* or *pneumoniae* in SorMC at OD_{600}=100

- Innoculate 1 liter of LB with a single colony of *K. aerogenes* or *pneumoniae*
- Grow overnight at 37°C
- Spin bacteria down
  - centrifuge: Avanti J-26 XP
  - rotor: Fiberlite F10BCI 6x500 (code F500)
  - tubes: 500ml PC tube (clear) max volume: 465ml, 10,000 max rpm
  - settings: 20 mins 6,000 rpm (they are difficult to spin down!)
- Wash cells once in SorMC.
- Resuspend cells in SorMC at OD_{600}=100. This will typically be about 25 ml.
- Store at 4°C. (Will be usable for several months)
