# Supplementary material for: Rapid and efficient genetic engineering of both wild type and axenic strains of Dictyostelium discoideum
Source: PLoS One. 2018 May 30;13(5):e0196809. doi: 10.1371/journal.pone.0196809 (PMC5976153; doi:10.1371/journal.pone.0196809)
Supplement: S3 Protocol — (PDF) [file pone.0196809.s022.pdf]

REMI expression

DNA preparation

- Digest about 20 µg of plasmid DNA with *Bam*HI.
- Ethanol precipitate and resuspend in 20 µl of H_2O or TE buffer.
- Check DNA concentration on a nanodrop.

Transfection

- Pre-chill a 2mm gap electroporation cuvette on ice.
- Prepare two 10cm Petri dish (tissue culture treated) with 10 ml SorMC buffer + *K. aerogens* at OD_{600}=2
- Scrape ~ $4 \cdot 10^6$ cells from the feeding front of an SM agar plate with a bacterial lawn or use a clearing plate.
- Transfer cells to an Eppendorf tube with 1 ml H40.
- Pipet cells up and down to get them into suspension (cells can be counted at this point, but exact numbers are not critical).
- Flash-spin cells 2 seconds 10,000 xg (or for 3 min @ 300 xg)
- Wash cells once in H40.
- Resuspend cells in 200 µl H40.
- Place on ice and let cells cool down.
- Prepare two tubes with 2 µg of BamHI digested DNA on ice.
- Prepare one tube with 5 units DpnII on ice.

- Add the cells to the DNA. Mix by pipetting (this is the no-enzyme control).
- Transfer DNA/cell mixture to the electroporation cuvette.
- Zap the cells using the following square-wave settings. Make sure cells are ice-cold during the zap:

- 350V
- 8ms
- 2 pulses
- 1 second pulse interval

Without delay, transfer the cells to the 10cm Petri dish (tissue culture treated).

- Add the cells to the DNA. Mix by pipetting.
- Add the DNA/cell suspension to the restriction enzyme. Mix by pipetting.
- Transfer the mixture to the electroporation cuvette (you can re-use the previous cuvette).
- Zap as above.

Without delay, transfer the cells to the 10cm Petri dish (tissue culture treated). Expect round cells and >90% survival. Optimal seeding density is about 10-20%. Higher densities have the chance of aggregating before the selection marker is becoming effective. Cells will recover their normal morphology in about 30 minutes.

After 5 hours, add selection marker.
Hygromycin 100 µg/ml

The yield has been variable. We got clones numbers between 50 and 2000 for the positive plate. The best is to dispense the transfected cells in a 1:10 dilution series across three 96-wells plates to ensure single colonies per well. Expect clones after 5-7 days.
