# Supplementary material for: Rapid and efficient genetic engineering of both wild type and axenic strains of Dictyostelium discoideum
Source: PLoS One. 2018 May 30;13(5):e0196809. doi: 10.1371/journal.pone.0196809 (PMC5976153; doi:10.1371/journal.pone.0196809)
Supplement: S4 Protocol — (PDF) [file pone.0196809.s023.pdf]

Extrachromosomal expression

DNA

- Use about 200 ng to 2 µg of an extrachromosomal plasmid (DNA solved in H_2O or TE buffer)

Transfection

- Pre-chill a 2mm gap electroporation cuvette on ice
- Prepare a 10cm Petri dish (tissue culture treated) with 10 ml SorMC buffer + *K. aerogens* at OD_{600}=2
- Scrape ~ $2 \cdot 10^6$ cells from the feeding front of an SM agar plate with a bacterial lawn or use a clearing plate
- Transfer cells to an Eppendorf tube with 1 ml H40
- Pipet cells up and down to get them into suspension (cells can be counted at this point, but exact numbers are not critical)
- Flash-spin cells 2 seconds 10,000 xg (or for 3 min @ 300 xg)
- Wash cells once in H40
- Resuspend cells in 100 µl H40
- Place on ice and let cells cool down
- Prepare a tube with 200 ng -2 µg of plasmid

- Add the cells to the DNA. Mix by pipetting
- Transfer DNA/cell mixture to the electroporation cuvette.
- electroporate the cells using the following square-wave settings. Make sure cells are ice-cold during the zap:

- 350V
- 8ms
- 2 pulses
- 1 second pulse interval

Without any delay, transfer the cells to the 10cm Petri dish (tissue culture treated). Expect round cells and >90% survival. Optimal seeding density is about 10-20%. Higher densities have the chance of aggregating before the selection marker is becoming effective. Cells will recover their normal morphology in about 30 minutes.

After 5 hours, add selection marker.

Hygromycin	100 µg/ml
G418	10 µg/ml

Expect clones after 3-4 days.
